# Supplementary material for: Identification of Shemin pathway genes for tetrapyrrole biosynthesis in bacteriophage sequences from aquatic environments
Source: Nat Commun. 2024 Oct 15;15:8783. doi: 10.1038/s41467-024-52726-3 (PMC11480375; doi:10.1038/s41467-024-52726-3)
Supplement: Supplementary file 1 — Supplementary Information [file 41467_2024_52726_MOESM1_ESM.pdf]

SUPPLEMENTAL MATERIAL

Identification of Shemin pathway genes for tetrapyrrole biosynthesis in bacteriophage sequences from aquatic environments

Helen Wegner[1,7], Sheila Roitman[2,3,7], Anne Kupczok[4], Vanessa Braun[1], Jason Nicolas Woodhouse[5], Hans-Peter Grossart[5,6], Susanne Zehner[1], Oded Béjà[2], Nicole Frankenberg-Dinkel[1*]

[1]Department of Biology, Microbiology, University Kaiserslautern-Landau, Kaiserslautern, Germany

[2]Faculty of Biology, Technion-Israel Institute of Technology, Haifa, Israel

[3]Department of Molecular Biology, Max Planck Institute for Biology, Tübingen, Germany

[4]Department of Plant Sciences, Bioinformatics, Wageningen University & Research, Wageningen, Netherlands

[5]Department of Plankton and Microbial Ecology, Leibniz Institute of Freshwater Ecology and Inland Fisheries, Stechlin, Germany

[6]Institute of Biochemistry and Biology, Potsdam University, Potsdam, Germany

[7]These authors contributed equally: Helen Wegner, Sheila Roitman

Figure S1.

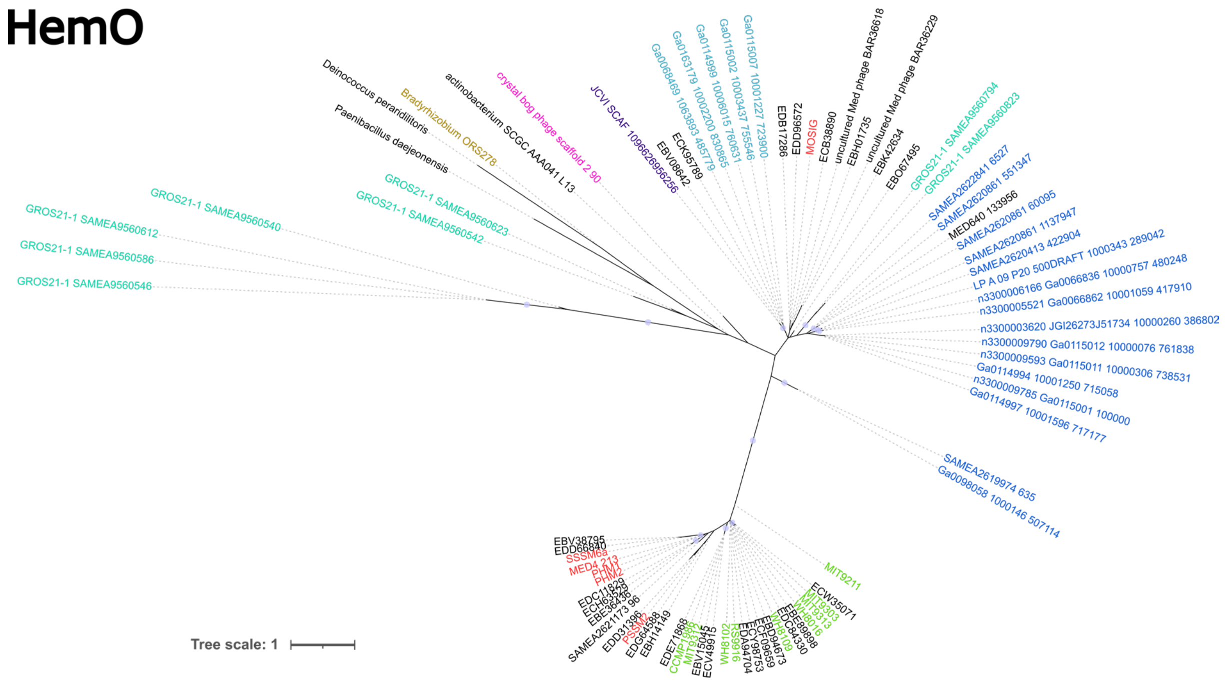

Figure S1. Phylogenetic analyses of HemO protein sequences including sequences derived from contigs containing v*alaS*. Maximum likelihood phylogenetic tree for HemO. Cyanobacterial strains are marked in light green; cultured phages are coloured red; black names denote non-cyanobacteria and metagenomic contigs of uncertain origin. The *Bradyrhizobium* bacterium containing the three gene-cassette is marked in gold. The CB_2 phage chosen for experimental characterisation is coloured pink. The previously characterised *hemO_pcyX* cassette [1] is marked in purple. Metagenomically retrieved contigs from this project are colour coded according to the bars in Figure 2. Circles represent bootstrap values >0.9. The scale bar indicates the average number of amino-acid substitutions per site.

Figure S2.

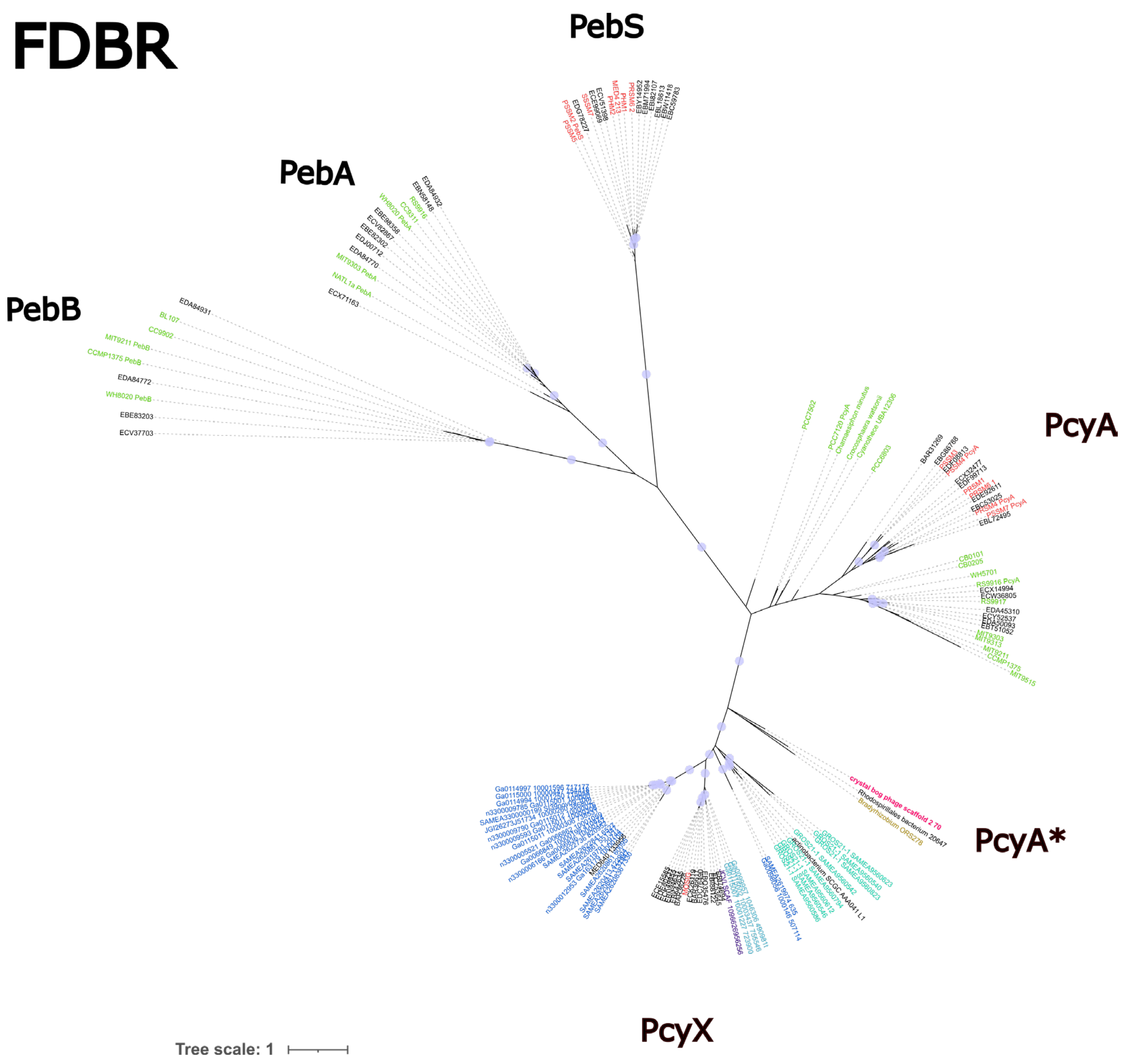

Figure S2. Phylogenetic analyses of ferredoxin-dependent bilin reductases (FDBRs) including sequences from contigs containing v*alaS*. Maximum likelihood phylogenetic tree for FDBRs. Cyanobacterial strains are marked in light green; cultured phages are coloured red; black names denote non-cyanobacteria and metagenomic contigs of uncertain origin. The *Bradyrhizobium* bacterium containing the three gene-cassette is marked in gold. The CB_2 phage chosen for experimental characterisation is coloured pink. The previously characterised cassette [1] is marked in purple. Metagenomically retrieved contigs from this project are colour coded according to the bars in Figure 2. Circles represent bootstrap values >0.9. The scale bar indicates the average number of amino-acid substitutions per site. PcyA* marks a subgroup of PcyA enzymes including the one investigated in this study as well as the one from *Bradyrhizobium* sp. ORS278 investigated earlier [2,3].

Figure S3

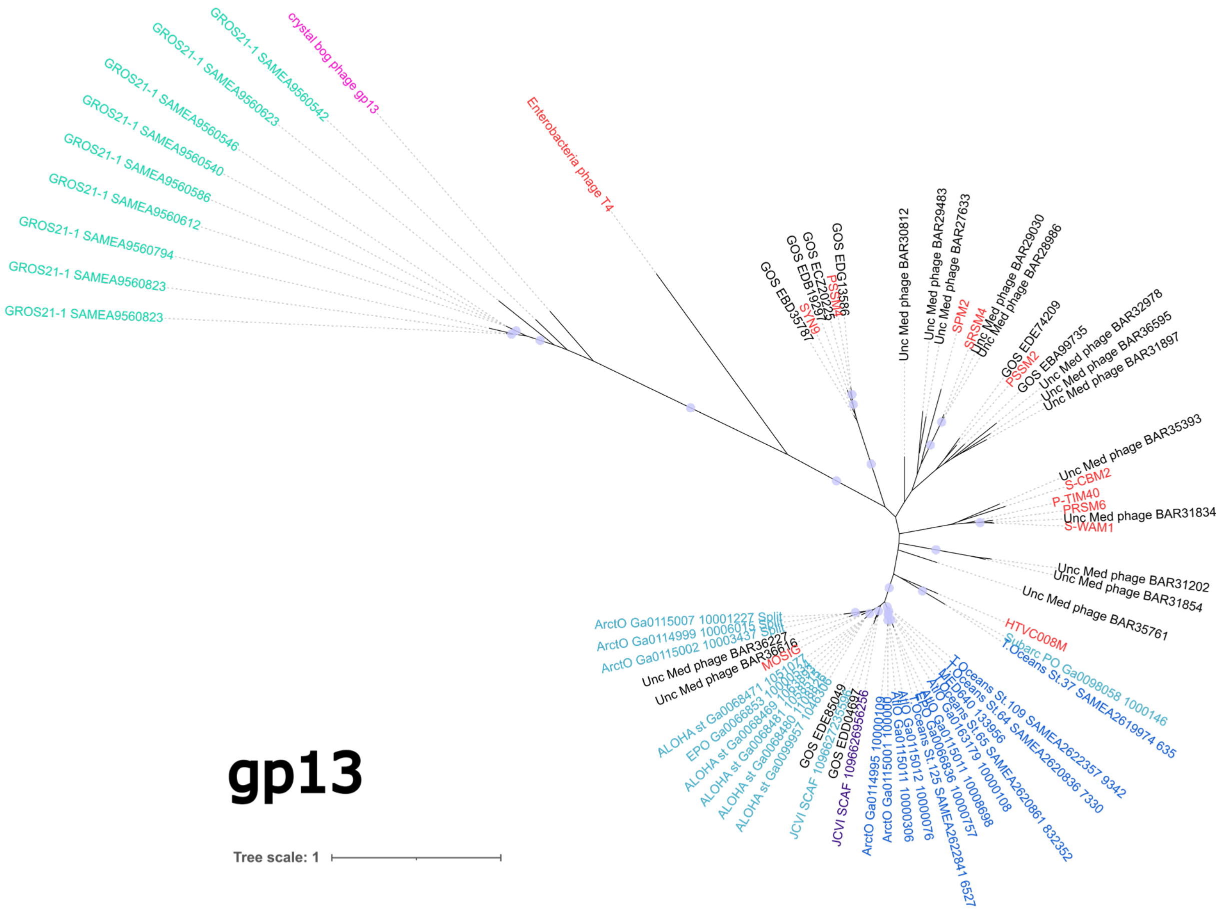

Figure S3. Phylogenetic analyses based on gp13 protein sequences. Maximum likelihood phylogenetic tree for gp13. Annotation in the phylogenetic tree represent gp13 source contigs/phages. Cultured phages are coloured red; black names denote metagenomic contigs of uncertain origin. The CB_2 phage chosen for experimental characterisation is coloured pink. The previously characterised cassette containing a *hemO_pcyX* [1] is marked in purple. Metagenomically retrieved contigs from this project are colour coded according to the bars in Figure 2. Circles represent bootstrap values >0.9. The scale bar indicates the average number of amino-acid substitutions per site.

Figure S4.

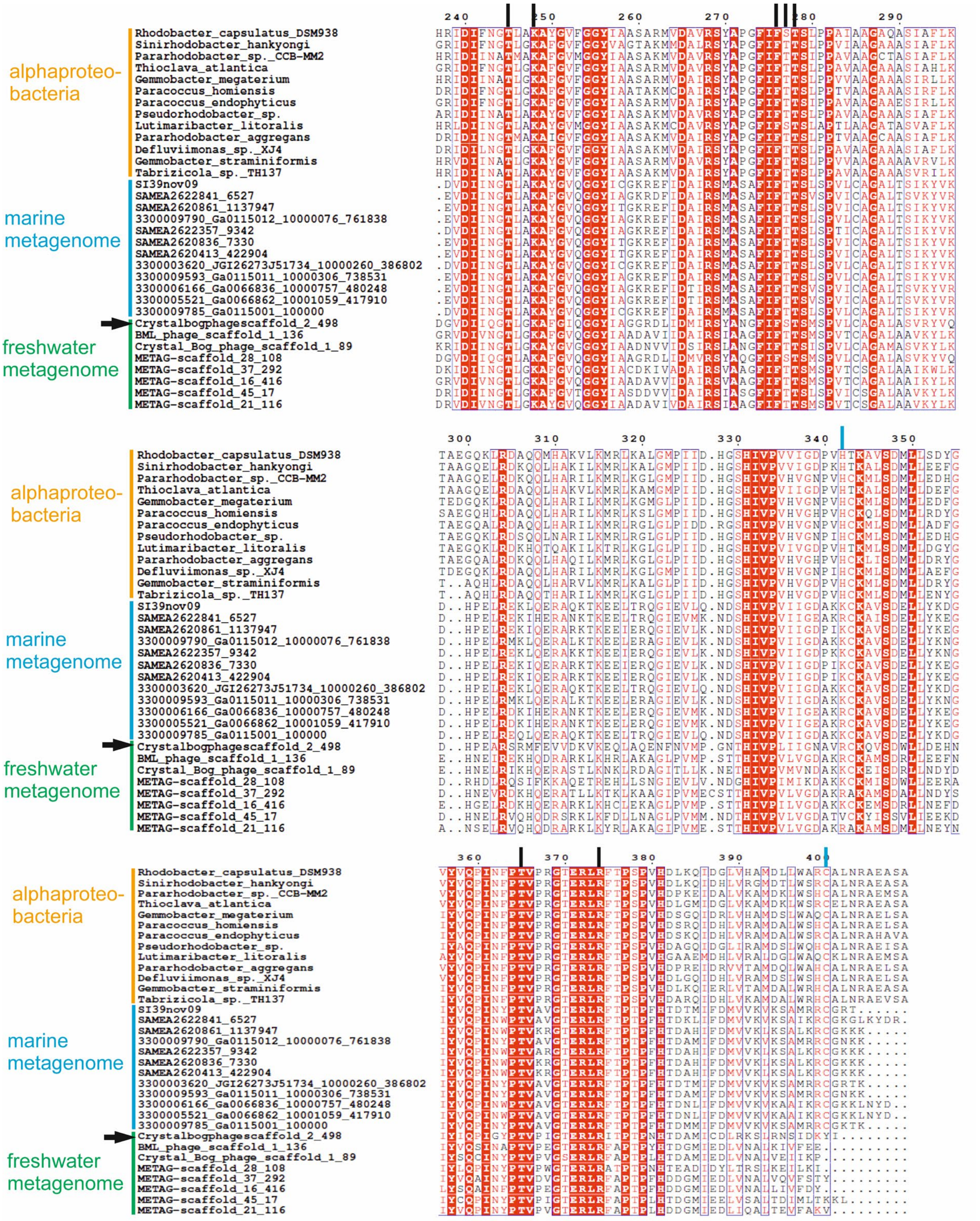

Figure S4. Partial amino acid sequence alignment of AlaS sequences from alphaproteobacteria and viral metagenomes revealed conserved catalytic residues. Reference sequences (shown in yellow) from the NCBI database were aligned with sequences from multiple sampling sites (blue and green) using Clustal Omega and ESPript 3. Highly conserved amino acids are highlighted in a red square with white lettering, similar amino acids are represented by a white box with red lettering, and non-similar amino acids are indicated by black lettering. Catalytic important amino acids of RcA, previously characterised in [4], are denoted by black lines at the top. Heme axial ligands of *Caulobacter crescentus* AlaS are

indicated with blue lines at the top [5]. Marked with an arrow is the sequence used in this publication for characterisation. Full amino acid sequence alignment in Supplementary Data 5.

Figure S5.

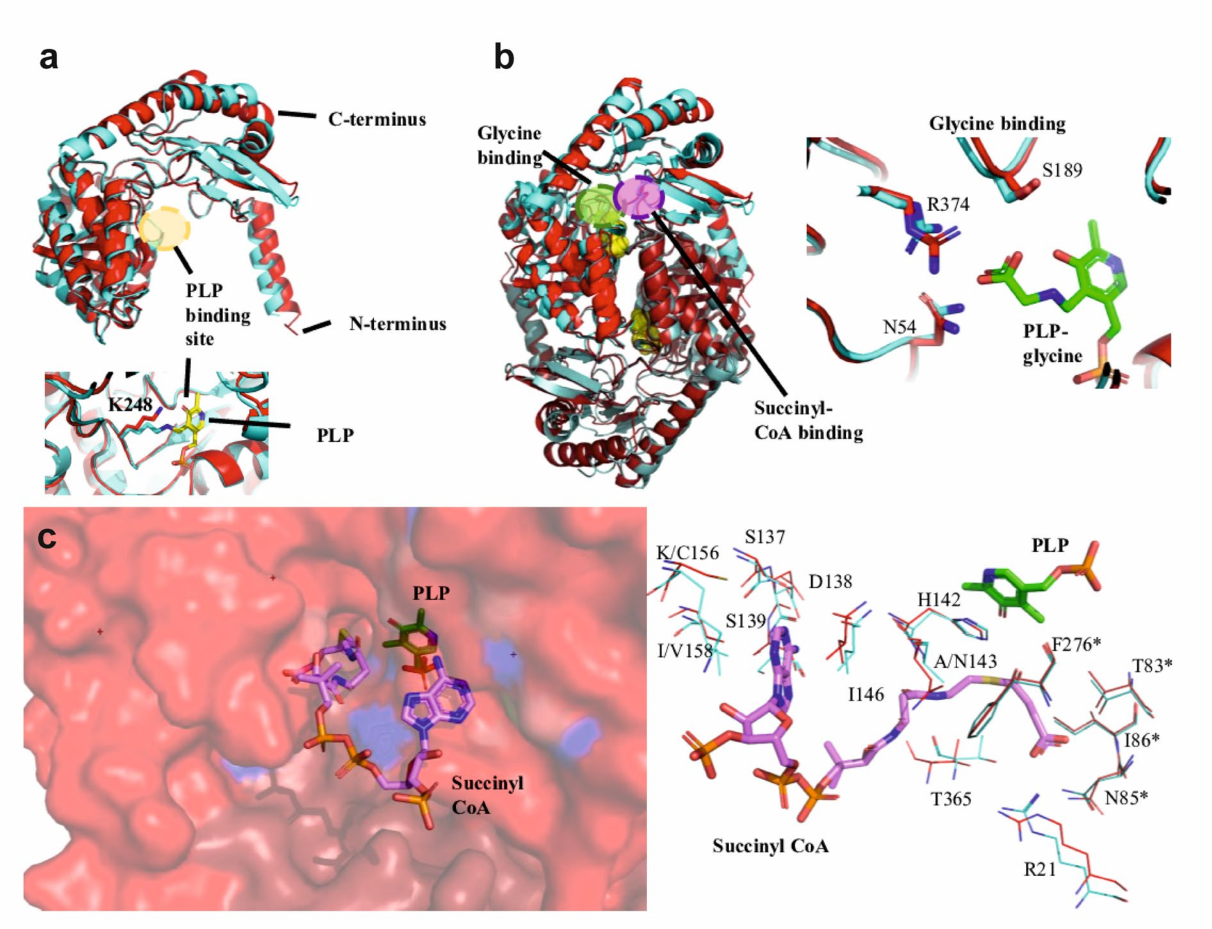

Fig. S5. Structural modelling of vAlaS revealed high similarity to bacterial AlaS.
a. Overlay model of monomeric AlaS from *Rhodobacter capsulatus* (RcA, blue, RCSB PDB: 2bwn) and the model of vAlaS predicted via AlphaFold (red), shows high structural similarity. The cofactor binding site of pyridoxal phosphate (PLP) is indicated with a yellow circle. A magnified view shows binding of PLP (yellow) onto amino acid K248 (sticks in blue/red) forming a Schiff's base linkage. **b.** Homodimeric view of vAlaS with RcA overlay, indicated are glycine binding (green circle) and succinyl-CoA binding (purple circle) and an enhanced view of the three important amino acid residues of the glycine binding (N54, S189, R374) onto the PLP-glycine intermediate (green chemical structure). **c.** The substrate channel for succinyl-CoA binding is visualised, with the ribose moiety outside the enzyme (purple) and PLP (green) inside (left picture). The model on the right side highlights all important amino acids for succinyl-CoA binding, in red (RcA)/blue (vAlaS) sticks. Some deviations of vAlaS are observed at position 143, 156, and 158. Asterisks indicate amino acids from the second monomer.

Figure S6.

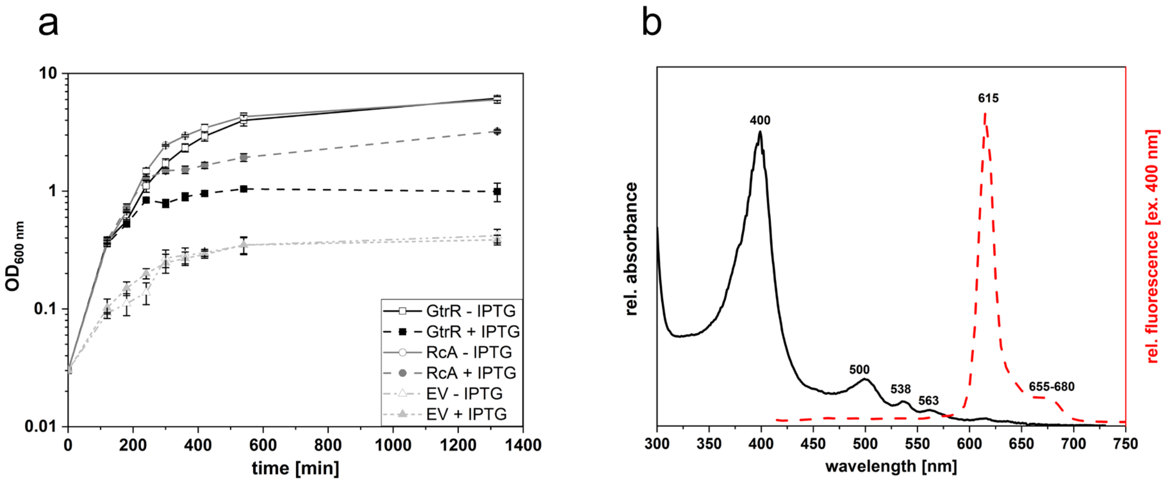

Fig. S6. a. Functional complementation of ALA-auxotrophic *E. coli* strain ST18 via plasmid-based complementation. *E. coli* growth was measured in the absence (open symbol) or presence (filled symbol) of gene expression inducer IPTG for *tac* promotor controlled C4 or C5 pathway genes in comparison to the empty vector control. GtrR (black squares), RcA (grey circle), empty vector (light grey triangle). The data shown represent mean values for three biological replicates for each strain with two technical replicates (n=6). The standard deviation is displayed as error bars for each time point. **b.** Viral AlaS overproduction in *E. coli* leads to porphyrin accumulation. Fluorescence excitation (615 nm emission) of cell-free lysate (black, solid line) and fluorescence emission (red, dashed line) measurement, show Soret and Q band, while fluorescence excitation at 400 nm show Stokes shift, indicating the presence of porphyrins in the cell lysate. Source data are provided as a Source Data file.

Figure S7.

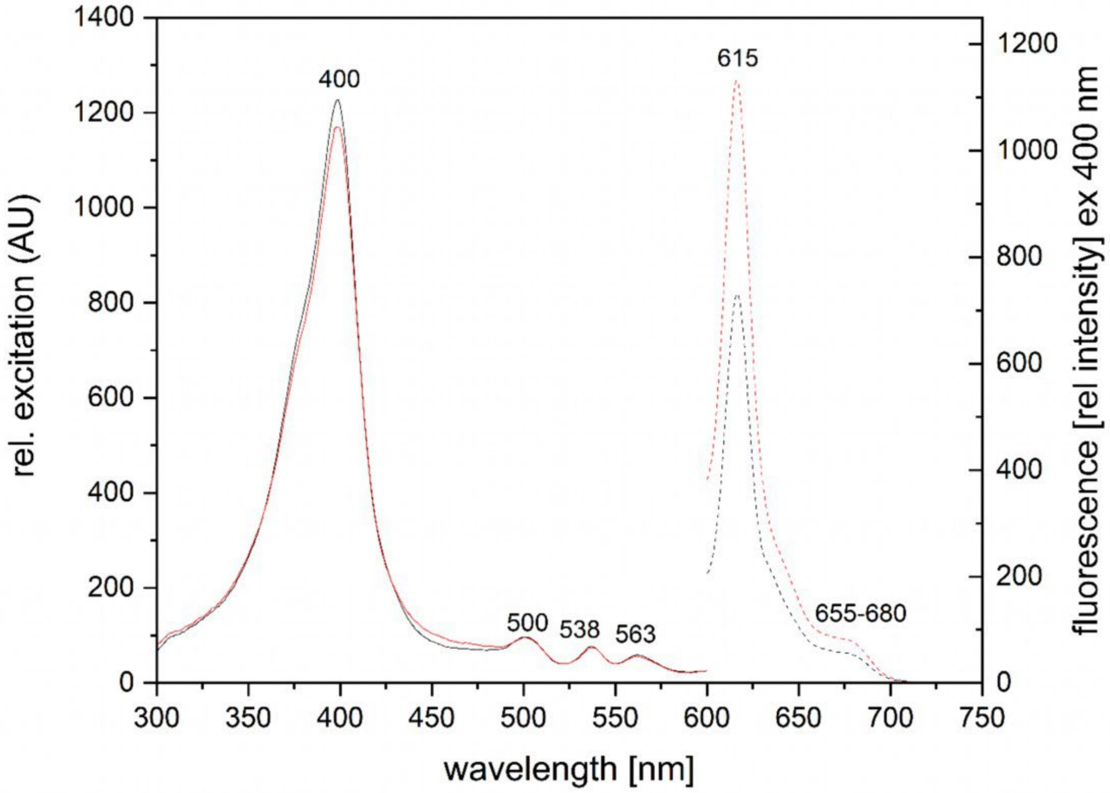

Fig. S7. Overproduction of three viral tetrapyrrole genes in *E. coli* leads to porphyrin accumulation. Cell lysates of *E. coli* strain BL21(DE3) plasmid encoded vAlaS (black) and vAlaS + vHo1 + vPebS (red) [6] were analysed for fluorescence excitation (615 nm emission) of cell-free lysate (solid lines) and fluorescence emission (dashed line).
Typical porphyrin spectra were observed for both lysates with excitation maximum at 400 nm and Q bands at 500 nm, 538 nm and 563 nm. Fluorescence emission was detected after excitation at 400 nm with a characteristic maximum at 615 nm. Source data are provided as a Source Data file.

Supplemental References

1 Ledermann, B., Beja, O. & Frankenberg-Dinkel, N. New biosynthetic pathway for pink pigments from uncultured oceanic viruses. *Environ Microbiol* **18**, 4337-4347, doi:10.1111/1462-2920.13290 (2016).

2 Ledermann, B. *et al.* Evolution and molecular mechanism of four-electron reducing ferredoxin-dependent bilin reductases from oceanic phages. *FEBS J* **285**, 339-356, doi:10.1111/febs.14341 (2018).

3 Jaubert, M. *et al.* A singular bacteriophytochrome acquired by lateral gene transfer. *J Biol Chem* **282**, 7320-7328, doi:10.1074/jbc.M611173200 (2007).

4 Astner, I. *et al.* Crystal structure of 5-aminolevulinate synthase, the first enzyme of heme biosynthesis, and its link to XLSA in humans. *EMBO J.* **24**, 3166-3177, doi:10.1038/sj.emboj.7600792 (2005).

5 Ikushiro, H. *et al.* Heme-dependent Inactivation of 5-Aminolevulinate Synthase from Caulobacter crescentus. *Sci Rep* **8**, 14228, doi:10.1038/s41598-018-32591-z (2018).

6 Dammeyer, T., Bagby, S. C., Sullivan, M. B., Chisholm, S. W. & Frankenberg-Dinkel, N. Efficient phage-mediated pigment biosynthesis in oceanic cyanobacteria. *Curr Biol* **18**, 442-448, doi:10.1016/j.cub.2008.02.067 (2008).
