# Supplementary material for: Identification of Shemin pathway genes for tetrapyrrole biosynthesis in bacteriophage sequences from aquatic environments
Source: Nat Commun. 2024 Oct 15;15:8783. doi: 10.1038/s41467-024-52726-3 (PMC11480375; doi:10.1038/s41467-024-52726-3)
Supplement: Supplementary file 3 — Description of Additional Supplementary Files [file 41467_2024_52726_MOESM3_ESM.docx]

Description of Additional Supplementary Files

**File Name:** Supplementary Data 1

**Description:** Contigs.

**File Name:** Supplementary Data 2

**Description:** Database links.

**File Name:** Supplementary Data 3

**Description:** Tara counts.

**File Name:** Supplementary Data 4

**Description:** FASTA sequences AlaS.

**File Name:** Supplementary Data 5

**Description:** AlaS alignment.

**File Name:** Supplementary Data 6

**Description:** FASTA Protein sequences (AlaSA, HemO, FDBRs, gp13).

**File Name:** Supplementary Data 7

**Description:** vAlaS ColabFold PDB file.
