# Supplementary material for: Identification of Shemin pathway genes for tetrapyrrole biosynthesis in bacteriophage sequences from aquatic environments
Source: Nat Commun. 2024 Oct 15;15:8783. doi: 10.1038/s41467-024-52726-3 (PMC11480375; doi:10.1038/s41467-024-52726-3)
Supplement: Supplementary file 7 — Supplementary Dataset 4 [file 41467_2024_52726_MOESM7_ESM.docx]

>Igi|133597819|gb|AACY020113167.1| Marine metagenome 1096626131462, whole genome shotgun sequence

ACAATTAAATCTTCGTAAAGTCTTTTTCGTTTTGGTTTCCCAATGCCGTTGCCACCTTGAAAGTAATGATTAACTGGCAT

AGCATTATCCTATCATCATTGCAGGATTTAATTCGTAAGTAGTTCTAATTTCAGTTTCTAATTTTTCTAAATCTTGTAGT

GCTTCTGAATATATTTGTTGACCATTTAAAGTTACTCCGCCAACCATTGCAACCCCACCAAATTTAGATAAGTTAGCACC

CCATTGTTTTTTAAATAAAGCAGTAGTATATCTTTTTAAATATATGTCATTCCAAACATCTGTATAACTATTTGGATCTA

ATTTTCTATAACATTCAATAACTATCCATTCATCTGTTTCTAAATCATTTGTCCAATCCATATCAATGTATAATCTATTA

TCGTGTTGATTAAATCTTAATGGTTTTTCACCTACAAGTATATGATCTAAAAAATCTAAATGTCTTAATACAACATCATA

ATTAATCACACTTGTTGAAGAAAAATCATACAAGTCATTTAATCTTAATTGGTATCTTACATCAAATAAGTTTAGATTAC

CTTTATCTGAAAAAGGAAATATATTAATTACAGAAATAATACTTTGAGGTACTACTAGGAAATTTCTATCTTCATACCAA

GTTGTAGATACAGAATTGTCTTTTAAGTCTGTTGCAGATTCAGTTTCACCATTTATAGCAGATAAACGAGTTTTATCAGC

AGCAGTTAATTTGTATTTTAAATAAGTTCTTCTAATACCATCATAATGATATTGTTGAAAATACTGAACCGCCTCGTCTA

TTCTATCTTCTAACTGGTCGTCATCTACATTTATTTCAATGACTGGTTTTCCTAATGCTCTTAATGAGTATTGCTTTAAT

GTTTCTCTTGTTGATGGTTCTGCCATAAATCCTCGTCTTTAGTACTATTTATAAGAATTATTTAATGGTAGGAAAGAGAT

TATCAGCACAAAATAACTTAATATCTTCTTCAGGTAAACCAAGTGATTTCATTGTTCTTGGCGTGTGAGGATTTTTTTGT

TGATTTTCACAATAAAAATTTTGTGCTCTTATGACATCTTCCTCGTTAGAATCACTATTAAAATGACCTATTTTATCAAT

ATATGCGTTTAAATTTGATACTGCCATAGTACAAATTTGATTTAACTCTCTCTCTTCCTGTACATTCCCAGCGGCTATCA

TACCTTCACTAAAGATTGCTTTTGCCCAATCAGGTAATTCTCTTACCTTTGACGGTTTGTACCATTTATTTTCTTCTATA

AACCATCTTGTTAATGGGTGTTCTTTCTTTAATAGTGGTGAGAAGTCGTGGAATGCGCCAGTAACCTTTTTCTTACCTGC

GATAATATCAAAACCGTAAATAGGTCCGCCATTTTTCTTCATAGGAAATAGACAAATATGTGCCATCCATAATCCTTTTG

TTTCTCTAGCGTCAACTACATCAACGTGGGCTCGTCTAATACTCATATTAGACCAAGTACGGTTTGTCCAACCAGGTTTA

TTAAATCTATCCATACCTGGTTCGTTATATTCAATTAAATGTTTATCTAATACTTCTATAATTTCTTTTTCTAATTTGAT

TAATCTTTCCCAAATCATTCAACAACCTCATCTGTCAACACAAGAGGTTTGTTCATTTCATTCATCTCTTTAAATAAATT

TGTTGCAGATTCAAAACAATATTCAACTTCACTTAATACATTTACTTGATACGTATTTAAATATTCGTGTATAATTTCTT

TTACTATTCTTTTCCATTCTTTTGCATTTGCGCCAAAGTCGTAATATCTTTTGACAGGTACTTTTTTTGCAATCATTTGA

CCACCTGATAAATCGCCTAAATGTCTTACATAGATATGAGCATATAGTTTTTCAGGATCATCTTGTATAGATTCTATATG

TTCTATGTACTTCTTTGTACTTTCAGTTATTCGTGGAGGTTGTTTAGGGTCAGGCCACAACTTTAAATAATCTTTAGATA

TATTTTCTGCTCTTTGTAGACCTGGTGTCTGTCTGAATAAATCATTGTGCATTCCATACTTTTCTAGTACAGAATAACAT

TGTAATTGATTATACAGATAGATAGCGTACAACTCTGGACGAATCGTACCGCTTAGTAAAGTTTTTACAAATTCTTGTCT

TTCTGCTTTTTGATGAATCTCTTTAGTGAGCTCTTTGATGTCGTACATAATATAAAACCAGCGATGTAAAATAATTAATT

AATAATTACGCTGACATAGCAGCAATTCTTGCCGCTTCTCTATCTGCTTCTGCTGACTTTTCAGCAGCATTTGCGTCTAG

TTGTGCCTGTACTGCTTCTTCGTCTGTTTTATCTACACCAGCATAAACATCAACTGAAGAAGTTCCAAAATTATATTTCA

TTCTCCAATCAGCAATGTTATCAGGAGCGTCAGATACTTTTACGCAATGACCTTTTGCTACACCGTCATCACCAGTTACT

TCATTTCTTGTAAAAGGTTCGCCTGTTACTACTTTAAAATACATTGTTGCCATTATTATTCTCCGAAATTACCACCGTAG

TTACTATCAGCACCACCGTAATTACCCCACCAGTCAATACCTGCGATTACAGGATAACAAGTTGAGTAGTATCCTCCGTG

TAGTCCTGTTCTGGATTCTGATAATGAATAGTTACCAGTTTTGTTAGTTACGTCTGTAGCTCTAGCAGTGTTATTGTTTG

CTATTACATCATTGTTGTCCGTTGATCCGTAATACACTTTTGTGTCTACCGTATGATCTGAATCTGTAGGATCAAAAGCC

CAACTATAAGTTCTCCATTGTTCGGAGTCAGTATTGTCTGACCATCCACCGTGGAATCCTGTTCTGCCCCAAGGCACCCA

AGGATTTGATCTACTTGATTTAGTTTGGTTAACACTAATGAATTTTCTAGGATTTTCTAAACTCATACAGAAAGCATTTA

ATCCACAACCATAGTAGTAGTATGGAGAGAATATCATTCCCCAAGTACCATCCCAAGTTGTATGGAATTTAGTGTAGTAC

TGAGCACCTTGGTTTGCACCATAAGATGTAGTTGTTGATCCTGCAAAGTCTTGCCAACCAACATATTGTCTACCAGTATC

AGTTGTACCTAATGATTGTCCTAAATTACAATTGAATGCTGAGTATCTCATTGAGTTACCGTTTTTGTGTCCAAATCCAA

CCCAACCGTTATTACCAACAGCAACCGTCATATCTCTATTGTTATCTGTTGTCCAAGTACCTGTAAAGTATTCAGTAGCA

GTTAAGTTATCAAAAAATTCTTTAATTCTTGTTACTTTATTTAAATCTTTAGAAGATTGGAATACGTGAATTGTTTTTCC

TGTGTCTGAACCCTCATCAGCAGAGTGAACCATAACAAGCATTTTGTTTTTCTCGTTATAACCAGTTCCACAAGCGTATG

TGTGAGTGTTATCTAATTTGTTAGATGTATAATCGTAATGGTCAAAAGCAGCACAAGAGTTATTACCCATTGTCATTTCT

CTTAATGAGTTTCTTCTATTACAGAATAATCTTCTTGGTCTTATACCTTCTGGTAATACGTGGTTGATTTTTGTCCAACC

GTTATCGTATTCAAAAGTTGATGTATAGTGGTGAAAGTTTTGCCAAGAAATATATCCATCTCTACTTGATGTATAATATT

GTGCGTGTGGATATTGGTCAATCTGATAAGTAGATTTCCAATATTTTGAATAGTCTGAATAACTTTCAGACGTTAAATTG

TCGTGTCCAATATCAGTACCGAAGTCTGATTGGTTATAAGCGTGAGAAGCATCCCCTAGCATACCAAAACGGTAGTTAGT

TGTTGAGTTACATACAGCACCCCAAGGTGATCCGCACATTTTAAATCCTGAGTCGAAGACTCTGAACACCATATGAGTGT

TCATATCTGAAGTTGAACCAAAAAGTCCAAATAGAGGTAATCCCTCTTTTCTATGGTCTACGGTAGTACCGCCACCTAAT

AAATTTGATAAACTAGCCATTGATGTTTTCTCCTAAATTCTTAATACTATTTATATTATTAGTATTTCCCATTTTAATTT

TATGTTAATACCCAACCGATATAACTTGAAGTTACATCTGGCGTTGTTTTATAAGTTAA

>lcl|3300000199_____SI39nov09_10mDRAFT_c1000172|16420_ _

ACTACACACACCCAACAAGAGTTGGTGTCAACTGGAACAGATACGGTGGAATTAAAATGTACAAGACAACTCACTCAAAC

GGTGGACACTCTTGTACTCACATATTAGACGGCGAAGGCTATATGTGGTTTACAGGTTACACAACTTCAGGTGCTTGGCC

AATCGGCTCACCTGGTTACACAGATACGCACCATATTGGTTCGTTCAGAAGAGAAGGTCACTTCTTAAATGGAGACATTG

ACTTCTTCTGGTGTGGTGGAGATGAAAACAAATGGTTATATATCAGACAGAAAACTACTGGTATGCTATGGGTACACGAT

GGTAACTATGGTACATACGGTGGTCGTGGACAATCAGTTGAAAGTAATGGATACTGGTATGCTTCAGGTGGTCACCCAGG

AAGTTTCATACATATGAAAGGTCCTAAATGGGCAGTTAATGTATGTGATGTAGGTATGAGTAGAGCTGATGGTTCTTATA

TGTACTCTTTCCCAATGATACTTGATGACGAAGGATTGATTTGGGGTGGTGCTCCATATTCAAACAACGAGCAAGGTATG

GGTGGTGACACTAACTTTAATGACCAATTTACCAACGGTGGTAGAAACGATACCGGTCAAGGTATGGAAGACAATGAAAT

GTTTAGAACAAGAAAGAAAATCGTATTTCAACCAGGTGGTGGACATAGATGGACAGACTTATTCTATTCAGGAACTGGTT

CTTCAAATATACCAAGAGCTGTTAACCAAAGAGGTCAAGTATACTGGACTGGTTATGATGGTGGTGCTTCGGTAACTATG

AACTATGACTACTATTCAGAAGGTGCAAACTCAAACCAAGTTTCTTACTTCTTCCATTTGGGTCCTAGAGACTAATATAA

ATAATTATACATTAAGACTTCACGGTCTTTTACATTAAACAATTGAGGTGAATATGAAAGCAATAGAAGAATTTGTTGAA

CAGGCTCGTAAAAAGTTTGAATCACAACCGTTCATTAACGACTATCTAAACAAGAAACTTAAACACAAAGAAGCTGTTGG

TACTTACTTGTACAATCAATGGGTCTATGTATGTCAAATAGAAGGACATTGCAAAGACGCAGGTGTACTTGATGGTATTG

AAGAGATTTGTATTAAAGAAAATCTAAAAGAAGCGTGGAAAGCAGAATGGCCTTATGACGCTAATGATATAACAAAACCT

TGGGTAGAACCATCTGTAATGTATGCAACGCAAAGTTGGTGTACTTCTATTATAGATGTTAAAGAAGATAAAGATTTATT

ACTCGCACACCTATATGCCTCTCATAGTGAGATAATGACCAATCAAGGTACTTCAATTCTCAAAGATAGACTTACAGAAA

AATTTACAGAAGCATACAATCGTAGACCAGACGAAATGCTTAACATTATAAAACTAAATTGGGACTTTAAAATTGGTATG

TCTGGTGATTTAGAAGCACACAACGAACATTTAGAAGAAGTCTTACCTAGAATTTCTTTATTCAAAGTTGCTGCTAAAGA

AATTGNTGAAGACAAATCAGGTTTAAATGATATGTCTGGTGGTAGTAGAGACGAATCAGAAGACCAAAAAGTAAGAGCAG

AATTAATGGCAAATCAAATCTTTATAGGTGAAATGAATTTAGATGATGTGCCAGAAGATTATCAAAGTTTTGTAAAAGAA

GACTTACATCAACTTGAACAAAAGAAAAAAGAGACTGAAAAAACATTTGAAGAAGCCCCTAAAAGATGAAAACATTAAAA

GAGTTAACTTGGGAACATCATAAAGAAGCAGAAAGACAACACTTTGTAAAAGTATTAATGTCTGGAAAGATATTAGAAGA

AGTTTACGCTGTTTATCTTTACAATCAACATCAAGCATATAACATATTAGAAGCAGTCGCAATGGCAGACGGTTTCTTTG

ATGATATGCCACAATTAAGAAGAGCACCTGAAATCTTAAAAGATTTTAATGAACTATGGACTTGGAAACATAAACCTTGG

TTATGTGAAAGTACAAAAAAATATGTTGAATATGTAAATAAAAATTTAATGGATAATCCTGAAAAAATAGCTGCACATAT

CTATGTAAGACATATGGGTGATTTATCAGGTGGTCAAATGATTAAAAGAAAGACACCAAGTCGTAATTATTATTATGATT

TCAATTTTAAGAAAGTTGATGATGGTGTACAAAAATATAAAAGCGTACAAGAAATAAAAGACGCATTAAGATTAAAGATA

GATAGTTTTCAAAAGTATTCAGACGCAAGTACACTTACAGAAAATGTAAATAATGTTGTATATGAAGCAAGAGTTTGTTT

TAGTTTTGCAACGGAACTATTTAAAGAAATGATGACATTTATTAATAACAATGAAAAGAGGTTTGGTGATGGAACGAAGA

AGTAGAATATGGGAAATGCTTGAGCAACACACTCATAGTATTATTGCAAATTTTGAAAGAGAAGGTGAAGAGATATTTGA

ACCTGCAATGAAAAAGTTTAATAGACCTGAAGAAGGTTGGGTTAATAGAGTATGGAAAACACCTGAAGCAAGAAGATGTC

ATTTAGATGTAGTTGACGCTAGAGACGAAAAAGGTTTGTTTATGTTTCATTGTTGCGTGTTTCCTAATCTAACAAGTGAA

GCACCTATATTTGGATTAGATGTAATCGCAGGTGCAAAAAAGGTTACAGGTTTCTTCCACGACTTTTCTCCTCTTGCAAA

AAGAGACCATTCAATGGTAGATTGGTTTGTAAAAGAATCAAAGAATTATACACCATCAAAAGCAAGACCATTACCTGATT

GGGCAATGAAAATTTTTAGTCCAGGTATGATTGCGGCTGGTAATATTAATACTGAAAAAGAATTAACACAAGCGTTAAGT

ATGGCACAATCAAACTTACAAGTATATTTTACATTATTAAGAAGAAACAAAGAAGTAGGAGACTTACAGGAAATCAAAGA

CGCACAAAACAGATACGCAAAACATCAAAGAGAAAATCCACATACACCAAGAGTAATGTTAAGTTTAGGTTTACCTGAAG

ATGATGTAAAAGAATTTTGTACAGACGCTTTATTTCCTTATGTGGAATAATGGAACATTTAGACAAGTTTAAACAAGTAA

TAACAGAATTAAAAGATGACGGTAGATACCGTGTATTCAATGATATCCTACGAACCAGAGGTAGTTATCCTAACGCAATC

TGGTATTCAAAATACTCAATCAAAAAAATAGTTAACTGGTGTTCAAATGATTATCTAGGTATGGGACAGCATTCCTATGT

GTTAGATAGTATGAAAACAGCACTGGAGACGAGCGGAGCGGGCGCTGGAGGGACGAGAAACATCTCCGGCACAACTCACT

ATCACATTGCTTTAGAACACGAATTAACTCAATTACACGGCAAAGAAAGTGCGTTATTATTCACTTCAGCATACAATGCT

AATCAAACAACTTTAGAAACAATGGGCAAGATTATGCCTGACTTGTTGTTTATTTCTGACGCCGAAAATCACTCTTCTAT

CATACAAGGATTAAGACATAGTAAATGTAAAAAAGAAATATTTAAACATAATGATTTAGATGATTTAGAAAGTATCTTAA

TGTCTAACCCTGGTCCTAAATGTGTAGTATTTGAAAGTGTGTATAGTATGGACGGAGATATTGCACCTGTAAAAGAGATT

GCTGATTTATGTAAAAAGTATAAAGCTATATCTTATATTGATGAAGTACACGGAGTTGGTTTATACGGACCTAACGGTGC

TGGTATTTGTGAACGAGATAAAGTTGATGTTGATATTATCAATGGAACATTAGCTAAGGCCTACGGTGTACAGGGAGGTT

ACATTTGTGGAAAGAGAGAGTTTATAGACGCAATTAGAAGTATGGCAAGTGCGTTTATATTTACAACTAGTTTATCGCCT

GTACTATGTGCTGGTGCATTAACAAGTATTAAGTATGTTAAAGACCATCCTGAATTAAGAGAAAAACTACAAGAACGAGC

ACAAAAAACAAAAGAAGAATTAACAAGACAAGGTATAGAAGTATTACAAAACGATAGTCATATTGTACCAGTAATTATAG

GTGACGCTAAAAAATGTAAAGCAGTTTCAGATGAATTACTTTATAAAGATGGTATCTATGTACAACCTATTAATTATCCT

ACGGTTGCTGTTGGTACTGAAAGATTAAGATTTACACCTACACCATTTCATACAGATACAATGATATTTGATATGGTAGT

TAAAGTTAAATCTGCTATGCGAAGATGTGGTAGAACTAAATGAACATAAAAGAAGAACTTGATTGGTTAATAGTTGATGG

TGCAAATGGTTTAGAAATCTTATGGTTTATGTTAAGATACGACACAACTATACAAAGTTTGTTAGGTATAGGGGTATTAT

TGGCATTAGTATGTTGGTATTTTGATAAAAAAGACGATAAAGAGACGAATTGGGATAGCGACCCTCACGGAAAATTATAA

ATATTGCTAAAGATAAAGGAATAAAACTATGGCAATACCTAACACTAGACAGACACTTATCTCATATGCAAAGAGAGCAT

TGGGACATCCTGTTATTGAAATTAATGTTGATGACGACCAAGTAGATGATAGAGTTGATGAAGCAATACAATATTATCAA

CAATATCACTATGACGGTATCAAAAGAGTATATTTAAAATATCAATACACACAGGCAGATTACAATAGAATTAATACAGA

TACTTCTGAAGGTGCTACAAAAAATTCTGTAACCACAACTTGGAAAACAGGAAACGGTTATATCGTTGTTCCTGATTCAA

TTGTAGGAGTACAAAACATATTTCCTTTTTCAAACAAAGGTAGTATGAATCTATTTGATGTTAGGTATCAATTAAGATTA

AATGACCTTTACGATTTCTCATCTACAAGTGTTATCAACTATGATATTGTAATGAGACAATTAGATTTCCTAGACCATAT

ACTAGTTGGTGAAAAACCATTAAGGTTTAATCAACACGACAATAAATTACACATTGATATGGATTGGGGAAGCGACTTAG

CAGTTGGTGAGTATCTAGTTATTGACGCATACAGAAAACTAGACCCAGACACTTATACAGATGTATATGATGACATTTGG

TTAAAGAGATACACAACTGCATTAGTTAAAAAACAATGGGGTGCCAACCTCTCTAAATTTAATGGAGTAGCAATGATTGG

TGGGGTTACATTAAACGGACAACAAATCTATTCTGAAGCACTACAAGATGTAGAAAAATTAGAAACAGAAATTAGAAGCT

CGTTTGAATTAAACCCAGCAATGTTAATAGGATAAAAATAAAATGGCCGTTAATCATTACTTTCAAGGCGGCGATGGCAT

AGGTAGTCAAAATGAGAAAAGATTAATAGAAGATTTAATCGTTGAAAATTTAAAAATCTATGGTCACGCTGTTTACTATT

TACCAAGAACTCTTGTCAATAGAGATTTAATTCTAGGCGAAGACTCTGCGTCTAGGTTTGACGAGTCGTATCTAGTTGAA

ATGTACTTTGAAACGGTAGAAGGATTCCAAGGCGAACAAGAAATAATTAGTAAGTTTGGTTTAGAAGTAAGAGACGATAC

AACTTTTGTAATTGCAAAAAGAAGATTCCAAGAACAAGTTGATGATACTGCAAACTTGGTAGTAGACGGTAGACCTAACG

AAGGTGATGTAATTTACTATCCTTTAATGAACAAGTTTTTTGAAGTTTCATTTGTTGAAGACCAAGAGCCTTTCTTTCAA

TTAGGTTCTTTACCTGTTTACAAATTAAGATGTAAAACTTTTGAATATTCAAGTGAAGAGTTTAATACAGGAATTGGTGA

TATAGATTCAGCAGATGATAGACTATCATTAGATACAAGTTTACAATATCAATTTAGACTTGAAGATGGTACACTAAATC

AGTCTTCTTATTCTGGTTTCTTACAATTAGAACAAGGTGACGCAAATGGTAATCCTCAATATGTAATACAAGAAGAATTT

GATGATATTACTACAGACGGAGACGCCGCTACAAGTATACAAACAAAATCTGTTTACGCTGATAATTTAGATTTAGATAC

TGAAGCAGGTTTTGATACTGCAACGGTTTCAGATGACATATTAGACTTTACAGAAAGTAACCCATTTGGAGATGTTAAGT

AATGTTCGGAACACATTTTTATAACGAAGGATTAAGAAAATTGACTATTGCATTTGGTCAGATTTTTAATAAGATTGTTG

TACAAACAAAAGACGCAAATGGTTCAGTTGTAAAACGATTTACGGTGCCTCTTGCATATGCACCTAAAGAAAAGTTTATT

ACAAGATTAACACAACAACCTGACTTGCAAGATACACAATTTTCAACTATTCTACCTCGTATGGGTTTTGAAATATCTGG

TCTACAATATGACCCTAGTAGAAAATTAAATAAGTTGACAAAAACTAGAACACAAACAGATGATGGTTCTACTTCTTTTC

AACAAAACAATATGAAGTTTAATTATACACCTGTTCCGTATAACATAAATTATTCTTTGTTTATCTTTACTGCAACAGCA

GAAAACGGATTACAGATTATGGAACAGATAGTTCCGTATTTTCAACCTGATTATACGGTGACTATAAATATGATACCAGA

CTTAAATATTAAGCGTGATGTACCTATTGTTATAGGCAACATTGCATACGAAGATAATTATGACGGAGACTTTAATACAA

GAAGAGCAGTAATTTATACGATTAACTTTACTGCAAAAACTTATCTATATGGACCATCTACAAATCAAGGTGTTGTTAAA

AGAGTACAAACAGACCTTGGTACAGATACAATAGATAAAGCAAGAGAAGAAAGAATAGTGATAACACCAAATCCATCTTC

AGCAAAACCTGGTGATGATTTTGGATTTACAACAAACATATCATTCTTCAATGATGGAAAGAAATATGATCCAACAAGTG

GAAGTGATACATAATGAGAGGCAAAAATGAACAAAGAAGATATACTAGTATTAGACAATGTTTTACCAGAAGTTGTAAAT

AACAGCTTTCAACAAAACATTACAAGATTGGCATATATCCTATCTATGGATATTCTACCTAATCAAATGGACAATAAAGG

TATTATGAGTGATGAGAATACATTTACATCTACACAAATGGTACACAGAGTCTATTTACATAATCAACCTCAAAAAGGTC

CACAAAATCCTGCTATTGAACCGATAAAACATTCATTATCTGAAATGGTTGGTAAAGCAGGTCTGTTAACTAAAGACTTT

GATAAAGTAGAATTGTTAAGAGCAAAATTTAATTTAATGTTTCCACATCCTGATTTCAAAGATGGTCAGTACAATGTGGC

ACACATTGATGACGAACAAGAAGAACATCTTGTTTGCATTTATTATCCACAAGATACAGATGGTGATACCGTATTGTTCA

ATGAGTTTTTTAATAAAGATAAGAAACCAGAAAAATTAACTATTGCAAAGAGAGTTGAACCAAAAGCAAATCGTTGTGTA

ATTTTTAATGGTTGGAGATTTCACGCAAGTAGTAATCCAAGTGCATATAATAATAGAATAGTTTTAAATACAAACTTTAG

GATTGTAAATAATGGGTAAACTAGAAGACAAAGTAAATGATATTTTAGGTATCAAAGAAGAGAGTACTCCTGTAGCAGAA

TTAATGTTGCAAGAGAAGTCTGTACCTGTACCTAGAGTTGAAGACCCAAAGAAAGATGATATAGAAAACGATTACAAATA

TAGTAGAGAGAATTACTATAATTTAATTGAACGAGGACAAGACGCAATACAAGGCATTTTAGATGTTGCAAAAGAAGGGC

AACACCCGAGAGCATACGAAGTCGCAGGTGCATTAATTAAAAATGTAGCCGACACCGTTGATAAATTACAAGACTTACAA

AGTAAATTATCTAAACTAAAAGATGTACCTAATAAGACAACTAATAACATTAAGAATGCTTTGTTTGTAGGTTCAACTGC

TGAACTACAAAAACATTTAAAAGATAAAAAGTTTGATGAGAATAATAGAGACACAGCAAACGATCCTTTTAAAGATACAC

CAATTGAAGGAAAAGATTAAGTTATGTCTGACGCATATCTAGGTAACCCTAATCTAAAAAAAGTAAACACACCACAAGAG

TTTACTTCGGACGAGATTAAAGAGTTTAAGAAATGTGAAAACAATCCTATATACTTTATGAAGAAGTATGTACAAATTGT

TTCACTTGACGAAGGACTAGTGCCTTTTGATATGTATCCTTTCCAAGAAAAAATTGTAAATACAATACACGAAAATAGAT

TTACGATTTGTAAACTACCTAGACAATCAGGTAAGTCTACAACAACTATATCATATCTATTACACTATGCGTTGTTTAAT

CCTAATTGTAATATAGCAATTCTTGCTAACAAATCTTCTACTGCAAGAGATATATTAGGAAGACTACAACTTGCATATGA

GAATTTACCAAAATGGTTACAACAAGGTGTGTTAAACTGGAACAAAGGTAATATAGAATTAGAGAATGGAAGTAAAGTAG

TAGCGGCCGCAACATCTTCAAGTGCTGTCCGAGGAGGTTCATATAACATTATCTTCCTTGACGAGTTTGCTTTCGTACCA

ACAACTATTGCTGAACAATTTTTTAGTTCCGTTTATCCTACGATTACTTCTGGTAAGTCAACTAAAGTTATTATTGTTTC

AACTCCTCACGGAATGAATCAATTTTATAAACTATGGGTTGACGCTGAAGCAGGACAAAACGATTACATACCTATTGAAG

TATCTTGGAGAGAAGTACCAGGTAGAGACGAAAAGTGGAGAGAAGAAACAATAAGAAACACTAGTGAGTCTCAATTTGCT

AGTGAGTTTGAGTGTGAGTTTTTAGGAAGTATTGATACACTTATCAGTCCTGCTAAAATCAAAGCGACACCGTATAAGAC

ACCACTTAAAACAAATGGACGATTGAGTATCTTTGAAGAACCTATAAAAGGCCATACTTACTTATGTACGGTTGATGTTG

CCAGAGGTACACTAAAAGATTTCTCAGCGTTTATTATATTTGATGTAACCGATTTACCATATAAAGTTGTTGCAACATTT

AGAGACAATGAAATTAAACCTATATTGTTTCCTGAAATGATTGCGAAAGTTTGTACTCAATATAACAAAGCACATATACT

TGTAGAAGTCAATGATATTGGCGCTCAGATTTCAGATGGTTTACATTATGAATTAGAATATGATAATATACTAATGACTA

CACAAAAAGGTAGAGCAGGACAAATACTTGGTGCAATGTTCAGTCAAAGAGGTTCACAATTAGGTGTACGAATGACTAAA

CAGATTAAGAAAATGGGTACTGCTAACATCAAAGCGATTATAGAGTCTGATAAAATAATTATTAATGACTTTAATATTAT

TGGAGAAATGTCTACCTTTACAAGAAAAAATCAAAGTTGGCAAGCTGAAGAAGGCTGTAATGATGACTATATGACTTGTC

TAACTATATTAGGTTGGGTTGCAAATCAAAGGTATTTCAAAGAAATGACTGATAGAAATATCAGAGCAGAAATGTATAAA

GAACAAGAGAAGTTAATAGAACAAGATATGGCGCCGTTTGGGTTTGTTGATAATGGTGTTGATACACCAGAAGAACAACC

ATTTTCAGATGAATATGGCCAGGTATGGCATCCCGTGGTACGCAAAGGTAGTTAATGAAGATCCCCTATTTGATAAATAT

AAACGATTGAGAAATTTGAATATGGGCGTATGAATAATACGAATTTTGACAAAGGAAAACATTATGTATTTTTATAAAAA

TACAAACAATAAAATAGAGGAGAAAACCTAATGGCATTTCAAGTATCACCAGGTGTTCTCGTACAAGAAAAGGATTTAAC

TAACATAATTCCTGCTGTTTCTACTAGTATTGGAGCTTTTGCTTTCAATTCTACAAGAGGTCCAGTTGGAGAGATAACGC

TTATCTCTTCTGAACAAGAATTAGTTAGTATCTTTGGGAAACCTACTGCAAGCAACTTTGAAGAGTATTTTACTGCTTCA

TCTTTCCTTCAGTATTCCAATGCTCTGAAAGTTGTACGAACTGAAAACACTGGAATTTTAAACGCTGTAACCAATAGTGG

TTCAGCAGTACTGGTCAAAAATACTGACCATTACAACTCAACATACTTAGCAGATGGTGCTTACACAGGTATTTCTGGTA

GGGAGTTTGTTGCTAGAACAGCAGGCGCTTATGGAAATGGATTATCTGTTTCTGTATGTCCTTCTGCAACTGCATATGAG

CAAGAAGCGGTAACAACCGTTAACGATAGTGCTGTATCAGTTGGCGACACAACTATAACAATGACAAGTGGAACTAACAT

TAGTGTTGGTGACATACTTGCATTTTCAACAACAGCCGCTACTAACGATTATGATGACGGAATTGAATACGAGGTAACAG

CCGTAAATTCTAACGACATTACAATTAAGAAAAGAGTTGGTGCTGGTGGTCTAACAAGAGTTGTCATAGACGGCGCTAAT

GTTAGAAGAAGATGGTCACATTACGATTCAGTAAATGGTGCACCTGGAACATCTCCAGATGTATTAGCTGCTGGTGGTAG

TGATGATGAATTACACATTGTTGTAACCGACGCTGATGGTTCTATATCAGGAACTAAAGGCGAAGTACTAGAAGTATACG

AAAAAGTATCAAAAGCTAAAGACGCAAAAGATAGTGGTGGTTCTAATAATTTCTACCCAGAAGTTATTTACAAAAAATCA

TCTTTTATCTATTGGGGAGACCATAACGGAAACGGAACTAATTGGGGTTCAGTAAAGACAACTGCTTTCACTGCTGTTTC

AGGACCTATTGCATTAACATTCGGAAACGGTGTTGATGGAACGGTAACTGACGGTGCTAGAAAGTCTGCATTTGAATTAT

TCCAAG

>lcl|3300000260_____LP_A_09_P20_500DRAFT_1000343|289042

GTAGTAACCGCCGCAGATTCATAAGCAGAAGTTGAAGGACAAACAGATACTTTTAATCCATTTCCCCAAGCTCCTGCGTA

TCTAGCAGCAAACTCAATGCTGGAGATTCCTGGATATAAGCCTTCGTTTAAGTATGTAGAATTGTATTCATTAGTATGTC

TAATCAATACTGCTGTTCCCGAGTTGGTTACAGCGTTTACCATGTTTAGGTTCTCAGTTCGTACAACTTTCAAAGAATTG

GAATACTGAAGGAAAGATGAAGCAGTAAAATACTCTTCAAAATTCGTTTGAGTCGGTTTTCCAAAAACACTAACAAATTC

TTGCTCAGAAGATATTAAAGTAACTTCTGAAACTGGACCTCTAGGTGCATTAAAAGCATATGCTCCAATACTTGTAGAGA

CAGCTGGGATTATGTTAGTTAAATCCTTTTCCTGTACGAGAACTCCTGGTGATACTTGAAATGCCATTAGGTTTTCTCCT

CTTTATTATATTTTTTATTGTATTTAATTAAATACATTATATTACCTTTGTGTCAAAATTCGTATTATTCATACGCCCAT

ATTCAAATTTCTCAATTACTTATATTTATCAATACCTAATCCTAGACTATTGACCTTTACGAACAACAGGATGCCATACG

GTTCCATATTCATCAACGGTTGGCTCATCTTCTTCCTTTGTTACACCATCATCTACAAAACCAAATGGTGCCATATCTTG

TTCTATTAGCTTTTCTTGCTCTTTATACATTTCTGCTCGTATGTTTCTATCTGTTAATTCTTTAAAATACTTCTGATTAG

CAACCCAACCAAGTATAAGCAAACAAGTCATATAATCGTCATTACAACCTTCTTCTGCCTGCCAAGATTGATTTTTTTGT

ATATAAGTAGACATTTCTTCAATAATATTAAAGTCATTTATAACTAACTTGTCTCCTTCAATGATTGACTTAATATTAGA

AGTACCTATTTTCTTAACCTGTTTAGTTGTACGAATACCTAATTGTGAACCTCTAGCACTAAACATAGCACCAAGTATTT

GTCCTGCTCTTCCTTTTTGTGTAGTCATTAATATATTTGGATATTCAATCTCAAAATGTAAACCATCTGAAATTTGAGCA

CCTATATCATTTACTTCAACAAGTATATGTGCCTCATTATATTGTTTACATACTTTAGCAATTGTTTCTGGAAATAATAT

TGGTTTAATTTCATTATCTCTAAATGTAGCAACTACTCTATAAGGTAAATTGGTTACATCAAAAATAATAAATGCTGAAT

AATCTTTTAAAGTACCTCTAGCAACATCAACCGTAGACAAGTATGTGTTTCCCTTAACTGGTTTCTCAAAAATACTTAAC

CTGCCGTTTGTATGTATAGGTGTAATATAAGGTGTCGCTTTTATTTTAGCAGGTGAAATTAATGTATTAACAGAACCTAA

AAATTCACACTCAAATTCTGAACTGAATTGAGCTTCCGAGGTATTTCTAATCGTCTCCTCTTTCCATTTGGCGTCTCGGC

CTGGTACTTCTGACCAATGTACTTCAACAGGTACATAATCATTTTGTCCATTTTCAGCGTCTATCCATAATTTATAAAAT

TGATTCATACCGTGAGGTGTTGAAACTATAATTACTTTAGTTGTCTTACCAGAAGTAATAGTAGGATAAACAGAACTAAA

AAATTGTTCGGCAATAGTAGTAGGTACGAAAGCAAACTCATCAAGGAAGATTATATTATAAGAACCTCCCCTTACTGCTG

ATGATGAAGTAGCAGCCGCTACTATTTTACTTCCATTTTCTAATTCAATATTACCTTTATTCCAATTCAATACACCTTGT

TGTAACCAACTAGGTAAGTTCTCATAAGCAAGTTGTAGTCTTCCTAATATATCTCTAGCAGTTGAAGATTTATTAGCAAG

AATTGCAATATTAGAATTTGGATTAAACATTGCATAATGTAAAAGATAAGAAATAATAGTTGTAGATTTTCCACTTTGTC

TAGGCAACTTATAAATTGAAAACCTATTATTGTGCATAGTATTAAGCATTTTATCTTGGAACTTATAAGTGTTAAAAGGT

ATTAAACCTTCATCAAGGGATACAATCTTTACATAATTTTTAATAAAATATGTTGGACTTGCAGAACACTTTTTAAATTC

TAAAACCTGTTCTTCTGTAAACTCAACAGCTGTACCTACTTTTTTTAAATTCGGGTTTCCAAGATAAGTATTATCCATCT

TTTTTCTCTTTGTCGTCTTCTACTATCGTAGCGTTTTTAATTTTATCTTTATTTAACATCTTTTGCAACTCGGTAGTACT

CCCAACAAATAAAGCATTTTTAATATTAGCAGTAGTCTTATTAGGCAACTCTTTTAGTCTTGCTAATTTTGCTTGTAAGT

CGTGTAATTTATCAACGGTATCTGCTACATTTTTAATTAATGCACCTGCAACTTCATATGCTCTAGGATGTTGTCCTTCT

TTTGCAACATCTAATATACCTTGAATAGCGTCTTGTCCTCTTTCAATTAAACTATAATAGTTCTCTCTACTATATTTGTA

ATCATTTTCTACATCATCTTTCTTTGGATCCTCTACTCTAGGTACAGGAACCTTTTTCTCTTGTACCATTAATTCAGCAA

CAGGAGTACTTTCTTCTTTGATACCTAAAATATCATTTACTTTATCTTCTAATCTTCCCATTAGTCTTTACCTTCTATAC

TAGTTCCTTTAAAAGGATCCTTTCTTGTATCTCTATTATCTTCACTATAATTTTCCAATAAAGAAAAATTACAATTTAAA

ACAACTCTCATATCATTTTTTATTGGATTACTACTTGCGTGAAATCTATTTCCTTTAAACATAACGCACCTATTTTGTTT

TGGTTCTATTCGTTTATGTATAGTTAGTTTTTCTGGTTTCTCTAGTTTATCAGGATCAAAAGATTCATTAAACAATACGG

TGTCGCCATCACTATTCATTGGATAATAAAGTAAAACAAAATGTCGTGCCATTTCTTCATCATCAATGTGTGGTGTATTA

TAGTTACCTTCTTTAAATCTTGGATGTGGTTGTATCATATTAAATTTTAATCTTAATATCTTATCTACCTTATAACCAAA

ACCTTCAACCATCATATTCAAAGCATATTTAATTGGTTCGTATCCTGGATTAACAACTGCTCTTTGGTCGGCGTGATTAA

AGACTCGGTGCACCATTTGAATAGTATTAAATGTATTATCATCTTTGACAATACTTGGTTTATCCATTTGTGTTGGTAAT

ATATCTTGTGCTATTATATATCCAAGTCTGTGTATATTTTGATAAAAACTATTGGCTAGTTGTTCTGGTAAAGCATTGTC

TTTGACTAATACATTGTTCATTATGTCCTCTCATATTATGAATCACTTCCTGTCGTAGGATTAAATTTCTTACCATCTTC

AAAAAATGATATAGTTGTTGTAAACCCAAAGTCGTCTCCTGAATTTGCTGTAGATGGATTTGGTGTAATTACTATTCTTT

CTTCTCTTGCTTTAGTGTCAAAATTTGTTCCTAAATCTGATTGTACTTTTCTAACAATACCAGCAGTTGTTGCAGGTCCG

TATAGATAAGTTTTAGCAGTAAAACTAATTGTATAAATTACTGCTCTTCTAGTTTCAAAGTTTCCATCATAACTATCTTC

ATAATCTACATTTCCAATTATGATTGGAACATCACGCTTAATACCCATACTAGGTACCATATTAATTGTAACCGTATAAT

CTGGTTGAAAGTATGGTACTATTTGTTCAACTATCTGTAATCCATTTTCAGCAGTTGCTGTAAATATAAACAATTTATAA

TCTATATCATATGGAACTGGAGTATAATTAAAAACCATTTTGTTTTTTTGGTCATCAGCAGTACCATCTGTTTTTGGCAT

CCTAGTTTTTTGTAATTTATTTAATTTTCTACTAGGGTCATATTTTATACCAGATATTTCAAACCCCATACGAGGTAGTA

CCGTTGCGAATTGTTTATCAGTTAAATCACCTTGTTGAGTTAATCTAACTAAAAACTTTTCTTTTGGTGCATATGCTAAT

GGCACCGTAAATCTTTTAACAACTGAATTATTTGCGTCTTTAGTTTGTACTACAATATTATTAAAAATTTGACCAAACGC

AATAGTCAATCTTCTCATACCTTCGTTATAAAAATGTGTACCGAACATTATCTAACCTCTCCAAATGGATTTGTTTCTGT

AAAGTCTAATATATCATCAGCAACCGTAGCAGTATCAAACCCAGCAGCTGTATCTAAATCTAAATTATCAGCGTAAGCAG

ATTTTGTTTGTATATCTGTAGCGGCGTCTCCATCAGTTGTAGTATCATCATATTCTTCTTGTATTATATATTGTGGATTA

CCATATGCGTCTCCTGTTTCTAATTGTAACATACCAGTATAAGAAGTTTCGTTTAGTGTACCATCTTCAAGTCTAAACTG

ATATTGTAAACTTGTATCAAGCGATTTTCTATCATCTGCTTGGTCAATATCAGGATGTCCAGTATTAAATTCTTCTGAGC

TGTATTCAAAAGTTTTACATCTTAATTTGTATACAGGTAAACTTCCTAATTGAAAGAAAGGTTCCTGGTCTTCAACGAAA

GCAATTTCAAAAAACTTGTTCATTAAAGGATAGTAAATTATATCACCTTCATTAGGTCTACCATCTACTACTAAATTTGC

TGAGTCGTCAACTTGTTCTTGGTATCTTCTTTTTGCAACAACAAAAGTTGTATCGTCTCTAATTTCCATTCCAAATTTAG

AAATAATCTCTTGTTCTCCAGCAAAACCTTCAACCGTTTCAAAATACATTTCAACCAAATAACTTTCATCAAACCTAGAC

GCAGAATCTTCACCAAGAATTAAATCTCTATTAACTAGAGTTCTCGGTAAATAATAAACAGCGTGTCCATAGATTTTTAA

ATTTTCTACGATTAAATCTTCTATTAATCTTTTCTCACTTTGACTACCTATGCCATCGCCGCCTTGAAAATAATGATTAA

CGGCCATTTGATTTTTATCCTATCATTAATGCTGGGTGTTCTTGTAAACTCTTCATTTCTTCCTCTAACTTTTCTATATC

GGATAATGCTTGAGTGTAAATTTCTTCACCCTTTAAAGTTACTCCACCTATCATTGCTACCCCAGCGAATTTAGATAAGT

TAGAACCCCATTGTTTTTTAAATAAAGCAGTTACATATCGTTTTAACCAAATATCATTATAGACATCTGTATATGTGTCT

GGATCTAATTTACGCCAACAATCTATAACTAGATATTCACCAACTTGTAAATCGTTTTTCCAATCCATATCAATGTATAA

TCTATTGTCGTGTTGATTAAATCTTAATGGTTTTTCACCAACCAATATATGGTCTAGGAAATCTAGTTGTCTCATTACTA

TGTCATAGTTAACAACAGAAGTTGATGAGAAATCATATAGGTCATTTAATCTTAATTGGTATCTAACATCAAATAGATTT

AAATTACCTTTGTTTGAGAAAGGAAATATATTTGTAACCGCAAATACGGTTTCAGGTACTATAATATAATTATTACCTTC

TTTCCAGGTACTAGTTACAGAATTTTTTGTTGCGCCTTCGGTACCATCTGCTAAAATTCTATTTTGGTCTGCTTGAGTAT

ACTGATATTTTAAATATGTTCTTCTGATTCCATCATAGTGATATTGTGAATAGTATTGTACTGCTTCATCTACTCTATCA

TCTAGTTGGTCATCATCTACATTTATCTCTATAACAGGATGTCCTAATGCTCTTTTAGCATAAGATATTAATGTTTGTCT

CGTATTTGGAGTTGCCATAGTTATTTCCTTTTATCTTCTGCTATATTTATAATTTATTGGTAGTCATTTGTTATCCATTT

GACATTATCTTCAATCGTTGCTTTATATCTATTTTGAGTGTCTTCTATTTCCTGTGTATTTTGTACTTCTTTATCATATC

TTCTTAATAGTGTAAAGTAAACACCTAGATTGCATTCTACTAAACTCAAAGCATTCATAAGTTCGCTTTCTTTTGTTATA

TTAGTAGCAGCTATTACATTAGGACTATAAATTTTAGTTGCCCAATCAGGTAACTCTCTTACTTTAGATGGTGTATATCC

CTTTACTTCTTTAGCAAACCAAGGAACCATAGAGTGGTCTTTCTGTACTGGAGTAAAATCGTGGAAGACATCAACTCTTT

TTGTAGTCGCACATATACTTAATCCGTAAATAGGACTATTATATTTTGGAAATACACAACAATGAAGCATATAGAATCCT

TTTGTTTCTTTTTCGTCAACAACCTTTAATTGACATCTTCTTGCTTCTGGTGTTTTCCATACTCCTTTACCAATCCATTT

ACCTTCTCTTTTAAAAATTGCTTGAATATGTGTAGTTACACCTTCTACCATTTCCCATATTCTATTTCGCTGATAGTAAG

CCATCGCCAAACCTCTTTTCATTATCTTTTATAAATTTTTGCATATCCTTAAATAGTTCTGTTGCAAATCCAAAACACAT

TCTTGCTTCATAAACAACACTATTAATATTTTCAGTTAAAGTACTTTGGTCTGAATATTTTTGATAACTATTTACTTTTA

CTTTTAATGCTTCTTCTAATTCTTTAATATTTTTATACAATTGTCTTCTTCCTTCAACATATCTAATATTAAAATTATAA

TATGTTCCTTGACCTGGTACTTTTGATTTTATCATTTGACCACCAGATAAATCTCCCATATGTCTTACATAAATGTGAGC

AGCTATCTTATCAGGATTATCCATTAAATTTTCATTACAATGGTCCAAATATTTCTTTGTAGAAGTCATCATTGCAGGTT

GCCATTTATATGTCCACAATTCATCAAAGTCTGCTTTAATAAATTTTGCTCTTCGCAATTCAGGCATATCATCAAGGATA

CCTTCTGACATTGCTAATGATTCTACTATATTATAACATTGATGTTGATTGAAAAGATAGACAGCATAAATCTCTGGATT

TATTTTACCTGACATAAGAGTTTTTACAAAATGTTGTCTTTCAGCTTCTTTATGATGTTCCCAAGTAAGTTCCTTTAATG

TTTTCATCTTTTAGGAGCGTCTTCAAATTTCTTATCTGTTTCTTTTTTCTTTAGTTCTTCTTGTTTTAAATCTTCTTCAA

CATACTCTTTATATTCTTCTGGTAAATCATCAATATTCATTTCTTTCATAGTAACCGCATTTTTCATTAATTCTGCTCTT

ACTTTCATATCTTCTGTTTCGTCTCTATTACCAGCAGACATATCATTTTTACCAGACTTATCTTCCATTATTTCTTTTGC

ACCAACTTTAAATAGATTAATTCTAGGTAACACTTCATCCATATATTCTTTATGTGCTTCTAAATCCCATACTAAACTTT

GTTTAAAATCCCAAGATGTTTTAATCTCAGCTAACATTTCATCTTTATGGTCTTTGTAATATTCGTTAAATTTTGTTTCT

AACTTTTCATATAATACAGAAGATTTTTGATGTTTATGTATCTCGCTATGAGTAGCGTATAGGTGAGAAATTATTTTGTG

TGGTATGTCTTTAATAGAATTTATATGTTCAACCCAATTTTGAGTTGCATACATCATTGATGGTTGTACCCAAGGCTTAC

TAACATCATCAGCCTCATAAGGCCATTCTTGTTTCCAAGCTTCGTTTAATTTGTTTTGTATTAGTACTTCGGGAGAGTCT

TTAGTATATTCACTACAAAGATTTTCTATTGATGATATAATCAACGATTGATTATAGAGAAAAGTTGCCTTTACCTCTCT

AATTAATTTGTTGTTTATATATTCTTCTATGAATGGTAAGGCGTCATATACTTTACGAGCCTTGTCTATAAAGGTTTCTA

GGTCTTTCATTTTTCACTCCAAGTTTTTTATATAGTGACCTGTTAACAGGTCTATAATATAGTTATTTATATTGTTGTTT

ATTAGTCTCTAGGACCCAAGTGGAAGAAGTATTGTGTTTGGTTACTAGCAGCACCTTCACTATAATAATCATAGTGTTGA

GTTACTGAAGTACTTCCATCATAACCAGTCCAGTATACTTGACCTCTTTGGTTAATAGCTCTTGGTATATTTGAAGAACT

AGTTCCTGAATAGAATAAATCTGTCCATCTATGACCACCTGAAGGTTGGAATACAATTCTTTTTCTTGTTCTAAACATTT

CATTGTCTTCCATAGCACCTTGCGTGTCATTACGACCACCATTATTCCAATGTTCTTCATTTGAAGACGGACCACCAAGT

CCTTGTTCTTCATTACTATATGGAGAACCACCCCATATTAATCCTTCGTCATCAAGTATCATTGGGAAAGAGTAAGTATA

AGTTCCATCACCTCTATTTTGTCCAACATCACATACATTGACAGCCCATTTAGGACCTTTTTGATGTATAAAGCTTCCTG

GATGTCCACCAGAAAAATAAGTGTATGCGTTGTTTTCTACTGATTGTCCACGACCACCATAAGTTCCATAGTTTCCATCG

TGTACCCATAACATACCTGTTGATTTTTGTCTTATATACAACCATTTTTCTTCATCGCCACCGCACCAGAAGAAATCTAT

ATCACCGTTTATAAAGTGACCTTCTCTTCTAAATGAACCAATGTGATGAGTTGTTGTTAATCCTGGAGAACCAATTGGCC

AGTTACCAGAAGTCGTATAACCTGTGAACCACATATATCCTTCACCATCTAAAATGTGTGTAGAGTGTTGGTCACCGTTA

GAGTGAGTAGTTCTATATAATTTAATTCCACCGTATCTATTCCAGTTAATTCCAACTCTATAAGGTACCGTATAGTAATG

AGATCCAACTCTATTACCTAATCCTAATTCACCGTAAATATTGTGACCCCAAGCCCATAATTGTCCTGTGTCATCTAAAG

CGTGCCAATAAGCATTCTGTGAACCAGAAGCCCACATATCTACAATTCTTCGTCCATCAAAATATGATTGTGGTAATCTT

ACTGGTCTTGGTATGTTTACAGAATAGAAAGCAGTTGAAGTTGGTCCGCCAACAGCGTCAGCAAGAGCATTAATTCCTGG

ATTACCTAGTCCAAGTTGACCTTCATTATTGTATCCCCATACCCATACTGAACCATCGGAACCGAGTGCGTATTGTGAAC

AAGTTGTTCCTTGGTCTTTTGCTTCGTCTGAAAATCCAACTTTAACCATTCTAGTTTGATTGAAAGTTTTTCCAATTGTA

TTTCCTTGCCAGTCTTGTGTATCTGTTGCAGTACATCTTACAGCATAAGGTCTATCTGTAGTTTCAGAATTTCCTAAATT

GTAATTTCCATTATTACCAGCAGCATATACTTCACCATTATTCATTAACCACATTGTTCTTTGATAATTATGTGCTATTT

GTATTACTCTTGGTGCTTTACCGTCAGGAGTTGTCATCCTTCCAGTCGCATTAATATTCCAATTTTGATTATCTGTAGAA

GCCATCCAATCTGTAAATGTGAAACCTGTTGACATATGTTTTGCAGGTTCGTCATTTCCAGCAGTACCTTCTCCTAATCC

AGAACATTGGGATGAGTCAGAAGAACCAACCCACATATCAGAACCATCTGAGCATATTGTAGCAGTTCTATATGATTGGT

CAGCACAACCAGTGTGTGAAACTCCCATATTGTATCTCCAACCTAACGGAGCACGGTTGTTAAATGATACAACTTCGTTT

CTAGTACACCACTTGTTTTCATTAACATATATTAATGTCCAATATTTTGATGGTCTTCCATCGTGTTCTTCTACCCAAGT

ATTACAATATCTTGTTCTTTGTAAACATAGATAAATTTTGTCAGCAACTTTAACCATTTCGCCTCTTTCATATTGTCTCC

AATATTGCCAAGTTTCAATGTTATCGTTACCTGCTAATAGTAATGTCCAATATCTTCTATTATCTGGTCTGTAACTTCTT

TTGACTAGCGTTGGTTGATAATTACCATACGAATTAGTATTTTGTGGAGCTTCAGAAGAAATTGTATAGTGAGTAGGTGT

ATCTTCTATACATCTATAACTTTTACCTCTCCAATAAACCACATCATTTTTATCGTAAGATTCTTTATCTT

>lcl|3300003620_____JGI26273J51734_10000260|87946

CAACAAGGTACTGGTTATGATGGTGCTCCTGGTTCTGCAATGTCACCTGACGGATTAGAAGATGTACACTTTATCAAAAT

CGCAATGAAATTAATGAACGAAGATTCAACACATACTCCTTGCGGATTAACAGATGATGGAGATGTATACACTTGGGGTT

ACAATGGTTACGGTGAAGTCGGAGACGGTAGAACAGATAACGCATACGGTCCTAAGAGAATACCAAGAGAATTCTTTAAT

GACGAAAAAATTATTGATATTTTAGCAACTGGTGGAGATAGTACTTCATTCTATGCTAGAACATCACAAGACAACATTTA

CGGTTGGGGAAGAAACAACATCGGCCAATTAGGAGATACAACAACGACTGACAAATACAGACCAGTATTAATGACTGGAT

TTAATGCTGCTGACAATGGTGGTATCGCTGTATGGCAAGGTGACGCTCACTCATCTAATTCTGCTTTCTACATATTAGAC

GGAAACGGATTTATATGGGCAACAGGTTACAATGGTTATGGTAACTTTGTTGATAACTCAACATCTAATAGAACACAATT

AACTCAATCAACTGCTTCTCCTAATGGAGATATAGCAGACTTCTGGACAATGTACTGGAATGGATATCATACAACATTTA

TGAGATTGAAAAATGGTGAAACTTGGACTGCTGGACATAGTGGTGGATACTACAACTCTGGTGATGGTGGAACTGGAACA

AACCAGGCACCTGTACAAGTAGATAAGATAACTAACCTAAAAGAAGTTTGTATATGTAATACATATTCAGACCAAGGTAG

AAGTTATTGGTTAACAGATAATGGTGAATTCTTTAGTCAAGGCCGTGATGTATACGGTTCTATGCCAAATAGTGTTGCTG

GAGATAACTGGAACGGTGAAGATGGAACATACAAACCTTTCCACGCTTTTGTACCTGCAGGAACTAGAATTAGAACTATG

TGTATTCAAGGTATTGACCAATCAACTAACTATTACGGATTACAACCAATGGTTGGAACAGAAGATGGACAAGTCTTGCT

TTGGGGTTACTCAAACAACAATAACCTAGGACACCACGCTAGTGCAACTTACTCATCAACGGGTCGTTCACAAATGTGGA

ATGCTGGTATTGGTAGATAATATAAATAGAAGTATAACAAATAAAGAAAACGGAGAAAAAACAAAATGGCAAAAGTAATT

TATTCAATGACTGCTGGAATCGGTCAAGGTGACGACTATACTGCTCCTACAGGAGATACGCCAATCAGTTTAGGAGAATT

AAGTGGAAAAACTTATTTCTCAATTGATGATGGTAATACTACTATCACAACTGATGGTGCAAATGATTCTGTATACGGTG

TAGCTGTTGTAACCGACGCTGATGAAAAAGCAAGTTTACAAGCTGCTTCAAACTATGTTGCACAAGGCTTGGGAAATTTA

GATAACGAATTTATGAATGGTAAATCAATGATTGACTTATTAGCTGATGTGGCTGATGATACTGCAGGAACTAAAACTGC

AATCGCAACTCACAAAGCTGCTAAAGCTTCATTCCTATCAAACTTAGGATTCTAAAGACAAATTAAATAAAAAGAAGGTA

ATAGCAAATGGCACTAGATATACAAAACTTTAAAGTATCCTGGAAAGGAAACTGGAAAGATAAAGAGAAGTATTATAAGA

ATGATATAGTTTACTGGAGAGGTAAGTCATACAGATGTACAGAAGAGACACCTGATAACTTTACTATTTCAAGTGAATCT

ATGGTCAATACGAACTCTTATGGCCAATATCAGCCTACGGTTGTTAGACGGTCATATAGACCCGACAACAATAGATACTG

GACGCTATTGCTTGCAGGTAACGACAACATTGAAACTTGGCAGTATTGGAGACAATATGAAAGAGGTGAAATGTGTAAAG

TTGCCGATAAGATTTATCTTTGTTTGAAAAAAACAAGATATTGTAATACTTGGGTAGAAGAACACGATGGTAGACCATCA

AAATATTGGGCACTTGTTTACATTAACGAAAACAAGTGGAGTACAAGAAACGAAGTTATTTCATTTAACAACCGAGCTCC

GTTAGGTTGGAAATATAATATGGGTGTTGACACACAAGATAGTGCTGACCAAACATATAGAACTTGTACACTATGCTCAG

ATGGTTCTGATATGTGGGTAGGTTCTTCTGAAGGAACTGGTTCATCTGGATTAGGTGACGGTGTTGCTGGAAATGACGAA

CCTGGAAAACACTTTTCTACAGGATTTACATTTACTGATTGGATGGCTTCTACAGACAACCAATCTTGGAATATTAATGC

TACAGGTAGAATGACTACACCTGATGGAAAAGCACCAAGAGTAATACAAGTTAGAAAAAATCAAAATAGAACTTTCTGGT

TAATGAACAATGGTGAAGTTTACGCCGCTGGTGAAAATGGAAACTACGGTTTAGGAAATTCAGAAACTTCAGATAGAAAT

TATAGTGTAAGGGTTACTGCTAATGATACACAAGATTGGCAAGGAAACACTATAGGCAAAACATTCAATCAAACTAAAAT

GGTTAAAGTCGGTATGTCCGACGCCGGACACGACGCTGGAACATCATCTTGTTTCGCATTAGGTGATGACGGTTCAGTAT

GGGTTTGGGGTTACAATAACAACGGTCAATTAGGACTAGGTAATCCATCAATAAACAATTCAACAGACACNTCTGGTGGN

CCAACAGGTACTGCTTTCTATAGTGCCAATGTNACCAGACCAGTTAGATTACCTCAATCATACTTTGATGGAAGACAAAT

CATTGATATGTGGACTTCAGGTTCAGAAGAGGCGTGGTTCCACGCATTAGACGAACACGGTCAACTATGGGCTTGGGGAC

ATAACCAACACGGTGAATTAGGAGTAGGAAACAGAAATGGCACTTACTACTACACACACCCAACAAGAGTTGGTGTCAAC

TGGAACAGATACGGTGGAATTAAAATGTACAAGACAACTCACTCAAACGGTGGACACTCTTGTACTCACATATTAGACGG

CGAAGGCTATATGTGGTTTACAGGTTACACAACTTCAGGTGCTTGGCCAATCGGCTCACCTGGTTACACAGATACGCACC

ATATTGGTTCGTTCAGAAGAGAAGGTCACTTCTTAAATGGAGACATTGACTTCTTCTGGTGTGGTGGAGATGAAAACAAA

TGGTTATATATCAGACAGAAAACTACTGGTATGCTATGGGTACACGATGGTAACTATGGTACATACGGTGGTCGTGGACA

ATCAGTTGAAAGTAATGGATACTGGTATGCTTCAGGTGGTCACCCAGGAAGTTTCATACATATGAAAGGTCCTAAATGGG

CAGTTAATGTATGTGATGTAGGTATGAGTAGAGCTGATGGTTCTTATATGTACTCTTTCCCAATGATACTTGATGACGAA

GGATTGATTTGGGGTGGTGCTCCATATTCAAACAACGAGCAAGGTATGGGTGGTGACACTAACTTTAATGACCAATTTAC

CAACGGTGGTAGAAACGATACCGGTCAAGGTATGGAAGACAATGAAATGTTTAGAACAAGAAAGAAAATCGTATTTCAAC

CAGGTGGTGGACATAGATGGACAGACTTATTCTATTCAGGAACTGGTTCTTCAAATATACCAAGAGCTGTTAACCAAAGA

GGTCAAGTATACTGGACTGGTTATGATGGTGGTGCTTCGGTAACTATGAACTATGACTACTATTCAGAAGGTGCAAACTC

AAACCAAGTTTCTTACTTCTTCCATTTGGGTCCTAGAGACTAATATAAATAATTATACATTAAGACTTCACGGTCTTTTA

CATTAAACAATTGAGGTGAATATGAAAGCAATAGAAGAATTTGTTGAACAGGCTCGTAAAAAGTTTGAATCACAACCGTT

CATTAACGACTATCTAAACAAGAAACTTAAACACAAAGAAGCTGTTGGTACTTACTTGTACAATCAATGGGTCTATGTAT

GTCAAATAGAAGGACATTGCAAAGACGCAGGTGTACTTGATGGTATTGAAGAGATTTGTATTAAAGAAAATCTAAAAGAA

GCGTGGAAAGCAGAATGGCCTTATGACGCTAATGATATAACAAAACCTTGGGTAGAACCATCTGTAATGTATGCAACGCA

AAGTTGGTGTACTTCTATTATAGATGTTAAAGAAGATAAAGATTTATTACTCGCACACCTATATGCCTCTCATAGTGAGA

TAATGACCAATCAAGGTACTTCAATTCTCAAAGATAGACTTACAGAAAAATTTACAGAAGCATACAATCGTAGACCAGAC

GAAATGCTTAACATTATAAAACTAAATTGGGACTTTAAAATTGGTATGTCTGGTGATTTAGAAGCACACAACGAACATTT

AGAAGAAGTCTTACCTAGAATTTCTTTATTCAAAGTTGCTGCTAAAGAAATTGNTGAAGACAAATCAGGTTTAAATGATA

TGTCTGGTGGTAGTAGAGACGAATCAGAAGACCAAAAAGTAAGAGCAGAATTAATGGCAAATCAAATCTTTATAGGTGAA

ATGAATTTAGATGATGTGCCAGAAGATTATCAAAGTTTTGTAAAAGAAGACTTACATCAACTTGAACAAAAGAAAAAAGA

GACTGAAAAAACATTTGAAGAAGCCCCTAAAAGATGAAAACATTAAAAGAGTTAACTTGGGAACATCATAAAGAAGCAGA

AAGACAACACTTTGTAAAAGTATTAATGTCTGGAAAGATATTAGAAGAAGTTTACGCTGTTTATCTTTACAATCAACATC

AAGCATATAACATATTAGAAGCAGTCGCAATGGCAGACGGTTTCTTTGATGATATGCCACAATTAAGAAGAGCACCTGAA

ATCTTAAAAGATTTTAATGAACTATGGACTTGGAAACATAAACCTTGGTTATGTGAAAGTACAAAAAAATATGTTGAATA

TGTAAATAAAAATTTAATGGATAATCCTGAAAAAATAGCTGCACATATCTATGTAAGACATATGGGTGATTTATCAGGTG

GTCAAATGATTAAAAGAAAGACACCAAGTCGTAATTATTATTATGATTTCAATTTTAAGAAAGTTGATGATGGTGTACAA

AAATATAAAAGCGTACAAGAAATAAAAGACGCATTAAGATTAAAGATAGATAGTTTTCAAAAGTATTCAGACGCAAGTAC

ACTTACAGAAAATGTAAATAATGTTGTATATGAAGCAAGAGTTTGTTTTAGTTTTGCAACGGAACTATTTAAAGAAATGA

TGACATTTATTAATAACAATGAAAAGAGGTTTGGTGATGGAACGAAGAAGTAGAATATGGGAAATGCTTGAGCAACACAC

TCATAGTATTATTGCAAATTTTGAAAGAGAAGGTGAAGAGATATTTGAACCTGCAATGAAAAAGTTTAATAGACCTGAAG

AAGGTTGGGTTAATAGAGTATGGAAAACACCTGAAGCAAGAAGATGTCATTTAGATGTAGTTGACGCTAGAGACGAAAAA

GGTTTGTTTATGTTTCATTGTTGCGTGTTTCCTAATCTAACAAGTGAAGCACCTATATTTGGATTAGATGTAATCGCAGG

TGCAAAAAAGGTTACAGGTTTCTTCCACGACTTTTCTCCTCTTGCAAAAAGAGACCATTCAATGGTAGATTGGTTTGTAA

AAGAATCAAAGAATTATACACCATCAAAAGCAAGACCATTACCTGATTGGGCAATGAAAATTTTTAGTCCAGGTATGATT

GCGGCTGGTAATATTAATACTGAAAAAGAATTAACACAAGCGTTAAGTATGGCACAATCAAACTTACAAGTATATTTTAC

ATTATTAAGAAGAAACAAAGAAGTAGGAGACTTACAGGAAATCAAAGACGCACAAAACAGATACGCAAAACATCAAAGAG

AAAATCCACATACACCAAGAGTAATGTTAAGTTTAGGTTTACCTGAAGATGATGTAAAAGAATTTTGTACAGACGCTTTA

TTTCCTTATGTGGAATAATGGAACATTTAGACAAGTTTAAACAAGTAATAACAGAATTAAAAGATGACGGTAGATACCGT

GTATTCAATGATATCCTACGAACCAGAGGTAGTTATCCTAACGCAATCTGGTATTCAAAATACTCAATCAAAAAAATAGT

TAACTGGTGTTCAAATGATTATCTAGGTATGGGACAGCATTCCTATGTGTTAGATAGTATGAAAACAGCACTGGAGACGA

GCGGAGCGGGCGCTGGAGGGACGAGAAACATCTCCGGCACAACTCACTATCACATTGCTTTAGAACACGAATTAACTCAA

TTACACGGCAAAGAAAGTGCGTTATTATTCACTTCAGCATACAATGCTAATCAAACAACTTTAGAAACAATGGGCAAGAT

TATGCCTGACTTGTTGTTTATTTCTGACGCCGAAAATCACTCTTCTATCATACAAGGATTAAGACATAGTAAATGTAAAA

AAGAAATATTTAAACATAATGATTTAGATGATTTAGAAAGTATCTTAATGTCTAACCCTGGTCCTAAATGTGTAGTATTT

GAAAGTGTGTATAGTATGGACGGAGATATTGCACCTGTAAAAGAGATTGCTGATTTATGTAAAAAGTATAAAGCTATATC

TTATATTGATGAAGTACACGGAGTTGGTTTATACGGACCTAACGGTGCTGGTATTTGTGAACGAGATAAAGTTGATGTTG

ATATTATCAATGGAACATTAGCTAAGGCCTACGGTGTACAGGGAGGTTACATTTGTGGAAAGAGAGAGTTTATAGACGCA

ATTAGAAGTATGGCAAGTGCGTTTATATTTACAACTAGTTTATCGCCTGTACTATGTGCTGGTGCATTAACAAGTATTAA

GTATGTTAAAGACCATCCTGAATTAAGAGAAAAACTACAAGAACGAGCACAAAAAACAAAAGAAGAATTAACAAGACAAG

GTATAGAAGTATTACAAAACGATAGTCATATTGTACCAGTAATTATAGGTGACGCTAAAAAATGTAAAGCAGTTTCAGAT

GAATTACTTTATAAAGATGGTATCTATGTACAACCTATTAATTATCCTACGGTTGCTGTTGGTACTGAAAGATTAAGATT

TACACCTACACCATTTCATACAGATACAATGATATTTGATATGGTAGTTAAAGTTAAATCTGCTATGCGAAGATGTGGTA

GAACTAAATGAACATAAAAGAAGAACTTGATTGGTTAATAGTTGATGGTGCAAATGGTTTAGAAATCTTATGGTTTATGT

TAAGATACGACACAACTATACAAAGTTTGTTAGGTATAGGGGTATTATTGGCATTAGTATGTTGGTATTTTGATAAAAAA

GACGATAAAGAGACGAATTGGGATAGCGACCCTCACGGAAAATTATAAATATTGCTAAAGATAAAGGAATAAAACTATGG

CAATACCTAACACTAGACAGACACTTATCTCATATGCAAAGAGAGCATTGGGACATCCTGTTATTGAAATTAATGTTGAT

GACGACCAAGTAGATGATAGAGTTGATGAAGCAATACAATATTATCAACAATATCACTATGACGGTATCAAAAGAGTATA

TTTAAAATATCAATACACACAGGCAGATTACAATAGAATTAATACAGAATTAATACAGATACTTCTGAAGGTGCTACAAA

AAATTCTGTAACCACAACTTGGAAAACAGGAAACGGTTATATCGTTGTTCCTGATTCAATTGTAGGAGTACAAAACATAT

TTCCTTTTTCAAACAAAGGTAGTATGAATCTATTTGATGTTAGGTATCAATTAAGATTAAATGACCTTTACGATTTCTCA

TCTACAAGTGTTATCAACTATGATATTGTAATGAGACAATTAGATTTCCTAGACCATATACTAGTTGGTGAAAAACCATT

AAGGTTTAATCAACACGACAATAAATTACACATTGATATGGATTGGGGAAGCGACTTAGCAGTTGGTGAGTATCTAGTTA

TTGACGCATACAGAAAACTAGACCCAGACACTTATACAGATGTATATGATGACATTTGGTTAAAGAGATACACAACTGCA

TTAGTTAAAAAACAATGGGGTGCCAACCTCTCTAAATTTAATGGAGTAGCAATGATTGGTGGGGTTACATTAAACGGACA

ACAAATCTATTCTGAAGCACTACAAGATGTAGAAAAATTAGAAACAGAAATTAGAAGCTCGTTTGAATTAAACCCAGCAA

TGTTAATAGGATAAAAATAAAATGGCCGTTAATCATTACTTTCAAGGCGGCGATGGCATAGGTAGTCAAAATGAGAAAAG

ATTAATAGAAGATTTAATCGTTGAAAATTTAAAAATCTATGGTCACGCTGTTTACTATTTACCAAGAACTCTTGTCAATA

GAGATTTAATTCTAGGCGAAGACTCTGCGTCTAGGTTTGACGAGTCGTATCTAGTTGAAATGTACTTTGAAACGGTAGAA

GGATTCCAAGGCGAACAAGAAATAATTAGTAAGTTTGGTTTAGAAGTAAGAGACGATACAACTTTTGTAATTGCAAAAAG

AAGATTCCAAGAACAAGTTGATGATACTGCAAACTTGGTAGTAGACGGTAGACCTAACGAAGGTGATGTAATTTACTATC

CTTTAATGAACAAGTTTTTTGAAGTTTCATTTGTTGAAGACCAAGAGCCTTTCTTTCAATTAGGTTCTTTACCTGTTTAC

AAATTAAGATGTAAAACTTTTGAATATTCAAGTGAAGAGTTTAATACAGGAATTGGTGATATAGATTCAGCAGATGATAG

ACTATCATTAGATACAAGTTTACAATATCAATTTAGACTTGAAGATGGTACACTAAATCAGTCTTCTTATTCTGGTTTCT

TACAATTAGAACAAGGTGACGCAAATGGTAATCCTCAATATGTAATACAAGAAGAATTTGATGATATTACTACAGACGGA

GACGCCGCTACAAGTATACAAACAAAATCTGTTTACGCTGATAATTTAGATTTAGATACTGAAGCAGGTTTTGATACTGC

AACGGTTTCAGATGACATATTAGACTTTACAGAAAGTAACCCATTTGGAGATGTTAAGTAATGTTCGGAACACATTTTTA

TAACGAAGGATTAAGAAAATTGACTATTGCATTTGGTCAGATTTTTAATAAGATTGTTGTACAAACAAAAGACGCAAATG

GTTCAGTTGTAAAACGATTTACGGTGCCTCTTGCATATGCACCTAAAGAAAAGTTTATTACAAGATTAACACAACAACCT

GACTTGCAAGATACACAATTTTCAACTATTCTACCTCGTATGGGTTTTGAAATATCTGGTCTACAATATGACCCTAGTAG

AAAATTAAATAAGTTGACAAAAACTAGAACACAAACAGATGATGGTTCTACTTCTTTTCAACAAAACAATATGAAGTTTA

ATTATACACCTGTTCCGTATAACATAAATTATTCTTTGTTTATCTTTACTGCAACAGCAGAAAACGGATTACAGATTATG

GAACAGATAGTTCCGTATTTTCAACCTGATTATACGGTGACTATAAATATGATACCAGACTTAAATATTAAGCGTGATGT

ACCTATTGTTATAGGCAACATTGCATACGAAGATAATTATGACGGAGACTTTAATACAAGAAGAGCAGTAATTTATACGA

TTAACTTTACTGCAAAAACTTATCTATATGGACCATCTACAAATCAAGGTGTTGTTAAAAGAGTACAAACAGACCTTGGT

ACAGATACAATAGATAAAGCAAGAGAAGAAAGAATAGTGATAACACCAAATCCATCTTCAGCAAAACCTGGTGATGATTT

TGGATTTACAACAAACATATCATTCTTCAATGATGGAAAGAAATATGATCCAACAAGTGGAAGTGATACATAATGAGAGG

CAAAAATGAACAAAGAAGATATACTAGTATTAGACAATGTTTTACCAGAAGTTGTAAATAACAGCTTTCAACAAAACATT

ACAAGATTGGCATATATCCTATCTATGGATATTCTACCTAATCAAATGGACAATAAAGGTATTATGAGTGATGAGAATAC

ATTTACATCTACACAAATGGTACACAGAGTCTATTTACATAATCAACCTCAAAAAGGTCCACAAAATCCTGCTATTGAAC

CGATAAAACATTCATTATCTGAAATGGTTGGTAAAGCAGGTCTGTTAACTAAAGACTTTGATAAAGTAGAATTGTTAAGA

GCAAAATTTAATTTAATGTTTCCACATCCTGATTTCAAAGATGGTCAGTACAATGTGGCACACATTGATGACGAACAAGA

AGAACATCTTGTTTGCATTTATTATCCACAAGATACAGATGGTGATACCGTATTGTTCAATGAGTTTTTTAATAAAGATA

AGAAACCAGAAAAATTAACTATTGCAAAGAGAGTTGAACCAAAAGCAAATCGTTGTGTAATTTTTAATGGTTGGAGATTT

CACGCAAGTAGTAATCCAAGTGCATATAATAATAGAATAGTTTTAAATACAAACTTTAGGATTGTAAATAATGGGTAAAC

TAGAAGACAAAGTAAATGATATTTTAGGTATCAAAGAAGAGAGTACTCCTGTAGCAGAATTAATGTTGCAAGAGAAGTCT

GTACCTGTACCTAGAGTTGAAGACCCAAAGAAAGATGATATAGAAAACGATTACAAATATAGTAGAGAGAATTACTATAA

TTTAATTGAACGAGGACAAGACGCAATACAAGGCATTTTAGATGTTGCAAAAGAAGGGCAACACCCGAGAGCATACGAAG

TCGCAGGTGCATTAATTAAAAATGTAGCCGACACCGTTGATAAATTACAAGACTTACAAAGTAAATTATCTAAACTAAAA

GATGTACCTAATAAGACAACTAATAACATTAAGAATGCTTTGTTTGTAGGTTCAACTGCTGAACTACAAAAACATTTAAA

AGATAAAAAGTTTGATGAGAATAATAGAGACACAGCAAACGATCCTTTTAAAGATACACCAATTGAAGGAAAAGATTAAG

TTATGTCTGACGCATATCTAGGTAACCCTAATCTAAAAAAAGTAAACACACCACAAGAGTTTACTTCGGACGAGATTAAA

GAGTTTAAGAAATGTGAAAACAATCCTATATACTTTATGAAGAAGTATGTACAAATTGTTTCACTTGACGAAGGACTAGT

GCCTTTTGATATGTATCCTTTCCAAGAAAAAATTGTAAATACAATACACGAAAATAGATTTACGATTTGTAAACTACCTA

GACAATCAGGTAAGTCTACAACAACTATATCATATCTATTACACTATGCGTTGTTTAATCCTAATTGTAATATNGCAATT

CTTGCNAACAAATCTTCTACTGCAAGAGATATATTAGGAAGACTACAACTTGCATATGAGAATTTACCAAAATGGTTACA

ACAAGGTGTGTTAAACTGGAACAAAGGTAATATAGAATTAGAGAATGGAAGTAAAGTAGTAGCGGCCGCAACATCTTCAA

GTGCTGTCCGAGGAGGTTCATATAACATTATCTTCCTTGACGAGTTTGCTTTCGTACCAACAACTATTGCTGAACAATTT

TTTAGTTCCGTTTATCCTACGATTACTTCTGGTAAGTCAACTAAAGTTATTATTGTTTCAACTCCTCACGGAATGAATCA

ATTTTATAAACTATGGGTTGACGCTGAAGCAGGACAAAACGATTACATACCTATTGAAGTATCTTGGAGAGAAGTACCAG

GTAGAGACGAAAAGTGGAGAGAAGAAACAATAAGAAACACTAGTGAGTCTCAATTTGCTAGTGAGTTTGAGTGTGAGTTT

TTAGGAAGTATTGATACACTTATCAGTCCTGCTAAAATCAAAGCGACACCGTATAAGACACCACTTAAAACAAATGGACG

ATTGAGTATCTTTGAAGAACCTATAAAAGGCCATACTTACTTATGTACGGTTGATGTTGCCAGAGGTACACTAAAAGATT

TCTCAGCGTTTATTATATTTGATGTAACCGATTTACCATATAAAGTTGTTGCAACATTTAGAGACAATGAAATTAAACCT

ATATTGTTTCCTGAAATGATTGCGAAAGTTTGTACTCAATATAACAAAGCACATATACTTGTAGAAGTCAATGATATTGG

CGCTCAGATTTCAGATGGTTTACATTATGAATTAGAATATGATAATATACTAATGACTACACAAAAAGGTAGAGCAGGAC

AAATACTTGGTGCAATGTTCAGTCAAAGAGGTTCACAATTAGGTGTACGAATGACTAAACAGATTAAGAAAATGGGTACT

GCTAACATCAAAGCGATTATAGAGTCTGATAAAATAATTATTAATGACTTTAATATTATTGGAGAAATGTCTACCTTTAC

AAGAAAAAATCAAAGTTGGCAAGCTGAAGAAGGCTGTAATGATGACTATATGACTTGTCTAACTATATTAGGTTGGGTTG

CAAATCAAAGGTATTTCAAAGAAATGACTGATAGAAATATCAGAGCAGAAATGTATAAAGAACAAGAGAAGTTAATAGAA

CAAGATATGGCGCCGTTTGGGTTTGTTGATAATGGTGTTGATACACCAGAAGAACAACCATTTTCAGATGAATATGGCCA

GGTATGGCATCCCGTGGTACGCAAAGGTAGTTAATGAAGATCCCCTATTTGATAAATATAAACGATTGAGAAATTTGAAT

ATGGGCGTATGAATAATACGAATTTTGACAAAGGAAAACATTATGTATTTTTATAAAAATACAAACAATAAAATAGAGGA

GAAAACCTAATGGCATTTCAAGTATCACCAGGTGTTCTCGTACAAGAAAAGGATTTAACTAACATAATTCCTGCTGTTTC

TACTAGTATTGGAGCTTTTGCTTTCAATTCTACAAGAGGTCCAGTTGGAGAGATAACGCTTATCTCTTCTGAACAAGAAT

TAGTTAGTATCTTTGGGAAACCTACTGCAAGCAACTTTGAAGAGTATTTTACTGCTTCATCTTTCCTTCAGTATTCCAAT

GCTCTGAAAGTTGTACGAACTGAAAACACTGGAATTTTAAACGCTGTAACCAATAGTGGTTCAGCAGTACTGGTCAAAAA

TACTGACCATTACAACTCAACATACTTAGCAGATGGTGCTTACACAGGTATTTCTGGTAGGGAGTTTGTTGCTAGAACAG

CAGGCGCTTATGGAAATGGATTATCTGTTTCTGTATGTCCTTCTGCAACTGCATATGAGCAAGAAGCGGTAACAACCGTT

AACGATAGTGCTGTATCAGTTGGCGACACAACTATAACAATGACAAGTGGAACTAACATTANTGTTGGTGACATACTTGC

ATTTTCAACAACAGCCGCNACTAACGATTATGATGACGGAATTGAATACGAGGTAACAGCCGTAAATTCTAACGACATTA

CAATTAAGAAAAGAGTTGGTGCTGGTGGTCTAACAAGAGTTGTCATAGACGGCGCTAATGTTAGAAGAAGATGGTCACAT

TACGATTCAGTAAATGGTGCACCTGGAACATCTCCAGATGTATTAGCTGCTGGTGGTAGTGATGATGAATTACACATTGT

TGTAACCGACGCTGATGGTTCTATATCAGGAACTAAAGGCGAAGTACTAGAAGTATACGAAAAAGTATCAAAAGCTAAAG

ACGCAAAAGATAGTGGTGGTTCTAATAATTTCTACCCAGAAGTTATTTACAAAAAATCATCTTTTATCTATTGGGGAGAC

CATAACGGAAACGGAACTAATTGGGGTTCAGTAAAGACAACTGCTTTCACTGCTGTTTCAGGACCTATTGCATTAACATT

CGGAAACGGTGTTGATGGAACGGTAACTGACGGTGCTAGAAAGTCTGCATTTGAATTATTCCAAGATAGTGAAACCGTTG

ATGTAGGATTGATTATGGCTGGTAACGCAAGTCTGAACTTGATTGGTGATTTAATTACAATCGCTGAAACAAGAAAAGAT

TGTATAGTATTTGCTAGTCCACAAAGAAGTGATGTAGTTAATATTGCTTCTGCTATAACTCAAACTAAAAATGTACTTGC

ATTTTTCAATGCTGTACAATCATCTAGTTATGTAATCTTTGATAGTGGTTACAAATATATGTATGACAGATATTCTGATG

TATATAGATATGTACCATTAAACGGAGATATGGCTGGTTTGTCAGCAAGAACTGATTTAACTAATGACGCTTGGTTTAGT

CCTGCTGGATTAAACAGAGGTATTATTAGAGGGGCAGTTAAACTTGCTTATAGTCCAAACAAAACTCAACGAGACGAACT

TTACAGAGCGAGAATCAACCCAGTTGTTTCTTTCCCTGGTCAAGGTATTATCTTGTTTGGTGATAAGACTGGACTAACAA

CACCATCTGCATTTGATAGAATAAATGTACGAAGATTGTTTATCGTTTTAGAGAAGGCGATTGCTACAGCTTCTAAATTC

CAACTCTTTGAATTCAATGATGAGTTTACAAGAGCTAACTTTAGAAACCTAGTAGAACCTTTTTTAAGAGAAGTACAAGG

TAGACGAGGTATCACAGACTTTTTAGTAGTATGTGATGAAACTAATAACACAGGCGAAGTAATTGATAGAAACGAATTTA

TTGCTGAGATTTACATTAAACCAGCAAGAAGTATCAACTTTATCACATTATCTTTTGTCGCAACAAGAACTGGCGTGGCT

TTTTCAGAAGTCGCAGGTTAGTAAAGAGGAGAAATAAAAAATGGCAAACATTAATGACTTCAAAACTAAACTTGCTGGCG

GCGGCGCTAGAGCAAACCAATTTAAGGTAACAATGCCTTTCCCTGGTTATGCACAAGTTGGTGGAGAAACAGAAGAATTG

GCGTTCTTATGTCAAACAACTACAATCCCGGCAATGAATATTGGAACTACTACGGTTAACTTCCGTGGAAGACCTGTATA

TTTGGCGGCTGATAGAAACTTTGAACCTTGGACGATTACGGTACTTAACGACACAAACTTTAAATTAAGAGACGCTTTTG

AAAGATGGCAAAATGGAATCAATAATATGTCTGATAACGAAGGATTAGTTAATCCAGTAGATTATCAAGTAGACGCTTTT

ATTGACCATTTAGACAGAAATGGCTCTACTATTAAATCATACACTTTAAGAAGTTGTTTTCCAACTTCAATCGGTGCTAT

TGATTTGAATATGGAACCAACAGAAGCAGTTGAAACATTTGAAGTATCGTTTAGATACCTTTTCTTTGAAGCAAGAACGA

CTACTTAATAGTTGAATAAATATATAATAAAAAGTAAACTTGTGAGGAAATTATAATGGCGGAACTTTTCGGTTTTCAAA

TTACTAGAGTTAAAGATACTCCAGACCCGAAGCAAAGTTTTACTCAACCTAAGGCAGATGACGGTACACAAACCGTCGCT

GCCGGAGGTTACTTTGGACAATACCTTGATATGGAAGGTAATGCCAAGACAGAGCAAGACTTAATAAGAAGGTATAGAGA

GATTTCAATCCATCCTGAATGTGATATGGCTGTTGAAGATATTGTCAACGAGGCTATAGTAGCAAACGAGATAGATAGAG

ACCCGGTGCGAGTAGATTTATCTGACACGGACTTTAGCGATAAAGTTAAGCGTAAGGTTGAAGATGAATTTAAAGAAATA

CTAAGGTTAATGAACTTTAGTACAAAAGGACACGACATATTCAGAAGATGGTATGTTGATGGAAGAATTTACTATCATAA

AGTTATTGATAGAGAATCACCTGTAAAAGGTATAACAGAATTAAGATATATTGATCCTCGTAAAATTAAAAAAATACGAG

AGATTAAAAAAGGTCGTCCAGTTGCTATGGCAAACATACAAGTGGTACACGACTACAACGAATATTTTTTATACAATGAA

AAAGGTGTTGCAGGACCTGGTATGGCAAGTGGTGGTATTAAGATTGCCACAGACGCTATCTCATTTTGTCCAAGTGGATT

AGTAGACTTGAACAAAAATATGGTTATGGGTTATATGCACAAGGCAATTAAACCTGTCAATCAATTAAGAATGATTGAAG

ACGCTGTTGTTATTTACAGAATTGCAAGAGCACCTGAAAGAAGAATATTTAAAATTGATGTAGGTAATTTACCTAAAGTA

AAAGCAGAACAATATCTCCGTGATGTTATGGCAAGATACAGAAACAAACTTGTCTATGACGCAAGTACAGGAGAGATTAG

AGACGATAGAAACTATATGTCTATGCTTGAAGACTTTTGGTTACCTAGTAGAGAAGGTGGAAGAGGAACTGATATTACTA

CATTACCTGGTGGACAAAACCTAGGTGAAGTATCTGATATTGAATACTTCCAGAAAAAATTATACAGAAGTTTAAATGTA

CCAGTAAGTAGATTAGAAGGAAGTCAAGGTTTCAATTTAGGTAGAACAACTGAAATTACTAGAGACGAACTTAAATTTAC

AAAGTTTGTACATAGATTAAGAAAGAAATTTACAGATTTATTTAATGACTTGCTTAGAACTCAATTAGTTTTAAAAGCAG

TTATAAATGAAGAAGATTGGCAATCAATAAGTGAAAAAATTAAATATGATTTTCTTGCTGATGGTCATTTCTCGGAACTA

AAAGACGCTGAACTATTAAGAGAAAGAATAGCATTAGCAAATGATGTGAGAGATTATGTTGGTAAATACTTTAGTGTAAA

ATTTGTACGACAAAGTATTTTAAAACAATCTGAAAGAGAAATTGAACAAATTGATAGTCAAATTAGAAAAGAAATTGATG

ATGGTCTTATCGCAGCTCCACAAACTAGTGTACCTGACGACAATGACATTATGTAATATAATAAGGAGAAAATAAAATGG

CAGACAATGAAATAAGTAAAGTAGATACTTTTGTAGACCAATTAGCAAAAGGCAACAACGCAGAAGCCGGTGAAGCTTTT

AAAGACGCTTTAAGAGATAAAGTCGGAGACGCATTAGATACAAGTAGAAAAGATTACGCTTCTTCTCTATTTCAAAGTGC

GGCTGATGTTATGACTGGACAGACAACAGATGTAGTTGATACTACAAATGCTGCTGAAGGTCATTCAGACGCAAAACCTG

ATGTTGCTACACCTATTGGACAAAGTGCTACACAAGACGAAGTACAACAAGCATTTAACCAGGCAACACCTGGTAACACA

GGAGAATAAAAATGGCATTAACGGTATCAAGTATTGTAGGACATACATCTGGTTACATCAAAAACGATAGATATAACGCT

CTATCGCCAGCGATGAAAACTAGTGTAGAAACTTTAGTAAGTGGCCTTGACGCAATTGATTGGAAGCAACCACAAGATTT

AGTTGACATTATTGAAACTAAAATCACAGAAGTTGCTGCCGGCGATAGCGATGTAGAGACTGCATTGACAACATATTTTT

CGGAGTAATTAAATGGCATTAAGTATTGTCTCAAAGGTAGACGATACCACTAAAGCTATTATTACGGCTAGTGGTGCGGA

CAACGAAAGTGGAACATTATATTCTGCTGGTCAATCTGTATCTTTAGCGAATGTATATTATGAGATTAGAGGAACAGGCA

CAGCGACTCTTAAACTCGGAGACCAATCTTTAAGTTTAACAGGGTTTGGTAATTGGGGATTAAAAGAAGGTGAAGCTCGT

AAAGTAATTGAACAAGAATTAAATACGGCAACAACTTTAGAGATTACGACAGACGCTAATGTTTCAAAATTTAATATGGC

TGTAGAAGTACAGAAAGAAACGGAGACAAAATAAAATGGCAGATTTGGTAACACAACAGATAGTATCGGATACAGCAGGT

GTTAAGTATGTTGTAAAACAAACTAACTATTCTGACGGCACAGGTGAAACAAACACCGTGATTGCTAATCCAACAACTTC

TAACTTTATGACAGCAGATGGTACTAAAGAGATTGCGAAAGTGTGGTATTCTATCAATACTGCAAACAGAAAATCAGCAG

TAGAGATTGCTTGGGGAGGCGCTACTGAAAATACAACATCATTATTATTATCAGGACAAGGGTATTTAGACTTTAGGGAT

GCTGGAAATGACATAGTAAACAATGCGACAAACCCTAATGGTTATGTCTATTTAAGTACTAAAGACTTTGCATTGAACGA

TAATTATACGATTGTTGTTGAATTTAGATAAGAATTATTATAAATATTAGGAAAGAGAGAGATAAACTATTATGAAATTA

ATTACAGAAACTTTGGAAAATGTAGAGTACATTACCGAAGAAGTTAAAGGTAAAACGAATTACAAAATTCGTGGTGTATT

TTTGCAATCTGAAATCAAAAACAGAAATGGCAGAGTCTATCCTAAGCAAACACTAGCTAATGAAGTTAGTAGATACAATA

GAGAATTTGTGGAACAGAAAAGAGCGTTTGGTGAGTTAGGACATCCTGATGGACCAACGGTTAACTTGGAAAGAGTAAGT

CATATGATTACTAAACTCTATCCAGACGGTAACAACTTTATCGGTGAAGCAAAGATAATGGACACTCCATACGGTAAGAT

TGTAAAAAATCTTATTGATGAAGGCGCTAAGTTAGGCGTTTCTTCTCGTGGTATGGGTTCATTAGAGAGAAGTCGTGGTG

GTGAAGCAAGAGTTGGAAACGACTTTTACCTTGCAACTGCAGCTGACATTGTGGCAGACCCATCTGCTCCTGACGCTTTC

GTAGAAGGCATTATGGAAGGAAAAGAATGGATTTGGGACAATGGTGTAATAAAAGAAAGAGATATTGAAGAGTATAAACA

ATACATTAAGGAAGCAAAAAGACTAAAACTTGCCGAAGCAAAAGCAGAGGTATTTAGTAAGTTCCTTAAAGGATTGTAAT

ATTATAAATATCTTATAACAAAACAAGAAAATAATTATTTTTTTTAAAAGAAATAAGGAGAACTTCAATATGGCCGAGAC

AGAAAAACAGGTTGTAGAAATGACGGCTCCAGACGCTCCTAAAAAGAACGCTGTAGCTGCTGAAACTTCACCACTATCTA

ATAGTGCTGAAGATTTAGGTGCTGCTGTAGTAAAACCTACAGATAGTAATCCAGACGCAACTAAAAAAGTTAAAGAAGTT

TCAGGTGACGCTCAACAGAAAAATTCTGGTAGTGCGGATCCAATGCCTTCTGTAAAGAAGGAAGAAACTGATTCTGAAGG

CGAGAAGATTTCCGAGGGAGAAATGCCTGACGGTCTGAAAAAATTCCTAGATAAAAAGAAAGATAAAGAGTCAAAAGAAG

AAGGCTATAAGATGAAAAAAGAATCTGAAGCTGAAACTAAAGACGCTAAATCTGCTGAAAAATCTGAGGAATCTACTGAC

CAGAAAGCTAAAGACATTGATGTAAAAGAACACATTGACGCTTTGACCTCTGGTGAATCAGACTTGTCCGAAGAATTTAA

AACAAAGGCTGCTACTATTTTTGAAGCTGCAATTACTAGTAAAGTAAAAGAAATTGCTGAAGAAATGGAAGTAGACTATA

ATAAGAAATTTGAAGAAGAAAGCTCTAAAGCTAAATCTGAACTAGTTGAGAAGGTAGACAATTATTTGAACTATGTTGTC

AACGAGTGGATGAAAGAAAACGAACTTGCTATTGAAAAAGGTATCAAGGGCGAAATTGCTGAGGACTTTATTTCTGGTCT

GAAAAAACTTTTTGAAGACCACTATATAGATGTACCTGATGAAAAATATGATGTGTTAGAAGACCAAGCTTCAAAAATCG

AATCGTTAGAAAATAAACTTAACGAACAGATTGCGAAAAATGTTGAATTGAATAGTAAGACTAACTTACTTGAAAAATCT

GACATTTTAGCTGATGTTGCTTCGGATTTAACAGATGTCTCTAAAGAGAAATTTGCTAAACTAACTGAAGCAGTTGAATT

TTCAAACGGTGAAGATTTTAGAAACAAGGTAACTACTATCAAAGAAAGTTATTTTGGTGCTAAAAAAGAAGCTAATTCTG

ACAGCGAAGTAGATAATGCGGTAGCTGATAATGGCGGTGTAGATTCTACACAAGATTTATCTAATGCAATGGCTGCTTAT

ACTACCGCTATTAGTAAAACAAAAGACTTGAAACTTTAATGTTCAAGTTAATAAAATAATAATAGGAGAGAGGAACAAGA

TATGTACTTATCTGAAAACTACCAAAAAAAGTGGCAGCCAGTATTAGAGCATCCTGATTTACCAAAAATCACGGATACTT

ATAAACGAGCTGTTACCAGTGTTATCCTTGAAAACCAAGAGAAAGCACTAAAAGAAGACGCTCAGTTTATGACTGAAACT

GCGCCTACTAACGCTACTGGTTCATCTATCGCTAACTGGGATCCAATCCTAATTAGTTTAGTTAGAAGAGCTATGCCGAA

CCTTATCGCTTACGATATTGCCGGCGTTCAACCAATGTCTGGTCCAACTGGACTTATCTTTGCAATGAGAAGCAGATTTA

AGGCTCAAAACGGAACTGAAGCATTATTTGACGAAGCTGAATCACAATTCTCAGCTGCGAAAACTACAGCAAATGTACCC

GGTTCTGCTGGAACATCATCTGCTGGAGAAACAAATCCTGCTGTACTTAACGACTCGTCACCTGGAGCATATACTGCTGA

AGGTGGAATGTCTACTGCTACGGCAGAAGCATTAGGAGACTCTGCGAACAATAGTTTTGCTGAAATGGCATTCTCAATTG

AGAAGTCAACGGTAACTGCTAAGTCAAGAGCTCTAAAAGCTGAGTACACAATGGAACTTGCACAAGACCTTAAAGCAATT

CACGGTTTAGACGCTGAGTCTGAATTAGCGAATATTCTTTCTGCTGAAATCCTTGCTGAAATTAATAGAGAAGTTGTAAG

AACTATCTATGTTAATTCAGAAAAAGGTGCTCAAACTGACACAACTGCTGCTGGTATCTTTGATTTAGATACTGACTCAA

ACGGTAGATGGTCTGTTGAAAGATTTAAAGGACTTATGTTCCAACTAGAAAGAGACGCTAACGCTATCGCACAAAGAACA

AGAAGAGGAAAAGGTAATGTTATTATCTGTTCTTCTGATGTTGCTAGTGCATTACAAATGGCTGGTGTTTTAGACTATAC

ACCTGCATTGAACAACAATTTATCTGTTGACGATACAGGAAACACTTTTGCTGGAGTATTAAACGGCAGATTTAAAGTGT

ACATTGATCCATATAGTGCAAACCAAGCAAGCAAACAATTTTATGTTGTTGGTTATAAAGGTACTTCACCTTATGACGCT

GGTATGTTCTACTGCCCTTATGTGCCATTACAAATGGTTAGAGCAGTTGGTCAGGACACTTTCCAACCGAAAATCGGTTT

CAAAACTAGATACGGCTTACAAGCAAATCCTTTTGCTGAAGCAGGTTCTGGCGATGCAGCTGTTGTTAACGGTTCAGGTT

CTGCAAACGCTAACAGATACTACAGAAAAGTACAAATCACTAACCTTGCGTAATTGCAAATAGTGATTGTATAGTAGACA

ATCTTACGAAAAAAGGCGATGTTAAAGTCGCCTTTTTTTTAGCCCAAATTATAATATCAGTATATTCAGATTGCTAGGGG

TTTATTAGGTCTTATAAATAGTATTATGACAAATACAAACGCAATAACACGACAACCAACACAACTTGATTATGCGTCAC

CAACGCAATTTAAGTTTAATATCATAAAGCTTCCAAAGGTAGAATACTTTTGTACAGAAGTTAATATCCCAAGCCTACAA

ATGACCAATGCAACACAGGTAACTCCATTGAGAGATATACCTTTGCCTGGAACTAAACTTGACTTTGGAGATTTAGTACT

TACATTTATGATAGACGAAAAGTTAGAGAACTATGAAGAGATATTTGGTTGGTTAAGAGGTCTAGGTTTTCCTGAAGACC

ATAGCGATTATGCTAATTTGTTATCTTCTGGAAGAGATAGATTTCCTACACAAGGCAAAGATAGTCAAAATCTAAATGCT

GGTAGAGAAGGAACTGCTGCTCCTCAAGGAGGTATTTTGTCTGACGCCACACTAACAATATTAAGTGCAAAGAATAATCC

GATTAAAGAAGTAAGATTTAAAGATATATTTCCTGTTTCGTTAACCGGCGTAAATTATAGTCAACAAGCAAGTGATGTTC

AATACTTAACTAGTAGTGTTAGCTTTAAATATACGACTTATTCTTTTGCTGAACCCGGCAAACCTTCTACGCTTTATACC

TCATAGAAGCTTGACAAATAACTAGATTTAGTATATAATTATACTTAAAACTAGAGGAAGATACTAGATTATGACATTAG

AAGAATTACAAGAACTTTCAGATAAGAAATTAAAAATCAATGATACTGAGCTTGATATTGAAGCTCTTAAAACACCACAA

CTACATAATGAGTTTTTGAAACATTACAATAAGTTTAATCTTTTACTTACTAAAACTGAAAGTGAGTTAAGGATTATAAA

ATTACATAAATGGGAATATTACACAGGGAAAGCGGACCCAGCAGTTTATCAAACTAAACCATTCAATTTAAAAATTCTAA

AGCAAGATGTTGATAAGTACATTGAAGCAGATGAAGATTACATAAAGATAAAACAAAAAGTAGATTACTTAAAAACTATA

TGTGATTATCTGGATAAAACAATCAAACAAATATCAAATCGTGGATTCCTAATCAAGGATGCTATTGAATGGCGTAAGTT

TACTTCTGGCGCTATTTAATAGATGGTAGAAAATCGTTATTTAATATTAGATAAGAAAGACGAAGTATATCTTTCAATAG

AAGCCGAGAGCGATATTCGTAGAGAACTATCGGAGTTTTTTACATTTGAAGTTCCTGGTTATAAGTTTATGCCTCAATAT

AGAAATAGATATTGGGACGGAAAGATAAGACTTTTTAAGTATGCAAGTGGTGAGATTTACTATGGTCTCTTACCATATAT

CAGAAAGTTTTGTGAAGATAACAATATAAAAATTGTATCTAACATAAAAGAGAAAGCAAAACCATTAGATAAGTTAGAGT

GTGCTAGATTTTGTAAAGCATTAAAGATACCTAAAATTACTATTAGAGATTATCAATTCAATGCTTTCTATCACGCAATA

CAAGAAGATAGATGTTTATTACTATCTCCAACTGCTAGTGGTAAATCACTTATTGCATATCTTATATTGAGATTTCAACT

ATTAAGAATTAAAGAAAAGAAAGCAAACAAAGTATTAATCATTGTACCTACAACATCACTAGTAGAACAATTATATAAAG

ACTTTGCTGACTATGGTTATAATACTAAACACATACATAGAATATATCAAGGACACGACAAAGACACAACAAAGAAAGTA

GTAATATCTACTTGGCAATCAATATATAAACTACCTAAAAAGTGGTTTGCACAATTTGGTTGTATACTTGGTGACGAAGC

ACATTTATTTAAATCCCAGTCCCTTACAAGTATAATGACAAAGATGACTAATTGTAAATATAGAATAGGTATGACTGGTA

CACTAGATGGTTCAAAGACACATAAACTAGTGCTAGAGGGTCTATTCGGGGCTGTAAATAGAGTTGCGAGTACAACTGAC

TTGATAGAGAAGAAACAACTAGCAGACTTTAAAATACATTGTTTAATACTTAAACACGGAAAGAATAGTAAAGACTTTTT

AAAAGATAAGAACTACCAAGAAGAAATGGATTTCTTATGTGCTAGTAAAGCAAGAAACAAATACATTACGAACTTGACAA

ATGGTCTTCAAGGTAATACACTATTGTTATTTCAATTTGTAGAAAAACACGGTAAGGTATTACAAGAACTAATTGAAAAG

AAAGTAGATGATGGTCGTAAAGTATTTTTTGTATATGGAGGAGTATCAGCAGATGATAGAGAAAACATTAGAGCAATTAC

AGAAAAGAGTGATAACGCTATTATTGTGGCTTCGTACGGTACCTTTTCAACGGGTATCAATATACGAAACTTACATAATA

TTATTTTTTCTAGTCCTAGTAAGTCTAGGATAAGAAACTTACAATCTATTGGTCGTGGTTTAAGACTAGGTGATAACAAA

GTTAATGCGACACTATATGATATATCAGATGATGTTTCATACGGAGAAAAAGAGAATTATACATTACAACACTTTAGAGA

AAGAATAAATATATACAACGAAGAAAACTTTGATTACGAGATACATAATGTGGAGTTAAAGGAGTAGTATGGACAAAAAC

AACGAAACTAAACCAGAAATTAAAATCGTAAAGATTATGAATGGCACAGATATTGTTTGCCATATACCTCAGGTAGCAAA

TCAACAACGAAATCCGTTGCTGACATTAGACAAACCATTAGAAATAAAATATGTACCACAGATTACTAATCTTGGTATCA

AAGACTACATAGCGCTTGTGAAGTGGGCAGCTTATACAAATGACCAATTGGTTACTATTCCTAAAGATAAGATACTGACT

ATTACAAATGCAAGTGCTGAAATGATTAAATCATATACACAAGTTATTAGCGACTACACAAACCACGACAAGGTTATGAG

ACGAGAAGACAATCCGAGAACTGAAAGACTTATGGATAGAGAAGTAATGCGAGAAGAAGAATTGGAAGAGTATGATGAAA

TATTTGAGGCCTTTAATGACATTAAAAAGAAAAGTACAATTCACTAGACGCTACTCTATAGACTCTATCTCTACCCAGCG

ACACGCTGATAATAACATAGGATCCAATATATGTCAAGCGCCTGTGCCAATAAAATTAAAAGTGTTAGGTGCTTGACTTA

TTTAACAACATATAGTATAGTGAGAACATAATGAAAACTAAAACAGATAAAAAAATTGAAACGGCAGAAGTGCCTGAAAA

AAAGAAACGCATACGAACACCGGCGAAGAAGGAACACTATGTTAATAACAAAGAGTTTCTAGCTGCAATGGTAGAGTATA

AAGACAAATGTAATAAAGCAGAAGCAAGAGGCAGAAAGAAACCTCCGGTTACTAATTACATTGGAGAATGTTTTTTAAAG

ATTGCAAACCACTTATCATATAGACCTAACTTTATCAATTATACTTTTAGAGACGATATGATTTCAGATGGTATTGAGAA

TTGTTTACAATATCTAGGTAACTTTAATCCAGAGAAGTCAAACAATCCATTTGCATACTTTACACAAATTATCTATTATG

CCTTTGTAAGACGAATACAGAAAGAAAAGAAACAAACAACAATCAAACATAAGCTTATTATGGATGCAAACTATGACGAT

ATGACTTTGCAACCAGGTGATGATAGAGACTTTAAAAATCAATTTACAGAATTCTTACAAAAGAACTTACCAAGCCAAGA

ACCAACAAGTGAACCTATAGAAACAAAACCTAAAGGTGCAAAACTAAAGAGAACCCGAAAAGCGAAGATAAATTTAGAGA

ACTTTTAATTATGAAGATAGCATTGTTGAATGACACTCACTTTGGTGCAAGAAGTGATAGTCCTGCATTTATTAAATATT

TTAACCGGTTTTATGATGAGATATTTTTTCCATACTTGGAAGAGAATAACATTACAACCTTAATACATTTAGGCGATGTA

GTAGACAGAAGAAAATTTATTAACTTTAATACTGCTCATAACTTTCAAAATAAGTTTTGGAAGAGACTATGGGATATGAA

GATTGATACACATATTATACTAGGTAACCACGACACATACTATAAGAACACAAACTCTATTAATAGTATGCAACAACTAA

TTACAACCTTTGATGGTGTAAACGAACCATTTATATATGAGAAACCAAAGACGGTTGAGTTTGATGGTTTGCCTATTCTA

TTCATACCTTGGATATGTCCAGAGAATGAAGAAGAAAGTCTAAAAGCAATATCAGAAAGTCAAGCACAAATATGTATGGG

TCACCTTGAAGTTAAAGGTTTTGAAATGCACAAAGGACACTTCCAAGAACACGGTTTAGAAATGGACTTGTTTAAAAGAT

TTGAGAAAGTATATTCTGGTCACTATCATAGAAAATCAGATAATGGTACTATCTTTTATCTAGGTACACAATACGAGATT

ACTTGGTCAGATTATCAATGTCCTAAAGGTTTTCATATCTTTGATACAGATACAAGAGAACTAACAAGAATTCCTAATCC

TATCAATATGTTTAAGAAGATAGTATACAATGATAAAAAAAATTCATATAGTAATATGGATATAAGTGAATATGAAGATT

GTTTTATCAAAGTTATTGTAGAAGAAAAAACAGATGTCAACCAGTTTGGTGACTTTATTGATAGACTACATAATGATATA

CACACAAACGAAGTAAATGTTATTGAAGATAGTTATAATATCAATTCAACTGCTGATGTTAATATAGTAGACCAAGGAGA

AGATACACTATCTTTCTTACAAAATTATATNAATAGTTTAGATACTGAATTAGATAAAAATAAGATGAATAGTATAGTGA

AAGACTTATATAGTGAGGTGCAAGATAAGTGATAATATTTCATAACATAACCTGGAAGAACTTTCTTTCTACAGGTAATA

CACCAATCAGCGTAAACTTAAATGAATCACCTACGACATTAATCATAGGTACTAATGGTTCAGGTAAATCAACTTTACTT

GACGCTTTATGCTTTGCATTGTTTAACAAACCTTTTAGAATTATTAAAAAAGACCAGATGGTTAATACAATCAACAATGC

TGATACCGTTGTTGAAGTTTANTTTAGTATTGGTCCAAAGAAATACAAAATACGAAGAGGTATTAAACCTAACATATTTG

AAATATACCAAGACGGCATTTTATTAAATCAGGATGCTTCTTCTATAGATTATCAAAAGTACCTTGAACAGAATATAATG

AAACTTAATTACAGGTCATTTTGTCAAGTTGTAATTTTAGGTTCTTCTTCTTATGAACCATTTATGAAGATGAGAGCAAG

TTATCGTAGGGATGTAATTGAAGAGATACTAGACATTAAAGTATTTGCAAGTATGAACTTATTGTTAAGAAGTAAACAAC

AAGACTTGACCAAAGACATTACCACAATGAGACATCAGGTAGATTTAATTGAAAACAAAGTTAATCTACAAGAGAAACAT

TACGAAGAATTACAAGGTAGAGATACAGACGCTATCACTAGAAAAAAAGAAGACATAGAGAAAGCACAACAAGGTAAAAG

AGATTATATGGTTCGTATCAATAGTCTCAATAACGAAATTGAAACAAACAAATTAAAACTACAAAACAAAGAAACGACTA

AAAGTAAGTTTCTTCAATTACAGAAACTAGAAAGTAAGATTGATACTAATTTAAAGACACACAAAAGAACATTAAAATTC

TTTGAAGAGAATACTAATTGTCCTGTTTGTACACAAGAACTTGAACCTAGTTTCAAACAAGAGAAAATCAATGAAGAGAA

GGCTGCTGTAGATAAACTTAATGAAGGTTATAAACAACTATTAACTGAAATTACTAATACAGAAGAGAAGATACTAAACC

TTGATAAAGTATCAGAAACTATTAGAACTATAGAAACAAATGTTTCAAAACTTAATCATAGTGTTGATGAGATTAAAAGA

CATAGTGATAGAATACAAGATGAGATTGAACT

>lcl|3300003620_____JGI26273J51734_10000260|386802

CAACAAGGTACTGGTTATGATGGTGCTCCTGGTTCTGCAATGTCACCTGACGGATTAGAAGATGTACACTTTATCAAAAT

CGCAATGAAATTAATGAACGAAGATTCAACACATACTCCTTGCGGATTAACAGATGATGGAGATGTATACACTTGGGGTT

ACAATGGTTACGGTGAAGTCGGAGACGGTAGAACAGATAACGCATACGGTCCTAAGAGAATACCAAGAGAATTCTTTAAT

GACGAAAAAATTATTGATATTTTAGCAACTGGTGGAGATAGTACTTCATTCTATGCTAGAACATCACAAGACAACATTTA

CGGTTGGGGAAGAAACAACATCGGCCAATTAGGAGATACAACAACGACTGACAAATACAGACCAGTATTAATGACTGGAT

TTAATGCTGCTGACAATGGTGGTATCGCTGTATGGCAAGGTGACGCTCACTCATCTAATTCTGCTTTCTACATATTAGAC

GGAAACGGATTTATATGGGCAACAGGTTACAATGGTTATGGTAACTTTGTTGATAACTCAACATCTAATAGAACACAATT

AACTCAATCAACTGCTTCTCCTAATGGAGATATAGCAGACTTCTGGACAATGTACTGGAATGGATATCATACAACATTTA

TGAGATTGAAAAATGGTGAAACTTGGACTGCTGGACATAGTGGTGGATACTACAACTCTGGTGATGGTGGAACTGGAACA

AACCAGGCACCTGTACAAGTAGATAAGATAACTAACCTAAAAGAAGTTTGTATATGTAATACATATTCAGACCAAGGTAG

AAGTTATTGGTTAACAGATAATGGTGAATTCTTTAGTCAAGGCCGTGATGTATACGGTTCTATGCCAAATAGTGTTGCTG

GAGATAACTGGAACGGTGAAGATGGAACATACAAACCTTTCCACGCTTTTGTACCTGCAGGAACTAGAATTAGAACTATG

TGTATTCAAGGTATTGACCAATCAACTAACTATTACGGATTACAACCAATGGTTGGAACAGAAGATGGACAAGTCTTGCT

TTGGGGTTACTCAAACAACAATAACCTAGGACACCACGCTAGTGCAACTTACTCATCAACGGGTCGTTCACAAATGTGGA

ATGCTGGTATTGGTAGATAATATAAATAGAAGTATAACAAATAAAGAAAACGGAGAAAAAACAAAATGGCAAAAGTAATT

TATTCAATGACTGCTGGAATCGGTCAAGGTGACGACTATACTGCTCCTACAGGAGATACGCCAATCAGTTTAGGAGAATT

AAGTGGAAAAACTTATTTCTCAATTGATGATGGTAATACTACTATCACAACTGATGGTGCAAATGATTCTGTATACGGTG

TAGCTGTTGTAACCGACGCTGATGAAAAAGCAAGTTTACAAGCTGCTTCAAACTATGTTGCACAAGGCTTGGGAAATTTA

GATAACGAATTTATGAATGGTAAATCAATGATTGACTTATTAGCTGATGTGGCTGATGATACTGCAGGAACTAAAACTGC

AATCGCAACTCACAAAGCTGCTAAAGCTTCATTCCTATCAAACTTAGGATTCTAAAGACAAATTAAATAAAAAGAAGGTA

ATAGCAAATGGCACTAGATATACAAAACTTTAAAGTATCCTGGAAAGGAAACTGGAAAGATAAAGAGAAGTATTATAAGA

ATGATATAGTTTACTGGAGAGGTAAGTCATACAGATGTACAGAAGAGACACCTGATAACTTTACTATTTCAAGTGAATCT

ATGGTCAATACGAACTCTTATGGCCAATATCAGCCTACGGTTGTTAGACGGTCATATAGACCCGACAACAATAGATACTG

GACGCTATTGCTTGCAGGTAACGACAACATTGAAACTTGGCAGTATTGGAGACAATATGAAAGAGGTGAAATGTGTAAAG

TTGCCGATAAGATTTATCTTTGTTTGAAAAAAACAAGATATTGTAATACTTGGGTAGAAGAACACGATGGTAGACCATCA

AAATATTGGGCACTTGTTTACATTAACGAAAACAAGTGGAGTACAAGAAACGAAGTTATTTCATTTAACAACCGAGCTCC

GTTAGGTTGGAAATATAATATGGGTGTTGACACACAAGATAGTGCTGACCAAACATATAGAACTTGTACACTATGCTCAG

ATGGTTCTGATATGTGGGTAGGTTCTTCTGAAGGAACTGGTTCATCTGGATTAGGTGACGGTGTTGCTGGAAATGACGAA

CCTGGAAAACACTTTTCTACAGGATTTACATTTACTGATTGGATGGCTTCTACAGACAACCAATCTTGGAATATTAATGC

TACAGGTAGAATGACTACACCTGATGGAAAAGCACCAAGAGTAATACAAGTTAGAAAAAATCAAAATAGAACTTTCTGGT

TAATGAACAATGGTGAAGTTTACGCCGCTGGTGAAAATGGAAACTACGGTTTAGGAAATTCAGAAACTTCAGATAGAAAT

TATAGTGTAAGGGTTACTGCTAATGATACACAAGATTGGCAAGGAAACACTATAGGCAAAACATTCAATCAAACTAAAAT

GGTTAAAGTCGGTATGTCCGACGCCGGACACGACGCTGGAACATCATCTTGTTTCGCATTAGGTGATGACGGTTCAGTAT

GGGTTTGGGGTTACAATAACAACGGTCAATTAGGACTAGGTAATCCATCAATAAACAATTCAACAGACACNTCTGGTGGN

CCAACAGGTACTGCTTTCTATAGTGCCAATGTNACCAGACCAGTTAGATTACCTCAATCATACTTTGATGGAAGACAAAT

CATTGATATGTGGACTTCAGGTTCAGAAGAGGCGTGGTTCCACGCATTAGACGAACACGGTCAACTATGGGCTTGGGGAC

ATAACCAACACGGTGAATTAGGAGTAGGAAACAGAAATGGCACTTACTACTACACACACCCAACAAGAGTTGGTGTCAAC

TGGAACAGATACGGTGGAATTAAAATGTACAAGACAACTCACTCAAACGGTGGACACTCTTGTACTCACATATTAGACGG

CGAAGGCTATATGTGGTTTACAGGTTACACAACTTCAGGTGCTTGGCCAATCGGCTCACCTGGTTACACAGATACGCACC

ATATTGGTTCGTTCAGAAGAGAAGGTCACTTCTTAAATGGAGACATTGACTTCTTCTGGTGTGGTGGAGATGAAAACAAA

TGGTTATATATCAGACAGAAAACTACTGGTATGCTATGGGTACACGATGGTAACTATGGTACATACGGTGGTCGTGGACA

ATCAGTTGAAAGTAATGGATACTGGTATGCTTCAGGTGGTCACCCAGGAAGTTTCATACATATGAAAGGTCCTAAATGGG

CAGTTAATGTATGTGATGTAGGTATGAGTAGAGCTGATGGTTCTTATATGTACTCTTTCCCAATGATACTTGATGACGAA

GGATTGATTTGGGGTGGTGCTCCATATTCAAACAACGAGCAAGGTATGGGTGGTGACACTAACTTTAATGACCAATTTAC

CAACGGTGGTAGAAACGATACCGGTCAAGGTATGGAAGACAATGAAATGTTTAGAACAAGAAAGAAAATCGTATTTCAAC

CAGGTGGTGGACATAGATGGACAGACTTATTCTATTCAGGAACTGGTTCTTCAAATATACCAAGAGCTGTTAACCAAAGA

GGTCAAGTATACTGGACTGGTTATGATGGTGGTGCTTCGGTAACTATGAACTATGACTACTATTCAGAAGGTGCAAACTC

AAACCAAGTTTCTTACTTCTTCCATTTGGGTCCTAGAGACTAATATAAATAATTATACATTAAGACTTCACGGTCTTTTA

CATTAAACAATTGAGGTGAATATGAAAGCAATAGAAGAATTTGTTGAACAGGCTCGTAAAAAGTTTGAATCACAACCGTT

CATTAACGACTATCTAAACAAGAAACTTAAACACAAAGAAGCTGTTGGTACTTACTTGTACAATCAATGGGTCTATGTAT

GTCAAATAGAAGGACATTGCAAAGACGCAGGTGTACTTGATGGTATTGAAGAGATTTGTATTAAAGAAAATCTAAAAGAA

GCGTGGAAAGCAGAATGGCCTTATGACGCTAATGATATAACAAAACCTTGGGTAGAACCATCTGTAATGTATGCAACGCA

AAGTTGGTGTACTTCTATTATAGATGTTAAAGAAGATAAAGATTTATTACTCGCACACCTATATGCCTCTCATAGTGAGA

TAATGACCAATCAAGGTACTTCAATTCTCAAAGATAGACTTACAGAAAAATTTACAGAAGCATACAATCGTAGACCAGAC

GAAATGCTTAACATTATAAAACTAAATTGGGACTTTAAAATTGGTATGTCTGGTGATTTAGAAGCACACAACGAACATTT

AGAAGAAGTCTTACCTAGAATTTCTTTATTCAAAGTTGCTGCTAAAGAAATTGNTGAAGACAAATCAGGTTTAAATGATA

TGTCTGGTGGTAGTAGAGACGAATCAGAAGACCAAAAAGTAAGAGCAGAATTAATGGCAAATCAAATCTTTATAGGTGAA

ATGAATTTAGATGATGTGCCAGAAGATTATCAAAGTTTTGTAAAAGAAGACTTACATCAACTTGAACAAAAGAAAAAAGA

GACTGAAAAAACATTTGAAGAAGCCCCTAAAAGATGAAAACATTAAAAGAGTTAACTTGGGAACATCATAAAGAAGCAGA

AAGACAACACTTTGTAAAAGTATTAATGTCTGGAAAGATATTAGAAGAAGTTTACGCTGTTTATCTTTACAATCAACATC

AAGCATATAACATATTAGAAGCAGTCGCAATGGCAGACGGTTTCTTTGATGATATGCCACAATTAAGAAGAGCACCTGAA

ATCTTAAAAGATTTTAATGAACTATGGACTTGGAAACATAAACCTTGGTTATGTGAAAGTACAAAAAAATATGTTGAATA

TGTAAATAAAAATTTAATGGATAATCCTGAAAAAATAGCTGCACATATCTATGTAAGACATATGGGTGATTTATCAGGTG

GTCAAATGATTAAAAGAAAGACACCAAGTCGTAATTATTATTATGATTTCAATTTTAAGAAAGTTGATGATGGTGTACAA

AAATATAAAAGCGTACAAGAAATAAAAGACGCATTAAGATTAAAGATAGATAGTTTTCAAAAGTATTCAGACGCAAGTAC

ACTTACAGAAAATGTAAATAATGTTGTATATGAAGCAAGAGTTTGTTTTAGTTTTGCAACGGAACTATTTAAAGAAATGA

TGACATTTATTAATAACAATGAAAAGAGGTTTGGTGATGGAACGAAGAAGTAGAATATGGGAAATGCTTGAGCAACACAC

TCATAGTATTATTGCAAATTTTGAAAGAGAAGGTGAAGAGATATTTGAACCTGCAATGAAAAAGTTTAATAGACCTGAAG

AAGGTTGGGTTAATAGAGTATGGAAAACACCTGAAGCAAGAAGATGTCATTTAGATGTAGTTGACGCTAGAGACGAAAAA

GGTTTGTTTATGTTTCATTGTTGCGTGTTTCCTAATCTAACAAGTGAAGCACCTATATTTGGATTAGATGTAATCGCAGG

TGCAAAAAAGGTTACAGGTTTCTTCCACGACTTTTCTCCTCTTGCAAAAAGAGACCATTCAATGGTAGATTGGTTTGTAA

AAGAATCAAAGAATTATACACCATCAAAAGCAAGACCATTACCTGATTGGGCAATGAAAATTTTTAGTCCAGGTATGATT

GCGGCTGGTAATATTAATACTGAAAAAGAATTAACACAAGCGTTAAGTATGGCACAATCAAACTTACAAGTATATTTTAC

ATTATTAAGAAGAAACAAAGAAGTAGGAGACTTACAGGAAATCAAAGACGCACAAAACAGATACGCAAAACATCAAAGAG

AAAATCCACATACACCAAGAGTAATGTTAAGTTTAGGTTTACCTGAAGATGATGTAAAAGAATTTTGTACAGACGCTTTA

TTTCCTTATGTGGAATAATGGAACATTTAGACAAGTTTAAACAAGTAATAACAGAATTAAAAGATGACGGTAGATACCGT

GTATTCAATGATATCCTACGAACCAGAGGTAGTTATCCTAACGCAATCTGGTATTCAAAATACTCAATCAAAAAAATAGT

TAACTGGTGTTCAAATGATTATCTAGGTATGGGACAGCATTCCTATGTGTTAGATAGTATGAAAACAGCACTGGAGACGA

GCGGAGCGGGCGCTGGAGGGACGAGAAACATCTCCGGCACAACTCACTATCACATTGCTTTAGAACACGAATTAACTCAA

TTACACGGCAAAGAAAGTGCGTTATTATTCACTTCAGCATACAATGCTAATCAAACAACTTTAGAAACAATGGGCAAGAT

TATGCCTGACTTGTTGTTTATTTCTGACGCCGAAAATCACTCTTCTATCATACAAGGATTAAGACATAGTAAATGTAAAA

AAGAAATATTTAAACATAATGATTTAGATGATTTAGAAAGTATCTTAATGTCTAACCCTGGTCCTAAATGTGTAGTATTT

GAAAGTGTGTATAGTATGGACGGAGATATTGCACCTGTAAAAGAGATTGCTGATTTATGTAAAAAGTATAAAGCTATATC

TTATATTGATGAAGTACACGGAGTTGGTTTATACGGACCTAACGGTGCTGGTATTTGTGAACGAGATAAAGTTGATGTTG

ATATTATCAATGGAACATTAGCTAAGGCCTACGGTGTACAGGGAGGTTACATTTGTGGAAAGAGAGAGTTTATAGACGCA

ATTAGAAGTATGGCAAGTGCGTTTATATTTACAACTAGTTTATCGCCTGTACTATGTGCTGGTGCATTAACAAGTATTAA

GTATGTTAAAGACCATCCTGAATTAAGAGAAAAACTACAAGAACGAGCACAAAAAACAAAAGAAGAATTAACAAGACAAG

GTATAGAAGTATTACAAAACGATAGTCATATTGTACCAGTAATTATAGGTGACGCTAAAAAATGTAAAGCAGTTTCAGAT

GAATTACTTTATAAAGATGGTATCTATGTACAACCTATTAATTATCCTACGGTTGCTGTTGGTACTGAAAGATTAAGATT

TACACCTACACCATTTCATACAGATACAATGATATTTGATATGGTAGTTAAAGTTAAATCTGCTATGCGAAGATGTGGTA

GAACTAAATGAACATAAAAGAAGAACTTGATTGGTTAATAGTTGATGGTGCAAATGGTTTAGAAATCTTATGGTTTATGT

TAAGATACGACACAACTATACAAAGTTTGTTAGGTATAGGGGTATTATTGGCATTAGTATGTTGGTATTTTGATAAAAAA

GACGATAAAGAGACGAATTGGGATAGCGACCCTCACGGAAAATTATAAATATTGCTAAAGATAAAGGAATAAAACTATGG

CAATACCTAACACTAGACAGACACTTATCTCATATGCAAAGAGAGCATTGGGACATCCTGTTATTGAAATTAATGTTGAT

GACGACCAAGTAGATGATAGAGTTGATGAAGCAATACAATATTATCAACAATATCACTATGACGGTATCAAAAGAGTATA

TTTAAAATATCAATACACACAGGCAGATTACAATAGAATTAATACAGAATTAATACAGATACTTCTGAAGGTGCTACAAA

AAATTCTGTAACCACAACTTGGAAAACAGGAAACGGTTATATCGTTGTTCCTGATTCAATTGTAGGAGTACAAAACATAT

TTCCTTTTTCAAACAAAGGTAGTATGAATCTATTTGATGTTAGGTATCAATTAAGATTAAATGACCTTTACGATTTCTCA

TCTACAAGTGTTATCAACTATGATATTGTAATGAGACAATTAGATTTCCTAGACCATATACTAGTTGGTGAAAAACCATT

AAGGTTTAATCAACACGACAATAAATTACACATTGATATGGATTGGGGAAGCGACTTAGCAGTTGGTGAGTATCTAGTTA

TTGACGCATACAGAAAACTAGACCCAGACACTTATACAGATGTATATGATGACATTTGGTTAAAGAGATACACAACTGCA

TTAGTTAAAAAACAATGGGGTGCCAACCTCTCTAAATTTAATGGAGTAGCAATGATTGGTGGGGTTACATTAAACGGACA

ACAAATCTATTCTGAAGCACTACAAGATGTAGAAAAATTAGAAACAGAAATTAGAAGCTCGTTTGAATTAAACCCAGCAA

TGTTAATAGGATAAAAATAAAATGGCCGTTAATCATTACTTTCAAGGCGGCGATGGCATAGGTAGTCAAAATGAGAAAAG

ATTAATAGAAGATTTAATCGTTGAAAATTTAAAAATCTATGGTCACGCTGTTTACTATTTACCAAGAACTCTTGTCAATA

GAGATTTAATTCTAGGCGAAGACTCTGCGTCTAGGTTTGACGAGTCGTATCTAGTTGAAATGTACTTTGAAACGGTAGAA

GGATTCCAAGGCGAACAAGAAATAATTAGTAAGTTTGGTTTAGAAGTAAGAGACGATACAACTTTTGTAATTGCAAAAAG

AAGATTCCAAGAACAAGTTGATGATACTGCAAACTTGGTAGTAGACGGTAGACCTAACGAAGGTGATGTAATTTACTATC

CTTTAATGAACAAGTTTTTTGAAGTTTCATTTGTTGAAGACCAAGAGCCTTTCTTTCAATTAGGTTCTTTACCTGTTTAC

AAATTAAGATGTAAAACTTTTGAATATTCAAGTGAAGAGTTTAATACAGGAATTGGTGATATAGATTCAGCAGATGATAG

ACTATCATTAGATACAAGTTTACAATATCAATTTAGACTTGAAGATGGTACACTAAATCAGTCTTCTTATTCTGGTTTCT

TACAATTAGAACAAGGTGACGCAAATGGTAATCCTCAATATGTAATACAAGAAGAATTTGATGATATTACTACAGACGGA

GACGCCGCTACAAGTATACAAACAAAATCTGTTTACGCTGATAATTTAGATTTAGATACTGAAGCAGGTTTTGATACTGC

AACGGTTTCAGATGACATATTAGACTTTACAGAAAGTAACCCATTTGGAGATGTTAAGTAATGTTCGGAACACATTTTTA

TAACGAAGGATTAAGAAAATTGACTATTGCATTTGGTCAGATTTTTAATAAGATTGTTGTACAAACAAAAGACGCAAATG

GTTCAGTTGTAAAACGATTTACGGTGCCTCTTGCATATGCACCTAAAGAAAAGTTTATTACAAGATTAACACAACAACCT

GACTTGCAAGATACACAATTTTCAACTATTCTACCTCGTATGGGTTTTGAAATATCTGGTCTACAATATGACCCTAGTAG

AAAATTAAATAAGTTGACAAAAACTAGAACACAAACAGATGATGGTTCTACTTCTTTTCAACAAAACAATATGAAGTTTA

ATTATACACCTGTTCCGTATAACATAAATTATTCTTTGTTTATCTTTACTGCAACAGCAGAAAACGGATTACAGATTATG

GAACAGATAGTTCCGTATTTTCAACCTGATTATACGGTGACTATAAATATGATACCAGACTTAAATATTAAGCGTGATGT

ACCTATTGTTATAGGCAACATTGCATACGAAGATAATTATGACGGAGACTTTAATACAAGAAGAGCAGTAATTTATACGA

TTAACTTTACTGCAAAAACTTATCTATATGGACCATCTACAAATCAAGGTGTTGTTAAAAGAGTACAAACAGACCTTGGT

ACAGATACAATAGATAAAGCAAGAGAAGAAAGAATAGTGATAACACCAAATCCATCTTCAGCAAAACCTGGTGATGATTT

TGGATTTACAACAAACATATCATTCTTCAATGATGGAAAGAAATATGATCCAACAAGTGGAAGTGATACATAATGAGAGG

CAAAAATGAACAAAGAAGATATACTAGTATTAGACAATGTTTTACCAGAAGTTGTAAATAACAGCTTTCAACAAAACATT

ACAAGATTGGCATATATCCTATCTATGGATATTCTACCTAATCAAATGGACAATAAAGGTATTATGAGTGATGAGAATAC

ATTTACATCTACACAAATGGTACACAGAGTCTATTTACATAATCAACCTCAAAAAGGTCCACAAAATCCTGCTATTGAAC

CGATAAAACATTCATTATCTGAAATGGTTGGTAAAGCAGGTCTGTTAACTAAAGACTTTGATAAAGTAGAATTGTTAAGA

GCAAAATTTAATTTAATGTTTCCACATCCTGATTTCAAAGATGGTCAGTACAATGTGGCACACATTGATGACGAACAAGA

AGAACATCTTGTTTGCATTTATTATCCACAAGATACAGATGGTGATACCGTATTGTTCAATGAGTTTTTTAATAAAGATA

AGAAACCAGAAAAATTAACTATTGCAAAGAGAGTTGAACCAAAAGCAAATCGTTGTGTAATTTTTAATGGTTGGAGATTT

CACGCAAGTAGTAATCCAAGTGCATATAATAATAGAATAGTTTTAAATACAAACTTTAGGATTGTAAATAATGGGTAAAC

TAGAAGACAAAGTAAATGATATTTTAGGTATCAAAGAAGAGAGTACTCCTGTAGCAGAATTAATGTTGCAAGAGAAGTCT

GTACCTGTACCTAGAGTTGAAGACCCAAAGAAAGATGATATAGAAAACGATTACAAATATAGTAGAGAGAATTACTATAA

TTTAATTGAACGAGGACAAGACGCAATACAAGGCATTTTAGATGTTGCAAAAGAAGGGCAACACCCGAGAGCATACGAAG

TCGCAGGTGCATTAATTAAAAATGTAGCCGACACCGTTGATAAATTACAAGACTTACAAAGTAAATTATCTAAACTAAAA

GATGTACCTAATAAGACAACTAATAACATTAAGAATGCTTTGTTTGTAGGTTCAACTGCTGAACTACAAAAACATTTAAA

AGATAAAAAGTTTGATGAGAATAATAGAGACACAGCAAACGATCCTTTTAAAGATACACCAATTGAAGGAAAAGATTAAG

TTATGTCTGACGCATATCTAGGTAACCCTAATCTAAAAAAAGTAAACACACCACAAGAGTTTACTTCGGACGAGATTAAA

GAGTTTAAGAAATGTGAAAACAATCCTATATACTTTATGAAGAAGTATGTACAAATTGTTTCACTTGACGAAGGACTAGT

GCCTTTTGATATGTATCCTTTCCAAGAAAAAATTGTAAATACAATACACGAAAATAGATTTACGATTTGTAAACTACCTA

GACAATCAGGTAAGTCTACAACAACTATATCATATCTATTACACTATGCGTTGTTTAATCCTAATTGTAATATNGCAATT

CTTGCNAACAAATCTTCTACTGCAAGAGATATATTAGGAAGACTACAACTTGCATATGAGAATTTACCAAAATGGTTACA

ACAAGGTGTGTTAAACTGGAACAAAGGTAATATAGAATTAGAGAATGGAAGTAAAGTAGTAGCGGCCGCAACATCTTCAA

GTGCTGTCCGAGGAGGTTCATATAACATTATCTTCCTTGACGAGTTTGCTTTCGTACCAACAACTATTGCTGAACAATTT

TTTAGTTCCGTTTATCCTACGATTACTTCTGGTAAGTCAACTAAAGTTATTATTGTTTCAACTCCTCACGGAATGAATCA

ATTTTATAAACTATGGGTTGACGCTGAAGCAGGACAAAACGATTACATACCTATTGAAGTATCTTGGAGAGAAGTACCAG

GTAGAGACGAAAAGTGGAGAGAAGAAACAATAAGAAACACTAGTGAGTCTCAATTTGCTAGTGAGTTTGAGTGTGAGTTT

TTAGGAAGTATTGATACACTTATCAGTCCTGCTAAAATCAAAGCGACACCGTATAAGACACCACTTAAAACAAATGGACG

ATTGAGTATCTTTGAAGAACCTATAAAAGGCCATACTTACTTATGTACGGTTGATGTTGCCAGAGGTACACTAAAAGATT

TCTCAGCGTTTATTATATTTGATGTAACCGATTTACCATATAAAGTTGTTGCAACATTTAGAGACAATGAAATTAAACCT

ATATTGTTTCCTGAAATGATTGCGAAAGTTTGTACTCAATATAACAAAGCACATATACTTGTAGAAGTCAATGATATTGG

CGCTCAGATTTCAGATGGTTTACATTATGAATTAGAATATGATAATATACTAATGACTACACAAAAAGGTAGAGCAGGAC

AAATACTTGGTGCAATGTTCAGTCAAAGAGGTTCACAATTAGGTGTACGAATGACTAAACAGATTAAGAAAATGGGTACT

GCTAACATCAAAGCGATTATAGAGTCTGATAAAATAATTATTAATGACTTTAATATTATTGGAGAAATGTCTACCTTTAC

AAGAAAAAATCAAAGTTGGCAAGCTGAAGAAGGCTGTAATGATGACTATATGACTTGTCTAACTATATTAGGTTGGGTTG

CAAATCAAAGGTATTTCAAAGAAATGACTGATAGAAATATCAGAGCAGAAATGTATAAAGAACAAGAGAAGTTAATAGAA

CAAGATATGGCGCCGTTTGGGTTTGTTGATAATGGTGTTGATACACCAGAAGAACAACCATTTTCAGATGAATATGGCCA

GGTATGGCATCCCGTGGTACGCAAAGGTAGTTAATGAAGATCCCCTATTTGATAAATATAAACGATTGAGAAATTTGAAT

ATGGGCGTATGAATAATACGAATTTTGACAAAGGAAAACATTATGTATTTTTATAAAAATACAAACAATAAAATAGAGGA

GAAAACCTAATGGCATTTCAAGTATCACCAGGTGTTCTCGTACAAGAAAAGGATTTAACTAACATAATTCCTGCTGTTTC

TACTAGTATTGGAGCTTTTGCTTTCAATTCTACAAGAGGTCCAGTTGGAGAGATAACGCTTATCTCTTCTGAACAAGAAT

TAGTTAGTATCTTTGGGAAACCTACTGCAAGCAACTTTGAAGAGTATTTTACTGCTTCATCTTTCCTTCAGTATTCCAAT

GCTCTGAAAGTTGTACGAACTGAAAACACTGGAATTTTAAACGCTGTAACCAATAGTGGTTCAGCAGTACTGGTCAAAAA

TACTGACCATTACAACTCAACATACTTAGCAGATGGTGCTTACACAGGTATTTCTGGTAGGGAGTTTGTTGCTAGAACAG

CAGGCGCTTATGGAAATGGATTATCTGTTTCTGTATGTCCTTCTGCAACTGCATATGAGCAAGAAGCGGTAACAACCGTT

AACGATAGTGCTGTATCAGTTGGCGACACAACTATAACAATGACAAGTGGAACTAACATTANTGTTGGTGACATACTTGC

ATTTTCAACAACAGCCGCNACTAACGATTATGATGACGGAATTGAATACGAGGTAACAGCCGTAAATTCTAACGACATTA

CAATTAAGAAAAGAGTTGGTGCTGGTGGTCTAACAAGAGTTGTCATAGACGGCGCTAATGTTAGAAGAAGATGGTCACAT

TACGATTCAGTAAATGGTGCACCTGGAACATCTCCAGATGTATTAGCTGCTGGTGGTAGTGATGATGAATTACACATTGT

TGTAACCGACGCTGATGGTTCTATATCAGGAACTAAAGGCGAAGTACTAGAAGTATACGAAAAAGTATCAAAAGCTAAAG

ACGCAAAAGATAGTGGTGGTTCTAATAATTTCTACCCAGAAGTTATTTACAAAAAATCATCTTTTATCTATTGGGGAGAC

CATAACGGAAACGGAACTAATTGGGGTTCAGTAAAGACAACTGCTTTCACTGCTGTTTCAGGACCTATTGCATTAACATT

CGGAAACGGTGTTGATGGAACGGTAACTGACGGTGCTAGAAAGTCTGCATTTGAATTATTCCAAGATAGTGAAACCGTTG

ATGTAGGATTGATTATGGCTGGTAACGCAAGTCTGAACTTGATTGGTGATTTAATTACAATCGCTGAAACAAGAAAAGAT

TGTATAGTATTTGCTAGTCCACAAAGAAGTGATGTAGTTAATATTGCTTCTGCTATAACTCAAACTAAAAATGTACTTGC

ATTTTTCAATGCTGTACAATCATCTAGTTATGTAATCTTTGATAGTGGTTACAAATATATGTATGACAGATATTCTGATG

TATATAGATATGTACCATTAAACGGAGATATGGCTGGTTTGTCAGCAAGAACTGATTTAACTAATGACGCTTGGTTTAGT

CCTGCTGGATTAAACAGAGGTATTATTAGAGGGGCAGTTAAACTTGCTTATAGTCCAAACAAAACTCAACGAGACGAACT

TTACAGAGCGAGAATCAACCCAGTTGTTTCTTTCCCTGGTCAAGGTATTATCTTGTTTGGTGATAAGACTGGACTAACAA

CACCATCTGCATTTGATAGAATAAATGTACGAAGATTGTTTATCGTTTTAGAGAAGGCGATTGCTACAGCTTCTAAATTC

CAACTCTTTGAATTCAATGATGAGTTTACAAGAGCTAACTTTAGAAACCTAGTAGAACCTTTTTTAAGAGAAGTACAAGG

TAGACGAGGTATCACAGACTTTTTAGTAGTATGTGATGAAACTAATAACACAGGCGAAGTAATTGATAGAAACGAATTTA

TTGCTGAGATTTACATTAAACCAGCAAGAAGTATCAACTTTATCACATTATCTTTTGTCGCAACAAGAACTGGCGTGGCT

TTTTCAGAAGTCGCAGGTTAGTAAAGAGGAGAAATAAAAAATGGCAAACATTAATGACTTCAAAACTAAACTTGCTGGCG

GCGGCGCTAGAGCAAACCAATTTAAGGTAACAATGCCTTTCCCTGGTTATGCACAAGTTGGTGGAGAAACAGAAGAATTG

GCGTTCTTATGTCAAACAACTACAATCCCGGCAATGAATATTGGAACTACTACGGTTAACTTCCGTGGAAGACCTGTATA

TTTGGCGGCTGATAGAAACTTTGAACCTTGGACGATTACGGTACTTAACGACACAAACTTTAAATTAAGAGACGCTTTTG

AAAGATGGCAAAATGGAATCAATAATATGTCTGATAACGAAGGATTAGTTAATCCAGTAGATTATCAAGTAGACGCTTTT

ATTGACCATTTAGACAGAAATGGCTCTACTATTAAATCATACACTTTAAGAAGTTGTTTTCCAACTTCAATCGGTGCTAT

TGATTTGAATATGGAACCAACAGAAGCAGTTGAAACATTTGAAGTATCGTTTAGATACCTTTTCTTTGAAGCAAGAACGA

CTACTTAATAGTTGAATAAATATATAATAAAAAGTAAACTTGTGAGGAAATTATAATGGCGGAACTTTTCGGTTTTCAAA

TTACTAGAGTTAAAGATACTCCAGACCCGAAGCAAAGTTTTACTCAACCTAAGGCAGATGACGGTACACAAACCGTCGCT

GCCGGAGGTTACTTTGGACAATACCTTGATATGGAAGGTAATGCCAAGACAGAGCAAGACTTAATAAGAAGGTATAGAGA

GATTTCAATCCATCCTGAATGTGATATGGCTGTTGAAGATATTGTCAACGAGGCTATAGTAGCAAACGAGATAGATAGAG

ACCCGGTGCGAGTAGATTTATCTGACACGGACTTTAGCGATAAAGTTAAGCGTAAGGTTGAAGATGAATTTAAAGAAATA

CTAAGGTTAATGAACTTTAGTACAAAAGGACACGACATATTCAGAAGATGGTATGTTGATGGAAGAATTTACTATCATAA

AGTTATTGATAGAGAATCACCTGTAAAAGGTATAACAGAATTAAGATATATTGATCCTCGTAAAATTAAAAAAATACGAG

AGATTAAAAAAGGTCGTCCAGTTGCTATGGCAAACATACAAGTGGTACACGACTACAACGAATATTTTTTATACAATGAA

AAAGGTGTTGCAGGACCTGGTATGGCAAGTGGTGGTATTAAGATTGCCACAGACGCTATCTCATTTTGTCCAAGTGGATT

AGTAGACTTGAACAAAAATATGGTTATGGGTTATATGCACAAGGCAATTAAACCTGTCAATCAATTAAGAATGATTGAAG

ACGCTGTTGTTATTTACAGAATTGCAAGAGCACCTGAAAGAAGAATATTTAAAATTGATGTAGGTAATTTACCTAAAGTA

AAAGCAGAACAATATCTCCGTGATGTTATGGCAAGATACAGAAACAAACTTGTCTATGACGCAAGTACAGGAGAGATTAG

AGACGATAGAAACTATATGTCTATGCTTGAAGACTTTTGGTTACCTAGTAGAGAAGGTGGAAGAGGAACTGATATTACTA

CATTACCTGGTGGACAAAACCTAGGTGAAGTATCTGATATTGAATACTTCCAGAAAAAATTATACAGAAGTTTAAATGTA

CCAGTAAGTAGATTAGAAGGAAGTCAAGGTTTCAATTTAGGTAGAACAACTGAAATTACTAGAGACGAACTTAAATTTAC

AAAGTTTGTACATAGATTAAGAAAGAAATTTACAGATTTATTTAATGACTTGCTTAGAACTCAATTAGTTTTAAAAGCAG

TTATAAATGAAGAAGATTGGCAATCAATAAGTGAAAAAATTAAATATGATTTTCTTGCTGATGGTCATTTCTCGGAACTA

AAAGACGCTGAACTATTAAGAGAAAGAATAGCATTAGCAAATGATGTGAGAGATTATGTTGGTAAATACTTTAGTGTAAA

ATTTGTACGACAAAGTATTTTAAAACAATCTGAAAGAGAAATTGAACAAATTGATAGTCAAATTAGAAAAGAAATTGATG

ATGGTCTTATCGCAGCTCCACAAACTAGTGTACCTGACGACAATGACATTATGTAATATAATAAGGAGAAAATAAAATGG

CAGACAATGAAATAAGTAAAGTAGATACTTTTGTAGACCAATTAGCAAAAGGCAACAACGCAGAAGCCGGTGAAGCTTTT

AAAGACGCTTTAAGAGATAAAGTCGGAGACGCATTAGATACAAGTAGAAAAGATTACGCTTCTTCTCTATTTCAAAGTGC

GGCTGATGTTATGACTGGACAGACAACAGATGTAGTTGATACTACAAATGCTGCTGAAGGTCATTCAGACGCAAAACCTG

ATGTTGCTACACCTATTGGACAAAGTGCTACACAAGACGAAGTACAACAAGCATTTAACCAGGCAACACCTGGTAACACA

GGAGAATAAAAATGGCATTAACGGTATCAAGTATTGTAGGACATACATCTGGTTACATCAAAAACGATAGATATAACGCT

CTATCGCCAGCGATGAAAACTAGTGTAGAAACTTTAGTAAGTGGCCTTGACGCAATTGATTGGAAGCAACCACAAGATTT

AGTTGACATTATTGAAACTAAAATCACAGAAGTTGCTGCCGGCGATAGCGATGTAGAGACTGCATTGACAACATATTTTT

CGGAGTAATTAAATGGCATTAAGTATTGTCTCAAAGGTAGACGATACCACTAAAGCTATTATTACGGCTAGTGGTGCGGA

CAACGAAAGTGGAACATTATATTCTGCTGGTCAATCTGTATCTTTAGCGAATGTATATTATGAGATTAGAGGAACAGGCA

CAGCGACTCTTAAACTCGGAGACCAATCTTTAAGTTTAACAGGGTTTGGTAATTGGGGATTAAAAGAAGGTGAAGCTCGT

AAAGTAATTGAACAAGAATTAAATACGGCAACAACTTTAGAGATTACGACAGACGCTAATGTTTCAAAATTTAATATGGC

TGTAGAAGTACAGAAAGAAACGGAGACAAAATAAAATGGCAGATTTGGTAACACAACAGATAGTATCGGATACAGCAGGT

GTTAAGTATGTTGTAAAACAAACTAACTATTCTGACGGCACAGGTGAAACAAACACCGTGATTGCTAATCCAACAACTTC

TAACTTTATGACAGCAGATGGTACTAAAGAGATTGCGAAAGTGTGGTATTCTATCAATACTGCAAACAGAAAATCAGCAG

TAGAGATTGCTTGGGGAGGCGCTACTGAAAATACAACATCATTATTATTATCAGGACAAGGGTATTTAGACTTTAGGGAT

GCTGGAAATGACATAGTAAACAATGCGACAAACCCTAATGGTTATGTCTATTTAAGTACTAAAGACTTTGCATTGAACGA

TAATTATACGATTGTTGTTGAATTTAGATAAGAATTATTATAAATATTAGGAAAGAGAGAGATAAACTATTATGAAATTA

ATTACAGAAACTTTGGAAAATGTAGAGTACATTACCGAAGAAGTTAAAGGTAAAACGAATTACAAAATTCGTGGTGTATT

TTTGCAATCTGAAATCAAAAACAGAAATGGCAGAGTCTATCCTAAGCAAACACTAGCTAATGAAGTTAGTAGATACAATA

GAGAATTTGTGGAACAGAAAAGAGCGTTTGGTGAGTTAGGACATCCTGATGGACCAACGGTTAACTTGGAAAGAGTAAGT

CATATGATTACTAAACTCTATCCAGACGGTAACAACTTTATCGGTGAAGCAAAGATAATGGACACTCCATACGGTAAGAT

TGTAAAAAATCTTATTGATGAAGGCGCTAAGTTAGGCGTTTCTTCTCGTGGTATGGGTTCATTAGAGAGAAGTCGTGGTG

GTGAAGCAAGAGTTGGAAACGACTTTTACCTTGCAACTGCAGCTGACATTGTGGCAGACCCATCTGCTCCTGACGCTTTC

GTAGAAGGCATTATGGAAGGAAAAGAATGGATTTGGGACAATGGTGTAATAAAAGAAAGAGATATTGAAGAGTATAAACA

ATACATTAAGGAAGCAAAAAGACTAAAACTTGCCGAAGCAAAAGCAGAGGTATTTAGTAAGTTCCTTAAAGGATTGTAAT

ATTATAAATATCTTATAACAAAACAAGAAAATAATTATTTTTTTTAAAAGAAATAAGGAGAACTTCAATATGGCCGAGAC

AGAAAAACAGGTTGTAGAAATGACGGCTCCAGACGCTCCTAAAAAGAACGCTGTAGCTGCTGAAACTTCACCACTATCTA

ATAGTGCTGAAGATTTAGGTGCTGCTGTAGTAAAACCTACAGATAGTAATCCAGACGCAACTAAAAAAGTTAAAGAAGTT

TCAGGTGACGCTCAACAGAAAAATTCTGGTAGTGCGGATCCAATGCCTTCTGTAAAGAAGGAAGAAACTGATTCTGAAGG

CGAGAAGATTTCCGAGGGAGAAATGCCTGACGGTCTGAAAAAATTCCTAGATAAAAAGAAAGATAAAGAGTCAAAAGAAG

AAGGCTATAAGATGAAAAAAGAATCTGAAGCTGAAACTAAAGACGCTAAATCTGCTGAAAAATCTGAGGAATCTACTGAC

CAGAAAGCTAAAGACATTGATGTAAAAGAACACATTGACGCTTTGACCTCTGGTGAATCAGACTTGTCCGAAGAATTTAA

AACAAAGGCTGCTACTATTTTTGAAGCTGCAATTACTAGTAAAGTAAAAGAAATTGCTGAAGAAATGGAAGTAGACTATA

ATAAGAAATTTGAAGAAGAAAGCTCTAAAGCTAAATCTGAACTAGTTGAGAAGGTAGACAATTATTTGAACTATGTTGTC

AACGAGTGGATGAAAGAAAACGAACTTGCTATTGAAAAAGGTATCAAGGGCGAAATTGCTGAGGACTTTATTTCTGGTCT

GAAAAAACTTTTTGAAGACCACTATATAGATGTACCTGATGAAAAATATGATGTGTTAGAAGACCAAGCTTCAAAAATCG

AATCGTTAGAAAATAAACTTAACGAACAGATTGCGAAAAATGTTGAATTGAATAGTAAGACTAACTTACTTGAAAAATCT

GACATTTTAGCTGATGTTGCTTCGGATTTAACAGATGTCTCTAAAGAGAAATTTGCTAAACTAACTGAAGCAGTTGAATT

TTCAAACGGTGAAGATTTTAGAAACAAGGTAACTACTATCAAAGAAAGTTATTTTGGTGCTAAAAAAGAAGCTAATTCTG

ACAGCGAAGTAGATAATGCGGTAGCTGATAATGGCGGTGTAGATTCTACACAAGATTTATCTAATGCAATGGCTGCTTAT

ACTACCGCTATTAGTAAAACAAAAGACTTGAAACTTTAATGTTCAAGTTAATAAAATAATAATAGGAGAGAGGAACAAGA

TATGTACTTATCTGAAAACTACCAAAAAAAGTGGCAGCCAGTATTAGAGCATCCTGATTTACCAAAAATCACGGATACTT

ATAAACGAGCTGTTACCAGTGTTATCCTTGAAAACCAAGAGAAAGCACTAAAAGAAGACGCTCAGTTTATGACTGAAACT

GCGCCTACTAACGCTACTGGTTCATCTATCGCTAACTGGGATCCAATCCTAATTAGTTTAGTTAGAAGAGCTATGCCGAA

CCTTATCGCTTACGATATTGCCGGCGTTCAACCAATGTCTGGTCCAACTGGACTTATCTTTGCAATGAGAAGCAGATTTA

AGGCTCAAAACGGAACTGAAGCATTATTTGACGAAGCTGAATCACAATTCTCAGCTGCGAAAACTACAGCAAATGTACCC

GGTTCTGCTGGAACATCATCTGCTGGAGAAACAAATCCTGCTGTACTTAACGACTCGTCACCTGGAGCATATACTGCTGA

AGGTGGAATGTCTACTGCTACGGCAGAAGCATTAGGAGACTCTGCGAACAATAGTTTTGCTGAAATGGCATTCTCAATTG

AGAAGTCAACGGTAACTGCTAAGTCAAGAGCTCTAAAAGCTGAGTACACAATGGAACTTGCACAAGACCTTAAAGCAATT

CACGGTTTAGACGCTGAGTCTGAATTAGCGAATATTCTTTCTGCTGAAATCCTTGCTGAAATTAATAGAGAAGTTGTAAG

AACTATCTATGTTAATTCAGAAAAAGGTGCTCAAACTGACACAACTGCTGCTGGTATCTTTGATTTAGATACTGACTCAA

ACGGTAGATGGTCTGTTGAAAGATTTAAAGGACTTATGTTCCAACTAGAAAGAGACGCTAACGCTATCGCACAAAGAACA

AGAAGAGGAAAAGGTAATGTTATTATCTGTTCTTCTGATGTTGCTAGTGCATTACAAATGGCTGGTGTTTTAGACTATAC

ACCTGCATTGAACAACAATTTATCTGTTGACGATACAGGAAACACTTTTGCTGGAGTATTAAACGGCAGATTTAAAGTGT

ACATTGATCCATATAGTGCAAACCAAGCAAGCAAACAATTTTATGTTGTTGGTTATAAAGGTACTTCACCTTATGACGCT

GGTATGTTCTACTGCCCTTATGTGCCATTACAAATGGTTAGAGCAGTTGGTCAGGACACTTTCCAACCGAAAATCGGTTT

CAAAACTAGATACGGCTTACAAGCAAATCCTTTTGCTGAAGCAGGTTCTGGCGATGCAGCTGTTGTTAACGGTTCAGGTT

CTGCAAACGCTAACAGATACTACAGAAAAGTACAAATCACTAACCTTGCGTAATTGCAAATAGTGATTGTATAGTAGACA

ATCTTACGAAAAAAGGCGATGTTAAAGTCGCCTTTTTTTTAGCCCAAATTATAATATCAGTATATTCAGATTGCTAGGGG

TTTATTAGGTCTTATAAATAGTATTATGACAAATACAAACGCAATAACACGACAACCAACACAACTTGATTATGCGTCAC

CAACGCAATTTAAGTTTAATATCATAAAGCTTCCAAAGGTAGAATACTTTTGTACAGAAGTTAATATCCCAAGCCTACAA

ATGACCAATGCAACACAGGTAACTCCATTGAGAGATATACCTTTGCCTGGAACTAAACTTGACTTTGGAGATTTAGTACT

TACATTTATGATAGACGAAAAGTTAGAGAACTATGAAGAGATATTTGGTTGGTTAAGAGGTCTAGGTTTTCCTGAAGACC

ATAGCGATTATGCTAATTTGTTATCTTCTGGAAGAGATAGATTTCCTACACAAGGCAAAGATAGTCAAAATCTAAATGCT

GGTAGAGAAGGAACTGCTGCTCCTCAAGGAGGTATTTTGTCTGACGCCACACTAACAATATTAAGTGCAAAGAATAATCC

GATTAAAGAAGTAAGATTTAAAGATATATTTCCTGTTTCGTTAACCGGCGTAAATTATAGTCAACAAGCAAGTGATGTTC

AATACTTAACTAGTAGTGTTAGCTTTAAATATACGACTTATTCTTTTGCTGAACCCGGCAAACCTTCTACGCTTTATACC

TCATAGAAGCTTGACAAATAACTAGATTTAGTATATAATTATACTTAAAACTAGAGGAAGATACTAGATTATGACATTAG

AAGAATTACAAGAACTTTCAGATAAGAAATTAAAAATCAATGATACTGAGCTTGATATTGAAGCTCTTAAAACACCACAA

CTACATAATGAGTTTTTGAAACATTACAATAAGTTTAATCTTTTACTTACTAAAACTGAAAGTGAGTTAAGGATTATAAA

ATTACATAAATGGGAATATTACACAGGGAAAGCGGACCCAGCAGTTTATCAAACTAAACCATTCAATTTAAAAATTCTAA

AGCAAGATGTTGATAAGTACATTGAAGCAGATGAAGATTACATAAAGATAAAACAAAAAGTAGATTACTTAAAAACTATA

TGTGATTATCTGGATAAAACAATCAAACAAATATCAAATCGTGGATTCCTAATCAAGGATGCTATTGAATGGCGTAAGTT

TACTTCTGGCGCTATTTAATAGATGGTAGAAAATCGTTATTTAATATTAGATAAGAAAGACGAAGTATATCTTTCAATAG

AAGCCGAGAGCGATATTCGTAGAGAACTATCGGAGTTTTTTACATTTGAAGTTCCTGGTTATAAGTTTATGCCTCAATAT

AGAAATAGATATTGGGACGGAAAGATAAGACTTTTTAAGTATGCAAGTGGTGAGATTTACTATGGTCTCTTACCATATAT

CAGAAAGTTTTGTGAAGATAACAATATAAAAATTGTATCTAACATAAAAGAGAAAGCAAAACCATTAGATAAGTTAGAGT

GTGCTAGATTTTGTAAAGCATTAAAGATACCTAAAATTACTATTAGAGATTATCAATTCAATGCTTTCTATCACGCAATA

CAAGAAGATAGATGTTTATTACTATCTCCAACTGCTAGTGGTAAATCACTTATTGCATATCTTATATTGAGATTTCAACT

ATTAAGAATTAAAGAAAAGAAAGCAAACAAAGTATTAATCATTGTACCTACAACATCACTAGTAGAACAATTATATAAAG

ACTTTGCTGACTATGGTTATAATACTAAACACATACATAGAATATATCAAGGACACGACAAAGACACAACAAAGAAAGTA

GTAATATCTACTTGGCAATCAATATATAAACTACCTAAAAAGTGGTTTGCACAATTTGGTTGTATACTTGGTGACGAAGC

ACATTTATTTAAATCCCAGTCCCTTACAAGTATAATGACAAAGATGACTAATTGTAAATATAGAATAGGTATGACTGGTA

CACTAGATGGTTCAAAGACACATAAACTAGTGCTAGAGGGTCTATTCGGGGCTGTAAATAGAGTTGCGAGTACAACTGAC

TTGATAGAGAAGAAACAACTAGCAGACTTTAAAATACATTGTTTAATACTTAAACACGGAAAGAATAGTAAAGACTTTTT

AAAAGATAAGAACTACCAAGAAGAAATGGATTTCTTATGTGCTAGTAAAGCAAGAAACAAATACATTACGAACTTGACAA

ATGGTCTTCAAGGTAATACACTATTGTTATTTCAATTTGTAGAAAAACACGGTAAGGTATTACAAGAACTAATTGAAAAG

AAAGTAGATGATGGTCGTAAAGTATTTTTTGTATATGGAGGAGTATCAGCAGATGATAGAGAAAACATTAGAGCAATTAC

AGAAAAGAGTGATAACGCTATTATTGTGGCTTCGTACGGTACCTTTTCAACGGGTATCAATATACGAAACTTACATAATA

TTATTTTTTCTAGTCCTAGTAAGTCTAGGATAAGAAACTTACAATCTATTGGTCGTGGTTTAAGACTAGGTGATAACAAA

GTTAATGCGACACTATATGATATATCAGATGATGTTTCATACGGAGAAAAAGAGAATTATACATTACAACACTTTAGAGA

AAGAATAAATATATACAACGAAGAAAACTTTGATTACGAGATACATAATGTGGAGTTAAAGGAGTAGTATGGACAAAAAC

AACGAAACTAAACCAGAAATTAAAATCGTAAAGATTATGAATGGCACAGATATTGTTTGCCATATACCTCAGGTAGCAAA

TCAACAACGAAATCCGTTGCTGACATTAGACAAACCATTAGAAATAAAATATGTACCACAGATTACTAATCTTGGTATCA

AAGACTACATAGCGCTTGTGAAGTGGGCAGCTTATACAAATGACCAATTGGTTACTATTCCTAAAGATAAGATACTGACT

ATTACAAATGCAAGTGCTGAAATGATTAAATCATATACACAAGTTATTAGCGACTACACAAACCACGACAAGGTTATGAG

ACGAGAAGACAATCCGAGAACTGAAAGACTTATGGATAGAGAAGTAATGCGAGAAGAAGAATTGGAAGAGTATGATGAAA

TATTTGAGGCCTTTAATGACATTAAAAAGAAAAGTACAATTCACTAGACGCTACTCTATAGACTCTATCTCTACCCAGCG

ACACGCTGATAATAACATAGGATCCAATATATGTCAAGCGCCTGTGCCAATAAAATTAAAAGTGTTAGGTGCTTGACTTA

TTTAACAACATATAGTATAGTGAGAACATAATGAAAACTAAAACAGATAAAAAAATTGAAACGGCAGAAGTGCCTGAAAA

AAAGAAACGCATACGAACACCGGCGAAGAAGGAACACTATGTTAATAACAAAGAGTTTCTAGCTGCAATGGTAGAGTATA

AAGACAAATGTAATAAAGCAGAAGCAAGAGGCAGAAAGAAACCTCCGGTTACTAATTACATTGGAGAATGTTTTTTAAAG

ATTGCAAACCACTTATCATATAGACCTAACTTTATCAATTATACTTTTAGAGACGATATGATTTCAGATGGTATTGAGAA

TTGTTTACAATATCTAGGTAACTTTAATCCAGAGAAGTCAAACAATCCATTTGCATACTTTACACAAATTATCTATTATG

CCTTTGTAAGACGAATACAGAAAGAAAAGAAACAAACAACAATCAAACATAAGCTTATTATGGATGCAAACTATGACGAT

ATGACTTTGCAACCAGGTGATGATAGAGACTTTAAAAATCAATTTACAGAATTCTTACAAAAGAACTTACCAAGCCAAGA

ACCAACAAGTGAACCTATAGAAACAAAACCTAAAGGTGCAAAACTAAAGAGAACCCGAAAAGCGAAGATAAATTTAGAGA

ACTTTTAATTATGAAGATAGCATTGTTGAATGACACTCACTTTGGTGCAAGAAGTGATAGTCCTGCATTTATTAAATATT

TTAACCGGTTTTATGATGAGATATTTTTTCCATACTTGGAAGAGAATAACATTACAACCTTAATACATTTAGGCGATGTA

GTAGACAGAAGAAAATTTATTAACTTTAATACTGCTCATAACTTTCAAAATAAGTTTTGGAAGAGACTATGGGATATGAA

GATTGATACACATATTATACTAGGTAACCACGACACATACTATAAGAACACAAACTCTATTAATAGTATGCAACAACTAA

TTACAACCTTTGATGGTGTAAACGAACCATTTATATATGAGAAACCAAAGACGGTTGAGTTTGATGGTTTGCCTATTCTA

TTCATACCTTGGATATGTCCAGAGAATGAAGAAGAAAGTCTAAAAGCAATATCAGAAAGTCAAGCACAAATATGTATGGG

TCACCTTGAAGTTAAAGGTTTTGAAATGCACAAAGGACACTTCCAAGAACACGGTTTAGAAATGGACTTGTTTAAAAGAT

TTGAGAAAGTATATTCTGGTCACTATCATAGAAAATCAGATAATGGTACTATCTTTTATCTAGGTACACAATACGAGATT

ACTTGGTCAGATTATCAATGTCCTAAAGGTTTTCATATCTTTGATACAGATACAAGAGAACTAACAAGAATTCCTAATCC

TATCAATATGTTTAAGAAGATAGTATACAATGATAAAAAAAATTCATATAGTAATATGGATATAAGTGAATATGAAGATT

GTTTTATCAAAGTTATTGTAGAAGAAAAAACAGATGTCAACCAGTTTGGTGACTTTATTGATAGACTACATAATGATATA

CACACAAACGAAGTAAATGTTATTGAAGATAGTTATAATATCAATTCAACTGCTGATGTTAATATAGTAGACCAAGGAGA

AGATACACTATCTTTCTTACAAAATTATATNAATAGTTTAGATACTGAATTAGATAAAAATAAGATGAATAGTATAGTGA

AAGACTTATATAGTGAGGTGCAAGATAAGTGATAATATTTCATAACATAACCTGGAAGAACTTTCTTTCTACAGGTAATA

CACCAATCAGCGTAAACTTAAATGAATCACCTACGACATTAATCATAGGTACTAATGGTTCAGGTAAATCAACTTTACTT

GACGCTTTATGCTTTGCATTGTTTAACAAACCTTTTAGAATTATTAAAAAAGACCAGATGGTTAATACAATCAACAATGC

TGATACCGTTGTTGAAGTTTANTTTAGTATTGGTCCAAAGAAATACAAAATACGAAGAGGTATTAAACCTAACATATTTG

AAATATACCAAGACGGCATTTTATTAAATCAGGATGCTTCTTCTATAGATTATCAAAAGTACCTTGAACAGAATATAATG

AAACTTAATTACAGGTCATTTTGTCAAGTTGTAATTTTAGGTTCTTCTTCTTATGAACCATTTATGAAGATGAGAGCAAG

TTATCGTAGGGATGTAATTGAAGAGATACTAGACATTAAAGTATTTGCAAGTATGAACTTATTGTTAAGAAGTAAACAAC

AAGACTTGACCAAAGACATTACCACAATGAGACATCAGGTAGATTTAATTGAAAACAAAGTTAATCTACAAGAGAAACAT

TACGAAGAATTACAAGGTAGAGATACAGACGCTATCACTAGAAAAAAAGAAGACATAGAGAAAGCACAACAAGGTAAAAG

AGATTATATGGTTCGTATCAATAGTCTCAATAACGAAATTGAAACAAACAAATTAAAACTACAAAACAAAGAAACGACTA

AAAGTAAGTTTCTTCAATTACAGAAACTAGAAAGTAAGATTGATACTAATTTAAAGACACACAAAAGAACATTAAAATTC

TTTGAAGAGAATACTAATTGTCCTGTTTGTACACAAGAACTTGAACCTAGTTTCAAACAAGAGAAAATCAATGAAGAGAA

GGCTGCTGTAGATAAACTTAATGAAGGTTATAAACAACTATTAACTGAAATTACTAATACAGAAGAGAAGATACTAAACC

TTGATAAAGTATCAGAAACTATTAGAACTATAGAAACAAATGTTTCAAAACTTAATCATAGTGTTGATGAGATTAAAAGA

CATAGTGATAGAATACAAGATGAGATTGAACT

>lcl|3300005430_____Ga0066849_10000198|139039_ _

AAATAGGGGAGAAAACAAGTGTCTCAAATAAAAGCTAGAATAGATTCAACAATTAGTAGACCTCAACAAGTATCGGTTAC

TATGCCTGCTGGAGCAGCGTCTCAAACTGCTGTTACCAACTCAACATTAAAATTAAGACTTCTATCCGATGTAGACGCAA

GTACCCTGGCTGATGGTTCAATGATTCAGTATTCTTCAACAAGTGATAAGTTTGTTGTAAGAGACGAAATCTCAACTACA

ACAGGTTCTATCACTTTAAATGGTGGTTCATTTTAAATAGAGAGAAAAGAATATGGCAACAATAATACGAATAAAAAGAA

GTGCTAACACAACTGCTCCTAGTACATTAAAACTTGGAGAATTAGCTTTAACTTATGGAACTGGTACTGCAGGAAATAAT

GGAGATAGATTATTTGCTGGAACAGGTGGGGTTGATGGAAATGGTGACGCAAACGATATTGATGTAATTGGTGGTAAGTA

TTTTACAAGTTTATTTCCTACTACAAACGGCGTAGTTGGTTCAGAAAAATTAATAACAACAGATTCAAATAACAGAATTG

ACCAAATGGTGTTTGGTAACTCAAATACTGATTCAGGTCAAATCACATTTAACGAAGCATTAAACAACGGTTCAAACAAT

GTTGTTTTAAAAGCTCCGTTATCATTAACAAACTCATCTACACTTTTATTACCTGATGGTGCTGGAAGTGCTGGACAATT

TTTAAAAGTAGATTCAGCAAGTGGTTCGGAAGCTCAATTAGGTTTCGCTGATGTTGATACTACTCTTACTTTAGAAGATA

GTACAGGAACATCAATTAACTATTCAACTGCAAACACTTTATTGTTAACAGGTGATGGTACAATTGATACTGCCGCTACT

GCTAACACAATTACAATTAAAGTACAAGATGGTTCAATTGGAACAACTCAACTTACAGACAACAATGTAACCAATGCTAA

ATTAGCAAACACTAGTGTTACCGTTGGAAGTACTGCTGTTGAATTAGGTGGTTCTGCATTAACAGACCTTGCTGGATTAA

CAAGTGCTATTGTTGATGACTTATCTTTAAACGGACAAGACATTTCAACAACAGGAAGTAATAAAGATATTACTTTAACT

CCACACGGAACTGGTGTTGTAACCGTACCTAGTGGATATAAAGATAGAGCAGGTTTTGGTGCTGACGCATTGGCAACTAA

AGAGTATGTTGATGGATTTACTTCAGGTTTAGATGTTAAAGATAGTTGTAGAGTTGCTACTACTGCTAACTTAACGGTTA

CTTATGACCAAACAAATAAAAGATTAGATAACGCTGACACACAAGCTGCTTTGGCAATTGATGGTGTTACCTTATCAGTT

AACGATAGAGTATTAGTTAAATCTCAAACAGAAGCAAGACAAAATGGAATTTACAAGGTAACAGATGTTGGTTCTAACTC

TTCTAATTGGAGATTAGAAAGAAGTTCAGATACGAATACAGGTGCTCAACTAACAGGTGGTTCATTTACTTTCGTTGAAG

AAGGAACTGCTAATGCTGATAATGGTTATACATTTACTCACAATGGTATACCTACATTAACAGACAATACTTTATCTAAC

AATACAGAATTACCAGTATCACAATTCTCGGGTGCTGGACAAGTAGTAGCTGGTGCTGCTCTTGTAAAAGCTGGGAATAC

TTTAGATGTTAATGTTGATAACGCTTCAATCGCTGTTGTATCAGACGCTTTACAAGTTAAAGCTGGTGGTATTACAAATG

CTATGTTGGCAGGAAGTATTACAAATGCTAATCTTGCTGATCCAAATGTTTCACTTGCAGGTGAAACTGGAACTGGTACG

GTTGGTCTTGGTGGTACATTAACTTTTACTGCTGGCGAAGGAATTGATACTTCTGCTTCTGGTTCTGCAATTACAATTGC

TGGGGAAAACGCTTCAACAACAAATAAAGGAGTTGCTTCTTTTAGTACTGATAACTTTACGGTAACAAGTGGTGATGTTG

CGGTTACTGAAATAGATGGTGGACCATTCTAATGGCTACCGTAATTAAACCTAAAAGAAGTTTTACGGCGACTGCTGTAC

CAACGGTTTCTGATTTAGAAATTGGTGAGTTGGCAATGAATGTTGCTGATGGTAAGTTTTTTACAAAATTAAATGCTAGT

ACTATTAAAGAAGTTGGTGGTGCAAGTGCTGTTAATATTCAATCAGTATTAAGTGCTGGTGCAATTTCAACAACAGATTT

AGTTTTTAATAATGCAAATATTATATTTGAAGGTGCTACACCTGACGCCTACGAAACAACATTAACGGTAGAAGACCCGA

CTGCTGATAGAACGGTTAAACTACCTAATTCAAGTGGAACTTTGGCGTTAACAGGTGATATTCTTGCTTTCGCTGTAGTA

TTTGGGGGATAATAAACAATGGCAAGTGCTTTTAAAAATGCTGGACAAGAAAATCCAACAACTGACGGTGTAGGTGCTAA

TGTATATACGGCTCCTTCTAATGGAACTGCTGTATTACACGCTGTTTATATCTCAAACAAGAATCCAGCTACTCAAGCAG

TAGTAGATGTATCGGTTACGATAGATGGTGGAACAACTTTTAGAAGTATTATAAAAGGTTGTATAATTCCACAGAATAAC

ACTTTTATATTAGACAAACCAGTCAATTTAGAAGCAAACGATATAATAAGAGTAGTGAGTAATGTGGCGAGTACGGATAC

TTTTATATCTGTACTAGAGAATACATAATAGTTAAAAGATAGATTATAAATAGTATAAATATAAACAAAGAGGAATTTTA

AATGGGATTAGCATTAGCAACAGGAGCAAGTCAAGCCGTTGGGGTAGACGCTGCTGGATTTCAGATTTCAAATGAATACG

CTATGCACGCCCTTAACCGTGATGTAAACGGACTATTGATTTATACAAAAACATCAATGGATAGTACGGATACTATTGAG

GTTAACGATGGACAAGGATTTGGTTATAATGGTTTTGAAGGACTTGCATTAGGAAAAGCAAGTGATGGTACAACCGTTCA

GAATACTTTACAAAGTACATATGACGAAAGTACAGACGACCACTATCAAACAAATGCTAAGTATAGAAAATATCAACAGG

TCAGATTTGACCCTTTGAAACTTTTTTATTTTATTAATGATGAAGGAATGTTAGTTGCGAGATACCAACACGACTATACA

TATGCTGCTACAGAAACGGCAACTTCAACTACTGGTAATAACTGGATACCTTCTGGTGGCGTTTATTATACACAAGCCAA

TCAGGAAAGGTATTTGTAAAACAAAAATTAGAGAGAGATAAAAATGGCAGATTTTATTTTAGGTAGACTTAAATTTCACT

TCAAAGGTGATTGGGTTACCTCAACAGCATATATTAAAGATGATGTTGTAAGATATGGTGGAAATAGTTTCGTTGCTATG

GCGAACCATACATCTTCAGCACTTTTTGAAACCGACCTTACATCAACAAAATGGAAAAAAATGGCAGCTGGGCAAGACTG

GAAAGGTGCTTGGGCAGCTACTACATTTTATAAAGTGGACGATGTTGTACAATGGGGAGGTTCTACCTTCGCTTGTAATG

AAGCACACACTTCACAAGCAGATTTATATGACGACACATCAAAATGGACATCTTTTGTTCCAGGATTTAAATGGACAGGT

ACTTACACAAATGGTACTGCATACAAAGTTAACGATTTAGCAAAATATGGTGCAAATGTTTATATTTGTTCCGTAGAACA

TACTGCCGCTTCTACAATAGATACTGCTAAATTTTCTATGTTCGTATCAGGACTAGAATTTGAAGATTCATACAACGCTG

GAACAGCTTACCAAGCTGGAGACATTGTATCTTATGGTGGATATAATTATGTTGCTAAAGTACAAAGTACTGGCGAAACA

CCATATAACAATGCTACTAAATGGGAAGTATTAACAACTGGTTATAAAATGGTTGGTACCTATGCAGGTGCAACCGCTTA

TAAAACTGGTGATGTAGTAATGTATGGTGGTCATACTTATGTTGCAAAAACAGACGCAACAGGAATTGTACCAACAACAA

CTGCAACTTGGGATTTATTAAACGAAGGTTTAAAATGGAATGATAGCTGGACTGACGGAGCTGAATATGCTCCAGGTGAC

GCTGTTGCTTATGGTTCATCTTCTTATAGATGTAAACTTGCTCATACTGCTACTGCTGTAGCTGGTGACGCTAAAAGACC

AGATTACGATACAGGTGGAGTTTATTGGGACTTACTTGCTGAAGGAGATTCAAACTTTGTTACCACTACAAGAGGTGACT

TATTAACTAGAAACGCAACACAGAATGTTAGATTAGGAATTGGAACAACTGGTTCAATGCTTAAATCTGATGGTACAGAT

GTTGCTTGGGCACTTGCTCAAACTAACGACAATGTATATTTTGTTGCACCTCACGGAACAGACGCTTTACCAAGTACAGA

CGCTGGTAGAGGAACTTCTTTAGATAAACCTTGGGCGTCAATTAAATACGCTTGTGATTGGTTAAAAGACACAGGTAACT

CTGGATTAGATTACACGGTTACGGTTACAGATTCAACTAACTTTACAATTACTTTAGGCACATCAACTTATGCTCACACA

TATTCAACTGGTGGTAAAGTACACAAATCAGATTTTAGTACGGTTAACATAACAAACGCTCCATATAACAATGGAACAGG

TGTGGTAACTATTACAACTTCAGGTGCTCACGGATTATCAACTAGTGATAAAGTAAAACTATCAGGAATAGTTTATACTT

GTTCTGCTGGAACAAAAACTTATCCAAAAGATACGGTTAACAAAACTCTTTATGTTAAAACTGGAGAGTATACAGAAGCA

TTACCAATTGTTGTTCCTGCGAACACACAAATGATTGGTGACGGAGTAAGAAGTACAAGAATTAAACCTGCTTCAGGTAA

TTCAGTAGTTGCTGGATTAACTAACACACCTAACGCAAGAGCAGATATGCTTAGAGTTAGAAATGGTGTTACCATTACAG

GATTTACTTTTTCTGGAATGACTGGAACAATGGGTGCTGCTGACGCAAATGGCGTAGCAAGACCAAATACTGCTGACGGT

GCAACTCGTTCAGGAGTAGTTATTGCTTTAGACCCAGGAACTGGAACTACAGATACTGCTGCTTGGATAGAAACTAAATC

ACCATTTATACAAAATTGTACACACTTTGGAACAGGTTCAGTTGGTATTAAGATTGATGGTTCACTACACGGTGGTGGAT

ATTCTTCAATTCTTGCAAACGACTTTACACAAATTTCAAGTGATGGTGTTGGTTGCTGGGCATTATCAAATGCTAAATCA

GAATTAGTATCAGTATTCACTTATTACTGCCATCACGGTTATTTGGCTGATAGTGGAGCTGTTATCAGAAGTTTGAACTC

TAACAACTCTTATGGAGAATATGGTTCAACTGCTGCTGGTATTGACGCAAACGAAACACCTTATACAGGTTCAGTAGATT

TAAGAAACAATGAAGCTAAAGTAGGAAGAACTTTAGTTGCTGGTTCTGGAATCGGAAGATTAGAAATGGAATATGCTGGA

GAAGGTTATTCTTCTGCTACAATTGCAATCGGAGGTTCTGGTGCTTCAGGTGCTGTAACCGCTGGATTTAGTGATGGTGC

TGTAAAACACATCACGGTTGCAACAACAGGTTCTACACACTTTACAACTTCTGCTTTCGCACAATCAGGAACTTCAACTA

CATTAAAACTTGCTGCTTCTGATTCACAACCAGACGATTTCTATAATGGAATGAGAATAAATGTTTACACAGGAACTGGT

TATGGTAATACTGGTATTATCGCTGACTATGTTGCTTCTACAAAAACTTGTTCAGTACAAAAAGAAAATGGTACTGCAGG

TTGGGATGTATGGGTAAACTCTGGTTTATCTTCTGCTACATCTTTTGATACAACATCTGGTTACGAAATAGAACCAAGAG

TAGTTTTATCTGGTGGTGGTTCTCCAACAAGAGACGCATTAGCAAGAGCTGTTGTAGATAATCAACAAATTTCAAAAATT

TATATATTAGACGGTGGTGCTGGATATACTTCTGCACCTACGATAACAATTACAGACCCTAACGCTTCAACGGTTGGTAC

TGCTACTTCTGAAATAGGAGATGGAGTAATATCACAAACAACGGTTGGAACTGCTGGTTCTGGATACAAAACAGAAACAA

CAACTGCTACCGTATCTGGTAATGGTTATGCTGAAATATCAAGTGAAGGTACTGCCTTTGTTAGATTAACAGGATTATCA

AAATCACCTACAGGTGGAGACATTGTAGAGTTTGCTGGAATAACTGGACAAGCTTACTATGTTGTGGCGGTTACAGGATA

TTCTGCTGGTGCTGGATTAGTTAGAGTAAATCCAAAATTTACAACTGCTAATAAACCAACTCACGGAGAGACTGCAACTT

TAAGAAGTAATTACTCAAACATAAGATTAACAGGACACGACTTCTTGGATGTTGGTACTGGAGATATTACAACAACTAAT

TATCCAAATGTCGCTACACAGGCGCCAGACGCTAATGATGAAATCTTTGAAGCTGATAGAGGAAGAGTATTCTATTCTTC

AACAGACCAAGATGGTAACTTTAGAGTAGGTAATTTATTTAAAATTGAACAGGCAACTGGTAAGGCAACATTGAACGCTG

AAGCATTTGACCTTTCTGGACTACAAGAATTAAGTCTAGGTTCAAGTGCTCAAGGAAACTTTGGTGCTACAATTGCTGAA

TTTAGTACCGATGGTACTTTGGCAGACAATTCTGATACTGCTTTAGTAACCGAAAGAGCAATTAAGACATATGTAGATGG

ACAACTTGGTGGTGGACAAAATGACTTGTCAGTTAACTCATTAACGGCTGGTTCAATTACTGCTACAGGACAAACAATAT

CTACAACAGGATTAAGTGGAACAGATGTAAACTTAACTATTGGTACACAAAACGATGGTATTATTACTCTTTCTGCTCAA

GCACAAACAAGTATTACACCAACAAGTGGCAACGACCTTGTTAATAAGTCATATGTTGACGCACAAGGAACACCAACATT

GCAAACACTTTCTATTGATGATGTAGATTTATCATTAAAAAGAAGAGTCATTACAAATGCTAATGAACTTATACAAAAAG

AAAGTACTCAATTTGATGGTACGGATGCAACTGAAGGATTTGAATTTATTAACGGCACAATGCAAATTAATATTGACAAA

GCTGGAGATTTGGTAATAGAAACAATATAAATATAGTTAAATTAGGAAGATAAACAATGGCAACAACAAAACAAAGAATT

GGAAATCTGTTCTTCAATTATCAAGGAGAATATTCAGCTACAAAGACTTATTACCACGATGATGTTGTACAATACAACAA

CACGGATTGGATTTGTACAAAGAATTCTGCTACAACAGGAACTGCTCCAGTAGACAATGAAAGAAGATATGTAAGAATTA

CTAAAGCTGTTTCAGCGAGTACAGGTTCTAACGCATACAAATGGGATGGCGAAGCTACTTGGCCGCAAACAGAATTACAA

TATAAAATTGGTGATACTTTGGTATTATACCAAGATGGAAACGATTTTGATGATAACAAAATTGCTTTTTCAGATTCATC

TACAAACAAAAATACAAATTTATTCCACAAAGATGTTACCTATTTCTTAAATGGCAAAGCTGTTGGTGCTGGTACGGCAA

CTAATGAATACTTTAATTCATCAGCTTTTAACAATGCTACTAAAAGAGAAATTAGAATAGAATTTACAAGTGAAACACCT

AAAGAAATTTATATGTTTAACTACGATAATCCAAGTGCCAATTGGGGTCCTAAAATAGTTGTTGCTGACCATTCAATTTG

GAAACCGATTAGACAATCATTTAAATGGAGAGGTGAACACGATAACACAAACGATAGTGGTTCATATCAAACTTATTACG

CAAATGACATAGTAAGAATAGTTGTTCCTTTAGATAACGACTTTTCTGAAACGGTTATGGACCAAGAACATATCCAATCT

ACAAGAGCAACTTATATTTGTATAAGACAACATACTTGTGATGGTACTGACAAATATTTACCTTGGGACCAAGAAGCTGA

TACAGACGACAATAAGTATTGGGAAAGAATTTCTGAAGAAATGCAATTTGACGATGAAATCGTTGAAGATAGTGGAGCTG

TTGCTACAATTACAAATGTATCTGCTGCTTCTCCAAGTAGAAAACAAGGATTTTACAGATATGTAAATTGTAATAACATA

ACTGCCGCTGCTGAAAATAGTGGCGCAGGAAGAACTGGTGGATATAATACACCAATGTTTGATGTTGAAGTAGAAGGTTA

TCAATCTGCAAAGACTTGGACGATTGAAACAAGTGCTCATTACAAAAGAAAAAAAGGAAAATACAATAATGTAACCCACA

CTTCTTCAAGTGGTGGTGGTTCAAACTCCTATTGGGATTTTGAAGTTGACCAAGAAGGAAAAGTAATTAAAGCTGTTCTT

ACGAAGAAAAATCTTGGTGGTGATATGGGTGGATTAGGATATGCTGTTAACGAAGAATTAACTTTTGCTGACGCAAGTTT

CGGTGGCGGTGGTGCTGCTGATGTAGTCTTAAAGATAACTGCAATAGGAACTTGGGGTGCATTAAACATAAATTTATGTA

AAGACGATAGACAAGGAAGACAACACGCACAATGGTATAACGACAACGAGGCACCAGTATTTGGTGGAGAAAATAACGCT

ACTAATGACCAATTAGGATTTGATGGACAAATATTTTATTCTTCTGCCAATGTAACCTTTGATGTTGCAAGTACAAGTAA

AAAAGCTAGAGGTTATGCTACAAGATTTTCAGGAAATAGATTAGAGTGTATGAGTTTATGTAATACAAATGGTCCAATTG

GAGACGATAACAAGTATTACAGATTACCTGGACAATTCCAACAAGCTAACTGCGTTAACTGGCCGTGTTTTATTAATGGT

CGTGGTGGTATTACAAGTTGGGGTTCAAACTCAACTGGACAAAACGGAATTAATCAAGGTTCTGTACTAATGGGAGTTGG

AATGACTTTCCCATTTACAGATTGGTATAGAAGTACAGATAACGGTGGTAGTGGTATTCATACTACTCCTGATGGAGAAC

CACCTAAAGCTATTCAATTAATATCTGGATATGAAACTGGTATGGCATTATTTAACAATGGCGAAATATACCATTGGGGT

TATGGAGGTCACGGACAAAGTGGTGACGCTGCTACTTCAACTAGAAGTTATCCAGTAAGATGTGGTGGAACATATCAGGA

AGTTTATGCTGCTGCTAATGCTTCAACACACACTTTAATGTCTACAAGAATTAAAAGAATATGGATGACTAATTGGGGTG

GAGACAATAATACAAGTACTCATAGTTGCTATGCGTTAGATACTGATGGCGAATTATGGGCGTGGGGTTATAATGGATAC

GGACAATTAGGAGATAATACTACAACAGAAAGAAATAGACCACAAAAAATTAACAAGACTTCTTACTTTAATGGTAATAA

GATTGTTGCTTTCTGGACTGCTGGAGCAGGATACGCTTTCTGTTTCGCTTTAGATGACGCTGGAAAATTATATAGTTGGG

GATACAATGGTTACGGTGTTTTAGGACACGCTAACACAACTAACTTATCAGTACCAACAGAAATTCCATCAATCACTTGG

GCAAATGGTTCTTCTGATCCTGGTAAGATTAAAAAATTATTAGTTGATTCTCAACAATCATACCAAAGGGTTGCTATTCT

TACAGACAAAGGTAAGATATATTGGTGTGGAAGAAATGAATACGGTTGGGCGATGATGGGAAATACAACAGATGTTAATA

CATTTACTCAAATGTCTGGTGGACCTGGAAGCGGAACTAATTCATATTGCGCTAATATGTGGTTTACTGGAAATGGAAGA

TACGCAAGTTTCTGGACTAAAGATAGTACAGGTGCTATAAAATGTTGTGGTTACAATAGTTCTTATGAATTAGGAATTGG

AAATAACAACAACCAATCAGCTGCTGTATCACCTAAATGGCAAATTAATGGAACAACAACTGCTGATTTAGAAAATATTA

AAGACATAGGTTGCAATAGTGAATATGGTAACCAATGGATGTGTAATGTATGGGTATTAACTTACGATGGATTTATGTTT

AATACTGGAAGAAACAACTATGGAATTGGTTGTCAAGGTTATTCTTCTAGTTATAATGACAGACAATCAACAAACAATAT

AGAAGAAACAGATGATTATTACTTCCAAATGCAAAGAATGCCGAATTATGCACACGGCAGAATCGAAGATGTAAGAGGAA

GAGGTTATTACTCAACTGATGGTAACAGATACCACTTTAGAGAAATTAGAACATTTGATAACAGGTACTTATTATGGGGT

TATGGTGGAGATTATATAATGGGACAAAATGATGGAAATTACCACTCAACTGCTCAACCACCTGTTCTTGGATAATAAAT

ATAATAGAAGGTAAATAGGAAAACAAAATGGCAAAAATAAATCTCGGTAGAATTAAACTCCAATTCCAAGGAGAGTTCAA

TAGAGACCAAATGTACAGAAGAGACGATATTGTCTACCACTCAAATGCGATGTGGATTATGACAAATGAATATCTTCCAG

ATGGTTCTAGTGCTTACGCTCCAGGAAGTAAAGTTCAAGGTTACAATGTAAAAGAAAAAAATATTGTACAAGGTGGACAA

GATCCAAACTATAACGGTGCTGACGCATTTAGTTATACACAATATTGGACAGAAAACGAAAGAAAAGCTGAAAGACAAAG

AACAGATAGAGACGGAAATCCTGTTCAATACAATTCAACTTATGGCTCTAACGAAGATGGAGCACACGAAATTAATTATA

ACCAAATAGATACTCAATTAGGAACAATTGTACGCCATCAATCCCACCTAATGGATGAGTATGACGCTATGTTCCGAGAA

ACAGAAGACGACTATTCAGAAATGGATACTTACAATGGTTATGAACAAACTTATTTTAGATACCACTACAGACCAGTAGA

CAATACTTTTGATGTTGCTGTTAATGTTTCTGGTGGTGTACCAGATTTCAAAATTGACAATAGAGTAGGTTCATCTACTA

AAGGAAGACAATTTGCTGGTTACAGAAACTTTGAGTTTGTTAAAGAAGGTCATAGATATGCTTTCGTACAACACAAAAAT

TCAAACAAATTTTATCCGTTAGGATTTTCTTATACTGCCGATGGTATTCATAATACTGGAAATACAGGTAAGTCATTAGG

ACAAGACCACGATGGTCCTTACTATGTTAAAGGTACTGCTTCAAATGGTGATAGTGGTTTCTTTTCTCCATTATACAAAA

CTGCTGTTGCTGCTAACGCTGAAGATACAAGACGAGGTGGACAAGGTGTTTCTCACAAATTAACTTTTGACCAAGGTGAT

GTACCTGGTTATGAAACAACTTCTGTTGCTTTACAAGGTTGGTTACACGATGACGGAACACAAAGAAAAGATACACAAGT

TTCTGTTCTAACAGACGCTTCTGATAACACTTACTTACAGGTTACAAATGCTTGGGAAGGAACTACTTCTGGTGGTGCTT

CTACAAGAACAAGAAAAACAATTTACTTAAACACAGGTGATTCAACTGAACATCACGCACAAACAGGAACAACTTATTCT

TATGTTTATGTTGATGGTGCTTTACAAGTTGGTTCAAATATAACTGCTGTTATAACACAAAGTAGAGACGATACAACAAC

TGCTAATAATGGTGCTGGAAGACAAAGACTATTAGTAAACGGAAAACCTGTTTACCAATTAGTTGCTGAAGCTTCTAATG

TTGTTGTTGGTGGTATATCTGGTGCTTATCAGGCAATAGATAATACTGGTACTGGAACAACTACTGCTTTAGGTTCATCT

CCAACTTCTAACGAAGATAAGATTGACCTTTATATGCCTAAACTTACAGACCCGACTGAAAATGCGGAAAGAACTTTCCA

AATGACGGTTGCTTCTAGTAAATTTAATGTAGATGGTGCTGTACCAACTGCTAATACGGTTAAATTAGAAGAAGGAAAAA

CTTATAAGTTTGACCAATCAGATTCTACAAACGCAAGTAAGACATTAAAATTCTCAACAACTAGTGATGGAACACACGCT

AGTGGTTCTGCATACACAACTGGAGTAACCACTTTTGGAACTCCTGGAAGTGCTGGTGCTCATACAATAATTAAAGTAAG

AGCTAATACTGCTAAACTTTATATTTACTGCCACGAAAGTGCTTCTCACGGATTTGCAACAGAAACATATGATAGTGCTA

CAAATTTAGGAAAATCATATGCACCTGCAAACATAATGAAATGGAGAGGTTTTGGTAAAAACGGATGGGTTAAATATTAC

CTAGATGGATACCAAGTAGATGAAAATACTTACATTGAGACATTTTTCAATTCAATTGGAGACAATGATGACCACGAGTA

CAGACAAAAAATGGAAAATGGCAAATGGAAAGGTGGAAAACAATACAACTTTGCAAACAAAGGTGAGAGAACGGTTGAGT

TATATGTACCTTATCAAACAACTCAATCTGAATCAGAAAAAACGGTTATATATCCATTCTGTTTAGAACCAACAACTGCT

AGTAGAGCTACTACTGGTATGTACAACGATTTAGGTTTCTCAATTGAGAAGTCTTGGAGAGGTTACAAACATTGGGACAA

ATTACAATCATCTTTAAGATTTAGAGGTGAGTATTCTGTTAACACACAATACAATCACAATGATGTTGTATCTTACAAAC

CTTACAAAAGAATATCAACTGGAGAGAAATGGTACAGACACGGTACTGGCTTATATAGAGCTATAAGAGATAACAAAGGT

AGACCACCTCAACACGGTTTCCAAGAACCAACAAGGTCTCCTTTAATGACTAAATCTTCGGTTACATCAAACAGACTTAC

TGGTTATGCTGACCACGAAAATAATAACGAAACAGGTAAAAACTATCCTCCACATATTCAATCATACCATAATGCTTGGG

AATCCTTTGCAGGTATGAACTCGCAAGAACAATGTGCTGGAGTTTGGTTCCCGAATAGAGGTCCAATCGCTTGGCCGTAT

AAAGACGGAAGAAGTGAAAATGGTAACATTTACAGATGTCATATGTACATTGATAAGAATGGTGCTGTATGGACAATTGG

ACACGGTACTTCTGCTTCTAATATGGAAAAAGACCGTTCATCTTCATACTTTAGAGAAGTAACCTTTAGATGGAGAGATT

TCTACAATTCAGAAAGTAGAAACGAAGGTGGATACAATCATAGAAAAGGACCTAAATGGTCTCGTTATGATAGAATGAGA

ACACCAAGAGCTATCCAAATAGAAATGTCTTATGACGCAACAATGATTCTATTTGATAACGGAGAAATATTCCACGGTGG

ATATGGTTCACACGGACAACAAGGTACTGGTTATGACGGTGCTCCTGGTAATGCAATGTCACCTGACGGTGTTGAAGATG

TCCACTTCATTAAAATGACAATGAAAATTCAAAACGAAGATTCAATACATACTCCTTGCGCTTTAACAGACGAAGGTGAT

GTATATGTTTGGGGTTACAATGGTTATGGCGAAGTTGGTGATGGTAGAACTCAACACGCATACGGACCAAAAAGAATTCC

AAGAGAATGGTTTAATGACGAGAAGATTATAGATATTACTTGCTCAGGCGGTGATAGTACATCTTTCTATGCTAGAACTT

CACAGGATAACATTTATGCTTGGGGAAGAAATAACATAGGTCAATTAGGAGATACAACAACTACAGACAAATACAGACCA

GTATTGATGACAGGATTTAATGCTTCTGATAATGGTGGTATCGCTGTATGGCAAGCTTGTTCTCACTCATCTAACTCTTG

TTTCCAAATATTAGATGGAAACGGATATATTTGGAGTACAGGTAAAAACGATTATGGTAACTTCTTTGATAACTCAACAA

CTGATAGAAGTACTATGACACAGGCAACTGCTTCTCCTGCTGGAGATATAGTTGACTTTTGGGCTTGTAGATGGAACGGA

TATAGAACAACTTTCGTTAGATTGAAAAATGGAGAGACTTGGACTGCTGGACATTCTGGAGGTTATTACAATTCAGGTGA

TGGTGGAACAGGAACAAATACAAGTCCTGTACAAGTAGATAAGATTAACAACTTAAAAGAAGTTGCAATTTGCAACACTT

ATTCTGACCAAGGAAGAAGTTATTGGTTAACTGATAATGGGGAATTCTTCTGTCAAGGTAGGGATGTTTATTCATCTATG

CCTAATCCAATTGCTGGAGATAACTGGACTGGTGAAGATGGAACATACAAACCATATCACGCTTATGTACCAGCGGCAAC

AAGAATAAGAACAATGTGTATTCAAGGTATTGACCAATCAACTAACTATTACGGACTTCAACCAATGGTTGGAACGGAAG

ATGGTCAAGTGCTACTTTGGGGATTTTCTAGTAATAACAACTTGGGACACCACGCAACTGCTACCTGGTCAAACACAGGC

AGACCGATGATGTGGCAAGCTGGTAATGGTAGATAAAGTATAAATAGAAGTATAACAAAAAAAGAAAACGGAGATAAAAA

CAATGGCAAAAGTAATATATTCAATGACTGCTGGAATTGGTCAAGGTGATGATTATACTGCTCCTACAGGAGATACGCCT

ATTAGTTTAGGAGAGTTAAATGGTAAAGCATACTTCTCTATTGATGATGGTAATACTACCATTTCAACTAGTGGTGCAAA

TGATTCTGTATACGGTGTATCAGTAGTATCAGACGCAGACGAAAAAACAGCAATCAAAAATAGTAGCTCTTATGTTGAAC

AAGGTTTAGATAACCTAGACAACGATTTTATGGCAGGTAAAAATATGATAGATTTATTATCAGATGTGGCAGATGACACT

TCTGCAACTAAAACTGCTATCGCTGACCATAAAACTGCAAAAGCTAATTTTTTGACAAATTTAGGATTTTAATTAAACAA

GTATTAGGGAAAGTAATATGGCATTATCAATAACAGATTTTAAAGTAACCTGGAGAGGTGCTTGGAGAGATAAGGAAAGC

TACAAGAAAAATGATGTAGTTTACTGGAGAGGTAAATCTTATAGATGTATTGAAGATACACCTATGAATTATACTATTTC

TTCTGAAGCAATGATTAATACTAACTCGTATGGTCAATATCAACCTACGATTAGAAAAAGAAGCTATAGACCAGATGACA

GAAGATACTGGACATTATTACTAGCAGGTAACGATAACATTGAAACTTGGCAATATTGGAGACAATATGAAAGAGGCGAA

ATGGTTAAAGTTGCTGACAAAATTTATCTTTGTTTACAAAGAACAAGATATTGTAATACTTGGGTAGAAGAACACGATGG

AAGACCATCAAAATATTGGGAACTAATTTACATAAACGAAAACAAGTGGTGTACAAGAAACGAAGTTGTATCATTTAACA

ACCGAGCTCCGTTAGGTTGGAGATACAATATGGGAGTTTCACACACAGGTTGCTCAGACCAATCATATAGAACTTGTACT

TTATGTTCAGATGGTTCTGATATGTGGGTTGGTTCTTCTGACAATACCTCATCTTCAGGATTAGGAGAAGGTACTGCTGG

AAATGACGAACCTGCAAAACATATGTCAACAGGTTTCACATTTACAGATTGGATGGCGTCAACAGATAATCAATCTTGGA

ATATTAATGCAACAGGAAGAATGACTACTCCTGATGGTAAAGCTCCAAGAGTTATCCAAGTTTCAAAAAATTATAACAGA

ACATATTGGTTGTTTAACAATGGTGAAGTATATGCTTCAGGTAACAATGGAAATTATTCTTTAGGAAATTCAGAAACAAC

AGATAGACCTTATGCAGTTAGGGTAACGGCAAACGATACGCAAGACTGGCAAGGTAATACAATTGGAAAAACATATAACC

AAACTAGAATGGTTAAAGTAGGATTTTCAGACGAGGCACACGATAGTGGTACTACTTCTAATTGGTCATTAGGATCAGAT

GGAAGTGTATGGGTTTGGGGTTACAATAACAACGGTCAATTAGGACTTGGTAATCCTTCAATTAATAACTCAACAGATAC

TACTGGTGGACCAACTTCAACTGCTTTCTATAGTGCCAATATTCCTAGACCAGTAAGATTACCACAATCATATTTTGATG

GAAGAAGAATTGTAGATATGTGGTCTTCAGGTTCAGAAGAATGTTGGTTCCACGCACTTGACGATACAGGTCAACTATGG

GCGTGGGGACATAACCAATACGGTGAATTAGGAGTAGGTAACAGAAATGGAACTTATTACTATACAAAACCTACAAGAGT

TGGAATTAACTGGAACAGATACGGTGGAATAAAATTATACAAATCAACTTGGTCAAACGGTGGAAACTCTTCTACACACA

TTTTAGATGGTGAAGGATATATGTGGTTCACAGGTTATACAACTTCAGGCGCTTGGCCGATAGGTTCTCCAGGTTATACA

GATACGCACCATATTGGTTCGTTCAGAAGAGAAGGTCACTTTATAAATGGTGATATTGACTTCTTCTGGTGTGGTGGAGA

TGAAAACAAATGGTTGTATTTAAGACAAAAATCAACAGGTATGCTATGGGTACACGATGGTAACTATGGAACTTATGGTG

GTCGTGGACAATCAGTAGAAAGTAATGGATACTGGTATTCTTCAGGTGGTCACCCAGGAAGTTTCATACATCAAAAAGGT

CCTAAATGGGCGGTTAATGTATGTGATGTAGGTATGAGTAGAGCTGATGGTTCTTATATGTACTCTTTCCCAATGATACT

TGATGACGAAGGAATTATATGGGGTGGTGCTCCATATTCAAACGATGAACACGGTTTAGGTGGAGACTCAAGTAATAATG

ACCAATACACTAATGGTGGTCGTAATGACACGCAAGGTGCTATGGAAGACAATGAAATGTTTAGAACAAGAAAAAGAATT

GTATTCCAACCTGCAGGTGGTCATAGATGGACAGATTTATTCTATTCAGGAACTGGTTCTTCAAACATACCAAGAGCTCT

TAACCAAAGAGGTCAGGTATACTGGTGTGGATATGATGGTGGATCTTCGGTAACTCAACACTATGACTATTATGGTGAAG

GTGCTAATAGTAACCAAACTGCTTACTTCTTCCACTTGGGTCCTAGAGACTAACATAAATACTATATTATAGACCTGTTA

ACAGGTCACTATATAACAACTTGGAGTGAAAAATGAAAGACCTAGAAACCTTTATTGAAAAGGCACGAAACAACTATGAA

TCAATGGATTTCATAGTAGATTACAACAACAATAAACTCATCAAAGAAGTTAAAGGAACTTACTTTTATAATCAATGGTT

GGTTATAAATCAGTTAGAACATTTAACTTTAGAAATCACTAAAGACTTTCCCGAAATAAACATAAGAGAAAAGCTATATG

AGGCATTTAAACAAGAATGGCCTTATGAAGCTGATGATGTTTCAAAACCTTGGGTAGAACCATCAATGATGTATGGTACC

GAAGTATGGGTAAATCATTTAAAACCTTTAAAAGATACTCCACACAAACTAGTTGCTCAACTATACGCTACTCATAGCGA

AATACATAAAAATCAAAAATCTTCTATATTAGTTGACAAGTTAAAAACTTTATTTGAAAAATATTACAAAGACCATAAAG

AAGAAATGTTAGAGGAAGTTAAAATGTCTTGGGATTTTAAAAGAGGTTTAGTACAAGATTTAATGGCACACCAAGAACAT

ATGGAAGAGGTCTTACCTAGAATTGCTTTATTCAAAATTGGTGCAAAAGAAATAATGGAAGATAAATCAGGTATTAATAA

TATGTCTGCTGGTAATAGAGACGAAACAGAAGATATGAAAGTAAGAGCAGAATTAATGAAAAATGCGGTTACTATGAGAG

AAATGGATGTTGATGATTTGCCAGAAGAATATAAAGATTATGTTAACGAAGATATAAAGGCAGAACAACAAAAGAAAGAT

GAACTAGATAAAAAATTTAAAGAAGCACCAAAAAGATGAAGACATTAAAAGAACTTACTTGGGAACATCATAAAGAAGCT

GAACGCCAAGGATTTGTAAAAACAATTATGTCAGGTAAAATAAATCCTGAAATATATGGTATCTATCTTTTCAATCAACA

TCAATGTTATAATATGTTAGAAGCGTTAGCAATGTCAGAAGGTATCTTTGATGATATGCCTGAATTGAGACGAGCACCAT

CTATCAAAGCAGACTTTGATGAATTGTGGACATATAATTGGAAACCACCATTGATGGAATCTACAAGTAAATATTTGGAT

TATATTAATAAAAATTTAATGGATAATCCAGAAAAAATAGCTGCTCACATTTATGTAAGACATATGGGAGATTTATCTGG

TGGTCAAATGATAAGAAAAAAGATACCAGGTCAAGGAAAATATTATCAATTTAATATTAGATATGTTGAAGGTAGAAATC

AACCATATAAAAACATTAAAGAATTAAAAGAAGCATTAAGAACTAAAGTGGATAGTTATCAAAAGTATTCAGACCAAAGT

ACTATATCTGAAAATATTAATAGTGTTGTTTATGAAGCAAGAATATGTTTTGGATTTGCAACAGATTTATTTAAAGATAT

GAAAAAATTTATTGAACAAAACGAAAAGAGGTTTGGTGATGGCAATTTATAAACGAAGTAGAATATGGCAGATGTTAGAA

GAAACTACTAACTATCTTACAGCAGTTTTTGATAGAGAAGGTAAAGAAATATTTGAACCAACTATGGAAAAATTTAATCG

TCCAAAAGACGGTTGGGTTAATAGAGTATGGGAAACACCAGAAGCGAGAAGATGTCATTTAGATGTTGTAGACGCAAGAG

GAACAAAAGGTTTATATATGTTTCATTGTTGTGTATTTCCAAAACTAACACACCCAGGTCCAATATATGGACTTGATGTT

ATTGCTGGTGCAAAAAAGGTTACAGGTTTCTTCCACGACTTTTCTCCACTTGCAAAGAGAGACCATTCAATGGTTGATTG

GTTTGTGAAAGAAGCAAGTAATTATAAACCATCTAAAGTACGAGAACTACCTGATTGGGCAATGAAAATTTTTAGTCCTG

GTATGGTTGCTGCTAGTAATATAACACAAGAAAAAGAATTAAATGCCGCTTTAAGTTTAGCACAAACTAATTTAGGTGCT

TACTTTACATTATTAAGACGAGAAAAAGGAGAAGGAAATATACAAGAAATAAAAGACGCACAAAACAGATACGCAAAACA

TCAAAGAGAAAATCCTCATACGCCTAGAGTAATGAAGTCTTTAGGATTAAAAGATGAAGATGTTGAAGAATTTTGTACAA

ACGCATTATTTCCTTATGTTGAATAATGGAACATTTAGATAAATTTAAACAGGTCATAGACGATTATAAATCAGATGGAA

GATATAGAACTTTTAATGATATTATAAGAACAAGAGGAAAGTATCCTCACGCCATTTGGTATTCAAAATACTCAATCAAA

AATATTGTCAATTGGTGTTCCAACGATTATCTTGGAATGGGACAACATAACTATGTCATAGACTCTATGAAAACAGCACT

TGAAACGAGCGGAGCGGGTGCTGGAGGGACAAGAAACATATCAGGCACTACTCACTATCATAATGCTCTGGAACGAGAAC

TAGCGTCTCTCCATAAGAAAGAAAAAGCATTATTATTTACTTCTGCTTATAATGCCAATCAAACAACTTTAGAAACAATG

GGTAAGGTTATGCCTGATTTATTGTTTATATCAGACGCACAAAATCATTCTTCTATCATACAAGGTTTACGCCATAGTAG

ATGTAGAAAAGAAATATTTAAACATAATGATTTAGATGATTTAGAAAGTATTTTAAAATCTGAACCAGGTCCTAAATGTG

TAGTATTTGAAAGTGTATATTCTATGGACGGAGATATTGCTCCTGTAAAAGAAATAGCTGACTTATGTAAAAAGTATAAT

GCAATTTCTTATATTGATGAAGTACACGCTGTTGGTCTTTATGGAAAAGAAGGTGCTGGAATATGTGAAAGAGATAATGT

AGAAGTTGATATAATAAATGGAACATTAGCAAAGGCGTTCGGTGTACAAGGTGGATACATCGCAGGAAAGAGAGAGTTTA

TTGACACAATAAGAAGTATGGCTAGTGCTTTTATTTTTACAACTTCTGTAAGTCCAGTTATTTGTGCTGGTGCTTTAACG

AGTGTTAAGTATGTTAGAGACCATCCTGAATTAAGAGATAAGATACACGAAAGAGCAAACAAAACAAAAGAAGAACTTGA

AAGACAAGGAATAGAAGTTATGAAAAATGATAGTCATATTGTTCCTGTTATTATTGGAGAAGCTAAAAGATGTAAAGCAG

TATCAGATGAATTACTTTACAAAGAAGGTATCTATGTACAACCTATCAATTGGCCAACGGTTGCTGTAGGTACTGAAAGA

TTAAGATTTACTCCAACTCCATTTCATACAGATAATTTGATATTTGATATGGTAGTTAAAGTCAAAGCTGCTATTAAAAG

ATGTGGAAAGAAACTGAATTATGATTGATAAAATTATAGCTGATGGTGGAGATGGATTAGATGTCCTAATATATTGTCTA

AAACACGAACCTTTTATACAAGGAATTATATTATTTGGTCTGTTTTTAGCGATATTTTCTTGGTACTATGATAATAAAGT

AGATGATAAGGCCGTTTGGTCAAATAACGACCATCTATAAATTATAAATATAGCAAAGAATTAGAAGGAAATAACTATGG

CTCAACCTAATACAAGACAGACATTAATCTCTTATGCTAAAAGAGCATTGGGGCATCCTGTTATAGAGATAAATGTTGAT

GATGACCAAATAGATGATAGAGTAGATGAGGCGCTACAATACTGGCAACAATATCACTATGATGGTATCAAAAGAACTTA

TTTAAAATGGCAATATACACAAGCAGAAAAAAATAGAATCTTAACTAGTAATAGTGAAGCAGGAACAAAGAATTCTGTAA

CCTCTACTTGGAAAGAAGATAACAATTATATTGTTGTTCCAGAAACCGTATTTTCGGTTACAAATATATTTCCTTTTTCA

AACAAAGGTAATTTAAACTTATTTGATGTTAGATACCAATTAAGATTAAATGACTTATACGATTTCTCATCAACTTCTGT

TATTAACTATGATGTAGTTATGAGACAATTAGATTTCCTAGACCACATATTAGTTGGTGAAAAACCATTAAGATTTAACC

AACACGATAATAGATTATACATTGATATGGATTGGGAAAACGATTTAATGATAGATGAATATATTGTTATTGAATGCTAC

AGAAAAATGGATCCAGACACATATACAGATGTCTATAATGATATTTGGTTAAAGAAATATACAACTGCACTAGTTAAAAA

ACAATGGGGTGCTAATCTATCAAAATTTGCTGGTGTTGCTATGATAGGTGGTGTAACCTTAAATGGTGAACAAATCTATA

CACAGGCATTAGCAGATATAGAGAAGTTAGAGGAAGAAATAAAATCTCTACAAGAACACCAAGCACTAATGATAGGATAA

AAATAAAATGGCCGTTAATCATTATTTTCAAGGCGGCGATGGCATAGGTAGTCAAAGTGAGAAAAGATTAATAGAAGATT

TAATCGTAGAGAATTTAAAAATCTATGGACACGCTGTTTATTATTTACCGAGAACTCTAGTTAATAGAGATTTAATTCTT

GGTGAGGATTCTGCGTCTAGGTTTGACGACTCGTATCTAGTAGAAATGTATTTTGACACACCACAAGGGTTTGCTGGTGA

AGAAGAAATAATTAGTAAGTTTGGATTAGAAGTAAGAGACGATACAACTTTCGTTATTGCTAAAAGAAGATTCCAAGAAC

AAGTAGATGACCCAGCAAACCTAATGGTGGATGGCAGACCTAATGAAGGTGATGTTATTTACTATCCTTTAATGAATAGG

TTTTTTGAAATTGCGTTTGTTGAAGACCAGGAACCTTTCTTTCAATTAGGAAATTTACCTGTCTATAAATTAAGATGTAA

AACATTTGAATACTCTAGTGAAGAATTTAATACAGGTCACGCTGACATTGACCAAGCTGATGATAGAAAATCACTTGATA

CATCTTTGGCACACCAGTTTAGACTTGAAGATGGTACATTAAATCAATCTTCTTATAGTGGTTTCTTACAATTAGAAACA

GGAGATAAACACGGTAATCCTTGTTATTTAATTAATGAAGATTGGGACGACACTACAACTGATGGAGACGCTGCTGAAAG

TGTACAAACAAAATCTGCTTATGCTGATAATTTAGATTTAGATTCAGCTGCTGGTTTTGATACTGCAACGGTTAATGATG

ATATACTTGACTTCACAGAAAACAATCCATTTGGAGAAGTTAAATAATGGAAAGAGATAGACATAAACAACTAGTAGAAC

ATACTAATAGAATTAATAAAGAAAAAAAAACTTTAGAGTTATCTAAAACTTTAAGAAAAGAAGTTGAGATAGGTGCTACA

GGCACACAAAAATATAGATTTAAAAAAGGACCTAATAAAGGTAAGGTAGTATAATGTTTGGAACTCATTTTTATAACGAA

GGTATGAGAAGATTGACTATTGCTTTTGGTCAAATCTTTAATAAGATTGTTGTACAAACAAAAGACGCAAATGGTTCAGT

AGTTAAAAGATTTACGGTGCCATTAGCATATGCGCCAAAAGAAAAATTTATTGTTAGATTAACTCAACAAGGTGATTTAA

CAGATAAACAATTTGCAACGGTACTACCTCGTATGGGATTTGAAATA

>lcl|3300005430_____Ga0066849_10000198|410874_ __Eastern_Pacific_Ocean_-_ETNP201406SV69

AAATAGGGGAGAAAACAAGTGTCTCAAATAAAAGCTAGAATAGATTCAACAATTAGTAGACCTCAACAAGTATCGGTTAC

TATGCCTGCTGGAGCAGCGTCTCAAACTGCTGTTACCAACTCAACATTAAAATTAAGACTTCTATCCGATGTAGACGCAA

GTACCCTGGCTGATGGTTCAATGATTCAGTATTCTTCAACAAGTGATAAGTTTGTTGTAAGAGACGAAATCTCAACTACA

ACAGGTTCTATCACTTTAAATGGTGGTTCATTTTAAATAGAGAGAAAAGAATATGGCAACAATAATACGAATAAAAAGAA

GTGCTAACACAACTGCTCCTAGTACATTAAAACTTGGAGAATTAGCTTTAACTTATGGAACTGGTACTGCAGGAAATAAT

GGAGATAGATTATTTGCTGGAACAGGTGGGGTTGATGGAAATGGTGACGCAAACGATATTGATGTAATTGGTGGTAAGTA

TTTTACAAGTTTATTTCCTACTACAAACGGCGTAGTTGGTTCAGAAAAATTAATAACAACAGATTCAAATAACAGAATTG

ACCAAATGGTGTTTGGTAACTCAAATACTGATTCAGGTCAAATCACATTTAACGAAGCATTAAACAACGGTTCAAACAAT

GTTGTTTTAAAAGCTCCGTTATCATTAACAAACTCATCTACACTTTTATTACCTGATGGTGCTGGAAGTGCTGGACAATT

TTTAAAAGTAGATTCAGCAAGTGGTTCGGAAGCTCAATTAGGTTTCGCTGATGTTGATACTACTCTTACTTTAGAAGATA

GTACAGGAACATCAATTAACTATTCAACTGCAAACACTTTATTGTTAACAGGTGATGGTACAATTGATACTGCCGCTACT

GCTAACACAATTACAATTAAAGTACAAGATGGTTCAATTGGAACAACTCAACTTACAGACAACAATGTAACCAATGCTAA

ATTAGCAAACACTAGTGTTACCGTTGGAAGTACTGCTGTTGAATTAGGTGGTTCTGCATTAACAGACCTTGCTGGATTAA

CAAGTGCTATTGTTGATGACTTATCTTTAAACGGACAAGACATTTCAACAACAGGAAGTAATAAAGATATTACTTTAACT

CCACACGGAACTGGTGTTGTAACCGTACCTAGTGGATATAAAGATAGAGCAGGTTTTGGTGCTGACGCATTGGCAACTAA

AGAGTATGTTGATGGATTTACTTCAGGTTTAGATGTTAAAGATAGTTGTAGAGTTGCTACTACTGCTAACTTAACGGTTA

CTTATGACCAAACAAATAAAAGATTAGATAACGCTGACACACAAGCTGCTTTGGCAATTGATGGTGTTACCTTATCAGTT

AACGATAGAGTATTAGTTAAATCTCAAACAGAAGCAAGACAAAATGGAATTTACAAGGTAACAGATGTTGGTTCTAACTC

TTCTAATTGGAGATTAGAAAGAAGTTCAGATACGAATACAGGTGCTCAACTAACAGGTGGTTCATTTACTTTCGTTGAAG

AAGGAACTGCTAATGCTGATAATGGTTATACATTTACTCACAATGGTATACCTACATTAACAGACAATACTTTATCTAAC

AATACAGAATTACCAGTATCACAATTCTCGGGTGCTGGACAAGTAGTAGCTGGTGCTGCTCTTGTAAAAGCTGGGAATAC

TTTAGATGTTAATGTTGATAACGCTTCAATCGCTGTTGTATCAGACGCTTTACAAGTTAAAGCTGGTGGTATTACAAATG

CTATGTTGGCAGGAAGTATTACAAATGCTAATCTTGCTGATCCAAATGTTTCACTTGCAGGTGAAACTGGAACTGGTACG

GTTGGTCTTGGTGGTACATTAACTTTTACTGCTGGCGAAGGAATTGATACTTCTGCTTCTGGTTCTGCAATTACAATTGC

TGGGGAAAACGCTTCAACAACAAATAAAGGAGTTGCTTCTTTTAGTACTGATAACTTTACGGTAACAAGTGGTGATGTTG

CGGTTACTGAAATAGATGGTGGACCATTCTAATGGCTACCGTAATTAAACCTAAAAGAAGTTTTACGGCGACTGCTGTAC

CAACGGTTTCTGATTTAGAAATTGGTGAGTTGGCAATGAATGTTGCTGATGGTAAGTTTTTTACAAAATTAAATGCTAGT

ACTATTAAAGAAGTTGGTGGTGCAAGTGCTGTTAATATTCAATCAGTATTAAGTGCTGGTGCAATTTCAACAACAGATTT

AGTTTTTAATAATGCAAATATTATATTTGAAGGTGCTACACCTGACGCCTACGAAACAACATTAACGGTAGAAGACCCGA

CTGCTGATAGAACGGTTAAACTACCTAATTCAAGTGGAACTTTGGCGTTAACAGGTGATATTCTTGCTTTCGCTGTAGTA

TTTGGGGGATAATAAACAATGGCAAGTGCTTTTAAAAATGCTGGACAAGAAAATCCAACAACTGACGGTGTAGGTGCTAA

TGTATATACGGCTCCTTCTAATGGAACTGCTGTATTACACGCTGTTTATATCTCAAACAAGAATCCAGCTACTCAAGCAG

TAGTAGATGTATCGGTTACGATAGATGGTGGAACAACTTTTAGAAGTATTATAAAAGGTTGTATAATTCCACAGAATAAC

ACTTTTATATTAGACAAACCAGTCAATTTAGAAGCAAACGATATAATAAGAGTAGTGAGTAATGTGGCGAGTACGGATAC

TTTTATATCTGTACTAGAGAATACATAATAGTTAAAAGATAGATTATAAATAGTATAAATATAAACAAAGAGGAATTTTA

AATGGGATTAGCATTAGCAACAGGAGCAAGTCAAGCCGTTGGGGTAGACGCTGCTGGATTTCAGATTTCAAATGAATACG

CTATGCACGCCCTTAACCGTGATGTAAACGGACTATTGATTTATACAAAAACATCAATGGATAGTACGGATACTATTGAG

GTTAACGATGGACAAGGATTTGGTTATAATGGTTTTGAAGGACTTGCATTAGGAAAAGCAAGTGATGGTACAACCGTTCA

GAATACTTTACAAAGTACATATGACGAAAGTACAGACGACCACTATCAAACAAATGCTAAGTATAGAAAATATCAACAGG

TCAGATTTGACCCTTTGAAACTTTTTTATTTTATTAATGATGAAGGAATGTTAGTTGCGAGATACCAACACGACTATACA

TATGCTGCTACAGAAACGGCAACTTCAACTACTGGTAATAACTGGATACCTTCTGGTGGCGTTTATTATACACAAGCCAA

TCAGGAAAGGTATTTGTAAAACAAAAATTAGAGAGAGATAAAAATGGCAGATTTTATTTTAGGTAGACTTAAATTTCACT

TCAAAGGTGATTGGGTTACCTCAACAGCATATATTAAAGATGATGTTGTAAGATATGGTGGAAATAGTTTCGTTGCTATG

GCGAACCATACATCTTCAGCACTTTTTGAAACCGACCTTACATCAACAAAATGGAAAAAAATGGCAGCTGGGCAAGACTG

GAAAGGTGCTTGGGCAGCTACTACATTTTATAAAGTGGACGATGTTGTACAATGGGGAGGTTCTACCTTCGCTTGTAATG

AAGCACACACTTCACAAGCAGATTTATATGACGACACATCAAAATGGACATCTTTTGTTCCAGGATTTAAATGGACAGGT

ACTTACACAAATGGTACTGCATACAAAGTTAACGATTTAGCAAAATATGGTGCAAATGTTTATATTTGTTCCGTAGAACA

TACTGCCGCTTCTACAATAGATACTGCTAAATTTTCTATGTTCGTATCAGGACTAGAATTTGAAGATTCATACAACGCTG

GAACAGCTTACCAAGCTGGAGACATTGTATCTTATGGTGGATATAATTATGTTGCTAAAGTACAAAGTACTGGCGAAACA

CCATATAACAATGCTACTAAATGGGAAGTATTAACAACTGGTTATAAAATGGTTGGTACCTATGCAGGTGCAACCGCTTA

TAAAACTGGTGATGTAGTAATGTATGGTGGTCATACTTATGTTGCAAAAACAGACGCAACAGGAATTGTACCAACAACAA

CTGCAACTTGGGATTTATTAAACGAAGGTTTAAAATGGAATGATAGCTGGACTGACGGAGCTGAATATGCTCCAGGTGAC

GCTGTTGCTTATGGTTCATCTTCTTATAGATGTAAACTTGCTCATACTGCTACTGCTGTAGCTGGTGACGCTAAAAGACC

AGATTACGATACAGGTGGAGTTTATTGGGACTTACTTGCTGAAGGAGATTCAAACTTTGTTACCACTACAAGAGGTGACT

TATTAACTAGAAACGCAACACAGAATGTTAGATTAGGAATTGGAACAACTGGTTCAATGCTTAAATCTGATGGTACAGAT

GTTGCTTGGGCACTTGCTCAAACTAACGACAATGTATATTTTGTTGCACCTCACGGAACAGACGCTTTACCAAGTACAGA

CGCTGGTAGAGGAACTTCTTTAGATAAACCTTGGGCGTCAATTAAATACGCTTGTGATTGGTTAAAAGACACAGGTAACT

CTGGATTAGATTACACGGTTACGGTTACAGATTCAACTAACTTTACAATTACTTTAGGCACATCAACTTATGCTCACACA

TATTCAACTGGTGGTAAAGTACACAAATCAGATTTTAGTACGGTTAACATAACAAACGCTCCATATAACAATGGAACAGG

TGTGGTAACTATTACAACTTCAGGTGCTCACGGATTATCAACTAGTGATAAAGTAAAACTATCAGGAATAGTTTATACTT

GTTCTGCTGGAACAAAAACTTATCCAAAAGATACGGTTAACAAAACTCTTTATGTTAAAACTGGAGAGTATACAGAAGCA

TTACCAATTGTTGTTCCTGCGAACACACAAATGATTGGTGACGGAGTAAGAAGTACAAGAATTAAACCTGCTTCAGGTAA

TTCAGTAGTTGCTGGATTAACTAACACACCTAACGCAAGAGCAGATATGCTTAGAGTTAGAAATGGTGTTACCATTACAG

GATTTACTTTTTCTGGAATGACTGGAACAATGGGTGCTGCTGACGCAAATGGCGTAGCAAGACCAAATACTGCTGACGGT

GCAACTCGTTCAGGAGTAGTTATTGCTTTAGACCCAGGAACTGGAACTACAGATACTGCTGCTTGGATAGAAACTAAATC

ACCATTTATACAAAATTGTACACACTTTGGAACAGGTTCAGTTGGTATTAAGATTGATGGTTCACTACACGGTGGTGGAT

ATTCTTCAATTCTTGCAAACGACTTTACACAAATTTCAAGTGATGGTGTTGGTTGCTGGGCATTATCAAATGCTAAATCA

GAATTAGTATCAGTATTCACTTATTACTGCCATCACGGTTATTTGGCTGATAGTGGAGCTGTTATCAGAAGTTTGAACTC

TAACAACTCTTATGGAGAATATGGTTCAACTGCTGCTGGTATTGACGCAAACGAAACACCTTATACAGGTTCAGTAGATT

TAAGAAACAATGAAGCTAAAGTAGGAAGAACTTTAGTTGCTGGTTCTGGAATCGGAAGATTAGAAATGGAATATGCTGGA

GAAGGTTATTCTTCTGCTACAATTGCAATCGGAGGTTCTGGTGCTTCAGGTGCTGTAACCGCTGGATTTAGTGATGGTGC

TGTAAAACACATCACGGTTGCAACAACAGGTTCTACACACTTTACAACTTCTGCTTTCGCACAATCAGGAACTTCAACTA

CATTAAAACTTGCTGCTTCTGATTCACAACCAGACGATTTCTATAATGGAATGAGAATAAATGTTTACACAGGAACTGGT

TATGGTAATACTGGTATTATCGCTGACTATGTTGCTTCTACAAAAACTTGTTCAGTACAAAAAGAAAATGGTACTGCAGG

TTGGGATGTATGGGTAAACTCTGGTTTATCTTCTGCTACATCTTTTGATACAACATCTGGTTACGAAATAGAACCAAGAG

TAGTTTTATCTGGTGGTGGTTCTCCAACAAGAGACGCATTAGCAAGAGCTGTTGTAGATAATCAACAAATTTCAAAAATT

TATATATTAGACGGTGGTGCTGGATATACTTCTGCACCTACGATAACAATTACAGACCCTAACGCTTCAACGGTTGGTAC

TGCTACTTCTGAAATAGGAGATGGAGTAATATCACAAACAACGGTTGGAACTGCTGGTTCTGGATACAAAACAGAAACAA

CAACTGCTACCGTATCTGGTAATGGTTATGCTGAAATATCAAGTGAAGGTACTGCCTTTGTTAGATTAACAGGATTATCA

AAATCACCTACAGGTGGAGACATTGTAGAGTTTGCTGGAATAACTGGACAAGCTTACTATGTTGTGGCGGTTACAGGATA

TTCTGCTGGTGCTGGATTAGTTAGAGTAAATCCAAAATTTACAACTGCTAATAAACCAACTCACGGAGAGACTGCAACTT

TAAGAAGTAATTACTCAAACATAAGATTAACAGGACACGACTTCTTGGATGTTGGTACTGGAGATATTACAACAACTAAT

TATCCAAATGTCGCTACACAGGCGCCAGACGCTAATGATGAAATCTTTGAAGCTGATAGAGGAAGAGTATTCTATTCTTC

AACAGACCAAGATGGTAACTTTAGAGTAGGTAATTTATTTAAAATTGAACAGGCAACTGGTAAGGCAACATTGAACGCTG

AAGCATTTGACCTTTCTGGACTACAAGAATTAAGTCTAGGTTCAAGTGCTCAAGGAAACTTTGGTGCTACAATTGCTGAA

TTTAGTACCGATGGTACTTTGGCAGACAATTCTGATACTGCTTTAGTAACCGAAAGAGCAATTAAGACATATGTAGATGG

ACAACTTGGTGGTGGACAAAATGACTTGTCAGTTAACTCATTAACGGCTGGTTCAATTACTGCTACAGGACAAACAATAT

CTACAACAGGATTAAGTGGAACAGATGTAAACTTAACTATTGGTACACAAAACGATGGTATTATTACTCTTTCTGCTCAA

GCACAAACAAGTATTACACCAACAAGTGGCAACGACCTTGTTAATAAGTCATATGTTGACGCACAAGGAACACCAACATT

GCAAACACTTTCTATTGATGATGTAGATTTATCATTAAAAAGAAGAGTCATTACAAATGCTAATGAACTTATACAAAAAG

AAAGTACTCAATTTGATGGTACGGATGCAACTGAAGGATTTGAATTTATTAACGGCACAATGCAAATTAATATTGACAAA

GCTGGAGATTTGGTAATAGAAACAATATAAATATAGTTAAATTAGGAAGATAAACAATGGCAACAACAAAACAAAGAATT

GGAAATCTGTTCTTCAATTATCAAGGAGAATATTCAGCTACAAAGACTTATTACCACGATGATGTTGTACAATACAACAA

CACGGATTGGATTTGTACAAAGAATTCTGCTACAACAGGAACTGCTCCAGTAGACAATGAAAGAAGATATGTAAGAATTA

CTAAAGCTGTTTCAGCGAGTACAGGTTCTAACGCATACAAATGGGATGGCGAAGCTACTTGGCCGCAAACAGAATTACAA

TATAAAATTGGTGATACTTTGGTATTATACCAAGATGGAAACGATTTTGATGATAACAAAATTGCTTTTTCAGATTCATC

TACAAACAAAAATACAAATTTATTCCACAAAGATGTTACCTATTTCTTAAATGGCAAAGCTGTTGGTGCTGGTACGGCAA

CTAATGAATACTTTAATTCATCAGCTTTTAACAATGCTACTAAAAGAGAAATTAGAATAGAATTTACAAGTGAAACACCT

AAAGAAATTTATATGTTTAACTACGATAATCCAAGTGCCAATTGGGGTCCTAAAATAGTTGTTGCTGACCATTCAATTTG

GAAACCGATTAGACAATCATTTAAATGGAGAGGTGAACACGATAACACAAACGATAGTGGTTCATATCAAACTTATTACG

CAAATGACATAGTAAGAATAGTTGTTCCTTTAGATAACGACTTTTCTGAAACGGTTATGGACCAAGAACATATCCAATCT

ACAAGAGCAACTTATATTTGTATAAGACAACATACTTGTGATGGTACTGACAAATATTTACCTTGGGACCAAGAAGCTGA

TACAGACGACAATAAGTATTGGGAAAGAATTTCTGAAGAAATGCAATTTGACGATGAAATCGTTGAAGATAGTGGAGCTG

TTGCTACAATTACAAATGTATCTGCTGCTTCTCCAAGTAGAAAACAAGGATTTTACAGATATGTAAATTGTAATAACATA

ACTGCCGCTGCTGAAAATAGTGGCGCAGGAAGAACTGGTGGATATAATACACCAATGTTTGATGTTGAAGTAGAAGGTTA

TCAATCTGCAAAGACTTGGACGATTGAAACAAGTGCTCATTACAAAAGAAAAAAAGGAAAATACAATAATGTAACCCACA

CTTCTTCAAGTGGTGGTGGTTCAAACTCCTATTGGGATTTTGAAGTTGACCAAGAAGGAAAAGTAATTAAAGCTGTTCTT

ACGAAGAAAAATCTTGGTGGTGATATGGGTGGATTAGGATATGCTGTTAACGAAGAATTAACTTTTGCTGACGCAAGTTT

CGGTGGCGGTGGTGCTGCTGATGTAGTCTTAAAGATAACTGCAATAGGAACTTGGGGTGCATTAAACATAAATTTATGTA

AAGACGATAGACAAGGAAGACAACACGCACAATGGTATAACGACAACGAGGCACCAGTATTTGGTGGAGAAAATAACGCT

ACTAATGACCAATTAGGATTTGATGGACAAATATTTTATTCTTCTGCCAATGTAACCTTTGATGTTGCAAGTACAAGTAA

AAAAGCTAGAGGTTATGCTACAAGATTTTCAGGAAATAGATTAGAGTGTATGAGTTTATGTAATACAAATGGTCCAATTG

GAGACGATAACAAGTATTACAGATTACCTGGACAATTCCAACAAGCTAACTGCGTTAACTGGCCGTGTTTTATTAATGGT

CGTGGTGGTATTACAAGTTGGGGTTCAAACTCAACTGGACAAAACGGAATTAATCAAGGTTCTGTACTAATGGGAGTTGG

AATGACTTTCCCATTTACAGATTGGTATAGAAGTACAGATAACGGTGGTAGTGGTATTCATACTACTCCTGATGGAGAAC

CACCTAAAGCTATTCAATTAATATCTGGATATGAAACTGGTATGGCATTATTTAACAATGGCGAAATATACCATTGGGGT

TATGGAGGTCACGGACAAAGTGGTGACGCTGCTACTTCAACTAGAAGTTATCCAGTAAGATGTGGTGGAACATATCAGGA

AGTTTATGCTGCTGCTAATGCTTCAACACACACTTTAATGTCTACAAGAATTAAAAGAATATGGATGACTAATTGGGGTG

GAGACAATAATACAAGTACTCATAGTTGCTATGCGTTAGATACTGATGGCGAATTATGGGCGTGGGGTTATAATGGATAC

GGACAATTAGGAGATAATACTACAACAGAAAGAAATAGACCACAAAAAATTAACAAGACTTCTTACTTTAATGGTAATAA

GATTGTTGCTTTCTGGACTGCTGGAGCAGGATACGCTTTCTGTTTCGCTTTAGATGACGCTGGAAAATTATATAGTTGGG

GATACAATGGTTACGGTGTTTTAGGACACGCTAACACAACTAACTTATCAGTACCAACAGAAATTCCATCAATCACTTGG

GCAAATGGTTCTTCTGATCCTGGTAAGATTAAAAAATTATTAGTTGATTCTCAACAATCATACCAAAGGGTTGCTATTCT

TACAGACAAAGGTAAGATATATTGGTGTGGAAGAAATGAATACGGTTGGGCGATGATGGGAAATACAACAGATGTTAATA

CATTTACTCAAATGTCTGGTGGACCTGGAAGCGGAACTAATTCATATTGCGCTAATATGTGGTTTACTGGAAATGGAAGA

TACGCAAGTTTCTGGACTAAAGATAGTACAGGTGCTATAAAATGTTGTGGTTACAATAGTTCTTATGAATTAGGAATTGG

AAATAACAACAACCAATCAGCTGCTGTATCACCTAAATGGCAAATTAATGGAACAACAACTGCTGATTTAGAAAATATTA

AAGACATAGGTTGCAATAGTGAATATGGTAACCAATGGATGTGTAATGTATGGGTATTAACTTACGATGGATTTATGTTT

AATACTGGAAGAAACAACTATGGAATTGGTTGTCAAGGTTATTCTTCTAGTTATAATGACAGACAATCAACAAACAATAT

AGAAGAAACAGATGATTATTACTTCCAAATGCAAAGAATGCCGAATTATGCACACGGCAGAATCGAAGATGTAAGAGGAA

GAGGTTATTACTCAACTGATGGTAACAGATACCACTTTAGAGAAATTAGAACATTTGATAACAGGTACTTATTATGGGGT

TATGGTGGAGATTATATAATGGGACAAAATGATGGAAATTACCACTCAACTGCTCAACCACCTGTTCTTGGATAATAAAT

ATAATAGAAGGTAAATAGGAAAACAAAATGGCAAAAATAAATCTCGGTAGAATTAAACTCCAATTCCAAGGAGAGTTCAA

TAGAGACCAAATGTACAGAAGAGACGATATTGTCTACCACTCAAATGCGATGTGGATTATGACAAATGAATATCTTCCAG

ATGGTTCTAGTGCTTACGCTCCAGGAAGTAAAGTTCAAGGTTACAATGTAAAAGAAAAAAATATTGTACAAGGTGGACAA

GATCCAAACTATAACGGTGCTGACGCATTTAGTTATACACAATATTGGACAGAAAACGAAAGAAAAGCTGAAAGACAAAG

AACAGATAGAGACGGAAATCCTGTTCAATACAATTCAACTTATGGCTCTAACGAAGATGGAGCACACGAAATTAATTATA

ACCAAATAGATACTCAATTAGGAACAATTGTACGCCATCAATCCCACCTAATGGATGAGTATGACGCTATGTTCCGAGAA

ACAGAAGACGACTATTCAGAAATGGATACTTACAATGGTTATGAACAAACTTATTTTAGATACCACTACAGACCAGTAGA

CAATACTTTTGATGTTGCTGTTAATGTTTCTGGTGGTGTACCAGATTTCAAAATTGACAATAGAGTAGGTTCATCTACTA

AAGGAAGACAATTTGCTGGTTACAGAAACTTTGAGTTTGTTAAAGAAGGTCATAGATATGCTTTCGTACAACACAAAAAT

TCAAACAAATTTTATCCGTTAGGATTTTCTTATACTGCCGATGGTATTCATAATACTGGAAATACAGGTAAGTCATTAGG

ACAAGACCACGATGGTCCTTACTATGTTAAAGGTACTGCTTCAAATGGTGATAGTGGTTTCTTTTCTCCATTATACAAAA

CTGCTGTTGCTGCTAACGCTGAAGATACAAGACGAGGTGGACAAGGTGTTTCTCACAAATTAACTTTTGACCAAGGTGAT

GTACCTGGTTATGAAACAACTTCTGTTGCTTTACAAGGTTGGTTACACGATGACGGAACACAAAGAAAAGATACACAAGT

TTCTGTTCTAACAGACGCTTCTGATAACACTTACTTACAGGTTACAAATGCTTGGGAAGGAACTACTTCTGGTGGTGCTT

CTACAAGAACAAGAAAAACAATTTACTTAAACACAGGTGATTCAACTGAACATCACGCACAAACAGGAACAACTTATTCT

TATGTTTATGTTGATGGTGCTTTACAAGTTGGTTCAAATATAACTGCTGTTATAACACAAAGTAGAGACGATACAACAAC

TGCTAATAATGGTGCTGGAAGACAAAGACTATTAGTAAACGGAAAACCTGTTTACCAATTAGTTGCTGAAGCTTCTAATG

TTGTTGTTGGTGGTATATCTGGTGCTTATCAGGCAATAGATAATACTGGTACTGGAACAACTACTGCTTTAGGTTCATCT

CCAACTTCTAACGAAGATAAGATTGACCTTTATATGCCTAAACTTACAGACCCGACTGAAAATGCGGAAAGAACTTTCCA

AATGACGGTTGCTTCTAGTAAATTTAATGTAGATGGTGCTGTACCAACTGCTAATACGGTTAAATTAGAAGAAGGAAAAA

CTTATAAGTTTGACCAATCAGATTCTACAAACGCAAGTAAGACATTAAAATTCTCAACAACTAGTGATGGAACACACGCT

AGTGGTTCTGCATACACAACTGGAGTAACCACTTTTGGAACTCCTGGAAGTGCTGGTGCTCATACAATAATTAAAGTAAG

AGCTAATACTGCTAAACTTTATATTTACTGCCACGAAAGTGCTTCTCACGGATTTGCAACAGAAACATATGATAGTGCTA

CAAATTTAGGAAAATCATATGCACCTGCAAACATAATGAAATGGAGAGGTTTTGGTAAAAACGGATGGGTTAAATATTAC

CTAGATGGATACCAAGTAGATGAAAATACTTACATTGAGACATTTTTCAATTCAATTGGAGACAATGATGACCACGAGTA

CAGACAAAAAATGGAAAATGGCAAATGGAAAGGTGGAAAACAATACAACTTTGCAAACAAAGGTGAGAGAACGGTTGAGT

TATATGTACCTTATCAAACAACTCAATCTGAATCAGAAAAAACGGTTATATATCCATTCTGTTTAGAACCAACAACTGCT

AGTAGAGCTACTACTGGTATGTACAACGATTTAGGTTTCTCAATTGAGAAGTCTTGGAGAGGTTACAAACATTGGGACAA

ATTACAATCATCTTTAAGATTTAGAGGTGAGTATTCTGTTAACACACAATACAATCACAATGATGTTGTATCTTACAAAC

CTTACAAAAGAATATCAACTGGAGAGAAATGGTACAGACACGGTACTGGCTTATATAGAGCTATAAGAGATAACAAAGGT

AGACCACCTCAACACGGTTTCCAAGAACCAACAAGGTCTCCTTTAATGACTAAATCTTCGGTTACATCAAACAGACTTAC

TGGTTATGCTGACCACGAAAATAATAACGAAACAGGTAAAAACTATCCTCCACATATTCAATCATACCATAATGCTTGGG

AATCCTTTGCAGGTATGAACTCGCAAGAACAATGTGCTGGAGTTTGGTTCCCGAATAGAGGTCCAATCGCTTGGCCGTAT

AAAGACGGAAGAAGTGAAAATGGTAACATTTACAGATGTCATATGTACATTGATAAGAATGGTGCTGTATGGACAATTGG

ACACGGTACTTCTGCTTCTAATATGGAAAAAGACCGTTCATCTTCATACTTTAGAGAAGTAACCTTTAGATGGAGAGATT

TCTACAATTCAGAAAGTAGAAACGAAGGTGGATACAATCATAGAAAAGGACCTAAATGGTCTCGTTATGATAGAATGAGA

ACACCAAGAGCTATCCAAATAGAAATGTCTTATGACGCAACAATGATTCTATTTGATAACGGAGAAATATTCCACGGTGG

ATATGGTTCACACGGACAACAAGGTACTGGTTATGACGGTGCTCCTGGTAATGCAATGTCACCTGACGGTGTTGAAGATG

TCCACTTCATTAAAATGACAATGAAAATTCAAAACGAAGATTCAATACATACTCCTTGCGCTTTAACAGACGAAGGTGAT

GTATATGTTTGGGGTTACAATGGTTATGGCGAAGTTGGTGATGGTAGAACTCAACACGCATACGGACCAAAAAGAATTCC

AAGAGAATGGTTTAATGACGAGAAGATTATAGATATTACTTGCTCAGGCGGTGATAGTACATCTTTCTATGCTAGAACTT

CACAGGATAACATTTATGCTTGGGGAAGAAATAACATAGGTCAATTAGGAGATACAACAACTACAGACAAATACAGACCA

GTATTGATGACAGGATTTAATGCTTCTGATAATGGTGGTATCGCTGTATGGCAAGCTTGTTCTCACTCATCTAACTCTTG

TTTCCAAATATTAGATGGAAACGGATATATTTGGAGTACAGGTAAAAACGATTATGGTAACTTCTTTGATAACTCAACAA

CTGATAGAAGTACTATGACACAGGCAACTGCTTCTCCTGCTGGAGATATAGTTGACTTTTGGGCTTGTAGATGGAACGGA

TATAGAACAACTTTCGTTAGATTGAAAAATGGAGAGACTTGGACTGCTGGACATTCTGGAGGTTATTACAATTCAGGTGA

TGGTGGAACAGGAACAAATACAAGTCCTGTACAAGTAGATAAGATTAACAACTTAAAAGAAGTTGCAATTTGCAACACTT

ATTCTGACCAAGGAAGAAGTTATTGGTTAACTGATAATGGGGAATTCTTCTGTCAAGGTAGGGATGTTTATTCATCTATG

CCTAATCCAATTGCTGGAGATAACTGGACTGGTGAAGATGGAACATACAAACCATATCACGCTTATGTACCAGCGGCAAC

AAGAATAAGAACAATGTGTATTCAAGGTATTGACCAATCAACTAACTATTACGGACTTCAACCAATGGTTGGAACGGAAG

ATGGTCAAGTGCTACTTTGGGGATTTTCTAGTAATAACAACTTGGGACACCACGCAACTGCTACCTGGTCAAACACAGGC

AGACCGATGATGTGGCAAGCTGGTAATGGTAGATAAAGTATAAATAGAAGTATAACAAAAAAAGAAAACGGAGATAAAAA

CAATGGCAAAAGTAATATATTCAATGACTGCTGGAATTGGTCAAGGTGATGATTATACTGCTCCTACAGGAGATACGCCT

ATTAGTTTAGGAGAGTTAAATGGTAAAGCATACTTCTCTATTGATGATGGTAATACTACCATTTCAACTAGTGGTGCAAA

TGATTCTGTATACGGTGTATCAGTAGTATCAGACGCAGACGAAAAAACAGCAATCAAAAATAGTAGCTCTTATGTTGAAC

AAGGTTTAGATAACCTAGACAACGATTTTATGGCAGGTAAAAATATGATAGATTTATTATCAGATGTGGCAGATGACACT

TCTGCAACTAAAACTGCTATCGCTGACCATAAAACTGCAAAAGCTAATTTTTTGACAAATTTAGGATTTTAATTAAACAA

GTATTAGGGAAAGTAATATGGCATTATCAATAACAGATTTTAAAGTAACCTGGAGAGGTGCTTGGAGAGATAAGGAAAGC

TACAAGAAAAATGATGTAGTTTACTGGAGAGGTAAATCTTATAGATGTATTGAAGATACACCTATGAATTATACTATTTC

TTCTGAAGCAATGATTAATACTAACTCGTATGGTCAATATCAACCTACGATTAGAAAAAGAAGCTATAGACCAGATGACA

GAAGATACTGGACATTATTACTAGCAGGTAACGATAACATTGAAACTTGGCAATATTGGAGACAATATGAAAGAGGCGAA

ATGGTTAAAGTTGCTGACAAAATTTATCTTTGTTTACAAAGAACAAGATATTGTAATACTTGGGTAGAAGAACACGATGG

AAGACCATCAAAATATTGGGAACTAATTTACATAAACGAAAACAAGTGGTGTACAAGAAACGAAGTTGTATCATTTAACA

ACCGAGCTCCGTTAGGTTGGAGATACAATATGGGAGTTTCACACACAGGTTGCTCAGACCAATCATATAGAACTTGTACT

TTATGTTCAGATGGTTCTGATATGTGGGTTGGTTCTTCTGACAATACCTCATCTTCAGGATTAGGAGAAGGTACTGCTGG

AAATGACGAACCTGCAAAACATATGTCAACAGGTTTCACATTTACAGATTGGATGGCGTCAACAGATAATCAATCTTGGA

ATATTAATGCAACAGGAAGAATGACTACTCCTGATGGTAAAGCTCCAAGAGTTATCCAAGTTTCAAAAAATTATAACAGA

ACATATTGGTTGTTTAACAATGGTGAAGTATATGCTTCAGGTAACAATGGAAATTATTCTTTAGGAAATTCAGAAACAAC

AGATAGACCTTATGCAGTTAGGGTAACGGCAAACGATACGCAAGACTGGCAAGGTAATACAATTGGAAAAACATATAACC

AAACTAGAATGGTTAAAGTAGGATTTTCAGACGAGGCACACGATAGTGGTACTACTTCTAATTGGTCATTAGGATCAGAT

GGAAGTGTATGGGTTTGGGGTTACAATAACAACGGTCAATTAGGACTTGGTAATCCTTCAATTAATAACTCAACAGATAC

TACTGGTGGACCAACTTCAACTGCTTTCTATAGTGCCAATATTCCTAGACCAGTAAGATTACCACAATCATATTTTGATG

GAAGAAGAATTGTAGATATGTGGTCTTCAGGTTCAGAAGAATGTTGGTTCCACGCACTTGACGATACAGGTCAACTATGG

GCGTGGGGACATAACCAATACGGTGAATTAGGAGTAGGTAACAGAAATGGAACTTATTACTATACAAAACCTACAAGAGT

TGGAATTAACTGGAACAGATACGGTGGAATAAAATTATACAAATCAACTTGGTCAAACGGTGGAAACTCTTCTACACACA

TTTTAGATGGTGAAGGATATATGTGGTTCACAGGTTATACAACTTCAGGCGCTTGGCCGATAGGTTCTCCAGGTTATACA

GATACGCACCATATTGGTTCGTTCAGAAGAGAAGGTCACTTTATAAATGGTGATATTGACTTCTTCTGGTGTGGTGGAGA

TGAAAACAAATGGTTGTATTTAAGACAAAAATCAACAGGTATGCTATGGGTACACGATGGTAACTATGGAACTTATGGTG

GTCGTGGACAATCAGTAGAAAGTAATGGATACTGGTATTCTTCAGGTGGTCACCCAGGAAGTTTCATACATCAAAAAGGT

CCTAAATGGGCGGTTAATGTATGTGATGTAGGTATGAGTAGAGCTGATGGTTCTTATATGTACTCTTTCCCAATGATACT

TGATGACGAAGGAATTATATGGGGTGGTGCTCCATATTCAAACGATGAACACGGTTTAGGTGGAGACTCAAGTAATAATG

ACCAATACACTAATGGTGGTCGTAATGACACGCAAGGTGCTATGGAAGACAATGAAATGTTTAGAACAAGAAAAAGAATT

GTATTCCAACCTGCAGGTGGTCATAGATGGACAGATTTATTCTATTCAGGAACTGGTTCTTCAAACATACCAAGAGCTCT

TAACCAAAGAGGTCAGGTATACTGGTGTGGATATGATGGTGGATCTTCGGTAACTCAACACTATGACTATTATGGTGAAG

GTGCTAATAGTAACCAAACTGCTTACTTCTTCCACTTGGGTCCTAGAGACTAACATAAATACTATATTATAGACCTGTTA

ACAGGTCACTATATAACAACTTGGAGTGAAAAATGAAAGACCTAGAAACCTTTATTGAAAAGGCACGAAACAACTATGAA

TCAATGGATTTCATAGTAGATTACAACAACAATAAACTCATCAAAGAAGTTAAAGGAACTTACTTTTATAATCAATGGTT

GGTTATAAATCAGTTAGAACATTTAACTTTAGAAATCACTAAAGACTTTCCCGAAATAAACATAAGAGAAAAGCTATATG

AGGCATTTAAACAAGAATGGCCTTATGAAGCTGATGATGTTTCAAAACCTTGGGTAGAACCATCAATGATGTATGGTACC

GAAGTATGGGTAAATCATTTAAAACCTTTAAAAGATACTCCACACAAACTAGTTGCTCAACTATACGCTACTCATAGCGA

AATACATAAAAATCAAAAATCTTCTATATTAGTTGACAAGTTAAAAACTTTATTTGAAAAATATTACAAAGACCATAAAG

AAGAAATGTTAGAGGAAGTTAAAATGTCTTGGGATTTTAAAAGAGGTTTAGTACAAGATTTAATGGCACACCAAGAACAT

ATGGAAGAGGTCTTACCTAGAATTGCTTTATTCAAAATTGGTGCAAAAGAAATAATGGAAGATAAATCAGGTATTAATAA

TATGTCTGCTGGTAATAGAGACGAAACAGAAGATATGAAAGTAAGAGCAGAATTAATGAAAAATGCGGTTACTATGAGAG

AAATGGATGTTGATGATTTGCCAGAAGAATATAAAGATTATGTTAACGAAGATATAAAGGCAGAACAACAAAAGAAAGAT

GAACTAGATAAAAAATTTAAAGAAGCACCAAAAAGATGAAGACATTAAAAGAACTTACTTGGGAACATCATAAAGAAGCT

GAACGCCAAGGATTTGTAAAAACAATTATGTCAGGTAAAATAAATCCTGAAATATATGGTATCTATCTTTTCAATCAACA

TCAATGTTATAATATGTTAGAAGCGTTAGCAATGTCAGAAGGTATCTTTGATGATATGCCTGAATTGAGACGAGCACCAT

CTATCAAAGCAGACTTTGATGAATTGTGGACATATAATTGGAAACCACCATTGATGGAATCTACAAGTAAATATTTGGAT

TATATTAATAAAAATTTAATGGATAATCCAGAAAAAATAGCTGCTCACATTTATGTAAGACATATGGGAGATTTATCTGG

TGGTCAAATGATAAGAAAAAAGATACCAGGTCAAGGAAAATATTATCAATTTAATATTAGATATGTTGAAGGTAGAAATC

AACCATATAAAAACATTAAAGAATTAAAAGAAGCATTAAGAACTAAAGTGGATAGTTATCAAAAGTATTCAGACCAAAGT

ACTATATCTGAAAATATTAATAGTGTTGTTTATGAAGCAAGAATATGTTTTGGATTTGCAACAGATTTATTTAAAGATAT

GAAAAAATTTATTGAACAAAACGAAAAGAGGTTTGGTGATGGCAATTTATAAACGAAGTAGAATATGGCAGATGTTAGAA

GAAACTACTAACTATCTTACAGCAGTTTTTGATAGAGAAGGTAAAGAAATATTTGAACCAACTATGGAAAAATTTAATCG

TCCAAAAGACGGTTGGGTTAATAGAGTATGGGAAACACCAGAAGCGAGAAGATGTCATTTAGATGTTGTAGACGCAAGAG

GAACAAAAGGTTTATATATGTTTCATTGTTGTGTATTTCCAAAACTAACACACCCAGGTCCAATATATGGACTTGATGTT

ATTGCTGGTGCAAAAAAGGTTACAGGTTTCTTCCACGACTTTTCTCCACTTGCAAAGAGAGACCATTCAATGGTTGATTG

GTTTGTGAAAGAAGCAAGTAATTATAAACCATCTAAAGTACGAGAACTACCTGATTGGGCAATGAAAATTTTTAGTCCTG

GTATGGTTGCTGCTAGTAATATAACACAAGAAAAAGAATTAAATGCCGCTTTAAGTTTAGCACAAACTAATTTAGGTGCT

TACTTTACATTATTAAGACGAGAAAAAGGAGAAGGAAATATACAAGAAATAAAAGACGCACAAAACAGATACGCAAAACA

TCAAAGAGAAAATCCTCATACGCCTAGAGTAATGAAGTCTTTAGGATTAAAAGATGAAGATGTTGAAGAATTTTGTACAA

ACGCATTATTTCCTTATGTTGAATAATGGAACATTTAGATAAATTTAAACAGGTCATAGACGATTATAAATCAGATGGAA

GATATAGAACTTTTAATGATATTATAAGAACAAGAGGAAAGTATCCTCACGCCATTTGGTATTCAAAATACTCAATCAAA

AATATTGTCAATTGGTGTTCCAACGATTATCTTGGAATGGGACAACATAACTATGTCATAGACTCTATGAAAACAGCACT

TGAAACGAGCGGAGCGGGTGCTGGAGGGACAAGAAACATATCAGGCACTACTCACTATCATAATGCTCTGGAACGAGAAC

TAGCGTCTCTCCATAAGAAAGAAAAAGCATTATTATTTACTTCTGCTTATAATGCCAATCAAACAACTTTAGAAACAATG

GGTAAGGTTATGCCTGATTTATTGTTTATATCAGACGCACAAAATCATTCTTCTATCATACAAGGTTTACGCCATAGTAG

ATGTAGAAAAGAAATATTTAAACATAATGATTTAGATGATTTAGAAAGTATTTTAAAATCTGAACCAGGTCCTAAATGTG

TAGTATTTGAAAGTGTATATTCTATGGACGGAGATATTGCTCCTGTAAAAGAAATAGCTGACTTATGTAAAAAGTATAAT

GCAATTTCTTATATTGATGAAGTACACGCTGTTGGTCTTTATGGAAAAGAAGGTGCTGGAATATGTGAAAGAGATAATGT

AGAAGTTGATATAATAAATGGAACATTAGCAAAGGCGTTCGGTGTACAAGGTGGATACATCGCAGGAAAGAGAGAGTTTA

TTGACACAATAAGAAGTATGGCTAGTGCTTTTATTTTTACAACTTCTGTAAGTCCAGTTATTTGTGCTGGTGCTTTAACG

AGTGTTAAGTATGTTAGAGACCATCCTGAATTAAGAGATAAGATACACGAAAGAGCAAACAAAACAAAAGAAGAACTTGA

AAGACAAGGAATAGAAGTTATGAAAAATGATAGTCATATTGTTCCTGTTATTATTGGAGAAGCTAAAAGATGTAAAGCAG

TATCAGATGAATTACTTTACAAAGAAGGTATCTATGTACAACCTATCAATTGGCCAACGGTTGCTGTAGGTACTGAAAGA

TTAAGATTTACTCCAACTCCATTTCATACAGATAATTTGATATTTGATATGGTAGTTAAAGTCAAAGCTGCTATTAAAAG

ATGTGGAAAGAAACTGAATTATGATTGATAAAATTATAGCTGATGGTGGAGATGGATTAGATGTCCTAATATATTGTCTA

AAACACGAACCTTTTATACAAGGAATTATATTATTTGGTCTGTTTTTAGCGATATTTTCTTGGTACTATGATAATAAAGT

AGATGATAAGGCCGTTTGGTCAAATAACGACCATCTATAAATTATAAATATAGCAAAGAATTAGAAGGAAATAACTATGG

CTCAACCTAATACAAGACAGACATTAATCTCTTATGCTAAAAGAGCATTGGGGCATCCTGTTATAGAGATAAATGTTGAT

GATGACCAAATAGATGATAGAGTAGATGAGGCGCTACAATACTGGCAACAATATCACTATGATGGTATCAAAAGAACTTA

TTTAAAATGGCAATATACACAAGCAGAAAAAAATAGAATCTTAACTAGTAATAGTGAAGCAGGAACAAAGAATTCTGTAA

CCTCTACTTGGAAAGAAGATAACAATTATATTGTTGTTCCAGAAACCGTATTTTCGGTTACAAATATATTTCCTTTTTCA

AACAAAGGTAATTTAAACTTATTTGATGTTAGATACCAATTAAGATTAAATGACTTATACGATTTCTCATCAACTTCTGT

TATTAACTATGATGTAGTTATGAGACAATTAGATTTCCTAGACCACATATTAGTTGGTGAAAAACCATTAAGATTTAACC

AACACGATAATAGATTATACATTGATATGGATTGGGAAAACGATTTAATGATAGATGAATATATTGTTATTGAATGCTAC

AGAAAAATGGATCCAGACACATATACAGATGTCTATAATGATATTTGGTTAAAGAAATATACAACTGCACTAGTTAAAAA

ACAATGGGGTGCTAATCTATCAAAATTTGCTGGTGTTGCTATGATAGGTGGTGTAACCTTAAATGGTGAACAAATCTATA

CACAGGCATTAGCAGATATAGAGAAGTTAGAGGAAGAAATAAAATCTCTACAAGAACACCAAGCACTAATGATAGGATAA

AAATAAAATGGCCGTTAATCATTATTTTCAAGGCGGCGATGGCATAGGTAGTCAAAGTGAGAAAAGATTAATAGAAGATT

TAATCGTAGAGAATTTAAAAATCTATGGACACGCTGTTTATTATTTACCGAGAACTCTAGTTAATAGAGATTTAATTCTT

GGTGAGGATTCTGCGTCTAGGTTTGACGACTCGTATCTAGTAGAAATGTATTTTGACACACCACAAGGGTTTGCTGGTGA

AGAAGAAATAATTAGTAAGTTTGGATTAGAAGTAAGAGACGATACAACTTTCGTTATTGCTAAAAGAAGATTCCAAGAAC

AAGTAGATGACCCAGCAAACCTAATGGTGGATGGCAGACCTAATGAAGGTGATGTTATTTACTATCCTTTAATGAATAGG

TTTTTTGAAATTGCGTTTGTTGAAGACCAGGAACCTTTCTTTCAATTAGGAAATTTACCTGTCTATAAATTAAGATGTAA

AACATTTGAATACTCTAGTGAAGAATTTAATACAGGTCACGCTGACATTGACCAAGCTGATGATAGAAAATCACTTGATA

CATCTTTGGCACACCAGTTTAGACTTGAAGATGGTACATTAAATCAATCTTCTTATAGTGGTTTCTTACAATTAGAAACA

GGAGATAAACACGGTAATCCTTGTTATTTAATTAATGAAGATTGGGACGACACTACAACTGATGGAGACGCTGCTGAAAG

TGTACAAACAAAATCTGCTTATGCTGATAATTTAGATTTAGATTCAGCTGCTGGTTTTGATACTGCAACGGTTAATGATG

ATATACTTGACTTCACAGAAAACAATCCATTTGGAGAAGTTAAATAATGGAAAGAGATAGACATAAACAACTAGTAGAAC

ATACTAATAGAATTAATAAAGAAAAAAAAACTTTAGAGTTATCTAAAACTTTAAGAAAAGAAGTTGAGATAGGTGCTACA

GGCACACAAAAATATAGATTTAAAAAAGGACCTAATAAAGGTAAGGTAGTATAATGTTTGGAACTCATTTTTATAACGAA

GGTATGAGAAGATTGACTATTGCTTTTGGTCAAATCTTTAATAAGATTGTTGTACAAACAAAAGACGCAAATGGTTCAGT

AGTTAAAAGATTTACGGTGCCATTAGCATATGCGCCAAAAGAAAAATTTATTGTTAGATTAACTCAACAAGGTGATTTAA

CAGATAAACAATTTGCAACGGTACTACCTCGTATGGGATTTGAAATA

>lcl|3300005521_____Ga0066862_10001059|142651_ __Eastern_Pacific_Ocean_-_ETNP2014F10-02SV255

GGACTTCAACCAATGGTTGGAACGGAAGATGGTCAAGTGCTACTTTGGGGATTTTCTAGTAATAACAACTTGGGACACCA

CGCAACTGCTACCTGGTCAAACACAGGCAGACCGATGATGTGGCAAGCTGGTAATGGTAGATAAAGTATAAATAGAAGTA

TAACAAAAAAAGAAAACGGAGATAAAAACAATGGCAAAAGTAATATATTCAATGACTGCTGGAATTGGTCAAGGTGATGA

TTATACTGCTCCTACAGGAGATACGCCTATTAGTTTAGGAGAGTTAAATGGTAAAGCATACTTCTCTATTGATGATGGTA

ATACTACCATTTCAACTAGTGGTGCAAATGATTCTGTATACGGTGTATCAGTAGTATCAGACGCAGACGAAAAAACAGCA

ATCAAAAATAGTAGCTCTTATGTTGAACAAGGTTTAGATAACCTAGACAACGATTTTATGGCAGGTAAAAATATGATAGA

TTTATTATCAGATGTGGCAGATGACACTTCTGCAACTAAAACTGCTATCGCTGACCATAAAACTGCAAAAGCTAATTTTT

TGACAAATTTAGGATTTTAATTAAACAAGTATTAGGGAAAGTAATATGGCATTATCAATAACAGATTTTAAAGTAACCTG

GAGAGGTGCTTGGAGAGATAAGGAAAGCTACAAGAAAAATGATGTAGTTTACTGGAGAGGTAAATCTTATAGATGTATTG

AAGATACACCTATGAATTATACTATTTCTTCTGAAGCAATGATTAATACTAACTCGTATGGTCAATATCAACCTACGATT

AGAAAAAGAAGCTATAGACCAGATGACAGAAGATACTGGACATTATTACTAGCAGGTAACGATAACATTGAAACTTGGCA

ATATTGGAGACAATATGAAAGAGGCGAAATGGTTAAAGTTGCTGACAAAATTTATCTTTGTTTACAAAGAACAAGATATT

GTAATACTTGGGTAGAAGAACACGATGGAAGACCATCAAAATATTGGGAACTAATTTACATAAACGAAAACAAGTGGTGT

ACAAGAAACGAAGTTGTATCATTTAACAACCGAGCTCCGTTAGGTTGGAGATACAATATGGGAGTTTCACACACAGGTTG

CTCAGACCAATCATATAGAACTTGTACTTTATGTTCAGATGGTTCTGATATGTGGGTTGGTTCTTCTGACAATACCTCAT

CTTCAGGATTAGGAGAAGGTACTGCTGGAAATGACGAACCTGCAAAACATATGTCAACAGGTTTCACATTTACAGATTGG

ATGGCGTCAACAGATAATCAATCTTGGAATATTAATGCAACAGGAAGAATGACTACTCCTGATGGTAAAGCTCCAAGAGT

TATCCAAGTTTCAAAAAATTATAACAGAACATATTGGTTGTTTAACAATGGTGAAGTATATGCTTCAGGTAACAATGGAA

ATTATTCTTTAGGAAATTCAGAAACAACAGATAGACCTTATGCAGTTAGGGTAACGGCAAACGATACGCAAGACTGGCAA

GGTAATACAATTGGAAAAACATATAACCAAACTAGAATGGTTAAAGTAGGATTTTCAGACGAGGCACACGATAGTGGTAC

TACTTCTAATTGGTCATTAGGATCAGATGGAAGTGTATGGGTTTGGGGTTACAATAACAACGGTCAATTAGGACTTGGTA

ATCCTTCAATTAATAACTCAACAGATACTACTGGTGGACCAACTTCAACTGCTTTCTATAGTGCCAATATTCCTAGACCA

GTAAGATTACCACAATCATATTTTGATGGAAGAAGAATTGTAGATATGTGGTCTTCAGGTTCAGAAGAATGTTGGTTCCA

CGCACTTGACGATACAGGTCAACTATGGGCGTGGGGACATAACCAATACGGTGAATTAGGAGTAGGTAACAGAAATGGAA

CTTATTACTATACAAAACCTACAAGAGTTGGAATTAACTGGAACAGATACGGTGGAATAAAATTATACAAATCAACTTGG

TCAAACGGTGGAAACTCTTCTACACACATTTTAGATGGTGAAGGATATATGTGGTTCACAGGTTATACAACTTCAGGCGC

TTGGCCGATAGGTTCTCCAGGTTATACAGATACGCACCATATTGGTTCGTTCAGAAGAGAAGGTCACTTTATAAATGGTG

ATATTGACTTCTTCTGGTGTGGTGGAGATGAAAACAAATGGTTGTATTTAAGACAAAAATCAACAGGTATGCTATGGGTA

CACGATGGTAACTATGGAACTTATGGTGGTCGTGGACAATCAGTAGAAAGTAATGGATACTGGTATTCTTCAGGTGGTCA

CCCAGGAAGTTTCATACATCAAAAAGGTCCTAAATGGGCGGTTAATGTATGTGATGTAGGTATGAGTAGAGCTGATGGTT

CTTATATGTACTCTTTCCCAATGATACTTGATGACGAAGGAATTATATGGGGTGGTGCTCCATATTCAAACGATGAACAC

GGTTTAGGTGGAGACTCAAGTAATAATGACCAATACACTAATGGTGGTCGTAATGACACGCAAGGTGCTATGGAAGACAA

TGAAATGTTTAGAACAAGAAAAAGAATTGTATTCCAACCTGCAGGTGGTCATAGATGGACAGATTTATTCTATTCAGGAA

CTGGTTCTTCAAACATACCAAGAGCTCTTAACCAAAGAGGTCAGGTATACTGGTGTGGATATGATGGTGGATCTTCGGTA

ACTCAACACTATGACTATTATGGTGAAGGTGCTAATAGTAACCAAACTGCTTACTTCTTCCACTTGGGTCCTAGAGACTA

ACATAAATACTATATTATAGACCTGCTTTACTATGGGTCACTATATAACAACTTGGAGTGAAAAATGAAAGACCTAGAAA

CCTTTATTGAAAAGGCACGAAACAACTATGAATCAATGGATTTCATAGTAGATTACAACAACAATAAACTCATCAAAGAA

GTTAAAGGAACTTACTTTTATAATCAATGGTTGGTTATAAATCAGTTAGAACATTTAACTTTAGAAATCACTAAAGACTT

TCCCGAAATAAACATAAGAGAAAAGCTATATGAGGCATTTAAACAAGAATGGCCTTATGAAGCTGATGATATTTCAAAAC

CTTGGGTAGAACCATCAATGATGTATGGTACCGAAGTATGGGTAAATCATTTAAAACCTTTAAAAGATACTCCACACAAA

CTAGTTGCTCAACTATACGCTACTCATAGCGAAATACATAAAAATCAAAAATCTTCTATATTAGTTGACAAGTTAAAAAC

TTTATTTGAAAAATATTACAAAGACCATAAAGAAGAAATGTTAGAGGAAGTTAAAATGTCTTGGGATTTTAAAAGAGGTT

TAGTACAAGATTTAATGGCACACCAAGAACATATGGAAGAGGTCTTACCTAGAATTGCTTTATTCAAAATTGGTGCAAAA

GAAATAATGGAAGATAAATCAGGTATTAATAATATGTCTGCTGGTAATAGAGACGAAACAGAAGATATGAAAGTAAGAGC

AGAATTAATGAAAAATGCGGTTACTATGAGAGAAATGGATGTTGATGATTTGCCAGAAGAATATAAAGATTATGTTAACG

AAGATATAAAGGCAGAACAACAAAAGAAAGATGAACTAGATAAAAAATTTAAAGAAGCACCAAAAAGATGAAGACATTAA

AAGAACTTACTTGGGAACATCATAAAGAAGCTGAACGCCAAGGATTTGTAAAAACAATTATGTCAGGTAAAATAAATCCT

GAAATATATGGTATCTATCTTTTCAATCAACATCAATGTTATAATATGTTAGAAGCGTTAGCAATGTCAGAAGGTATCTT

TGATGATATGCCTGAATTGAGACGAGCACCATCTATCAAAGCAGACTTTGATGAATTGTGGACATATAATTGGAAACCAC

CATTGATGGAATCTACAAGTAAATATTTGGATTATATTAATAAAAATTTAATGGATAATCCAGAAAAAATAGCTGCTCAC

ATTTATGTAAGACATATGGGAGATTTATCTGGTGGTCAAATGATAAGAAAAAAGATACCAGGTCAAGGAAAATATTATCA

ATTTAATATTAGATATGTTGAAGGTAGAAATCAACCATATAAAAACATTAAAGAATTAAAAGAAGCATTAAGAACTAAAG

TGGATAGTTATCAAAAGTATTCAGACCAAAGTACTATATCTGAAAATATTAATAGTGTTGTTTATGAAGCAAGAATATGT

TTTGGATTTGCAACAGATTTATTTAAAGATATGAAAAAATTTATTGAACAAAACGAAAAGAGGTTTGGTGATGGCAATTT

ATAAACGAAGTAGAATATGGCAGATGTTAGAAGAAACTACTAACTATCTTACAGCAGTTTTTGATAGAGAAGGTAAAGAA

ATATTTGAACCAACTATGGAAAAATTTAATCGTCCAAAAGACGGTTGGGTTAATAGAGTATGGGAAACACCAGAAGCGAG

AAGATGTCATTTAGATGTTGTAGACGCAAGAGGAACAAAAGGTTTATATATGTTTCATTGTTGTGTATTTCCAAAACTAA

CACACCCAGGTCCAATATATGGACTTGATGTTATTGCTGGTGCAAAAAAGGTTACAGGTTTCTTCCACGACTTTTCTCCA

CTTGCAAAGAGAGACCATTCAATGGTTGATTGGTTTGTGAAAGAAGCAAGTAATTATAAACCATCTAAAGTACGAGAACT

ACCTGATTGGGCAATGAAAATTTTTAGTCCTGGTATGGTTGCTGCTAGTAATATAACACAAGAAAAAGAATTAAATGCCG

CTTTAAGTTTAGCACAAACTAATTTAGGTGCTTACTTTACATTATTAAGACGAGAAAAAGGAGAAGGAAATATACAAGAA

ATAAAAGACGCACAAAACAGATACGCAAAACATCAAAGAGAAAATCCTCATACGCCTAGAGTAATGAAGTCTTTAGGATT

AAAAGATGAAGATGTTGAAGAATTTTGTACAAACGCATTATTTCCTTATGTTGAATAATGGAACATTTAGATAAATTTAA

ACAGGTCATAGACGATTATAAATCAGATGGAAGATATAGAACTTTTAATGATATTATAAGAACAAGAGGAAAGTATCCTC

ACGCCATTTGGTATTCAAAATACTCAATCAAAAATATTGTCAATTGGTGTTCCAACGATTATCTTGGAATGGGACAACAT

AACTATGTCATAGACTCTATGAAAACAGCACTTGAAACGAGCGGAGCGGGTGCTGGAGGGACAAGAAACATATCAGGCAC

TACTCACTATCATAATGCTCTGGAACGAGAACTAGCGTCTCTCCATAAGAAAGAAAAAGCATTATTATTTACTTCTGCTT

ATAATGCCAATCAAACAACTTTAGAAACAATGGGTAAGGTTATGCCTGATTTATTGTTTATATCAGACGCACAAAATCAT

TCTTCTATCATACAAGGTTTACGCCATAGTAGATGTAGAAAAGAAATATTTAAACATAATGATTTAGATGATTTAGAAAG

TATTTTAAAATCTGAACCAGGTCCTAAATGTGTAGTATTTGAAAGTGTATATTCTATGGACGGAGATATTGCTCCTGTAA

AAGAAATAGCTGACTTATGTAAAAAGTATAATGCAATTTCTTATATTGATGAAGTACACGCTGTTGGTCTTTATGGAAAA

GAAGGTGCTGGAATATGTGAAAGAGATAATGTAGAAGTTGATATAATAAATGGAACATTAGCAAAGGCGTTCGGTGTACA

AGGTGGATACATCGCAGGAAAGAGAGAGTTTATTGACACAATAAGAAGTATGGCTAGTGCTTTTATTTTTACAACTTCTG

TAAGTCCAGTTATTTGTGCTGGTGCTTTAACGAGTGTTAAGTATGTTAGAGACCATCCTGAATTAAGAGATAAGATACAC

GAAAGAGCAAACAAAACAAAAGAAGAACTTGAAAGACAAGGAATAGAAGTTATGAAAAATGATAGTCATATTGTTCCTGT

TATTATTGGAGAAGCTAAAAGATGTAAAGCAGTATCAGATGAATTACTTTACAAAGAAGGTATCTATGTACAACCTATCA

ATTGGCCAACGGTTGCTGTAGGTACTGAAAGATTAAGATTTACTCCAACTCCATTTCATACAGATAATTTGATATTTGAT

ATGGTAGTTAAAGTCAAAGCTGCTATTAAAAGATGTGGAAAGAAACTGAATTATGATTGATAAAATTATAGCTGATGGTG

GAGATGGATTAGATGTCCTAATATATTGTCTAAAACACGAACCTTTTATACAAGGAATTATATTATTTGGTCTGTTTTTA

GCGATATTTTCTTGGTACTATGATAATAAAGTAGATGATAAGGCCGTTTGGTCAAATAACGACCATCTATAAATTATAAA

TATAGCAAAGAATTAGAAGGAAATAACTATGGCTCAACCTAATACAAGACAGACATTAATCTCTTATGCTAAAAGAGCAT

TGGGGCATCCTGTTATAGAGATAAATGTTGATGATGACCAAATAGATGATAGAGTAGATGAGGCGCTACAATATTGGCAA

CAATATCACTATGATGGTATCAAAAGAACTTATTTAAAATGGCAATATACACAAGCAGAAAAAAATAGAATCTTAACTAG

TAATAGTGAAGCAGGAACAAAGAATTCTGTAACCTCTACTTGGAAAGAAGATAACAATTATATTGTTGTTCCAGAAACCG

TATTTTCGGTTACAAATATATTTCCTTTTTCAAACAAAGGTAATTTAAACTTATTTGATGTTAGATACCAATTAAGATTA

AATGACTTATACGATTTCTCATCAACTTCTGTTATTAACTATGATGTAGTTATGAGACAATTAGATTTCCTAGACCACAT

ATTAGTTGGTGAAAAACCATTAAGATTTAACCAACACGATAATAGATTATACATTGATATGGATTGGGAAAACGATTTAA

TGATAGATGAATATATTGTTATTGAATGCTACAGAAAAATGGATCCAGACACATATACAGATGTCTATAATGATATTTGG

TTAAAGAAATATACAACTGCACTAGTTAAAAAACAATGGGGTGCTAATCTATCAAAATTTGCTGGTGTTGCTATGATAGG

TGGTGTAACCTTAAATGGTGAACAAATCTATACACAGGCATTAGCAGATATAGAGAAGTTAGAGGAAGAAATAAAATCTC

TACAAGAACACCAAGCACTAATGATAGGATAAAAATAAAATGGCCGTTAATCATTATTTTCAAGGCGGCGATGGCATAGG

TAGTCAAAGTGAGAAAAGATTAATAGAAGATTTAATCGTAGAGAATTTAAAAATCTATGGACACGCTGTTTATTATTTAC

CGAGAACTCTAGTTAATAGAGATTTAATTCTTGGTGAGGATTCTGCGTCTAGGTTTGACGACTCGTATCTAGTAGAAATG

TATTTTGACACACCACAAGGGTTTGCTGGTGAAGAAGAAATAATTAGTAAGTTTGGATTAGAAGTAAGAGACGATACAAC

TTTCGTTATTGCTAAAAGAAGATTCCAAGAACAAGTAGATGACCCAGCAAACCTAATGGTGGATGGCAGACCTAATGAAG

GTGATGTTATTTACTATCCTTTAATGAATAGGTTTTTTGAAATTGCGTTTGTTGAAGACCAGGAACCTTTCTTTCAATTA

GGAAATTTACCTGTCTATAAATTAAGATGTAAAACATTTGAATACTCTAGTGAAGAATTTAATACAGGTCACGCTGACAT

TGACCAAGCTGATGATAGAAAATCACTTGATACATCTTTGGCACACCAGTTTAGACTTGAAGATGGTACATTAAATCAAT

CTTCTTATAGTGGTTTCTTACAATTAGAAACAGGAGATAAACACGGTAATCCTTGTTATTTAATTAATGAAGATTGGGAC

GACACTACAACTGATGGAGACGCTGCTGAAAGTGTACAAACAAAATCTGCTTATGCTGATAATTTAGATTTAGATTCAGC

TGCTGGTTTTGATACTGCAACGGTTAATGATGATATACTTGACTTCACAGAAAACAATCCATTTGGAGAAGTTAAATAAT

GGAAAGAGATAGACATAAACAACTAGTAGAACATACTAATAGAATTAATAAAGAAAAAAAAACTTTAGAGTTATCTAAAA

CTTTAAGAAAAGAAGTTGAGATAGGTGCTACAGGCACACAAAAATATAGATTTAAAAAAGGACCTAATAAAGGTAAGGTA

GTATAATGTTTGGAACTCATTTTTATAACGAAGGTATGAGAAGATTGACTATTGCTTTTGGTCAAATCTTTAATAAGATT

GTTGTACAAACAAAAGACGCAAATGGTTCAGTAGTTAAAAGATTTACGGTGCCATTAGCATATGCGCCAAAAGAAAAATT

TATTGTTAGATTAACTCAACAAGGTGATTTAACAGATAAACAATTTGCAACGGTACTACCTCGTATGGGATTTGAAATAG

AAGGTATAGAATATGACCCTAGTAGAAAGTTAAATAAATTACAAAAATTTAGAAAACCAAACACAGATGGTTCTTCTACG

GATCAAGCTAATAAAATGGACTTTAACTATACTCCAGTTCCATATAATATAACATATAAATTGTTTATATTTACAGCAAC

TGCTGAAAATGGTTTACAAATTTTAGAACAAATAGTACCGTACTTTCAACCAGATTATACGGTTACAATTAATATGGTTC

CTGATTTAGGAATTAAGCGTGATGTTCCAATTGTAATTGGAGACATACAATACGAAGATAGTTATAGTGGAGATTTTGAA

ACTAGAAGAGCAGTAATATATACTATGACCTTTACTGCTAAAACTTATCTATACGGACCTTCTACAACAGCAGGTGTTGT

TAGAAAAGTACAAACAGATTTAGGAACTGATTCAGTCAGTAAGGCAAGAGAAGAAAGAATAGTAATTACTCCTGACCCTA

CAACAGCAAAACCTGGTGATGATTTTGGATTTACAACAACTATATCATTTTTTGAAGATGGTAAAAAATATGACCCTTCA

AGTGGAAGTGATACATAATGAGAGGATACAATGGACGATATTTTATACAAAGAAAACTGCCTACCAGGTAATGTAGCAAA

TAGTTTTCAACACAACATATACAGATTAGGTTATATAATCTCTAAAGATATATTAGACCAACAAATGAGCAATCCAGGTA

TTGTTAAAGATGACAATACATTTACTACCGTTCAAATGGTACACCGAATCTATTCACACCTAGACCAAAGACCACAAGTT

AACCCAGGATTAGAACCAATTAAATATGCTTTGAATATAATGGTTGAAGGTTTTGGTTATAAAGTGAAAGATATATTAAG

ATTAAAGTTTAATTGTATGCAACCTCATCCAAATTTCAAAGAAGGTATGTATAACACACCACACATTGATGACGAAGAAA

TGGCACAACATTGGATTTTAATTTACTATCCAATAGATTGTGATGGTGATACTTATTTGTTTAATGAGAAATTTGATAAA

ACAAAGAAACCAGAAAGACTAACTATACATAAACAAATAACACCAAAAGCAAATAGTTGTGTTATGTTTAGAGGAGATAG

ATTTCACGCAAGTGCTAACCCAATGAAAAGTGAAATGAGAATTATATTAAATTGTAATTTTTCTTTATTAGAGAATAAGG

ATGTGTATAATGAAAATAATAGAGATACAAGTAAAGACCCTTTCAAAGGAACTAGTATAGAAGGTAAAGACTAATGGGAA

AATTAGAAGATAAAGTAAATGATATTTTAGGTATTAAGGAAGAAAGTACTCCTGTCGCTGAATTAATGGTGCAAGAGAAA

AAAGTTCCTGTGCCTAGAAAAGAGGATCCTAAAAAGGACGATATAGATAATGATTACAAATATAGTAGAGAAAACTATTA

TAATTTAATTGAAAGAGGACAAGACGCTATTCAAGGTATATTAGATGTTGCTAAAGAGGGACAACATCCAAGAGCATATG

AAGTTGCAGGTGTATTAATTAAAAATGTAGCTGACACCGTTGATAAATTACAAGACTTACAAACTAAATTATCAAAACTA

AAAGAGTTACCTAATAAGACTACTGCTAAGATTCAAAATGCTTTATTTGTTGGGAGTACTACAGACTTGCAAAAGATGTT

GAAAGATAAAAAAATTGTTAAAACAACTTCTGAAAAAATGCAAGACGATTTAGAACCGATTGTAGTAAACGACAAAGAGA

AAAAAGATGATTAATGACGCATATTTAGGAAATCCAAATCTTAAAAAATCAGGTACTAAAACCGAGTTTACGGAAGAACA

AGTAAATGAGTTTCAAAAATGTTCTGAAGATCCAATCTATTTTATTAAAAATTATGTAAAGATTGTATCGCTTGATGAAG

GTTTGGTTCCTTTCAACACTTATAAGTTCCAAGATAAGATGATTGAGACTATGCACAACGAAAGGTTTTCAATCTACAAA

CTACCTAGACAAAGTGGTAAATCTACAACTATTATATCTTACTTATTACATTACGCATTATTTAATCCCAATTCAAGTAT

AGCTATTCTTGCTAATAAATCTTCAACTGCTAGAGATATATTAGGAAGATTACAACTTGCTTATGAAAACTTACCAAAGT

GGTTACAACAAGGTGTTATCAATTGGAACAAAGGTAATATAGAATTAGAGAATGGAAGTAAACTAGTAGCGGCCGCAACT

TCTTCAAGTGCTGTCCGAGGAGGTTCATATAACATTATCTTCCTTGACGAGTTTGCTTTCGTACCTACAACTATTGCCGA

ACAATTTTTTAGTTCCGTTTATCCTACAATTACTTCTGGTAAATCAACTAAAGTAATTATCGTATCAACTCCTCACGGAA

TGAATCAGTTTTATAAATTATGGATAGACGCTGAGAATGGACAAAATGATTATGTACCAATTGAAGTACATTGGTCAGAA

GTACCAGGTAGAGACGCCAAATGGAAAGAAGAAACAATTAGAAATACATCGGAAGCACAATTTGCTAGTGAGTTTGAGTG

TGAATTTTTAGGTAGTATAGATACATTAATTTCAGCTGCCAAAATAAAAGCGACACCGTATATAACACCATTACAAACAA

ATGGCAGATTAAGTGTCTTTGAAAAACCTATTAAAGGAAACACATATCTATGTACGGTTGATGTTGCCCGAGGTTCTTTA

AAAGATTATTCAGCATTTATTGTTTATGATGTAACCAACTTACCTTATAGAATAG

>lcl|3300005521_____Ga0066862_10001059|417910_ __Eastern_Pacific_Ocean_-_ETNP2014F10-02SV255

GGACTTCAACCAATGGTTGGAACGGAAGATGGTCAAGTGCTACTTTGGGGATTTTCTAGTAATAACAACTTGGGACACCA

CGCAACTGCTACCTGGTCAAACACAGGCAGACCGATGATGTGGCAAGCTGGTAATGGTAGATAAAGTATAAATAGAAGTA

TAACAAAAAAAGAAAACGGAGATAAAAACAATGGCAAAAGTAATATATTCAATGACTGCTGGAATTGGTCAAGGTGATGA

TTATACTGCTCCTACAGGAGATACGCCTATTAGTTTAGGAGAGTTAAATGGTAAAGCATACTTCTCTATTGATGATGGTA

ATACTACCATTTCAACTAGTGGTGCAAATGATTCTGTATACGGTGTATCAGTAGTATCAGACGCAGACGAAAAAACAGCA

ATCAAAAATAGTAGCTCTTATGTTGAACAAGGTTTAGATAACCTAGACAACGATTTTATGGCAGGTAAAAATATGATAGA

TTTATTATCAGATGTGGCAGATGACACTTCTGCAACTAAAACTGCTATCGCTGACCATAAAACTGCAAAAGCTAATTTTT

TGACAAATTTAGGATTTTAATTAAACAAGTATTAGGGAAAGTAATATGGCATTATCAATAACAGATTTTAAAGTAACCTG

GAGAGGTGCTTGGAGAGATAAGGAAAGCTACAAGAAAAATGATGTAGTTTACTGGAGAGGTAAATCTTATAGATGTATTG

AAGATACACCTATGAATTATACTATTTCTTCTGAAGCAATGATTAATACTAACTCGTATGGTCAATATCAACCTACGATT

AGAAAAAGAAGCTATAGACCAGATGACAGAAGATACTGGACATTATTACTAGCAGGTAACGATAACATTGAAACTTGGCA

ATATTGGAGACAATATGAAAGAGGCGAAATGGTTAAAGTTGCTGACAAAATTTATCTTTGTTTACAAAGAACAAGATATT

GTAATACTTGGGTAGAAGAACACGATGGAAGACCATCAAAATATTGGGAACTAATTTACATAAACGAAAACAAGTGGTGT

ACAAGAAACGAAGTTGTATCATTTAACAACCGAGCTCCGTTAGGTTGGAGATACAATATGGGAGTTTCACACACAGGTTG

CTCAGACCAATCATATAGAACTTGTACTTTATGTTCAGATGGTTCTGATATGTGGGTTGGTTCTTCTGACAATACCTCAT

CTTCAGGATTAGGAGAAGGTACTGCTGGAAATGACGAACCTGCAAAACATATGTCAACAGGTTTCACATTTACAGATTGG

ATGGCGTCAACAGATAATCAATCTTGGAATATTAATGCAACAGGAAGAATGACTACTCCTGATGGTAAAGCTCCAAGAGT

TATCCAAGTTTCAAAAAATTATAACAGAACATATTGGTTGTTTAACAATGGTGAAGTATATGCTTCAGGTAACAATGGAA

ATTATTCTTTAGGAAATTCAGAAACAACAGATAGACCTTATGCAGTTAGGGTAACGGCAAACGATACGCAAGACTGGCAA

GGTAATACAATTGGAAAAACATATAACCAAACTAGAATGGTTAAAGTAGGATTTTCAGACGAGGCACACGATAGTGGTAC

TACTTCTAATTGGTCATTAGGATCAGATGGAAGTGTATGGGTTTGGGGTTACAATAACAACGGTCAATTAGGACTTGGTA

ATCCTTCAATTAATAACTCAACAGATACTACTGGTGGACCAACTTCAACTGCTTTCTATAGTGCCAATATTCCTAGACCA

GTAAGATTACCACAATCATATTTTGATGGAAGAAGAATTGTAGATATGTGGTCTTCAGGTTCAGAAGAATGTTGGTTCCA

CGCACTTGACGATACAGGTCAACTATGGGCGTGGGGACATAACCAATACGGTGAATTAGGAGTAGGTAACAGAAATGGAA

CTTATTACTATACAAAACCTACAAGAGTTGGAATTAACTGGAACAGATACGGTGGAATAAAATTATACAAATCAACTTGG

TCAAACGGTGGAAACTCTTCTACACACATTTTAGATGGTGAAGGATATATGTGGTTCACAGGTTATACAACTTCAGGCGC

TTGGCCGATAGGTTCTCCAGGTTATACAGATACGCACCATATTGGTTCGTTCAGAAGAGAAGGTCACTTTATAAATGGTG

ATATTGACTTCTTCTGGTGTGGTGGAGATGAAAACAAATGGTTGTATTTAAGACAAAAATCAACAGGTATGCTATGGGTA

CACGATGGTAACTATGGAACTTATGGTGGTCGTGGACAATCAGTAGAAAGTAATGGATACTGGTATTCTTCAGGTGGTCA

CCCAGGAAGTTTCATACATCAAAAAGGTCCTAAATGGGCGGTTAATGTATGTGATGTAGGTATGAGTAGAGCTGATGGTT

CTTATATGTACTCTTTCCCAATGATACTTGATGACGAAGGAATTATATGGGGTGGTGCTCCATATTCAAACGATGAACAC

GGTTTAGGTGGAGACTCAAGTAATAATGACCAATACACTAATGGTGGTCGTAATGACACGCAAGGTGCTATGGAAGACAA

TGAAATGTTTAGAACAAGAAAAAGAATTGTATTCCAACCTGCAGGTGGTCATAGATGGACAGATTTATTCTATTCAGGAA

CTGGTTCTTCAAACATACCAAGAGCTCTTAACCAAAGAGGTCAGGTATACTGGTGTGGATATGATGGTGGATCTTCGGTA

ACTCAACACTATGACTATTATGGTGAAGGTGCTAATAGTAACCAAACTGCTTACTTCTTCCACTTGGGTCCTAGAGACTA

ACATAAATACTATATTATAGACCTGCTTTACTATGGGTCACTATATAACAACTTGGAGTGAAAAATGAAAGACCTAGAAA

CCTTTATTGAAAAGGCACGAAACAACTATGAATCAATGGATTTCATAGTAGATTACAACAACAATAAACTCATCAAAGAA

GTTAAAGGAACTTACTTTTATAATCAATGGTTGGTTATAAATCAGTTAGAACATTTAACTTTAGAAATCACTAAAGACTT

TCCCGAAATAAACATAAGAGAAAAGCTATATGAGGCATTTAAACAAGAATGGCCTTATGAAGCTGATGATATTTCAAAAC

CTTGGGTAGAACCATCAATGATGTATGGTACCGAAGTATGGGTAAATCATTTAAAACCTTTAAAAGATACTCCACACAAA

CTAGTTGCTCAACTATACGCTACTCATAGCGAAATACATAAAAATCAAAAATCTTCTATATTAGTTGACAAGTTAAAAAC

TTTATTTGAAAAATATTACAAAGACCATAAAGAAGAAATGTTAGAGGAAGTTAAAATGTCTTGGGATTTTAAAAGAGGTT

TAGTACAAGATTTAATGGCACACCAAGAACATATGGAAGAGGTCTTACCTAGAATTGCTTTATTCAAAATTGGTGCAAAA

GAAATAATGGAAGATAAATCAGGTATTAATAATATGTCTGCTGGTAATAGAGACGAAACAGAAGATATGAAAGTAAGAGC

AGAATTAATGAAAAATGCGGTTACTATGAGAGAAATGGATGTTGATGATTTGCCAGAAGAATATAAAGATTATGTTAACG

AAGATATAAAGGCAGAACAACAAAAGAAAGATGAACTAGATAAAAAATTTAAAGAAGCACCAAAAAGATGAAGACATTAA

AAGAACTTACTTGGGAACATCATAAAGAAGCTGAACGCCAAGGATTTGTAAAAACAATTATGTCAGGTAAAATAAATCCT

GAAATATATGGTATCTATCTTTTCAATCAACATCAATGTTATAATATGTTAGAAGCGTTAGCAATGTCAGAAGGTATCTT

TGATGATATGCCTGAATTGAGACGAGCACCATCTATCAAAGCAGACTTTGATGAATTGTGGACATATAATTGGAAACCAC

CATTGATGGAATCTACAAGTAAATATTTGGATTATATTAATAAAAATTTAATGGATAATCCAGAAAAAATAGCTGCTCAC

ATTTATGTAAGACATATGGGAGATTTATCTGGTGGTCAAATGATAAGAAAAAAGATACCAGGTCAAGGAAAATATTATCA

ATTTAATATTAGATATGTTGAAGGTAGAAATCAACCATATAAAAACATTAAAGAATTAAAAGAAGCATTAAGAACTAAAG

TGGATAGTTATCAAAAGTATTCAGACCAAAGTACTATATCTGAAAATATTAATAGTGTTGTTTATGAAGCAAGAATATGT

TTTGGATTTGCAACAGATTTATTTAAAGATATGAAAAAATTTATTGAACAAAACGAAAAGAGGTTTGGTGATGGCAATTT

ATAAACGAAGTAGAATATGGCAGATGTTAGAAGAAACTACTAACTATCTTACAGCAGTTTTTGATAGAGAAGGTAAAGAA

ATATTTGAACCAACTATGGAAAAATTTAATCGTCCAAAAGACGGTTGGGTTAATAGAGTATGGGAAACACCAGAAGCGAG

AAGATGTCATTTAGATGTTGTAGACGCAAGAGGAACAAAAGGTTTATATATGTTTCATTGTTGTGTATTTCCAAAACTAA

CACACCCAGGTCCAATATATGGACTTGATGTTATTGCTGGTGCAAAAAAGGTTACAGGTTTCTTCCACGACTTTTCTCCA

CTTGCAAAGAGAGACCATTCAATGGTTGATTGGTTTGTGAAAGAAGCAAGTAATTATAAACCATCTAAAGTACGAGAACT

ACCTGATTGGGCAATGAAAATTTTTAGTCCTGGTATGGTTGCTGCTAGTAATATAACACAAGAAAAAGAATTAAATGCCG

CTTTAAGTTTAGCACAAACTAATTTAGGTGCTTACTTTACATTATTAAGACGAGAAAAAGGAGAAGGAAATATACAAGAA

ATAAAAGACGCACAAAACAGATACGCAAAACATCAAAGAGAAAATCCTCATACGCCTAGAGTAATGAAGTCTTTAGGATT

AAAAGATGAAGATGTTGAAGAATTTTGTACAAACGCATTATTTCCTTATGTTGAATAATGGAACATTTAGATAAATTTAA

ACAGGTCATAGACGATTATAAATCAGATGGAAGATATAGAACTTTTAATGATATTATAAGAACAAGAGGAAAGTATCCTC

ACGCCATTTGGTATTCAAAATACTCAATCAAAAATATTGTCAATTGGTGTTCCAACGATTATCTTGGAATGGGACAACAT

AACTATGTCATAGACTCTATGAAAACAGCACTTGAAACGAGCGGAGCGGGTGCTGGAGGGACAAGAAACATATCAGGCAC

TACTCACTATCATAATGCTCTGGAACGAGAACTAGCGTCTCTCCATAAGAAAGAAAAAGCATTATTATTTACTTCTGCTT

ATAATGCCAATCAAACAACTTTAGAAACAATGGGTAAGGTTATGCCTGATTTATTGTTTATATCAGACGCACAAAATCAT

TCTTCTATCATACAAGGTTTACGCCATAGTAGATGTAGAAAAGAAATATTTAAACATAATGATTTAGATGATTTAGAAAG

TATTTTAAAATCTGAACCAGGTCCTAAATGTGTAGTATTTGAAAGTGTATATTCTATGGACGGAGATATTGCTCCTGTAA

AAGAAATAGCTGACTTATGTAAAAAGTATAATGCAATTTCTTATATTGATGAAGTACACGCTGTTGGTCTTTATGGAAAA

GAAGGTGCTGGAATATGTGAAAGAGATAATGTAGAAGTTGATATAATAAATGGAACATTAGCAAAGGCGTTCGGTGTACA

AGGTGGATACATCGCAGGAAAGAGAGAGTTTATTGACACAATAAGAAGTATGGCTAGTGCTTTTATTTTTACAACTTCTG

TAAGTCCAGTTATTTGTGCTGGTGCTTTAACGAGTGTTAAGTATGTTAGAGACCATCCTGAATTAAGAGATAAGATACAC

GAAAGAGCAAACAAAACAAAAGAAGAACTTGAAAGACAAGGAATAGAAGTTATGAAAAATGATAGTCATATTGTTCCTGT

TATTATTGGAGAAGCTAAAAGATGTAAAGCAGTATCAGATGAATTACTTTACAAAGAAGGTATCTATGTACAACCTATCA

ATTGGCCAACGGTTGCTGTAGGTACTGAAAGATTAAGATTTACTCCAACTCCATTTCATACAGATAATTTGATATTTGAT

ATGGTAGTTAAAGTCAAAGCTGCTATTAAAAGATGTGGAAAGAAACTGAATTATGATTGATAAAATTATAGCTGATGGTG

GAGATGGATTAGATGTCCTAATATATTGTCTAAAACACGAACCTTTTATACAAGGAATTATATTATTTGGTCTGTTTTTA

GCGATATTTTCTTGGTACTATGATAATAAAGTAGATGATAAGGCCGTTTGGTCAAATAACGACCATCTATAAATTATAAA

TATAGCAAAGAATTAGAAGGAAATAACTATGGCTCAACCTAATACAAGACAGACATTAATCTCTTATGCTAAAAGAGCAT

TGGGGCATCCTGTTATAGAGATAAATGTTGATGATGACCAAATAGATGATAGAGTAGATGAGGCGCTACAATATTGGCAA

CAATATCACTATGATGGTATCAAAAGAACTTATTTAAAATGGCAATATACACAAGCAGAAAAAAATAGAATCTTAACTAG

TAATAGTGAAGCAGGAACAAAGAATTCTGTAACCTCTACTTGGAAAGAAGATAACAATTATATTGTTGTTCCAGAAACCG

TATTTTCGGTTACAAATATATTTCCTTTTTCAAACAAAGGTAATTTAAACTTATTTGATGTTAGATACCAATTAAGATTA

AATGACTTATACGATTTCTCATCAACTTCTGTTATTAACTATGATGTAGTTATGAGACAATTAGATTTCCTAGACCACAT

ATTAGTTGGTGAAAAACCATTAAGATTTAACCAACACGATAATAGATTATACATTGATATGGATTGGGAAAACGATTTAA

TGATAGATGAATATATTGTTATTGAATGCTACAGAAAAATGGATCCAGACACATATACAGATGTCTATAATGATATTTGG

TTAAAGAAATATACAACTGCACTAGTTAAAAAACAATGGGGTGCTAATCTATCAAAATTTGCTGGTGTTGCTATGATAGG

TGGTGTAACCTTAAATGGTGAACAAATCTATACACAGGCATTAGCAGATATAGAGAAGTTAGAGGAAGAAATAAAATCTC

TACAAGAACACCAAGCACTAATGATAGGATAAAAATAAAATGGCCGTTAATCATTATTTTCAAGGCGGCGATGGCATAGG

TAGTCAAAGTGAGAAAAGATTAATAGAAGATTTAATCGTAGAGAATTTAAAAATCTATGGACACGCTGTTTATTATTTAC

CGAGAACTCTAGTTAATAGAGATTTAATTCTTGGTGAGGATTCTGCGTCTAGGTTTGACGACTCGTATCTAGTAGAAATG

TATTTTGACACACCACAAGGGTTTGCTGGTGAAGAAGAAATAATTAGTAAGTTTGGATTAGAAGTAAGAGACGATACAAC

TTTCGTTATTGCTAAAAGAAGATTCCAAGAACAAGTAGATGACCCAGCAAACCTAATGGTGGATGGCAGACCTAATGAAG

GTGATGTTATTTACTATCCTTTAATGAATAGGTTTTTTGAAATTGCGTTTGTTGAAGACCAGGAACCTTTCTTTCAATTA

GGAAATTTACCTGTCTATAAATTAAGATGTAAAACATTTGAATACTCTAGTGAAGAATTTAATACAGGTCACGCTGACAT

TGACCAAGCTGATGATAGAAAATCACTTGATACATCTTTGGCACACCAGTTTAGACTTGAAGATGGTACATTAAATCAAT

CTTCTTATAGTGGTTTCTTACAATTAGAAACAGGAGATAAACACGGTAATCCTTGTTATTTAATTAATGAAGATTGGGAC

GACACTACAACTGATGGAGACGCTGCTGAAAGTGTACAAACAAAATCTGCTTATGCTGATAATTTAGATTTAGATTCAGC

TGCTGGTTTTGATACTGCAACGGTTAATGATGATATACTTGACTTCACAGAAAACAATCCATTTGGAGAAGTTAAATAAT

GGAAAGAGATAGACATAAACAACTAGTAGAACATACTAATAGAATTAATAAAGAAAAAAAAACTTTAGAGTTATCTAAAA

CTTTAAGAAAAGAAGTTGAGATAGGTGCTACAGGCACACAAAAATATAGATTTAAAAAAGGACCTAATAAAGGTAAGGTA

GTATAATGTTTGGAACTCATTTTTATAACGAAGGTATGAGAAGATTGACTATTGCTTTTGGTCAAATCTTTAATAAGATT

GTTGTACAAACAAAAGACGCAAATGGTTCAGTAGTTAAAAGATTTACGGTGCCATTAGCATATGCGCCAAAAGAAAAATT

TATTGTTAGATTAACTCAACAAGGTGATTTAACAGATAAACAATTTGCAACGGTACTACCTCGTATGGGATTTGAAATAG

AAGGTATAGAATATGACCCTAGTAGAAAGTTAAATAAATTACAAAAATTTAGAAAACCAAACACAGATGGTTCTTCTACG

GATCAAGCTAATAAAATGGACTTTAACTATACTCCAGTTCCATATAATATAACATATAAATTGTTTATATTTACAGCAAC

TGCTGAAAATGGTTTACAAATTTTAGAACAAATAGTACCGTACTTTCAACCAGATTATACGGTTACAATTAATATGGTTC

CTGATTTAGGAATTAAGCGTGATGTTCCAATTGTAATTGGAGACATACAATACGAAGATAGTTATAGTGGAGATTTTGAA

ACTAGAAGAGCAGTAATATATACTATGACCTTTACTGCTAAAACTTATCTATACGGACCTTCTACAACAGCAGGTGTTGT

TAGAAAAGTACAAACAGATTTAGGAACTGATTCAGTCAGTAAGGCAAGAGAAGAAAGAATAGTAATTACTCCTGACCCTA

CAACAGCAAAACCTGGTGATGATTTTGGATTTACAACAACTATATCATTTTTTGAAGATGGTAAAAAATATGACCCTTCA

AGTGGAAGTGATACATAATGAGAGGATACAATGGACGATATTTTATACAAAGAAAACTGCCTACCAGGTAATGTAGCAAA

TAGTTTTCAACACAACATATACAGATTAGGTTATATAATCTCTAAAGATATATTAGACCAACAAATGAGCAATCCAGGTA

TTGTTAAAGATGACAATACATTTACTACCGTTCAAATGGTACACCGAATCTATTCACACCTAGACCAAAGACCACAAGTT

AACCCAGGATTAGAACCAATTAAATATGCTTTGAATATAATGGTTGAAGGTTTTGGTTATAAAGTGAAAGATATATTAAG

ATTAAAGTTTAATTGTATGCAACCTCATCCAAATTTCAAAGAAGGTATGTATAACACACCACACATTGATGACGAAGAAA

TGGCACAACATTGGATTTTAATTTACTATCCAATAGATTGTGATGGTGATACTTATTTGTTTAATGAGAAATTTGATAAA

ACAAAGAAACCAGAAAGACTAACTATACATAAACAAATAACACCAAAAGCAAATAGTTGTGTTATGTTTAGAGGAGATAG

ATTTCACGCAAGTGCTAACCCAATGAAAAGTGAAATGAGAATTATATTAAATTGTAATTTTTCTTTATTAGAGAATAAGG

ATGTGTATAATGAAAATAATAGAGATACAAGTAAAGACCCTTTCAAAGGAACTAGTATAGAAGGTAAAGACTAATGGGAA

AATTAGAAGATAAAGTAAATGATATTTTAGGTATTAAGGAAGAAAGTACTCCTGTCGCTGAATTAATGGTGCAAGAGAAA

AAAGTTCCTGTGCCTAGAAAAGAGGATCCTAAAAAGGACGATATAGATAATGATTACAAATATAGTAGAGAAAACTATTA

TAATTTAATTGAAAGAGGACAAGACGCTATTCAAGGTATATTAGATGTTGCTAAAGAGGGACAACATCCAAGAGCATATG

AAGTTGCAGGTGTATTAATTAAAAATGTAGCTGACACCGTTGATAAATTACAAGACTTACAAACTAAATTATCAAAACTA

AAAGAGTTACCTAATAAGACTACTGCTAAGATTCAAAATGCTTTATTTGTTGGGAGTACTACAGACTTGCAAAAGATGTT

GAAAGATAAAAAAATTGTTAAAACAACTTCTGAAAAAATGCAAGACGATTTAGAACCGATTGTAGTAAACGACAAAGAGA

AAAAAGATGATTAATGACGCATATTTAGGAAATCCAAATCTTAAAAAATCAGGTACTAAAACCGAGTTTACGGAAGAACA

AGTAAATGAGTTTCAAAAATGTTCTGAAGATCCAATCTATTTTATTAAAAATTATGTAAAGATTGTATCGCTTGATGAAG

GTTTGGTTCCTTTCAACACTTATAAGTTCCAAGATAAGATGATTGAGACTATGCACAACGAAAGGTTTTCAATCTACAAA

CTACCTAGACAAAGTGGTAAATCTACAACTATTATATCTTACTTATTACATTACGCATTATTTAATCCCAATTCAAGTAT

AGCTATTCTTGCTAATAAATCTTCAACTGCTAGAGATATATTAGGAAGATTACAACTTGCTTATGAAAACTTACCAAAGT

GGTTACAACAAGGTGTTATCAATTGGAACAAAGGTAATATAGAATTAGAGAATGGAAGTAAACTAGTAGCGGCCGCAACT

TCTTCAAGTGCTGTCCGAGGAGGTTCATATAACATTATCTTCCTTGACGAGTTTGCTTTCGTACCTACAACTATTGCCGA

ACAATTTTTTAGTTCCGTTTATCCTACAATTACTTCTGGTAAATCAACTAAAGTAATTATCGTATCAACTCCTCACGGAA

TGAATCAGTTTTATAAATTATGGATAGACGCTGAGAATGGACAAAATGATTATGTACCAATTGAAGTACATTGGTCAGAA

GTACCAGGTAGAGACGCCAAATGGAAAGAAGAAACAATTAGAAATACATCGGAAGCACAATTTGCTAGTGAGTTTGAGTG

TGAATTTTTAGGTAGTATAGATACATTAATTTCAGCTGCCAAAATAAAAGCGACACCGTATATAACACCATTACAAACAA

ATGGCAGATTAAGTGTCTTTGAAAAACCTATTAAAGGAAACACATATCTATGTACGGTTGATGTTGCCCGAGGTTCTTTA

AAAGATTATTCAGCATTTATTGTTTATGATGTAACCAACTTACCTTATAGAATAG

>lcl|3300005603_____Ga0066853_10000534|149210_ __Eastern_Pacific_Ocean_-_ETNP201406SV61

ATTATCAGCGAGTGATAGTATAAATTTATTCGGAATTAAAACAAGTTGTGTATATGGAGTTAAAGTTTATCCACAAGTAC

CTAATTCAGGAATCTTTAATGTTAAATCATCTAGTTCATCTACTAAATTAAATTTCTTTTTAGCACCAAGTGAAATTGAA

CATACTTATGTTAGTGGTGGTACAGTTAAATCAGCAACACCAACTAGTGTTGGTAGTGCAACTGAAATTAATAGTGCTAC

TTATGATAATGTAACAGGACTTATTACGATAGGATCACCTGGTCACGGTTTAATAGTTAATGATTTAGTACAAGTACAAG

GAATGCAATTCACTTGTTCAACAGGTAGTAAAGCATATCCTGATGATATATTAAGTTCAGGAATATTTAAAGTTTATGAT

GTGCCTGACGTAGATACATATATCTTTGGTGTTGATAAATCAGCAATTGCTCATACTTATGTAATTGGTGGTACTTCACA

AAAAGTTACCATCGCTACAAGTTCTAGTGTTAACGTTTCAGGTTTTGTTTTCAACAGAACAGCTGCTGCTCAACAAGGAC

AAAGGGGTCCTTTGATTGCAGTTAAATCAGGTACAACAACTTTAAATGCTGTTGATATGATAGCTTTAGCAAGTAATGTT

AAATTCCCTAATGATAATACATTTTATAGAGTAGGGTTAGTATCAGAAGAAGATACGAGTGCTGGAACAGCAGTAATAAG

ATTAACTCAAAATATTGGTTTGAGTAAAGCCAAAAATGAGGATACAGTAAATAACATAACAGAATTATATTCAAATATTC

GTTTAACAGGTCACGATTTCTTAAATATTGGTACTGGTGATTTTACTACAACTAATTATCCAAATGTACCTTTACAAACA

AACGACCAAGCAGATGAAGTAACTGAAGTTAACGGTGGTCGTGTATATTGGGTATCAACTGACCAAACTGGTGACTTTAG

AGTTGGTGATTTATTCAAAATTGAACAAGCAACTGGTAGTGCAACATTAAACGCAGACGCATTTAACCTTTCAGGATTAA

GTGAATTAAAACTTGGTTCTATTGGTGCAGAATTAGGTGCTGCCATAAATGAATTTAGTACAGACGCAACTTTAGGCGGT

AATTCAAATACAGCTATACCTACTGAAAATGCTGTTGTCGGTTATATGACAAGAGATAAAGCAGGTACAGGTGCGTGGGT

TCCACCAACAGGAACATCATCACAAAGACCTGTAGGCGGTGAATTATTTGCAGGTGCTTTAAGATACAATTCTTCAATAA

TTTCTTGGGAAGGTTATAATGGAACAAGTTGGACAGGTCTAGCTGGAGGAACTCCTTGGACAACTCTAGTTGGAGATGGT

TCAACTGTACCTACAGCAATAGGTGGACAAAGATTATTAATAGATACAAGTTTATTTGCAATGACAGTTAAATTGCCTGC

TAGTCCACTAGTAGGAGATTCAATTGTATTTTTAGATTTAAACGGATCATTTCAATTAAGACATTTAACTGTTGATAGAA

ATGGTCAAGATATTATGAATTTACAACAAGATATGATTGCTGATATCAACCACGCAGGATTCACTTTAGTTTATACTGGA

TCAACAAATGGTTGGAAATTAGTAGAAGTAGCGTAATAAATAAATATAGAAGAGAAATATAAATGAGCAAATTAACAGAT

TTTACAGTTACATCCGCTGAGAAAGATGACTTTTATGGATTCCATAGAGTTGCTCCTTCTCAAACGATACATAGAACCCT

TACCTTAATTACTGGTAATGAAAGTGTATATGAATATACATTAGGAACAGGTTGGGATATTTCTACAATGGCATATTCAA

CATCTTATTACATAGGGTTTCACGATTCAAATCCATTAAACACAACGTTTAGTACTGATGGAACAAAAATGTTTGTTATG

GGCAATGCAGATAAACACGTTGATGAATATATATTGACTACAGCTTTTGATATTTCTACAGCAAGTTGGAGAACACATAA

AGATGTATCTGCTCAAGATGATAATCCAAGGGCAGTAAGATTTAATCCAGACGGAACTAAAATGTATGTTGTGGGTAGAG

ATGGAGTACCAAGTGCAGGTATAGCTGCTTGTAATATTAATGAATATGCATTAAGTACAGCTTGGGATATAACTACAGCA

ACTTATACAGATTTATTTTCTTGTCTTGCTCAAGATACTGCTATTAGTGATATGCGATTTGGTGATAATGGAGATTTATT

AATTGTTCTTGGCGATGAAGGTAATGATGTTAATGAATATGAATTAACTACAGCGTATGATATTTCTACAGCAACTTTTG

TAGATTCTTTTTCTATCGGTGCTCAAGAAACAAGTCCAGCTGGGTTAGGTTTTAATACAGATGGAACAAGAATGTATATT

GCAGGAACAGATGGAGATGATGTTATACAATATCCATTAGTAACAGGTTTTGATGTTTCAACTACACAAGCACTTACACA

CGAAGTTGGTTTAACTAATGCTCCTTCAGCACCAACAATGAACCCACGTGGTTTAACTTTTAATGCTGATGGAACAAAAC

TGTATGTTATAGGAACTGCTGGTACATTAATGATTGATGGTGGCGATGATGAATTACCATATAGTCACGTAGCTAGAAGT

CAAAACACTATGACATTTCTTGAAGGAAATACGTATGTGTTTGATGTTTCTCAATCTGCTTTAGTCGGACACTCATTAAA

ATTTTCAACAACAGTTGATGGTACACATAAAGCAGGTGGAACTGAATATTTAACAGGAGTAAGTTCATCTGGAACTCCTG

GAACTCCTGGAGCTACAACAACAATTATTGTTCCAAGTAAAACACCAAGTATAGAACCAGGAAGTGCTGTAGATAAGTTG

TATTATTATAATGGTGGTCATCCAAGTCAAGGTGGTGAAATTTTTACACCTGAATGGAAAGGCAATTTACAGATTACTTA

CACAAATGGATATGATGATATTGACACTAGACATAAAACTAAACATCAAGAAGATATATTTGAGGATAGTGTACTATGGA

AAAGAGGGTTGGTATTTACGGTAGTCAACGGAAACCTAACCATAGAAATGGGCTAAAAAAATATTTGAATTAACTAAAAT

GGAAGAAGAGAACTATTATAAATATAAATAAGGATCAAGAGAATTATGGCAACTATAAATTTAGGAAGAATTAAACCAGT

ATTTCAAGGGGCATATAATGGCGCTACTGCTTATGTAGTGGACGACATTGCTACCTATGGAGGCGAAACTTTTATTTGCA

TTTTAGCTTCAACTGGAAACGCAACTTCAAATGCAACCTATTGGTCTAAAATAGCCAAAAAAGGTGATGACGTAACACAA

CTTACTACCCACGGCGATATACTGTTTAGGGATGCAAGTGGTGTACAAAGATTAGCGGCAGGCACAGCAAATCAAATGTT

AGTAACTAAAGGTGCTAGTGCTGATCCTGTTTGGGGTTCTTCAACTTCAATTTTATGGGAAACTAAAACAGCTAATTTTA

CTGCTGTTCACGGCGGTGCATATATATGCAATACAACAGACGGTGTATTTACAATGACACTACCTGCTTCACCAGTAGAT

AACGACTTTGTTATTATCAATGATGGTATGGGAGTTTTTGATACAAAAAATCTTACAGTTGATAGAAACGGAAATAATAT

AGCAGGAAGTGCTACAGATTTAATAGTAGATAAAAAGTATGCTAATTTCAGATTAACATTTAAAACAATACCAGATGTAA

CTTCATCTTTTATTGGTTGGATAATTTCATAATGAAAGATATAAACTGTATAAATAGTATTACAAACAAAATTTTAAGGG

AGAACATTTAATGAGTTCATTAACAACACTTTTAAGCGGCGGTAGTGCTGGTGCAATAGACCACAGAAAAGAAAGTCTAC

CATTATACGGATTTTGGGGAGATAACTCCGACGGTAATCACCATATAAATTACAGAATCTTTGATTCTGGTCATAATAAC

GTAGGGTCTCCTTGGGGTGCTGTATGTAACTCAACAACAAATTATAGATTTGGGATGATGGGTGACGCTTCTTTTACTTA

TTCACACAATGACCACGGTACAAACGTATCACACGCAGATTTAACTTCACAACAATATGACTCTTGGACTAATTGGAATA

AATCATTATACCAATGTGACCAATATCCACACGCACAATATTATTCATCTTCAAGAGATGGATTTGTTTCTTGGCATAGT

TTCCACGAATACACATCTTCTTTTGAATATCAAGATGGTTGGACAAAATTAAATATGGTTCTTCCTGAAGGTATTAGACC

TAGACGTATGTTTGTTAATAGACGATTTACGTTAAGAGAAAGATATCCAGGTAATCACGGTGCTCCAAATATAGACACGT

ATGATTATACTTCTAACTTACTAGAAACAACTCAAACATATTCAACTGGTACTGGATACAATGAGAAAACAAAAACTTTA

GTTATGGTTCACTCTGGTGACGAAAGTGGAAATACTTCAAAAACTATTCACATTTTCAAATCTAGTAAATGTTTAAATAA

AATAGACAGAATTAAAGAATTCTTTGATAACTTAACTTCAACTGAATACTTTACTGACACTTGGACTAATCAATGTACTA

AAGATTGGTGCGTTGTTGTTGGTAATAATGATTTCGTTGGATTCGGAAACAAACAAAGTAATAGTAAAAGATACGGTGTA

TTTGATTGTTCAGTCAAAGGCGGAACTGCTCACCAAACTGGTGCAAGTAGACAATGGTCAACTTGGCAAGATTTTACAGG

ATCAACAACTACATCTTACGGTGCTAATAACGGACACCAATACTACTGTAAATTTATGACGACTTGGGATGGAACTTGGG

GAATGATTTATTCTTCATATTATTACTATGGTGTTGGTATCAATGGTTTCTGTATGAGTATAGAAAATCCTAAGAAATTT

ATTAATATAAATCAAACTAAATCAAGTAGAGCCAATCCTTACTTTGCTTGGGGACGTACAGGTTTCCACGGAGGTTGGTC

AGACAACTGCGACGGAACTTCTCATAGAACTTACTCTTGGTCTTTTGATCCTACGGCAACAGATGAAACCGAAGACACAC

TTGTTTATCAAGGTGGTTCTTCAGGAGATGAAATTATACCTAATTCTAACGCACACGTTGGAACTACAGTAACTAATAAA

ACTGGAAATTACGGTTTACACGCTTGCAGAACTTGGCTAACTGGAGGTTTCCATTCAACAAACTATCCACTATTAATGCA

AATTGACTGGTGGGGAAATTATGGAAACAATGATTCTACTTACGGTGGAAAATACGGAACATAGGAGATTATAGAAATGG

CAAGAACATATTACTTTACACTAGCAGGTGAACCTTTTTCACCAAATGCTGAAACTGGAGATGACGCAGTAGCAAAAGGA

AACGCAATCAAAGTTGATGACGTTCCTGAAGGAATTGAAGCGTGGAGATTATCAATTAATCCTGCAACAAAAGAATTAAC

AATCGTTGGCGGAGCTGACGGTGATGAAGCGGCTGCTGATACTGAAAGAGAAACAAAAGCTAATGACGAAGCAGCTGTAC

TAGCTAAAAAAGCAGAAGACTTATTAAAAGCAGAAGTTGCAGAAACTAAAAGACTTCAAGACGCTGGTCTAGCTTAATTT

ATGTAGTGATTTTATTATGATAAGAATTTTATTATGTATGACATCAAAGAATTAACCAAAGATATACACCAAAACGCAGA

AAGACAAGAGTTTGTCAAAACTCTTATGTCAGGTTCTATTGAACCTAATCTTTACGCAACCTATCTTTACAATCAATTAC

AATGTTATGCTGTATTAGAAAAATATGGAATAGAAAATTCTCTATTTCGTACAACTCCTAATTTACCTAGAGCAGAACAT

ATACATTATGATTTTAAAGCATTATGGACAAGTGAAGGCAAACCAACTATAACTCAAAGTACAAAAGATTATGTTGCTCA

TATTGAAACAATCAAAGAAGACGCAGAAAAATTATACGCTCATATCTATACTAGATATTTAGGAGATATATCTGGTGGTC

AAATGATAATGAGAAAAACACCAGGACCTAATCGTTATTACAAGTTTAAACATAAAGAAATAAAAGAGTATAAACGAATA

GTAAGAGAAATGATAAACAGTTATTTAAATGTTTATAAACTTAATATTCTAAATGAAGCTAAATTTTGTTTTGCGTCTGC

TACAAAATTATTCCAAGAGATGAAAGAGATTGAAGATATGTATTACAAACCTTTAATTTTAACTAACGAGGTTAAAGATG

ATTTGGGAACGACTAATTAAATTAGAAAAAGATATAATCGCTATACTTGATAAGCGTTGTAAAGAATACAACGAAGATGG

TATGGATAGATTTAATAATGATACTTGGGTTAACCGTACTTGGTCTAATATGAGTATAAGACGTGCTCACGTAGACGTAG

TGGATGCTAGAGAAACAAAAGGTCTTTGGATGGCACACATATGTTTATTTCCAATGTTAACTAATGGTGGTCCAATTTAT

GGTTTTGATGTTATTGCAGGTAAGAATAAAGTTACAGGTGCTTTCCACGATTATAGTCCGCTATTATTAAAAGAACATCC

CTTAACAAAGTATTTTATAGAAGAAACAAAATGGTATAAACCATCTAAAGAAAGAGAATTGCCTGATTGGGCAAAGGAAA

TCTTTAGTCCAGGTATGATTGCCGCTGGTAATATATCAGATGAAAAAGAATTAAACCAAATTTGTACTCTTGCTACATCT

AATTTAGAAAATTATCTTGACAAAATTGGTCATTATAATAGCGATTCAAAGGAAGAAGATGTAATAAGAGCACAAAATTT

TTATTGCGAACACCAACAACAAAATCCACACACCCCTAGAGTAATGAAAACTCTTGGACTGCCTGAAGATGATATAAAAG

TATTCTGTACTGATAATTTGTTTCCGAAGATATAATTGTTATTATAAATATACAATAAAGGAACCAGTATGGCAGAACCA

GCATCCAGAGAAACAGTAAAACAATACGCTTTAAGAGCATTAGGTAAACCAGTAATTGAAATCAACGTTGATGACGACCA

ACTGGAAGATAGACTTGATGAAGCATTACAATATTTTGCTCAATACCACTATGATGGTGTTAAAAGAACCTATTTAAAAT

ACAAGTATACAGCGGCAGATAAAGCTAGAATTTTAGCAGATAGTACTGAAACTGAATCTAAAACGTATGGTGATTCTTCT

GTAGTAAATACAGAATGGAAAGAAGGCAATCAGTATATTGTATGTCCTGAATCTGTTATATCTGTAATTAACATTTTTCC

ATTTTCAAATAAAGGTAATTTAAATTTATTTGATGTTAGATATCAATTAAGATTAAATGACCTATATGATTTTTCTTCAA

CGTCTGTTATTAACTATGATGTTGTATTAAGACATTTAGATTTTTTAGACCATATATTAGTCGGAGAAAAACCTTATAGA

TTTAACCAATTAGATAATAGACTTTATGTTGATATGGATTGGAAAAATGATTTACAAGTAGATGAATTTCTTGTAATAGA

ATGCTGGAGAAAATTAGACCCTAACACATATACAGATGTCTTTAATGATATTTGGTTAAAAAGATACGTAACTGCTTTAT

TTAAAAAACAATGGGGAGCCAATTTAAGTAAGTTTGATGGTGTTGCAATGCTTGGTGGAGTTACATTAAACGGTAAACAA

ATTTATAGTGAAGCACTAGAAGATTTGGATAAATTGGAAATAAAATTAAGAAGCGAGTTTGAAGAACCGCAACCTTTTAT

GATAGGATAATGCTATGCCAGTTAATCATTACTTTTCAGGTGGAAAAGGCATAGGTAATGCTGCCGAAAAAAGACTACAC

GAAGATATAATAGTTGAAGGTCTTAAAATTTACGGTCAGGATGTCTATTACTTACCACGAACATTAGTCAATAAAGATTT

AATACTAGGAGAAGATGTATCTAGTAGATTTGATGATTCTTATTTGATAGAAATGTATTTTGAAAATAATACAGGATTTG

CTGGTGAACAAGAAATCATAAGTAAGTTTGGATTAGAAATTCGTGATGATACATCATTAATGGTTTCAAAAAGAAGTTGG

ACAAATTTAGTTGGTAATAAGGCAACACAGGTTGGTTCTTCTCTATCAATTACAGGAAGACCAAACGAAGGTGATATTAT

ATATGTGCCTTTGATGAAATCTTTTTTTGAAATTTTATTTGTAGAAGACCAAGAACCATTTTTCCAATTAGGCAATCTGC

CAGTTTATAAATTAAAAGTAACTCGTTGGGAGTATGCAAGTGAAAAACTTGATACTGGTTTATCTACTATTGACCAACAC

GAAGATACACATACACTAGACCAATTAGCATATAAATTTACTTTAGAATATGGACAAGAAGTTATGACAGGTGCAGGTTC

AGTACAATTAGAAAGTTACCACGATTATTCAACTGGTCAACCAGCACTTTTAATGAACGAAGATTTTACAGAGTCTAATA

TACAAACACAATCTCCATATGCAGATAATTTAGACTTGAATAAAGAGGCAGGATATGATACAGTATCAACAGCGGATGAT

ATACTTGACTTTACAGAAAGAAATCCATTCGGGGAAATTGACGAGTAGACTATATGTTCGGAACACATTTTTATAATCAA

AGTTTAAGAAGACTAACTATTGCATTTGGACAGATTTTTAATAATATAATTATTCAACAAAAATCTGGTACAGGTGCTAT

TACTAAAAGAATACGTGTGCCTTTAGCATACGCTCCTAAAGAAAAGTTTATAGCCAGAATAGACCAACAAGCAAGTTTAG

AAAAAGGTAAAACGTTTGCTATTGTATTACCTAGAATGGGATTTGAATTAACAGGTTTAAAGTATGACGCTACTAGAAAA

CTAAACAAACTTCAAAAAACAGTTAGAGTTAAAACTTCTGATTCTACTATACATAATTTTAATTATTCACCAGTACCCTA

TGATATAAGTTTTAGTCTTTATTCTTTTACTGCTACAGCAGAAAATGGACTACAGATAATTGAACAAATATTACCATATT

TTGCACCAGACTATACAGTTACTATTAATGCAATACCAGAATTAAATATTAAAAGGGACGTGCCTATTGTTTTAGATACT

GTAAATTATGAAGATACTTATGATGGTGAATTTAATAAGCGTAGAGCAGTTATATATACTTTAGAGTTTACTGCTAAAAC

TTACTTATATGGACCTATGGCACAAAGTAAAGTTATTAGAAAATCACAATCAGATTTAGGAACATCTACGGATGCTCCTT

TATCAAGAGAAGAAAGAATTATAGTAATACCAAATCCTGAAAGTGCTAATGCAGATGATGATTTTGGATTTACAACAAAG

ATTAGTTTCTTTGATGATACAAAGAAATATAATCCAGTAACAGGAGAAGATGAATAATGCCTAAATTGGAAGATAGTGTA

AATGAGATATTGGGATTAGAAGGAAATAATAAAGTTGTACCAGAGAACCTTGAACCACAAAAAGGTTTTCAACCACCTGT

TCCTAGAAAGAATGGAGAAGTTCCTTTAAAAGTTGAAAAGGATATTAATACTGATTATGATTACAGTAGAGAAAGTTATT

ATAGTATAATAGAAAAGGGACAAGAAGCAATACAAGGCATATTAGATATTGCAAAAGAAGGACAACACCCTAGAGCATAT

GAAGTTGTTGGTCAATTGATAGGACAAGTTGGTACTACAGTTGATAAACTACAAGATTTACAAAAGAAATTTAAAGACTT

AAAAGAACTACCTGGAAGAACAAATGCAAATATTAAAAATGCATTGTTTGTAGGGTCAACTGCTGAATTACAGAAGATGT

TAAATAAGCAAACTATGGAAACAAAAATGGAAAAGAAAAATGAAAATGAAACTATTGACGGCAAATCAAAAGATACCGAA

TAAAATTCCTATCCTACTAAAAGACTTAATCTATATTAAGTCAATGACACCACTAAAAGAATTATTAGATGGTGAAGAAT

TACAAAATCCAATAGAAGTAAAAGAACACGTTGTATCAGAAGTACCTAGATACGGTGCAATGGGTATACCCTATATAGAA

AGAGAATATAGTGTGTGGAGAGGTAGTCAACGAGTGCAGGCAGCTAAACAATTAGGGTATACACATATAGAAGGAGTGAT

AGTTAAGTGAAACATTTAGAAGAATTTACAAAAATAATAAATGAATATAAAGAAGATGGAAGATACCGAGTCTTTAATGA

TATAGTTAGGACTAGAGGAAACTTTCCTCACGCTATTTGGTATTCAAAATACTCAATTAAAAAAATAGTTAATTGGTGTT

CTAACGATTATTTAGGTATGGGACAACACTCTTATGTTATAGACTCAATGAAAACAGCATTAGAATCAAGTGGTGCTGGG

GCAGGAGGTACAAGAAACATATCTGGTTCTACTCACTATCATAAAGCGTTAGAAAATGAATTAGCAGATTTTCATAAAAA

AGAAAAGGCATTATTATTTACTTCAGCATATAATGCTAATCAAACAACTTTAGAAACTTTAGGAAAAATTATACCTGACT

TATTGTATATATCAGACTCATTAAATCACTCTTCTCTTATACAAGGCATTAGGCATAGTAGATGTAAGAAAGAAATATTT

AAACATAATGATGTAGAAGATTTAGAAAGAATTTTAAAATCATACGAAGGTCCAAAATGTGTAGTATTTGAAAGTGTATA

TTCTATGGACGGAGATATTGGACCAGTAAAAGAAATAGTAGAACTAGCTAAAAAATATAATGCAATAACATTTTTAGATG

AAGTACACGCTGTTGGTCTTTATGGACAAGAAGGTGCTGGAATATGTGAAAGAGATAATGTAGAAGTTGATATAATAAAT

GGAACATTAGCAAAGGCGTTCGGTGTACAAGGTGGATACATTGCAGGAAAAAAAGATTTTATTGACGCCATAAGAAGTTT

GGCAAGTGCTTTTATATTTACAACTAGTTTAAGTCCAGTTATTTGTGCTGGTGCTTTAACAAGTATTAAATATGTTAGAG

ACCATCCTGAATTAAGAGAACAAATACACGAAAGAGCAAATAAAACTAAAGAAGAACTTGCTAGACAAGGAATAGAAGTT

ATGAAAAATGATAGTCATATTGTTCCTGTAATTATTGGGGATGCTAAAAAATGTAAATCAATATCAGATGAACTTTTATA

TAAAGAAGGTATCTATGTACAACCTATTAATTGGCCGACTGTTCCTGTAGGTACTGAAAGATTAAGATTTACTCCTACAC

CATTTCATACAGACGCATTAATCTTTGATATGGTAGTAAAACTAAAAGTGGCAATGAAAAAATGTGGTGGTAGAAATGCA

ATACAAAGTAATGCCTAAACATAAAGAATATATTTTACCAACAACTAGTTTAATAGGAGGTTGGTATATTCCTTCTGGTA

TTTGTGATGGACTTATAAACTTATTTAAAGATAATAAACAAGCACAAAAACCAGGTGTTGTAGGTTTCACTTCAAAAATT

AATAAAGAAGTAAAAGATTCTATAGATATTGGATTAGATCCAAATTGGGAAGAACCAAGGTTTATGAAATATAAAAATGC

GTTGAAAGAATGTGTTGGTCTATACGAAGAGAAATATCCTGAAGTTAAAGAGTTTGAAAGATATGGAATGGTTGAAGGAG

GAAATTTACAATACTATCCACCAGGTGGAGGTTATTTTACTAAGCATTGTGAAAGAAACTCTAGGCACGAAAACCGTTGT

CTTGTTTGGCTGACTTATTTAAATAATGTTCCTAACGGTGGTACACATTTTAAATATCAAAATGCAACAACTCCTGCTGA

AAAAGGTTTGACTTTGATTTGGCCGACTGACTTTACGCATACACATAGCGGACAAATTTCCAAGACCCACGAAAAATATA

TCATAACTGGTTGGTTTGGGTATCAATTATAAATAGTAATATGCCAGCAACAGACGCATATTTAGGAAATCCTAATTTAA

AAAAAGTAAATATACCAGTTGAATTTACTAAAGACCAAATTGTAGAATTTCAGAAGTGTAAAACAGATCCAATATATTTT

ATGGAGAAATGGATGAAAATCGTTTCTCTTGATGAAGGACTTATATCTTTTAAACTATATGACTTCCAAAAGAAGATTGT

AACTACAATAGATAAAGAAAGATTTACTATTTGCAAATTGCCTAGACAATCAGGTAAATCAACAACAACAATTGCATATC

TTTTACACTATGCAATATTTAATCCAAATTCAAACATAGCAATTCTTGCTAATAAATCTTCTACTGCTAGAGATATATTA

GGAAGATTACAATTGGCATATGAAAATTTACCTAAATGGTTGCAACAAGGAGTTATTAATTGGAACAAAGGTAATATAGA

ATTAGAAAATAAATCTACTATTATTGCTGCCGCTACATCTTCAAGTGCAATACGAGGAGGAACATATAATATAATATTTC

TTGATGAGTTTGCTTTCGTACCTGCTAACATTGCTGAAATGTTTTTTAGTTCAGTTTATCCTACTATTACATCTGGTAAA

ACTTCAAAGGTTATTATAGTATCAACACCTCACGGTATGAATCAGTTTTATAAATTATGGACAGACGCTGAAAATGGAAG

AAATGATTATAAACCTATTGAAGTACATTGGTCAGAAGTTCCAGGTAGAGATGACAAATGGAAAGAAACAACTATACGTA

ATACATCAGCAGCACAATTTCAACAAGAGTTTGAGTGTGAATTTTTAGGGTCAGTAGATACATTAATTTCACCAGTTAAG

ATTAAACAAACACCTTATATGACACCATTAACTTCAAGTGGTGGTTTAGATGTATTTGAAAAGGTTGTAAATGGTAGAAA

TTATGTTTGTTGTGTTGATGTAGCAAGAGGTGTAGATAGAGATTATTCAGCATTTTTAATGTTTGATGTAACTCAAATGC

CTTATAGAGTTGTTGCCAAATATAGAAGTAATGAAGTTAAACCAATTCTATTTCCACACTTAATACAAAAAGCGTGTAAG

GGTTATAACACGGCAGATATTCTTTGTGAAACAAATGATATAGGTCAACAAATAGGTGAATCATTAAACTATGAATTAGA

ATATCCTAATCTATTAATGACTACTCAAAGAGGTAGAGCAGGTCAGATATTGGGTGCAGGATATAGTGGAAGAGGTTCTG

GTTTTGGTGTTCGTATGACAAAACAAATTAAAAAAGTTGGTTGTTCTAACATTAAGACATTGATTGAAGGAGATAAAGTT

GTTATTAATGACTTCAATATCATAGAAGAAATGTCAACCTTTGCTCGTAAAGGAAATTCTTGGCAAGCGGAAGAAGGATG

TAATGATGATTTAATGACTTGTCTTGTATTATTTGGTTGGTTGTCTAATCAACCTTACTTTAAAGAAATGACTAATACAA

ATGCTAGACAACAATTATATGAAGAACAAGAAAAATTAATAGAGCAAGATATGGCTCCTTTTGGTTTTGTAGATGATGGT

ATACCTGATTGGGAAAAAGAAACAGTAGATGAATATGGAACAGTCTGGTATCCAGTTGTCAGAAAAGGGCTCTAAATTAA

GTATTATATAAATATCCATAGTTATGAAATTTGACTATGGTCGTATGAAAACATACGGAATATGCGAAAAGATACAAACT

AATTAGTTAATTATAAGGAGAAAACCTAATGGCATTTCAAGTATCACCAGGTGTTCTCGTACAGGAAAAAGACTTAACAA

GAATTATTCCTGCCGTATCAACATCTTCTGGAGCTTTTGCTGGAACTTTCAGTAAAGGACCTCTTGACGAAGTTGTAAGT

ATCGGTAGTGAATCTGATTTATTGCTAACGTTTGGAAAACCAGATAGCTCAAATTTTGAGAGTTATTTTAGTGCTTCAAA

CTTTTTACAATATTCAAATAACTTGAAAGTAGTTCGTGTACAGAACTCATCTGTTTCAAACGCAACTGAAAGTGGTAGTG

CGTTTGTTATAAAGAATACTACTGATTACCAAAACAATTATGCTGACGGTTCTGCTTCTGTAGGAATGTGGGCTAGTAGA

ACAGCGGGTGCGTGGGGAAATAATTTAAGTATTTCTCAATGTCCTTCTGCTACTGCTTATGAAGAAACTGCTAAAACAAC

TGTTGCTGACGCTTCAACAAGTGTCGGAGATACAGTAGTTACAGTTACTTCCGCTACAGGAATTAGTGCTGGAGATATAG

TTAATTTCGGTGATGAATATGAATATAGAGTTATTAGTATTTCAACTAACGACTTAAACATTGTGCGAAAAGAAGAACCA

ACATATATTGGTACTTCTGACTCTTCTGGATTACAAAAGACTATTACAAATGGTGCTAATGTAAGACGAAGATGGAGATA

TTATGACCTATTTAATAAAGCACCAGGAACATCTACTTACGCTTTAACAAGAGGCGGTAGTGGAGATGAACTACACATAA

TTGTAGTTGATGAAGACGGTGGAATTAATGGAACTAAAGGGGAAGTTTTAGAAAAATT

>lcl|3300005603_____Ga0066853_10000534|429135_ __Eastern_Pacific_Ocean_-_ETNP201406SV61

ATTATCAGCGAGTGATAGTATAAATTTATTCGGAATTAAAACAAGTTGTGTATATGGAGTTAAAGTTTATCCACAAGTAC

CTAATTCAGGAATCTTTAATGTTAAATCATCTAGTTCATCTACTAAATTAAATTTCTTTTTAGCACCAAGTGAAATTGAA

CATACTTATGTTAGTGGTGGTACAGTTAAATCAGCAACACCAACTAGTGTTGGTAGTGCAACTGAAATTAATAGTGCTAC

TTATGATAATGTAACAGGACTTATTACGATAGGATCACCTGGTCACGGTTTAATAGTTAATGATTTAGTACAAGTACAAG

GAATGCAATTCACTTGTTCAACAGGTAGTAAAGCATATCCTGATGATATATTAAGTTCAGGAATATTTAAAGTTTATGAT

GTGCCTGACGTAGATACATATATCTTTGGTGTTGATAAATCAGCAATTGCTCATACTTATGTAATTGGTGGTACTTCACA

AAAAGTTACCATCGCTACAAGTTCTAGTGTTAACGTTTCAGGTTTTGTTTTCAACAGAACAGCTGCTGCTCAACAAGGAC

AAAGGGGTCCTTTGATTGCAGTTAAATCAGGTACAACAACTTTAAATGCTGTTGATATGATAGCTTTAGCAAGTAATGTT

AAATTCCCTAATGATAATACATTTTATAGAGTAGGGTTAGTATCAGAAGAAGATACGAGTGCTGGAACAGCAGTAATAAG

ATTAACTCAAAATATTGGTTTGAGTAAAGCCAAAAATGAGGATACAGTAAATAACATAACAGAATTATATTCAAATATTC

GTTTAACAGGTCACGATTTCTTAAATATTGGTACTGGTGATTTTACTACAACTAATTATCCAAATGTACCTTTACAAACA

AACGACCAAGCAGATGAAGTAACTGAAGTTAACGGTGGTCGTGTATATTGGGTATCAACTGACCAAACTGGTGACTTTAG

AGTTGGTGATTTATTCAAAATTGAACAAGCAACTGGTAGTGCAACATTAAACGCAGACGCATTTAACCTTTCAGGATTAA

GTGAATTAAAACTTGGTTCTATTGGTGCAGAATTAGGTGCTGCCATAAATGAATTTAGTACAGACGCAACTTTAGGCGGT

AATTCAAATACAGCTATACCTACTGAAAATGCTGTTGTCGGTTATATGACAAGAGATAAAGCAGGTACAGGTGCGTGGGT

TCCACCAACAGGAACATCATCACAAAGACCTGTAGGCGGTGAATTATTTGCAGGTGCTTTAAGATACAATTCTTCAATAA

TTTCTTGGGAAGGTTATAATGGAACAAGTTGGACAGGTCTAGCTGGAGGAACTCCTTGGACAACTCTAGTTGGAGATGGT

TCAACTGTACCTACAGCAATAGGTGGACAAAGATTATTAATAGATACAAGTTTATTTGCAATGACAGTTAAATTGCCTGC

TAGTCCACTAGTAGGAGATTCAATTGTATTTTTAGATTTAAACGGATCATTTCAATTAAGACATTTAACTGTTGATAGAA

ATGGTCAAGATATTATGAATTTACAACAAGATATGATTGCTGATATCAACCACGCAGGATTCACTTTAGTTTATACTGGA

TCAACAAATGGTTGGAAATTAGTAGAAGTAGCGTAATAAATAAATATAGAAGAGAAATATAAATGAGCAAATTAACAGAT

TTTACAGTTACATCCGCTGAGAAAGATGACTTTTATGGATTCCATAGAGTTGCTCCTTCTCAAACGATACATAGAACCCT

TACCTTAATTACTGGTAATGAAAGTGTATATGAATATACATTAGGAACAGGTTGGGATATTTCTACAATGGCATATTCAA

CATCTTATTACATAGGGTTTCACGATTCAAATCCATTAAACACAACGTTTAGTACTGATGGAACAAAAATGTTTGTTATG

GGCAATGCAGATAAACACGTTGATGAATATATATTGACTACAGCTTTTGATATTTCTACAGCAAGTTGGAGAACACATAA

AGATGTATCTGCTCAAGATGATAATCCAAGGGCAGTAAGATTTAATCCAGACGGAACTAAAATGTATGTTGTGGGTAGAG

ATGGAGTACCAAGTGCAGGTATAGCTGCTTGTAATATTAATGAATATGCATTAAGTACAGCTTGGGATATAACTACAGCA

ACTTATACAGATTTATTTTCTTGTCTTGCTCAAGATACTGCTATTAGTGATATGCGATTTGGTGATAATGGAGATTTATT

AATTGTTCTTGGCGATGAAGGTAATGATGTTAATGAATATGAATTAACTACAGCGTATGATATTTCTACAGCAACTTTTG

TAGATTCTTTTTCTATCGGTGCTCAAGAAACAAGTCCAGCTGGGTTAGGTTTTAATACAGATGGAACAAGAATGTATATT

GCAGGAACAGATGGAGATGATGTTATACAATATCCATTAGTAACAGGTTTTGATGTTTCAACTACACAAGCACTTACACA

CGAAGTTGGTTTAACTAATGCTCCTTCAGCACCAACAATGAACCCACGTGGTTTAACTTTTAATGCTGATGGAACAAAAC

TGTATGTTATAGGAACTGCTGGTACATTAATGATTGATGGTGGCGATGATGAATTACCATATAGTCACGTAGCTAGAAGT

CAAAACACTATGACATTTCTTGAAGGAAATACGTATGTGTTTGATGTTTCTCAATCTGCTTTAGTCGGACACTCATTAAA

ATTTTCAACAACAGTTGATGGTACACATAAAGCAGGTGGAACTGAATATTTAACAGGAGTAAGTTCATCTGGAACTCCTG

GAACTCCTGGAGCTACAACAACAATTATTGTTCCAAGTAAAACACCAAGTATAGAACCAGGAAGTGCTGTAGATAAGTTG

TATTATTATAATGGTGGTCATCCAAGTCAAGGTGGTGAAATTTTTACACCTGAATGGAAAGGCAATTTACAGATTACTTA

CACAAATGGATATGATGATATTGACACTAGACATAAAACTAAACATCAAGAAGATATATTTGAGGATAGTGTACTATGGA

AAAGAGGGTTGGTATTTACGGTAGTCAACGGAAACCTAACCATAGAAATGGGCTAAAAAAATATTTGAATTAACTAAAAT

GGAAGAAGAGAACTATTATAAATATAAATAAGGATCAAGAGAATTATGGCAACTATAAATTTAGGAAGAATTAAACCAGT

ATTTCAAGGGGCATATAATGGCGCTACTGCTTATGTAGTGGACGACATTGCTACCTATGGAGGCGAAACTTTTATTTGCA

TTTTAGCTTCAACTGGAAACGCAACTTCAAATGCAACCTATTGGTCTAAAATAGCCAAAAAAGGTGATGACGTAACACAA

CTTACTACCCACGGCGATATACTGTTTAGGGATGCAAGTGGTGTACAAAGATTAGCGGCAGGCACAGCAAATCAAATGTT

AGTAACTAAAGGTGCTAGTGCTGATCCTGTTTGGGGTTCTTCAACTTCAATTTTATGGGAAACTAAAACAGCTAATTTTA

CTGCTGTTCACGGCGGTGCATATATATGCAATACAACAGACGGTGTATTTACAATGACACTACCTGCTTCACCAGTAGAT

AACGACTTTGTTATTATCAATGATGGTATGGGAGTTTTTGATACAAAAAATCTTACAGTTGATAGAAACGGAAATAATAT

AGCAGGAAGTGCTACAGATTTAATAGTAGATAAAAAGTATGCTAATTTCAGATTAACATTTAAAACAATACCAGATGTAA

CTTCATCTTTTATTGGTTGGATAATTTCATAATGAAAGATATAAACTGTATAAATAGTATTACAAACAAAATTTTAAGGG

AGAACATTTAATGAGTTCATTAACAACACTTTTAAGCGGCGGTAGTGCTGGTGCAATAGACCACAGAAAAGAAAGTCTAC

CATTATACGGATTTTGGGGAGATAACTCCGACGGTAATCACCATATAAATTACAGAATCTTTGATTCTGGTCATAATAAC

GTAGGGTCTCCTTGGGGTGCTGTATGTAACTCAACAACAAATTATAGATTTGGGATGATGGGTGACGCTTCTTTTACTTA

TTCACACAATGACCACGGTACAAACGTATCACACGCAGATTTAACTTCACAACAATATGACTCTTGGACTAATTGGAATA

AATCATTATACCAATGTGACCAATATCCACACGCACAATATTATTCATCTTCAAGAGATGGATTTGTTTCTTGGCATAGT

TTCCACGAATACACATCTTCTTTTGAATATCAAGATGGTTGGACAAAATTAAATATGGTTCTTCCTGAAGGTATTAGACC

TAGACGTATGTTTGTTAATAGACGATTTACGTTAAGAGAAAGATATCCAGGTAATCACGGTGCTCCAAATATAGACACGT

ATGATTATACTTCTAACTTACTAGAAACAACTCAAACATATTCAACTGGTACTGGATACAATGAGAAAACAAAAACTTTA

GTTATGGTTCACTCTGGTGACGAAAGTGGAAATACTTCAAAAACTATTCACATTTTCAAATCTAGTAAATGTTTAAATAA

AATAGACAGAATTAAAGAATTCTTTGATAACTTAACTTCAACTGAATACTTTACTGACACTTGGACTAATCAATGTACTA

AAGATTGGTGCGTTGTTGTTGGTAATAATGATTTCGTTGGATTCGGAAACAAACAAAGTAATAGTAAAAGATACGGTGTA

TTTGATTGTTCAGTCAAAGGCGGAACTGCTCACCAAACTGGTGCAAGTAGACAATGGTCAACTTGGCAAGATTTTACAGG

ATCAACAACTACATCTTACGGTGCTAATAACGGACACCAATACTACTGTAAATTTATGACGACTTGGGATGGAACTTGGG

GAATGATTTATTCTTCATATTATTACTATGGTGTTGGTATCAATGGTTTCTGTATGAGTATAGAAAATCCTAAGAAATTT

ATTAATATAAATCAAACTAAATCAAGTAGAGCCAATCCTTACTTTGCTTGGGGACGTACAGGTTTCCACGGAGGTTGGTC

AGACAACTGCGACGGAACTTCTCATAGAACTTACTCTTGGTCTTTTGATCCTACGGCAACAGATGAAACCGAAGACACAC

TTGTTTATCAAGGTGGTTCTTCAGGAGATGAAATTATACCTAATTCTAACGCACACGTTGGAACTACAGTAACTAATAAA

ACTGGAAATTACGGTTTACACGCTTGCAGAACTTGGCTAACTGGAGGTTTCCATTCAACAAACTATCCACTATTAATGCA

AATTGACTGGTGGGGAAATTATGGAAACAATGATTCTACTTACGGTGGAAAATACGGAACATAGGAGATTATAGAAATGG

CAAGAACATATTACTTTACACTAGCAGGTGAACCTTTTTCACCAAATGCTGAAACTGGAGATGACGCAGTAGCAAAAGGA

AACGCAATCAAAGTTGATGACGTTCCTGAAGGAATTGAAGCGTGGAGATTATCAATTAATCCTGCAACAAAAGAATTAAC

AATCGTTGGCGGAGCTGACGGTGATGAAGCGGCTGCTGATACTGAAAGAGAAACAAAAGCTAATGACGAAGCAGCTGTAC

TAGCTAAAAAAGCAGAAGACTTATTAAAAGCAGAAGTTGCAGAAACTAAAAGACTTCAAGACGCTGGTCTAGCTTAATTT

ATGTAGTGATTTTATTATGATAAGAATTTTATTATGTATGACATCAAAGAATTAACCAAAGATATACACCAAAACGCAGA

AAGACAAGAGTTTGTCAAAACTCTTATGTCAGGTTCTATTGAACCTAATCTTTACGCAACCTATCTTTACAATCAATTAC

AATGTTATGCTGTATTAGAAAAATATGGAATAGAAAATTCTCTATTTCGTACAACTCCTAATTTACCTAGAGCAGAACAT

ATACATTATGATTTTAAAGCATTATGGACAAGTGAAGGCAAACCAACTATAACTCAAAGTACAAAAGATTATGTTGCTCA

TATTGAAACAATCAAAGAAGACGCAGAAAAATTATACGCTCATATCTATACTAGATATTTAGGAGATATATCTGGTGGTC

AAATGATAATGAGAAAAACACCAGGACCTAATCGTTATTACAAGTTTAAACATAAAGAAATAAAAGAGTATAAACGAATA

GTAAGAGAAATGATAAACAGTTATTTAAATGTTTATAAACTTAATATTCTAAATGAAGCTAAATTTTGTTTTGCGTCTGC

TACAAAATTATTCCAAGAGATGAAAGAGATTGAAGATATGTATTACAAACCTTTAATTTTAACTAACGAGGTTAAAGATG

ATTTGGGAACGACTAATTAAATTAGAAAAAGATATAATCGCTATACTTGATAAGCGTTGTAAAGAATACAACGAAGATGG

TATGGATAGATTTAATAATGATACTTGGGTTAACCGTACTTGGTCTAATATGAGTATAAGACGTGCTCACGTAGACGTAG

TGGATGCTAGAGAAACAAAAGGTCTTTGGATGGCACACATATGTTTATTTCCAATGTTAACTAATGGTGGTCCAATTTAT

GGTTTTGATGTTATTGCAGGTAAGAATAAAGTTACAGGTGCTTTCCACGATTATAGTCCGCTATTATTAAAAGAACATCC

CTTAACAAAGTATTTTATAGAAGAAACAAAATGGTATAAACCATCTAAAGAAAGAGAATTGCCTGATTGGGCAAAGGAAA

TCTTTAGTCCAGGTATGATTGCCGCTGGTAATATATCAGATGAAAAAGAATTAAACCAAATTTGTACTCTTGCTACATCT

AATTTAGAAAATTATCTTGACAAAATTGGTCATTATAATAGCGATTCAAAGGAAGAAGATGTAATAAGAGCACAAAATTT

TTATTGCGAACACCAACAACAAAATCCACACACCCCTAGAGTAATGAAAACTCTTGGACTGCCTGAAGATGATATAAAAG

TATTCTGTACTGATAATTTGTTTCCGAAGATATAATTGTTATTATAAATATACAATAAAGGAACCAGTATGGCAGAACCA

GCATCCAGAGAAACAGTAAAACAATACGCTTTAAGAGCATTAGGTAAACCAGTAATTGAAATCAACGTTGATGACGACCA

ACTGGAAGATAGACTTGATGAAGCATTACAATATTTTGCTCAATACCACTATGATGGTGTTAAAAGAACCTATTTAAAAT

ACAAGTATACAGCGGCAGATAAAGCTAGAATTTTAGCAGATAGTACTGAAACTGAATCTAAAACGTATGGTGATTCTTCT

GTAGTAAATACAGAATGGAAAGAAGGCAATCAGTATATTGTATGTCCTGAATCTGTTATATCTGTAATTAACATTTTTCC

ATTTTCAAATAAAGGTAATTTAAATTTATTTGATGTTAGATATCAATTAAGATTAAATGACCTATATGATTTTTCTTCAA

CGTCTGTTATTAACTATGATGTTGTATTAAGACATTTAGATTTTTTAGACCATATATTAGTCGGAGAAAAACCTTATAGA

TTTAACCAATTAGATAATAGACTTTATGTTGATATGGATTGGAAAAATGATTTACAAGTAGATGAATTTCTTGTAATAGA

ATGCTGGAGAAAATTAGACCCTAACACATATACAGATGTCTTTAATGATATTTGGTTAAAAAGATACGTAACTGCTTTAT

TTAAAAAACAATGGGGAGCCAATTTAAGTAAGTTTGATGGTGTTGCAATGCTTGGTGGAGTTACATTAAACGGTAAACAA

ATTTATAGTGAAGCACTAGAAGATTTGGATAAATTGGAAATAAAATTAAGAAGCGAGTTTGAAGAACCGCAACCTTTTAT

GATAGGATAATGCTATGCCAGTTAATCATTACTTTTCAGGTGGAAAAGGCATAGGTAATGCTGCCGAAAAAAGACTACAC

GAAGATATAATAGTTGAAGGTCTTAAAATTTACGGTCAGGATGTCTATTACTTACCACGAACATTAGTCAATAAAGATTT

AATACTAGGAGAAGATGTATCTAGTAGATTTGATGATTCTTATTTGATAGAAATGTATTTTGAAAATAATACAGGATTTG

CTGGTGAACAAGAAATCATAAGTAAGTTTGGATTAGAAATTCGTGATGATACATCATTAATGGTTTCAAAAAGAAGTTGG

ACAAATTTAGTTGGTAATAAGGCAACACAGGTTGGTTCTTCTCTATCAATTACAGGAAGACCAAACGAAGGTGATATTAT

ATATGTGCCTTTGATGAAATCTTTTTTTGAAATTTTATTTGTAGAAGACCAAGAACCATTTTTCCAATTAGGCAATCTGC

CAGTTTATAAATTAAAAGTAACTCGTTGGGAGTATGCAAGTGAAAAACTTGATACTGGTTTATCTACTATTGACCAACAC

GAAGATACACATACACTAGACCAATTAGCATATAAATTTACTTTAGAATATGGACAAGAAGTTATGACAGGTGCAGGTTC

AGTACAATTAGAAAGTTACCACGATTATTCAACTGGTCAACCAGCACTTTTAATGAACGAAGATTTTACAGAGTCTAATA

TACAAACACAATCTCCATATGCAGATAATTTAGACTTGAATAAAGAGGCAGGATATGATACAGTATCAACAGCGGATGAT

ATACTTGACTTTACAGAAAGAAATCCATTCGGGGAAATTGACGAGTAGACTATATGTTCGGAACACATTTTTATAATCAA

AGTTTAAGAAGACTAACTATTGCATTTGGACAGATTTTTAATAATATAATTATTCAACAAAAATCTGGTACAGGTGCTAT

TACTAAAAGAATACGTGTGCCTTTAGCATACGCTCCTAAAGAAAAGTTTATAGCCAGAATAGACCAACAAGCAAGTTTAG

AAAAAGGTAAAACGTTTGCTATTGTATTACCTAGAATGGGATTTGAATTAACAGGTTTAAAGTATGACGCTACTAGAAAA

CTAAACAAACTTCAAAAAACAGTTAGAGTTAAAACTTCTGATTCTACTATACATAATTTTAATTATTCACCAGTACCCTA

TGATATAAGTTTTAGTCTTTATTCTTTTACTGCTACAGCAGAAAATGGACTACAGATAATTGAACAAATATTACCATATT

TTGCACCAGACTATACAGTTACTATTAATGCAATACCAGAATTAAATATTAAAAGGGACGTGCCTATTGTTTTAGATACT

GTAAATTATGAAGATACTTATGATGGTGAATTTAATAAGCGTAGAGCAGTTATATATACTTTAGAGTTTACTGCTAAAAC

TTACTTATATGGACCTATGGCACAAAGTAAAGTTATTAGAAAATCACAATCAGATTTAGGAACATCTACGGATGCTCCTT

TATCAAGAGAAGAAAGAATTATAGTAATACCAAATCCTGAAAGTGCTAATGCAGATGATGATTTTGGATTTACAACAAAG

ATTAGTTTCTTTGATGATACAAAGAAATATAATCCAGTAACAGGAGAAGATGAATAATGCCTAAATTGGAAGATAGTGTA

AATGAGATATTGGGATTAGAAGGAAATAATAAAGTTGTACCAGAGAACCTTGAACCACAAAAAGGTTTTCAACCACCTGT

TCCTAGAAAGAATGGAGAAGTTCCTTTAAAAGTTGAAAAGGATATTAATACTGATTATGATTACAGTAGAGAAAGTTATT

ATAGTATAATAGAAAAGGGACAAGAAGCAATACAAGGCATATTAGATATTGCAAAAGAAGGACAACACCCTAGAGCATAT

GAAGTTGTTGGTCAATTGATAGGACAAGTTGGTACTACAGTTGATAAACTACAAGATTTACAAAAGAAATTTAAAGACTT

AAAAGAACTACCTGGAAGAACAAATGCAAATATTAAAAATGCATTGTTTGTAGGGTCAACTGCTGAATTACAGAAGATGT

TAAATAAGCAAACTATGGAAACAAAAATGGAAAAGAAAAATGAAAATGAAACTATTGACGGCAAATCAAAAGATACCGAA

TAAAATTCCTATCCTACTAAAAGACTTAATCTATATTAAGTCAATGACACCACTAAAAGAATTATTAGATGGTGAAGAAT

TACAAAATCCAATAGAAGTAAAAGAACACGTTGTATCAGAAGTACCTAGATACGGTGCAATGGGTATACCCTATATAGAA

AGAGAATATAGTGTGTGGAGAGGTAGTCAACGAGTGCAGGCAGCTAAACAATTAGGGTATACACATATAGAAGGAGTGAT

AGTTAAGTGAAACATTTAGAAGAATTTACAAAAATAATAAATGAATATAAAGAAGATGGAAGATACCGAGTCTTTAATGA

TATAGTTAGGACTAGAGGAAACTTTCCTCACGCTATTTGGTATTCAAAATACTCAATTAAAAAAATAGTTAATTGGTGTT

CTAACGATTATTTAGGTATGGGACAACACTCTTATGTTATAGACTCAATGAAAACAGCATTAGAATCAAGTGGTGCTGGG

GCAGGAGGTACAAGAAACATATCTGGTTCTACTCACTATCATAAAGCGTTAGAAAATGAATTAGCAGATTTTCATAAAAA

AGAAAAGGCATTATTATTTACTTCAGCATATAATGCTAATCAAACAACTTTAGAAACTTTAGGAAAAATTATACCTGACT

TATTGTATATATCAGACTCATTAAATCACTCTTCTCTTATACAAGGCATTAGGCATAGTAGATGTAAGAAAGAAATATTT

AAACATAATGATGTAGAAGATTTAGAAAGAATTTTAAAATCATACGAAGGTCCAAAATGTGTAGTATTTGAAAGTGTATA

TTCTATGGACGGAGATATTGGACCAGTAAAAGAAATAGTAGAACTAGCTAAAAAATATAATGCAATAACATTTTTAGATG

AAGTACACGCTGTTGGTCTTTATGGACAAGAAGGTGCTGGAATATGTGAAAGAGATAATGTAGAAGTTGATATAATAAAT

GGAACATTAGCAAAGGCGTTCGGTGTACAAGGTGGATACATTGCAGGAAAAAAAGATTTTATTGACGCCATAAGAAGTTT

GGCAAGTGCTTTTATATTTACAACTAGTTTAAGTCCAGTTATTTGTGCTGGTGCTTTAACAAGTATTAAATATGTTAGAG

ACCATCCTGAATTAAGAGAACAAATACACGAAAGAGCAAATAAAACTAAAGAAGAACTTGCTAGACAAGGAATAGAAGTT

ATGAAAAATGATAGTCATATTGTTCCTGTAATTATTGGGGATGCTAAAAAATGTAAATCAATATCAGATGAACTTTTATA

TAAAGAAGGTATCTATGTACAACCTATTAATTGGCCGACTGTTCCTGTAGGTACTGAAAGATTAAGATTTACTCCTACAC

CATTTCATACAGACGCATTAATCTTTGATATGGTAGTAAAACTAAAAGTGGCAATGAAAAAATGTGGTGGTAGAAATGCA

ATACAAAGTAATGCCTAAACATAAAGAATATATTTTACCAACAACTAGTTTAATAGGAGGTTGGTATATTCCTTCTGGTA

TTTGTGATGGACTTATAAACTTATTTAAAGATAATAAACAAGCACAAAAACCAGGTGTTGTAGGTTTCACTTCAAAAATT

AATAAAGAAGTAAAAGATTCTATAGATATTGGATTAGATCCAAATTGGGAAGAACCAAGGTTTATGAAATATAAAAATGC

GTTGAAAGAATGTGTTGGTCTATACGAAGAGAAATATCCTGAAGTTAAAGAGTTTGAAAGATATGGAATGGTTGAAGGAG

GAAATTTACAATACTATCCACCAGGTGGAGGTTATTTTACTAAGCATTGTGAAAGAAACTCTAGGCACGAAAACCGTTGT

CTTGTTTGGCTGACTTATTTAAATAATGTTCCTAACGGTGGTACACATTTTAAATATCAAAATGCAACAACTCCTGCTGA

AAAAGGTTTGACTTTGATTTGGCCGACTGACTTTACGCATACACATAGCGGACAAATTTCCAAGACCCACGAAAAATATA

TCATAACTGGTTGGTTTGGGTATCAATTATAAATAGTAATATGCCAGCAACAGACGCATATTTAGGAAATCCTAATTTAA

AAAAAGTAAATATACCAGTTGAATTTACTAAAGACCAAATTGTAGAATTTCAGAAGTGTAAAACAGATCCAATATATTTT

ATGGAGAAATGGATGAAAATCGTTTCTCTTGATGAAGGACTTATATCTTTTAAACTATATGACTTCCAAAAGAAGATTGT

AACTACAATAGATAAAGAAAGATTTACTATTTGCAAATTGCCTAGACAATCAGGTAAATCAACAACAACAATTGCATATC

TTTTACACTATGCAATATTTAATCCAAATTCAAACATAGCAATTCTTGCTAATAAATCTTCTACTGCTAGAGATATATTA

GGAAGATTACAATTGGCATATGAAAATTTACCTAAATGGTTGCAACAAGGAGTTATTAATTGGAACAAAGGTAATATAGA

ATTAGAAAATAAATCTACTATTATTGCTGCCGCTACATCTTCAAGTGCAATACGAGGAGGAACATATAATATAATATTTC

TTGATGAGTTTGCTTTCGTACCTGCTAACATTGCTGAAATGTTTTTTAGTTCAGTTTATCCTACTATTACATCTGGTAAA

ACTTCAAAGGTTATTATAGTATCAACACCTCACGGTATGAATCAGTTTTATAAATTATGGACAGACGCTGAAAATGGAAG

AAATGATTATAAACCTATTGAAGTACATTGGTCAGAAGTTCCAGGTAGAGATGACAAATGGAAAGAAACAACTATACGTA

ATACATCAGCAGCACAATTTCAACAAGAGTTTGAGTGTGAATTTTTAGGGTCAGTAGATACATTAATTTCACCAGTTAAG

ATTAAACAAACACCTTATATGACACCATTAACTTCAAGTGGTGGTTTAGATGTATTTGAAAAGGTTGTAAATGGTAGAAA

TTATGTTTGTTGTGTTGATGTAGCAAGAGGTGTAGATAGAGATTATTCAGCATTTTTAATGTTTGATGTAACTCAAATGC

CTTATAGAGTTGTTGCCAAATATAGAAGTAATGAAGTTAAACCAATTCTATTTCCACACTTAATACAAAAAGCGTGTAAG

GGTTATAACACGGCAGATATTCTTTGTGAAACAAATGATATAGGTCAACAAATAGGTGAATCATTAAACTATGAATTAGA

ATATCCTAATCTATTAATGACTACTCAAAGAGGTAGAGCAGGTCAGATATTGGGTGCAGGATATAGTGGAAGAGGTTCTG

GTTTTGGTGTTCGTATGACAAAACAAATTAAAAAAGTTGGTTGTTCTAACATTAAGACATTGATTGAAGGAGATAAAGTT

GTTATTAATGACTTCAATATCATAGAAGAAATGTCAACCTTTGCTCGTAAAGGAAATTCTTGGCAAGCGGAAGAAGGATG

TAATGATGATTTAATGACTTGTCTTGTATTATTTGGTTGGTTGTCTAATCAACCTTACTTTAAAGAAATGACTAATACAA

ATGCTAGACAACAATTATATGAAGAACAAGAAAAATTAATAGAGCAAGATATGGCTCCTTTTGGTTTTGTAGATGATGGT

ATACCTGATTGGGAAAAAGAAACAGTAGATGAATATGGAACAGTCTGGTATCCAGTTGTCAGAAAAGGGCTCTAAATTAA

GTATTATATAAATATCCATAGTTATGAAATTTGACTATGGTCGTATGAAAACATACGGAATATGCGAAAAGATACAAACT

AATTAGTTAATTATAAGGAGAAAACCTAATGGCATTTCAAGTATCACCAGGTGTTCTCGTACAGGAAAAAGACTTAACAA

GAATTATTCCTGCCGTATCAACATCTTCTGGAGCTTTTGCTGGAACTTTCAGTAAAGGACCTCTTGACGAAGTTGTAAGT

ATCGGTAGTGAATCTGATTTATTGCTAACGTTTGGAAAACCAGATAGCTCAAATTTTGAGAGTTATTTTAGTGCTTCAAA

CTTTTTACAATATTCAAATAACTTGAAAGTAGTTCGTGTACAGAACTCATCTGTTTCAAACGCAACTGAAAGTGGTAGTG

CGTTTGTTATAAAGAATACTACTGATTACCAAAACAATTATGCTGACGGTTCTGCTTCTGTAGGAATGTGGGCTAGTAGA

ACAGCGGGTGCGTGGGGAAATAATTTAAGTATTTCTCAATGTCCTTCTGCTACTGCTTATGAAGAAACTGCTAAAACAAC

TGTTGCTGACGCTTCAACAAGTGTCGGAGATACAGTAGTTACAGTTACTTCCGCTACAGGAATTAGTGCTGGAGATATAG

TTAATTTCGGTGATGAATATGAATATAGAGTTATTAGTATTTCAACTAACGACTTAAACATTGTGCGAAAAGAAGAACCA

ACATATATTGGTACTTCTGACTCTTCTGGATTACAAAAGACTATTACAAATGGTGCTAATGTAAGACGAAGATGGAGATA

TTATGACCTATTTAATAAAGCACCAGGAACATCTACTTACGCTTTAACAAGAGGCGGTAGTGGAGATGAACTACACATAA

TTGTAGTTGATGAAGACGGTGGAATTAATGGAACTAAAGGGGAAGTTTTAGAAAAATT

>lcl|3300005611_____Ga0074647_1002732|431381_Saline_surface_water_microbial_communities_from_Etoliko_Lagoon,_Greece_

ATCCGTGAAAATGCTGTTCACGCTTTTGTTATCAAAAAACTTACTGCAAAAACTGAAGCAATGGGTTTTAAAGTTGGTGA

CATTTTGAAACCTGCTGGATGGAGAGCGCCTGCTTTAAACAAAGCTCGAGGCAATGTACTTGAAGGCAATTTTTATATTA

ACTGGACAGGTCCTTTGTACCTGTCTTAATTGAGAAAGGACTATATTATGAAATATGAAGTTTATCACAATGCGTTTGGC

GACAAAGATGTTCATGTTGCTAATGTTGAAATTGCTGGTTATGTTCCAGTGATGAAAGCACTTGAAGAGTGTTTTCGCAA

AACTAACAACATTGAAGGCTCTTGGTCAAAAGGTCCTACTTTTGAATTTAAAGGAGAAACTTTTGACAATTCAGATTATT

CTGAAAATGTTGAAGTTGTAAAACCACTTCTTGTTAAAGACGGAGTTGAGTGGGGTCACCGTTCAACATCAGTTGGTGAT

TATGTCATTGTTAACGGTACAAAATATAATTGTGCCATGGTCGGTTGGGAGAAAGCCGCTTGAGTATAAAACAAATTGAA

TATTTGAAGTGGTTTGGTACGATATGCTTTTTATCAGCAGCTACCTTACTGTCTTCAAATATTGAAGTAAGTCGCTGGGG

ATTTTTTATCTTTCTTGCCGGCCATGTATCATTGACTTGGTTATTCTGGCGATTGAAAGATAATCCTATGATGATACAAA

ATGGATTCTTCATCTTCATTGATGCTTGGGGAATTTATCGTTGGTTCTTTTAAATATTAGCGCCAAGTCTAAACGCTATT

TTTTATAAATACTTATATGAAAACTTGTAAAAATTGTGGTGTAAAACACCAAATGGTAGGTTTAGAGTGTAGAGTATGTA

AAGATGGCCGATACAGATATGGTATGACAAGAACGGATATGATAAAATTACACGAACAACAAAATAAAAAATGTTATTTG

TGTGATAAAGAAATTGAAATGTTTAAAGGTCATTCTGGAGGTATGATTGACCATGACCATAAAACGGGAAAGGTCAGAAG

TATATTGTGCAACAAATGTAATACAATTGTTGGTGGATTTGAAACTCACAAAAACAAAAAAAAGTTGTTAAAGTACATCA

ATAAAGCTTGACACAGAATAAAGACTACCATATAATATAAGAACAGTACGGAGATTGGCTCAGCCTGGTAGAGCATCTGG

TTTGGGACCAGAGGGTCGTTGGTTCGAATCCAACATCTCCGACCATTAGCCATTTTTAAAGGAGAAAGTTTAAGTGGCCT

ATGTTAATCGTAACAAACGGTTTAAGAACCGTGAACTTCAATACAATACATCAAAAGAGAATCAACTGCGTCTAGTAGGT

GATTATATCCTTCACACATATAGTTTTAAAAATATACCGTACCAGTATTTTGAAACACTAAAAGAGTATTCACAAAAGGT

ACGCAAAATCAAGTTAAAAGTTAAAACCGAGAAAGATGAAAAAGGCAAAGTCTGGCCTGTATCTTTCAATGTTTATAAAG

TATCTGCCGTTAGCTCAGCTGGATAGAGCAACAGCCTTCTAAGCTGTGGGTCGCAGGTTCGAATCCTGCACGGCAGGCCA

ATTTCTAAGAGGAGAAAAAAATGGCCGCATTATTTGAAAAAACACGCTTTGATGTAGAACAAGAAATTATGGAACTTCAT

AGTTTCGCAACTATCATTCAGAACTATGCTAATATGTTATATGATGGCGAATATGAACAAACGGAAGATGATATACACAC

CACACTTTCTGGTTTTGCAAATCTTCTAATTAGTCATTCTGAAATGATGATGGACACTCATTGTAAACATTATGGACTAA

ACCAATATGCAACACCTGAACAAAAGGCAATGCACAAAGAAATGGGTATTGATAAGTTTTACCACGATATTGAAAAGGAA

AGGGGTAATGTCTGATACAGTTTATACTTGTGACATTATCCATGATGAAAAAACAGGCGACCAAATTCTACAGTTTCCAG

ATAAAATGATGGAAGAATTAGATTGGCGTATAGATGATGTTCTAGGTTTTGAAGTAGCAGAACTAGAAAGCAAAACAAGT

ATTATTATTCGAAATATCTCACTTGAAAAGAGGAAAGAAAATGGAAGTTAAAACAGGTTATACATTAATCAAACATTATT

ATAGTGTCACCTTTGATGAATTTGCACATAGTAAAGTAACCTTTGATATTGCTGAAGATGCTTTTTTAGACCAAATGTTA

GATGCGTTTGAACATTTTTTAAAGGCATCAGGTTTTAGTTTTGATGGACGATTAGACTTTGTAAAAGATGATGAAGATGA

CCCTTTTATTGATGAACTAGTCAATCATCAAAAATCAGAATTACCTTACGCTAGTGATAAAGAAATAGCAGACGAAAAAA

TAGGTAAGTGGTTAAGTGCAGCCTTGGAAGACCCAAGCACTTGTCAATCTATGAAGGACGACATCAATGAATGGTTTAGG

GTTACTGAATTACCACCTGTACGATTTCGGGCTAAAGAACCACATCCACCTCGGTGGGCGGCCACATCTGACCGTAATTC

AGTCTTTGATGAAAAGATGTAGTTAATGCCTCCGTGGTGGAAATGGTAGACACTCAGCACTTAAAATGCTGTGCTTAAAG

CGTGCCGGTTCGAATCCGGCCGGAGGCACCAACTATTATATTACCAATTATTTGTGGACTTGGTTATTCTATGTGCTAAC

ACTTTACCTTTATTAGTACCGTGTTTAACAATATAGCCAGATGTTCCATTTGCATTTATATCAACTTCTTTTCTAGCACC

AAATAACACTTTTGTTTTTTCTAACAAAGTTTGTGCTTTGTGTCTAGCTTTGAATAAATGGGTGAATCTGTCTGTCATAA

CACCCTCCTTTAATTAAAGTTAGGTGCGTTCCTTCGGCAAATGCCTACTTCCGGCCTGATGGCTGAACGGTAGTATTATT

TATATAACCAGGGGTACCAAAAGGCTGTGACTAAGTGAAGTGTTTTATGAACTGCCTTCCAATCACATTCTAACCATTCT

GTTTCATAGTTATATTCTTGGAAGTTACCAGCGTTATAAGTGTGTGTGTGCATTGCAATATTTAGTTTAACACAAAGTAC

AACAATCAGGTGTTCCACAATTTTCGTGTAATGTTACTTTATTTCTCTTTAGAGCTTCTTTTAGTTTGACAACCATATCA

TAAATCATACTATCTGTATGATAAGGTCCTGGTGTAAATCTTAATCTTTCTGTACCAACAGGCACCGTAGGATAATTGAT

AGGTTGTACATAAATGCCATCTTTGTAAAGTAAGTCATCTGATATTCTTTTACATTTAACGGCATCACCAATCATCACTG

GTACAATATGTGAATTGTTTTTCATCACAGGTATGCCTTCACTTTTCATTATGTCTTTTACTTTTTGACTTCTTTCATAT

AACTCTTCTCTAAGTTGATGATGTTCTCTTACATACTTAATACTGGTTAATGCACCAGCACAAATAACAGGAGATAAGGA

AGTTGTGAAAATGAAAGCAGGCGCCAGACTTCTTATGGCGTCTAGGAAGATGCGTTTGCCGGCAATATATCCGCCTTGGA

CTCCGAAACCTTTTGATAGAGTACCGTTAATTATGTCCACTTCCACGGCATCTCTTTCGGTCACACCTCCACCGGTTTTA

CCGTAAAGACCAACTCCGTGTACTTCATCTATGTAAGTTATTGCATTATACTTTTTACATATTTCAACAATCTCTTTTAT

AGGTGCAATGTCACCATCCATACTATAAACAGATTCAAAGACAACACATTTAGGACCTGTAACTTGTTTCATTAACAATT

CTAATTTTTCTAAATCATTATGCGACCAGATAATTTTATTTGCTTTACTGTGTCTAATACCTTGTATGATTGAACTGTGA

TTGTTTGCATCTGAGATATAAGTCAAATTAGGAATAATTTTTGCCATTGTTTCTAAAGTAGTTTCATTGGCATTAAAGGC

ACTTGTAAATAATAATGCGGCTTCTTTGTTATGAAGTTTTGCAATCTCTAATTCTAATGCAACATGGTAATGAGTTGTAC

CTGAGATGTTACGAGTACCGCCTGAACCTGCACCTGACATTTCTAAGGCCGTCTTCATACTATCAATAACAAATTGATGT

TGACCCATACCTAGGTAATCATTTGAACACCAATTGATAATGTTGTTGATTGAATACTTTGAATACCAAACGGCTGAGGG

GAATTTACCTTTTTGACGGAGTATATCATTGAAGACACGGTACTTGCCGTCTTTCTTCAAATCATTAATTACATTTTCAA

ACAATTCTAAATGTTGCATACACCTATTTATCACTTTAATAAAAACTTAATATCCCATTAATCTTGTTTTTCCTAAATAA

TAGTGACATCAACATTCAAAAAAAGGACATAACTATGTCAGACCCTAAAAATATATTAGAAAGATTAGAAGAATCAATTG

AAGACTTAAAAACTAGAATTGAAGAAATTGAATCCGTACTAGAAATTGAAAGAGAAGAGGAAGACAATGATTGGGAAGAT

GAAGACGATACTGAAGAAGATTCAGATGATGATACTGAAGAAAGTAAAGAAGACTAAATAATATTAAGGGCGGCTTCGGC

CGCCCACACTTTTTAGAGGTCTATATTTTGAACACACCCACCAAATACAAATCCATTTTTATTTCAGATTTACATTTAGG

TACACCAGGTTGTTCAGCTACCGAACTTTGCGACTTTCTTAAAAACAATGCCTGTGAAAGACTGTATCTTGTAGGAGATA

TTATTGATGGTTGGCAGTTATCTAAGAAAATGTTTTGGCCACAAGAACACTCAGATGTTATTAGAAGAATTTTAACAAAG

GCCAAAAGAGGCACCAAGGTTTATTGGATTGTTGGTAACCATGATGAGATGTTACGAAACTGGTTTGACTTTCGATTACA

GTTTGGCCGTATAAGAGTTTTAAATGAATATGTACATCATGCAATCAATGGTAAAAAATACTATGTCACACATGGTGATA

TATTTGACCCACTCATGCACTCTGGTAAATTCTTAATGTATTTTGGTGATTTCATTTACACATGGTTAATGAGATTAAAT

AGGTGTGTAGCATTTGTAAGAAGAAAATTAAGATTGCCTTATTGGTCACTATCTGCTTATCTAAAAGCACAAACAAAAGA

AACAATTAATATGCTTTATAAGTATAAAGAAACAATGGTGTTGCATTGTAAAAAGAAAGGTTATGACGGCATTATTTGTG

GTCATATTCATACACCTGCAATTGAGATGATTGATGGTGTTGAATACATGAATGATGGCGATTGGGTAGAAAGTCGAACT

GCCCTTGTTGAACATTATGACGGAAAATGGGAACTCATCCACTACAAAGATTGATTGGTGGTTTGAAAATCTTTGTGTTG

TAGCAGTCTATATTGTCATACTCT

>lcl|3300006166_____Ga0066836_10000757|181448_Eastern_Pacific_Ocean_-_ETNP201302SV91

CAGTACCAACAGAAATTCCATCAATCACTTGGGCAAATGGTTCTTCTGATCCTGGTAAGATTAAAAAATTATTAGTTGAT

TCTCAACAATCATACCAAAGGGTTGCTATTCTTACAGACAAAGGTAAGATATATTGGTGTGGAAGAAATGAATACGGTTG

GGCGATGATGGGAAATACAACAGATGTTAATACATTTACTCAAATGTCTGGTGGACCTGGAAGCGGAACTAATTCATATT

GCGCTAATATGTGGTTTACTGGAAATGGAAGATACGCAAGTTTCTGGACTAAAGATAGTACAGGTGCTATAAAATGTTGT

GGTTACAATAGTTCTTATGAATTAGGAATTGGAAATAACAACAACCAATCAGCTGCTGTATCACCTAAATGGCAAATTAA

TGGAACAACAACTGCTGATTTAGAAAATATTAAAGACATAGGTTGCAATAGTGAATATGGTAACCAATGGATGTGTAATG

TATGGGTATTAACTTACGATGGATTTATGTTTAATACTGGAAGAAACAACTATGGAATTGGTTGTCAAGGTTATTCTTCT

AGTTATAATGACAGACAATCAACAAACAATATAGAAGAAACAGATGATTATTACTTCCAAATGCAAAGAATGCCGAATTA

TGCACACGGCAGAATCGAAGATGTAAGAGGAAGAGGTTATTACTCAACTGATGGTAACAGATACCACTTTAGAGAAATTA

GAACATTTGATAACAGGTACTTATTATGGGGTTATGGTGGAGATTATATAATGGGACAAAATGATGGAAATTACCACTCA

ACTGCTCAACCACCTGTTCTTGGATAATAAATATAATAGAAGGTAAATAGGAAAACAAAATGGCAAAAATAAATCTCGGT

AGAATTAAACTCCAATTCCAAGGAGAGTTCAATAGAGACCAAATGTACAGAAGAGACGATATTGTCTACCACTCAAATGC

GATGTGGATTATGACAAATGAATATCTTCCAGATGGTTCTAGTGCTTACGCTCCAGGAAGTAAAGTTCAAGGTTACAATG

TAAAAGAAAAAAATATTGTACAAGGTGGACAAGATCCAAACTATAACGGTGCTGACGCATTTAGTTATACACAATATTGG

ACAGAAAACGAAAGAAAAGCTGAAAGACAAAGAACAGATAGAGACGGAAATCCTGTTCAATACAATTCAACTTATGGCTC

TAACGAAGATGGAGCACACGAAATTAATTATAACCAAATAGATACTCAATTAGGAACAATTGTACGCCATCAATCCCACC

TAATGGATGAGTATGACGCTATGTTCCGAGAAACAGAAGACGACTATTCAGAAATGGATACTTACAATGGTTATGAACAA

ACTTATTTTAGATACCACTACAGACCAGTAGACAATACTTTTGATGTTGCTGTTAATGTTTCTGGTGGTGTACCAGATTT

CAAAATTGACAATAGAGTAGGTTCATCTACTAAAGGAAGACAATTTGCTGGTTACAGAAACTTTGAGTTTGTTAAAGAAG

GTCATAGATATGCTTTCGTACAACACAAAAATTCAAACAAATTTTATCCGTTAGGATTTTCTTATACTGCCGATGGTATT

CATAATACTGGAAATACAGGTAAGTCATTAGGACAAGACCACGATGGTCCTTACTATGTTAAAGGTACTGCTTCAAATGG

TGATAGTGGTTTCTTTTCTCCATTATACAAAACTGCTGTTGCTGCTAACGCTGAAGATACAAGACGAGGTGGACAAGGTG

TTTCTCACAAATTAACTTTTGACCAAGGTGATGTACCTGGTTATGAAACAACTTCTGTTGCTTTACAAGGTTGGTTACAC

GATGACGGAACACAAAGAAAAGATACACAAGTTTCTGTTCTAACAGACGCTTCTGATAACACTTACTTACAGGTTACAAA

TGCTTGGGAAGGAACTACTTCTGGTGGTGCTTCTACAAGAACAAGAAAAACAATTTACTTAAACACAGGTGATTCAACTG

AACATCACGCACAAACAGGAACAACTTATTCTTATGTTTATGTTGATGGTGCTTTACAAGTTGGTTCAAATATAACTGCT

GTTATAACACAAAGTAGAGACGATACAACAACTGCTAATAATGGTGCTGGAAGACAAAGACTATTAGTAAACGGAAAACC

TGTTTACCAATTAGTTGCTGAAGCTTCTAATGTTGTTGTTGGTGGTATATCTGGTGCTTATCAGGCAATAGATAATACTG

GTACTGGAACAACTACTGCTTTAGGTTCATCTCCAACTTCTAACGAAGATAAGATTGACCTTTATATGCCTAAACTTACA

GACCCGACTGAAAATGCGGAAAGAACTTTCCAAATGACGGTTGCTTCTAGTAAATTTAATGTAGATGGTGCTGTACCAAC

TGCTAATACGGTTAAATTAGAAGAAGGAAAAACTTATAAGTTTGACCAATCAGATTCTACAAACGCAAGTAAGACATTAA

AATTCTCAACAACTAGTGATGGAACACACGCTAGTGGTTCTGCATACACAACTGGAGTAACCACTTTTGGAACTCCTGGA

AGTGCTGGTGCTCATACAATAATTAAAGTAAGAGCTAATACTGCTAAACTTTATATTTACTGCCACGAAAGTGCTTCTCA

CGGATTTGCAACAGAAACATATGATAGTGCTACAAATTTAGGAAAATCATATGCACCTGCAAACATAATGAAATGGAGAG

GTTTTGGTAAAAACGGATGGGTTAAATATTACCTAGATGGATACCAAGTAGATGAAAATACTTACATTGAGACATTTTTC

AATTCAATTGGAGACAATGATGACCACGAGTACAGACAAAAAATGGAAAATGGCAAATGGAAAGGTGGAAAACAATACAA

CTTTGCAAACAAAGGTGAGAGAACGGTTGAGTTATATGTACCTTATCAAACAACTCAATCTGAATCAGAAAAAACGGTTA

TATATCCATTCTGTTTAGAACCAACAACTGCTAGTAGAGCTACTACTGGTATGTACAACGATTTAGGTTTCTCAATTGAG

AAGTCTTGGAGAGGTTACAAACATTGGGACAAATTACAATCATCTTTAAGATTTAGAGGTGAGTATTCTGTTAACACACA

ATACAATCACAATGATGTTGTATCTTACAAACCTTACAAAAGAATATCAACTGGAGAGAAATGGTACAGACACGGTACTG

GCTTATATAGAGCTATAAGAGATAACAAAGGTAGACCACCTCAACACGGTTTCCAAGAACCAACAAGGTCTCCTTTAATG

ACTAAATCTTCGGTTACATCAAACAGACTTACTGGTTATGCTGACCACGAAAATAATAACGAAACAGGTAAAAACTATCC

TCCACATATTCAATCATACCATAATGCTTGGGAATCCTTTGCAGGTATGAACTCGCAAGAACAATGTGCTGGAGTTTGGT

TCCCGAATAGAGGTCCAATCGCTTGGCCGTATAAAGACGGAAGAAGTGAAAATGGTAACATTTACAGATGTCATATGTAC

ATTGATAAGAATGGTGCTGTATGGACAATTGGACACGGTACTTCTGCTTCTAATATGGAAAAAGACCGTTCATCTTCATA

CTTTAGAGAAGTAACCTTTAGATGGAGAGATTTCTACAATTCAGAAAGTAGAAACGAAGGTGGATACAATCATAGAAAAG

GACCTAAATGGTCTCGTTATGATAGAATGAGAACACCAAGAGCTATCCAAATAGAAATGTCTTATGACGCAACAATGATT

CTATTTGATAACGGAGAAATATTCCACGGTGGATATGGTTCACACGGACAACAAGGTACTGGTTATGACGGTGCTCCTGG

TAATGCAATGTCACCTGACGGTGTTGAAGATGTCCACTTCATTAAAATGACAATGAAAATTCAAAACGAAGATTCAATAC

ATACTCCTTGCGCTTTAACAGACGAAGGTGATGTATATGTTTGGGGTTACAATGGTTATGGCGAAGTTGGTGATGGTAGA

ACTCAACACGCATACGGACCAAAAAGAATTCCAAGAGAATGGTTTAATGACGAGAAGATTATAGATATTACTTGCTCAGG

CGGTGATAGTACATCTTTCTATGCTAGAACTTCACAGGATAACATTTATGGTTGGGGAAGAAATAACATAGGTCAATTAG

GAGATACAACAACTACAGACAAATACAGACCAGTATTGATGACAGGATTTAATGCTTCTGATAATGGTGGTATCGCTGTA

TGGCAAGCTTGTTCTCACTCATCTAACTCTTGTTTCCAAATATTAGATGGAAACGGATATATTTGGAGTACAGGTAAAAA

CGATTATGGTAACTTCTTTGATAACTCAACAACTGATAGAAGTACTATGACACAGGCAACTGCTTCTCCTGCTGGAGATA

TAGTTGACTTTTGGGCTTGTAGATGGAACGGATATAGAACAACTTTCGTTAGATTGAAAAATGGAGAGACTTGGACTGCT

GGACACTCTGGAGGTTATTACAATTCAGGTGATGGTGGAACAGGAACAAATACAAGTCCTGTACAAGTAGATAAGATTAA

CAACTTAAAAGAAGTTGCAATTTGCAACACTTATTCTGACCAAGGAAGAAGTTATTGGTTAACTGATAATGGGGAATTCT

TCTGTCAAGGTAGGGATGTTTATTCATCTATGCCTAATCCAATTGCTGGAGATAACTGGAATGGTGAAGATGGAACATAC

AAACCATACCACGCTTATGTACCTGCTGCTACAAGAATAAGAACAATGTGTATTCAAGGTATTGACCAATCAACTAACTA

CTACGGACTTCAACCAATGGTTGGAACGGAAGATGGTCAAGTGCTACTTTGGGGATTTTCTAGTAATAACAACTTGGGAC

ACCACGCAACTGCTACCTGGTCAAACACAGGCAGACCGATGATGTGGCAAGCTGGTAATGGTAGATAAAGTATAAATAGA

AGTATAACAAAAAAAGAAAACGGAGATAAAAACAATGGCAAAAGTAATATATTCAATGACTGCTGGAATTGGTCAAGGTG

ATGATTATACTGCTCCTACAGGAGATACGCCTATTAGTTTAGGAGAGTTAAATGGTAAAGCATACTTCTCTATTGATGAT

GGTAATACTACCATTTCAACTAGTGGTGCAAATGATTCTGTATACGGTGTATCAGTAGTATCAGACGCAGACGAAAAAAC

AGCAATCAAAAATAGTAGCTCTTATGTTGAACAAGGTTTAGATAACCTAGACAACGATTTTATGGCAGGTAAAAATATGA

TAGATTTATTATCAGATGTGGCAGATGACACTTCTGCAACTAAAACTGCTATCGCTGACCATAAAACTGCAAAAGCTAAT

TTTTTGACAAATTTAGGATTTTAATTAAACAAGTATTAGGGAAAGTAATATGGCATTATCAATAACAGATTTTAAAGTAA

CCTGGAGAGGTGCTTGGAGAGATAAGGAAAGCTACAAGAAAAATGATGTAGTTTACTGGAGAGGTAAATCTTATAGATGT

ATTGAAGATACACCTATGAATTATACTATTTCTTCTGAAGCAATGATTAATACTAACTCGTATGGTCAATATCAACCTAC

GATTAGAAAAAGAAGCTATAGACCAGATGACAGAAGATACTGGACATTATTACTAGCAGGTAACGATAACATTGAAACTT

GGCAATATTGGAGACAATATGAAAGAGGCGAAATGGTTAAAGTTGCTGACAAAATTTATCTTTGTTTACAAAGAACAAGA

TATTGTAATACTTGGGTAGAAGAACACGATGGAAGACCATCAAAATATTGGGAACTAATTTACATAAACGAAAACAAGTG

GTGTACAAGAAACGAAGTTGTATCATTTAACAACCGAGCTCCGTTAGGTTGGAGATACAATATGGGAGTTTCACACACAG

GTTGCTCAGACCAATCATATAGAACTTGTACTTTATGTTCAGATGGTTCTGATATGTGGGTTGGTTCTTCTGACAATACC

TCATCTTCAGGATTAGGAGAAGGTACTGCTGGAAATGACGAACCTGCAAAACATATGTCAACAGGTTTCACATTTACAGA

TTGGATGGCGTCAACAGATAATCAATCTTGGAATATTAATGCAACAGGAAGAATGACTACTCCTGATGGTAAAGCTCCAA

GAGTTATCCAAGTTTCAAAAAATTATAACAGAACATATTGGTTGTTTAACAATGGTGAAGTATATGCTTCAGGTAACAAT

GGAAATTATTCTTTAGGAAATTCAGAAACAACAGATAGACCTTATGCAGTTAGGGTAACGGCAAACGATACGCAAGACTG

GCAAGGTAATACAATTGGAAAAACATATAACCAAACTAGAATGGTTAAAGTAGGATTTTCAGACGAGGCACACGATAGTG

GTACTACTTCTAATTGGTCATTAGGATCAGATGGAAGTGTATGGGTTTGGGGTTACAATAACAACGGTCAATTAGGACTT

GGTAATCCTTCAATTAATAACTCAACAGATACTACTGGTGGACCAACTTCAACTGCTTTCTATAGTGCCAATATTCCTAG

ACCAGTAAGATTACCACAATCATATTTTGATGGAAGAAGAATTGTAGATATGTGGTCTTCAGGTTCAGAAGAATGTTGGT

TCCACGCACTTGACGATACAGGTCAACTATGGGCGTGGGGACATAACCAATACGGTGAATTAGGAGTAGGTAACAGAAAT

GGAACTTATTACTATACAAAACCTACAAGAGTTGGAATTAACTGGAACAGATACGGTGGAATAAAATTATACAAATCAAC

TTGGTCAAACGGTGGAAACTCTTCTACACACATTTTAGATGGTGAAGGATATATGTGGTTCACAGGTTATACAACTTCAG

GCGCTTGGCCGATAGGTTCTCCAGGTTATACAGATACGCACCATATTGGTTCGTTCAGAAGAGAAGGTCACTTTATAAAT

GGTGATATTGACTTCTTCTGGTGTGGTGGAGATGAAAACAAATGGTTGTATTTAAGACAAAAATCAACAGGTATGCTATG

GGTACACGATGGTAACTATGGAACTTATGGTGGTCGTGGACAATCAGTAGAAAGTAATGGATACTGGTATTCTTCAGGTG

GTCACCCAGGAAGTTTCATACATCAAAAAGGTCCTAAATGGGCGGTTAATGTATGTGATGTAGGTATGAGTAGAGCTGAT

GGTTCTTATATGTACTCTTTCCCAATGATACTTGATGACGAAGGAATTATATGGGGTGGTGCTCCATATTCAAACGATGA

ACACGGTTTAGGTGGAGACTCAAGTAATAATGACCAATACACTAATGGTGGTCGTAATGACACGCAAGGTGCTATGGAAG

ACAATGAAATGTTTAGAACAAGAAAAAGAATTGTATTCCAACCTGCAGGTGGTCATAGATGGACAGATTTATTCTATTCA

GGAACTGGTTCTTCAAACATACCAAGAGCTCTTAACCAAAGAGGTCAGGTATACTGGTGTGGATATGATGGTGGATCTTC

GGTAACTCAACACTATGACTATTATGGTGAAGGTGCTAATAGTAACCAAACTGCTTACTTCTTCCACTTGGGTCCTAGAG

ACTAACATAAATACTATATTATAGACCTGCTTTACTATGGGTCACTATATAACAACTTGGAGTGAAAAATGAAAGACCTA

GAAACCTTTATTGAAAAGGCACGAAACAACTATGAATCAATGGATTTCATAGTAGATTACAACAACAATAAACTCATCAA

AGAAGTTAAAGGAACTTACTTTTATAATCAATGGTTGGTTATAAATCAGTTAGAACATTTAACTTTAGAAATCACTAAAG

ACTTTCCCGAAATAAACATAAGAGAAAAGCTATATGAGGCATTTAAACAAGAATGGCCTTATGAAGCTGATGATATTTCA

AAACCTTGGGTAGAACCATCAATGATGTATGGTACCGAAGTATGGGTAAATCATTTAAAACCTTTAAAAGATACTCCACA

CAAACTAGTTGCTCAACTATACGCTACTCATAGCGAAATACATAAAAATCAAAAATCTTCTATATTAGTTGACAAGTTAA

AAACTTTATTTGAAAAATATTACAAAGACCATAAAGAAGAAATGTTAGAGGAAGTTAAAATGTCTTGGGATTTTAAAAGA

GGTTTAGTACAAGATTTAATGGCACACCAAGAACATATGGAAGAGGTCTTACCTAGAATTGCTTTATTCAAAATTGGTGC

AAAAGAAATAATGGAAGATAAATCAGGTATTAATAATATGTCTGCTGGTAATAGAGACGAAACAGAAGATATGAAAGTAA

GAGCAGAATTAATGAAAAATGCGGTTACTATGAGAGAAATGGATGTTGATGATTTGCCAGAAGAATATAAAGATTATGTT

AACGAAGATATAAAGGCAGAACAACAAAAGAAAGATGAACTAGATAAAAAATTTAAAGAAGCACCAAAAAGATGAAGACA

TTAAAAGAACTTACTTGGGAACATCATAAAGAAGCTGAACGCCAAGGATTTGTAAAAACAATTATGTCAGGTAAAATAAA

TCCTGAAATATATGGTATCTATCTTTTCAATCAACATCAATGTTATAATATGTTAGAAGCGTTAGCAATGTCAGAAGGTA

TCTTTGATGATATGCCTGAATTGAGACGAGCACCATCTATCAAAGCAGACTTTGATGAATTGTGGACATATAATTGGAAA

CCACCATTGATGGAATCTACAAGTAAATATTTGGATTATATTAATAAAAATTTAATGGATAATCCAGAAAAAATAGCTGC

TCACATTTATGTAAGACATATGGGAGATTTATCTGGTGGTCAAATGATAAGAAAAAAGATACCAGGTCAAGGAAAATATT

ATCAATTTAATATTAGATATGTTGAAGGTAGAAATCAACCATATAAAAACATTAAAGAATTAAAAGAAGCATTAAGAACT

AAAGTGGATAGTTATCAAAAGTATTCAGACCAAAGTACTATATCTGAAAATATTAATAGTGTTGTTTATGAAGCAAGAAT

ATGTTTTGGATTTGCAACAGATTTATTTAAAGATATGAAAAAATTTATTGAACAAAACGAAAAGAGGTTTGGTGATGGCA

ATTTATAAACGAAGTAGAATATGGCAGATGTTAGAAGAAACTACTAACTATCTTACAGCAGTTTTTGATAGAGAAGGTAA

AGAAATATTTGAACCAACTATGGAAAAATTTAATCGTCCAAAAGACGGTTGGGTTAATAGAGTATGGGAAACACCAGAAG

CGAGAAGATGTCATTTAGATGTTGTAGACGCAAGAGGAACAAAAGGTTTATATATGTTTCATTGTTGTGTATTTCCAAAA

CTAACACACCCAGGTCCAATATATGGACTTGATGTTATTGCTGGTGCAAAAAAGGTTACAGGTTTCTTCCACGACTTTTC

TCCACTTGCAAAGAGAGACCATTCAATGGTTGATTGGTTTGTGAAAGAAGCAAGTAATTATAAACCATCTAAAGTACGAG

AACTACCTGATTGGGCAATGAAAATTTTTAGTCCTGGTATGGTTGCTGCTAGTAATATAACACAAGAAAAAGAATTAAAT

GCCGCTTTAAGTTTAGCACAAACTAATTTAGGTGCTTACTTTACATTATTAAGACGAGAAAAAGGAGAAGGAAATATACA

AGAAATAAAAGACGCACAAAACAGATACGCAAAACATCAAAGAGAAAATCCTCATACGCCTAGAGTAATGAAGTCTTTAG

GATTAAAAGATGAAGATGTTGAAGAATTTTGTACAAACGCATTATTTCCTTATGTTGAATAATGGAACATTTAGATAAAT

TTAAACAGGTCATAGACGATTATAAATCAGATGGAAGATATAGAACTTTTAATGATATTATAAGAACAAGAGGAAAGTAT

CCTCACGCCATTTGGTATTCAAAATACTCAATCAAAAATATTGTCAATTGGTGTTCCAACGATTATCTTGGAATGGGACA

ACATAACTATGTCATAGACTCTATGAAAACAGCACTTGAAACGAGCGGAGCGGGTGCTGGAGGGACAAGAAACATATCAG

GCACTACTCACTATCATAATGCTCTGGAACGAGAACTAGCGTCTCTCCATAAGAAAGAAAAAGCATTATTATTTACTTCT

GCTTATAATGCCAATCAAACAACTTTAGAAACAATGGGTAAGGTTATGCCTGATTTATTGTTTATATCAGACGCACAAAA

TCATTCTTCTATCATACAAGGTTTACGCCATAGTAGATGTAGAAAAGAAATATTTAAACATAATGATTTAGATGATTTAG

AAAGTATTTTAAAATCTGAACCAGGTCCTAAATGTGTAGTATTTGAAAGTGTATATTCTATGGACGGAGATATTGCTCCT

GTAAAAGAAATAGCTGACTTATGTAAAAAGTATAATGCAATTTCTTATATTGATGAAGTACACGCTGTTGGTCTTTATGG

AAAAGAAGGTGCTGGAATATGTGAAAGAGATAATGTAGAAGTTGATATAATAAATGGAACATTAGCAAAGGCGTTCGGTG

TACAAGGTGGATACATCGCAGGAAAGAGAGAGTTTATTGACACAATAAGAAGTATGGCTAGTGCTTTTATTTTTACAACT

TCTGTAAGTCCAGTTATTTGTGCTGGTGCTTTAACGAGTGTTAAGTATGTTAGAGACCATCCTGAATTAAGAGATAAGAT

ACACGAAAGAGCAAACAAAACAAAAGAAGAACTTGAAAGACAAGGAATAGAAGTTATGAAAAATGATAGTCATATTGTTC

CTGTTATTATTGGAGAAGCTAAAAGATGTAAAGCAGTATCAGATGAATTACTTTACAAAGAAGGTATCTATGTACAACCT

ATCAATTGGCCAACGGTTGCTGTAGGTACTGAAAGATTAAGATTTACTCCAACTCCATTTCATACAGATAATTTGATATT

TGATATGGTAGTTAAAGTCAAAGCTGCTATTAAAAGATGTGGAAAGAAACTGAATTATGATTGATAAAATTATAGCTGAT

GGTGGAGATGGATTAGATGTCCTAATATATTGTCTAAAACACGAACCTTTTATACAAGGAATTATATTATTTGGTCTGTT

TTTAGCGATATTTTCTTGGTACTATGATAATAAAGTAGATGATAAGGCCGTTTGGTCAAATAACGACCATCTATAAATTA

TAAATATAGCAAAGAATTAGAAGGAAATAACTATGGCTCAACCTAATACAAGACAGACATTAATCTCTTATGCTAAAAGA

GCATTGGGGCATCCTGTTATAGAGATAAATGTTGATGATGACCAAATAGATGATAGAGTAGATGAGGCGCTACAATATTG

GCAACAATATCACTATGATGGTATCAAAAGAACTTATTTAAAATGGCAATATACACAAGCAGAAAAAAATAGAATCTTAA

CTAGTAATAGTGAAGCAGGAACAAAGAATTCTGTAACCTCTACTTGGAAAGAAGATAACAATTATATTGTTGTTCCAGAA

ACCGTATTTTCGGTTACAAATATATTTCCTTTTTCAAACAAAGGTAATTTAAACTTATTTGATGTTAGATACCAATTAAG

ATTAAATGACTTATACGATTTCTCATCAACTTCTGTTATTAACTATGATGTAGTTATGAGACAATTAGATTTCCTAGACC

ACATATTAGTTGGTGAAAAACCATTAAGATTTAACCAACACGATAATAGATTATACATTGATATGGATTGGGAAAACGAT

TTAATGATAGATGAATATATTGTTATTGAATGCTACAGAAAAATGGATCCAGACACATATACAGATGTCTATAATGATAT

TTGGTTAAAGAAATATACAACTGCACTAGTTAAAAAACAATGGGGTGCTAATCTATCAAAATTTGCTGGTGTTGCTATGA

TAGGTGGTGTAACCTTAAATGGTGAACAAATCTATACACAGGCATTAGCAGATATAGAGAAGTTAGAGGAAGAAATAAAA

TCTCTACAAGAACACCAAGCACTAATGATAGGATAAAAATAAAATGGCCGTTAATCATTATTTTCAAGGCGGCGATGGCA

TAGGTAGTCAAAGTGAGAAAAGATTAATAGAAGATTTAATCGTAGAGAATTTAAAAATCTATGGACACGCTGTTTATTAT

TTACCGAGAACTCTAGTTAATAGAGATTTAATTCTTGGTGAGGATTCTGCGTCTAGGTTTGACGACTCGTATCTAGTAGA

AATGTATTTTGACACACCACAAGGGTTTGCTGGTGAAGAAGAAATAATTAGTAAGTTTGGATTAGAAGTAAGAGACGATA

CAACTTTCGTTATTGCTAAAAGAAGATTCCAAGAACAAGTAGATGACCCAGCAAACCTAATGGTGGATGGCAGACCTAAT

GAAGGTGATGTTATTTACTATCCTTTAATGAATAGGTTTTTTGAAATTGCGTTTGTTGAAGACCAGGAACCTTTCTTTCA

ATTAGGAAATTTACCTGTCTATAAATTAAGATGTAAAACATTTGAATACTCTAGTGAAGAATTTAATACAGGTCACGCTG

ACATTGACCAAGCTGATGATAGAAAATCACTTGATACATCTTTGGCACACCAGTTTAGACTTGAAGATGGTACATTAAAT

CAATCTTCTTATAGTGGTTTCTTACAATTAGAAACAGGAGATAAACACGGTAATCCTTGTTATTTAATTAATGAAGATTG

GGACGACACTACAACTGATGGAGACGCTGCTGAAAGTGTACAAACAAAATCTGCTTATGCTGATAATTTAGATTTAGATT

CAGCTGCTGGTTTTGATACTGCAACGGTTAATGATGATATACTTGACTTCACAGAAAACAATCCATTTGGAGAAGTTAAA

TAATGGAAAGAGATAGACATAAACAACTAGTAGAACATACTAATAGAATTAATAAAGAAAAAAAAACTTTAGAGTTATCT

AAAACTTTAAGAAAAGAAGTTGAGATAGGTGCTACAGGCACACAAAAATATAGATTTAAAAAAGGACCTAATAAAGGTAA

GGTAGTATAATGTTTGGAACTCATTTTTATAACGAAGGTATGAGAAGATTGACTATTGCTTTTGGTCAAATCTTTAATAA

GATTGTTGTACAAACAAAAGACGCAAATGGTTCAGTAGTTAAAAGATTTACGGTGCCATTAGCATATGCGCCAAAAGAAA

AATTTATTGTTAGATTAACTCAACAAGGTGATTTAACAGATAAACAATTTGCAACGGTACTACCTCGTATGGGATTTGAA

ATAGAAGGTATAGAATATGACCCTAGTAGAAAGTTAAATAAATTACAAAAATTTAGAAAACCAAACACAGATGGTTCTTC

TACGGATCAAGCTAATAAAATGGACTTTAACTATACTCCAGTTCCATATAATATAACATATAAATTGTTTATATTTACAG

CAACTGCTGAAAATGGTTTACAAATTTTAGAACAAATAGTACCGTACTTTCAACCAGATTATACGGTTACAATTAATATG

GTTCCTGATTTAGGAATTAAGCGTGATGTTCCAATTGTAATTGGAGACATACAATACGAAGATAGTTATAGTGGAGATTT

TGAAACTAGAAGAGCAGTAATATATACTATGACCTTTACTGCTAAAACTTATCTATACGGACCTTCTACAACAGCAGGTG

TTGTTAGAAAAGTACAAACAGATTTAGGAACTGATTCAGTCAGTAAGGCAAGAGAAGAAAGAATAGTAATTACTCCTGAC

CCTACAACAGCAAAACCTGGTGATGATTTTGGATTTACAACAACTATATCATTTTTTGAAGATGGTAAAAAATATGACCC

TTCAAGTGGAAGTGATACATAATGAGAGGATACAATGGACGATATTTTATACAAAGAAAACTGCCTACCAGGTAATGTAG

CAAATAGTTTTCAACACAACATATACAGATTAGGTTATATAATCTCTAAAGATATATTAGACCAACAAATGAGCAATCCA

GGTATTGTTAAAGATGACAATACATTTACTACCGTTCAAATGGTACACCGAATCTATTCACACCTAGACCAAAGACCACA

AGTTAACCCAGGATTAGAACCAATTAAATATGCTTTGAATATAATGGTTGAAGGTTTTGGTTATAAAGTAAAAGATATAT

TAAGATTAAAGTTTAATTGTATGCAACCTCATCCAAATTTCAAAGAAGGTATGTATAACACAGCACACATTGATGACGAA

GAAATGGCTCAACATTGGATTTTAATTTACTATCCAATAGATTGTGATGGTGATACTTATTTGTTTAATGAGAAATTTGA

TAAAACAAAGAAACCAGAAAGACTAACTATACATAAACGAATAACACCAAAAGCAAATAGTTGTGTTATGTTTAGAGGAG

ATAGATTTCACGCAAGTGCTAACCCAATGAAAAGTGAAATGAGAATTATATTAAATTGTAATTTTTCTTTATTAGAGAAT

AAGGATGTGTATAATGAAAATAATAGAGATACAAGTAAAGACCCTTTCAAAGGAACTAGTATAGAAGGTAAAGACTAATG

GGAAAATTAGAAGATAAAGTAAATGATATTTTAGGTATTAAGGAAGAAAGTACTCCTGTCGCTGAATTAATGGTGCAAGA

GAAAAAAGTTCCTGTGCCTAGAAAAGAGGATCCTAAAAAGGACGATATAGATAATGATTACAAATATAGTAGAGAAAACT

ATTATAATTTAATTGAAAGAGGACAAGACGCTATTCAAGGTATATTAGATGTTGCTAAAGAGGGACAACATCCAAGAGCA

TATGAAGTTGCAGGTGTATTAATTAAAAATGTAGCTGACACCGTTGATAAATTACAAGACTTACAAACTAAATTATCAAA

ACTAAAAGAGTTACCTAATAAGACTACTGCTAAGATTCAAAATGCTTTATTTGTTGGGAGTACTACAGACTTGCAAAAGA

TGTTGAAAGATAAAAAAATTGTTAAAACAACTTCTGAAAAAATGCAAGACGATTTAGAACCGATTGTAGTAAACGACAAA

GAGAAAAAAGATGATTAATGACGCATATTTAGGAAATCCAAATCTTAAAAAATCAGGTACTAAAACCGAGTTTACGGAAG

AACAAGTAAATGAGTTTCAAAAATGTTCTGAAGATCCAATCTATTTTATTAAAAATTATGTAAAGATTGTATCGCTTGAT

GAAGGTTTAGTTCCTTTCACAACTTATAAGTTCCAAGATAAGATGATTGATACTATGCACAGCGAAAGGTTTTCAATCTA

CAAACTACCTAGACAAAGTGGTAAATCTACAACTATTATATCTTACTTATTACATTACGCATTATTTAATCCTAATTCAA

GTATAGCTATTCTTGCTAATAAATCTTCAACTGCTAGAGATATATTAGGAAGATTACAACTTGCTTATGAAAACTTACCA

AAGTGGTTACAACAAGGTGTTATCAATTGGAACAAAGGTAATATAGAATTAGAGAATGGAAGTAAACTAGTAGCGGCCGC

AACTTCTTCAAGTGCTGTCCGAGGAGGTTCATATAACATTATCTTCCTTGACGAGTTTGCTTTCGTACCTACAACTATTG

CCGAACAATTTTTTAGTTCCGTTTATCCTACAATTACTTCTGGTAAATCAACTAAAGTAATTATCGTATCAACTCCCCAC

GGAATGAATCAATTCTATAAATTGTGGGTTGACGCTGAGAATGGACAAAATGATTATGTACCAATTGAAGTACATTGGTC

AGAAGTACCAGGTAGAGACAATAAGTGGAAAGAAGAAACAATTAGAAATACATCGGAAGCACAATTTGCTAGTGAGTTTG

AGTGTGAATTTTTAGGTAGTATAGATACATTAATTTCAGCTGCCAAAATAAAAGCGACACCGTATATAACACCATTACAA

ACAAATGGCAGATTAAGTGTCTTTGAAAAACCTATTAAAGGAAACACATATCTATGTACGGTTGATGTTGCCCGAGGTTC

TTTAAAAGATTATTCAGCATTTATTGTTTATGATGTAACCAACTTACCTTATAGAATAGTTGCGACATTTAGAGACAATG

AAATTAAACCTATGTTGTTTCCTGAAATGATTTCTAAAGTATGTAAGCAATATGACAATGCACATATACTTGTTGAAGTA

AATGATATAGGCGCTCAAATTTCAGATGGTTTACATTTTGAAATTGAGTATCCAAATGTATTAATGACTACACAAAAAGG

TCGTGCTGGTCAAATACTTGGTGCGATGTTCAGTCAAAGAGGTTCACAATTAGGTGTTCGTATGACAAAACAGGTAAAGA

AAATGGGTACTGCTAATATCAAATCAATTATAGAGAGTGATAAACT

>lcl|3300006166_____Ga0066836_10000757|480248__Eastern_Pacific_Ocean_-_ETNP201302SV91

CAGTACCAACAGAAATTCCATCAATCACTTGGGCAAATGGTTCTTCTGATCCTGGTAAGATTAAAAAATTATTAGTTGAT

TCTCAACAATCATACCAAAGGGTTGCTATTCTTACAGACAAAGGTAAGATATATTGGTGTGGAAGAAATGAATACGGTTG

GGCGATGATGGGAAATACAACAGATGTTAATACATTTACTCAAATGTCTGGTGGACCTGGAAGCGGAACTAATTCATATT

GCGCTAATATGTGGTTTACTGGAAATGGAAGATACGCAAGTTTCTGGACTAAAGATAGTACAGGTGCTATAAAATGTTGT

GGTTACAATAGTTCTTATGAATTAGGAATTGGAAATAACAACAACCAATCAGCTGCTGTATCACCTAAATGGCAAATTAA

TGGAACAACAACTGCTGATTTAGAAAATATTAAAGACATAGGTTGCAATAGTGAATATGGTAACCAATGGATGTGTAATG

TATGGGTATTAACTTACGATGGATTTATGTTTAATACTGGAAGAAACAACTATGGAATTGGTTGTCAAGGTTATTCTTCT

AGTTATAATGACAGACAATCAACAAACAATATAGAAGAAACAGATGATTATTACTTCCAAATGCAAAGAATGCCGAATTA

TGCACACGGCAGAATCGAAGATGTAAGAGGAAGAGGTTATTACTCAACTGATGGTAACAGATACCACTTTAGAGAAATTA

GAACATTTGATAACAGGTACTTATTATGGGGTTATGGTGGAGATTATATAATGGGACAAAATGATGGAAATTACCACTCA

ACTGCTCAACCACCTGTTCTTGGATAATAAATATAATAGAAGGTAAATAGGAAAACAAAATGGCAAAAATAAATCTCGGT

AGAATTAAACTCCAATTCCAAGGAGAGTTCAATAGAGACCAAATGTACAGAAGAGACGATATTGTCTACCACTCAAATGC

GATGTGGATTATGACAAATGAATATCTTCCAGATGGTTCTAGTGCTTACGCTCCAGGAAGTAAAGTTCAAGGTTACAATG

TAAAAGAAAAAAATATTGTACAAGGTGGACAAGATCCAAACTATAACGGTGCTGACGCATTTAGTTATACACAATATTGG

ACAGAAAACGAAAGAAAAGCTGAAAGACAAAGAACAGATAGAGACGGAAATCCTGTTCAATACAATTCAACTTATGGCTC

TAACGAAGATGGAGCACACGAAATTAATTATAACCAAATAGATACTCAATTAGGAACAATTGTACGCCATCAATCCCACC

TAATGGATGAGTATGACGCTATGTTCCGAGAAACAGAAGACGACTATTCAGAAATGGATACTTACAATGGTTATGAACAA

ACTTATTTTAGATACCACTACAGACCAGTAGACAATACTTTTGATGTTGCTGTTAATGTTTCTGGTGGTGTACCAGATTT

CAAAATTGACAATAGAGTAGGTTCATCTACTAAAGGAAGACAATTTGCTGGTTACAGAAACTTTGAGTTTGTTAAAGAAG

GTCATAGATATGCTTTCGTACAACACAAAAATTCAAACAAATTTTATCCGTTAGGATTTTCTTATACTGCCGATGGTATT

CATAATACTGGAAATACAGGTAAGTCATTAGGACAAGACCACGATGGTCCTTACTATGTTAAAGGTACTGCTTCAAATGG

TGATAGTGGTTTCTTTTCTCCATTATACAAAACTGCTGTTGCTGCTAACGCTGAAGATACAAGACGAGGTGGACAAGGTG

TTTCTCACAAATTAACTTTTGACCAAGGTGATGTACCTGGTTATGAAACAACTTCTGTTGCTTTACAAGGTTGGTTACAC

GATGACGGAACACAAAGAAAAGATACACAAGTTTCTGTTCTAACAGACGCTTCTGATAACACTTACTTACAGGTTACAAA

TGCTTGGGAAGGAACTACTTCTGGTGGTGCTTCTACAAGAACAAGAAAAACAATTTACTTAAACACAGGTGATTCAACTG

AACATCACGCACAAACAGGAACAACTTATTCTTATGTTTATGTTGATGGTGCTTTACAAGTTGGTTCAAATATAACTGCT

GTTATAACACAAAGTAGAGACGATACAACAACTGCTAATAATGGTGCTGGAAGACAAAGACTATTAGTAAACGGAAAACC

TGTTTACCAATTAGTTGCTGAAGCTTCTAATGTTGTTGTTGGTGGTATATCTGGTGCTTATCAGGCAATAGATAATACTG

GTACTGGAACAACTACTGCTTTAGGTTCATCTCCAACTTCTAACGAAGATAAGATTGACCTTTATATGCCTAAACTTACA

GACCCGACTGAAAATGCGGAAAGAACTTTCCAAATGACGGTTGCTTCTAGTAAATTTAATGTAGATGGTGCTGTACCAAC

TGCTAATACGGTTAAATTAGAAGAAGGAAAAACTTATAAGTTTGACCAATCAGATTCTACAAACGCAAGTAAGACATTAA

AATTCTCAACAACTAGTGATGGAACACACGCTAGTGGTTCTGCATACACAACTGGAGTAACCACTTTTGGAACTCCTGGA

AGTGCTGGTGCTCATACAATAATTAAAGTAAGAGCTAATACTGCTAAACTTTATATTTACTGCCACGAAAGTGCTTCTCA

CGGATTTGCAACAGAAACATATGATAGTGCTACAAATTTAGGAAAATCATATGCACCTGCAAACATAATGAAATGGAGAG

GTTTTGGTAAAAACGGATGGGTTAAATATTACCTAGATGGATACCAAGTAGATGAAAATACTTACATTGAGACATTTTTC

AATTCAATTGGAGACAATGATGACCACGAGTACAGACAAAAAATGGAAAATGGCAAATGGAAAGGTGGAAAACAATACAA

CTTTGCAAACAAAGGTGAGAGAACGGTTGAGTTATATGTACCTTATCAAACAACTCAATCTGAATCAGAAAAAACGGTTA

TATATCCATTCTGTTTAGAACCAACAACTGCTAGTAGAGCTACTACTGGTATGTACAACGATTTAGGTTTCTCAATTGAG

AAGTCTTGGAGAGGTTACAAACATTGGGACAAATTACAATCATCTTTAAGATTTAGAGGTGAGTATTCTGTTAACACACA

ATACAATCACAATGATGTTGTATCTTACAAACCTTACAAAAGAATATCAACTGGAGAGAAATGGTACAGACACGGTACTG

GCTTATATAGAGCTATAAGAGATAACAAAGGTAGACCACCTCAACACGGTTTCCAAGAACCAACAAGGTCTCCTTTAATG

ACTAAATCTTCGGTTACATCAAACAGACTTACTGGTTATGCTGACCACGAAAATAATAACGAAACAGGTAAAAACTATCC

TCCACATATTCAATCATACCATAATGCTTGGGAATCCTTTGCAGGTATGAACTCGCAAGAACAATGTGCTGGAGTTTGGT

TCCCGAATAGAGGTCCAATCGCTTGGCCGTATAAAGACGGAAGAAGTGAAAATGGTAACATTTACAGATGTCATATGTAC

ATTGATAAGAATGGTGCTGTATGGACAATTGGACACGGTACTTCTGCTTCTAATATGGAAAAAGACCGTTCATCTTCATA

CTTTAGAGAAGTAACCTTTAGATGGAGAGATTTCTACAATTCAGAAAGTAGAAACGAAGGTGGATACAATCATAGAAAAG

GACCTAAATGGTCTCGTTATGATAGAATGAGAACACCAAGAGCTATCCAAATAGAAATGTCTTATGACGCAACAATGATT

CTATTTGATAACGGAGAAATATTCCACGGTGGATATGGTTCACACGGACAACAAGGTACTGGTTATGACGGTGCTCCTGG

TAATGCAATGTCACCTGACGGTGTTGAAGATGTCCACTTCATTAAAATGACAATGAAAATTCAAAACGAAGATTCAATAC

ATACTCCTTGCGCTTTAACAGACGAAGGTGATGTATATGTTTGGGGTTACAATGGTTATGGCGAAGTTGGTGATGGTAGA

ACTCAACACGCATACGGACCAAAAAGAATTCCAAGAGAATGGTTTAATGACGAGAAGATTATAGATATTACTTGCTCAGG

CGGTGATAGTACATCTTTCTATGCTAGAACTTCACAGGATAACATTTATGGTTGGGGAAGAAATAACATAGGTCAATTAG

GAGATACAACAACTACAGACAAATACAGACCAGTATTGATGACAGGATTTAATGCTTCTGATAATGGTGGTATCGCTGTA

TGGCAAGCTTGTTCTCACTCATCTAACTCTTGTTTCCAAATATTAGATGGAAACGGATATATTTGGAGTACAGGTAAAAA

CGATTATGGTAACTTCTTTGATAACTCAACAACTGATAGAAGTACTATGACACAGGCAACTGCTTCTCCTGCTGGAGATA

TAGTTGACTTTTGGGCTTGTAGATGGAACGGATATAGAACAACTTTCGTTAGATTGAAAAATGGAGAGACTTGGACTGCT

GGACACTCTGGAGGTTATTACAATTCAGGTGATGGTGGAACAGGAACAAATACAAGTCCTGTACAAGTAGATAAGATTAA

CAACTTAAAAGAAGTTGCAATTTGCAACACTTATTCTGACCAAGGAAGAAGTTATTGGTTAACTGATAATGGGGAATTCT

TCTGTCAAGGTAGGGATGTTTATTCATCTATGCCTAATCCAATTGCTGGAGATAACTGGAATGGTGAAGATGGAACATAC

AAACCATACCACGCTTATGTACCTGCTGCTACAAGAATAAGAACAATGTGTATTCAAGGTATTGACCAATCAACTAACTA

CTACGGACTTCAACCAATGGTTGGAACGGAAGATGGTCAAGTGCTACTTTGGGGATTTTCTAGTAATAACAACTTGGGAC

ACCACGCAACTGCTACCTGGTCAAACACAGGCAGACCGATGATGTGGCAAGCTGGTAATGGTAGATAAAGTATAAATAGA

AGTATAACAAAAAAAGAAAACGGAGATAAAAACAATGGCAAAAGTAATATATTCAATGACTGCTGGAATTGGTCAAGGTG

ATGATTATACTGCTCCTACAGGAGATACGCCTATTAGTTTAGGAGAGTTAAATGGTAAAGCATACTTCTCTATTGATGAT

GGTAATACTACCATTTCAACTAGTGGTGCAAATGATTCTGTATACGGTGTATCAGTAGTATCAGACGCAGACGAAAAAAC

AGCAATCAAAAATAGTAGCTCTTATGTTGAACAAGGTTTAGATAACCTAGACAACGATTTTATGGCAGGTAAAAATATGA

TAGATTTATTATCAGATGTGGCAGATGACACTTCTGCAACTAAAACTGCTATCGCTGACCATAAAACTGCAAAAGCTAAT

TTTTTGACAAATTTAGGATTTTAATTAAACAAGTATTAGGGAAAGTAATATGGCATTATCAATAACAGATTTTAAAGTAA

CCTGGAGAGGTGCTTGGAGAGATAAGGAAAGCTACAAGAAAAATGATGTAGTTTACTGGAGAGGTAAATCTTATAGATGT

ATTGAAGATACACCTATGAATTATACTATTTCTTCTGAAGCAATGATTAATACTAACTCGTATGGTCAATATCAACCTAC

GATTAGAAAAAGAAGCTATAGACCAGATGACAGAAGATACTGGACATTATTACTAGCAGGTAACGATAACATTGAAACTT

GGCAATATTGGAGACAATATGAAAGAGGCGAAATGGTTAAAGTTGCTGACAAAATTTATCTTTGTTTACAAAGAACAAGA

TATTGTAATACTTGGGTAGAAGAACACGATGGAAGACCATCAAAATATTGGGAACTAATTTACATAAACGAAAACAAGTG

GTGTACAAGAAACGAAGTTGTATCATTTAACAACCGAGCTCCGTTAGGTTGGAGATACAATATGGGAGTTTCACACACAG

GTTGCTCAGACCAATCATATAGAACTTGTACTTTATGTTCAGATGGTTCTGATATGTGGGTTGGTTCTTCTGACAATACC

TCATCTTCAGGATTAGGAGAAGGTACTGCTGGAAATGACGAACCTGCAAAACATATGTCAACAGGTTTCACATTTACAGA

TTGGATGGCGTCAACAGATAATCAATCTTGGAATATTAATGCAACAGGAAGAATGACTACTCCTGATGGTAAAGCTCCAA

GAGTTATCCAAGTTTCAAAAAATTATAACAGAACATATTGGTTGTTTAACAATGGTGAAGTATATGCTTCAGGTAACAAT

GGAAATTATTCTTTAGGAAATTCAGAAACAACAGATAGACCTTATGCAGTTAGGGTAACGGCAAACGATACGCAAGACTG

GCAAGGTAATACAATTGGAAAAACATATAACCAAACTAGAATGGTTAAAGTAGGATTTTCAGACGAGGCACACGATAGTG

GTACTACTTCTAATTGGTCATTAGGATCAGATGGAAGTGTATGGGTTTGGGGTTACAATAACAACGGTCAATTAGGACTT

GGTAATCCTTCAATTAATAACTCAACAGATACTACTGGTGGACCAACTTCAACTGCTTTCTATAGTGCCAATATTCCTAG

ACCAGTAAGATTACCACAATCATATTTTGATGGAAGAAGAATTGTAGATATGTGGTCTTCAGGTTCAGAAGAATGTTGGT

TCCACGCACTTGACGATACAGGTCAACTATGGGCGTGGGGACATAACCAATACGGTGAATTAGGAGTAGGTAACAGAAAT

GGAACTTATTACTATACAAAACCTACAAGAGTTGGAATTAACTGGAACAGATACGGTGGAATAAAATTATACAAATCAAC

TTGGTCAAACGGTGGAAACTCTTCTACACACATTTTAGATGGTGAAGGATATATGTGGTTCACAGGTTATACAACTTCAG

GCGCTTGGCCGATAGGTTCTCCAGGTTATACAGATACGCACCATATTGGTTCGTTCAGAAGAGAAGGTCACTTTATAAAT

GGTGATATTGACTTCTTCTGGTGTGGTGGAGATGAAAACAAATGGTTGTATTTAAGACAAAAATCAACAGGTATGCTATG

GGTACACGATGGTAACTATGGAACTTATGGTGGTCGTGGACAATCAGTAGAAAGTAATGGATACTGGTATTCTTCAGGTG

GTCACCCAGGAAGTTTCATACATCAAAAAGGTCCTAAATGGGCGGTTAATGTATGTGATGTAGGTATGAGTAGAGCTGAT

GGTTCTTATATGTACTCTTTCCCAATGATACTTGATGACGAAGGAATTATATGGGGTGGTGCTCCATATTCAAACGATGA

ACACGGTTTAGGTGGAGACTCAAGTAATAATGACCAATACACTAATGGTGGTCGTAATGACACGCAAGGTGCTATGGAAG

ACAATGAAATGTTTAGAACAAGAAAAAGAATTGTATTCCAACCTGCAGGTGGTCATAGATGGACAGATTTATTCTATTCA

GGAACTGGTTCTTCAAACATACCAAGAGCTCTTAACCAAAGAGGTCAGGTATACTGGTGTGGATATGATGGTGGATCTTC

GGTAACTCAACACTATGACTATTATGGTGAAGGTGCTAATAGTAACCAAACTGCTTACTTCTTCCACTTGGGTCCTAGAG

ACTAACATAAATACTATATTATAGACCTGCTTTACTATGGGTCACTATATAACAACTTGGAGTGAAAAATGAAAGACCTA

GAAACCTTTATTGAAAAGGCACGAAACAACTATGAATCAATGGATTTCATAGTAGATTACAACAACAATAAACTCATCAA

AGAAGTTAAAGGAACTTACTTTTATAATCAATGGTTGGTTATAAATCAGTTAGAACATTTAACTTTAGAAATCACTAAAG

ACTTTCCCGAAATAAACATAAGAGAAAAGCTATATGAGGCATTTAAACAAGAATGGCCTTATGAAGCTGATGATATTTCA

AAACCTTGGGTAGAACCATCAATGATGTATGGTACCGAAGTATGGGTAAATCATTTAAAACCTTTAAAAGATACTCCACA

CAAACTAGTTGCTCAACTATACGCTACTCATAGCGAAATACATAAAAATCAAAAATCTTCTATATTAGTTGACAAGTTAA

AAACTTTATTTGAAAAATATTACAAAGACCATAAAGAAGAAATGTTAGAGGAAGTTAAAATGTCTTGGGATTTTAAAAGA

GGTTTAGTACAAGATTTAATGGCACACCAAGAACATATGGAAGAGGTCTTACCTAGAATTGCTTTATTCAAAATTGGTGC

AAAAGAAATAATGGAAGATAAATCAGGTATTAATAATATGTCTGCTGGTAATAGAGACGAAACAGAAGATATGAAAGTAA

GAGCAGAATTAATGAAAAATGCGGTTACTATGAGAGAAATGGATGTTGATGATTTGCCAGAAGAATATAAAGATTATGTT

AACGAAGATATAAAGGCAGAACAACAAAAGAAAGATGAACTAGATAAAAAATTTAAAGAAGCACCAAAAAGATGAAGACA

TTAAAAGAACTTACTTGGGAACATCATAAAGAAGCTGAACGCCAAGGATTTGTAAAAACAATTATGTCAGGTAAAATAAA

TCCTGAAATATATGGTATCTATCTTTTCAATCAACATCAATGTTATAATATGTTAGAAGCGTTAGCAATGTCAGAAGGTA

TCTTTGATGATATGCCTGAATTGAGACGAGCACCATCTATCAAAGCAGACTTTGATGAATTGTGGACATATAATTGGAAA

CCACCATTGATGGAATCTACAAGTAAATATTTGGATTATATTAATAAAAATTTAATGGATAATCCAGAAAAAATAGCTGC

TCACATTTATGTAAGACATATGGGAGATTTATCTGGTGGTCAAATGATAAGAAAAAAGATACCAGGTCAAGGAAAATATT

ATCAATTTAATATTAGATATGTTGAAGGTAGAAATCAACCATATAAAAACATTAAAGAATTAAAAGAAGCATTAAGAACT

AAAGTGGATAGTTATCAAAAGTATTCAGACCAAAGTACTATATCTGAAAATATTAATAGTGTTGTTTATGAAGCAAGAAT

ATGTTTTGGATTTGCAACAGATTTATTTAAAGATATGAAAAAATTTATTGAACAAAACGAAAAGAGGTTTGGTGATGGCA

ATTTATAAACGAAGTAGAATATGGCAGATGTTAGAAGAAACTACTAACTATCTTACAGCAGTTTTTGATAGAGAAGGTAA

AGAAATATTTGAACCAACTATGGAAAAATTTAATCGTCCAAAAGACGGTTGGGTTAATAGAGTATGGGAAACACCAGAAG

CGAGAAGATGTCATTTAGATGTTGTAGACGCAAGAGGAACAAAAGGTTTATATATGTTTCATTGTTGTGTATTTCCAAAA

CTAACACACCCAGGTCCAATATATGGACTTGATGTTATTGCTGGTGCAAAAAAGGTTACAGGTTTCTTCCACGACTTTTC

TCCACTTGCAAAGAGAGACCATTCAATGGTTGATTGGTTTGTGAAAGAAGCAAGTAATTATAAACCATCTAAAGTACGAG

AACTACCTGATTGGGCAATGAAAATTTTTAGTCCTGGTATGGTTGCTGCTAGTAATATAACACAAGAAAAAGAATTAAAT

GCCGCTTTAAGTTTAGCACAAACTAATTTAGGTGCTTACTTTACATTATTAAGACGAGAAAAAGGAGAAGGAAATATACA

AGAAATAAAAGACGCACAAAACAGATACGCAAAACATCAAAGAGAAAATCCTCATACGCCTAGAGTAATGAAGTCTTTAG

GATTAAAAGATGAAGATGTTGAAGAATTTTGTACAAACGCATTATTTCCTTATGTTGAATAATGGAACATTTAGATAAAT

TTAAACAGGTCATAGACGATTATAAATCAGATGGAAGATATAGAACTTTTAATGATATTATAAGAACAAGAGGAAAGTAT

CCTCACGCCATTTGGTATTCAAAATACTCAATCAAAAATATTGTCAATTGGTGTTCCAACGATTATCTTGGAATGGGACA

ACATAACTATGTCATAGACTCTATGAAAACAGCACTTGAAACGAGCGGAGCGGGTGCTGGAGGGACAAGAAACATATCAG

GCACTACTCACTATCATAATGCTCTGGAACGAGAACTAGCGTCTCTCCATAAGAAAGAAAAAGCATTATTATTTACTTCT

GCTTATAATGCCAATCAAACAACTTTAGAAACAATGGGTAAGGTTATGCCTGATTTATTGTTTATATCAGACGCACAAAA

TCATTCTTCTATCATACAAGGTTTACGCCATAGTAGATGTAGAAAAGAAATATTTAAACATAATGATTTAGATGATTTAG

AAAGTATTTTAAAATCTGAACCAGGTCCTAAATGTGTAGTATTTGAAAGTGTATATTCTATGGACGGAGATATTGCTCCT

GTAAAAGAAATAGCTGACTTATGTAAAAAGTATAATGCAATTTCTTATATTGATGAAGTACACGCTGTTGGTCTTTATGG

AAAAGAAGGTGCTGGAATATGTGAAAGAGATAATGTAGAAGTTGATATAATAAATGGAACATTAGCAAAGGCGTTCGGTG

TACAAGGTGGATACATCGCAGGAAAGAGAGAGTTTATTGACACAATAAGAAGTATGGCTAGTGCTTTTATTTTTACAACT

TCTGTAAGTCCAGTTATTTGTGCTGGTGCTTTAACGAGTGTTAAGTATGTTAGAGACCATCCTGAATTAAGAGATAAGAT

ACACGAAAGAGCAAACAAAACAAAAGAAGAACTTGAAAGACAAGGAATAGAAGTTATGAAAAATGATAGTCATATTGTTC

CTGTTATTATTGGAGAAGCTAAAAGATGTAAAGCAGTATCAGATGAATTACTTTACAAAGAAGGTATCTATGTACAACCT

ATCAATTGGCCAACGGTTGCTGTAGGTACTGAAAGATTAAGATTTACTCCAACTCCATTTCATACAGATAATTTGATATT

TGATATGGTAGTTAAAGTCAAAGCTGCTATTAAAAGATGTGGAAAGAAACTGAATTATGATTGATAAAATTATAGCTGAT

GGTGGAGATGGATTAGATGTCCTAATATATTGTCTAAAACACGAACCTTTTATACAAGGAATTATATTATTTGGTCTGTT

TTTAGCGATATTTTCTTGGTACTATGATAATAAAGTAGATGATAAGGCCGTTTGGTCAAATAACGACCATCTATAAATTA

TAAATATAGCAAAGAATTAGAAGGAAATAACTATGGCTCAACCTAATACAAGACAGACATTAATCTCTTATGCTAAAAGA

GCATTGGGGCATCCTGTTATAGAGATAAATGTTGATGATGACCAAATAGATGATAGAGTAGATGAGGCGCTACAATATTG

GCAACAATATCACTATGATGGTATCAAAAGAACTTATTTAAAATGGCAATATACACAAGCAGAAAAAAATAGAATCTTAA

CTAGTAATAGTGAAGCAGGAACAAAGAATTCTGTAACCTCTACTTGGAAAGAAGATAACAATTATATTGTTGTTCCAGAA

ACCGTATTTTCGGTTACAAATATATTTCCTTTTTCAAACAAAGGTAATTTAAACTTATTTGATGTTAGATACCAATTAAG

ATTAAATGACTTATACGATTTCTCATCAACTTCTGTTATTAACTATGATGTAGTTATGAGACAATTAGATTTCCTAGACC

ACATATTAGTTGGTGAAAAACCATTAAGATTTAACCAACACGATAATAGATTATACATTGATATGGATTGGGAAAACGAT

TTAATGATAGATGAATATATTGTTATTGAATGCTACAGAAAAATGGATCCAGACACATATACAGATGTCTATAATGATAT

TTGGTTAAAGAAATATACAACTGCACTAGTTAAAAAACAATGGGGTGCTAATCTATCAAAATTTGCTGGTGTTGCTATGA

TAGGTGGTGTAACCTTAAATGGTGAACAAATCTATACACAGGCATTAGCAGATATAGAGAAGTTAGAGGAAGAAATAAAA

TCTCTACAAGAACACCAAGCACTAATGATAGGATAAAAATAAAATGGCCGTTAATCATTATTTTCAAGGCGGCGATGGCA

TAGGTAGTCAAAGTGAGAAAAGATTAATAGAAGATTTAATCGTAGAGAATTTAAAAATCTATGGACACGCTGTTTATTAT

TTACCGAGAACTCTAGTTAATAGAGATTTAATTCTTGGTGAGGATTCTGCGTCTAGGTTTGACGACTCGTATCTAGTAGA

AATGTATTTTGACACACCACAAGGGTTTGCTGGTGAAGAAGAAATAATTAGTAAGTTTGGATTAGAAGTAAGAGACGATA

CAACTTTCGTTATTGCTAAAAGAAGATTCCAAGAACAAGTAGATGACCCAGCAAACCTAATGGTGGATGGCAGACCTAAT

GAAGGTGATGTTATTTACTATCCTTTAATGAATAGGTTTTTTGAAATTGCGTTTGTTGAAGACCAGGAACCTTTCTTTCA

ATTAGGAAATTTACCTGTCTATAAATTAAGATGTAAAACATTTGAATACTCTAGTGAAGAATTTAATACAGGTCACGCTG

ACATTGACCAAGCTGATGATAGAAAATCACTTGATACATCTTTGGCACACCAGTTTAGACTTGAAGATGGTACATTAAAT

CAATCTTCTTATAGTGGTTTCTTACAATTAGAAACAGGAGATAAACACGGTAATCCTTGTTATTTAATTAATGAAGATTG

GGACGACACTACAACTGATGGAGACGCTGCTGAAAGTGTACAAACAAAATCTGCTTATGCTGATAATTTAGATTTAGATT

CAGCTGCTGGTTTTGATACTGCAACGGTTAATGATGATATACTTGACTTCACAGAAAACAATCCATTTGGAGAAGTTAAA

TAATGGAAAGAGATAGACATAAACAACTAGTAGAACATACTAATAGAATTAATAAAGAAAAAAAAACTTTAGAGTTATCT

AAAACTTTAAGAAAAGAAGTTGAGATAGGTGCTACAGGCACACAAAAATATAGATTTAAAAAAGGACCTAATAAAGGTAA

GGTAGTATAATGTTTGGAACTCATTTTTATAACGAAGGTATGAGAAGATTGACTATTGCTTTTGGTCAAATCTTTAATAA

GATTGTTGTACAAACAAAAGACGCAAATGGTTCAGTAGTTAAAAGATTTACGGTGCCATTAGCATATGCGCCAAAAGAAA

AATTTATTGTTAGATTAACTCAACAAGGTGATTTAACAGATAAACAATTTGCAACGGTACTACCTCGTATGGGATTTGAA

ATAGAAGGTATAGAATATGACCCTAGTAGAAAGTTAAATAAATTACAAAAATTTAGAAAACCAAACACAGATGGTTCTTC

TACGGATCAAGCTAATAAAATGGACTTTAACTATACTCCAGTTCCATATAATATAACATATAAATTGTTTATATTTACAG

CAACTGCTGAAAATGGTTTACAAATTTTAGAACAAATAGTACCGTACTTTCAACCAGATTATACGGTTACAATTAATATG

GTTCCTGATTTAGGAATTAAGCGTGATGTTCCAATTGTAATTGGAGACATACAATACGAAGATAGTTATAGTGGAGATTT

TGAAACTAGAAGAGCAGTAATATATACTATGACCTTTACTGCTAAAACTTATCTATACGGACCTTCTACAACAGCAGGTG

TTGTTAGAAAAGTACAAACAGATTTAGGAACTGATTCAGTCAGTAAGGCAAGAGAAGAAAGAATAGTAATTACTCCTGAC

CCTACAACAGCAAAACCTGGTGATGATTTTGGATTTACAACAACTATATCATTTTTTGAAGATGGTAAAAAATATGACCC

TTCAAGTGGAAGTGATACATAATGAGAGGATACAATGGACGATATTTTATACAAAGAAAACTGCCTACCAGGTAATGTAG

CAAATAGTTTTCAACACAACATATACAGATTAGGTTATATAATCTCTAAAGATATATTAGACCAACAAATGAGCAATCCA

GGTATTGTTAAAGATGACAATACATTTACTACCGTTCAAATGGTACACCGAATCTATTCACACCTAGACCAAAGACCACA

AGTTAACCCAGGATTAGAACCAATTAAATATGCTTTGAATATAATGGTTGAAGGTTTTGGTTATAAAGTAAAAGATATAT

TAAGATTAAAGTTTAATTGTATGCAACCTCATCCAAATTTCAAAGAAGGTATGTATAACACAGCACACATTGATGACGAA

GAAATGGCTCAACATTGGATTTTAATTTACTATCCAATAGATTGTGATGGTGATACTTATTTGTTTAATGAGAAATTTGA

TAAAACAAAGAAACCAGAAAGACTAACTATACATAAACGAATAACACCAAAAGCAAATAGTTGTGTTATGTTTAGAGGAG

ATAGATTTCACGCAAGTGCTAACCCAATGAAAAGTGAAATGAGAATTATATTAAATTGTAATTTTTCTTTATTAGAGAAT

AAGGATGTGTATAATGAAAATAATAGAGATACAAGTAAAGACCCTTTCAAAGGAACTAGTATAGAAGGTAAAGACTAATG

GGAAAATTAGAAGATAAAGTAAATGATATTTTAGGTATTAAGGAAGAAAGTACTCCTGTCGCTGAATTAATGGTGCAAGA

GAAAAAAGTTCCTGTGCCTAGAAAAGAGGATCCTAAAAAGGACGATATAGATAATGATTACAAATATAGTAGAGAAAACT

ATTATAATTTAATTGAAAGAGGACAAGACGCTATTCAAGGTATATTAGATGTTGCTAAAGAGGGACAACATCCAAGAGCA

TATGAAGTTGCAGGTGTATTAATTAAAAATGTAGCTGACACCGTTGATAAATTACAAGACTTACAAACTAAATTATCAAA

ACTAAAAGAGTTACCTAATAAGACTACTGCTAAGATTCAAAATGCTTTATTTGTTGGGAGTACTACAGACTTGCAAAAGA

TGTTGAAAGATAAAAAAATTGTTAAAACAACTTCTGAAAAAATGCAAGACGATTTAGAACCGATTGTAGTAAACGACAAA

GAGAAAAAAGATGATTAATGACGCATATTTAGGAAATCCAAATCTTAAAAAATCAGGTACTAAAACCGAGTTTACGGAAG

AACAAGTAAATGAGTTTCAAAAATGTTCTGAAGATCCAATCTATTTTATTAAAAATTATGTAAAGATTGTATCGCTTGAT

GAAGGTTTAGTTCCTTTCACAACTTATAAGTTCCAAGATAAGATGATTGATACTATGCACAGCGAAAGGTTTTCAATCTA

CAAACTACCTAGACAAAGTGGTAAATCTACAACTATTATATCTTACTTATTACATTACGCATTATTTAATCCTAATTCAA

GTATAGCTATTCTTGCTAATAAATCTTCAACTGCTAGAGATATATTAGGAAGATTACAACTTGCTTATGAAAACTTACCA

AAGTGGTTACAACAAGGTGTTATCAATTGGAACAAAGGTAATATAGAATTAGAGAATGGAAGTAAACTAGTAGCGGCCGC

AACTTCTTCAAGTGCTGTCCGAGGAGGTTCATATAACATTATCTTCCTTGACGAGTTTGCTTTCGTACCTACAACTATTG

CCGAACAATTTTTTAGTTCCGTTTATCCTACAATTACTTCTGGTAAATCAACTAAAGTAATTATCGTATCAACTCCCCAC

GGAATGAATCAATTCTATAAATTGTGGGTTGACGCTGAGAATGGACAAAATGATTATGTACCAATTGAAGTACATTGGTC

AGAAGTACCAGGTAGAGACAATAAGTGGAAAGAAGAAACAATTAGAAATACATCGGAAGCACAATTTGCTAGTGAGTTTG

AGTGTGAATTTTTAGGTAGTATAGATACATTAATTTCAGCTGCCAAAATAAAAGCGACACCGTATATAACACCATTACAA

ACAAATGGCAGATTAAGTGTCTTTGAAAAACCTATTAAAGGAAACACATATCTATGTACGGTTGATGTTGCCCGAGGTTC

TTTAAAAGATTATTCAGCATTTATTGTTTATGATGTAACCAACTTACCTTATAGAATAGTTGCGACATTTAGAGACAATG

AAATTAAACCTATGTTGTTTCCTGAAATGATTTCTAAAGTATGTAAGCAATATGACAATGCACATATACTTGTTGAAGTA

AATGATATAGGCGCTCAAATTTCAGATGGTTTACATTTTGAAATTGAGTATCCAAATGTATTAATGACTACACAAAAAGG

TCGTGCTGGTCAAATACTTGGTGCGATGTTCAGTCAAAGAGGTTCACAATTAGGTGTTCGTATGACAAAACAGGTAAAGA

AAATGGGTACTGCTAATATCAAATCAATTATAGAGAGTGATAAACT

>lcl|3300006306_____Ga0068469_1063893|485779_ __Station_ALOHA_-_HOT229_1_0500m

TGTCAATTAATCCAGAAACAAAAGAATTAACAATCGTTGGCGGAGCTGGCGGTGATGAAGCGGCTGCTATAACTGAAAGA

GAAACAAAAGCTGATGAAGAAGCAGTTGTACTAGCTAAAAAAGCAGAAGACTTATTAAAAGCAGAAGTTGCAGAAACTAA

AAGACTTCAAGACGCTGGTCTAGCTTAATTTATGTAGTGATTTTATTATGATAAGTATTATATTATGTATGACATCAAAG

AATTAACCAAAGATATACACCAAAACGCTGAAAGACAAGAGTTTGTCAAAACTCTTATGTCAGGTTCTATTGAACCTAGA

CTTTATGCAACCTATCTTTACAATCAATTACAATGTTATGCTATATTAGAAAAATATGGAATAGAAAATTCTCTATTTCG

TACAACTCCTAATTTACCTAGAGCAGAACATTTACATTATGATTTTAAAGCATTATGGACAAGTGAAGACCTTCCAACAG

TAACTCAAAGTACTAAAGATTATGTTGCTCATATTGAAACAATCAAAGAAGACGCAGAAAAATTATACGGTCATATCTAT

ACTAGACATTTAGGAGATGTATCTGGTGGTCAAATGATAATGAAAAGAACACCAGGACCTAATCGTTATTACAAGTTTAA

ACATAAAGAAATAAAAGAGTATAAACGAATAGTAAGAGAAATGATAAACAGTTATTTAAATGTTTATAAACTTAATATTC

TAAATGAAGTTAAATTTTGTTTTGCAACTGCTACACAATTGTTCAAAGAAATGAACGATATGGATTATTCAAAACCTTTA

ATTTTAACTAACGAGGTTAAAGATGATTTGGGAACGACTAATTAAATTAGAAAAAGAAATAATCGCTATACTTGATAGAC

GTTGTAAAGAATACAACGAAGAGGGTATGGATAGATTTAATAATGATACTTGGACCAACCGTACTTGGTCTAATATGAGT

GTAAGACGTGCTCACGTAGACGTAGTGGACGCCAGAGAAACAAAAGGTCTTTGGATGGCACACATATGTTTATTTCCAAA

TTTAACTAACGGAGGTCCAATTTATGGATTTGATGTTATTGCAGGTAAGAAAAAGATAACAGGTGTCTTTCACGATTTTA

GTCCACTATTATTAAAAGACCATCCCTTAACAAAGTATTTTATAGAAGAGAATAAATGGTTTAAACCATCTAAAGAAAGA

GAATTGCCTGATTGGGCAAAGGCTATCTTTAGTCCTGGTATGATTGCTGCTGGTAGAGTAACAGAAGAAAAAGAATTAAA

CCAAATATGTACTCTTGCTACGTCTAATTTAGAAAATTATCTTGACAAAATTGGTCATTATAATAGCGATTCAAAGGAAG

AAGATGTAATAAGAGCGCAAAACTTCTATTGCGAACACCAACAACAAAATCCACACACCCCTAGAGTAATGAAAACTCTT

GGACTGCCTGAAGATGATATAAAACTATTCTGTACTGATAATTTGTTTCCGAAGATATAATTGTTATTATAAATATACAA

TAAAGGAACCAGTATGGCAGAACCAGCATCCAGAGAAACAGTAAAACAATACGCTTTAAGAGCATTAGGTAAACCAGTAA

TTGAAATCAACGTTGATGACGACCAACTGGAAGATAGACTTGATGAAGCATTACAATATTTTGCTCAATACCACTATGAT

GGTGTTAAAAGAACCTATTTAAAATACAAGTATACAGCGGCAGATAAAGCTAGAATTTTAGCAGATAGTACTGAAACTGA

ATCTAAAACATATGGTGATTCTTCTGTAGTAAATACAGAATGGAAAGAAGGCAATCAGTATATTGTATGTCCTGAATCTG

TTATATCTGTAATTAACATTTTTCCATTTTCAAATAAAGGTAATTTAAATTTATTTGATGTTAGATATCAATTAAGATTA

AATGACCTATATGATTTTTCTTCAACGTCTGTTATTAACTATGATGTTGTATTAAGACATTTAGATTTTTTAGACCATAT

ATTAGTCGGAGAAAAACCTTATAGATTTAACCAATTAGATAATAGACTTTATGTTGATATGGATTGGAAAAATGATTTAC

AAGTAGATGAATTTCTTGTAATAGAATGCTGGAGAAAATTAGACCCTAACACATATACAGATGTCTTTAATGATATTTGG

TTAAAAAGATACGTAACTGCTTTATTTAAAAAACAATGGGGAGCCAATTTAAGTAAATTTGATGGAGTTGCAATGATTGG

TGGAGTTACATTAAACGGTAAACAAATTTATAGTGAAGCACTAGAAGATTTGGATAAATTGGAAATAAAATTAAGAAGCG

AGTTTGAAGAACCGCAACCTTTTATGATAGGATAATGCTATGCCAGTTAATCATTACTTTCAAGGTGGAAAAGGCATAGG

TAATGCTGCCGAACAAAGACTACACGAAGATATAATAGTTGAAGGTCTTAAAATTTACGGTCAGGATGTCTATTACTTAC

CACGAACATTAGTCAATAAAGATTTAATACTAGGAGAAGATGTATCTAGTAGATTTGATGATTCTTATTTGATAGAAATG

TATTTTGAAAATAATACAGGATTTGCTGGTGAACAAGAAATCATAAGTAAGTTTGGATTAGAAATTCGTGATGATACATC

ATTAATGGTTTCAAAAAGAAGTTGGACAAATTTAGTTGGTAATAAGGCAACACAGGTTGGTTCTTCTTTATCAGTTACAG

GAAGACCAAACGAAGGTGATATTATATATGTGCCTTTGATGAAATCTTTTTTTGAAATTTTATTTGTAGAAGACCAAGAA

CCATTTTTCCAATTAGGCAATCTGCCAGTTTATAAATTAAAAGTAACTCGTTGGGAGTATGCAAGTGAAAAACTTGATAC

TGGTATATCTACTATTGACCAACACGAAGATACACATACACTAGACCAATTAGCATATAAGTTTACTTTAGAATATGGAC

AAGAAGTTATGACAGGTAAAGGTTCAGTACAATTAGAAAGTTATCACGATTATTCAACTGGTCAACCAGCACTTTTAATG

AACGAAGATTTTACAGAGTCTAATATACAGACACAATCTCCATATGCAGATAATTTAGACTTGAATAAAGAGGCAGGATA

TGATACTGTTTCAACAGCGGATGATATACTTGACTTTACAGAAAGAAATCCTTTTGGGGAGATAGATGAATAATGTTTGG

AACACACTTTTATAATCAAAGTTTAAGAAGACTAACTATTGCATTTGGACAGATTTTTAATAATATAATTATTCAACAAA

AATCTGGTACAGGTGCTATTACTAAAAGAATACGTGTGCCTTTAGCATACGCTCCTAAAGAAAAGTTTATAGCCAGAATA

GACCAACAAGCAAGTTTAGAAAAAGGTAAAACGTTTGCTATTGTATTACCTAGAATGGGATTTGAATTAACAGGTTTAAA

GTATGACGCCACTAGAAAACTAAACAAACTTCAAAAAACAGTTAGAGTTAAAACTTCTGATTCTACTGTACATAATTTTA

ATTATTCACCAGTACCCTATGATATAAGTTTTAGTCTTTATTCTTTTACGGCTACAGCAGAAAATGGACTACAGATAATT

GAACAAATATTACCATATTTTGCACCAGACTATACAGTTACTATTAATGCAATACCAGAATTAAATATTAAAAGGGACGT

GCCTATTGTTTTAGATAATGTAAACTATGAAGATACTTATGATGGTGAATTTAATAAGCGTAGAGCTGTTATATATACTT

TAGAGTTTACTGCTAAAACTTACTTATATGGACCTATGGCACAAAGTAAAGTTATTAGAAAATCACAAGCAGATATAGGA

ACATCTACGGATGCTCCTTTATCAAGAGAAGAAAGAATTATAGTAATACCAAATCCTGAAAGTGCTAATGCAGATGATGA

TTTTGGATTTACAACAAAGGTTAGTTTCTTTGATGATACAAAGAGATATAATCCAGTTACAGGAGAAGATGAATAATGGC

TAAATTGGAAGATAGTGTAAATGAGATATTGGGATTAGAAGGAAAAAATAAAGTTATACCAGAGAACCTTGAACCACAAA

AAGGTTTTAAAGTTCCTGTTCCTAGAAAGAATGGAGAAGTTCCTTTAAAAGTTGAAAAGGATATTAATACTGATTATGAT

TACAGTAGAGAAAGTTATTATAGTATAATAGAAAAAGGACAAGAAGCAATACAAGGCATATTAGATATTGCAAAAGAAGG

ACAACACCCTAGAGCATATGAAGTTGTTGGGCAATTGATAGGACAAGTTGGTACTACAGTTGATAAACTACAAGATTTAC

AAAAGAAATTTAAAGACTTAAAAGAATTACCTGGTAGAACAAACGCAAATATAAAAAATGCATTGTTTGTTGGTTCAACA

GCAGAATTACAAAAGATGTTGAATAAGCAAAGTATGGAAACTAAAAAAGAAAAGAGAATTGAAAATGAAACTATTGACGG

CAAATCAAAAGATAGCGAATAAAATTCCTATCGTACTAAAAGACTTAATTTATATTAAGTCAATGACACCATTAAAAGAA

TTATTTGATGGAGAATCACTAAATTATCCAATAGAAGTATAAGAACACATTGTATCCGAAGTACCTAGATATGGTCCTAT

GGGCATACCCTATATAGAAAAAGAATATAGTGTATGGAAAGGAAGTCAAAGAGTACAGGCCGCTAAACAATTAGGATATA

CACATATAGAAGGAGTGATAGTCAAGTGAAACATTTAGAAGAATTTACAAAAATAATAAATGAATATAAAGAAGATGGAA

GATACCGAGTCTTTAATGATATAGTTAGGACTAGAGGAAACTTTCCTCACGCTATTTGGTATTCAAAATATTCAATTAAA

AAAATAGTCAATTGGTGTTCTAACGATTATTTAGGTATGGGACAACACTCTTATGTTATAGACTCAATGAAAACAGCATT

AGAATCTAGTGGTGCAGGCGCTGGAGGAACAAGAAACATATCTGGTTCTACTCACTACCATAATGCTTTAGAAACCGAAT

TAGCAGATTTTCATAAAAAAGAAAAGGCATTAATATTTACTTCAGCATATAATGCTAATCAAACAACTTTAGAAACTTTA

GGAAAAATTATACCTGACTTATTGTATATATCAGACTCATTAAATCACTCTTCTCTTATACAAGGCATTAGGCATAGTAG

ATGTAAGAAAGAAATATTTAAACATAATGATGTAGAAGATTTAGAAAGAATTTTAAAATCATACGAAGGTCCAAAATGTG

TAGTATTTGAAAGTGTATATTCTATGGACGGAGATATTGGACCAGTAAAAGAAATAGTAGAACTAGCTAAAAAATATAAT

GCAATAACATTTTTAGATGAAGTACACGCTGTTGGTTTATATGGTGCAACAGGTGGTGGTATTACTGAAAGAGATAATAT

AGAAGTAGATATTATTAATGGAACATTAGCAAAAGCATTTGGAGTACAAGGTGGATACATTGCAGGAAAAAAAGATTTTA

TTGACGCCATAAGAAGTTTGGCAAGTGCTTTTATATTTACAACTAGTTTAAGTCCAGTAATTTGTGCTGGTGCTTTAACA

AGTATTAAATATGTTAGAGACCATCCTGAATTAAGAGAACAAATACACGAAAGAGCAAATAAAACTAAATTAGAACTTGC

TAGACAAGGTATAGAAGTTATGAAAAATGATAGTCATATTGTTCCTGTAATTATTGGGGACCCTAAAAGAGCTAAAGCAA

TATCAGATGAACTTTTATATAAAGAAGGTATCTATGTACAACCTATTAATTGGCCGACTGTTCCTGTAGGTACTGAAAGA

TTAAGATTTACTCCTACACCATTTCATACAGACGCATTAATCTTTGATATGGTAGTAAAACTAAAAGCGGCTATGAAAAA

ATGTGGAGGTAAAAGTGCAATACAAAGTAATGCCAAAACATAGAGAATATATTATACCAACAACTTCTTGTATAGGAGGT

TGGTATATTCCTTCTGGTATTTGTGATGGACTTATAAATTTATTTAAAGAGAATAAACAAGCACAAAAACCAGGTGTTGT

AGGTTTCACTTCAAAAATTAATAAAGAAGTAAAAGATTCTATAGATATTGGATTAGATCCAAATTGGGAAGAACCAAGGT

TTATGAAATATAAAAATGCGTTGAAAGAATGTGTTGGTCTATACGAAGAGAAATATCCTGAAGTTAAAGAGTTTGAAAGA

TATGGAATGGTTGAAGGAGGAAATTTACAATACTATCCACCAGGTGGAGGTTATTTTACTAAGCATTGTGAAAGAAACTC

TAGGCACGAAAACCGTTGTCTTGTTTGGATGACTTATTTAAATGATGTTCCTAACGGTGGTACACATTTTAAATATCAGA

ATACAACAACTCCTGCTGAAAAAGGGTTGACTTTGATTTGGCCAACTGACTTTACGCATACACATAGCGGACAAATTTCC

AAGACCCACGAAAAATATATCATAACTGGTTGGTTTGGGTATCAATTATAAATAGTAGTATGCCAGTAACAGACGCATAT

TTAGGAAATCCTAATTTAAAAAAAGTAAATATACCAGTTGAATTTACTGAAGAACAAATTGTAGAATTTCAGAAATGTAA

AACAGATCCAATATATTTTATGGAGAAATGGATGAAAATCGTTTCTCTTGATGAAGGACTTATATCTTTTAAACTATATG

ATTTCCAAAAGAAGATTGTAACTACAATAGATAAAGAAAGATTTACTATTTGCAAATTGCCTAGACAATCAGGTAAATCA

ACTACAACAATTGCATATCTTTTACACTATGCAATATTTAATCCAAATTCAAACATAGCAATTCTTGCTAATAAATCTTC

TACTGCTAGAGATATATTAGGAAGATTACAATTGGCATATGAAAATTTGCCTAAATGGTTGCAACAAGGAGTTATTAATT

GGAACAAAGGTAATATAGAATTAGAAAATAAATCTACTATTATTGCTGCTGCTACATCTTCAAGTGCAATACGAGGAGGA

ACATATAATATAATATTTCTTGATGAGTTTGCTTTCGTACCTGCTAATATAGCAGAAATGTTTTTTAGTTCAGTTTATCC

TACTATTACATCTGGTAAAACTTCAAAAGTTATTATCGTATCAACACCTCACGGAATGAATCAATTTTATAAATTATGGA

CAGACGCTGAAAATGGAAGAAATGATTATAAACCTATTGAAGTACATTGGTCAGAAGTTCCAGGTAGAGATGAAAAATGG

AAAGAAACAACTATACGTAATACATCAGCAGCACAATTTCAACAAGAATTTGAGTGTGAATTTTTAGGATCAGTAGATAC

ATTAATTTCACCAGTTAAGATTAAACAAACACCTTATATGACACCATTAACTTCAAGTGGTGGTTTAGATGTATTTGAAA

AGGTTGTAAATGGTAGAAATTATGTTTGTTGTGTTGATGTAGCGAGAGGTGTAGATAGAGATTATTCAGCATTTTTAATG

TTTGATGTTACTCAAATGCCTTATAGAGTTGTTGCCAAATATAGAAGTAATGAAGTTAAACCAATTCTATTTCCACACTT

AATACAAAAAGCGTGTAAGGGTTATAACACGGCAGATATTCTTTGTGAAACAAATGATATAGGTCAACAAATAGGTGAAT

CATTAAACTATGAATTAGAATATCCTAATCTATTAATGACTACTCAAAGAGGTAGAGCAGGTCAGATATTGGGTGCAGGA

TATAGTGGAAGAGGTTCTGGTTTTGGTGTTCGTATGACAAAACAGATTAAAAAAGTTGGTTGTTCTAACATTAAGACATT

GATTGAAGGAGATAAAGTTGTTATTAATGACTTCAATATCATAGAAGAAATGTCAACTTTTGCTCGTAAAGGAAATTCTT

GGCAAGCGGAAGAAGGATGTAATGATGATTTAATGACTTGTCTTGTATTATTTGGTTGGTTGTCTAATCAACCTTACTTT

AAAGAAATGACTAATACAAATGCTAGACAACAATTATATGAAGAACAAGAAAAATTAATAGAGCAAGATATGGCTCCTTT

TGGTTTTGTAGATGATGGTATACCTGATTGGGAAAAAACAGAAGTAGATGAATATGGAACAGTCTGGTATCCAGTTGTCA

GAAAAGGCCTCTAAATTAAGTATTATATAAATATCCATAGTTATGAAATTTGACTATGGTCGTATGAAAACATACGGAAT

ATGCGAAAAGATACAAACTAATTAGTTAATTATAAGGAGAAAACCTAATGGCATTTCAAGTATCACCAGGTGTTCTCGTA

CAGGAAAAAGACTTAACAAGAATTATTCCTGCCGTTTCAACGTCCTCTGGAGCTTTTGCTGGAACTTTCAGTAAGGGTCC

TCTTGATGAAGTTGTAAGTGTCGGTAGTGAATCTGATTTATTGTTAACGTTTGGAAAACCAGATAGCTCAAATTTTGAGA

GTTATTTTAGTGCTTCAAACTTTTTACAATATTCAAATAATTTGAAAGTAGTTCGTGTACAGAACTCATCTGTTTCAAAC

GCAACTGAAAGTGGTAGTGCGTTTGTTATAAAGAATACTACTGATTACCAAAACAATTATGCTGACGGTTCTGCTTCTGT

AGGAATGTGGGCTAGTAGAACAGCGGGTGCGTGGGGAAATAATTTATGTATTTCTCAATGTCCTTCTGCTACTGCTTATG

AAGAAACTGCTAAAACAACTGTTGCTGACGCTTCAACAAATGTCGGAGATACAGTAGTTACAGTTACTTCCTCTACAGGA

ATTAGTGCTGGAGATATAGTTAATTTTGGTGATGAATATGAATATAGAGTTATTAGTGTTGCAACTAACGACTTAAACAT

TGTGCGAAAAGAAGAACCAACATATATTGGTACTTCTGACTCTTCTGGATTACAAAAGACTATTACAAATGGTGCTAATG

TAAGACGAAGATGGAGATATTATGACCTATTTAATAAAGCACCAGGAACATCTACTTACGCTTTAACAAGAGGCGGAAGT

GCTGATGAACTACATATAATTGTAGTTGACGAAGACGGTGGAATTAATGGAACTAAAGGGGAAGTTTTAGAAAAATTTGA

AGCAGTATCAAAAGCTTCAGACGCTAAGAGTCCTCAAGGTGACACTAATTACTATTCAGACGTACTTTACAATTCAAGTA

ATTACGTTTTCTGGATGGACCACAACGCTTCTGGATCCAATTGGGGCACGGCGGCAGCAAGTACTGCATTTACAGACGTA

ACTTCTGTAAGCAAAGTATCATTATCAAATGGTTCTGACGGTACAACTGCTACAACTGCTCAAGTTAAATCTGCTTATGA

AAAATACCAAGACGCTGAAACAACAGACGTTGGATTAATCATTGCAGGTGCTGGTGACTCAACACATATAGACAATTTAA

TTACTATTGCTGAAAACAGAAAAGACTGTGTAGTTTTTGCAAGTCCTGAAAGAAGTGATGTAGTTAATGTAACTAACTCA

GCAACACAAAAAGATAATGTTGTTAATTTCTTTAATGGAATTTCTTCATCTTCTTATGTGTTTTTTGATAGCGGTTACAA

ATATATGTACGATAGATATAATGACATTTATAGATATGTACCTTTAAATAGCGATATGGCAGGATTATCAGCAAGAACTG

ATATGCTTGCAGACGCTTGGTACTCACCTGCAGGATTAAACCGAGGTGTAGTAAGAGGTGCTGTTAAACTGGCATTTAAT

CCAACTAAATCACAAAGAGATGAATTATACAGAGCAAGAGTAAATCCTGTGACTACGTTCCCAGGACAAGGAACTGTATT

ATTCGGTGATAAAACTGGACTAACAAATCCTAGTGCATTTGACAGAGTCAATGTACGAAGATTGTTTATCGTTTTAGAAA

AGGCAATATCAACTGCTTCTAAAGTTCAACTTTTTGAATTCAATGATGAATTCACTAGAGCTGGATTTAGAAATATGGTA

GAACCATTTTTAAGAGAAGTACAAGGACGAAGAGGGATTACAGACTACCTAGTAGTTTGTGATGAAACTAACAACACAGG

CGAAGTAATTGATAGAAACGAATTTGTTGCTGAGATATTTGTTAAACCAGCAAGAAGTATCAATTTTATCTCACTTCAAT

TTGTGGCAACAAGAACAGGCGTTTCCTTTGAAGAGGTCGCAGGCTAATAGAGAGAATAACGGAGAAATAAAATGGCAAAC

ATAAATGATTTCAAAGCTAAACTTTCGGGCGGCGGCGCAAGAAGTAACCAGTATAAAGTGGTTATGCCTTTCCCAGGCTA

CGCTCAAGTTGGTGGAGAAATAGAAGACCTAGCATTTTTATGTCAAGGTGCTGAATTACCAGGAATGGAAATTGCAAGCA

TTGATGTTCCTTTTAGAGGAAGAGCTATAAAAATTGCTGGAGATAGAACGATTGGTAATTGGACTATCAAAGTAATAAAT

GATACTAATTTCAAATTGCGTAATGCATTTGAAAGATGGATGAACGGTATAAACAATATGACTGATAACGAAGGATTAAC

AAATCCAGTTGACTACCAAGTTGACGCTTTCGTTGACCAATTAGATAGAAACGGTAATCAAATAAAGTCTTACACTTTAA

GAGGTGTATTTCCTACGAGTATCAACGCTATCGCATTGGATTATACGGCTAAAGCTGAATTATCAGAAACAAGTGTTACA

TTGGCGTTCCAATACTTTGAAAGTAACACAACTACTTAAAAACTACTTATAAATAGTAGTGTATTTTTAAGGAGAATAAA

TTATGGCTGAACTATTTGGATTTTCTATAACAAGGGTTAAGAAACCTCAAGATCCAAAACAAGCATTTACACAACCACAA

GCGGATGATGGAACACAAACCATCGCCGCTGGTGGGTATTACGGTCAATACTTGGATATGGAAGGTCAGACAAAGACCGA

GCAAGACCTTATCAGACGTTATAGAGAAATCGCTTTACATCCCGAGTGCGATATGGCAATTGAGGATATAATAAATGAAT

CAATTGTTGCAAACGAAGTCAAAGACGCAATAAGATTAAACCTAGAATATTTACCATTCGGTAAAGATGTCAGAAGAAAA

ATAGAAGACGAGTTTAAAGAAGTTTTAAGATTGATGAACTTCCATACTAGAGGTCACGATATCTTTAGAAGATGGTACGT

GGACGGTAGATTATATTATCATAAAGTAATTGATAGAGAATCTACAAGAAAGGGTATTACAGAATTAAGATATATAGACC

CTAGAAAAATTAAAAAGATTAGAGAAGTAAGAAAGAGAAGACCAGATGGACCTACTCCATATGGTTTAAATGTTATTGAT

GAAGTTAAAGAATACTTTTTGTTTAATGAAAAAGGTGTTACAAATACTACATCTGGTGGAATTAAAATTGCTGTTGACGC

AATAGCATTTTGTCCAAGTGGACTGATAGACCAAAACAAAAATATGGTCTTATCATATTTACATAAAGCAATTAAACCTG

TTAATCAATTACGTATGATTGAGGACGCAAGTGTTATATACAGAATTGCAAGAGCACCAGAAAGACGTATATTTAAAATT

GATGTTGGTAATTTACCTAAAGTAAAAGCAGAACAATACTTACGTGATGTTATGGCAAGATATAGAAACAAACTTGTCTA

TGACGCAAGTACAGGTGAGATACGTGATGACAGAAATTATATGTCAATGCTTGAAGACTTTTGGTTACCAAGTAGAGAAG

GTGGAAGAGGAACTGATATTACTACTTTACAAGGTGGACAAAATCTTGGTGAAATGGGAGATATAGAATACTTTAGAAGT

AAATTATATCGTTCTTTAAATGTTCCTGCTAGTAGATTAGAAGCGTCAACTGGATTTAATCTAGGACGTTCAACTGAAAT

TACTAGAGATGAACTTAAATTTACGAAATTTGTACAAAGATTAAGAAAGAAATTTACTGAAATATTTAACGATATATTAA

GAACTCAATTAGTTTTAAAAGCCGTTATTACGGATGAAGATTGGTTAGTTATAAGGGATGTAATCCAATATGACTTTTTG

CAAGATGGACATTTTGCTGAACTAAAAGATTCTGAAATGTTATTAGAAAGATTAAGACTTGCCGATTCAGTAAGAGATTA

TGTAGGTAAGTATTATTCAGTAGAGTATGTTCGTAAGAAAATTTTACGACAAAACGATAGGGATATTGAAGATATTAACA

GTCAAATTAAAAGAGAAGTTAAAGATGGTATACTTGCAGACCCTATGCAACAATATACAGCAAACAAAGATAGTATAGAA

GGAGATATGTAATGGCAGACCCAAGCGTTCCAAGTAAGACAGCGGAGTTTATTGACAAATTACAAGCGGGTAAAAACGCA

GACGCAGGAGAAGCATTTAAGGATGCTTTAAGAGATAAAGTAGCAAGTGCTCTTGATAGACAAAGAGTAGATGTTGCTGG

CAAAATTTTTAAAGGTATAGAACCTGAAAAATTTAGTGACCCTAAACCTGCGGTAACGTCAGCAAGTCCGAGAACTGATA

AGATTATGGATACAGATGGAAAAGAAATAGCTTTTGAACCGACTAAAGAACCGAGTCCAGAAGCAAGTAAACCTGAAGCG

CCAACTATGGCACCAGGACACGAAACACCACCAGACGCAGGTGTAGGAACACCAGCGCCAGACGCAGGTGTATAGAAATG

GATACGAGTTTACTTTTTACAAGTAAGATAGTTGAAGATAGTAAGTATCTTGACTCTAAAAGTTATGGAGATTTATCTCC

TAAACTAAAGTTAGCAGTACAAGATACTTTCAATCTAATTGAAAGAACGTCTGGAGATATTATAAGTAAGTTTGAAAATT

CAGTAGAGAGAGTTGCTGAAGCAAGAAAAATAAATAAAGAAGAACTATATCAATATTTTGACAAAGAAGTAAACGAACAA

TTAGGAGAGTAATATGGCGTGGGTAGATGTACCAGGATCAAATAGTATTTGGCAATATGAAAATAGTGCCACAGCATCCA

ATACGTATGCAGACGCACCTGGAACTTATTCAGGTGGCATAAGAACTTATACAACTCCTGGAACAGGACAAGTAAATAAG

ATTTATGCTAGATGTAGAAAAAAAGGAACAACAGTAGAACGTGGCGAATTATCAAAAGATTTTTTTGATGCTACACACGT

AGGATTCTAATATGGCAGATACAGTTACAACACAAACAATAGCAGATACATCTGGAGTTAAGTACGTAATTAAGATGACT

AACTTATCAGATGGTTCTGGTGAAAATAACGTACTAAAAATAGACGCTTCAGAAACAACTTTTATGACCGAAGATGGTGA

AAGACGTATAGCAAGAGTGTATTATTCTATCAATACGTCTGATAATAAATCAGGAGTAGAATTAATATGGGGCGGTGTTG

CAAATGCTACTGCTTTATTTTTATCAGGACAAGGAACAATAGATTTAAGAACTGATGGAAACTCATTTCCAAACAATGCT

ACAACACCTACAGGTGATGTATTGTTAAGTACAAAGAACTTTGCTAAGGGCGATAACTACTCAATACTTGTTGAATTTAG

ATAAAAAATCTTATAA

>lcl|3300006310_____Ga0068471_1051077|485885_ __ALOHA_-_HOT229_3_0500m Visibility public - Ed de Long 2015

CAGTAAGAGAGAGACCATAGAAGGCAGTAAGACATCTAACACCACTATGTCAGTAATACATAGAGGCGCCACTATTGATT

TGAACTAAACATATATATATCATACTCAAAAAATTCTAAATAAAAGTTTTAAAAAACAGGCCAGAGAAAACGCATTTGTT

TAATTAATTAAAAAACTTAAACTATAAATGCAATAACAGACACAAACAATATACACACTTCCTTTTCCAGAAAAAAACCG

GCCAGCATAAATATCTCTATAATGTCTATTACAAAAGAATCATACGCCGACTTAAAAGAGTATTGGGACTTTCAACGTAA

GATAGAATACAATAAAGAGAAACTTCGGTTGATGTCCAAAGAAATGCACGGTAAAGTGTACAATCAAATGGGTATGTTAA

CCGAGCAAGAACTCTTTGATAGTATCTGGACTAAACTTCCGCAAGAGGCATACGAAACACCTGCACCGACTTGGATACCA

GAAAATAAGGATTACAGATTTGACTGGGAAGGCGAACCCAAAAGCCACGTGAAGGCTATCCCTTACAATAAGCCAGGACG

TAAAGTTGTTTTACGTGCAAGAGAAAAATTAGATGAGGTTTATCCTTGGGATGACTAGTGTATATGTAATGGTGATTATA

TTCATAGTCTTTGCAATCATTGTTGGAAACTTATAAAGCATATATAGACGTGTAGTGTCTGCAAGAATGCTTTGAGTGCT

TAAAGACCACGACTCGTCAAAAATCTAATATCTCTTTCCTTTTACATAACTCTTATTCCACAAATAGATATAGTGACCTC

TACGTGTATAAGTTATAGTGAGTGGTTTCCATCTATCGTGTTGTCTTAATGTTTTAATATAGGCAATGCAATACTCTGGT

TCCCAATCTACAATTCTTACACGAAATGGTTGACGTGAGTATAATGCCTTTTGACAATAGACGATAAAGTCTACGTCAGC

AAAGGTGTGACTATGATGGCTTGATTCTATGATGATTTTATTTTTAGACAGGACCTTCATCTAAAGTTATATTTATAAGA

GTTTATCAGGTTTGGCTGGTAATGTCAAGTGCCAAATTTTTGCGAATCTGAATCAATCGTAGAACCTTTAAGCGTGGGCG

CAGGTCTTATAAATAGTATTATAGGAGATTATGTAATGAACAGATTTTTAGATGATATCGCAAACAACACACCCAACTCT

AGTATGTTTGACCAGGTCAAGCAAAAATCAAAAAAACTACGACACCAAGTTATCAACTTAAATAACTATGAACGTTATTG

GGATAACTCAACACCCACAGGACACCAAATAAGAATAGTTAACAAAGATGATTCTACTTTAACACTCAATCTGAATTGGC

CCAAAGACTATAACCCACGACTACACGATATAGACGAAAGGACATTATATGGCGGAATTAAAAGCAAATCACAAAGACTT

AAAAAGAAAAACAAGACTACAAGAAGAGGTACGCAATAACGATAGGTCATCTGATTCTTGGAGAGATTTAAAAGACTTGA

AGAAACTGAAACTCGCATTGAAAGATAAACTACAGTATGAAAAACGCAACACAACACTTTCTAAAGTACAGACCTAATGC

ACGGTATATAGAAAGAGTTGAACATAGACCGAGTCCTAGTTATGAACCACGTGAGTGTATGAATATGTCGGTGGTAGAAG

CAAAAAACTTTGGTACGAAGTTTATGACAGGTTGGTTGATTGATGATTATAATAAAAGTATAGACGCAACACCAATCATA

CATCATTGTTGGAATATAGATACAAATGGAATGCATTATGATACAGCACCGACCATACAGAATAAATATGATTATGTTAT

GGATCCAGATGTTTATAAATTTTATAAACACGAAGAGGTAGAGTACACGACACCAGTATTTTATTTAACGGATACACAAC

TAAAGATAATAATACGTGATGGTAAAACAGCAACCATTACAGCAGAAGAACATAAACGATTTATCAATGCACACACGACC

GCTGATGATAAAGAAAAAGAAATCATTTTAGAGATGATAAATAAAATAAGGGAATAATAACTATGTATGATGAAATGAAT

GGACTACAAGTACTATGGTACTTATTAACAAATTGGGAAGAAGGAAAGGGTCTTTGGTTGATAATTGGCTTTGCAATGAT

TGTCTTATGTTTTTCAATATGGGCAGATAAACATTTTGATAATGACGGATACAAACCTAAACCAAATGAATATGATTATT

GGATATGATAGAACTAGATAACAAAAACACAAAGATAGAACAAGAACAAAAAGGTGGCAATTTAAATTTTGGTCCTTATG

TGGCTATGTATTTTGCTCACGAAGAATTATTAGAAGGACTTGAGGACCGAGGTAATAAAAGTCGTGAAGGTTCGGGCAAT

AGTAACTTAGCAGGTATAATGGAAGACCAACGAGGTTATACAAAAGAAGATAAAGAATGGTTTGTAAAAGAGTTTCAACG

ATACATAGATGATTATGTACAAAGTTCAGCGGAGTATATTGGAAAACCTTTTACAGAAGAACAGTTTTCAACTAAATTCA

CACTCATAGATTTATGGATTAATTATATGAAAGAGAACGAAGATAATCCTGAACATACACACGGTGGTATGTTATCTTGG

GTTATATTTTTAAAGACACCAGACTTAACAGAAGAAAGAAAAAAGTATAAAGGCAAGAGTTTTGGTCCTGGTGGAATAAC

GTTTCATTATGGTGAACACTCTAATCCAAAATGGACAGAACACTCCTATGGTTATGAACCCCAAAATGGTGGACTGTGGA

TATTTCCAGCACAATTAAGACATCAAGTAATTCCTTTTCATACACCAGGAACAAGAGTAAGTGTATCAGGTAATTTATTC

TTTAATCATCCGAAAGATACATCAAAAGTGCTTCAAGACCCATTAGAAAGAAAATTGGAACAGTTTGCTCAGAAAGTAGC

AGCAGAAACAGACTAGATAATGAAACAAGAAGCGTTGCAATGGTTTATTGAATTGCAAAAATCAATTTGTCAGACTATTG

AAGAGTTAGAAACTAAAGCAAAATTTAAAGATAACAAATGGAAATTTGGTAACTTTAAAACAATTAAAGGTGACGTGATT

GAAAAGGGAGGTGTCACCTTTAGTAATGTTGTAGGTAAGTTTCCAAAAGACTTCGCAAAAGAAATCCCTGGTACAAAACA

TAGTAGAAACTTCTGGTCAACAGGAGTATCAGTAGTATTACATCCAAAGAATCCTAAAGTACCTGCAATACATTTTAATA

CAAGATATATTGAAACAGAAAAAAGTTGGTTTGGTGGTGGTACAGATATGACACCGTGTTTGAAAGATGATGTAGAAAGA

AATTTATGGCATTACAGATTAAAACAATTATGTGATAGACATAATAAACTTTATTACAAAACATATAAGAAATGGTGTGA

TGAGTATTTTTATTTACCCCATAGAGAAGAAACAAGAGGTGTAGGTGGTATCTTCTTTGATTACAAATATGGTAAGAAAC

ATTTTGAATTTGTTAAAGATGTTGGTAGAACCTTTTGTCAATTACTTAAAGAGATTATAACACCAAAGATGAATTTAAAA

TATACGAAGCAAGATAAGTATACACAACTATTAAAACGTGGACGATACGTTGAATTTAATTTAATGTATGATAGAGGAAC

AAAGTTTGGATTGAGAAGTGGAGGAAATCCTGAAGCAATATTAATGTCTATGCCACCAGGTGCGATATGGGAAGAGTAAT

ATCTTGTTCAGCAAGAAAACAATGGGACGTACATATCAAAGCATTTTTAGATAGTCATATACGCCACGCAGAAGAACATT

CTAATTTTTCATTTGGCTATGATGATATAGATTCTGTTTTTGGACAAACGGAAGAACATTGTCCTTTATACGGTGGACGT

CCTGCAGAAACTCCAGAAATGTTTGAGAAAGATATTGAATGGATTTATGATAAGGGTATTGGTCTTAAACTTACTTTACA

GAATAAATTTATAACAGATGATAAGTATAAAGAAAGTAAACCGTTTTTAAAAGAATATCATAGAAAAGGAAATGCAGTTA

TTACAGCAACTGATAAGTTGGCTGAATATATTAGAAATGATTTTCCTGATTATAAAATAGAAGCAAGTTGCATACAAGAT

ATTACAGACAATGAACATTATGAAAAAGTTGTTGCAAAGAATTTATACGATACAATTGTTTTACCTATTCATAGTAATGA

TGATTTAAAGTTTATAGAAAGTATTAAGAGAAAAGATTTATTAAGATTGTTTATGAACATAGAGTGTTCTTATAATTGTC

CTAGTAAAGTTTGTTATGGTACAACTTCTAAAATTAATAGAGAAGAAAGAAAAGGAATGATATGTAGTTTAATTCATTTA

GGTATGGAACGTACTTTTTATAATGATGATATAACTTGGAGTGAATTTTATTTTGATTTACCTATGTATGAGAAAATGGG

TATATCTAAATTTAAATTAGTAGCACCTCACGAAGAACAACAACGAACAGCATTGATGTATAAAAGAAACCATCAAATGT

TAGCAAAGTCAGCAAAATGAGATTAATACAACCAATCTTTGCTAGTAACTCTACAAGAGAAACTGGACTAGGAGAAGATA

ATAAAATCTTTAGTGCAAAAGATGTCAAAGAACGTGTTAAGAAAGATATTGATTTAGGAGTAAAAGAATTTCTTTTATTT

TACATACCTGAATTTAAGTTATTTAACTTTGACAAAGTGTGTGAAACTGCTCACTCTTTACAACGTCTATCACATATACC

TATAAAATTAAATGTTGATGTATGTTTATGTTCTTATACCCACGATGGACATTGTTGTGTAACAGGAGACCAAGAAAAAA

CAGATGACCTATTACTACAATCAGCTTTAGAGATTTACACAGCGTCAGGTGCTACAATAGCGCCAAGTGATTGTCAAGAC

AATACAGTTAAGAATATTAAATCAATTAATAATGGTCAGATACCTGTAATGAGTTATAGTACAAAATTTCGTTCAACATT

TTATAGAGGTTGGCGTGATGTAATGAAGATACCAAAAGGTATTCATAGACCCTATCAATTAGATGTTAGCGATAGACATA

AGGCAATTATAAGGTCTATAAAATATTCAGATGATGGCGCAGATGAATTAATGGTAAAACCTGGTATGACAAGTTTAGAC

TTAATTGAACCAATAAGAAATATTACAAAGAAACCTGTAGGTGTATATCAAACATCTGGAGAATGGTTAGGTATTGGTGC

GCCTGGTAGTTTAGAAGAAACATATCACATATTCAAAAGAGCAGGTGCTTGTTATATGATAACTTATGGTGCAAGACGAT

TATGTCGCTCACAATAGGATCAAGAGGAAGTAAATTAGCACGTATCTATGCTGAAAAAGCTAAAGCAGTATTAAACAAAG

AAATACTTATCAAAGAAATTGAAACCAAAGGTGACCAACTTAAAGATGTAAGACTTTCTGAAGCTGGAGGTAAAGGTCTC

TTTTCCAAGATGATAGAAGTTGAATTATTAGATAAAAAAATAGATATAGCCGTTCACGCATTAAAAGATATGCCAACGGA

AGAAACAGAAGGTTTATTAACAAACTGTTTTTTAGAAAGAAACGATCCAAGAGAAATTTTAATTAGTAGAGATAATAAAC

ACTTAAAAGATTTAGCTCCAAATTCAATTATTGGTACTTCTTCATTTAGAAGAGAATTCCAATTAAAGAATATAAGAAAA

GACCTTATATGTAAGTTGATAAGAGGAAACGTTGATACACGAATTAAAAAACTAAATGATGGTTTATTTGACGCTATTAT

TTTGTCTTATGCAGGAATTCAATCATTAGGTTTAGAGAACAAGATTTCTCAAACTTTTTCTACTAGTGAAATGATACCGT

GTGCAGGACAAGGTGTTATTGCCTTACAATGTAGAGATAATGATGAAGAGATAATTGAGTTATTAAAAAGTGTTAATCAT

ACAGAAACACACAATTGTGTTAAGGCAGAAAGAAACGTTTTAAAAATTATAGAAGGAGATTGTGATACAGCGGTAGGTGT

ATTTGCAAATATTGATGGTAATACGATTAATCTTGAAGCAGAACTATTTTCACCAGATGGTAAAGATAGATTCTATTCAA

AATCATCTAAAACTATTGATAAAGCAAGTGAGCTAGGAATAGAGATAGGCTATTTATTAAAATCTAGTGGTTAGTCTTGT

TCAGAATACAACGTTTGCGAGTATAATGCTAAAATAAACATAGCAATTCCTAACAATGACAAAGTACCACATAAAAACCA

ATTATCGTTCATTGGAACTCCGTTATAACCGCCGTCAATTGCGCCGACAGCACCGATTAAACAGAAAGTACCTACTATTG

ATAAAATGATAGTTAAATATTCAAGTAGTTTTTTCATAATGTTCTCCTTTTCAACTTATACGTTAAATATACACTAAAAA

TTTAGGAAAGTCAAGGGAAAAATTCAAAAAAAATGAGAAAAATCAAGGTTTTTTTAGTTGTGTGTTCGCTTTTTGTTCTA

GTTTCTTGTGGAAACGTTCATAATTGCAGATTTTCCTATGACATAGACAAATTTCCGAATCGGGAAGCAGTTTACCTGTG

TGATTTTTAGTATAAATACTATATTATGACTTATTGCAACAATTGTGGGAGAGAATCCCATTGCGGAAAGCCAAAGTATG

AAATGATGGAAGCAAGAAAATTGGAAATCTGTAAATATTGTAGATGTGATGATAAAAAATGTAGTGTGAAAAGGAATAAA

CAGAATGTCAAAAGAAAAAAAGTTTAAGTTTACAGATAATAAAGAAATAAATCAAGAAATATCTGCTACGAGTTGGAAAA

AGGCAGTTAAATCTTTTCAAAATAAAGTAAAAACGCCATTAATCTTTATTGAGTGGATAAGTAAGAAAGGTCAAGAGATG

ACCAAGTGGCAAAAACTACCTATAGGTAGAAAAGATAAGATAGGAAAGTAAATTATGAGTAATATTGATACGTTAGTAGA

ACAATTGGGTAAATTAACAGTTATTGAAGCGGGTGAATTAGCAAAAAAACTAGAGAAGACTTGGGGTTTAGATTTAAATG

CTATAATGAGTACACCTGCACCAGTTGAAGAAGTAAAAGAAGAGTCTTTATTCAAAATTACACTAACAGGTTTTGATCCT

GATAAAAAAATTAGTGTAATTAAAGCAATTAGAGCTTTTAAAGATATGGGACTACTTGAAGCAAAGAATTTTGTAGAAGG

TTGTCCTTCAATTATCGCTGAAGACCAAGCAAAAGATGAAGCAGATAAAATTAAGGCAGATATTGAGTCTGCTGGAGGTA

AAATAGAGGTAAAATGATAGAATATTTAAAAGACGCAAAAAAATGGTTAACTGAAACTAAAGTTCCAGTATACGTTTTAA

TATTAGTAGTTCTAATTTGGATATTAGCATAAAGCAATGCCAGGTATCAGTAGAAAAGGCGACCAATTAACAACAGGACA

CACTTGTGCTGGAGTAACTACGTTAGATACTCCAACACAATCTACTGTAAGAGCAAATAATATTGTTATAGCAAGAAAAA

CAGATAAAACTGTAGTACACCCAGCACCACCACCACCTTTTTGTCCAGCACACGTGATGATGGTTAATGTAGGAAGTTCT

ACAGTTAGAGTTGTAGGATTACCTGTATCACGAATTGGTGATAGCGCAGACGCAGGACAATTAATTAAAGGTTCTACTAC

TGTAAGAGCGGGTTAAACTGTATAAATATTACAGTTATGGCACAAAGCAATTCAGCATTTTTAGATGATTATACAAAACA

CGTTAAAAGTACTAGTACTAGAACATCTAGGAAATTTAAAGATATAGATTTAGACTTTGGTAGAAATCCAGTTACTAATG

ACGTTAATGTGGTTGAAGACGCAATAGCAATAAAGAGGTCTGTTAAAAATTTAATACAAACAAATTTCTATGAAAGACCT

TTCCATCCAGAATTAGGTTGTGGTGTAAGGGGATTGCTTTTTGAAAATTATTCTCCAGTATTGAATGTCTATTTAAAAAG

AAAAATAGAAGAGTGTTTAATTAATAATGAACCTAGAATTGAGTTAACTGGTATTATGATAAATGGAGATGATTTTGAAA

AAGGTGCAAGAGTTAGTGGTAATGCTGATGAGAATAGATTAGATGTAACCATATATTTTAATATTATAGGTGTACCAGAA

CCACAAGAAACAGCAATAAGTTTACAAAGGTTAAGATAAGAAGATTATGAGTGAATATAATAAATTTTTAGAAAGAACAA

TTGCAAAATCACAGCAATGTAATAGGAATTGGGATTTATCTAAACAAATTCCAGATAAAGATATTAAAACGATGGAACAG

GCGGTGACACAATGTTCATCTAAACAAAACCGTGTATTTTATAAAGTTCTATATACACAAGACCGTAATAAGATTGAAGC

AATACATAATGCTACAGATGGTTTTACATATCGTTTGCAAAAGGACAAAGATGGTAATTATTTAACAATTACTAATCCTC

AAGTATTAGCAAATACATTATTTGTTTTTGCAAAAGATAGAGATGATAATATGGAAGCCAGAACGGATAAAGAGAATGAA

CTTGGCATAGAAGAAGAAAGAAATTCTGAAGACGGAAAGACGGATGAAAATCGTGCAATTGGAATTGCTGCTGGATATTT

AACTCTTACTGCTAATCTTTTAGGATATGAATCAGGTTGTTGCCAGTGTTTTGATGGAGATAAAGTTAAAAGTATATTAG

GTGAAGATGTATTTTTGTTAATGGGTGTAGGTTATGGTGATAAAACAAGACCACGAAAAGAACATCATATGGATCCAAGC

ATTACATTTCCTTCTTTCAATAAAAAAATAAAAGTAGAACGAGTATAATAAGATGTCACAACATAAATTACAAATATCAG

AATTAGATTTTGATTTAATTAAAGCAAATTTAAAAACATTTTTACAAAGTCAAACTCAATTTCAAGACTATGATTTTGAA

GGGTCTAGTTTATCTATTCTATTAGATGTACTATCATATAACACTCACTACTTGTCATACATTGCTAATATGTCAACTAA

TGAAATGTATTTGGATAGTGCTGATATTAGAAAAAATATTGTTTCATTAGCAAAGATGTTAGGATATACTCCTACATCTC

CTAGAGCACCAAGAGCGTCTATTGATGTTGTTGTTAATGGCGCAACAGGTTCGTCTGTTACAATGCAGAAGGGAACAGTT

TTCACAACTACAGTTGATACAGTTGATTATCAATACGTGACTAATGAAGATATAACAATTTCACCAGTAAATGGAGTTTA

TAAATTTGAAAATGTGCCTCTTTATGAAGGAACATTGGTTACATTTAAATATACATATGACACAAATGATACTGACCAGA

AATTTGTTATACCTAGTGTTTTAGCAGATACTTCAACTTTAAAAGTTATTGTTCAAACTAGTGGAACAGATACAGCACAA

AAGGTTTATACAGTAGCGGGTGGTTATAATGATGTATCAAGTATTTCAAAAGTATATTTTATACAAGAAGGTGTAAGTAA

TAAGTATGAAATTTATTTTGGTGATGGTGTAACAGGTAGAAAATTAGAAGATGGTAATATTGTAATAATGGAATATATTG

TAACTAATACAATAAATTCAAACGGTGCTTCAAAATTTAATTTATCAGGAAATGTTGGTGGATTTACAAACGTAACTATA

ACAACTGAATCAAATTCTTCAGGTGGTGCAATTGGAGAATCAAATGAGTCAATAAAATTTAATGCACCTTTACAATATGG

TGCTCAAGATAGAGCAGTTACAGCAACTGATTATGAAACTATAGTTAAATCAATTTATCCAAATGCATTATCAGTAAGTG

CTTGGGGTGGAGAAGATGATGAAACTCCAACTTACGGTGTTGTAAATATTTCAATTAAAGCAAAATCAGGAACAGTATTA

TCAGATACATCAAAAGCAGATATAGTAACTCAATTAAAACCTTATAACGTTGCTTCAGTAAGACCAATTATAAAAGATCC

AGAAGCAACTTCTGTATTAATTACTTCAAATGTTAAGTATGACGCAAAGGCAACAGCAAAAACTGCTGATACTATAAAGG

CAGATGTTATTGATAAGTTACTAGCTTATAATACTTCTACTTTACAAAAGTTTGATTCAGTATTTAGATTTTCAAAAGTT

ACAGGTTTGATTGATAATACAGATGATAGTATTTTATCAAACATCACAACTGTTAAAATAAGAAAATCTTTCCAACCTAT

ACTTTTAACATCTTCAAAATATAGTATCTATTTTAGAAATGCATTATATAATCCACACTCTGGACATATGGCAAGTACAG

GTGGAATATTAAGTTCATCTGGATTTAAAATTGATGGTAATGATAACGAATGCTTTTTTGATGATGATGGCGCAGGTAAT

GTAAGATTATATTATTTGTCAAGTGGAGTAAAATCTTATTTAAATTCAACACAAGGTACTATTGATTATGGCACAGGTGC

AATAATAATTAATTCATTAAACATTGCTAGTATATCAAATATTAGAGGCACAACTTCAACAGTAGTTGAATTAACAGTAA

CACCAAGTTCTAATGATGTTGTTCCAGTTAGAGACCAAATTGTTGAAATGGATATTGCAAATTCAACTATAACGGTTACT

GCTGATAGTTTTGTAGGAGGAAGTGCTGAGGCAGGTGTGGGATACACAACTACTTCCAGTTATTAATGACAAATGGCAAA

ATTTAATGATAAGATTTCTACAATACTTTCTGGTCAACTACCTGAATTCGTAGTTACTGAACATCCAAAGTTTGCTGAAT

TTCTTAAAGTCTATTACCAATTACTAGAGTCCGCTGAGTTATCAGTAACTTCTGTTAAATCTACAGAAGGTATCTTATTA

GAAACAGAAACAGACCAAGCAAATAATTTAGTTTTAAATGCAAGTGCTTTAGGTAGTGCAAGAACATCACTTGACGCAGG

TGATAAACTTATTTTTGAAATTTACTCTGGTACTGAATATGGAAAATTTGAAAGAGGTGAAACAATTACAGGACAAACTT

CTGGTGCAACTGCTGTTGTATTAACAGAAGATTTAGATACTAAACGTTTATTCATAAGTGCTAATAGTAAATTTATAACA

GGTGAAATAATTGTAGGTGGTAGTTCAAATGCTTATGCAACAATAAATAATTATAAACCCAATCCAGTAAATAATATTGC

TGACCTAGTTAACTTTAGAGATCCAGATAATGTAATTAGTAATTTCTTATCAAATTTTAGAGATGAGTTTCTTGCAACAC

TACCAGATACATTAGCAAATCAAGTTGACAAAAGAAGTCTTATAAAAAATGTTAAATCACTTTATCGTTCAAAAGGAACG

AATAGAGGACACGAAATATTTTTTAGAATATTATTCAATGAAGAATCACAAACCTTTTATCCAAGAGAACAAATATTAAG

AATATCAGATGGTAAGTATGATACATTAAAAGTTTTAAGAGCTATTGCTGATATTGGCGATACAGCACAATTAGTTGGAA

GAACAATTACAGGTGCAAATAGTGGTGCCTATGCAGTTGTTGAAAATGTTACCAATTTTCAAATAGGTGCAGATACAGTT

ACAGAATTTATTTTAAATAGTGATTCTATTCAAGGCACATTTCAAATTGGAGAACAACTACAAGGTTCTGCTTCAGATGA

AGACGATTGGTATATTAAAGCAACTATAACAGGAATACCAGGAACAAAATCACTTACAAATGATGGTGCATTAAATGAAA

CTACTGATACAATTAAAGTTATTGCAGGTGGAGTTGGTGCTATATTTAATATTGATGAAGTTGGTTCTGGTGGAATAACA

GATATTGTAATTAATAATAAAGGAATAAATTATGAAGTCGGTGATAAATTAGTATTTGATAATACTGGAACAGGTGGAAG

GGATGCTGCTGGATTTGTAAGAGTTATTAATGGTGGTATTGCAGGTGAAGATTCTGACCAAATAGTTTTAGAAGATGGTA

CTATGGCAGCAGACCCATATTTTGGTAATAGTATTATGCAAGAATTAGGAACAGGCACAGGAACAATTGAAAAGATATTT

TTAATTTATAATGGAACAGGATATACTTCTTTACCTGGCGTAACTATAACATCATCAGGCGGTTCAACTGGAAGTGTAAA

GGCGTGGGGTGATGAAATTGGAAGAATTATTGCATTAAAAACAATTGAGTTAGGAAAAAAATATCAAGACGCTCCTAGTC

CTCCAGTATTAGAATTTTATAACAGTTGTGTATTAACAGGTGCTACTGGTCTATTTACAGTAGGGCAATCTTGTACAGTT

TCAGGTGGACAAGGAACAATTGTTTCATATAACACTTCTACAAATGTATTAAGAATTAAAAGTATTACAGGTGCATTTAC

AGAAGGTCAATTATTATCAGCAGATTCAGGTGGGTCAGGAACTATTGCTAAAATTGATGTTGCAACAGCAAATGTCAATG

TAGTTTCAGTTTCAGATACAGATGGTAAATTTATTAATGAAGATGGTAAACTTTCTGAAGTAACAATGAAAGTACAAGAT

AGTAGATACTATCAAGATTTTTCTTATGTATTAAAAGTTGCTAGTTCTATTGCAGTATGGCGGGACGCATTTAAAAAGAC

AATGCATACAGCAGGATTCTATTTTACAGGTCAAGTAGATATTACTTCACAACTAGATGTTAGAGGAACACTACCATTAG

TTGGTGCTGTTTCTGGTAGAACAGAAGTTGAAATACCATTAATTGCAATTCTTAATACTTTATTCTCGGTGATATTTGGT

AGAAGATTAGGAACGATAGATGATGGAACATCTTTAAGAGCAAAACCTCTTGAACCAGGAGCTATTGATTTAGACCATAA

TACAAATGAACATTTTGAAGCAAATCAAAGAGATTTAACTTTAACAAGACCTGGATTAACAATAGATTATTTGAGTAGAA

AAAGGGCAACAATAGGTGGTCAATTTGTTAAAGCTGGTTACGCATATGCTGGACCAAAATGGGGAACACTTAATAAGTAT

GCAAATACTATATTTAATACTTCAATTGGTGGTACAGGACATACGTTTGAACAATTAAATAATTTAAAAATATTTGGAAC

AAGAACTAGTTTAGATGGTCAAGGTGGAGTATTCCTAATGTCTTCTCATCCTGAAGGACAGAAAGTTAAAATGGCTCTTG

CGTTTCCTTCATTCTTAACTTATAGTAATAATGAGTTTAGTAATACAGTAACTAACTTCTCTCAAACAGGACCAACTTTT

GATGATACAACACCGTAAATGATTATAAATAGTAAAGTAATTTAAGGAAGAAATGGCAAAAAAATCAATAGATATAGGAT

CAGCAGCTAATGACGGAACAGGTAGTAACCTACGTGTTGGTGGTGGTATTATAAATGATAACTTTAATGAAATTTATACT

GCTCTAGGTGACGGTACTACTATAGACCAGAATAGATTACTTAATTTAGCAGGTGGTACTGGTATTGATACTACTTTAGT

TGGTAATACTTTAACTTTTGATATTGACGCTACAGTTCTTACAGAAACATCAACAGATACATTAACAAATAAATCAATTG

ATTTAGCAACTAATACCATTACAGGTACTACAGCACAATTTAATACTGCTTTGTCAGGTGATGATTTTGCGACACTTTCT

GGTGTAGAAGTTCTTACAAGTAAAACTTTAACTACTGCTACTCTTGGTGGTAAATTGATAAACGATTCTGGTGATATGGA

GTTAGAACCTGTTACTGCTAATTTAGTAATTAGAGGTGATGGTTCTTCCCAAGACGCAAAAATTACACTAAATTGTGATG

CTAATACACACGGACAGACTATAACAGCACAACCTCATAGTTCGGGTCAAACTAACACAATGTTATTACCAAAAGGCGGT

AATTCAACTTTAGTTTCAGAAATTGCAACACAAACTTTAACAAATAAAACTTTAGACAGTCCAGTTATTAACACACCAAC

AGGTGATGTAGTATCATTATCAGGTTTTCAAACCCTTACAAATAAAACTATTTTAACTCCTGTAATTACAGGTTCTTTAT

TCAATATTGCAGATGATACATCAACAACTTCTTCCATAGCACAAGGAGATGTTTTTAAAATATCTGGTGGTACTGGTATA

ACAACAGTTGTAAGTGGTGATACTGTTACACTAACAGCTGGAGGACTTACAAATTCAGAATTAAGTGGTACTGCTGGAAT

TTCAAATGCTAATTTAGCAAATAATTCAGTTACTATTGGTTCTACAGCAATTGAATTAGGATCAACTGCTTCAACAGTAA

ATGGTTTATCATTAATAGGTTCTGCTTATATAACAGTTAGTGGACAAAATTCAGCAATAAGATTTAATCACGTAAACTTA

GCAGCCTTTCCTAATTCAACTACTTATTCAGGTACACCTGCTTTAGATGAAACAACACTTAAACCTTATATAGCAACTAC

TTCAGGTTGGGTTGAAATGTTAACAGAAAATTCTAGTGCTGATGATATTTCAAATGTAAGTATGACAGGAATTACTGATG

GACAAGTATTGGCTTGGAATTCTTCAACTACAAAATTTGAACCAACAGCTGCCGCTTCAGCTACACCTTTCACAACAGAT

AAATCACACGTAGGTGATGGTTCAACAACAGGATTTACAATTCTTGGTAGTAGAACTGTAGATAATATTTTAGTATTTGT

AAATGGTATTTGTTTAGTACCAACAGACGATTATACATATGCTGGAACAACATTAACTTTTATAACAGCACCTGCCAATA

GTGCAGAAATAGTAATAAGATATTTAGGATCATAAAATGGGAATTAGAACAAGAAATAGAGCTAACAATGTAAATGCAGA

TGGCACACCTTTAACTTTAGGTGCAAGTGTGCAACCAGTTAAAGATGATGTAACAGCTTTGGCTTTAAGAGAAGCAACAA

ACGAATCGTCTGCTGCTTTCAATTTGCCTAATACTTTTATAGATACGTTTTCAGATGACACAAATCTAGGAACACAAACA

GATGTAGATAGAGTTAGTGGTCATATAACAACTGCTATAACAACTGTTGACGAATGGGTAAGCGATGCTAATACTTTAGC

ATTATTACATTATAATGGAGCTAATGCTGGAACAGTATTTACAGATAGTTCTTCACACAATAGAACAATAACTAGACGTA

ATCAACCTACTACAAATACAGGAGAGAAAAAATTTGGAAGTGCTTCTGCTTTCTTTGATGGTTCAGATGACTCTTTATCA

ATGCCTGATAGTGATGATTGGATTTGGGGAACAGGTGATTGGACTATGGAAACTTGGATATATATGAACGTCAATACAGG

CACACACGGATATGATATATTCAACCAAGCAAAAACAGATGCTACCGATATTGGTGGTGCTTGGCATTGGGGTATTAGTC

CTAATGCAGGAATGAAACAAAAATTTAATGTTTATCATAGAAATAGTACAACAACAGCAGATGATTTTGCTTTTGAATCT

GGTACTGCTATGTCAACAGGTCAATGGTATCATATGGCAGTAGTTAGAGATGGAGGTACAATAAGATTTTATAAAGATGG

TGTACAAGATGGTTCTGCTTCTGTACCAACTTCTTCTGGTGGTCATTTAATGACTGGTGCTTTAGGTGGTCAAGTATGGA

TAAGTAAAAGAGCTTATACTGACTCTTATGGTGTTCTTAACGGTTATTTAGATGAAATGAGAATATCAAATAATTGTAGG

TATCCTAATGGAACAACTTTTACTCCACAAGAACGTACAACATCAACAGCTACAGGAACATTAATTCAATCAGCTAATAC

AGTAGATGTAGCTAAAACAAAAGTAGCTGGAACAATGCTTTATAAAGATGGTATTGGAACAGGAGTTATCGGAACGGATT

TAAAGATATATTTCAGTTGTGATAATGGTTCAAATTGGACTGAAGCGGTAAGTTATGATACTATTACTCCAGTCTATAGT

ACTGGTGTTAAACAAGTCCGTTTAGGAGAAACAACTTGTACAAGTGGCACAGGAGTTATTTACAAAGCTGTTTGGGCGAA

CCAGACAGATACTACTAAAGAAACACAACTCCACGGAATAGGAATTAATTACTAATTAGAAAACTTGTATAAATATAGAT

AAAGGAAATAAGAATGCCAGCAATTATAACAAATAAATTCAGAATACACAACTCGGAACAATTCCAAGAAGCGTTTACTG

AAGCGTCAGGAAATACTTTCTATTTAGGTATAGGAAGACCTCAACCATTTGGGACTTCTACAAGAGGAGATGGAAGAACA

AATAATGAAGGAACAGACGCATTACCTGTGACTCCTGCAGATAACGAAAATACACAAAATTTTACATATGATGATATGCT

TGCTTGTAAAAAAGTAGCAAGTACAAATGCTGGATTTGTAATTCCTAGAAGAAATTGGACGACTGCTACTGTATATGATT

ATTACAGACACGATTATGGAGAATACATTACTGGAACAACAACAGCACAAACTTCAAATAGTGGTGCTGTTACTTTATAT

GACGCAACTTTTTATGTATTAAGTGCTGCTAGAAATGTTTACAAATGTTTAGATAATAATAGCGGTGGTACTTCAACTGT

AGAACCTACAGGAACATCAACAACAATATTAGCAACTGCTGATGGATACAAGTGGAAATATATTTACACTTTAACTGCTG

CTCAACAAGCAGAATTTTTATCAGTAGATTTTATGGCAGTTGGTACAAATGGAACAGTTAATGCTGCTGCTGTAGATGGT

GCAATCAATGTAATTAAAATTAAAACAGCGGGTTCTGCTGGTACAGATGGAACACACGCAAGTGTTCCAATACGAGGAGA

TGGATCAAGTGGAGTTTGTTCAGTAACCATTGCTTCAGGTGCAGTTACAGCGGTAACCGTAACTACTCCAGGAACAGGAT

ACACTTACGCATATATTAAACTTGCAGATATAAATGCTGCTGGTGGTGGTGCATTAATTAGTACAGAATTAGATTGTATG

ATTGAACCAAAAGGTGGACACGGATTTAATGCAGTACAAGAGTTAGGTGGATTTTTTGTTATGTTGAATACAAGTTTAGA

AGGAACAGAATCAGCTAATTCAGGTGACGTAACTGTTGCAAATGATTTTAGAAAAGTAAGTTTAATAAGAGACCCGAAAT

CAGGTGGTACTGCTGCTTCTGCTGCTACATTGAGAGCAACAAGTGCCGTTGTTGGTGCGTCCAGTAATTTAACATTTTCA

GTTGACGAAAAAATTTCACAAGCAAGCACAGGTGCAGTTGGAAAAGTTGTAGAGTGGGATCCAACAAATAAAATATTATA

TTATATTCAACCAAGACACAATGATGAGGGAGTAGATACTAACGGTAATCAGACAGCATTTAGTGGCACAAATATTATTA

CTGGTGCAGATACAAACGCAACTTTAACACCTGCTACGACAACAGGTACAGTTAATAGTCAAACATTTTCAAACGGATAT

TCTAGTTCGGAAATTGACCACGGTTCAGGTGAAATAGTTTATGTAGAAAATAGAGCGCCAATCACTAGAGCTGCTGACCA

AACCGAAAATATCAAATTGATTATAGAATTTTAGGGGAGTTAAATGCCAAGTCCAACAGATTTTAACTTATCGCCCTATT

ATGATGACTTTAATGAAAATAAAAAATTTCATAGAGTTCTTTTCAGACCAGCATTTGCTGTACAGGCGAGAGAGTTAACA

CAATCACAGACGCAATTACAAAACCAAGTAGAAAGGGTTAGTGACCATCTATTTGAAAAAGGTGCTATGGTTATACCTGG

AGAAATCGGGTACGACTTAAATTACACTTCAGTAAAACTTTCAGCAAAATCAAACTCAACTTTAGCAGATTATAATGGAT

TAGAAGTAACAGGTGCAACTTCAGGTCTTGTTGCAAAAGTTATAGGTGTATCTGTAGCTGATGGAACTGATCCAGATACA

TTATTTGTAAAATATTCAAAAACTGGAACAGATAATACATCAACTTCCTTTTCTGATACAGAAATTTTAGATTGTACAAT

TAATTCTTTGGCTGCTACAGCGACTGTTGCTTCAACACATAAAGGTTGCGCTGCTGAAGTACAAAAAGGAGTTTATTATA

TTAATGGATATCACGTTGAAGTTTTACAACAAACAGTAGTACTAGACAAATATACAAACACACCTTCATATAGAGTTGGT

TTATTAGTTACAGAATCTTTTGTAACTCCAAATGAAGATGGAAGTTTAAATGATAATGCTCAAGGAACATCAAATCAAAA

TGCTCCTGGTGCTCATAGATTTAAAATTGATTTAACACTTCAAAAGAAAACTTTAGCAGCTACAGATGATAAAAATTTCA

TAGAATTATTAAGACTATCAAAAGGTATTTTACAGAATAGAGTTAGAAATACAGAATATGGAGTATTAGAAGATACTTTT

GCTCGTAGAACATATGACGAGTCAGGAGATTATATTATTAAAGGTTTTGAATTAGATATAAGAGAACATTTACTATCAGG

AAATAATAGAGGTATTTACGCTTCAGGTGCAGGTGGTGATTCATCTAAACTTGCAATAGGTTTAGCTCCAGGAAAAGCAT

ATGTTAAAGGTTATGAAATAGAAAAATTAGGAACAACTTTTGTTGATGTAGATAAAGCAAGAGAGTTTGATACAGAACCA

AACTTTAAAACTAGATTTGATTTAGGTAATTACTTAAACGTAACTAACGTTTATGGTACACCAGATATTGGTTTTGTAAC

AGGTGATATAGAATCATATAAAAGTCTTGACTTATATAGTACGGCAACTGCTGTTAGAGGTGTAGTTAATACAGCAGCAG

GTTCAAGTATTACTCAAATAGGAAGAGCAAAGTCAAGAGGATTTGAATACTCATCTGGTACTGCTGTAAATAATATTGTT

TCAAGTGGTGGTTTAACAAGTTCTATTTTTAAACATTATCTATTTGATGTAGAAACGTATACTCACTTAAATATATTAAC

AGCTCAAGCTTTTACAACTGGTGAAACTGTAACAGGTGGCACTTCTGGTGCAACTGGTGTTGTTCAATCAATATCAACAA

CAGAAACGGCAACAATTAATAGTATAACTCAAGCAAATCCTTGCGTAGTTCAACACGCAAGTCCCCATAATTTTTTAGAT

GGACAACAAGTTACTATTGCAGGTGTTGGAAGTTCTTGGGCAATTGATTCAGTTGTAACTACTGGTGGAGTATTTACAGT

TAGAGTAGTTAATTCTACAGATTATAATTTATACAAAGAAGATGGAACAACTCCTGTTAATTGTACAACTCCAGGTACAG

GTGGAACAACAGCACACGGAATTGTAATAGCTTCAAATGTACAAGGTTCATTTACACCAGGTGAAATAATTACAGGTGGA

ACTTCAAGTAATACAGCAACTTTACAAGCAGATGTTTTAGGACGTAAAGCGGCAATAGTTTATGGTTCTTCTGATATTAA

AACAATTAGTATGACAGGTTCTCCTGGGTATGTTGCGGATGTAGTGAGAGATAATGTTAGTATGACAGGTACACTATCTG

TCGCAGGTGCAGGAACAATAATTAATGGATTTGGTACAAGATTTACAGATGAATTAAAAATTGGAGATAAAATTACATAT

ATAACAAATACTCCTCTTGCAGAAACAAATGAAGTAGCCTATGTTATTAATGATACAAGTTTTGCAGTAACTTTAGGTGC

AGGTGCTGATGTTACCAAATCATCTTTTACTAGAGGACGTGGTGCAGTAAAAGAACCAGGTAAAAATATTTCTATCTTTA

AAATGCCTTATGAAACTATTAAGACATTAAAGACAACTGTTAATTCAGGAATAACAGATACAAATTTTGCAATTAGAAGA

CATTTCACAGCAACATTATCAGGTAATGGTGACGCTTCAATTACAGCAGGAACAAATGAAACATTTAGTGGATTAAAAGA

ATCAGACTTTACTGTTTCTATTATGACAACTGGTGGTGGTAGTGCTGGTGCAGTATCAGATATATTAAGTTTAAGTGGTA

CTAACCATTTAGCACAAACAATATTTACTTTAGCAGGTTCTCCAACTGGTAAAACTTTAACATTAAATTTTGGTACTAAT

TATGCAGGACATAAAGTAAAAATATTAGCAACAGTTAATCGTTCAGTTGCAAGTTCTAAAACTAAAACATTAAATTCAGC

ACAAACAGTACAAATTGCTACACAAGCAACTGTTGAATCTGGTGTATGTGGATTAGGTAGAGCAGATGTTTATAAAATTA

ATAACATTTATATGGCAGCAGATTTTTCTACAAATGCTATTGCTGGTGATACAGATATTACAGATAGATTTGATTTAGAT

ACAGGACAAAGAGATAACTTCTATGACATTGGAAGAATTAAATTAAAAAATGGTGCATTAACACCAACAGGAAGATTACT

AGTTAATTTTGATTACTTCTCTCACGGTTCTGGAGATTATTTTGATGTAGACTCTTATTCAGGTATTGTTGATTATGAAA

ATATACCAGCATACGAATCATCAACAACTAATGAAAGATTTGAATTAAGAGATACTTTAGATTTCAGACCAAGAGTTGAT

GACGCTTCAACAATAAATTCAGGTAGCCAAGACCGTTCATATGACGGTGTAGGTGCTTCTACAGTTGATTCAGTTAAATT

TGGTACAGACATAACATCCGATTTTGAATATTATTTACAAAGAGTTGATAAAATATATTTAGATTCAGCTGGAAAATTTC

AAGTATTGAGTGGTGCAAGTTCAGCAACACCAGATGTTCCTGGTTCTTTAGATAATGCTATGCATATGTACACTTTATTT

TTACCAAGTTATGGATTAGATACAGCTGATGTTAGTATAGAAACAGTTGACAACCGAAGATATACAATGAGAGATATTGG

ACATTTAGAAAGAAGAATAACAAATGTTGAATATTATACTCAATTGTCTTTATTAGAAGTTGCTGCTCAAACATTACAAA

TACAAGACGCTGATGGATTTGATAGATTTAAAAATGGATTTGTTGTAGATAATTTTACAGGACACGCAATAGGTGACCCA

GGAAATGTAGATTATAAAGTTTCAATGGATATGGCGAAAGGTGAAATGAGACCAACATTCCACGAGGATGCTATTCAACT

TATTGAACGTGATGGTGATGGTACAGCTATTGTTGCCGCTGATAGAACGGATGGACAATACCAAAAAACTGGAGATTTAA

TTACATTACCTTATAGTGAAACTGCTTTAATAACTCAACCGTATGCAAGTAAATCAATTAATGTTAACCCATTTGGAATA

TTTACTTGGATAGGTTCAATTGCATTAACACCAGCAAATGATGAATGGAAAGAAACAGAAAGAGCACCAGAATTAACAAT

TACAAATGATGATGGTACTTGGGATACTTTAGTTGCTGTTTCTGGTAATCCAAATCTTCAAGAAGTAGAGATAGGTACAG

TATGGAACGAGTGGACAAATAATTGGACTGGAACATCAACAACTAATACTACAGAAAATTTTGAACAAAGAGGTGGTCAC

GGTTGGAGAGTAATGCAAAGAGATATCCAAACAACTACTCAAACTGGAACAAGAACAAGAGCAGGAATTAGACAAGTAAT

AGTTCCAAAAACAATAACAAATAGTATTGGTGATAGAATTATAAGTGTAGCATTTGTTCCGTTTATTAGAAGTAGAACAG

TATCATTTACTGCTACAAGAATGAGACCAAATACAAGAGTTTATGCTTTCTTTGATAGTGATGATATATCTACTTACATA

ACACCAGATGGTGGTTCTTTAGGTGGTAATCTTGTCACGGATGCTAACGGTGCTGTAACGGGAACTTTTGCAATACCTGA

TCCAAAAGTAAATTCAAATCCAAGATGGCGAACAGGTCAAAGAATTTTTAGATTAACAAGTTCATCTACAAACGTTTTAA

CACAAGCACCAGATACAGCTGCTAATGCTGAATATATTGCTAGAGGTATTATAGAAACAGTACAAAATACAATTATTTCT

ACAAGAACAGCTGCTATAGAATTTAGAGCAACTAATGAAACAGAAAATGTAACTAGAACGGATGTCCAACGAGGGGCAGC

TAGACAAGTTGGTTATCACGATCCATTAGCACAAACATTTATGATTGATGACGCTGGAGGTGTTTTCTTAACATCTATGG

ATTTATTTTTCAGTTCTAAAGACGCTAATGTACCTTTAACTTTACAAATTAGAAATACTGTAAATGGTTATCCAGGACAA

AATATATTACCGTTTAGTGAAGTATCATTAAATCCACCTTCAGTTAATATAAGTGCTGACGCAACAGTTAAAACTACATT

TACTTTTGCAAGTCCTGTTTATGTACAAGAAAATACAGAATATTCTTTTGTATTAATGGCAAACTCAACTGATTACAATT

GTTATGTTGGTAGATTGGGAGAAACTGTAATAGGTTCAGATAGAACAATATCAGCACAACCATATGCTGGTGTAATGTTT

AAATCACAAAATGGTGTGACTTGGACAACTGAACAAAACGAAGATATTAAATTTACATTGAAGAGAGCAGAATTTGAAAA

TGTAACTGGAGATGTTATGTTGACAAATAATACTTTACCTGCTAGAACACTTAAACTTAATCCATTACGAACAACAAATG

CTTCAGGTGTAGTTAGAGTTTATCATCCAAATCACGGAATGCACGGCACATCAAATAATGTGACCATTGCAGGAGTGTCG

GCTGGTACCTATAATGGTATTGCTCATACTGATTTAAATGGAACATATACAAGTATTTCAAATGTAACCCTAGATAGTTA

TGATATTACAACAGCTGGAACAGCAACTGCTACTGGTGATATTGGAAGCAATACTGTAACTGCAACACAAAATAGAAAAT

ATGATGTAATGAATTTATCAGGTGTTCAAACAATGAAATTACCTGGTACAAGTATTGAATGGTATATTAGACCAACTACT

GGACAATCAATGCACGGTGCAGAATCAGAATTTAGTTTAACTTCAACGGCAAATAAAGTTGCTGTTGTTAATTCTGAAAA

TGTACACTTTAACAATCCAAAAGCTGTAATGAGTGAAATAAATGAAACAAATGAAATGTCAGGTCAAAAATCTTTTTGGT

TAAATTTAAAACTTTTAACTACTAATACAAAAGTTTCACCAGTTTTAGATACACAACGAATGAGTGCTTTTATGATTTCA

AATAGATTAAATAATCCAACTTCTGGTAATACACCAGATTTTGTTGATGATATTGCTTCAACTGGTTCATCTACAGCTGC

TCTTTATTTAACTAAACCTGTAGTATTAGAAAATTCATCAACTTCTTTAGACGTTAGATTGACTTCAAATGTTAGGTCAA

GTTCAGTAGTACAAGTTTATCATAGAACATTAGGACCAGAAGATGATAGAAAGATTGAAGATTTATCTTGGACACCATTT

AATACAGATGGTTCGGAAGATACAACAGTAACACCTGCTGAAGATGACACAACATTTAGAGAATACAAATATTCAGTAGC

TAGTTTAAATCCATTTACATCTTTACAAATTAAAATAGTAATGAAAGGGTCTATCTCATCATATCCTCCAATTGTAAGAG

ATATGAGAACAGTAGCATTGGCGATATAATAAAATGGCAAACGGAATATTGAAAGTTGAAGGACACTCAAATTTAGTTAG

AGATATAAGTACTAATGCTATAGTAAGAACAAGTAATGAATATGCTATCTATATGAAAAGAATAAGACAAAGAGAAGAGA

ATGCAGACCAATTAAGAGGTATGTGTTCCGAGATAAATAATTTAAAGAAAGAATTAAGAGAAATAAAAGATTTAATTAAG

AAGGTTATAAAATAAAATGGCTGTAAGAAATATAGCATTAACAGATACACTAGAACAATTTAGAACAAATTTTAACGATA

TGTGTCTAAATGATTTTGGAGATATTGGAACTTTAGACCCTTCAATGTCGGCAACTAGTGTTATTGGTGCAGTTAATGAA

TTAGCTAGTCAAATTTTTGCTGCTGAAGGTTGGAAGATGGAAGATTCTTCTTCAACTGTACAGCAAATTGGTGCAGGTCA

AACTGCTCAATTTAAAGGTGTTTCAAACCAAACAACTGCTGTTGTATCTGTTCCAGATATTTTAACAATTGGTTTAACAA

ATGTTGTTAATATTACTACTTCTTTATCAGCTCCAATTGTATCTGCAGGAAGTTTAACTTTAACAAATGGTTCTATTACA

GATTCAAGTGGTTCTATAGATTTTGGAGATGACCATATAATAACAACTGGTGACATACAAGCAAATAATATCACAGCGCC

GACTATTACAACTACAGGTGGGACGAATACTTTAGGAACAGTTTCAGTAGTTGGTAATACATTTTCATCTACAGATTCTA

CTGTACTTCATTTTGATGATGATGTTCAAATTGAAGGTGGATTAAAAACAAATACTATTACTGCTCGCACAGGAGATAGT

GTTAATTTTGGTGGTAGTCATATATCAACAAATAATATAACAACAAGTGGGGCTATTTACTTAACAGGTGCAAATAGCAA

TTTAGGTATTTATTTTGAAGGTGCAACAAATGACGCATTTAAAACATATATAAGACCTACAGACCCTACAGCAGAAAGAA

TAGTAACATTACCTGACTCAACAGGAACAGTTGCTTTAACTAATACGACAGGATATGCTGATGGTACTATTTTTACATCT

TCATCAACTTTAGTAATCTACAATTCAGCGGGAGTAGCGCAAAAGACGATTGTTGGTAGTGCTACATAGGAGATTTAATT

ATGGCTATACGAGCTCCTTTATATTATAGCGGCGGCAATTTGAAAGAGATGTCTTCTGCCGAAGTAGACCAAATAATAGC

ACAAGCAATTTATCAATATTCACAAACACCATCTGTTGTATTATCAGTAGTAGGTTCTGGCGGAAACGTAGGATCAATAA

CTGATACAAGACAACAAGCGGGTGCTATGTTAACACACGCTTCAGCATTTCCAAATGAAGCAACAACACCAGAACCTACA

ACGGTAACTATTACTTACGATAAAATAGAGCAAACTGTAACCTCTGGTTCAGCACCAACTGATTCAGGAAAAAGTTGGCC

TGTCTATAGAACAGCAGGTAATGATATTCAAGCAATGTCTTTACAAGATGTTAAAGATACATTTATACATCCTGCTATTG

ATTTATTGACAGCGGCGACCACAACAAGTGAACAAGCTGGAACATATCATATTAATGCTTCAAATAGTGTTGCAGGTTCT

ACTTTAGTTAACGCAACACCAATTTATGTAAATACTCAAGCAGATACAACTGCTTATGCTGCTGGAGATATTCCTGAAGA

TTTAGACCAACCTACAACAATTGCAAGTTATTATTTGCATAGAGTAGATGGTGTTGATACTACTATAACAAGAAGTCCGC

TTTATGTAACTGGTGGAAATGATTTACAAGATTTTACAGAAGTTGCTTTTGAAACATTATTAGCAAGTCATATTAAATAT

ACTGCCGCTGCTTCAGTAGATGGTTATAAAATAACTTATAGTTATGCAAGTGGAGTAAATAGAGGTTCTGGTATGGCAGA

TACAATACTGACAGGTACAGGAGATTATCAAACACATCAAGCGGGTGGAGATGATTATAGAGCGCAAGAGTTTCCAAATG

GTACACCTACAACAGCAAATACTTGGTATTTGAAAATATTAAAATCGTAAATTATAGGAGTATATTATGATTAATGAAGA

AAATTTTATAACAGCACATTTCATAGATAATGAAAGAAAAAATATTGAAGTATTATTAAAAAGTGATGACGGAACAGCAG

TCAATCCACATATACTTGAATATGATGTTGACAATCCAAATTGTCAAGAGTTATTAAAACTTTGTTCTTTAGACCAACTT

CACGAAAATACTTATACTAAAAAACAAGAAGAACGAAAAGCTTATATTAGTCAAATTAAAAATATAGCACAAAAAGAAGG

TCTTATTAAAGATATAATAGAAGAGGTTAATCCTAAATTTATTGAGTTGATGATGGATTTTCTTTTAAGTAATAAGAAAG

AACATATAGACCGTTTATTCAATTTTAAAATATATTTGTTTGAACAAGATGTAGTAAAGAATTGTAAAGACCAAAGTACA

AAATCTGCTATTAGAAAAGCAAAAACTCCTTTAGAAGCACTTAAAATTTATATTAAACTTTGGGAAGATACCAATAAGAT

TTAATCCATTCTTCTTTACAAGAGTGTATATCAGTTTCGGGTCCAGCAAAGTGTACTATTTTTATATTAGGATGTGGTTT

ATCTAATATCATATAATCTTCTTCAAATCTTTTACAATACTCTACATTATTTTCTAAATTTTGTTCTTTATCTAAAGTAT

ATTTACCAATCCATTTACCTTTAACTAAAGATATTATTATATCATTTTTAGTTGCTATATCTTCAACAAAGTTTTGTTCA

CCAAAATATTTGTAATGTACTTGTCCTGTATTATAATAATGTAGTTGCCATTTTTCTGGATCTTTTATAAAAGTATCCCA

TATAACTTTACATTGTCCAGATTTAAATTTAAAGAAACCGCCTTGAACTTTTGGTTTTGGTAATCCCCACCATTTGTCAT

AAGATACTAATTCATTATCTACTACAGGCCAACCTATTAAATCATCCATATTATTTACAATAATCTGGTCAATATCCATA

ATAATAATTTCATCTCTAGGATTTTGTTCCATAAATTCTGGATTAAAGAAAGATAATTTATGCCAATGGCGTTTTATTTT

ACTATCTTTTGGTAAAGGAATAACTTTATCTGCTTTAACATTTGGATTATCGCTATAACAAATAAATTCAAATGGTAGGA

AACAATATTTTTTTAATCCATTATAAAGTTTTTCAACATAATCAGGTGAATATTTTCCTTCAAAATAAACACAACATATT

TTAATATTAGTTTTTCTATCTTCTGTAAGTGCGTATTTGTCGTGTGGGTTTTCTCTTGCTCTATTAGCACTATGATGTAA

AGGTACACTACAAAATCTTTGGCAAAGTGTAGCTGGATTAGTTTTTAATTCTTCAAAAAAGTTTTTCCATTCTTTACTAT

TAATAATATCTTCTATCTTTTCGTTATTTGCAAGTTTTAAATGTTTTTGTCTTAATCTTGCTATTTGAGGTTCTATTAAA

GCTATAGGAGTGTCTACCCAACAACAAGGCATTATATAACCAGTTGCTGTATAAGAAAGTTCTTTAAACGATAAACATTT

TGGATTAATCTTTTTCATTTATTTTCCTAGTTGATAAAATTGTTTTTTTAGGACTATGTGTATTTTTAAAAAATCTTTCA

TCTTTTAATTCATCTAATGCTACTTTCCAATCTTTATAACCTTTATTTTTTATATTTTTAAACATTCGTAAATCATCATT

ATAACGGTTAACATAGTAATTTGGATTTCTAGGTTTATATTTGTCACCACCGTAATGCCAACGACCTGAAAAAACTAAAT

CAAATTCAATATTGTTTGCCTTTGCCATACTTCTTGCTTCTTCTATATCATATTCATTATATCTGAAAACAATATATTGC

CAAACAGAAGTTTTACATTTTTTAGCACATAACTTTGCCATTTCAAATAATTTTTCACCATCTTGATGAATTCTATATTT

GTGACTATCTTTAGGTAGACCATCAATACCAAACAACCAAACTCCTTTAGGGTTTGCTTCAAATGCCTTTTCATACCATT

TTACAGGTTTGTGTGTGGATGCTGTATTAATCGTTACTGGAATATTTTTTTTGTAACAATATTTTAACATATTAATCAAT

AAAGGATTAGCAGTAGGGTCTGATATTTGACCACAAAAAAGTAGTCCCTTTTCAAAGTAATTAGCAATTTTTATAAAATC

ATCCCACGGCATATCTTGTCCAGGTACTGGATGTCCAGTTCTTCTTATTGCTTGTCTTTGACATTTAGGACACTCTAAAG

GACACTTATGACTAGAGTCTATATTAATTTTTCTATTTGAGAAATCGTATATCATTTACACTTTCCACAATGTCTTTGGC

ATTGCCATACAGCATTAACATCATTCCAACTATTAGGTAATGTTTCTGTATACCATTTATGATTTATTATATCTAGTAAA

CTAGAAGTAAAAATATTAAGTTCTTTTTTATATTTTTCATATTCTTTCATAACTGGATGTTTCATAAAATCACTTTCTTT

AGAAGGATTGTGTTCATCTAAAAAATAAGGATTAGAGAAATAACAGCAAGGTAAAACTTGACCACTTGATTCAATTTCAA

GTTTATTTGCTTTTTGCCATTTGCAAGTTATATCTATTATTTCTTTTTTTTCTTTTCTTATTTTTTTAACCATTTTACTA

TCTAGTACTGTTAATTTTGTTGTTTGTTTAGTAAAGTCACCAACTTTTTTTTCTCTACCTTCATCATCTGTAAAACAATC

TGCTTCACGTTTGTTATAGGTCTCTTCTTCTTTTGAAAAATCAACACCAGCTTTTTCTAAACTTGCAGTTTCTTTTCCAG

TAAAAAAAGTCATTTCATTACGACCTTCCCAACGGTCAGTTAAAATTCTGTTGTGTTTGGTTGCTCCGTGATCCATAACT

AATTTTTCAATTTCATCTAAATGATTTTCATTATGTTTCCATACTAAAGTATCAACTCGTATTCTTGCTGGAGTGTTAGA

TAACATCTCCATATTTTTTAAAATTTTATCCAAAAAAGTAAACTGTCTATATCTTTCGTGCATTTTTTGTGTGGTGCCTT

CAACAGCAAAAACAACGTGTAGGTTTTTACCTCCTATTGATCCAAACTCCCACCACCAATCTTCATTTCTTAATGAACCA

TTTGTATTAATAGAAATATGTGCTTCTGGATTTACTTGTCTAATATATCTTGCTATATCTAGTAAGTCATTATTTGTTAA

AGGGTCTCCCCACGTACCACAAATATTATAATTATAAATGTGTTTAGCAACTTGTACAGGAAAAGCTCTTTTAAATTGGT

CTAAAGACCATACAATATCTGGTAACCATTCGTGTGCTTTTAATCCAGTTAAGTTTGTTCTATGACATTGAGGACATCCA

GCATTACATCTAGTTGTAATATCAAGTTTTAAATCAATAGGAAATATCCACATTATATTATCTCCCATAACTTTTCAAAT

TTCTTACTAATTAAATGTATTAGTTTAGCAGATTTTAATTCTTTAGTGTGTTCATCTTCTGGCATTACATAAGTGTGCCA

TTCTTTAGGTATATTATACCAATTTATATTATACTTGTCTAATAGATAATGGAAAAAGACTTCATTGTTAGGAAAAAATT

GTTTTGATATTTCTTCTCCAAATAGTTTTTCTTTTTTTGCTGTATATAAAACATCTAAAAGTTCATCTAATTTTTCTGTA

TATTTTAATTGTTTTATAGCATTTGAATTGCCACCTAATATAGCTGTATTTGCTATTAAATAATCCGTATCAAAATTGTT

ATCTAATGCTAACATTGATTTTTTAGCAATAGCTTTAACGTACATACTATATCCATCAAAATTGCTAACTATTTCCTCAT

ATGTGTTTATTTGTTTCTTATAACGTTTTAATGCGCTTGTACTCCATATATTTTCTTTAGTTGCATTGGGAGCGTGAACA

CATATCTTATTCATATCAAACTTTTCAAAAAAGGATTCAGTTGTATTTGGTACTACATCAAAATCAAAATAAAGAACATT

GTCAAATTCTTCACCTAGTTTTTCCCATATATGTATCTTGTATAGATTGATTATATCAAATTGATAACCACTAAATTTCT

TTTTAAATTTTTGCCAATAGGTGTCGTTTTCATAAAGTCTATATTCAGCATTACAATGCTTAGCATATTCTTTCTTAACA

TCTATTAATTTTTGATAGTGTTTTGCTAATTGTATTTTAGTGAATTGATGTTTTTCACTTAAATTAGGTTCATTATTTTC

TATGTATATACTATAGATTATATTTTTCATCATATCTCCATACGGCGTCAAAGTCTTTGCAGACAGCGTGTACTATTTTT

GTTTCTTTTGGTATAAAATGTTGACTATCAAAGAAGTAATGCCACCTTCTATCTAACCATTGTATACCAATTTTATTTAT

ATTTACTTTATATGAAAAGATTGTTTCATTATCATATCTAAACATATCAATAATATTTTGTGGATACATACCATCTTTAT

CATTTCTTAATTTTGTCATTAAATCTATTGTATCTTTAAAATCACCAAAGAAATCTAATTTTAAAATTTGTTCTTTTGAA

GCACCAATGATACCAGTATTGATAACATCATTTTTAGGATCAAGTCCTTTCTCTATAAGCATTGCTTGACAATTAAAATA

CTTTGCTGTTGGACTTCTAATGCCGTGTTTAATTTCATCTATACTCATCATCATTTTATTAATCATAGTATTGTTATTAT

AAACAGCAATATGATTTTGTATATCCCATATATCAAAAAAGGAATCTTTGGTTACAGGTACAGCATCAAAATCTAAATAT

AAAATTTCATCATATTTTTTTGCTAGTTCATATAGTAAATGTATCTTGTAAAAATTTACAATTTCATAACCTGTTAATTC

TGGAAAGTCTTTACGTAGATTTTTTTCAAAGGTTTGATAACGTTTATCATATTCAAACATAATAAAAGTTGCACCTATGG

TGTCAGCATAGTTGCGTTTGGATTCAATTAACCTTTTATAGTGCTTTTTAAATGCATTAACGGTTATGATTGCCTTTTCT

GCTGTGTCTTTTCTATGCTTTGATTTACCATAATGCTCTTTCGCTGGCACATCAATGTAGATACTGTAAATTGCTCTTTT

CATACTCATATAAATAATACTAGACTATTTATTAAAGATTATGGATACTATATTAAAAGTGATTGATGGAGTGACCACGG

ATAGACTGGTCAAGTCTATTATAAATTCACTAAACGAAAATCAAGAGAAAAGTAAAGATTGGCTTATAGAGAAGTCAACG

AAATACTTTAGCTTTTTTAATTCTCCTTCTGTAGTTATAGCGGCAGGTTGGTATGGGCATTTAGCAAATAAGCTAAAAGA

ATATACTAAAGGAGAGATTGTTTCCTTTGATAAAGACCTAATGTGTAAAAAGATTGGGCAAAGACTTTATAAAGATATTA

CATTTACAGAAGCAGATGTAAAGTATTATGATATTAAAAGATTTAATATAGTTATATGTACTTCTTGCGAACATCTTGGT

CAACATTTAATAGATGATTTTTTAAAGAGAAGAAAGAAAGGATCGTTAGTTATATTTCAATCAAATAATTATTTTTCAAT

TGACGAGCATATTAATTGTCATAATAGTGTAGTAGAATTTGAGAAGAGTTTAAAATTAGAAAAGGTATTATATAGGGGCA

CTTTAAAATTGGATAAGTATGAAAGATATATGGTGATAGGATTATGAAGATATTATTAACAGGTAGTGATGGATTTATTG

GTAAAAATCTTTCAGTATGGTTATCAGAAAAACATTTTGATGTAATTGGTTTAGACCGTAATACAGGTAAAGAATTACTT

ACCTGTGATTTAAAATATGATGTTGATTGTGTTGTACATCTGGCAGGTCTATCTGGTGTCAGACAAAGTTTTGAAAATCC

TACAGACTATTGGAAACAAAACGTTATCGTAAGTCAAAGAATATTTGACTATTTTAAAGATACAAGAATTTTGTATGCAA

GTTCAAGTACTGCATATGAACCCTGGAGAAATCCTTATGCAATGAGTAAGTATAGTATGGAACAAATTGCTCCTGCAAAT

AGTTTAGGTATGAGATTTACTACTGTATATGGACCAGGTGCAAGAGATACTATGTTGATACCAAAAATTTTAAAAAATGA

TGTGCCATATGTTAATACAAATCATAGTAGAGATTTTATACACGTGTATGATATTTGTTCAGCGATTGAATCTATATTAA

GACAAAAATCAATTACAGCATTTCCTGAAAAGACAGGTGCAATAGATATAGGAACAGGTATTACAAATAAGTTAACTGAT

ATGATGGACTACTTTGGAATTACTCCTGAAAAAAGAGTTGGTGGTGATACTGAAAGACTTGACAACAAAGCAAACATAGA

CGCAATGACAAGTTATGGTTGGGAACCTCAATATGAAGTAAAGAAATATATTGAAGATAATAGGAGAACGAATTAAATAT

GAGTTACTTATATGACACAATAGCAAGATATGGCGACCTTATTCCTTTGAATTGTAATTTAAAGTATAAAATATTTGAGG

AAGGTTTAAAATTATTTGATGATAAATGGGTTCAATATAATCCTAGAAAAAAGATTGCTAGGTATGGTTTAAGTATTACT

AGTTTGGATGGTAATTTTTCTGGCATACCAGATTTGGATTCAATAAAAGAATATAATAGAGAAAATAATTTAAAACTTAA

TGAACCAGATTTTAAAACTCTAACCCCCTTTTGGCCTTATGTTGAATCAGTATTATCAAAATTTAAAAATCATTTAGGAA

GAACTCATATTATTAAGATGTCAGCGGGTGGACAATTTCCATCTCATAGAGACCATTTTGATAGAGAATTACCAACGTGT

AGATTATTTGTTCCAATCTATAATTGTAATCCACCATATAATTATTTTATTTTAGATAATAAAGTTTTGAATTTTGAATA

CGGAAGATTATATTTTTTAAATACTGCTAAAGAACATATAGTATTTACAAGTAAACAAGAATCAATGTTTATAGTAGCAA

ATACTATCTTAACAGAAAAATCTACTGATTTAATATTACATAATATGATGAGTAGTTAAATGAACTCATACCAATGTATA

AATGATATAATAAGTATTGATGAAAGAAAGCAACTTTTGTCTGAAGCAATGAGTTATAATTATGAAAATTATAAAACTTA

TAAAGGAACACCTACAGGAATACAAGTTGTTAGTACATACAAAGGAGCTGTTCCTATAGTAGGTTTGGTTAAAAAAATGA

TTAATAAGACCAACCCTAAATTGAATTTTTTTATAGCAGTTTTTATGAAGTTTGCTCCAAATGATGGAACTGGAATAGGT

AACAATAACTATGGTATTCATAAAGATGACCACCTTGGAAGAACGTCTTGTATTACTTGGGCATTATATCCAGAATTAAA

AGATTTTTCTCCCATAAAATATTATAATGAAGATGAAACTTTTAATGAAGCTGTATATTATAAAGAGAAACCTTTAATCA

TTACAACTAGAAATAATCATAGTGTTGATAATAAAAGTGAAAAAGCAAGATATACTTTTCAAATATGTTTTTATGATTCA

ATAGAAAAATTGGCAGAATTAGACCAAAAGGGAGAATTGTTTATATGAAAACAGATATAAAAACTCTTCGCCAATACGCT

AGATTAAAAAAACTTCCTGAAGTTATTAATCTTGGTAATATTGAAGAAAGTAAAAGATTATCTCTATTAAAAGATGTTGA

TAAAACACCTATAAGTAATGAACGTGCTATTAATAATAGAAAGGGTGTGTATGGTGTAGAACATAATTATTTAACACCAG

CAGGAAAAACTTATAATCAAAGGCATATAGATTACTTTAATATTGATATAGAATGCTTAAAAAATTTTATAGATAAATCT

GATTGGAGATATGCAGAATTAGAAAAAAAATCAAATATACCTGAACATTTAGATAACCCTTATTATTATAGATTAATTGT

TATGTTAAAAGGCGAACACGAATTTATTTCAAAAAAAAATAAAATTATAATGCGTGAAGGAGAGGTTTGGTTTATTAACT

CCGCTTATCACCATTCGGTATACAATATTAACAAAGATAAAAGAATTGCATTGTTAGGAAAAATGGAGATTAATGAAAAC

AATACCAAATTATTACGAGCTAGAACCTGAAAATAACATTTTTCAAGATGTTGTAATTGATGTTACACATAGATGTAATA

TGAATTGTAAGAATTGTTATATTCCAAATAGAGAAATACCTGATATGGATATTAACAAAATGTTAGAGGCAATTAAAAAG

TTTCCTAAAAAAACAATGATACGAATTATTGGTGCAGAACCAACTATGCGTAAAGATTTACCAGAAATGATAACTCTTAT

TAAAAAAACTGGTCATAGGTGTACTTTAATTACAAACGGTTTAAGATTAGCAAATGACTCATATGTAAAAACTTTAAAAC

AACACGGTTTAACACATTGTTATATTAGTATGAATGGTGCTGATAATGATGATTGGTATGAGAAAATAGATGAGTTAAGA

TGTGCTACTAAAAAAGTAAAAGCTCTTGAAAATTTAAAAAATAATAAATTTCTTATTGACACAGGAACAATTATTGTAAA

AGGTATTAATGATGATGTAATAAGTAGACTATTATCTATGTTTGAAAGATTAGAAGTTAAAAATGTAATGGCTAGAATAA

AAAATGTAGGCAATCTTGGTAGAAGTATGTATGATAATGAAAGTGGAAATTATACTATGGATGATTTGATAGGGTTGGCA

AGTAAACAAACTGGTCTTAGCGTAGATTATATTGAATCGTGGCGAAACAAACCTATCTATCAAAACACCGAACCAGAAAT

AGATAGTTTTATTTTTCCATTAAAAAAAGAACAAGAAGGTAAACTATTACATAAAAGTGGTATATGGTTTAAAATAGCAA

ATTGGAAAGGCAATGGCGGTAAAATACCTTTTGCAGGTCAAACTAGAAGAGGTAGATTAACACCTGATTTTAAAGTTGCA

CCTTTCTTTGAACACGTGGTAGCAAATGAAGGCGGATATTAATATAAAAAATGTTTTAAATGATAAAGATACATTATCTA

AATTATCATCTAAAGCAGTAAAAGACTCTTATCATAATTTTCACAATTTTGATAGAAGACTATCAAATTATTTAAATTAT

CATATTGTAGAATATGAAAATGAAGTTATTGCAATGGCAGGAATGTTTCAAAGTAAATTTTGGCCTTCTAATTTTGTAAG

AGTATTAGATAGATGTTATTATTTTAAAAAGGTAAGAAGTAATACATTGAATTCTTATCAAACAGGTGGAATTGCTACAA

CTCATTTATTACCTTTACATATAAAAATAGCGTTAGAAAAAAACTTGATACCTTTCTTTTCAATAGCTGGTATTAAAAGA

AGAGCTGCTATGAAAAAGATGATAAAGAGGTGGAATATTAATCACAAACACAAATTAGTGCTGTTGCCTAAAATGTACTT

TACCTGTAATCAAAATGTTGATGAAAATCCAAACGATATTTTTTGCTGGCAAAATGTTGCAATTTTAGACGTTGATGGTT

ATAAAAATTTTAATTTACCTTACCGTGAATTAAAGCGTAACTGAAGGTTCAGTTTCACCAACTAGTTCCTCAGTAAATAC

TATATTATTACTTGAGCAATAGTTTTCTCTAGCAGTTTCATTAGCAACTCCTATATCACTTTCTTGTAATAAATCGGCAG

TTGCTCCATCTTTGTAATTTAATTTATAATATTGCTTTAAACCATCATTTGACTCAAGCTTTTCGTAAGATATAATAGAT

GGATTAGCTCCATCATCTGCTTTTAAGCTTTCTATATATGCTTTTGCTTCATCATTAACTTGATACCACTCCGTATCAGT

ATTTGGTCTGGTGTATGTAATTAGTGTCCAGAAACTCATAAATATTCTCCTTTTAATATGTAACATTATTATTTATACCT

GTATAAATAGTAATATGAGTTTAAAAATGGAGATAAATGTATGATAACAATTGACAAAAAAGAGTATGATGAAACGAAGT

TTAGTCCTGAATTACAGAATTGCATAGCAGTAAGACAAGAAATCCAGGTAAGCAAAACTAGACATTTAATTGAGATTGAA

AAGATAGATGTTTTAACTAAATATTACAACGAAAAAATAGTCAAATTGATTAAAAAAGAAGTACCAGAATCCGAGAAAAA

GTAAATGGCAGCAATAGCTAATCTAACGATAGACCAAGGCGCAACCTTTAGTTCAGACGTAACTGTAAAGGATGCTAATG

GACAGGCGTTTGACCTAACTGGTTATACGGCGGCGGCGAAGATGGCTAAAGGTTTTGCTTCCACAAGAACAAGAATTAAT

ATGTCTACTTCAATAGCAGCAGACGCTACCACAGGAGTAGTTACTCTCTCATTAACAGCAACAGAAACAGGTGATTTGGA

TGCTGAGAGATATGTGTACGACCTTGAGATTACAAAGGATGCTGGAGTTACTAGAGTTATTGAAGGCATTATAACTGTAA

GACCACAGGTTACTGTCTAATTAAATATTATAAATATTCACAGGAGAGAATTGGATGGCAGATATTACAGCCACGGTAGG

GCAAAAAACTACTACAACAGCAAATATAAATGTAAATACTGGAGATGGTCCAGAGGCAGTTTCGGTAACTTTACCATCTA

CGGTAGCAGTACAAAATTCTTCCCTAAAATTTGCTCTTCTTGGTGATGTTGACACAACACATTTAGATGATGGCGCAATG

ATTCAATATAGGTCTAGTGATAATAAATTTGTAACTAGAACCGAAATAGTTACTACAACAGGAACACTATTATTTAATTG

TGGGAGTTTTTAAAAACTAGCATATGGCAACAGTAATACAGATAAAACGTTCATCAAGTACTTCAGCACCAGCTACATTA

AAACTTGGGGAATTAGCATTTACTTATGGAACAGGAACTCAAGGTAATCTAGGAGATAGATTATTCATTGGGGAAGGTGG

AGTTGATGGAAATGGTGACGCAAATAATATATCAGTTATCGGTGGACAATATTTCGCTGAAATTTTGGATCACGTACAAG

GTACATTAACAGCAAACGGTGCTGTAATAATAGATTCAAATAAAGCAATAGACGAATTCATTGTAGGTAATTCTACTAGT

GTAGGTGGAACAATAAAATTTAATGAAGGTACTAATAACGGTACTAACTTTGTAGGACTTAAAGCACCTAACTTACTATC

AGCTACAACAACATTTGCATTACCAGGTGCTGATGGTTCTCCTGGTCAGTTCTTAAAAACTGACGGTGCTGGTAATTTAG

AGTTTATGACTGTTAATCAATATATTGATTTAGCAGGTGATACAGGAACAGATACTTACAATACAGCTGAAACATTAACT

TTCGCTGGTGGCGCAGGTATGGATACAGTTGTTACCGATAACAATGTAGAAATTCAGGCGAACACATTAACAGATTCAAA

TTTATCGGGTAGTGCAGGTATATCAAATTCTAATTTAGCAAATCCTACTACAACATTAGGTAGTTCAGTATTAACTTTAG

GTGCTACTGAAACAGATATTGCAGGATTAACTTCTTTAGTAATTGATGACATTACAATTGACGGTCAATCAGTTACAACA

ACAGCGGCAAATAAAAATATTAATTTAACACCACACGGAACAGGTACAGTTATTTTACCAAGTGGTTATGAAGATAGAGC

AGGATTTCAAAATCAATCAGTTGCAAACAAAGCATACGTTGACCAAGTTGCTCAAGGTTTAGATACTAAACCATCTTGTA

AAGCAGCAACAACTGCTGATTTAGTAGCAACTTATAATAATGGAACATTAGGTGTTGGTGCAACATTAACAGCAGATTTT

AACGGTGCAATATCAGTTGACGATATAGCATTAAGTGTTAACGATAGACTTTTAGTTAAAGACCAAACAGACGCAACCGA

AAACGGTATTTATAAAGTTGACCAAGTTGGTACTGGATCAACTCCTTTTATATTAACAAGAGCAACTCCAGAAGACCAAC

CATCTGAATTAAGTGGTGGTTCATTTGTATTTGTAGAAGAAGGAACTATTGGTTCTAACAATGGATATACATTTACACAT

ACAGGACAACCAGTATTTGGAACAACTGATTTAGATGTATCACAATTTTCTGGTGCAGGTCAAATTACTGCAGGTGCAGG

TTTGATTAAAGATGGTAATACGATAGATACAAATCCTGATAATAGTTCAATTGAAGTTTCAGGTGACCAAATAAGAGTTA

AACCTTTAGGTGTTACAGATTCAATGATTGCAAATAATACTTTAACAGGTGCTAAATTTGCTGATCCTCTTTATTTCAAA

GATGAGTCTTCAACACAAGGACAAGTTTCAATTGGAGGCACTTTAGAATTTTTAGCAGGTGAAGGAATTAATACAATTGC

TAGTGGCAATCAATTACAAATAGTTGGAGAATTAGCAAGTACATCAAACATTGGTGTTGCTTCTTTTTCTGCTGATAACT

TTACAATAACAGCTGGTGACGTTGAAGTAACTACAGTAGATGGAGGAACTTTCTAATGTTTGGTTGGATAAAAAGATTAA

TTAATAAAACAGTTAGTTCTTACGAACCAGTTAAACCAAAGACTACTACTATTACAATTAAAGATTTAAAGAACAAAACA

AAAAAAGAATTAGAGAGAATTGGTAGAAAATTAGGAATTGAATTAGATAGAAGATTATCTAAAACAAAATTAGTAAATAG

AATTAAATTTAGGGCGAAATTAAAAAGGAAGAAATAAACTATGGCAACAAAAATAAAACCATACCGTACAGAAGTAGCAA

CTCGTATTCCAGACGCAAATAATATGGATGTTGGAGAGTTGGCTGTTAATGTAACAGATGGTAAATTTTATATAAAAAAA

TCAGCTGGACAGATTAAAGAAATTGGTGGTGCAGGTTCGGTAACTTTGCAAGACGCAACTAGTAATGGTTCTATTACAAA

TAGAGATATTACTATGAACGGATCAAATTTTATATTTGAAGGGAATTTAGAAAATGCGTTTGAAACTACTTTATCAGTAG

AAGAACCAACAGCAGATAGAATATTAAAATTACCTAACACTTCAGGTACTATTGGTACTTCGGATGACGCATTAGCATAT

TCTGTAGTTTTTGGTTCATAGGTTTGTTGAAAGATTATGCCGTCAACATTTAAAAATGCAGGAATGACTGTAGGGGTTTT

AGATAATTCCTCAGCAGATTTATATACAGCAGGCGGTTCTGAAACTGCTGTAATTCACGCATTATATATTTCAAATAAAA

GTGGATACAGTACAGCAAGAGTTAATGTAAAAGTTACTACTGACGGTGGAACAACTTATAGACATATAGGTAGAAATTTA

GAAGTTCCTGCTAGTAATACATTAACTTTAGATAAACCAGTAAATTTGGAGAACAATGACATATTAAGAGTGGTCGCTGA

TCCTTCTCCTGATTCAACTTCTGTTGATGTTGAGGCAGTAGCAAGTATATTGGCAATAACTTAATAAATAAATATAGAGA

AATAAAATGGCTTATATAATCCCAGGAGAAATCAAAAAACAAAAAGTATTCAATGGTATAAGACGTACTAAAGAGGGTAT

GTGTTATCTATCTTCTATTGATCCAAATTTTACTACTCAACCAATAGAAGTATCAAAGTACTATGAAGATGGTAAATCTG

ATAGTGTTGCTAGAGATGAAGGAGATTACCTTGAAGAAAGATTAGAGATGTTTGAAGTTCAATATTTCACAGGTGACGGT

GCTACCAAACAATTTACAATATCAACACCAGTTTTAAATGAAACAAGAATAGCTTGTTTTATGGATGGTGTTAGACAAGA

AGCATTTTCAACCTATACATTAACAGGTGGAACATCACTAAATTTCGTATTAATTCCAGCGTCAGGTGCTAGTATTGTGG

TTGGTCAAATTAATAAAAGATACTATAATAATGATAGCGATAGGTACCAACAAATTAAATATTCAGATGATACCACAACT

ACATTTCTTATAAATAGTGATAGTGGAGATTTAGTTAGAAGAAGTAAGCAAATAGCAATAAGGTCAGAATTAGCAATTGA

TGACTTTAATACTTTTGAAGATTTAACGTCAACGGTTAATGCAACGACTTATCAAAGCGCTGTTTAAGATGGACAAAATT

AGTAGGTAAATAGAGAGAAAAATGGCAGATTTCAAATTAGGACGATTAAAGTTTAAATGGAGAGGCGATTGGTCAGGAAC

AACTGGCTATGTTATTGATGATATCGCAAAATATGGTGGTAATGCTTATGTGTGTATTCAAAATCACACGTCACCAGCAA

CAGAACAAGATTTTTATACAAGTCCTGGAACATTTACAGAATATTGGCAATTACACCAAGAATCATTTTACTTTAAAGGT

GCATATGCTGACGGAACTTGGTACAAATTAAACGACCTAGTTTCTTATGGTGGTAAACAATACCGTTGTACTACTCAATA

CACATCATCAGGCACAGTTTTAGACCAGTCTAAATTTGAACAATTTAGTGATGGTATTATTTTTAAAGGTGATTATGCTT

CTAGTACACAATACAAATTAAACGACCTAGTTAAGTATGGTGGTAGAACATATAGATGTACTACTGAACATACATCAGCG

GCTGGTGGAGATATCAATATAGTTTTAGGAAACTTTGATATCTATAGTGAAGGTTTAGCATTTAAAGGCGACTTCCAAGT

TAACACATATTACAAATTAGATGATGTTGTTAAATTTGGTGCATATCAATATAAATGTATTGTTGCTCATACTTCGGGTG

GTGCTCTATCAGATTTTGCTGAAGAAAATTTTTCAGTTTATTCAGAAGGTTTACAATTTGAAGATTCTTATAACGCTGCT

ACAGTTTACCAACAAGGTGATGTAGTAACTTATGGTGGGTATTCTTATGTTTATGTTCAATCAAATGAATCTTCTGGCAA

TACACCAGGAACTCCAGCTGTACAAGAAACAACAGGTGGAGATATTACTACATCAACTGCTCACGGAAGAAGTGTTTCCG

ATTTAATTGAAGTAAGAGATATAGTAGTACAATGTGATACAGGACAGAAAACATATCCAATACACTCAACTTCTACTCAA

TTTACAGTAGAGGCAACAAATTTAACAGCAACTAATTTCCAAATTGAATTAGGAACAAGTGCTATTGCACAATCTTATGT

TAGTGGTGGTACGGTTCTTAAATCTAATGGTACTAGATTAGCAATTACAGGTTTTGTTTATAATACAGCAACTGGTAAAG

TAGTAATTACTACAGCAACGCACGGATTATCAGCAAGTGATACAATAGATGTATTTGGAGTTCAAACAACTTGTGCTTTT

GGAACTAAAGTTTATCCACAAGCACCTTATTCAGGACTTTATCCTGTTAAAGCAAGTCCATCAGCTACAAAATTAAGTAT

CTTTTTAGCACCAAGTAATATTGACCATACTTATGTAAGTGGTGGTACAGTTAAATTAGCAACAGTTTCAAATGTTGGAA

GTTCAACTGCTCTTACTGGTTTTTCTTATGATAATGCAACAGGACTTATTACAGTAACATCTGCTACTCACGGATTAAGT

AGAAATGATTTAGTTAAATTAGATAGTATAGTAGTTGAATGTTCAACAGGACAAAAAACATATCCTAATACTACAAACTA

TTCAGGAATATTTAAAGTTTATGATGTACCTGATTCAAGTACATATGTTGTTGCTACTGATAAATCAGCAATTGTTCATA

CTTATGTAAGTGGTGGTACTTCTCAAAAGATTTCATATACTACAAGCGCAGATAAAAATATTTCAAACTTTATTTATAAT

CGGTCAAATAAAAAGTTTTGGGATGTAGTAACTACAGGTTTTAAAGCACAAGGTGTTTATGTACACGGAACATTATACAA

AACTGGTGATACAGTTCAGTATGGTGGTAATTCTTATGTATGCGTATTAGACGCTCAAAGTCAAAGACCTTCATTAAATA

CTGGTATTGTAAATACAACTTATTGGTCTTTAGTAGTTGAAGGATTTAAATGGACAGGTGCATATAGTACATCTACAACT

TATAATATTGGTGAAACAGTTAGATATCTTTCTAACTCTTATGTTAATTTAAAAGACCAAGTTCTTAATATAGAACCAGG

TACAGATGGAACAGTTTGGCAAGGAATTGCTTTAGGTGACTCTGGTGCTGTATTACAGACTCGTGGTGATATGATTACGC

AGTCCGAAGCTGGTACTGCTAGATTACCTATAGGTCTTCCAGGTTCAGTATTAACTAATGATGGTTTTGATGTTCTATGG

TCTGGTAATTCAGCTAAAAATGTTATATGGGTTTCTCCAACAGGAGTAGATGGTGATTCAGGATCAGAAAGTCAACCTTA

TAAAACATTAGCATATGCTGTTAAACACGCAAAACATCACGCTATTAGAGAAATAAAAGAACAGTCTGGTGGTATCGGCG

GTACGGAAGACGTTTATAATAATATTCGTGGAATTTCTTCAAGAGAATTTGAAGTTAGTCAATATAATATAACTGCTAAT

TCTTTTGAAATTCAAATGGGAACTGACACCAATGCTCATACTTATGTAAGTGGTGGTACAGTTAGAAAAGCAGATGATAC

TACTTTAACAATAACTAACGCTCCATATGCTCACGGTTCAGGTGTTATTACAATTCACACTTCTACAGCTCACGGATTAT

TAGCAACTAATAAAGTAAGATTATGGGGATTAAATTATACTTGTTCTAACGGTGCAAAAACATATCCAGAAGTTGGTGGT

CCATCACTCTATAGAGTTAATACTAAAGGTGGTTCTGTTAAAGTTGATATAGTTAACGGTTCAGCTAACCATAAAGTAGA

TGAGTGCGTTAGAATTGATGGTAATGATATAGGGTTTACAGGTCCTACAGGTGGAGATGTAACTACAGCAATTCCTCACG

CTAGAAGTGTTGGAGATTTAGTTGAAATAAGAGATTTACTAGTAAGTTGTCCTACAGGAAATAAAACATATCCTGTAATT

TCAACAAGTACAGCATTTACAGTAGAAGCAGCAAATTTAACAACAACAACATTTCAAGTTGATATTGGAACAAGTACTGT

TGCACAAACTTATGTAAGTGGTGGTGAAGTTGTTAAATCAGATAACAGTAGACTAGCAGTTACAAATTTTGTTTATAATA

TAGCGACAGGTAAAGCTGTACTTACTACAGCAACGAATGGATTATCAGCAAGTGATACAGTTAACTTATTTGGAATTAAA

ACAAATTGCGAATTTGGTGATAAAGTTTATCCACAAGTTCCAGTTTCAGGAGTTTATCCTGTTGTATCAACAAAATCAGC

TACAGAATTAAATTTCTTTTTACCACCAAGTGATGTTGCACATACTTACGTTAGTGGTGGTACAGTTAGATTAGCAACAG

TTTCAACTGTTGGTAGTTCATCAGCAGTTACTAATGCTGTCTATGATAATCTAACAGGACTTATTACAGTAACAGCTACT

TTACACGGATTAGCAAATGGCGATTTAGTTAAATTAGATAGTATATTATTCTCTTGTTCTATGGGAAGTAAAACATATCC

TGATAATACTTTTAGTTCAGGAATATTTAAAGTTTATAATGTAGTTGACGCCAATACATATATTTTTGGTACTGACAAAT

CAGGATTTGCACATACTTATGTAAGTGGTGGTACTTCTCAAAAAGTTACCTATGTAACTAGCGAACAGAAATCTGTTGAC

GCTTTCACTTATAAACGAGTTGGTGGTTCTAATGTTTTAAACTTTAAAGTTAAGAGTACTGCAGGAGATACAATCAGACT

TAAAAACGGTACTTTCACAGAACAATTACCAATGAGAGTAAGAGAAGGTGTTTCAATAGTTGGAGAAAGTTTAAGAAATA

CAAGAATACATCCAGCAAGTGGTACAGGTTCTCAAATTGCAACAGTAGAATTATTACAAAATGCTAGTGGTGCAACAGAT

GGTGCTTACAATTATATTCACCAAGGTAGAGCCGAAAGAAGTTATACAGTTTTAGATGTACCAGCCGCTGATTCATTTAC

AATCAATGTAGGTACTGATCCTAGAGAACACTCATATGTTGATGGTGGTATAGTTACAAATGCCGCTTATGGTAAATTTA

CGGTAACAAATGCTCCATATGTTCACGGTACAGGTGTTATTACAATTACAACTTCCACTAACCACGGATTATCAGCAAGT

GATACTATCAAATTATCAGGTTTAAAATATCATTGTGATGAAGGAGAAAAAGTTTATCCACAAAATGGGGATGCTTCAGT

ATGGAACGTAGTGATATCAGGTGGTGTCGCAACACAAATTATAACTTATCACGGTGGTATTAATTTTGAAGTTAATGATA

TAATTACATTAAACTCGGCAGATGTTGGTACTGGTGGTGACATAACATTAAAAGTTAAAAAATTAGAAAATAACAAAGCT

TGTAACTGGTTATTACTTAACGAAAAAAATAATATAAGAAATATGACTTTCTTGGATCTTAATGAAAAGAAACAGTCAGG

AGGATTATATCAAGTAACAGTAACATCAGGAACAGAATTTACAGTTCAAATGGGAACATCAACTTTTGTACATACGTATA

AGAGTGGTGGTCACGTTATAGCAGTTGGTAATGAATCAGTTAAATTAGGATTATCTAATATTCTTTACAATAATGCCAAC

GGTAGGGTTACAGTTACTACAAGCAATTCTCACAGTTTAACTACAGGTGATTGGGTTACTTTAGGGAAAATGAAATTTGA

ATGTGATTTAGGAGAAAAAGTTTATCCAAGTGGTCCTTACGAACAAGCTCTTACATCTTTAGACCCAATTGGTAATATAT

ATCTTACTTCACCTTATGTACAAAACTGTACATCTTTAAATCCAGGTGCTTGTGGAGTTCAAATTGACGGTAATCTCCAC

TTACGTCCTTTCCCACGAAGTTATAAATCAATGTTGGCAAATGACTTTACACAAATTAATGAAGATGGAATTGGTATTCA

CATTTTAGGATATGGACGTGTTGAAGCGGTGTCAGTATTCGTATACTATTGCGACAAAGCCGTTTATGCAGAATCAGGTG

GATTTATTCGTGCCTTAAACTGCTCACACGCATATGGAGAACAAGGTGTTGTTGCTTCAGGTACAAACGAAGCAGAAGTT

CCTATCAATCTTAAATCTCGTGGTATGATGTTGCAATGGGACAAAGATACTTTTGGTGGAACAGCAACTGCTTCAGATAT

AGAAAATTCAATTGCAGTACAAGGTCAAGGTACAGCTACAATAGTAGGTAGTGAATCTGGCGCAACTGCTACACTTTTCA

GATATAATGTATCATTACTATATTTACATATAGAAAATATTACTGGTAACTTTAAACAAGGTGAAACAGTTACAATTACA

AAAGAAGATTCAACAACATTTACTGTTGATTTGGATGGTTACTTTGGTGGTCAATCAGGTTCTACAGGCGCAGATGTAAC

TACAGCACTTGCTCACGGAAGAAGTTCTGGAGAATTAATTGAAGTATCAGATGTAATATTGAGTTGTCTTTCTTCTGATT

CAACAGTTATAGGAAATAAAACATATCCTGTATCATCTACTACTACACAATTTACTGTAGAGGCACCAAATTTAACAGGA

ACAACATTCCAAGTTGATTTAGGAACAAGTAATACAGCACAATCTTATGTTAGTGGTGGTACACTTCTTAAAACTGGTGG

TACTAGATTATCAATTTCAAATTTTGTTTATGATATAGCGACAGGTAAAGCAATAATTACTACACCAACACACGGATTAT

CAGCGAGTGATACTGTAAATTTATTCGGAATTAAAACAAGTTGTGTATATGGAGTTAAAATATATCCACAAGTACCTACT

TCAGGAATCTTTAATGTTAAATCATCTAGTTCAGCTACTAAATTAAATTTCTTTTTAGCACCAAGTGATATTGAACATAC

TTATGTTAGTGGTGGTACAGTTAAATCAGCAACACCAACTAGTGTTGGTAGTGCAACTGAAATTAGTAGTGCTAGTTATG

ATAATGTAACAGGACTTATTACGGTAGGATCAACTGGTCACGGTTTAATAGTTAATGATTTAGTACAAGTACAAGGAATG

CAATTCACTTGTTCAACAGGTAGTAAAGCATATCCTGATGATATATTAAGTTCAGGAATATTTAAAGTTTATGATGTGCC

TGACGCAAATACATATATCTTTGGTGTTGATAAATCAGCAATTGCTCATACTTATGTAATTGGTGGTACTTCACAAAAAG

TTACCATCGCTACAAGTAATAGTGTTAACGTTTCAGGTTTTGTTTTCAACAGAACAGCTGCTGCTCAACAAGGACAAAGG

GGTCCTTTGATTGCAATGAAATCAGGTACAACAACTTTAAATGCTGTTGATATGATAGCATTAGCAAGTAATGTTAAATT

CCCTAATGATAATACATTTTATAGAGTAGGGTTAGTATCAGAAGAAGATACGAGTGCTGGAACAGCAGTAATAAGATTAA

CTAGTAGCATTGGTTTGAGTAAAGCCAAAAATGAGGATACAGTAAATGACATAACAAAAGAATATTCAAATATTCGTTTA

ACAGGTCACGATTTCTTGGATATAGGTACTGGTGATTTTACTTCAACTAATTATCCATTACCACCTTTACAAGCGTCTGA

CCAGTCAGATGAAGTAATTGAAGTTAACGGTGGTCGTGTATATTGGGTATCAACTGACCAAACTGGTGACTTTAGAGTTG

GTGATTTATTCAAAATTGAACAAGCGACTGGTAGTGCAACATTAAACGCAGACGCATTTAACCTTTCAGGATTAAGTGAA

TTAAAACTTGGTTCTATTGGTGCAGAATTAGGTGCTGCCATAAATGAATTTAGTACAGACGCAACTTTAGGCGGTAATTC

AAATACAGCCATACCTACTGAAAATGCTGTTGTCGGTTATATGACAAGAGATAATGCAGGTACAGGTGCGTGGGTTCCAC

CAACAGGAACATCAGCACAAAGACCTGTAGGCGGTGAATTATTTGCAGGTGCTTTAAGATACAATTCTTCAATAATTTCT

TGGGAAGGTTATAATGGAACAAGTTGGACAGGTCTAGCTGGAGGAACTCCTTGGACAACTCTAGTTGGAGATGGTTCAAC

TGTACCTGTAGCAATAGGTGGACAAAGATTATTAATAGATACAAGTTTATTTGCAATGACAGTTAAATTGCCTGCTAGTC

CACTAGTAGGAGATTCACTTGTATTTTTAGATTTAAACGGATCATTTCAATTAAGACCTTTAACTGTTGATAGAAATGGT

CAAGATATTATGAATTTACAACAAGATATGATTGCTGATATCAACCACGCAGGATTCACTTTAGTTTATACTGGATCAAC

AAATGGTTGGAAATTAGTAGAAGTAGCGTAATAAATAAATATAGAAGAGAATTATAAATGAGTAGATTAACAGATTTTAC

AGTCACATCCGCTGAGAAAGATGACTTTTATGGATTCCATAGAGTTGCTCCTTCTCAAACGATACATAGAACCCTTACCT

TAATTACTGGTAATGAAAGTGTATATGAATATACATTAGGAACAGGTTGGGATATTTCTACATTAGCATATACAACATCT

TATTACATAGGGTTTAATGATTCAAATCCATTAAACACAACGTTTAGTACTGATGGAACAAAAATGTTTGTTATGGGTAA

TGCAGATAAACACGTTGATGAATATACATTGACTACAGCTTTTGATGTTTCTACAGCAAGTTGGAGAACACATAAAGATG

TATCTGCTCAAGATGATAATCCAAGGTCAGTAAGATTTAATCCAACAGGAACTAAAATGTATGTTGTGGGTAGAGATGGA

GTACCAAGTGCAGGTATAGCTGCTTGTAATATTAATGAATATGCATTAACTACAGCTTGGGATATTACTACAGCAACTTA

TACAGATTTATTTTCTTGTCTTGCTCAAGATACTGCTATTAGTGATATGCAATTTAATGCTGATGGAACTTTATTAATTG

TTCTTGGCGATACTGGTAATGATGTTAATGAATATGATTTAAGTACAGCGTATGATGTTTCTACAGCAACTTTCGTAGAT

GCTTTTTCTATCGGCGCTCAAGAAACAGCACCAGCTGGTTTAGGTTTTAATACAGATGGAACAAGAATGTTTATTGCAGG

AACAGATGGTGATGATGTTATACAATATCCATTAGTAACAGGTTTTGATGTTTCAACTACACAAGCACTTACACACGAAG

TTGGTTTAACTAATGCTCCTTCAGCACCAACAATGAACCCACGTGGTTTAACTTTTAATGCTGATGGAACAAAACTGTAT

GTTTTAGGAACTGCTGGTACGTTAATGATTGATGGTGGCGAAGATGAATTACCATATAGTCACGTAGCTAGAAGTCAAAA

CACTATGACATTTCTTGAAGGAAATACGTATGTGTTTGATGTTTCTCAAACTGCTTTAGTCGGACACTCATTAAAATTCT

CAACAACAGTTGATGGTACACATAAAGCAGGTGGAACTGAATATGTAACAGGAGTAAGTTCATCTGGAACTCCTGGAACT

CCTGGAGCTACAACAACAATTATTGTTCCAAGTAAAACACCAAGTACAGACCCAGGAAGTGCTGTAGATAAGTTGTATTA

TTATAATGGTGGTCATCCAAGTCAAGGTGGTGAAATTTTTACACCTGAATGGAAAGGCAATTTACAGATAACTTACACAA

ATGGACTTGATGATATTGACACTAGATATAAAACTAAACATCAAGAAGATATATTTGAGGATAGTGTACTATGGAAAAGA

GGGTTGGCATTTACGGTAGTCAACGGAAACCTAACCATAGAAATGGGATAAAAAATTATCTGAATTAACTAAAATGGTAG

AAGAGAACTATTATAAATATAAATAAGGATCAAGAGAATTATGGCAACTATAAATTTAGGAAGAATTAAACCAGTATTTC

AAGGGGCATATAATGCTGCTACTGCTTATGTAGTGGACGACATTGCTACCTATGGAGGCGAAACTTTTATTTGCATTTTA

GCTTCAACTGGAAACGCAACTTCAAATGCAACCTATTGGTCTAAAATAGCCAAAAAAGGTGATGACGTAACACAACTTAC

TACCCACGGCGATATGCTGTTTAGGGATGCAAGTGGTGTACAAAGATTAGCGGCAGGCACACCAAATCAAATGTTAGTAA

CTAAAGGTGCTAGTGCTGATCCTGTTTGGGGTTCTTCAACTTCAATTTTATGGGAAGCAAAAACAGCTAACTTTACTGCT

GTTTCTGGCGGTGCATATATATGCAATACAACAGACGGTGTATTTACAATGACACTACCTGCTTCACCAGCAGATAACGA

CTTTGTTATTATCAATGATGGTATGGGAGTTTTTGATACAAAAAATCTTACAGTTGATAGAAATGGTGAAAACATAGCAG

GAAGTGCTACAGATTTAATAGTAGACAAAAAGTATGCTACTTTCAGACTAACATATAAAACAATACCAGATGTAACTTCA

TCTTTTATTGGGTGGTTAATTTCATAATGAAAGATATAAACTATATAAATAGTATTACAAACAAAATTTTAGGGGAGAAC

ATTTAATGAGTTCATTAACAACACTTTTAAGCGGCGGCAGTTCAGCTGGTGCAATAGACCACAGAAAAGAAGGTCTTCCA

CTATTCGGTATGTGGGGAGATAACTCCGACGGTAATCACAACGTAAACTACAGAGTCTTTGATTCTGGTTTTCAAAACGT

AGGGTCACCTTGGGGTGCTGTATGTAACTCAACAACAAACTATAGATTTGGTATGTTGTCGGATGCTTCTCACGCTTATA

CAGATAATGACCACGGTCAACACGTATCTCACGAAAATTTAACAACACAAGATTATACATCTTGGACATATTGGAACAAA

AGTACGTACCAATGTGACCAATATCCACACGCACAATATTATTCATCTTCAAGAGATGGATTCGTATCTTGGCATTCTTA

TCACCAATACACATCTTCTTTTGAATATCAAAATGGTTGGACAAAATTAAATATGGTTCTTCCAGAAGGAATTAGACCTA

GACGTATGTTTGTTAATAGACGATTTACGTTAAGAGAAAGATATCCAGGTCAGCACGGTGCTCCAAATATAGACACGTAT

GATTATACTTCTCATATGTTGAATACAGACCAAACTTATTCAACTGGTACTGGATACAATGAGAAAACAAAAACTTTAGT

TATGGTTCACTCTGGTGACGAAGGTGGAAATACTTCTAAAAAGATTCACATTTTCAAATCTGCTAAATGTTTAAATAAAA

TAGACAGAATTAAAGAATACTTTGATAACTTAACTTCAACTGAATATTTTTCTGACACTTGGACTAATCAAAATGTTAAA

GATTGGTGCGTTGTTGTTGGTAATAATGACTATGTTGGATTTGGATTAAAACAAAGTAATAGTAAAAGATACGGTGTATT

TGATTGTTCAGTCAAAGGCGGAACTGCTCACCAAACTGGTGCAAGTAGACAATGGTCAACTTGGCAAGATTTTACAGGAT

CAACAACTACATCTTACGGTGCTAATAACGGACACCAATACTACAGTAAATTTATGACGACTTGGGATGGAACTTGGGGA

ATGATTTATTCACCATATTATTACTATGGTCCAGGTATCAATGGTTTCTGTATGAATATAGAAAACCCTAGAAAATTTAT

TTGTATAAACCAAACTAAATCAAGTAGAGCCAATCCTTACTTTGCTTGGGGACGTACAGGTTTCCACGGAGGTTGGTCAG

ACAACTGCGATTCAGAGTCTCATAGAACATATGCTTGGTCTTTTGATCCTACGGATTCAGATGAAACAGTAAGTACACTT

GTTTATCAAGGTGGTTCTTCAGGAGATGAAGTTATACCAAATAACAACTCACACGTTGGAACTACAGTAACTAATAAAAC

TGGAAATTACGGTTTATTCGCTGCTAGAACTTGGCTACACGGAGGTTTCTACTCAACTAACTATCCACTATTAATGCAAA

TTGACTGGTGGGGTCAGTTCGGAAATGCCGAAAATACTTACGGTGGAAAATACGGAACATAGGAGATTATAGAGATGGCA

AGAACATATTACTTTACACTAGCAGGCGAACCTTTTTCACCAAATGCTGAAACTGGAGATGACGCAGTAGCAAAAGGACA

AGCAATCAAAGTTGATGACGTTCCTGATAACATTGAAGCGTGGAGAATGTCAATCAATCCAGATACAAAAGAATTGACAA

TAGTTGGCGGAGCTGGCGGTGATGAAGCGGCTGCTCTAACAGCAAAAGAACAAGCGCAAACAGATGAAGACGCTGCTGAG

AAAACAAAATCTGATGATTTAAATAAAGCTAAAATTGCAGAAGCAAAAAGACTTTCAGACGCTGGTCTAGCTTAATTTAT

GTAGTGATTTTATTATGATAAGTATTATATTATGTATGACATCAAAGAATTAACCAAAGATATACACCAAAACGCTGAAA

GACAAGAGTTTGTCAAAACTCTTATGTCAGGTTCTATTGAACCTAGACTTTATGCAACCTATCTTTACAATCAATTACAA

TGTTATGCTATATTAGAAAAATATGGAATAGAAAATTCTCTATTTCGTACAACTCCTAATTTACCTAGAGCAGAACATTT

ACATTATGATTTTAAAGCATTATGGACAAGTGAAGACCTTCCAACAGTAACTCAAAGTACTAAAGATTATGTTGCTCATA

TTGAAACAATCAAAGAAGACGCAGAAAAATTATACGGTCATATCTATACTAGACATTTAGGAGATGTATCTGGTGGTCAA

ATGATAATGAAAAGAACACCAGGACCTAATCGTTATTACAAGTTTAAACATAAAGAAATAAAAGAGTATAAACGAATAGT

AAGAGAAATGATAAACAGTTATTTAAATGTTTATAAACTTAATATTCTAAATGAAGTTAAATTTTGTTTTGCAACTGCTA

CACAATTGTTCAAAGAAATGAACGATATGGATTATTCAAAACCTTTAATTTTAACTAACGAGGTTAAAGATGATTTGGGA

ACGACTAATTAAATTAGAAAAAGAAATAATCGCTATACTTGATAGACGTTGTAAAGAATACAACGAAGAGGGTATGGATA

GATTTAATAATGATACTTGGACCAACCGTACTTGGTCTAATATGAGTGTAAGACGTGCTCACGTAGACGTAGTGGACGCC

AGAGAAACAAAAGGTCTTTGGATGGCACATATATGTTTATTTCCAAATTTAACAAATGGTGGTCCAATTTATGGATTTGA

TGTTATTGCAGGTAAGAAAAAGATAACAGGTGTCTTTCACGATTTTAGTCCACTATTATTAAAAGACCATCCCTTAACAA

AGTATTTTATAGAAGAGAATAAATGGTTTAAACCATCTAAAGAAAGAGAATTGCCTGATTGGGCAAAGGCTATCTTTAGT

CCTGGTATGATTGCTGCTGGTAGAGTAACAGAAGAAAAAGAATTAAACCAAATATGTACTCTAGCTACGTCTAATTTAGA

AAATTATCTTGACAAAATTGGTCATTATAATAGCGATTCAAAGGAAGAAGATGTAATAAGAGCGCAAAACTTCTATTGCG

AACACCAACAACAAAATCCACACACCCCTAGAGTAATGAAAACTCTTGGACTGCCTGAAGATGATATAAAACTATTCTGT

ACTGATAATTTGTTTCCGAAGATATAATTGTTATTATAAATATACAATAAAGGAACCAGTATGGCAGAACCAGCATCCAG

AGAAACAGTAAAACAATACGCTTTAAGAGCATTAGGTAAACCAGTAATTGAAATCAACGTTGATGACGACCAACTGGAAG

ATAGACTTGATGAAGCATTACAATATTTTGCTCAATACCACTATGATGGTGTTAAAAGAACCTATTTAAAATACAAGTAT

ACAGCGGCAGATAAAGCTAGAATTTTAGCAGATAGTACTGAAACTGAATCTAAAACATATGGTGATTCTTCTGTAGTAAA

TACAGAATGGAAAGAAGGCAATCAGTATATTGTATGTCCTGAATCTGTTATATCTGTAATTAACATTTTTCCATTTTCAA

ATAAAGGTAATTTAAATTTATTTGATGTTAGATATCAATTAAGATTAAATGACCTATATGATTTTTCTTCAACGTCTGTT

ATTAACTATGATGTTGTATTAAGACATTTAGATTTTTTAGACCATATATTAGTCGGAGAAAAACCTTATAGATTTAACCA

ATTAGATAATAGACTTTATGTTGATATGGATTGGAAAAATGATTTACAAGTAGATGAATTTCTTGTAATAGAATGCTGGA

GAAAATTAGACCCTAACACATATACAGATGTCTTTAATGATATTTGGTTAAAAAGATACGTAACTGCTTTATTTAAAAAA

CAATGGGGAGCCAATTTAAGTAAGTTTGATGGTGTTGCAATGCTTGGTGGAGTTACATTAAACGGTAAACAAATTTATAG

TGAAGCACTAGAAGATTTGGATAAATTGGAAATAAAATTAAGAAGCGAGTTTGAAGAACCGCAACCTTTTATGATAGGAT

AATGCTATGCCAGTTAATCATTACTTTCAAGGTGGAAAAGGCATAGGTAATGCTGCCGAACAAAGACTACACGAAGATAT

AATAGTTGAAGGTCTTAAAATTTACGGTCAGGATGTCTACTACTTACCACGAACATTAGTCAATAAAGATTTAATACTAG

GAGAAGATGTATCTAGTAGATTTGATGATTCTTATTTGATAGAAATGTATTTTGAAAATAATACAGGATTTGCTGGTGAA

CAAGAAATCATAAGTAAGTTTGGATTAGAAATTCGTGATGATACATCATTAATGGTTTCAAAAAGAAGTTGGACAAATTT

AGTTGGTAATAAGGCAACACAGGTTGGTTCTTCTTTATCAGTTACAGGAAGACCAAACGAAGGTGATATTATATATGTGC

CTTTGATGAAATCTTTTTTTGAAATTTTATTTGTAGAAGACCAAGAACCATTTTTCCAATTAGGCAATCTGCCAGTTTAT

AAATTAAAAGTAACTCGTTGGGAATATTCAAGTGAAAAACTTGATACTGGTTTATCTACTATTGACTCACACGAAGATAC

ACATACACTAGACCAATTAGCATATAAGTTTACTTTAGAATATGGACAAGAAGTTATGACAGGTGCAGGTTCAGTACAAT

TAGAAAGTTATCACGATTATTCAACTGGTCAACCAGCACTTTTAATGAACGAAGATTTTACAGAGTCTAATATACAGACA

CAATCTCCATATGCAGATAATTTAGACTTGAATAAAGAAGCAGGATATGATACTGTTTCAACAGCGGATGATATACTTGA

CTTTACAGAAAGAAATCCATTCGGGGAAATTGACGAGTAGACTATATGTTCGGAACACATTTTTATAATCAAAGTTTAAG

AAGACTAACTATTGCATTTGGACAAATTTTTAATAATATAATCGTTCAACAAAAATCTAGTACAGGTGCTGTTACTAAAA

GAATACGTGTGCCTTTAGCATACGCTCCTAAAGAAAAGTTTATAGCCAGAATAGACCAACAATCAAATTTGCAGAAAGGT

AGAACTTTTGCAATTGTTTTACCTAGAATGGGATTTGAATTAAAAGGTTTAAAGTATGACGCTACTAGAAAACTAAACAA

ACTTCAAAAAACAGTTAGAGTTAAAACTTCTGATTCTACTATACATAATTTTAATTATTCACCTGTACCCTATGATATAC

AATTTAATCTTTATTCTTTTACTGCTACAGCAGAAAATGGACTACAGATAATTGAACAAATATTACCATACTTTGCACCA

GATTATACAGTAACTATTAATGCAATACCAGAATTAAATATTAAAAGGGACGTACCTATTGTTTTAGATGATGTAAGTTA

TGAAGATACTTATGATGGTGATTTTAATAAGCGAAGAGCTGTTATATATACTTTAGCGTTTACTGCTAAAACTTACTTAT

ATGGACCTATGGCACAAAGTAAAGTTATTAGAAAATCACAAGCAGATTTAGGAACATCTACGGATGCTCCTTTATCAAGA

GAAGAAAGAATTATAGTAATACCAAATCCTGAAAGTGCTAATGCAGATGATGATTTTGGATTTACAACAAAGATTAGTTT

CTATGATGATACAAAGAAATATAATCCAGTAACAGGAGAAGATGAATAATGGCTAAATTGGAAGATAGTGTAAATGAAAT

ATTGGGATTAGAAGGAACAAATAAAGTTGTTCCAGAGAACCTTGAACCACAAAAAGGTTTTAAACCACCTGTTCCTAGAA

AGAATGGAGAAGTTCCTTTAAAAGTTGAAAAGGATATTAATACTGACTATGATTACAGTAGAGAAAGTTATTACAGTATA

ATAGAAAAAGGACAAGAAGCAATACAAGGCATATTAGATATTGCAAAAGAAGGACAACACCCTAGAGCATATGAAGTTGT

TGGGCAATTGATAGGACAAGTTGGTACTACAGTTGATAAATTACAAGACCTACAAAAGAAATTTAAAGACTTAAAAGAAT

TACCTGGCAGAACAAACGCAAATATAAAAAATGCTTTATTTGTAGGTTCAACAGCAGAATTACAAAAGATGTTGAATAAA

CAAAGTATGGAAACTAAAAAAGAAAAGAGAATTGAAAATGAAACTATTGACGGCAAATCAAAAGATAGCGAATAAAATTC

CTATCGTACTAAAAGACTTAATTTATATTAAGTCAATGACACCATTAAAAGAATTATTAGATGGTGAATCACTAAATTAT

CCAATAGAAGTAAAAGAACACATTGTATCCGAAGTACCTAGATATGGTCCTATGGGTATACCATATATAGAAAAAGAATA

TAGTGTGTGGAAAGGTAGTCAACGAGTGCAGGCTGCTATACAATTAGGTTATACCCATATAGAAGGAGTTATAGTTAAGT

GAAACATTTAGAAGAATTTACAAAAATAATAAATGAATATAAAGAAGATGGAAGATACCGAGTCTTTAATGATATAGTTA

GGACTAGAGGAAACTTTCCTCACGCTATTTGGTATTCAAAATACTCAATTAAAAAAATAGTCAATTGGTGTTCTAACGAT

TATTTAGGTATGGGACAACACTCTTATGTTATAGACTCAATGAAAACAGCATTAGAATCAAGTGGGGCAGGTGCTGGAGG

TACAAGAAACATATCTGGTTCTACTCACTACCATAATGCTTTAGAAACCGAATTAGCAGATTTTCATAAAAAAGAAAAGG

CATTAATATTTACTTCAGCATATAATGCTAATCAAACAACTTTAGAAACTTTAGGAAAAATTATACCTGACTTATTGTAT

ATATCAGACTCATTAAATCACTCTTCTCTTATACAAGGCATTAGGCATAGTAGATGTAAGAAAGAAATATTTAAACATAA

TGATGTAGAAGATTTAGAAAGAATTTTAAAATCATACGAAGGTCCAAAATGTGTAGTATTTGAAAGTGTATATTCTATGG

ACGGAGATATTGGACCAGTAAAAGAAATAGTAGAACTAGCTAAAAAATATAATGCAATAACATTTTTAGATGAAGTACAC

GCTGTTGGTTTATATGGTGCAACAGGTGGTGGTATTACTGAAAGAGATAATATAGAAGTAGATATTATTAATGGAACATT

AGCAAAAGCATTTGGAGTACAAGGTGGATACATTGCAGGAAAAAAAGATTTTATTGACGCCATAAGAAGTTTGGCAAGTG

CTTTTATATTTACAACTAGTTTAAGTCCAGTAATTTGTGCTGGTGCTTTAACAAGTATTAAATATGTTAGAGACCATCCT

GAATTAAGAGAACAAATACACGAAAGAGCAAATAAAACTAAATTAGAACTTGCTAGACAAGGTATAGAAGTTATGAAAAA

TGATAGTCATATTGTTCCTGTAATTATTGGAGACCCTAAAAGAGCTAAAGCAATATCAGATGAACTTTTATATAAAGAAG

GTATCTATGTACAACCTATTAATTGGCCGACTGTTCCTGTAGGTACTGAAAGATTAAGATTTACTCCTACACCATTTCAT

ACAGACGCATTAATCTTTGATATGGTAGTAAAACTAAAAGCGGCTATGAAAAAATGTGGAGGTAAAAGTGCAATACAAAG

TAATGCCAAAACATAGAGAATATATTATACCAACAACTTCTTGTATAGGAGGTTGGTATATTCCTTCTGGTATTTGTGAT

GGACTTATAAATTTATTTAAAGAGAATAAACAAGCACAAAAACCAGGTGTTGTAGGTTTCACTTCAAAAATTAATAAAGA

AGTAAAAGATTCTATAGATATTGGATTAGATCCAAATTGGGAAGAACCAAGGTTTATGAAATATAAAAATGCGTTGAAAG

AATGTGTTGGTCTATACGAAGAGAAATATCCTGAAGTTAAAGAGTTTGAAAGATATGGAATGGTTGAAGGAGGAAATTTA

CAATACTATCCACCAGGTGGAGGTTATTTTACTAAGCATTGTGAAAGAACTTCCAGGCACGAAAACCGTTGTCTTGTTTG

GATGACTTATTTAAATGATGTTCCTAACGGTGGTACACATTTTAAATATCAAAATGCAACAACTCCTGCTGAAAAAGGTT

TGACTTTGATTTGGCCGACTGACTTTACACATACACATAGCGGACAAATTTCCAAGACCCACGAAAAATATATCATAACT

GGTTGGTTTGGGTATCAATTATAAATAGTAATATGCCAGTAACAGACGCATATTTAGGAAATCCTAATTTAAAAAAAGTA

AATATACCAGTTGAATTTACTGAAGACCAAATTGTAGAATTTCAGAAATGTAAAACAGATCCAATATATTTTATGGAGAA

ATGGATGAAAATCGTTTCTCTTGATGAAGGACTTATATCTTTTAAACTATATGATTTCCAAAAGAAGATTGTAACTACAA

TAGATAAAGAAAGATTTACTATTTGCAAATTGCCTAGACAATCAGGTAAATCAACTACAACAATTGCATATCTTTTACAC

TATGCAATATTTAATCCAAATTCAAACATAGCAATTCTTGCTAATAAATCTTCTACTGCTAGAGATATATTAGGAAGATT

ACAATTGGCATATGAAAATTTGCCTAAATGGTTGCAACAAGGAGTTATTAATTGGAACAAAGGTAATATAGAATTAGAAA

ATAAATCTACTATTATTGCTGCTGCTACATCTTCAAGTGCAATACGAGGAGGAACATATAATATAATATTTCTTGATGAG

TTTGCTTTCGTACCTGCTAATATAGCAGAAATGTTTTTTAGTTCAGTTTATCCTACTATTACATCTGGTAAAACTTCAAA

AGTTATTATCGTATCAACACCTCACGGAATGAATCAATTTTATAAATTATGGACAGACGCTGAAAATGGAAGAAATGATT

ATAAACCTATTGAAGTACATTGGTCAGAAGTTCCAGGTAGAGATGAAAAATGGAAAGAAACAACTATACGTAATACATCA

GCAGCACAATTTCAACAAGAATTTGAGTGTGAATTTTTAGGATCAGTAGATACATTAATTTCACCAGTTAAGATTAAACA

AACACCTTATATGACACCATTAACTTCAAGTGGTGGTTTAGATGTATTTGAAAAGGTTGTAAATGGTAGAAATTATGTTT

GTTGTGTTGATGTAGCGAGAGGTGTAGATAGAGATTATTCAGCATTTTTAATGTTTGATGTTACTCAAATGCCTTATAGA

GTTGTTGCCAAATATAGAAGTAATGAAGTTAAACCAATTCTATTTCCACACTTAATACAAAAAGCGTGTAAGGGTTATAA

CACGGCAGATATTCTTTGTGAAACAAATGATATAGGTCAACAAATAGGTGAATCATTAAACTATGAATTAGAATATCCTA

ATCTATTAATGACTACTCAAAGAGGTAGAGCAGGTCAGATATTGGGTGCAGGATATAGTGGAAGAGGTTCTGGTTTTGGT

GTTCGTATGACAAAACAGATTAAAAAAGTTGGTTGTTCTAACATTAAGACATTGATTGAAGGAGATAAAGTTATTATTAA

TGACTTCAATATCATAGAAGAAATGTCAACTTATGCTCGTAAAGGAAATTCTTGGCAAGCGGAAGAAGGATGTAATGATG

ATTTAATGACTTGTCTTGTATTATTTGGTTGGTTGTCTAATCAACCTTACTTTAAAGAAATGACTAATACAAATGCTAGA

CAACAATTATATGAAGAACAAGAAAAATTAATAGAGCAAGATATGGCTCCTTTTGGTTTTGTAGATGATGGTACTCCTGA

TTGGGAGAAAACAGAAGTAGATGAATATGGAACAGTCTGGTATCCAGTTGTCAGAAAAGGGCTCTAAATTAAGTATTATA

TAAATATCCATAGTTATGAAATTTGACTATGGTCGTATGAAAACATACGGAATATGCGAAAAGATACAAACTAATTAGTT

AATTATAAGGAGAAAACCTAATGGCATTTCAAGTATCACCAGGTGTTCTCGTACAGGAAAAAGACTTAACAAGAATTATT

CCTGCCGTTTCAACGTCTTCTGGAGCTTTTGCTGGAACTTTCAGTAAGGGTCCTCTTGACGAAGTTGTAAGTATCGGTAG

CGAATCTGATTTATTGTTAACGTTTGGAAAACCAGATAGCTCTAATTTTGAGAGTTATTTTAGTGCTTCAAACTTTTTAC

AATATTCAAATAACTTGAAAGTAGTTCGTGTACAGAACTCATCTGTTTCAAACGCAACTGAAAGTGGTAGTGCGTTTGTT

ATAAAGAATACTACTGATTACCAAAACAATTATGCTGACGGTTCTGCTTCTGTAGGAATGTGGGCTAGTAGAACAGCGGG

TGCGTGGGGAAATAATTTATGTATTTCTCAATGTCCTTCTGCTACTGCTTATGAAGAAACTGCTAAAACAACTGTTGCTG

ACGCTTCAACAAATGTCGGAGATACAGTAGTTTCAGTTACATCCTCTACAGGAATTAGTGCTGGAGATATAGTTAATTTT

GGTGATGAATATGAATATAGAGTTATTAGTGTTGCTACTAATGACTTAAACATTGTGCGAAAAGAAGAACCAACATATAT

TGGTACTTCTGACTCTTCTGGATTACAAAAGACTATTACAAATGGTGCTAATGTAAGACGAAGATGGACATATTATGACC

TATTTAATAAAGCACCAGGAACATCTACTTACGCTCAATCAAGAGGTGGAAGTGGAGATGAACTACATATAATTGTAGTT

GACGAAGACGGTGGAATTAATGGAACTAAAGGGGAAGTTTTAGAAAAATTTGAAGCAGTATCAAAAGCTTCAGACGCTAA

GAGTCCTCAAGGTGACACTAATTACTATTCAGACGTACTTTACAATTCAAGTAATTACGTTTTCTGGATGGACCACAACG

CTTCTGGATCCAATTGGGGCACGGCGGCAGCAAGTACTGCATTTACAGACGTAACTTCTGTAAGCAAAGTATCATTATCA

AATGGTTCTGACGGTACAACTGCTACAACTGCTCAAGTTAAATCTGCTTATGAAAAATACCAAGACGCTGAAACAACAGA

CGTTGGATTAATCATTGCAGGTGCTGGTGACTCAACACATATAGACAATTTAATTACTATTGCTGAAAACAGAAAAGACT

GTGTAGTTTTTGCAAGTCCTGAAAGAAGTGATGTAGTTAATGTAACTAACTCAGCAACACAAAAAGATAATGTTGTAAAT

TTCTTTAATGGAATTTCTTCATCTTCTTATGTGTTTTTTGATAGCGGTTACAAATATATGTACGATAGATATAATGACAT

TTATAGATATGTACCTTTAAATAGCGATATGGCAGGATTATCAGCAAGAACTGATATGCTTGCAGACGCTTGGTACTCAC

CTGCAGGATTAAACCGAGGTGTAGTAAGAGGTGCTGTTAAACTGGCATTTAATCCAACTAAATCACAAAGAGATGAATTA

TACAGAGCAAGAGTAAATCCTGTGACTACGTTCCCAGGACAAGGAACTGTATTATTCGGTGATAAAACTGGACTAACAAA

TCCTAGTGCATTTGACAGAGTCAATGTACGAAGATTGTTTATCGTTTTAGAAAAGGCAATATCATCTGCTTCTAAAGTTC

AACTATTTGAATTCAATGATGAATTCACTAGAGCTGGATTTAGAAATATGGTAGAACCATTTTTAAGAGAAGTACAAGGA

CGAAGAGGTATCACAGACTTTTTAGTAGTGTGTGATGAAACTAACAACACAGGCGAAGTAATAGATAGAAACGAATTTGT

AGCAGAAATTTTTGTTAAACCTGCTAGAAGTATCAACTTTATCTCATTGCAATTCGTTGCAACAAGAACAGGCGTTTCCT

TTGAAGAGGTCGCTAGCTAATAGAGAGAATAACGGAGAAATAAAATGGCAAACATAAATGATTTCAAAGCTAAACTTTCG

GGCGGCGGCGCAAGAAGTAACCAGTATAAAGTGGTTATGCCTTTCCCAGGCTACGCTCAAGTTGGTGGAGAAATAGAAGA

CCTAGCATTTTTATGTCAAGGTGCTGAATTACCAGGAATGGCAATTACAGCAATAGAAGTTCCTTTTAGAGGACGAGCTA

TAAAAATCGCTGGAGATAGAACAATTGCAGACTGGTCTATCAAAGTAATAAATGATACTAATTTCAAATTGCGTAATGCA

TTTGAAAGATGGATGAACGGTATAAACAATATGACTGATAACGAAGGATTAACAAATCCTGTTGACTATCAAGTTGACGC

ATTCGTAGACCAATTGGACAGAAACGGTAATCAAATTAAGACTTACACTTTAAGAGGTGTATTTCCTACAGCGATTAACG

CAATTCCATTGGATTATGCTGCTAAAACTGATATATCAGAAACAAGTGTTACGTTAGCGTTCCAATACTTTGAAAGTACT

ACAACTACTTAAAAACTACTTATAAATAGTAGTGTATTTTTAAGGAGAATAAATTATGGCTGAACTATTTGGATTTTCTA

TAACAAGGGTTAAGAAACCTCAAGATCCAAAACAAGCATTTACACAACCACAAGCGGATGATGGAACACAAACCATCGCC

GCTGGTGGGTATTACGGTCAATACTTGGATATGGAAGGTCAGACAAAGACCGAGCAAGACCTTATCAGACGTTATAGAGA

AATCGCTTTACATCCCGAGTGCGATATGGCAATTGAGGATATAATAAATGAATCAATTGTTGCAAACGAAGTCAAAGACG

CAATAAGATTAAACCTAGAATATTTACCATTCGGTAAAGATGTCAGAAGAAAAATAGAAGACGAGTTTAAAGAAGTTTTA

AGATTGATGAACTTCCATACTAGAGGTCACGATATCTTTAGAAGATGGTACGTGGACGGTAGATTATATTATCATAAAGT

AATTGATAGAGAATCTACAAGAAAGGGTATTACAGAATTAAGATATATAGACCCTAGAAAAATTAAAAAGATTAGAGAAG

TAAGAAAGAGAAGACCAGATGGACCTACTCCATATGGTTTAAATGTTATTGATGAAGTTAAAGAATACTTTTTGTTTAAT

GAAAAAGGTGTTACAAATACTACATCTGGTGGAATTAAAATTGCTGTTGACGCAATAGCATTTTGTCCAAGTGGACTGAT

AGACCAAAACAAAAATATGGTCTTATCATATTTACATAAAGCAATTAAACCTGTTAATCAATTACGTATGATTGAGGACG

CAAGTGTTATATACAGAATTGCAAGAGCACCAGAAAGACGTATATTTAAAATTGATGTTGGTAATTTACCTAAAGTAAAA

GCAGAACAATACTTACGTGATGTTATGGCAAGATATAGAAACAAACTTGTCTATGACGCAAGTACAGGTGAGATACGTGA

TGACAGAAATTATATGTCAATGCTTGAAGACTTTTGGTTACCAAGTAGAGAAGGTGGAAGAGGAACTGATATTACTACTT

TACAAGGTGGACAAAATCTTGGTGAAATGGGAGATATAGAATACTTTAGAAGTAAATTATATCGTTCTTTAAATGTTCCT

GCTAGTAGATTAGAAGCGTCAACTGGATTTAATCTAGGACGTTCAACTGAAATTACTAGAGATGAACTTAAATTTACGAA

ATTTGTACAAAGATTAAGAAAGAAATTTACTGAAATATTTAACGATATATTAAGAACTCAATTAGTTTTAAAAGCCGTTA

TTACGGATGAAGATTGGTTAGTTATAAGGGATGTAATCCAATATGACTTTTTGCAAGATGGACATTTTGCTGAACTAAAA

GATTCTGAAATGTTATTAGAAAGATTAAGACTTGCCGATTCAGTAAGAGATTATGTAGGTAAGTATTATTCAGTAGAGTA

TGTTCGTAAGAAAATTTTACGACAAAACGATAGGGATATTGAAGACATAAACAGTCAAATTAAAAGAGAAGTTAAAGATG

GTATACTTGCAGACCCTATGCAACAATATACAGCAAATAAAGATAGTATAGAAGGAGATATGTAATGGCAGACCCAAGCG

TTCCAAGTAAGACAGCGGAGTTTATTGACAAATTACAAGCGGGTAAAAACGCAGACGCAGGAGAAGCATTTAAGGATGCT

TTAAGAGATAAAGTAGCAAGTGCTCTTGATAGACAAAGAGTAGATGTTGCTGGCAAAATTTTTAAAGGTATAGAACCTGA

AAAATTTAGTGACCCTAAACCTGCGGTAACGTCAGCAAGTCCGAGAACTGATAAAATTATGGATACAGATGGAAAAGAAA

TAGCTTTTGAACCGACTAAAGAACCGAGTCCAGAAGCAAGTAAACCTGAAGCGCCAACTATGGCACCAGGACACGAAACA

CCACCAGACGCAGGTGTAGGAACACCAGCGCCAGACGCAGGTGTATAGAAATGGATACGAGTTTACTTTTTACAAGTAAG

ATAGTTGAAGATAGTAAGTATCTTGACTCTAAAAGTTATGGAGATTTATCTCCTAAACTAAAGTTAGCAGTACAAGATAC

TTTCAATCTAATTGAAAGAACATCTGGAGATATTATAAGTAAATTTGAAAATTCAGTAGAGAGAGTTGCTGAAGCAAGAA

AAATAAATAAAGAAGAACTATATCAATATTTTGACAAAGAAGTAGAAGAACAATTAGGAGAGTAATATGGCGTGGGTAGA

TGTACCAGGATCAAATAGTATTTGGCAATATGAAAATAGTGCCACAGCATCCAATACGTATGCAGACGCACCTGGAACTT

ATTCAGGTGGCATAAGAACTTATACAACTCCTGGAACAGGACAAGTAAATAAGATTTATGCTAGATGTAGAAAAAAAGGA

ACAACAGTAGAACGTGGCGAATTATCAAAAGATTTTTTTGATGCTACACACGTAGGATTCTAATATGGCAGATACAGTTA

CAACACAAACAATAGCAGATACATCTGGAGTTAAGTACGTAATTAAGATGACTAACTTATCAGATGGTTCTGGTGAAAAT

AACGTACTAAAAATAGACGCTTCAGAAACAACTTTTATGACCGAAGATGGTGAAAGACGTATAGCAAGAGTGTATTATTC

TATCAATACGTCTGATAATAAATCAGGAGTAGAATTAATATGGGGCGGTGTTGCAAATGCTACTGCTTTATTTTTATCAG

GACAAGGAACAATAGATTTAAGAACTGATGGAAACTCATTTCCAAACAATGCTACAACACCTACAGGTGATGTATTGTTA

AGTACAAAGAACTTTGCTAAGGGCGATAACTACTCAATACTTGTTGAATTTAGATAAAAAATCTTATAAATAGTAAGAGA

GAGAACTATGAAACTAATTACAGAAGAAGCAGCCGATTCAAAGTTTATTGTAGAAGAAGTTGGCGGCAAAAAACAATTTA

AAATTAAAGGTATCTTTTTACAAGCAGATATCAAAAATAGGAATGGCAGAGTCTATCCTAAAGAGATATTGCAAAAAGAA

GTTTCAAGATACAATAGAGAATTTATCAATAAAAGACGTGCATTTGGCGAGTTAGGACATCCTGATGGACCAGTTGTAAA

TCTTGAAAGAGTAAGTCATATGATAACAGACTTACATCCTGATGGACATAATTTTGTTGGTGAAGCAAAAGTGATGGACA

CACCATATGGTAAGATTGTTAAAAATCTTATTCAGGAAGGTGCTCAATTAGGAGTATCTTCAAGAGGTATGGGATCACTA

GTGCGTGGACGAGGTGGAGTTAACGAAGTAGGAAGAGATTTTTACTTAGCAACTGCCGCTGATATTGTTGCAGACCCGAG

TGCTCCAGACGCTTTCGTAGAAGGTATTATGGAAAACAAAGAGTGGGTATGGGACAATGGTGTTATCAAAGAGAGAGATA

TTGAAGAGTGGAAACAGTACATAAATGAAGCAAAAAGACTACGTTTAGCAGAAGCAAAGGCAGATGTATTCAAAAAATTC

ATTGAAAATCTATAATCTTATAAATATCTATTAACAAAGAGAGAACTAATTTAAACGTTTAAATTAATTAAGGAGAGTTT

TCAAATGGCTGAAACAGACAAAATAGAAGCGTTAGAAGCAAAAGCAGTGGAAGAGGCGAATTCACCTAATCCTCAAGCGG

ATGCTCCTAAAAAGAATGCTGTGGCGGCTGAACCTTCTCATATTGCTAAAATGAGTGAATATGAAGATTTAGGTAAGGCA

GTAGTTAAACCTACAGACAGCAATCCTGACGCAACTAAAAAGATGAAAAAAGTTTCTGGACAAGCTCCTCAAAAATCACA

AGGCGCTGCTGACGCAATGCCTAAATTGAGTGGACACAACACTAAATTGGAGAATAAAGAAACTAAAGACAAAGACGGTA

AAGAAATTAAGGAAGGCGACTTACCACCAGCACTTCAAAAAGCTATTGACGCTAAAAAAGATAAAAAAGATGTCAAAGAG

TCTGACGAAAAGAAAGACGATAAGAAAAAAGATGACGCTGAAGTAAGAACAGAAGACGAAGACAAAGAAAAGAAAAAAGA

GATTGACGTAAAAGAACACGTTGACGCTCTTATCGCTGGAGAAAAAGACTTAACCGAAGAGTTTAAGGCTAAAGCTGCTA

CTATTTTTGAAGCGGCTATCAAATCTAAAGTAAAAGAAATTACTGAAGAATTGGAAACAGATTATAACAAAAAATTTGAG

CAAGAAAGTGCTAAAGCAAAATCTGAATTAACTGAAAAAGTTGATTCTTACTTAGCATATGTTGTTGAAGAGTGGATGAA

AGAAAACGAAATCGCTCTTGAACGAGGTATCAAAGGAGAAATTGCTGAAGACTTTATCAATGGTCTGAAAAAATTATTTG

AAGACCATTACATTGATGTACCAGATGAAAAATATAATGTGCTTGAAGACCAAGCAGGTAAAATTGAAAAACTGGAAAAA

GACCTCAATGAGCAAATTCAAAAAAATGTTGAGTTAAATAAGGAAGTTGGAACTAAAGTTAGAGATGAAATCAAAACTAA

AGTTTCTGAAGACCTTGCTGACACAGCAAAAGAAAAGTTTGCTAAACTTGCCGAAGGAATTGAATACTCTAACGCAAAAG

ACTATCAGAAGAAATTAGAAACTGTTAAAGAATCTTATTTTGGAAAGAAAAATAATGAAGCGAAAGAGAACCTAGATGAT

GTGGCGGCAGATGGATCAGTTAATCCTGATTTATCAAATTCTATGGCTGCTTACAGCGCCGCTATAAGCAAAACTAAAGA

CATTAAACTGTCTATTAAGTAAATATAAAGGGAGATAAACACATATGTACTTATCTGAAACACACGAAAAAAAATGGCAG

CCAGTACTAGAGCATCCTGATTTACCAAAAATTACTGATGCTTATAGACGTGCCGTTACATCCGTGATATTAGAAAACCA

AGAAAGAGCTTCTAAAGAAGACTCTGCTTACTTGGCAGAAGCGGCTCCAACAAACGCAACAGGTAGTGCTGTAGCGAATT

GGGATCCAATTCTAATTTCATTAGTACGTAGAGCAATGCCGAATTTAATTGCATACGATATCGCAGGTGTTCAACCAATG

ACTGGTCCGACAGGACTAATTTTCGCAATGAGAAGTAGATATACTTCACAAGCAGGCGGAGAATCATTCTTTGACGAAGC

AGACACAGACTTTTCAGGCAGAAATGCTGCTGGATCTTCTGTTGATGGCTACTCAGCGACTGACCACTCGGGTGCAAACC

CAGCTGTCTTAAACGACGGTTCACCTGGAACATATACAGCTGGAACAGCTATGACTACAGCGAAAGCTGAAGCATTAGGC

GACGCTAGCGGTAATGCATTTGCAGAAATGGCTTTCTCAATAGAGAAATCTACTGTAACTGCTAAATCAAGAGCTCTAAA

GGCTGAATACACTATGGAACTTGCTCAAGATTTAAAAGCAATCCACGGTTTAGATGCTGAAACAGAACTTGCAAACATCT

TATCTGCTGAAATTTTAGCAGAAATTAATAGAGAAGTTGTAAGAACTATTTACATTAATTCAGAAAAAGGTGCTCAAACT

GGTAACGTAACAACTGCTGGTATTTTTGACCTAGATACAGACTCTAATGGTAGATGGTCTGTTGAAAGATTCAAAGGCTT

AATGTTCCAATTGGAACGTGATGCTAATAGAATCGCTCAAAGAACCAGAAGAGGAAAAGGTAATATAATTATCTGTTCAT

CTGATGTAGCTTCTGCTCTTCAAATGGCAGGTGTATTAGATTACACTCCAGCTTTAAATAACAACCTAAATGTTGATGAC

ACAGGCAATACTTTTGCAGGTGTTCTTAACGGTAGATTTAAAGTATACATTGATCCTTATTCAGCAAACAGTACTGCTAA

ACAGTATTATGTTGTTGGATATAAAGGAACATCACCATATGACGCAGGTATTTTTTACTGCCCATATGTTCCTTTACAAA

TGGTTCGTGCAGTTGGTCAAGACACTTTCCAACCGAAAATCGGATTCAAGACTAGATATGGCTTAATTGCTAATCCATTC

GCTGAAACTGGTGCTCAATCAGGTGCTGCTACAGCAGTAAATGACGCTGGAAGTGCTAACGCTAACAGATATTACCAAAA

AGTTCAAGTTGCGAACTTGATGTAATTTCATTGGTTTGAAAAACATTTTTCAAACAGAATTAAGGGGGAGTTTTTACTCC

CCTTTTTTTTGGCCTAGATGACTTATAAATAGTATTATGACAATAAAACAATCATACAAAAGACAACCAACTAAATTTGA

TTATGCTGCTCCAACGCAGTTTAAATTTACTATTACAAAACTTCCTAAAGTAGAATTCTTTTGTACAGCAGTTAACTTAC

CAGGCATATCATTAGAAGGTAATATGGCGCAAGAAACACCATTGAAAGATATACCTATTCCTGGAGATAAACTATCATAT

AGTCCACTATCTATGGATTTTATGGTTGATGAAAATTTAGAAAACTATAGAGAAGTACACGGTTGGTTAACTGGTTTAGG

TTTTCCTAAAGATAGAAAAGAATTTAGAGACCTATTAGGTGGTGGTACAGATAGATTTCCAACATCTACTGGTGCGAATC

AAGAAACAGACGCAGGTAAACAGAAATTTAGTGCAGGAGATTCAGGTGCCGTTTATTCAGACGCAACATTAAGTATATTA

ACAAGTAAAAATACAACAAATATTCAAGTAAGATTTAGTGATGTATTTCCTACAGCATTATCTGGATTGAATTACAACCA

ACAACAAACAGATGTCAACTATTTAATAGCAACAGTTACGTTTCAGTACAAAATATATGAATTTGCAGAAGGAAGCGGAC

AAACTTCGGTAACGGTTTCGTAACAAACTTTACTTTTTTATTATAGTATGATATAGTATATAAGGAGATTATGACATTAG

AAGAATTGCAAGAATTAGCAGACAAAGATTTAAAGATTAACGATAGTGAACTTGATTTAGAATCAATCAAAACTCCACAA

ATACATAACAAATATATGAAGCACTTAAGCAAGTTTAAGTTAATGTTAAGTAGGGCCGAAAGTGAATTACATATAGTTAA

AAGAACAAAATGGGAATACTATACAGGTAAAGCAGACTCTACTGTTTATGTTGAAAAACCTTTTAACTTAAAAATATTAA

GACAAGATGTTGACAAGTATATTGATTCAGATGAAGAAGTTATTAAAGCAAAACAAAAGGTTGATTACCTTACTACAGTA

GTAGATTTTTTAGATAGAAGTATTAGACAAATTTCAAACAGAACATTTACTATTAAGAACGCAATTGATTGGAAGAAGTT

TACATCAGGAGCCATTTAATATGGGTGCAGTTAATATAATAAAATTTAAAAGTGAAGATAAAAGAACTTTTTTTGCTCCC

GAGTATGATTATACAATATTTGAAACTCAAGCATTTGAAATTGATTTTAAAGAGTTAGCAAAACTTATCTTAAGCAAAGA

AAAAGAACTATTAAGTTTGCCTATATCAACTACATCAGGTGACGCTTACACAGGACTAAAAAAGGATAGTACAACAACTA

GATTTGATAAGTATAATGTTTTAAAATGGGACGGTGAAAATATACAGCATATAAAAGGAAACATAATAAGTTTTCATAAT

CACATTTTAAAATATTTTAAACAACCACCTGCTAATGAATTGTATATACAATGTTGGACTAATATAATGAGAAAAGGAGA

ACAAATAAAACCTCATCTACATAATATAGGACCAAGCTGTTATTTAGGAGGTCATATTTGCGTACAATGTGATGATACAT

CTACCCATTATATCAATCCAATAAATCAAATTAATGACCCTATGACATATAGTAGTAAAAATGATGTAGGTAAAATGACT

ATATTTCCAGATAATGTACCACACTATACAGATATACACAATTCAGATAAAGAAAGAATAACAATTGCATTTGATTTATT

AGCAGAAAATCCATACAAAGATAACTATTTAAAGTTAATATGAAAAATGTAAGATATCTTGTAATAGACAAGAAGGATGA

TGTCTATTTAAAGATAGAAGCAGAAGATTCTATTAGAAGAGAATTAGGTCAACACTTTACTTTTGAAGTACCTGGTTTTC

GTTTTATGCCTCAATTTCGTAATAGAGTATGGGACGGTAAGATAAGATTATTTTCATATGCAACTGGTCAAATATATGTT

GGGTTGTATCCATATATACTTAACTGGTGCAAAGAAAATGAAATAGAAGTTGTTGATGGATCAAAGATAGAAGATACTAA

AGTTGACGATAGTAAAGTAGAAAAGTTTATTAATGCTCTTAAAGTTCCTATGGACGTAAGAGATTATCAAAAAAAGGCAT

TTACATATGCTGTCAGAAAGAATAGATGTTTATTACTATCTCCAACTGCTAGTGGTAAATCACTAATTCTATATCTATTA

GTACGTTTCAATCTATTAAGACTACCTAAAAATAAGAAGATATTAATTATAGTACCTACCACATCACTAGTAGAACAACT

ATATAAAGATTTTAAAGACTATGGTTATGATAGTTTAAAAAATGTACATAGAATCTATGAAGGACATAGTAAGATAACAC

CTAAAAGAGTAGTCATATCTACTTGGCAATCAATCTATAATCTATCAAAGAACTATTTTAGCGACTATGGTATGATAATT

GGTGACGAAGCACATCTATTTAAAGCAATATCATTAACTAAAATACTAACGAAGTTAACTAATTGTAAATATAAAGTAGG

TTGTACAGGTACCCTAGATGATAGTAAGACACACAAACTAGTATTAGAAGGACTGTTTGGTGCAGTCAATAAAGTCACTA

CAACAACTGAACTCCAAGACAAAGAACAACTAGCTAAACTTAAAATTTTCTGTTTAGTATTACAATACAGTAAAGTACAA

AGAGAATTCTTAAAAAATAAAACATACCAAGAAGAAATGGATTTTTTAGTTAGAAATGTTAAAAGAAATAAATACATAAA

AAATCTGGTCACTAATTTGCACGGCAACACTTTATGCTTGTTTCAGTATGTAGAAAAGCACGGTAAGTTATTATTTGAAC

TAATAAAAGAGAAGGCAGGTGACCGACCTATATTCTATATCCACGGAGGAGTGGAAGCTGATGAACGAGAACAAGTCCGA

GCAATTACCGAGAAGTCTGATAATGCGATTATTGTCGCTTCTTATGGGACGTTCAGTACTGGTATTAATATCCGCAATCT

ACACAATATTGTTTTTAGTAGCCCTAGTAAATCTCGTATAAGAAATTTACAATCTATTGGAAGAGGATTGAGATTAAAAG

ATAACAAATCACACGCAACGTTATATGACATTGCTGATGACCTTTCCTATGGGGAAAAGGAAAATTACACTTTACAACAC

TTTAGAGAGCGTATAAATATATACAATAGTGAAGATTTTGACTATGAAATCCACAACATAGAACTGGAGAAAAATGGAAG

TAAAAGCTGACATAAGAATAATCAAACTAGTTAATGGTGATGATGTTGTTGCACATTTACCAGCTGGAGAGAAACAACTA

CCAGACAAATCTCCTTTGCTACGAATCAGCAAACCATTACAGATTAAGTATATTCCACAGATGACACCTATGGGAATAAG

AGATTATATTGCTCTTATTAAATGGGTTAATTATACACCAGATAAGGTAGTTACTATTCCGAAAGATAAGATAATGACAA

TAACTATGGCGTCTGGAGATATGTCAAAAAGTTATTTAAACCTGGCAAATGATTATGATAAATTAGACCAACCTAAAAAG

GGCAAGGGTGGGTTCTTTCAACGAGAACAATTGTCAGAAGAAGATAATGAAATGTTAAATGAAATCTTTAAAGATAAGGC

AACGAAAAGAACTCTCCACTAGGGTGCTTGGAGCGTTCTCCAACAGACTACATAGTCCATTATACACAAAATTTGGGAAA

AGTCAATGTTGGTTTCAGCATTGACAATATATAAAAAATGGTGTAATATGTAATTATGATGAATTCAACAAGAACAAAGA

AAAAACCAGAACATTACGTAAATAATAAAGAATTTTTTGCCGCTATGGTGGACTATAGAAAGTCTGTTAATAAAGCAAAA

AGAGAAAAACGAAATAAACCAGTTGTACCAGACTATGTTGGTGAGTGTTTTTTGAAAATAGCGAATCACTTATCTTATAG

ACCAAATTTTATAAATTATACCTATCGTGATGATATGATATCAGATGGTATAGAAAACTGTTTACAGTATCTTGACAATT

TTAATCCTAAAAAAACTAACAATCCCTTTGCATATTTCACACAAATTATCTACTATGCTTTTGTACGGAGAATACAGAAA

GAGAAGAAACAAACAACTATTAAACACCGTATGATTCAAAATGCAAACTATGATGATATGACTTTGCAACCTGGAGAAGA

TAGAGAGTTTAAAAATCAATTTACAGAATTTCTACGAAAGAATATACCAGCGGAAGAACCAGTTAAGAAAAAAAAGAAAC

CAGTTAAAAGTTTTTATAAAAGAAAAAAATAATTATGAAGTGTGATAAGATTTTGATAGTGGGTGGAGGTTCTGCTGGCT

GGATGACGGCTGCTACTTTAGTTAGAGCATTTCCCAACAAAGATATAACAGTAATAGAATCACCTAACGTTCCAACAATA

TCAGTTGGTGAAAGTACAATACAAAAAATTAGACAATGGACAAAATTTTTAGACATTAATGATAAAGAATTTTTAGCAGA

AACAGATGGCACTCTTAAATTTAGTATTAAGTTTACAGACTTTAATGGAAAAGATGAGGCGGCATTTCACTATCCTTTTG

GTTCAGTTGACATACAAGGAACTAAATTAAATTATAATGATTGGTGGATGAAAAAGGCATTTAATCCAGAAACACCTGTT

TCAGATTATGCTGATAGTTTTGCTCCTAATATGGCATTAATTAATCAACGTAAGGGTACTTATAATTTTTACGATTTTAA

AGTTGACCGTGATTCTGCTTATCAGTTTGACGCAATTAAATTTGGTATATGGTTGAAGGTTAATTATTGTATCCCTAGAG

GTGTCAAATATATTCAAGAAGATATAAAAGATATTAAACAAGATGAAAATGGTATTGTTTCTTTAAACAAACATACAGCA

GATTTATATGTTGATTGTACAGGTTTTAAATCAATGCTTTTAGGTGGTGCATTAAAAGAATCTTTTGAACCTTTACAAAA

TCTACCAAATAATAAAGCGTGGGTAACAAAGATTCCTTATGTTGATAAAGAAAAAGAAATAGAAGCGTTTACCAATTGCA

CAGCAATAGAGAACGGTTGGGTATGGAATATACCATTGTGGAGTAGAGTTGGTACAGGTTATGTTTATTCAGATAAATTT

GTAGATGATGAAACTGCTTTAAAAGAATTTAAAAATCATTTAGCAGGTGTACGACCTGGGTTTGGTAATGAAGAACACGA

ATTTAGAAATATTAAAATGAGAACTGGTACACACGAAAGATTATTTGTAAAAAATGTTGTTGCTATAGGACTATCTGCTG

GATTTATTGAACCATTAGAAAGTAATGGTTTGTTTTCAGTACACGAATTTTTAATGATGTTGGTAAGAAATTTAAGAAGA

GGTGAAATTACACAATGGGATAAAGACAACTTCACATTTTCTTGTAAATCTATCTATTATGGATTTGCTGAATTTGTTGG

GTTGCATTATGCATTATCAACAAGAAATGATACACCATATTGGAAAGCAAATAACGAAACAGTATGGGAAGAAAGTTTAT

CTAATTTTAAACCTAAAATTATGCTTGGATATTTACAAGCTGCTTTACAAAGAAATCTTAATTCGGAGTTTCCTGTTGAT

CCTGCTCTTACGCCAGGTAAATATGGATTACATTATATAGCGGCTGGTTTCAATTGGGCACCAACTGATTTACCTAATAT

GATAAATCTAACACATAAGAGTGAGAAATATATAAAAGAATTTATGAAACCATACATAAAGAAACTAGATGAACGTAAAG

AAAAATGGAAAAAGGAAGTTGAAAAGTTTCCAAGTTATTACCACTTTCTATTGGAGAATTATTACAAGTGAAGATTGCCC

TACTAAACGATACACACTTTGGTTGTCGGAATGATAGTCCTCATTTTGTACAGTATCAAGAACGGTTTTATAATGAAGTG

TTTTTTCCATATATAAAAGAACACAACATCACAACGTTAGTACATTTAGGTGACGTAGTTGATAGACGTAAATTTATAAA

CTATAAGACGGCTAATTTCTTTCGCCAAAAGTTTATGAAAAGACTTTGGAAAGAAAAAGTAGATACACATATTATATTAG

GTAATCACGATACTTATTATAAGAATACAAATAATGTAAATGCAATTACTGAATTATGTACAACTTATGATGGTCAAAAT

GAACCTTGGATATATGCAAAACCAAAAGAAGTAACCCTAGATGGTTTAAAGATTTTGTTTATGCCTTGGATATGTGATGA

TACGTATGAAGATTCTTTATATGCTATTGAAAATTCAAATGCGGAAATATGTATGGGGCATTTAGAAATTAAAGGGTTTG

AAATGAATAGAGGATTTATGAACGAACAAGGGTTAGATAAATCAATGTTTCATAGATTTGAAAAAGTTATATCAGGACAC

TTTCATAAGAAATCAGATGATGGTCGGATATATTATTTAGGTACACAATATGAAATTACTTGGAACGATTATAAAGACCC

TAAAGGGTTTCATATATTTGATACAGACACTAGAGAGTTAACTAGAATACCTAACCCAATAAGAATCTTTAAAAAGATAG

TGTATGATGATACACAACACGATTATAATACGTTAAACATATCAAGATTTGATAACAGTTATATTAAACTGTTTATATCT

CAAAAAACAGATGAAGATATGTATGATAAGTTTATCAATAGGTTGTATAGTACATTAAATATACACGAACTAAATATATT

TGAGGATACTAGTGATGTAACTGCTAGTGTAAAAGAAGATTTAATAGAACAAGGTGAAGATACACTTACATTTTTGGGCA

AGTATATAGACCAACTTGATACAGACCTTGACAAAAGTAAATTGAAAGATTATACTAAAGAACTATATAGTGAGGTAAAT

CAATGAGTATAAAGAGTAGAGAAAAAGAAAAAGAACAAAAGGCAAAAGAGTTTGAAAGATTAAAAGAAGAAAAATCAAGG

GAACACAATTGGGTAATTGGCGAACCAGAGGGTGAAGAAGTAAAACAAAAAAGAGATGAAGAAACAGAACAAAAAGAAAA

AAAATTAAGGGAAGAATTTCAACAAAAAGTTGAGGCACAGATGAATAATCCTGATAATCAAGAACAAGCACAAGTATTAG

GACATTTAAATTTTGGACCATATTGTGCTTATATGAAAATAGATGATGATTTTTTTAAAGGACTTTTAAAAAGAGGTAAA

GACAGTACACCAGGTTCAAGTAATAAAAAATTAGCAGGTCTATTAGGTGACCAAAGAACTTATAGTGATGAAGATAAAAA

TTGGTTTATACAAAAATTTCAACCATATATGGACTCATATGTTGAAGGTGCTTGTGCATTTATTGGACAACCATATGATG

AAAGTCAGTTTTCAAAATCATTTACATTAATGGATTTGTGGATTAATTTTATGAAAGAAACCGAAATGAATCCTAGCCAT

ACACACGGTGGACAATTCACTTGGGTTATGTATTTAAAAGTACCAGACCTAGAAGCAGAAAGAAAAGCATTTCAAGGAAC

TGGTCTTGGTCCAGGAGTTATAGGATTTCATTATGGGGAAAACTCTCAACCAAAATGGGCAGAACACACATACAAATATT

TACCTGAAGCAGGTTATATGTGGATATTTCCTGCTCAATTAAGACACGAAGTATTTCCTTATAGAACACCAGGAGAAAGA

ATAAGTGTATCAGGTAATTGTTTTATGAATCCTCCAAACCAAAAATCAAAAATATTATCTCCAGGGTTCCACCCTTATGT

TGGATTAGGAAAGAATTTGAAACCGCAATAAAACATTTAAGTTTTATATTATGATAAAGTTTAAGCGAATATCTTATAAG

AATTTTTTATCAACAGGCAATGTTCCAATTGTTATTGATTTAAATAAATCTCAACTAACATTAGTTATAGGTTCTAATGG

AAGTGGTAAATCTACTTTGTTAGACGCATTATGTTTTGCTTTATTTAATAGACCATTTCGTATTATTAAAAAAGACCAGA

TGGTGAACACTATTAACAATGGTGGTTGTGAGGTTGAATTAGAATTTAATGTAGGACCTAAAGAGTACGTTGTTAAGAGA

GGAGTTAAACCAAACTTCTTTGAAATACATTGTGATGGACAATTAATGAGTCAAGACGCAAGCGCAATTGATTATCAAAA

GTATCTTGAAGCAAATATTATGAGGTGTAACTATAGGTCATTTTGTCAAGTAGTATTATTAGGGTCTTCTTCTTATATGC

CATTTATGAAAATGAGAGCAAGTTTTAGAAGAGAGGTCGTTGAAGAGATTTTAGACATAAGAGCATTTAGTAGAATGGAT

ACTATATTGAGTGGTCAACAAAGAGATTTACAGAACAAAATAACAGAAGTAAGACATCAATGTGAACTTATAGAAACCAA

GTATCAGACTGAAGCAAAGTACTTGGACACCCTTCTCCACAAGGATATAGACGTCCAAACGCATAGAAATAGGGTGGTTG

AGCAGAACACCAAGGATAGACTAGAATATGAGAGTAAGATGGTTACGATTAATAAAGAGATAGATTCAGCTAAAGAGAGT

GTAAAAGATAGAGTTGATGTTGATAACAAGAATACAAAATTAAATAAAATAGAAGCGAAGATTGAACAGAATTTAGAAAG

ACATAAGAACTCTTTAAAATTTTTTGAAGAAAATGATGTATGTCCAACCTGTACACAACCATTATCTCCAGAATTTAAAC

ATCAAAAATGTGATGAAGAGAAGAGTAAGATTAATACATTACAAGATGGTATGGAAAAGTTATTAAAAGAACTTGTAAGT

ATGGGTGAAAAAATAACTGAATACGATAAAGTAGCAGATAAGATTTATAGTTTAAATGTTGATTTATCAAAAGTAGAAAC

GTCATTAGATAGTTTAAAAACTCATAGTGATAGCATAGAAGAAGATTTAAAAGTATTTAAAAATAAGGATGAAGATATAG

CAAATATTAGAAAACAATTAAATGAAATGAAAGACCAATTAAGACATTGTAAAATAGAATTAGATAAGATTGTTGAAGAT

AAAAAGTATCAAGATGTATTAAGACAAGTATTAAATGACAAAGGTGCCAAGGCACAAATCATTAAGAAGTACATACCTAT

AATGAACCAATTGATTAATAAATATTTACAAGCAATGGAATTTTATGTATCGTTTCATTTAGATGAAGAGTTTAATGAAA

CAGTAAAGAGTAGATTTAGAGATACATTTAATTATAATAACTTTAGTGAAGGTGAAAAGATGAGAATAGATTTAGCATTG

TTATTTACTTGGAGAGATATAGCTAAACTTAAAAACTCTACTAATACAAATCTATTAATACTAGATGAGATATTTGATTC

AAGTTTAGACCTTGCTGGAACAGATGATTTCTTTAAAATAGTACAGAAATTATCAAACGAAAATGTCTTTATTATTTCAC

ATAAAGGTGATATACTATTTGATAAATTTACAAATATAATTAAGTACAAAAAAGACCAAAACTTTTCAGTACTAGATAGG

ATATAATGCCAGAAGAGAAAAAGAAAGAAAGATTTTTACAGTTGATACCACCTAGTGACCCTAGAGTTAAATCAGCAATA

GCACCTTTTACAGATGATATGTTAAAGGAACACGATTTAAAAGATAGAAAAGAACTATCAGATTTAATGTTTAAAACAAT

GTTAAGATATGGTGGTATAGGTTTATCTGCTAATCAAGTAGGGTTACCATTTAATATGTTTGTATTAGGTGACCATTTAA

ATTTAGAAAATGGTTTAAAAATGACTTGTTTTAATCCTATGATAGTTAGTACAAGTGAGGAAACAGTATTAATGAAAGAA

GGTTGTTTAACATATCCTTTTTTATTTTTATCAATAGTAAGACCTAGAAAATGTGTAGCAAAATATACAGATGAAAATGG

TGATTTAAAAGAAGGACAATTAGATGGTATGATAAGTAGAATATTCCAACACGAATATGAACATATGTTAGGTAGAACTT

TTACAGAACACGCAAGTAAAATGAAGTTGGAAATGGCAGAAAAAAAGGCAAAGAAGATGTGGTTGGCGTACCAAAAAAGA

AGAAATGAAAAAACTAACAAATAGAACTGATACAAAAAATGCATTAGAAGCATTTGGTGTAGAGATAATGGGTGAAGACC

TTATTACTAGTAAGGGTAACCTAAAGGGTGTAGTTAAAGACGAAGAAGAATTACAATCTAATATTGATAAAGTTTATGAG

TATTGGATGAAAAGAGGATTTCCTTATTATGCTACAGATAAACAGTATAGAGAATCACAATTTAAAACATTACAATCAAC

AGATTTTAAGGGGTTATTAACGCAAGAGAAAGTTATTAAACCAAATCAAACAGGTCTATCTTTAGCGTGGTCATATATGC

CACATAGTTTTGGTATTCGTTGTGGTAAAATGAGAACTCCTATGGAGATATACACTAACGAAGAGCATTTTAAAAAGGGT

ATACGTAAGTTATTAACAGGTAGTTTCTTTGGTCAAGTATCAGTAGATGATTTAATGCCACTTGCAAGTAATTTATATGG

TGAAATAACTGAAATATCTCCTGCTTCAAAACATAAATCAGAAAGTGTTATGAGGTCTTTATTAAGAAGATATACAGGAA

CTCAATGTGTATCTAATTTTAGACCTACAGCAGCGGCGTGTTTATATTCACACTTTGCCCACCCAGGTGCTATGGTATGG

GATATGTCAATGGGATATGGTGGTCGTATATTAGGTGCAATTATATCAGATATTAACTATATAGGTACTGATCCAGCTGA

ATTAACATTTAAAGGATTAAAAGAAATTAAGAAAGATTTTGGTAGAGAGAATAGACATTACTTTTTAAACAAGTGTGGTA

GTGAAACCTTTGTTCCAAAAGAAAACACTTTAGATTTTGCATTTACAAGTCCACCTTATTTCAATTGGGAACAATATGGT

GATGAAGCAGAACAATCATTTAATCAATATAGTGGTAATGAAGAGTGGAACAATGGCTTTTTAAGAACAACAATACAAAA

TGCATATATAGGATTAAAAAAAGGTAAGTATATGGGGTTAAATGTAGCGAACATTAAATCACATAAAACCTTTGAAGATG

ATACAGTACGAATCGCTGTAGAAGAAGGATTTGAACATACGGATACGTACAAATTACAGTTATCCTCGCAAGAATCAGGT

GCAAAATACGAGCCAATTTTCATATTTAAGAAATAGGATCGTCTGGAAGCCGCATAAAATAAGGGGAAATGAACACTTGA

CTTTCCCGATTTTTTCCTGTATGATATATACATACTATGAAAAAAGGCACTACAACAATAAATTTAGATACAAAAAGTCA

GTTAGCTAAATTACTTGCAACAGAAAATATAATCATACAACACAACAACGTTTCAACAGCGTCTTTTAATACAAAGACTC

GTGTGTTAACTCTCCCTATATTTAAAGAATCACACGGTGATGTTTATGATATGTTAATTGCTCACGAATGTGCCCACGCA

TTATTTACTCCACAAAACGGTTGGAAAAAAATTCAAGATGATGATGAGTTAAGAACTTACGTTAATGTTTTAGAAGATAC

TAGAATAGATAAAAAAATTCAAAAGAAATACCCAGGAGTTGTTAGAAATTATATCAACGGTTTTGATATATTAGAAAAAC

AAAACTTCTTTGGTATGAAAGATAAAGATTTAAATAAAGAACTTATGTTAATTGATAAGATTAATTTAAGAAGTAAATCA

AGTAATAGATTACCATTTATATTTGATGATAAAGCAAACAATTGGTTAGATAAAGTTGATTCAATTAAATCATTTACAGA

CGTTGTTAGAGTTGCTAAAGATATGTTAAATTGGCAGAAAAAACAAGTTGAACAAATGAAGAAATTACCAGATTTTGATG

ACCACCCATTAATTAAAAATTATGAATTATCAGATGAAGACGCAACTGATCCACAAGATAAAAAAGAACAAGACCAAAAT

TCTGAAAGTGCTGATAATCCAGATGTACAAGATGAAAAAAATGATAAAAGAGATTCAGAAGAAAAAGCAACAGACAAAGA

TTTAAAAGATAAAGAAAAAGAAAACGCTAAAACAGCAACACAACACGCCAAAGGTGCTGACGGAGACCCTAAACCTAAAA

AATTAAAAGCTATTACAAATGATTTCTTTGAACAGAAAAAAGAATCGTTGTTAGATAAAAAGACTTCTTATGTATACGGA

ACATTACCAAAACCAAATCTTAACCAATGTTTAGTATCATATAAAACTTTTTTAAATGATTTCAGAAAACATATAAGTGA

CCAAGTACAATCTAATCCAGAAAGTACACTTGAATATAAAAGATGGATTTTAGATAAGTTTAAAAAATTTAGAACTGAAA

ACAAGAAGACAGTTATGTATCTTGTTAAAGAATTTGAAATGAAAAAAGCAGCGTCTGCTTATAAAAGAGCAAATACTGAT

AAAACAGGAGTTATTGATCCTTTAAAATTAAGAAATTACAAATTTAGTGAAGACATATTTAAAAGAATGACTATTATACC

TGACGGTAAGAACCACGGTATGATAATGTTATTAGATTGGTCAGGAAGTATGAGTGATTGTATTGCTGATACTGTTGCTC

AATTAATTAACCTAGTAGAATTTACAAGAAAAGTTAATATACCATTTGAAGTATACTTTTTTACAAGTGAAAGAGATTCA

GATGAAAGAGATAAACAATATTGGAATTATAAGTACGGTGATTTTTGTTTTGATGAATTTAAATTAGTGAATTGTTTAAG

TCATAGAATGAAAAAAAATGAATTTGAAGAAGCACTATTATATATGTATCATATGGCAAAAGATTATGACCAAAGATGGT

CAAGAAATTGGAGTGATCCAGAATATCCAAAAGGAAGTAACTATCATATACCAGACAAATATTATTTAGGAAATACACCT

TTAAATGAAGCATTGATAGTATGTAATAATATAATACCAGAATTTCAAAAGAAATATAAAGTTGAAAAACTTACTTTTAT

TACTTTAACTGACGGAGGCGGAAACAGTTTCAGACACAATCAGATAATACCTATACCAGATAAACCTACTAGAGCAATTG

ATGAGTATGAAATTAAAGACGCAAAAGCAAAGAAACAAAATTATATAAAAAGAAGTATTGATTATGAATCTAAAGTAGTT

ATTACACATAAGAATAAAAAGATAATTTTAACAGACGGTTGGTACGGTAGTGCTATGACCGATACATTATTAAGTATGAT

AAAATCAGACCACAATCCTACAATAGTCGGTTTCTATATTATAAAAAGAATTAGACGTTGGGAACTAGATAGATTTATCG

GAAGTGATTATAAAGATTATGAACATAAAGAAAAATTAAGACTTAAAATACAAAAAAGTTTTAGAACTGATAACGCTGCT

ATAGTATACCAAAGTGGATATGATAAGTATTTCTTACTAAACGGCAAGAAATTGAAAGTACAGAATTTTAACCTACAAGA

CGCAACAGTTAAAAAAGGAACTGGTGCTGAACTAAAAAGAATCTTCGGTAAGAGTATGAAGAATCGACTAGTTTCAAGAG

TAGTTTTAAACAAATTTATAGCGGAGGTCGCATAAACAATGAAAAAAACATTGAAAAATAAGGGTATTTTGTATATTGAC

TTTTCTAAAAAAGTCCTGTATAATATACTTATAATATAATGATGAAAGGACGTGAAAACACTATGTTAAATCAGAAACAA

ATAGACTTTGTTAAACACGCTAAGAAGTTGTTTCCAAACAAAGTTGAATTAACACTTGCTGATTTAGTACTTGCCAATAA

AGAATTTGGACATAAGTACGAACCGCAATGGTTAACAAAAAATAAGAACCTAAAAGTTGATAGGGGATTATTTAGATTAC

CAAACATAGATGATAAAGTTGAAGAAACAAAAGTTTCTAAAACTGAAACCGTTAAAGAGAATAAAGTAAGTGAAGCAGCT

TATATAGTTTCTTCTTTGACTGGCGATATTGTACCTAAAAAGGATTCAGTATTCGTATCATTTGGTAGTTATCCTGACTT

AAAATCAATCGTCAAATCCAGAATGTTTTATCCTGTTTTTATTACAGGACTTTCAGGAAACGGTAAGACTATGGGAGTTA

CCCAAGCTTGTGCCGAAAACAGACGTGAAATGATTAGAGTCAATGTAACAATAGAAACAGACGAGGATGATTTGCTCGGC

GGTTATAGATTAAGAGAAGGACAAACCGTATGGCAAAATGGACCTGTTATAGAAGCAATGGAAAGAGGCGCAATTCTTTT

ACTTGATGAGATAGACCTTGCAAGTAATAAGATAATGTGTTTACAACCAATTCTTGAAGGTTCTGGAATCTTTGTTAAGA

AGATTAATAAATTCGTGAAACCTGCCGACGGATTCAACGTGATTGCTACTGCTAATACTAAAGGACAAGGTAGTGAAGAC

GGAAAATTTATCGGAACTAACGTGCTTAACGAAGCATTTTTAGAAAGATTTCCGATTACATTTGAACAGAAATATCCAAG

TGTTAAGATTGAAGAAAAAATCTTAATTAAAACTCTTGAAAGAAGTGGAAAAAAAGATAAAGACTTCTGTAAAAAGTTAG

TCACTTGGGCAGACGTTATAAGAAAAACTTTCTTTGACGGAGGCGTAGATGAGATTATCTCAACAAGAAGATTAGTCCAC

ATAGTTCAAGCATTTACTATCTTTAAAGATAAGATAAAAGCTATTGAAGTATGTACTAATAGATTTGATGAAGATACAAA

GAATTCATTTGTAGAGTTATATTCTAAAGTTGACGGAGGTGCTACAGCAGAAACAATTGCTGAAGACCAAAGAAAACAAG

AAGTAGCTGACCAAGTGAAAGAGGAAGAGAGTGACTCAAAAGATGACGCTGCTGAATCAGACAATGATACATCAGCTCAT

ATTTAAAAACTCTCAATCATAGTGTAAGTCCTGAAGCGGAGGTAGTGCTCCGCTTCATAAACTACACTTGAAAGGAACTA

TGAACGATTTTTATAGCGAATATATGAAGAAACAAAACAAAAGAAATAAATTAGAAAGATTTTTAGATAGACATAACCAT

ACAATGGAATTAATTAGAACTATACTTCCAGTTATTTTGTTAGTTATGCAAACAATAATTTTATTAAAGATACTTTAATG

GCACATAGTAAAAAAGAAGTTTTGAAGATATTAGAGAAGAATAAGATTAGTAATTTTACTACACCTGAAGACCAAATAAT

GATGAAATTAAAAACAGCGAAAGTTGAAACATTAGAATCTCAAAAAAAAGAATTAGAAGATAGTTTGGTTCAATCCAGAC

ACCAAGAATATATTAGAATAGAAATGGATAAGATTAGAAAAGAAGGAAAAATGAAAGAGGAGTGTACAACTGATACACAT

AAGATTATTAATGAAGCGGAGAGGAGGGTAAAAGAACGTGGCAATAACAGTTGAAGTAAGACACGGTAATGTAGAGAAAG

CTATGAGAGTGCTTAAGAAGAAAGTACTTAAAGCAGGTATACTAAAAGACTATAGATTAAAGCAGTACTATAGAAAACCA

TCAGAAATTAAAAGAGAAAAGAAAAAAGAAGGTATCAAGAACTGGAAAAAGAAGTTGAAATTGATACAGAATAGATTATA

GAATTACACGCCTGTGCTTGAATAAATATATTATACCAGGCAGTTCATAAGTCCTGGGGCGTGGAAGGGTGCCAACACCT

GCAGATTTAATATCTGAAAGTTGGTAGTAGTTTGAGGTCTACTATAACAAAACCTCAATGAATTACGAGTTTTGTGGGAG

TTTGGGGAGACTCTAAAACCCATAGAAGGTCCAAAGGTTTCAATACCAATAGCGGACACTACGATAGATATTTTGGTGGA

TTCATACTATTAAAAATAAATCCATCGGCGCTACTTGTATATTGTAGGAAACTACATATATAAGTAGTAGTGAGTTGCCA

TTAAGGGACTCATAAACGATAACTTTGCTTAATAAAAGGAGGTTTTTATGACCAATAAAGCATTATCTATTTTTAACAGG

TTAAGACCAGTATCGGTTGGATTCGATTCAATCTTTGACCATTTCGGTTCAATGTTTGATGACGATTTCATCAACGATAT

ACAACCTAGTTACCCACCATACAATATAGTTAAGTCAGGTAAGAATACTTACGATATTGAAGTTGCATTAGCTGGATTCA

ACAAGAAAGACATTACTGTCAATGTTGAAAACGGTATGCTAACTATTGAAACCAAGAAGGAAGATGAATCTTCTGACAAG

GATGAAGATGGTGAAGTATTACATAAAGGTATCTCTAAAAGATACTTTAAAAGGTCTTTCACAATCGCTAATGATGTAAA

AGTCAAAGGTGCTGAACTGAAAGATGGACTGTTAAAAGTTTCTATGGAGAAGATTATTCCAGAAGAAAAGAAACTAAAAA

CAATAGACGTTAAATAATAACGTAATATAGATAGTGGCGGGTAAAACCGCCGCTATTTTTTTTGGGCCTAACCAACATTG

ACTTATAAATACTATTGTTATATAATAAACTAAATGAGGAAATTATGAATAAAATATTAGTGATTTTAATGAGTTTGATT

CTCTTAAGCGCTTGTTCAAGCATAAAAAATCCAAGTATAGCATTCGGTAAAAAGTGCGTAGCAAAAGGTGACCAGGTTCA

CTATTCTTACGTATGGATATTTGATGGCAATGCTGGGTTACAAGCAGATGAGATTACTTGCGAATTAATTGATAAGAAGG

AAAAGAAATGAAACTTAAAGATATAAAAAATAAGATATCAGGAACAGCAGTAGCAGCAGTTATCGCTATAGTTGCGTTAT

TGATTATTACTGTAGACTTAAAAAATAAAAAAGATAAAGCACTAGCTTTATTAGCAAACGCTGAGGTAGGTATTTCTACT

GTAGAAGCAGAATACTTGGATAGTATTGCATTATTGGAAGGTACAGTACAAGCATATGAACTTGAATTAGGTTCAATTAA

AGTTGTATTAACAGAACAAAATAGTGAATTAGCGACAATAGAGTCGGAGTTAAATACAGCAGAAGCAGTAGCAACTCAAT

TAGCAGACCAATTGGTTACAGCAAATGCTACTATTATAGACTTAACAGAAAATCCTAACTGTCCTGTTCAGTAACCAACA

TTGACTTTATGTGAGGTTGGTGATATATTACAGTATGATTAAATTATGAAGGAGTTGATATGAATCTATCAAACAGTACA

GTTGCAATTTTAAAAAATTTTTCTGAAATTAATAAGAACATTTTAGTTAAACCAGGAAAGCAACTACAAACTATTTCTAC

TTTAAAGAATATTCTTGCTGAAGCAGACATAGATAATAAATTTGAGCAAGAATTTGCGATATATGATTTACCAGAATTTT

TAAGAGCAGTTGAATTATTTTCTAAATCAGATATCAAATTTAATGGTACTAACAAATTGGTTATATCAGACGCCAATTCA

AGACAATCTGTTAAGTATTTCTTTGCAGATAAATCAGTAATTGTAGCACCAACTAAATCAATTAATATGCCTGATAAGTA

TGTAACTTTTACATTAAAGGGTAAATGTTTTAATGACTTATTGAAAGGTATAGTTACATTGAACTTACCAGACATTGCAG

TAAAAGGTGATGGTAAAAACATTACAATGATTGCAACTGATAAAAAGAATAAATCATCTAACGATTATTCTGCTGTAGTA

GGTACAACTGATAAAACTTTTGTAGCATATTTCAAAGCAGAAAATTTAAAAATCATACCAGATGATTATGATATTGCAAT

TTCTAAACAAAGAATAAGTCATTTTGTTAATAGAAATAAACCAGTACAATATTGGATAGCATTAGAACCAGATAGTGAGT

TTTAATTATGTCAGTAAAAGACTGGACGTGTACTTTTATTTGTAAACATACAGCAAGAGGTTCACATAGGTGGGCATTTT

GGCTGGAAGGTATTATAATAGGAGTATTGATAGGGTTGATTATATGAAAGTGAATATATTATGGCAGAAAATTTATGGGT

TGAGAAGTACAGACCAAGAAAAATTGAAGATTGTATTTTAACCAATGAACTAAAAGAAACTTTTAAACAGTTTATAAATC

AAAAAGAACTCCCAAACCTATTACTATCAGGTACAGCAGGTACAGGTAAGACTACTGTAGCACGTGCTTTATGTGAAGAG

TTAAGTGTTGATTACATTATCATTAATGGATCAGACGAAGGTAGACAGATAGATACGTTAAGAAATAAGATTAAAAACTT

TGCGTCAACTGTATCTTTAACTGAAACAGCAAGTCATAAAGTTGTTATACTTGATGAGGCGGACTATATGAATCCAGAGT

CCGTTCAACCTGCATTAAGAAATTTCATAGAAACATTTTATAAGAATTGTAGATTTATCTTTACTTGTAATTATAAGAAT

AAGATATTACCAGCATTGCATAGTAGATGTACTGTTATTGATTTTGCGATTACTAATGGTGATAAAATTAAAATAATGAC

ACAACTTATGAATAGGTTGTGTGGTATCTTAACAGAAGAAGTGATAGAGTTTGATAAAAAAGTAATTGCAGAATTAATAC

AGAAATACTTTCCAGATTTTAGAAGAACTATTAATGAACTTCAACGATATTCAGTAAGAGGTAAGATTGATAGTGGTATA

TTATTCAGTTTAACTGAAGCAGATACTAAACAACTTATCGCAATTTTAAAAGAAAAAAGATTTAATGATATGAGAAAATG

GGTTATACAAAACCTAGATAAAGAACCATCAGCATTATTTTCAAAGGTATATGAAATACTATACAAACATTTACAACCAC

AATCTATACCACAGGCAGTTTTAGTTATTGCTGGGTATCAATACAAGGCAGCTTTTGTAGCAGACCAAGAGATTAATATG

GTCGCTTGTTTAACAGAAGTAATGGCAAACTGTAAGTTTAAATAATGAAAGCAAATAATAGAAAAGAATACTATAAACAA

TACCATTTAAAAAATAAAGAACGTAAAAAAGAATACGATAAAAGATGGTATTTAAAAAATAAAGAACGTAAAAAAGAATA

CTATAAAAGATACCGTTTAAAATATAGAGAATGGTATAAAAAATATTCAAGAGAATACCGTTTAAAAAATAAAGAAAAAA

TAAAAAAACATAAAAAAGAATACGATAGTAGACCCGAAGTAAAAAAACATAAAAAAGAATGGAAATTAAAAAATAAAGAC

CGAATAGATGAATGGAGAAGAGGATGGGACAAGAATAAACGCCTAACGGATCCCAATTATAGAATAGTTGATTCTTTACG

AAGCAGATTGTATAAACTTTTAAAAGGTAAAGATAAGTCAGCTTCAACTATGGAATTACTTGGTTGTACAATTGATGAAT

TATGGACTCATTTAGAATCTAAATTTGAACCTTGGATGACAAAAGAGAATTATGGTAGAGGAGGTTGGGATGTAGACCAT

ATTGAAGCGTGTTCTAAATTTAATTTAACCGATCCAGAACAGCAACGCATATGTTTTCATTGGAGTAATTTACAACCTAT

GGAGCATATTGCTAATATAAAAAAAGGGGCAAGATGAAATGATGGATAGAAAAGCAGTACTCGGTCAAATAGGTGAAAAG

ATAGTAAGTAATTACTATTCTAGGGCAGGTAAAGTAGTAGAACACTCTATTGATCCTTTTGATAGTCATAAAGATTTATT

AGTAGAAGGTGAACGAGTTGAAGTTAAAACACAAACTCGCTATTATACTAAAGATTGTTTTACTCTTAAAACGAATCAAC

TTAAAAAATGTATGGATGGGTTTTATATTGTAGAGTGTCCTACATCAGCAAGTAATGTATCTTCACTATATAAAGTAGAC

AAAGGTTTTAGATATACCACAGGTCAGATGAATAATGGAGATATTAGATACGAAATAAAAAGATTGCAACCAGCTATCAC

AAAATTAACAGACATTGAAGGTAAAGAGAAAATGTTATTGAGAAGATACAGCACAAATTATGTACGAACTAAAAGATTAT

CTTAAAGCTATTAATGAATCAAAAGAGAATTTATTAGACACACCAGACCCGACTTGGGAAAAGAAGTACCCACCCTTTGT

AATTAACCGTTGTCTATCTATGTTCTATGATACCATAATGCATAGTAATGAAATGAACGGACTACACTTTCTATCTAAAC

GTATGCAATTTCACTATTTAATAAATAGTATACGAAAGAAAAGGCGATTTGGAGGTAGGTGGTTATCAAAAACCAAGTTG

AAGAACCTAGACATTGTAAAAAAGTATTATGGATATAGCAATACAAAAGCAAAGGAAGTACTCAACATACTTACAGATGA

CCAAATTGAAAGACTTAAATTAAACCTTATACAAGGTGGGAGAAAGTTTAAATGAGTGAGGATATTATTAGTTGGTCACA

AGGCGATATGCTTGAGGTGACCATTAAACAACCTGATGATTTTTTAAAAGTCAGGGAAACGTTAACAAGAATAGGTGTGG

CGAGTCGTAAAGATAAAACATTATATCAATCTTGTCATATATTACACAAGCAAGGCAAATATTATATAGTCCATTTCAAG

GAATTATTTGCATTAGATGGTAAAAAATCTACATTAACTCAAAACGATATTCAAAGAAGAAATACCATATCTTTATTATT

ACAAGATTGGAACTTAATTGATATAGTTAAAAAGGATATAACAGAAGATAAAGCACCATTGAGTCAGATAAAAGTATTAC

CATTTAAAGAAAAGAAAGAGTGGACGTTATCTGCTAAGTACAACATTGGGAAGAAAGTGGACGACAAGAAAAAAGAATTT

AAAACAACACCAACTACGAGTCCAATGAGTGATGAATAAATGCAGATACCAAAATTCAAAGATTACATAACAGAAGCAAA

AACTTCTGGATCATATAGATTAATCATTATATCAGATGAACCTGAAGATGATTTAAATTTCCATACAGCAAAGAACTTAA

TGAAACAAGCAGATAAGCTTGGTCATAAGTCATACATCTATAGAAATACTGGTGGGTATGTAACCGTTGAAGATGATGGA

GAAATGTATTTCCATAATCAAGATGACAAAAAAGGATTTAGAGTATCAGCAAAAGATACAGTTGCTATTGTAAGAGGTTC

AGTAGTACGTAGAGATAGTTGGATGGACTTGGTATCAAGATTAGAAAAACACCAAGTGTGTGTAATTAACAGTAGACAAT

GTATTAGTATGTGTGCTGACAAATATAGAACTTCATTAAGATTAGCAGACTATGGTATTAAACAACCTAAATCAGTATTG

GTAACTGATCCAGAAAATTCAATACAATCATTTGAAAGTTTGGAAGAAAAGTTTCCTGTTATCTTAAAGACATTAAGAGG

TTCAAAAGGTGTTGGTGTCTTGTTTATTGAATCAGAAAAATCATTAGATTCAATAGTACAATTACTTAATAAACAAGATG

AAGATTCTGATATACTATTGCAACAATATATAAAAACTAAATGGGATGCTAGAGTATTAGTATTGCAAGGTAAAGTATTT

GCTGCTATGAAAAGAAAAGTTGTACCAGGAGATTTCAGAAGTAATGTATCAAGAGGTGCAGAAGTAGAAGAATTAACACT

AACAAAAATAGAAACAGAAGAAAGTTTAAAAGCGGCGAAGGCAGTAGATGGTCAATGGGTAGCAGTAGACTTTATTCCTT

CGGAAGATAGAGTAAAAGAATCACCATTTGTTATTGAAGTTAACTCTTCACCAGGTACAGAAGGTATAGAAGAGGCAACA

CATAGAAATTTAAGTAAAGAAATAGTACAATATTTTGAAGATAGAGATAACTGGAAGAAAGTACCTACCGAGTGTGGATA

TAAAGAAGTTGTCCATATACATCCATTTGGACGTATAGTAGGTAAGTTTGATACAGGTAACTCTGGTACGTCTGTTATAC

ACGCTGATAAAATAAAAAAAGGCGGTGGTAAAGTTACTTGGTCATTAGAAGGCAAGACACTTACAAACGATATAGTGCGT

AAGCAAACAATTGAAGTAGGCGGATTAAGAGATTATAAAGAAGAAAGATTAGTAATAAAACTTGATGTAGAATTTGCAGG

TGGATATTACAAAGAAGTAGAATTTACCCTAGATGATAGGGATGAAAAATCTAAAATATTATTTGATAGAGAAACAATGA

ATCGTTTTAACGTAATGGTTAATCCGAATCGTAAATATATAATAACTACAAAGTATAGTTTAGATGACAAGAAAGGAGAA

GATAAAAAATAAGATGAGTATAAACGGAAAAGTAAAATGGTTTAACCCAACAAAAGGTTATGGTTTTATTGCTAGAGAAG

ACAATGAGAAGGATGTTTTTGTACATTCCTCAGCGGCTAAAGCAGCAAACCTGGAACTTAATGAAGGCGATGAACTAACT

TTTGAAATTACTGAAACACAAAAAGGTAATTCAGCAGTAAATTTACAAAGACAATAAAGAAGGGACTACAATGGTTGAAA

TAACCAGAAGTAAAGAAGAGATAGCAAAGGACATTAAATTTATCTTGGAAGATAAAGTTGCTCCTGCTGTGGCTCAGCAC

GGTGGGTTTATAAATTATTTAGACTTTGATATGGATTTAGGTGTTGCAAAATTAGAACTAGCAGGTAGTTGTTCAGGATG

TGCAATGTCTAAACAAACATTACATCAAGGTGTGGAAGATATGCTTAAGCATTATGTTCCTGAAGTCCAAGCCATTGTAG

GAGAAGACGACCAAAAAGCAGAAGAACGAGGTTATTCACCGTTCATACCTAGAACTAACATTGACTAATTTTTAATTCTA

TGATATATTATAATCAAGGAGAAATATAATGGCACTTAGCGAAACTGAAATCTTAATCTGTAGATTGATAACTGGAGAAG

ATGTTATCGGAAAAATTACAGTAGGTTCAAAAGTTATCACAATACATAAAGGGTACGTTATCATACCAACACAATCAGCA

AAGGGACAACCTATACAGTTGATGATGACGCCTTATGCTCCATATTCAGATGGAGATATTGTTGAAGTCAACGCAGATAA

AGTTGTATCTATAACAAAACCAAAAGAACATATTAAACAAAACTATGTCAGTAGTACTTCATCTATTGTAACACCTAAAA

AACAGTTAATAACTGAAACAGGTTTGCCTACATTAGATAAGTGATAGATGTATATTTTGTAAGGGACGGATCCAAGATTC

GTGTTCAGACTAAAGAAGGTTTGAGTGCAATGGAGGCAGCGAAATTTGAATCACACGTACCAATACCAGAAATTCCTGCC

GATTGTGGTGGTAATTGTATGTGTTGTACGTGCCACGTATATGTTGATGAGAAGTGGATAGACAAAGTACCACAACCAGA

AGACTTATCAATAGAAGAAGAACAATTAGAATATGAAAGAGGATATAAACCAGGTGTTAGTAGATTAAGTTGTCAAATAA

AACTTACTAAAGACCTTGACGGTTTAATTCTCCATTTGAGACCAGATGAACTTTTATAAAAATGTAATAGAACATAAAGG

CAAACTACTTGTTAGAGGTGTAAGAGATAGCAAAGAGTTTAAAGAGAGGATTAATTTTTCTCCAACATTATATTCAGTAT

CACAACATCAAGAAGAATTTAAATCATTACAAGGACATAATTTAAGACCTATTACTTTTTCATCTATTGACGCTGCTCGT

AGATTTAAACGTGATGTCGCTACTAAAAATGCACCAGTCTATGGACTTGATAGATTTCATTATCAATACATCAATGAAAA

GTATACAAAACAAGTTAAGTGGTCAAAAGAATTAATTAAAATATTTACATTAGATATAGAATGTACCTGTGAAAATGGAT

TTCCAGAAGTAAATACTCCAGTTGAAGAACTATTATGTATTACAGTTAAAAATCAATCAAACAAACAAATTATAACGTGG

GGTGTTGGTGAGTTTAAAACTTTACGTACAGACGTAACTTATATTAAATGTACAGATGAAAGACATTTAATAATGGAGTT

TATGAAATTCTGGTTGAAGAACTATCCAGATGTTATTACAGGTTGGAATACTAAATTCTTTGACCTACCATACTTGATGA

ATAGAATTCAATTAGTTGCAGGTGCTAAAGTTGCAAGTAGAATGTCGCCTTGGAACTTAATACATAAAGAAGAAATAATT

ATAAGAGGTAGACCTAATACATATTATTCATTGTTTGGTATTGCAATGTTAGATTATCTTGATTTATATAAATGGTTTAT

ACCAGTAAGACAAGAGAGTTATAGATTATCTTTTATAGGTGAAACTGAATTAGGTGAAACTAAAGTAGAAAATCCATATC

CAACATTTAAAGATTTCTATACAAAAGATTTTCAAAAATTCGTAGAGTATAATATTCAAGACGTAGAAATAGTTGATGGT

TTAGAAGACAAGTTAGGGTTAATTGATTTATCTTTAACCTTTGCGTATGAAACTAAAGTAAACTATAACGATATTTTCTC

ACAGGTGAGAGTTTGGGATACATTAATCGCAAACCACTTGATGACAAAAAAGATTTGTGTACCACCTAGGGAAGACCACA

TAAAGGACACCAAATATGAAGGTGCGTATGTGAAAGAACCTAGATTAGGTATGCAAAAATGGGTGGTGTCTTTTGATATC

AACTCTCTTTATCCACATATTATTGTACAATATAATATTTCTCCCGAAAAAATATTAGGTGTTAAACCATCTGGTGTTTC

TGTGAATAAAATGTTGAGTAAGAAGACACCCCTAGATTATTTAAAAACTGAAGGTGCTTGTATAACACCTAACGGTGCAA

TGTTTAAAAGAGATAGTCAAGGGTTCTTACCTGAAATGATTGAAAAGATTTATAAAGACCGTGTGATATATAAGAAACGT

GAGTTAAAAGCACAAAGAGAATATCAAAAGAATCCAACAAACGAATTAAAAAAAGAAATTGCTAGATGTCATAATGTACA

ATGGGCAAGAAAGATTGCATTAAACAGTTGTTATGGTGCAATAGGTAATCAGTACTTTAGATATTATGATATAGCACAAG

CAAGTGCTGTAACTACAGCAGGACAATTCATTATTAGATTTGTAGAACAAAAAGTAAATGAATATCTAAATCAAATATTA

CAAACACATAATGAAATAGATTATGTACTAGCGTCTGATACAGATTCAATTTATGTATCGTTTGATAAACTTGTAGAGAA

GACTTGTAAAGATAAAACAGACCAACAAGTATGTGATTTTCTTGCTAAGGTATGTGATAATAAATTAGAACCGTTTATCG

CAAAACAATTTGAAGACATTGCAGACTATACAAACGCATTTAAGAACGCAATGGTTATGGCACGTGAAGTTATTGCGAAC

AAAGGTATATGGGTTGCGAAAAAAAGATATATGTTAAATGTATTAGATGAGGAAGATGTAAGATTGTCTGAACCTAAACT

AAAGATTATGGGTGTAGAGGCAATTAAATCTTCAACTCCACAAGTATGCCGAGGTAAGATTAAAGAAGCAATTAAAATAA

TTATGTCAAAAGAACAATCTGATTTACATACTTTCATTGCAGGTTTCAAAAAAGAATTTATGAGTATGTCTGCTGAGCAG

ATATCATTTCCAAGGTCTTGTAATAATATGAGAAAATATGCTAGTAGTAAAGATGTGTTTATCAAAGGTACACCAATACA

CGTTAAAGGTTCTTTGATTTATAATCATCAAATAAAAGAATTTGGATTGCAGAATAAGTATCCTTATATACAAGAAGGAG

ATAAGATTAAGTTTATTAAATTACTACAAGCAAATCCATTTAAGTTTGATGTGATTAGTTATATAACTAAACTACCAAAA

GAGTTTAATCTACAAGAGTATATTGATTATGAAGTACAATTTGAGAAAACTTTCCTAGACCCTATGAGATTTATATTAAA

CTCAATAGGTTGGGAACACGAAAAGAAAGCAAGTCTGGAAGCGTTTTTAGGATGAAAGTATATATTTTTTGTCAAAGTCA

AATAGGTAATGGACATTATGTTAGATGTAATAATATAAGAAAAGGTCTTGATGATTGTAAGTTTGAATATATAACTGGTA

CATTTACAGATGATGAAAGAAGAACAATATTTACTAAGCAATTAGAAATAATAAATGATTATAAACCAGATGTTATATTA

TTGGATGGATTTCCTTTTATGAGATATGAATGGTTTGATTCTGGTATGGAATTTTTATTAAAGTCTGTAAATAATAATGT

AAAAATTGTATCATCTATTAGAGATATTTGTTATCCATTTAAAGGAGGTAAAAAGCCAACAAGATTTCCTGCTAGAACTG

TTGAGTGGGCAAATGAATATATTGACGTTATACTTGTACACGGAGATAAAAATTTTATTGAGTTAGATGAAAGTTTTGAA

CATTTAAATTTAATTGATCCACCTGTTTACTATACAGGTTATGTAACTGACACATATAAACCAGAACCACAGAAAAGAAA

TGGTACAGTTGTATCTGCTGGAGGTGGTAGAGTTGCAGAAGAAGTTTTTGATAAGGCAATGGAATTATATGATGGAACAG

ATTGGACTTTTTCTGTTGGTCCTAATTATCCAAAAGAACATTTAGAAAAATTTAAACAATGGGCAAGTGATAAGAAAAAA

GTAAAGATAGTTTATAATGTTAAAAATTTTAGAGATTTACTTGCTAAACATAAACTATCTATTAGTCAGGCAGGATATAA

TACAGTTATGGACTTATGGATGACTGATACACCTGCAAAATTTATTCCATATGTAGACCAATTTGGAGAACAAGAACAAA

CTACAAGAGCAAGACTTGTAGATAATATGAAAACTAAACCAGATATGAACGGTGTACAAAAGACAAAACAATTATTGGAG

AAAATATGCGTGAATTAATTTTAAGGGATGATGATTGTTTTGAGTTGACACAAAGCACTCGTAGATTTTTAGAACTTACA

AAAAGGATACCAGTAATGTTGGCAGTTATTCCTGGTAGTATAAAATTTAATCTTGTAGAATTAATTAAAAAATATCCTAA

TGTAACTGTAGTACAGCACGGTTGGAAACATATTAATAGTGCAGATAAAGATAAACCAAAATTTGAAAAGTTTGATAGGT

TAGATATACAAACAGGTAAAAGTATGTTAGAATCATTATTTAAGAAGCAATTTTATTCTTGTTTTGTTCCACCGTGGAAT

AAATTTGATGGTGATTATAAGTTGTTATATGATATGGGATTTAAAAAAGTTTCTGATAGTAAAAATGTAATTGATTTGAT

GAAAGTTAAAGATAACGTTGTGGAAGAAATTAAAGTACCAGAAAATGATACAATAATGACACACCATACACATAAAAATT

GGGATGATAAATGTTGGTTGTGTTTAGAAGTGTTAATTGAACAAGAGAATATAAAATGGAAAACTATAAAATCTTAATAA

CAGGTGGTCACGGATTTATTGGTTGCCACATATCACATATATTAAAAAAGTTAGGTCATACCGTAGGTGTAATAGACAAC

TATACAGATTACAAATACTATGATGTTAGATTGTATAGGAAAGTATTAGCGCAAAGAATAAAATATGCTAAGGCAGATAA

TATATATTTAAGAGATATTTTAAAATCTGAAACTGTATTTGAAAGTTTTAAACCTGATATAGTTATACATTTAGCAAGTT

GTCCTAATGCTAATATGTTATTAGGTAATATTGAACAAGAAACTAAAACAGCAATTACTGGTACTTTAAAGATTTTACAA

TTGTGTGTTAAACATAAAGTAAAAAGAATTGTATATGCGTCTTCAAGTATGGTGTATGGTGATTTTTTAACACAAGCACC

AGATGAATTTCATCCAACAGACCCTAAAACACTTTATGGTTCTTATAAATTAGCAGGCGAACAAATGATTAAATCTTTTA

ATAAGGATTATGAATTAGAATATTCTATATTAAGACCTAGTGCAATATATGGAACAAGAGATATGATTATAAGAGTTATA

AGTAAAATGGCAAAAAGTGCTATAGAAAAAAGTGAAATTGATGTTAATGGTGTTCATAGTAAATTAGATTTTACAGATGT

TTCAGAAGTTGCAGAAGCATTTATACACGGTGCATTGCATAAAGGTGCTGTAAATCAGATTTTTAATTGTTCAAGAGGAC

GAGGTAGAACTATAGTAGAAGCGGCAAGAATTGTTAAAGATTGTATGGGACAGTTTAGTTGTAAATTAAATATTAAAGAT

TCGGATTCTTTTTATCCAACTAGAGATACTTTAGATAATAGTAAATTGAAAAAAATAACAGGATGGGAACCAAAAATAGA

TATTGAATATGGTATTAGAAAATATATAAGTTGGTTTAAGGAGAATAATGAATAACTTTATAATGTTTTTTGCTGTATTG

TTTGGAGGTTTTCTAGCACAGAATACTAATATAAAACTCTACCAGTTTATTATATTCTTATTAGTAGTGAGATTTTTAGG

TAAGGCATATGGATATTAAAAACGCAGAAAGTTTAGAACATTTAAAAACACTTGAAGATAATAGATTTGATTCGTGTGTA

ACTGATCCACCATATCATTTGGCGTCTATACTTAAACGATTTGGTCCAGGTCAAAAAGGCATTAATAATAAAGATGAGAA

AGAAGGACGTAATGGACCTTATCATAGAGCGGCGAAAGGATTTATGGGACAGACTTGGGACGGTGGTGATATAGCATTTA

ATAAAGATTTTTGGAAAGAAGTATTAAGAGTTATGAAACCAGGTGCAGTACTCTTATCATTTGCTGCCACTAGAAACTAT

CATAGAATGGCAGTTGCAGTAGAAGACGCTGGGTTTGAAATATTTGATATGATTAATTGGATGTATGGTAGTGGATTTCC

TAAAAGAAAAAACTATTTAAAACCTGGTCACGAACCTATAGTAATGGCACGTAAAGGAGTTAATAAAAGTTTAAACATAG

ATGAGAGTAGAGTACCTGGATACGAGTGGGACACAACTAAAAACAGAAGAGAACCTAAAAAACATAAAGAAGCAATTTAT

AAATTAGGTTTAAAGAAAACAGGTACAGGAGAAAAAATAGAAGGAAGATATCCTGCTAATGTTATACACGATGGATCATA

TTATGAAGAATGGGCAAAGTATTTTTATTGTGCCAAGGCAAGTAAAAAAGAAAAAGGAAATACAGAACACCCTACAGTTA

AACCATTAGAGTTAATGAGATATCTTGTTAAGTTAGTTACACCTAAAGATGGAACAGTATTGGATCCATTTGCAGGTACA

GGTACTACTGGTGAAGCGGCGTTATTAGAAGGTCGTAAGTATTACTTGATAGAAAGAGAAAAGAATTATTTTAAAGACAT

AGAGAAGAGATTAAAGAAAGTGAATCCGTTTTTTGTATGACAATTTTATTATCAATGTTATTTGTATTATTAATTTATGC

AATACCTTTATGTTTATTATTAATGTGGAACAATGAAAAACCTAGACCTTAAACAATTCGCAGACGAAAATAGATTGCCT

ATTATGGACTCTATTCAATTTAAAAATTGGACAGATGAAATAGGTAAAGAAAAATTTAGAGAACTATTAGCAGAATATAT

TGCTGAACATAGACCAGAATTTCCTTTAAATAAAATTTCATATGATGTTATGAAAGATAATATAATAAAATTAAGTAAGT

TTGATACTAGCAAACTTTGTACACCTAATGAACAAAGTCATAAAGATATATTTGAAAAGTATGATGACTATAAGTATCCT

TATTCAAAATATGGTCTAGGACTAATTGACGCTCCATCAATATATAATAAGTGTAGTAATTATTTTCATCAAGAGTTAAG

ATTAAATTGTTCAAGTTATAGTTTTAGAGCACCAATTGAAGTTTTTAAAAATGGTAATGCAAAAGATATATGGAAATGTT

TAGGTGCATTATGGAGAGGTGTGAATAGTACCAAAGATTTATCACCAAATAGTTATAGAGAAGCAATAAGATTAGGTACA

TATGTTGCAACACAATTTAAACCAGTTGTTGCAAAAACAATATACGATATGACCAATGCAGAAACAGTATTAGATACGAG

TTGTGGTTGGGGAGATAGACTAGCTGGTTTCTTTGCTAGTAAGGCAACACATTATTATGGTTGCGACCCTAATCCAAATA

CGTATAAGAATTATCAAAAACAAATAGAAGAGTATAGTAAATTCTTTAAAAATAAAACTGTTAAGATATGGAATTGTGGT

GCAGAAGATTTACCTTATAACGAACTACCAGATATAGATTGTGCGTTTACAAGTCCACCTTATTTTAGTACTGAACAATA

TAATAAAGGTGGTGATAAAGAAGAACTACAATCTTGGCATAAGTTTAATGAGTATGATAAATGGAGAGATAGTTTTTATC

TTCCAGTTGCAGAAAAGACAATGAGTAAATCAAAATTTATGTTTGTTAATATTATGGATCCAAAGATTAAGGGTACTAGA

TATAGGTCAAGTGATGAACTAGTTGATAGATTTAAAGATAAGTTTTTAGGTCAAATTGGTATGAGGATTATGCAAAGACC

ACAAGGTACTAAAAAGTTTAAAACAAAAGAAGAGTTGAATGTCTTTATGGCAATGACTTATATTGAGAATATTTGGTGTT

TTGGAGAGAAGATAGACTTATTTAAACACGCAAGAGTAGGGACGTTAGAGGCGTTTATATAAATAATAATATGGATTACT

TTTATTTATGTTTGGTTATATTCATCATCAATGATGGTTTTGCTATGTCAAGGCATTACTGTTCCTATTTAAGAAATTTA

CGAAAGAAAATTATAGAGAAACTAACCTATGGTTGGTGGATTTCTATACATAGCGTTATAGATATAGGAAGTATTATTGG

TATGATGGTATATTATAAACACCCACAGCACTTTTGGGTTGTTATTTCCATACCGATAGTTATTATACTATGGTATATAC

CTTTAGGATGGAAAAAGTATCGTGAGAATAACGATTTATAGAAGACCAGATGATTACATTAGTCATAACTTTCTACCAAA

GGAACTTGACTCGGTGAAGGAATTATGTTATATTAATAATATAAAATTTTATGTATTAAATTATTCAGAAGAGGAATATA

AAGAGTATGAAAGACTTTCTAAAAGAGATTATTAAAGAAACAGGAAATGAATTTGCTAGTTTAGCAAGTGAAGGTATCAC

AGCAGGTGATGTAACTTCATTTATAGATACAGGTTCTTATTCTTTTAATGCTCTTCTTTCAGGTTCAATTTACGGTGGGT

TACCAGGCAACCGTATTACAGCAATTGCAGGTGAGGCAGCAACTGGTAAAACATTTTTCGCATTAGGTATTCTCAAACGA

TTTTTAGATAAAGACAAAGACGCAGGCGTTGTTCTGTTTGAATCAGAAAATGCAGTATCAAAAGATATGATAGAGTCAAG

AGGTGTTGATAGTAAAAGAGTTGTAGTAGTACCAGTATCAACAGTACAAGAATTTAGAGCGCAGGCAATTAAAATAGTAG

ACAAATATTTAGAACAAGAAGAAAAAGATAGACAACCTTTAATGTTTGTGTTAGATAGTTTAGGAATGCTATCTACTACA

AAAGAAATGACAGACACAGCAGAAGGTAAAGAAACAAGAGATATGACAAGGTCACAAATTGTCAAATCTACATTTAGAGT

TTTAACACTTAAACTAGGACAAGCAAATGTTCCTTTGTTAATGACCAATCACACGTATGATGTTATTGGTTCAATGTTCC

CACAAAAAGAAATGGGTGGCGGTTCAGGATTGAAATACGCTGCTTCAACAATCATCTATCTTGGTAAACGAAAAGAGAAA

TTGGGTACCGAGGTTATTGGAAATATTATTCATTGTAAAATATATAAATCAAGAATTACTAAAGAAAATGCTAAAGTGGA

TGTTAAGTTAACTTATAAACACGGTTTGGATAAGCATTATGGACTATTAGAACTAGGAGAAGAGGCAGGTATCTTTAAGA

AAGTATCAACAAGATATGAAATGCCAGATGGTTCTAAAGTATTTGGTAAACAAGTTAATGATAATCCAGATAAGTATTTT

ACAAAGGAAGTATTAGATAAGATAGATGAACACGCAAGACACAAATTTACCTACGGATCAGAAGAGTAAACCTTACGTAT

TTGTACAAAGAGATAAAGATGATTTCTCTTGTATCAAAATAACAGAAGGTAAATATAAAGATATAATCTTCCATTACGGC

AAGGTTGGGTTTGGAAAAGATGAGAATCCAGATGGAACTTTGCCTATGAAGTTTGATTATACAGTAATAAAAAATCCCAA

TGATATGGATACACTTGACAATAAAGAATTTATAGATTATATTGGTGACTTATTGATAGAATTATTAGATGAGAAAATAA

AAAGTGGAAAATAAAAATTTTATTAATGTTTATGATGGTGCATTAGAACCAAATCAATGTCAACATTTGATTGATAAGTT

TGAAGATTCAAAACATCAATGGACTAAAACAGAATTAAAAGGTCATAGGTCTTTTACAGAAATTAATATAAATTTACATT

CAGATTGGCAAGAATATGTGGACATAATATACAAAGTATTAAGACCATATGTTGATAAGTATTGTGAAGATAATAATATA

GATATGACGCACCAATGGCCGAATAAATTTGGATTTGAACAAATCCGTTTTAAGAAATATGAAGTTAATAATCAAGATGA

ATTTAAAGAACACGTTGATGTTATGGACTATGCAAGTGCAAAAAGATTTCTTGTATTCTTTTTATATTTAAAAAAAAATA

AGGGAGGTCAAACCTCTTTTCCTGAATATGATTTGAAAGTTGAACCAAAGCCAGGTAGATTATTAATGTTTCCACCTTTG

TGGACTCATAAACATATAGGACATAAACCAATAGAAGAACCAAAATATATAATAGGAAGTTATTTGCATTACGTATGAGC

GAAAGATTAGAAACAACTATATTAAATAACCTCTTCTTTCAAGAAGAGTATGCTAGAAAAGTATTGCCTTTTTTAAAAGA

AGATTATTTTCCATTAAGAACTGATAAGATTTTATTTACAGAAATATATAAGTTTGTTGAGAAGTATAATAATCTTCCAA

CAAAAGAAGCAATCTTAATTGAATTAGGACAAAGAAAAGATGTTAATGAAGATGAACATAATACATTAAAAGATAATATT

AATTCTATAACTAAATTAGATTCCGATCCACAATGGTTGTTAGATACTACAGAAAAGTTTTGTAAAGATAGAGCAGTACA

TAATGCTGTACTAGATGGTATTAGAATATTAGATAAGAAAGATAGCAAGAGAACGCCAGAAGCGATACCAGGCATACTAG

CAGACGCATTAGCAGTATCATTTGACCAACATATTGGTCACGATTATATAGATGACGCTGATAGAAGATTTAAATGGTAT

CATACTAAAGAAACAAAATATCAATTTGATTTAGATTATATGAATAGAGCAACCAAGGGTGGTGTTCCAAGTAAGACTTT

GAATATTGCATTGGCAGGTACAGGTGTAGGTAAGTCTTTGTTTATGTGTCATTGTGCAAGTGCTTATTTGGCACAAGGTT

TAAATGTATTGTATATCACTTTAGAAATGGCAGAAGAAAGAATTGCTGAAAGAATTGACGCAAACTTATTAGATGTAACT

ATGGACGATTTACATACAATGCCAAAAGAGTTATATGATAATAAGATAGATAAGATAAGAAAGAAGACTGGTGGTAAATT

AATTATTAAAGAATATCCAACTGCCGCTGCTCATAGTGGACACTTTAGAGCATTGTTTAATGAACTTGCATTAAAGAAAA

GTTTTAAAGCAGATGTAGTGTTCATAGATTATTTAAATATATGTGCGTCAAGTAGATTTAAAGGTGGCAACATAGGTTCT

TATTTCTATATCAAAGCAATTGCAGAAGAATTAAGAGGACTTGCAGTAGAATTTAATGTACCTTTGTTTTCTGCTACACA

AACAACAAGAACTGGATTTATGAGTACAGACATAGGACTAGAAGATACGGCAGAAAGTTTTGGATTGCCAGCAACAGCAG

ACTTTATGTTTGCAATAATATCCAATGATGATTTAGAAGCATTAGGTCAGTTAAAGATTAAACAATTGAAGAATAGATAT

AATGACCCAGGAATTAATAGGTCATTTATTATAGGGGTTGATAGACCTAAAATGAGATTGTATGATGTAGGTCAAACAGC

ACAAAACATAGTTGACTCTAATCAAAAGGAAACAAAAGAAGAGAAAACTGCTTACGATAAGTTTTCAGATTTTAAAGTAT

GATGAGAAAGAAAACTATTTTTACAGTAGATATATATGAGAAATATAATTTTCTAAATGATACAGAAATAGATAAATTAA

TTAATAGCATTAATAAAGAAGATTTAGGGCAGTATGATTACTTTAAAGGTAATGCTAAAACAACATATGTTAATATGGGA

GGCAATGCTTATATTTTAGACTATCATAAAGATATAGAAGATAAAATTATGAACGAAGTTTATACACCCAATCAAAGAAT

GCAAGACTCTTGGTGCAATATACAAGGTGAAGATAGTACACTAAACTATCATAGTCATCCTAATTCAATTTGCTCTGGTG

TTATATTTTTAAAAGTAGATGAAAATAGTAGCAAGTTAGTTTTTCAAAACCCTTTTTCTTTTACAAAAGAAACTTATCAA

ATAACACCTACAAAAGGAATGATGGCAATGTGGCCGAGTTTTTTAATGCACGGTTCAGGAGATAGTATTAATAAAAGTAA

AGAAAGGACTGTATTAAGTTTTAACACATATTGGAAGTAATATGGGAATGAAAGTAGGCGTTAAAGGAAAAGTATTTGAT

GGTGCTTGGAGACCAGTAAATAAAGCATACAAAGATGGACACGAAAGAATCTTTGGTGCAAAGAAAAAGAAAAAAGAAAA

GAAGGTAGTAAAAAAAGTAGTGAAGAAAAAACAAGAACCACAATTAATAGATAGTAGTAAGATGATATGAAAAGTTTAGT

TGAAGAATGGGATAAATTTTTTAAACAACACCCAACTGGTGGACCTTGGGATTATAAAAATCCTAGTATGGAGCGAAAGC

TACAAGATGGTCACGTGGTTGATTTTATAAAGTATTATAATTTTGAAAAAAATTTAAAGGTTTTGGATTGTGGATGTGCA

GATGGTAGAAATTCTGAATGGCTTATAGCCGAGGGGTTTGAGGTAACAGGTGTAGATTTTTCACAAACGGTGATAGAAAG

AACTCAAAAGCGTTTACCAAAAGGAAAATTTCTTGTTGGAGATATAAGAAAATTAGATGAGATTGAAGAAAATAGTTTTG

ATTTTCTTATTGACGCTGGAGCTTTCCACGTGAACTATCCAGAAGATACTCTATCCATTATAAAAGAGTATCATAGAATA

TTAAAAAATTCAGGAAAAATGTTTATTAGAGTTTTCAATAAGGAAGACCATACATCTAATCCTATTTTTACTATAAATGA

AGACTTAACTATGCCTGTGTTTGGGTATAGTCAGTTTGTATTTACTAATCACATTAAAAATTATTTTAATGTTAAACATA

AAATATATGATCCTAACTATGGAATGCACGGTCAAGGATGTAGTTATTATTATTTGGAAAGTATATGAAAAAACAAAAAG

TAAGGTTCAGTAGAGGCGATAAGAGACCAGCAAAGTATACCTATAAACTTTCTTACGAAAAGAAGTTAGTTAAGAAAGGT

AAGAAAATAGTTTGGCACGTCATAGAGAAACCAACTGGTTCTGTATTATCAGAATTCTTTTTTGAAGAGGATGCTGACAA

GTTAGCGAAGTTCCAAAATAAGAATAAAGTCTGGCAAGAGAACGGTGGTATAGTTAAACACCTGTGTTTCCAATCTAAAT

AATTCCAATATAAATAGTCATAGGAGAGAATATGGCATACGAAATTTCAGAAATCTGTACAGCGGCAGCATTAATGTTCA

CTACAGATGAACTAAAAGAATTACAGTCAGGATTTAATTCAGGTGAATTAACTAGGGATGATTTATTAGCAAAACTAGAA

GAAGCTAAATCACTTATGAAGATTGGTAAACCTAAAGCTAAAGTTGGGCAGGTTGTTTTTACAGATGGTAGCCAACAACA

AGGGTTTATGAATTTAATTACTGACAAGAAAGCAAGTAATCCAGATAATAAAGTATTAGATAATTTTGCTGTTGGTTTAT

CAGCGGCATTAGGTATTCGTGGTTTTGCTAGAAAAAAAGGTGGTACTCAACCAACTAAAAAAGTTTTTATGACTGGCTCT

AAATGGCCGAGTGAGATTGAAAAGTTTTCTTTACCAGAAGGAAGTTTTAATTATAATTCTGCTGATATATTGGTTGAAAA

TAATTCTGCTAAAGCAAAAGTAAAAAAATATTATGGTATTTCTTTAAAGAAAAAACCTACACCAGCAGCGAAACCACCAC

CACTTATTAATAAAGCTTTTGATACAGTTGTGGCAGGTGATAAAGATTTTGAGAAATTATTGGAAGTACTTGACAAACAA

AAACATAAATTTTTTGCTGGGTTACTTAAAAAAATAATTGGAAAAGGTAAATTATTGCAATTGAGAGGGATGACAATGCC

AACAAGTGATGAAGATATATTTAATATGAAAATTAAACATCCATTTAAGTCTGGAAATATAAGACTAATATCTATAAAAA

ATGGTGGAATAGTAAAACCAGTTTTTAATATGTCGTTGGCGGGAGATAAAAAATTAGCAGAAATATTATTTGCAATACCT

AAAGAAGAATATCCAGAAGCTAAATGGAAAGTGAGGCAGTATTTTAATCAAGCTTTATATGGTGGAGGTAAAAGTAAATA

TTGGCAAGGGGTACTAAAAATATTGGATGAGCATTCTGAAAAATTTGCAGAAGCACTTATTGATGTTATTTTAAAAGTTA

ATTTATATAATAAATTAAAGAAACAAGATGTAGATGAATCGGAGTTTGATTTTCAAGTAACACAAGGTATAGGTAGAGTT

ACTCCAAAAGGTAGGGTAAGTATAAATGATTCAACAAATTTTCCAATTAATACTTTACTTTGTGGTTATAATAGAATTCA

GCAAAAAGAATTTAAGAATAAAAATTGGAATATTAAATTGAGAGATGAAGTAGATGAAGACGGTAAACCTGTTAAGGGTG

ATACAATAGACAAGGCAGCAAAAATTAAAATGTGGTTAATGAAAGGTGACGACCAAAAAGTATTAGATTTAGAATTAAGA

TATAAAGGTAAATTTGGTCAACAACCACAATTTACTGGCACTCTTCATCCAGATTTCAAAAAATTATTAGATAAAGAATG

TTTTTAATTATATATGGTCTTCAACAAAGTCAGATAACTGTCTGAACAAAGAACTTAAATCATAAGTTTGAATTTGTACA

GCGTCATCTTCATCTTCATACTTTGTACTATAATCATCTATAAGAGTATTCATTTTCGTTTCAGCTTTATCTAAAACTTT

TTGAATTTCCTCTTGTAAAATGTCAAAGTCTTTTTCGTGTGGTTTCATATGTATATACTATCAGTTTTGGATGGGACAAT

CTAGTGAAATCGTAATCAATCTTTTTCACGTAGTAAAATAGTATAAATAGTAGTATATGATTTGTTAATGGATATGTGAA

TATAGATTATACTAATGGAATAAATGAGGAATAATGTTTAGTTTTAAAGGTTTTACAACAAACGATAAGAACACACACCT

AGAACACCTAGAAGATGATATAATCAATAGAGGTTCATCTGGTGGAGTTAACGCAATTAACTTTCTAAAATCAGTAAGAG

ATATGCTCGCAGGTCACTCGGGAGCAAAAATCAATACTACTGTTAAATGGGATGGGGCACCTGCTATTATATGTGGGGTC

AATCCTGAAAACGGTAAATTCTTTGTTGGTACAAAATCAATATTCAACGTAACCCCTAAAATCAATTACACAACAGCAGA

CATAAGACGGAACCATTCTGGTCCTGTTGCTCAAAAACTATCAGTATGTCTTAATCATTTATCTAGTCTAAACATTAAAC

AAATTTTACAAGGTGATTTATTATTCACTAACGATAAAAAATCAGCTTCAATTGATGGTGAAAAAATGATAACCTTTACA

CCAAATACAATCACATATGCAGTACAGGCAAGTAGCAATATTGGTAAGAAGATTGCTCGTGCTAAAATGGGTATAGTATT

TCATACAATGTATACTGGTAAAGATATGAAAAGTTTAAATGCAAGTTTTGGTAATGTTAGAGGGTCAGGTAATGCAAGAG

TATGGGTTGCGAGTGCTGAATATAAGGATGACTCTGGTTCTATTACTTTTACTAAATCAGAACTAGGAAAATTTGATGCT

CAATTAAGAATGGCAGAAGGTTCTTTAGGTAGAGCAAGTAAGATATTAGATGAAATGACTAGTCGTGCTAGTGACCCTTT

ATCTGTAGGGTTTAGATTAAAAGCATTTTTCAATCATTACATTAGAAATAATAAAGGCAGTATGGCAAAGGTTAGAGTCT

TGCAAGATATGTTTAGAGATTATTATGAAAACATTTTGAAGACAGAAATAGACCAAAGAAAAACTGAAAAAGCAAAACAA

AAATATAGAGATATATTAGCAGATGGATTAAGATTTATTAATCAAAATAAATCAGGTCTATATATGGCAATTGCAAGTCA

CGTAACTTTAGGTAATGCGAAGAACACATTGATACAAAAGATGAATCAAATTCAACAGATAGGACACTACATTAAAACTG

GAACAGGATATAGAGTAACAGCACCTGAAGGATATGTTGCAGTAGATAGAGTAGCAGGTGCAGTAAAAATAGTAGATAGA

TTAGAATTTAGTAGGGCAAACTTTACGTTGCCAAAAGGATGGAAATAAATGAAGTTTAAAGAATTTTTATTACAAGAAGG

TGTATACGATCCAGGTATATTTAAAGCCTTTTTTATGGCAGGTGGACCAGGTTCAGGAAAAACCTTTATCGCTTCTTCCG

CATTTGCTGGAACTGGATTAAAGTTTGTTAATTCAGATACAATATTTGAAAGAGCATTAAAACAGGCAGGTCTTTCAGAC

AAAATGCCTGACCAAGAAGCATATTTTAGAGACCTTATAAGAGCCAGAGCAAAAGGTACAATGATGAAACAATTACATAC

CTATGTAGGGGGCAGATTAGGTTTAGTTATTGACGCAACTGGAAGAGATTATGGAAGAATAAGTTCCGAATATAATATGC

TACACGCATTAGGTTATGATTGTTATATGATATTTGTTAACACAACATTACCTGTTGCGTTAGAAAGAAATAAAATAAGA

AGTAGACAAATACCAGAATATGTTGTGCAAACATCTTGGGAGAAAGTACAATCTAATATAGGTAAGTTTCAAAGATTATT

TGGTCAATCAAATTTTATTGTAGTAGATAACAATAGGTCAGATAAAGAATTGGTAACAGCAACATTAGCAGGTTGTGATA

GATTAGTTAGACGATATATGAGAGCACCAGTTAAAAGTCATATTGCAAAAAATTGGATGTCAAGAGAACAAATGTTTAAG

AATACAATGTTTAGTGTTGGTAGAAGATTGGTAATGGGAGAAAGTATTATAGATGTACCTAGAAGTAGATATGCTGTTGG

TGTATTTGATAATGCTGAAACAGAAAATCCAAAATTAAAACCAAAAGTTTTTGATATGATTAAAGCAGGTGCAAATCATT

TTTCAAAATGGGGTCCTGTTGTGTCAGTAAAATTAATAGGTTCTATTCTTGGAAAGAGATATAGAAATGACGCAGATTTA

GATATTGATGTATTGATAGACATACCAGAAAAAGATAGAGAAACAGTTGGTTTAGAAGCAAGACAATCTGTAGGAGAAGT

TAGTGGTAAGTTAGTACCAGGTACGCAACACCCGATTAATTATTATGTTCAAACAGACCCTAAAGTAAATGACGCTCATC

TTGCAACTTCTATAGGAGTTTATGATGTATTCAAACAGAAATTTGATAAGAGACCTCCAGCACAAACGTTTGATCCTAAA

GTATATGAGGCAGAATTTCAAAAGAAAGTGGCAGAACTAGATGTAGTTAAAGGAGAATTACAAAGAGATATAGTTGACTA

TGAAGAGTTAAAAGAATTAAAACCAAATGATGTATTAGATTTACATATAAAAACTGCTGAGAAATTAGAAGAGATAGAGG

ATGCTATAAAGAAGATGTCCGAAATGGGTGATAACCTTATGCAGGCAAGAAGAGATTTATTTAATAGACCTTTGACACCA

GAAGAGATTAAAAAATATGGTATCGCTCATAAGTTACCTAAAAATGTTGTCTATAAGTATTTGGAGAAATTTCATTACAC

AAAATTCTTTTACAAATGTAAAAAAATATTAGAAGATGAAAAGGTAACAGACGCAGAAATAAAAAGTTTAAAATCAGAAG

CAGTAGGAGAAAAATCTATTGCAATTACATTTGGAAGATTTAATCCTCCAACAATAGGTCACGAAAAACTTATTAATAAA

GTTGTAAGAGCAGATAGAAATTATAAAATCTATATCAGTAGGTCAGAAGATAGTAAAAAGAATCCATTATCTCCTAGAGA

AAAATTATCTTGGATGAAAAAGATATTTCCACAATATGCTAGAAACATTGAAATTAATACAACAAATATGATATTAGATA

TTGCTACTATGTTATATAACAAAGGGCATAATGTTCTTAAATTTGTAGTAGGTAGTGATAGAGTAAGAGAATTTGAAACG

ATACTTAAAAAATATAACAATGAAAAGAATAGACACGGATTCTATAACTTCAAAACAATAGATGTTATATCTGCTGGAGA

GCGTGATCCAGACGCTGAGGGCGCTTCAGGTATGAGTGCGAGCAAGATGAGGGATGCTGCTCAAAAAGGTGATGTAGCGT

CATTTAAGAGAGGACTACCATCTCATTTTAGAGATGTTGATGGACTGTTTAAAGCAGTAAGAAAAGGTATGGGTATAAGA

GAAGACTATAAACCAGATATTTCAAAACCTGTTATGACATTAGGACAGTTTGAACAGAAACAAGTTAGAGACCTATACGT

TAGGGAGATGATATTTAATATTGGAGACCAAGTCAAGTATCTTAAAGAAGATAAACAAGGTAAGGTAGTACGAAGAGGTA

CGAACTATGTTGTACTAGAAGATACGAATAATAATTTACACAAATGCTGGATATGGGATTGTATACCAATTGCTGCTGAC

AAAGAACCAATGTTAAGAGAATACAACCTAGATGTTGATTATGGATTTGAAGCAGTTGAAACAATACCAGTACCTAAACC

TTACACAGCAATTAGAGATAGTTATGAAATAGGTGCTGACTATGCTAATCATTGTAAACAGATGACACCAGGAGAAAAAG

AGGATGCACCTCCTGTTGACTCAAAAGACCGAGGAAAACCTACTGATACATACATATCACGCCCAGGTAAGGCAACGGAT

GCTAAAGTTGGTGAGGATAAATTAACTACAAAGGAAGTAAAAGAATGGTCAAACCAAGATTCAACAATAGATAAATATAA

ACAACGTTATAAAGAAGAGTGGAAAGCGAAACTAAACGAAGTAGTCGCTAAGATGATTGAGAAACTTTAGAGAGAGAAAT

GGCAACTAAATTCAAAGAATACGTACAAAAACTATACGTAGCAGAAAGTTCGGCTATGGTCTTAAAGGGAGTTGATGATT

ACCTTAAAATCGCTAGGGAAAAAATCAAAAGACACCCACAATTTGCCAATTTATATAGAGACCAAAAACGTGATGTAGCT

ACAAGTGTTGGTGGTAAGTATATCAAAATTTGGGATACGGAACGAGGACAAAAGAGAAGTATCCACGCCTTCGTAGATAA

GGTAACAGGAGATGTTTTGAAGGCAGCTGGTATTAATGCTCCAGCAAAAGGTGCTAGAGGAAACGTTTTAGATAGAAAGT

ATATGGACTCATTAAATCGTGTATTTGACACACACGGTGGACACTTATACAGCAGACATAGTTTATCATACAATTTCAAA

AGACAAAACATATTTAAATAAAATGGGCGACTTAAAAGAACAATTAGAAAAGTGGAAACAAAGTAGGTATAGAGAACCTA

TGAGTTTGACTCGCTATAAAATGAGCGAAGGTTTTGAGTGGGGAGTCCAAACTTTTTTAAAGAAACACGGCATTGGTCGT

ACTAGATTTTCATACGGTATCTTATTCCTTCCAAGAGCGGATGTGGAAAAAGCTAAATCATTACTAAAAAAAGAAGTAGA

AAAAACTAATGGTGATGTAGCAATAATGCCTAAATCAATTTTAGGTGAAGAACACGATTGTTCAAAAGTACACCCAGGTA

AAAAACATAGCGAATGGTTGAATAAAGAAGAACAACTAGAAGAACTTGATGAAGGTAGAATGAAAGATATCTACACGATG

GATCAAGAAGGTAAATCTAAAGAAGAGATTGCTAAAAGATTAAAACTAAAAGTATCAACTGTAAAAACTATTTTAGGTGA

AGAAGTATTTGCTGAATTTACTGACACTCAAATAGCAAGTTTGAAAAAAGATTACGCAGGATTACAAGGTAAAACTATAA

GTGGAATTAATGCAAACAAACTTATGAAAATTTTTGATAAGTTTGATAAGAGCAAACCGCTTTTAATAAAGTTATTAAAA

GCAAAGATTCCATTTGTGTCAATGTTAGCGCAAGCAAGACTCATTTCAAAACACGGAGCTAATGCTGCCCAATTGGCACA

AATGAGAAGAGAACAGCTTGATGAAAAAGCAGAATTTAGATTGTCTTATAGTGATAAGTACGGTAAACACGCAGGTTTTG

AAGACGCAAAAACTTTACAAGATTTACAAAACAGAGCACAGAAATTAAGAAGTAAGGGATTTAAGATTGATAAAATGGGT

AGAAATACATCACCTGTTGAACAAAGATTACCAGAACCAGAAGGCAAACAAATTGCTACAGAAGCAAAATATAATTATAA

TATTAAGATATCATCCGATTACGGTAATGATGGTGGGGATCAAGAAGATGAAGGAACATTAAATGCTTCTAATGATAAAG

ACGCACAAAAACAAGCAGATAAATTAGCAGATAAATTTGCTGAGTTGTGGAATAAACGAAAGAGGTCTGTTGGTAGACCT

GGTGGTTTTGATCCAGTAGAAATTGATGTATATAAAGAAGCATATACAGTACAGATTACAAAAACAGATGGAAGTAAATT

AGTTATTGGTAAATATAATACACCTGCTGAAGCAGAAAAATATATTAGTTGGTATAAAACAGGTGATATGAGTAAGACTA

AATCAGCTAAAGTAATTAAAGAAGGCAGGTGGGCAATTTCTGGTATTACAGGATATAAAAACATATCTGGTCAAGATAGA

TTTAAAATGATTATTAGTGCGTCTAGTAAACAGGATGCTGAAAGAAAATGGGAAAAAGAATTAGATAAACATAGAAAGAA

AAGAAACATAGGACCAGGTGGTGGTGGAAGTATTGAAGATCCAGATGATATTGAAGTAGAACCAGCTGGACCAAAAGATA

GAGTCGGAGATATAGAATATTCTATGACTCATAGTTATGATCCATCTTATGGAAAAATAAAAGAAGATAGTGGATATTTA

CAAAGTAAAATGAGTGATAAACAAATTGCTAATATTAAGGGTGTATGGAAGAATAAAAAAGCAACAGATGTAACCGACGC

TGTTAAACAAATGATTAAGAGAATGGATATACCTACACAACTTGCAATTAAAGCGGCAGACATACCACACATATCAAAAT

TAGTAGAAGATGATGACAAAGCATATGCAATAGGTATGGCAAAGGCAAAAGAGATTAAAAAGGATAGCGGAACACCGTTG

AAAAAATCAACGATAGTAAAAGGTCACGAAATTGCTAAGGCAATTAAAAAGGACGAGCACGTAGGTGCTAAAATGACTTT

TGAAAGATTGTGGTCCCAGCATAAACGAGGAGATAAATAGTATTATGGCATATTTAAAACAAAAACCAGGCAGTATTGAG

GATGTGGTATATAAACATCAAGCGAAGTATAAACAAGAAGGTTATAAAAAGAAATTCGCTGAAGCATTAAAAAATACTAT

GGGTGGTATTGGTTCAATGACACCTAAAGAAAAGATAGCATTTTTTAATAAAGTAGAAGAAGAATGGAAACCTTCTACAG

GAAAACACGCTGATGAGCAGTTGATGAAAGACTTTATTGACAAAGGTGGTAAAGTAGAAAAGATACCTGAAAATAAAAGA

GCATATAATGGCAATAGAATCAAACCTCATTTAGCAAACGACAAGAATTTAGCAAGACAAGAAGCAATGTCGGAAGAAAC

TGTTTCAGAAGCAATGCCAGGTGGAGCTAATTCATCTTCAAGAAGAGGTACTGCTTCAAGTGGTGTTAGGAAAAAATATA

GATTTGGTTATAGAGTTAGTGAGAAAGAACCTAAAGGCAAAGATATAGAAGAAGAATTACCAAGACAACTTAAAGACCCT

AAAAAAGAAACAATGGTTTCTAAACACGGCAAGACTATCGTAATAGATAAGACTAAAGAAAAAGAATATTTAGCAAAAGG

ATGGAAACTTGCAGAAGGATATGATACAGATACACCTTTTGCAATATCAGGTGTAGAATCACAAAGACCTACAGAAGATA

TGCAAGAAACGATTGACGCTGGAGAAGTATCTAAAATGGCTGATAAGAAAAAAGAAGTTAAGTTTAGTACAGCAAATGTA

TCTACTACAGAAGATAAAAAAGAACCAAAGAAAGATGTTATTGGTAGTGATAAACCAGAACCATCAGTAAATTTAGAAAA

TACTATTAGAAATATTTGGAATAAAGCAGCTAATGAAACAGCAACAAGAGGAGATTCTGTACTGTTACCTACAAGAAATG

AAAGCAAAATTCCACCTATTGCAAAAGATAATAAACCAGGTGTAAAGATTGCAAAGATAAGAGCAACAAGGGATTCTAAA

GATAGTAAAGATGGACCAAGTGCTAAAGACCCAGCAGCACAAGAGAAACAAATTTTAACGTTGCAAGGTCAAGTAAATAT

ATTAAAAGCAAAATTAGAAAATGAAAAAAATAAAGTAATTAAACCTGTTGCAGATAAAGAAACAGGAGAAGTTCCTTTAA

CAGTTGGACTAGCACAAAAACTTTTAAGAGATAAAGCAGAAAAAAAAGAAGACAAAGAAGACAAAGACGAAGTTAAAAAA

GAAGCAGTAAGTCCTTACAAACTAAAATATGAAACTTTAAGAGCAAGACTTAAAGAAAAAGCAGAAAAAGCAAAACTTGC

TAAAAAGAAAGATGAACCTACCAAAGGTAGAACTATGACTGGTAACCCTGCTTCAAAAATTGAAACTGATCCAGAGATAA

GTTATACTAATTAAAGGAGGCAATTCAGATTATGCCTCTTCCTAAACTCTATTGTGATATGGACGGCGTATTGGCAGACT

TTAAAAAAGGTGCCGAGAAAGCAACAGGAGTCCCTATCAGTAAATGGATGTCCCTAACAAAAAAAGAAAAATGGAACCCA

ATCAGAAATGATAAAACTTTTTGGGAAAGATTACCTTGGATTTCTGATGGTAAACAATTGTGGAACTATATCAAAAAACA

TAAGCCAGATATATTATCAGCGTATGTTAAGCGTGATATAGACCCGAACTGTATACCAGGTAAAAGTAAATGGTGCAGAA

GTCAATTAGGTTTAAGTGGTGCAAGAGTTAATTTAGTTTTAAGAAGTCAGAAACAAAATTATGCTCAAACAGGATACAGA

AGTCCTGCAGTATTAGTTGACGATTATAAACCTAATACGGATGCATTTACAAGTCGTGGTGGTATAGGAATATATCATAC

TTCAGCGTCTAATTCAATAAGACAGCTAAAACAATTAGGTTTCTAACACACCTTTTTCTTATAAATATACACATATATTA

AGAATTGAGTACTTTAACATAAAATATTAAAGGAGAGAATAATATGTCAAGTCACACTAATAAAGACGAAGCGGCTGGAG

CACCCTTATGGGCAGTAGCGGCAATTAGATTGCCGTTTACTAGTGTTCACCGTACGAACCTTTTTAATGACGCAACTGCT

GACAATTTCATCACAGGTGTTACTATCGGATTGTTTAATTACAAAGATAGTGAAGTATCAGATGGAAAAGTAGCTCACGC

AGGTTGGAATTTAAAAACAACTGGTTCAGGTGGCAGAGCAGGTCGTGTATCACAAGAAACTCTTGTTGCATTGACTAATT

CAGCTGACGCTTAATAATCAATAACGTAGGGGCAATCCTCTAGGGTTGCCCTTATAAATAATATTATGATGTAGGAATTA

CCTACAGTAGCATTCCCGAAAGGGTTAATAGGAGAAACAAATGGCAGATAAAAAAGTCACCCAGCTTACCGATTTAGGTA

ACGCAATAGCAAGTGTAGACCTGTTTCACGTAATAGATGATCCATCAGGCACACCGATAAACAAAAAAGTATCAGCAGCA

AATGTATTTAATAACGTACCAACGTTTTTAGGTCTTGCTCAAGCATCCCAAACATTAACAGCAAATGGAGTTGATCCATT

AGTAGCAGATGTAGAAAGTGCAGTAACAGAAGTTAACGCAACTACTGGAACAGGTGCAGTATCATTAGCAGATGGCTCAA

GCGGTCAAGTTAAAACTGTTATTAATACATCAACAGCAGGTACTAACGTAATAACTATAACACCAACAAATTTAAGAGGT

GGAACAAACATCGCTTTAAATGCGTCAGGTGAAACAGTTACTTTATTATTTAAAAATTCAAATTGGAACGTAATTGCAGG

TTTCGGATACGCAGTTGCATAATATATAATATAAGGAGATAATTATGACTATAAGTATTGGGCAACTTAACGAAGAAAAA

CAAGGTCTACAAGTAGATTTTGATAAGTTGGCAAAAAATATAAAACAAGTTGAAATTGATTTGGGTCAGATGAAAGCAAA

TTTAAATGCAATCAATGGCGCTATTCAACAAGTTAATAAGTTAATAGGAATGACTGGAGATAATCCAGTTAAAACAGAAG

ATGTTAAAAAAGTTTAAGATTTTTAATGAAGATAAAGACTTGGACGATTTTGAGGAAGAAGTAATAGCTGATTGTCCTAA

AGAGGACACAGCAAAAGAAAAGGGAAAAGAAAAAAATGAAAACGTTTAAACAACACATAACAGAAGACGGCAAGATGGTT

GGCACAGCTACATCTAATGCTGTTGAAGATGGTAATTTAGGCGCTCATAACGTTTCCGATCCAGAAGTATTAAATAGAGT

TAATGCTTTTGTAGGTTCTATAGGAGATATGGAATACCTTAAACCACAACAAGCGGTAGATAGTTTGAGAGAAAAACTAA

ACCGAATTGGTTTAACAGTATCACCAGTTAATATGGAAGGAACATCTGGAAAAGTTAGTGCGAAAGTTAGTCAATTTGGT

GGAAGGTTTGGAAAAGATACAGATGGTTCTGATTTAAATGATGATGGTATATCACACAAAAAATCTGGCGGTCTAAATTT

AGAAGTTAATTATGAAACTTTGAAAAACGGAACATCAAAGGTCTACGCTAAATTGGTGTAGGTCAGATGTTTGAGGAAAT

AACCAGAGATAATTGGTTGCTATTTGCTCAGAAAAATTATAGCAATCCTACATTGGAAGATAATGTAGAGTTTTTGGAAG

ATATAAAACGATTTAAATATCTTAAAAGGTTATTTCGTAAGTATAAAACTACAGGTGATGTTAAAATAAGATTAATTATT

AATCACATTGTAGTATTACAAAATGTTTTTGGTGCAGACGTAGCAATAACTTTGTTATTGTTTAAGATAGATAGAGAATA

TTGGTCAGTAATGAAAACGGTATTGAGCTATCTTAAATTACTTTATGAACACGAAATGCCTGATGTAGACGAAGATGAAA

AGATAAAACAAATGTTAAAGGAACTGTAATGGCAAGTAGAGCAATAGATATGTTAATAACTTACCGAGTAGTTAAACTAT

TGATAACACCTTGGGAGAAGCAAGAAGCATTTAAACAAGGTATCATTGATAAGAAAGGTAAAGTATTAAGACCTAATAAG

ACATTGCAAGCAAGTAAAGATAAAAAAGCATATACTTATTTACATAGGTTTGTTTTTAATATGAAAAGATTATTTGCAAA

AGTAGGATTAGGTAGTAGGTTTGGTTCTTTTTTTGGTGCTATGGCAATGATATTGAGAGAAGATAAACAGTTAATGGTAC

ACAAAGACGCAATAGAAGCAGGTGTAGTTTCATATTTAAAAGAAACTAACCAGTATGAAACTATGTTAAATGAAGTAAGA

GATATACCAGACATAGAAGATGAACCAGTAATGACTTGTCTAGGTGTAGGTATATATGAACAAAATAATAAACTAGTATC

GGAGTATGAGTATGCCAAAACATTATAAAGATATGATGGACGAACTCATCAACAAGATTGATGATAAAGAAAAAAAAGAA

GACGCACCAGCAAATGCAGTAGCACACGGTGGTGTAGATATGAATCCAACTGGTAAGAAAAGAGTTATGGGTACTTTAAA

AAGAAAAGTACAAGAGAGTGAAGACAATAACAATGTTGTATTGAAAGGTGTTTATAAAGTGTTAAATAAACTTGAAAATA

AGATTGATGAAATAAGTGGAGTTGTGAAAGAAGAAATTAAAATTGAAACACCTAAAAGAAAAAAAACTATTAAAGAAAAA

GCAAGAGTATGAAAAGTTTTAAAGAATTTATAGGTACAACAGGTGTTAGAATAGGAAACTATTCAAATACACAACCTATA

GCAAGTTTAGGTGATACACCACCTAAAAAAAGAGCAGGTGGAAAAAATGTTAGAGGTGTTGGATTACACGCTGCTTATAC

AACAGCAGTAAATCAAAGACCTTTTTTGTCTGCTGATCCAAAAGTAGAACCAAGAAATAAAAAGAAAGAAAATACTATGG

GCGGTATGGTTCACGTAAGAGGAGCACAACCAACGTCTAGTATAAAAACTAGAAAGAAATAATATGGAACTAATAATAAG

TTTAGCAATGAAGTTTTGGATGTGGACAATAGTAATTTTAGTTATTATAGCAGGATTAATTATCAATTTATTTGATAAGA

AGAAACCTAAATGTCATAACTTTACATATAATAAAATGCCAGTTATGAGAGCGTTGCCAATAAGAACAAAAGGCAAAGGT

TTTTTTAAAGGAATTCTTTTGTGGATACTCACTACAAGAAATTGGGAAATTGCAGAAGATTTTGAATACGAATTAAACGA

TATAAAATATACGATACCAGCTGGATTTAAATTTGACGGTGCAAGTATACCAAAATTCTTGCATACATTTTTATCACCAG

TAGGAGTACTTTTAATGGGTGGTTTGGTACACGATTATGCTTATAAATATCAAACACTATTAAAAATAAATAAGGCAGAT

ACCCTTGGTGTTATATCTCAAAAAAGAGCAGATGAAATCTTTAGAGATATTAATATCGGAGTAAATGGATTCTATCTTAT

GAACTATCTAGCATATTATTCTTTAAGACTAGGTGGTTTTTTGGCTTGGAATAAGCACCGAAAAGTTGGTGCCAAGATAA

AATAAAAAAGGAGAACCAATATGGGTTTTATAACAGGAAGACTAAAAGAACTAACATCACTACACGGTGGAGTATTAATA

GGATTAGGCGTTGTAGTTTTGTTTTTCAGTCCAGTTGCTAAAATTGCTGCTTGGGCGGCAATTGCTTACGGAGCTTGGGC

AGTTCTAAAGAAAGACTAGAAAGTTATGTTTGGTTATTTAAAAATGGCGATGGTAGTTGTGATGATTACAGGACTTGCAG

GTGCAGGTATGTATGTAATGAAGTTACGATCCGATAATGCCATTTTAAAAGCGAATCAAATCAAATTGGAAGAAGCAGTG

AGTTCTCAAAAGGAACTCATTGCTAAGCAACAAGAAGACTTTAAAGAAATTTTAAAAGCAAACAACAAGATGAACGAACT

TGTTTCAGCTCTTAAAAAAGATTTAGATGATTTAGATAAAAGATTTAATAAAAAAGGTCGTGACTTTGGTAAACTCGCCA

TAGAAAAAACAGAAACTATACAAAGAATTATTAACGGTGCTAGTGATAAAGCAATCCGTTGTGTAGAGATAGCAGGTGGT

TCACCACTTACCGAAAAAGAAATTAACGCAACTAAAAAATCAGAAATTAATAGGGAATGTCCAAGCATTGCCAACCCAAA

TTATGTACCGTATAATAATTAGTATCATTGCTGTTCTAATACTTACTGGTTGTTCAATTGGTGAGAAACAACTTAAAATA

TTTAAGTTAGAAGAACCTAGACAGAAATTAGATTTAGTTAAACCTACTATGCCTGAACTTGAAAAGTTGAGGTGGATTAT

AATTACTTCTGACAATGCAGAAGAAGTATTCCAAAAGATGGAAGAACAAGGACTTGATCCAGTACTATTTGGATTGAGTG

ATAAGGACTTTCAATTAATTGCAAAAAACTTTGCTCAAATAAGAGCACACTTGAAACACACTAATGATTTACTTGACCAA

TATAAAGAATATTATGAACCAGATGATAAGAAAAAAGAAAAGGAGAAGGAATAATGGTAATGCAATTTTTACAGCAAAAG

TATGATCCAAAAACAGGAAAATATAAACAAACAACGATAAAAACTAGAGCAACAATAGAAAAGCTAGGTAGTGGTTTATT

AACAAGGACTAAAGATATTAGTAAAAGTACTGTTGATTTAGTTAAGAGAACAATTAATAGAGGAAAATAGATAATGAAAA

TATTATGTATATTATATGACGACCCTAAAGATGGAATGCCAAAATCATATCCAGTCAAAGACTTACCAAAATTAGAAAAG

TATCCAGATGGAATGACACTACCTACACCAAAGGGTATTGATTTTAGACCAGGTGATTTATTAGGATGTGTGTCAGGTGA

ATTAGGATTAAGAAAGTTTTTAGAAGACGCAGGACATACGTTAGTAGTTACTTCTGATAAAGACGCAGATGGTTGCGTTG

CAGATAAAGAACTAGTTGACGCAGATGTAGTCATATCACAACCATTCTGGCCGTACTATTTAAATAGAAAAAGAATAGAG

AGTGCTCCAAATTTAAAGATGGCAATTACAGCAGGTATTGGTTCAGACCACGTAGATTTACAAGCGGCTATGGATCATAA

AGTTGATGTAGTTGAAGTAACTTATTGTAATAGTAGAAGTGTTGCAGAACATATTGTTATGATGATACTAGCACTAGTTA

GAGATTATCATAATCAACATAGAATAGTTAACGAAGGTGGTTGGCATATAGCTGACGCAGTTAAACGTTCTTATGACGTA

GAAGGTATGCATATAGGTACAATTGCTGCTGGACGTATAGGTTATGATATGTTAAGAAAGATGAAACCATTTGATGTACA

CCTACATTACTTTGATAAGCATAGATTGCCAGAAGATAAAGAGAAAGAATTAAATTTAATATATCACAATTCAGTTGAGG

AGTTAGTTGCAGTATGTGATGTGATTAATATAAGTTGTCCTTTACATCCTGAAACTGAACATATGTTTAATGATGATTTA

ATTAGTAAGTGCAAAAAAGGTGCGTACATAATTAATACAGCAAGAGGAAAAATTTGTGATAAGGATGCTGTTGCTAGAGG

ATTAGAGTCAGGACAATTAAGCGGTTATGCAGGTGATGTATGGTTTCCACAACCAGCACCTAATGACCACGTATGGAGAA

CAATGCCTAACCACGGTATGACACCACACACTTCAGGTACTTCACTATCAGCACAAACAAGATATGCTGCTGGTGTAAGA

GAAATACTAGAGTGTTTATTTGATGGAACAGAAATAAGAAATCAATATTTAATTGTTAAAGATGGAGACCTTGCAGGTAT

GGGTGCTCATTCTTATACAAAAGGAACAGCAACAGGCGGTTCAGAAGAGGCAACAAAGTTTAAAAAGTAATGGAAAAATG

TAAAAATTGTGGTAGGGAAGCTCATTGTCCTGAAAAGTTAATGGAGTATATTACTTCTAGTCCAGATGGATTACGAAATC

AAAAGACAGTAACTATTATTTGTAGTAAATGTGATTGTTCTGTTTGTGAGAAACCAAGACCCAATGTAAAAACAGGAGAT

GAAATAGTACAGTAATGGATAGTGAAGCAGTATTAATGTTGAGTAGACTTTGGCCGATGCTAGTAGCATTTGTGTTATTG

ATAGTTACTTTAGCACAATCACACTATAGAATTAAAGTGTTGGAAGAGAAAGTTAAAGTAGCGTTTGAACTTATTAATAA

GTTAACAGACAAAAAATGAAGACAATATATACCTTAGCAGGTGTTGTTGCAGTATTAGGAATAGTTATAGGGTTAAGTTG

TTATTTGATATTAACATTTATTTAATAGAAAAACAATTAACCTTTTCTTTTTTACCTTTTACAGTAGAGATACCTAGTTT

ATAGAATTTAAATCCATCTGGTCTTGCCAATGGAACAAAGTCAATACCTTCAGCAGTATGTTGAGATATTACTATTGTAG

TATCAAAATCTTTACTCTTACCTTCCAATCTACTTGCCAAATTAACAGCGTCACCAATAACAGAATAATCAAAACGTTGT

TCAGACCCCATATTACCTACAAGACACTCACCAGTATTGATACCTATACCAATCTTTAAAGGTGGGTCAAACTCTCCACT

CTTATTCATTTCTTCAACTGTTTTTTTCATTTGGGATGCAGATAGTACTGCTAGTCTTTGATGATTTGGATTGTCTAGGG

GTGCGTTCCAAAATGCCATTATACAATCACCCATATACTTATCAATAGTACCACCATTTTTTAATATTATATCAGTCATT

ACAGTCAAGAACTTATTAACATACTTGGTAAGTTTTTCTGGATTACCTTTCATAGATTCCGATATAGGAGTAAAGCCACG

TATGTCTGAAAACAAAAACGTCATTACTTTTCTTTCACCACCAAGTTTTAATAGAGATGGATTCTTTTGTAGTTTCTTAA

CCATATCTGGAGATAGATAGTGTTCAAATTGTTTCTTAATTTGTAGTCTTAATTTATTTTCTTTTGAATAGTTATTGTAT

GTTAACTGACCCCATATTACAGACCCTATTACAAGTGGACTGAACCAATCAGTTATGATTAAGTGTTGCGTCCATAGATA

AGAACTTGCAAGTGTTAAATCAAATAAGTATCCACCAAACCATATCGCAGACCACATTACACCACATCTAGGTATAACTA

TGAGAAAGAATAGAGCACCTAATAGTATAAAAGCAAATTCACTATATGGTAACCAATAAGGTCTACTAATAAAATTACCT

GACAATAAAGTTTCTGTACTGATTGCCATAATTTCGTGTGTGTTTTTTAATCCATTAGGAGTAGTAACAAATGTTGAACC

TTGGAATGTTGTACCTATGAAAACTATTTTACCTTTTACAGATGACCAATCTTTATCGGTGTAATCTATTCTAGGAATAT

TATGTCTGAAATCAATCCAAATAGTATCTTCTGCTAAAGTTTTTGCATTAATAACTTTTAAGATAACTTCAGGTATGGAT

TTATCAAGAGGTAGTTTTCTTATAGTGCCATCTATATCAACTGGTGCAGATACATTACCTATTGCTATTGCTTTACGTGA

GATACTAATAAGATTTTTTACAGTATCGGTTTCAGTAAGTATGATTGGATATTTTGAAATCATTTTCAAAAACATTTCAT

CACCACCTAATCTATCTTTATGTGGGAAGACTACTTGCAGAACTACAAGAGCGGCACCATTTTTATATGCATTGATTATG

ACACGACCAAGTAAATCTCTCTTCCACGGCCATTGCCCATTCTTTTCTAATGCTTTGTCTGATATGTCTACTAGTACTAA

ACTCTTGGATTCATAATTCTTACCAAATGTTTGGTAGAAATCAAAGGTGTTTAGTTTTGCTGACTGTAGCAAGATAGGAT

TTGATATATATATTCCAAGTAATATAATCAACGTCAAATATACTGCCCACGTTTTCGTCAATATTTTAATCATACGTACT

ATTTAGTACAGATATCTTATAAATATATACATCAAAGAGAGAAGGAAAATGCGAATAATCATTACAATATTAGCAGTATT

ATCATTATATTCTATAGCAAATGCAAGTGAATTAACATTTAAATTTGATAGTCCATCCTTTAGTGGGGAAGGAAAATCAT

CACACTATTTAACTGTAGAAAATATAGAGAAGACTCGTAAAGACGCTATAAAAGCAGCTGAAAAAGCAGCGGCTGATAAA

GTCAAAGAGGATGCTAAAAATACAGCAGTAGCAAAATTCAAAGCAAATTTAGAATCAAGGTTCTATACAGCACTAGCAAA

ACAAATTACAACAAATATATTTGGTGCTGATGGTCTACAACAAGATACAGGTTCATTTACATCACCAATTGGTGGAGAAC

TTGTTGAGTGGACAACACCTTCAGGAACAGGTAACGTAGTAGTGACTGTTACTGAAACGGATGGAACAGTAACGACATTT

ACTATGCCGAAGGAAGATTAATGTTTAGAAGTATAGCAATATTTTTGTTAACATTATTGTTAGTTAGTTGTGCAGGTAAG

CCAGACTTTGATGTTAGAAAACAAATACCAGCAGTTAAAAATTTAACTACAATAGAATCACCTGTCGGTGAACCTGTTGT

AATTGCTGTGTATGATTTTATGGATTTAACTGGTCAAAAGAAACCAGGTGGCAATTATGCAACAATGAGTACGGCAGTAA

CTCAAGGTGCATATCAAATATTAATTAAAGCATTACAAGACGCAGGCGATGGCAAATGGTTTAGAGTAGTAGAACGAGCA

AGTTTGCCAAGTCTATTACAAGAACGAAAATTAATACGTTCTACAAGACAACAAGTTGATGGTGATAAAGCACAATCTTT

ACCTCCACTATTGTTTGCAGGTGCATATCTTACAGGTGGAATTATAGGTTATGATAGTGATGTAAAGACAGGCGGTTATG

GTGCAAGAATTTTAGGCATTCAAGCACATAAACAATGGCGACAAGATGTAGTTACTATTATTTTAAGATTAGTTAATGTA

CAAACTGGTGAAGTAGTAATATCAATAGTAGTTGAGAAAACTATAATTTCAGGTGAAACTGGTGGAGATATATTCAAATA

CTTTGACGCTGATACATTGTTATTAGAGTTAGAGGTTGGTGTTGCAAGAAATGAACCTGTTACCTTTGCAGTAAGAAAAG

CAATAGAAAAAGGTATAGTAGAATTGATAAAAGAAGGTGACGAAAAAGGCTTATGGGAAATTGAAAAGCCAGTTATTGTT

GAAGAGTCAGACGCAGAAGTCATTACAGAAGAAAAGGTTGAAGTAGTTATAGAACAACAAGAACCTAATGTAGAGAAAAC

ATACGAAGATTATCTATTAGAAAAAGAAAACCAAAAAGAAGAACGTAAGAAAAAGATTCAAGACGAGTTAAAAGAAAAGG

AGAACACAAATGAAGAAAATGATAATGGCTCTAGTGATTCTTCTAGCGACAACGATTAATTGTCTAGCAGGAAATTCAGT

ATATATTTGGCAAGTTAACCAAGATGATGATGGTTCCATCTACATCAAACAAGATGGTACTGGTAATATGGTTGGGTTAA

GCACATCATATCCTTTTTTAGTCAACGGACCAAATCTTACTCTTATAATTAAACAAATAGGTGACAATAATGTTGCTAAA

GATAGCAACCATAGAGCATTTATGGGTTCTAATATGACCTTTGATTATTATGCTGTAGGTAGTTCCAATGTATTAAGATT

AGATTTAGATGATGTAGGCGCAGATGGACACTATTATGACATTGATATTACAGGTTCATCAAACATAGTAGAGATAGATA

CAAATACAAGTGATGATGTACAGGATACACACCTTGATTTAGATATTGTAGGAGACTCAAATGATTTTTGGATGAATTCT

CACGGTGATTCACACTTCTTATATGTATTGATTAATGGAGATTCAAATGATGTAGAATTTTATAGTAACAGAGCAGAATC

AAAAGGTATGGTTGGTTCTGACAAGGCAAATATAATGATAGGTCCAGATGTTGCTTCTCACGGAATCTTTGCAGATGATT

CAGGTAGTGAAGGTGCAACAATAGATTTTTACATTATAGGAAATTCTAATAGAGTACACACAGGAATTTGGGGCGAAAGT

AACTACCAAGTTCACGATATCATAGGTGATTCAAATATTTTAGATGTTCACTCCTGTTGCACAGGTAGTCACGTAAGAAT

GATACAAAGAGGTGATGACAATTGGATGAAAACGGTTACTGTTGGAAACGATAATACATTTACTTACTATGCAAATGGTG

ATGATAATGTAGCAAAAGTTTATATCTATACAGATGACGCAATTGTTAACCTTAAACAACTTAATGATGACAACTATGCT

TATCTTTATGTATCAGGTGATTCAATATACGACTATACTTTACACGTGAATCAAGACGGTAATGATAGTTGTACCTATTC

ATATAACAGAAACAATCAAACAGCAGATTATACTAATACGATAACTGGCGGGTGTTAAATGAAAGGGTTTGTCGCTTTCT

TTTTAATATTTGGTACAATATGTTTATCAGGTGGCATAATCTGTACACTAATCCACAATTACTATCCAGAAAAATGGAAA

AGAAAAGAAAAGAAGAAGAAATTTAAATGAAACTATTTCACAACACTTGGGAAAAGTGGGCAATAATAATTTGTGTTACT

GTATTATTAATATTAGGCATATCATCAGCGAAAGCAAATGAATTATTAATGGCACACAATCCTATGTGTGGTATATGCCA

AAACTTTTTACAAGAGGTAGGAGTTGACTATGAGTATGAAGATTTACCTTTAGTTATTATTAATTTATATAATCAACCTA

ACTGGTTTAAAGAAGCATATGCTGAAGGAAGAATTAAACCTATTAGAGGAACACCAACATTTATAATATGGAATGGTAGA

AAAGAATTAACAAGAATAATAGGGTATGCAGATAAACAATCGTTTTATAATGATTTAGATGAGGTGTTTCAGAAATGAAA

AAACTTTTATTCATAGTATCGTTTCTGATACTATTTACTACACAAGCATTAGCAGTTACTATTGGTTCTGTTTTTGATAA

AATGGGTCAAACTTGGAACGAAAGAGATGGACGTACCGAAGAAGTAATTAAAGGTTACCTATTAGAAATGAACGATTTTC

TCCAAACAGGAGAAGATGGTGGAATGATTTTACATTATAAAGATGATACTAAATTTACAATGGGTCCGAATACAGAATTA

ATCATTGATGAATTTGCTTTTGATACGTCTGTTATACCAATTGAAATTGCAATGAACATATCAATTAATGTTGGTACATT

TACATATGAATCAGGAGATGTAAAAAAATTAGGTGGTGAGGTTGAATTAGTTACACCAACAGCAACTATAACTATGCAAG

GTACTGCCTTTTCAGGTACAGTAGATACTTCAGGTCAAACAACAATTACATTACTTCCAGATAGTTCTGGTGATGTAGGA

CAAGTAACCGTAACCAATGACGCAGGTTCTCAAACATTAACTAACGTATACACTTCTGTAACTGTAGTTGGTTCAGATGT

TATGATTAAAACTCCATCAGCATTGGATAACAATCAGAAAAAGAATTTGTTTGATATTGATAGTATTGATGATGATATAA

AAAAGAAACACGACCAACAATTTGATAGAGCAGAAAACAATGAACAATTACAAAAAATGGAAGAAGCTATTATTTCTGAA

GAGAGTTCAATTACAGAAACATATGATACATATGACACTACTTCCGATTTAGGTACTTCTGGAAGTGATGATAGTTATAG

TTATGAAGAATCAGCAGTAGACCAAGCAGGAAAAGAATCGGAAGTTGATACTTCTTATTATGATGAATGGGAAGAAGATT

TAAAAGATTGGGGATACATTGATGAAGATAATCAAATATCAGTATGGGATGCTACTGGTGAACAGAAAATGGATTGGGAT

ACAGCTAAAACTATGTACGCTGAAATGGATCAAGCATACTTTGACGCAATAGGTTGCTCAGATTGTACTTGGGATACTAT

TAATTGGGAAGAGGTTGATTGGGATAAAGTTGATTGGGATGCTTATTATGATGAGTATAATGATACGTTAGAAAAATATG

GATTAACTTCTTATGATGTAAAGGAAGAAGATGTAACTGAAACAGTTGAAGAAGAAGCAACATCAACAGTTGTTGGATAC

ACTTGGGAAGATTTTGATTTATCAAATGAATATTATAATAATGCAGATTACATAGCGGCAGGTGGACCACCAACGTTAAC

AATAGAAAACTATTGTGATTATAATGGTTGGGATACATCTTGGTGTAACCAGAGTTATATAGATTATTTAAACGCTTGGT

ACGAAGACGATTGGACATTGTTTAAAGATTATGATAGTTGGGAAAAAGGAGCTAGAAAATTATTTAAAAAATGGTATGGT

TGGTGTGGTACTTGGGACAAACCAGAGTGGTGTTCTGGACAACCTAAACCTTGGAAGATGGCAAGTTTAAAAGACAAATA

TGTTTCTGAATGGACCAATTCAGATTGGCAAAAATTTTATGACAATACATCTAGTTGGTGGTACACAGGTTCATATGATG

ATACAGGAGATGATTCTACAACATTGGAAGATGAATATAGTTATGAGGATGATTATGACATAGACGCTGAATTAGAATTA

TGGTTAGCAGATATAGATAATGAAAATGATTGTGTTAATTGGGGATACTATTGGGACAAAGCAAATGCTTCTTGTGGAAC

AGAATGGGTTGACAATTCAAAATCAGAAACGACAACAACAACTAGTGGTGAAATATTAAATTACACCACAGGAGATATTA

CTCAAACTACTACAACAACAGAAGGTGATGTAAAAACTTCTGAAACATTAACAGGAAGATATACAACAGGTAGCAACACT

TATGACGCTGATGTTGATACGTCTGTTAGCGGCTACTCAATTATAAATAGATATAATGACAACCACCGTGCTTATTTAAA

AATTGAAACTGCTGACGAGGCAGACGTTCAAATTTTACAAGATAAAGAAGCACAACACATTGATATTGGCAGCAGTTCCA

GCCAAGAAAATATTACAATCATACAAACGGATTAATGAGTAGGTTAATAGCAATAAAGGAATTAATAAAAGGATTTTGGT

GTATTTTTATTATGGGCAGAAATCCAGCACAATTTAAACATTGGTACACCGAATGGTGGTTGCCAACACAATGGAGAAAC

GTATAATGGATTGGGGAACAATTAATTTTTGGTTAATAATGGGTCTATTACTCTATATGAATTGGAGTATTTACAAGTGG

ATAGATAGAGATTTCTAATGGACGAAAATAACGGAATTAAAACAAAAGTAGATATTGCTAAACTCAAAAAAGACGTTGAA

GAATTTGATAGAATTCACAACCGTTTAGACACAGCAATAGACAAATTAACAGATGTATCAACATCTATTAAGTCTATGTT

GGCAGTCCATTCAGAAAAAATCAATAGACAAGAACAAATTGACGAAGTTATATTTGAAAAGTTAAAAGAACGTGCAGGTG

AAATAGACAATATACGTAGAGATTTAACTAGAGAAATAGAACAAACTGAAAGACGTTTATTACTAGAGATTAAAACAGTT

TACAATGCTATTGGTGTTAGAGTTGGTGTATTAGAAAAATATAGATGGATTATATTAGGTGCTGCTATAGTAATAGGATG

GATAGTAGCAGGTAATTTTGAGCATATTTTGAAGATGCTGAGCTGATAGAAAACTCTTACCTTTTACCCGAGGAAAATAG

CCTGGGTATTTTTTTGTCACCAGGTTTTCGTGTACAGACTTGACTTTTTTTGTGATATGGTGTATATTATGAGATAGTGT

TATGTCAAGTTATATAGATTTAAAGTATATTAATGCCATTTCTTCAGCATTAAGCCAGTTTAAAAAGAAGACAGATTATC

TTTTCAATTTTAGGTGTCCACATTGTGGAGACTCGCAGAAGAGTAAAACAAAAGCAAGGGCATATCTTTATAGAGTAAAA

AATGATATGTTCTTCAAATGCCACAATTGTGGTATGGGTCAGAATTTAGCAAATTTCATTAAATTCTTGGATCCCAAAAA

ATACGGAGAATACTTATTAGAGAGATACAAAGGATCGGCACCATCCACGCCCCAGCCGAAATTTGACTTTAAACCAACAA

AATTTAAAGAAACAAATTTATTAGATTCTTGTATTAAAGTAAGTACATTAAAAGACGGACATCCTGTAAAGGATTACGTA

AAAAAGAGATTGATACCTCCACAATATTATGAGATAATTTATTTTGTTGACAAATTTCACAATTTTGCTAATAAAGTGAA

ACCAGGGACTTTTAAAGAACGTTATGAACACCCTAGATTAATAATTCCTTTCTTTGACGTAACTGGTACGTTGTTTGCAT

TCCAAGGCAGAGCATTTGGAAAAGAACAACCAAAATATATTACTATTAAACTTGATGAAACAAAACAAAAAGTATATGGA

CTTGAACGTGTAAATTTTCAAAAACATATTTACATAGTTGAAGGTCCACTTGATAGTTTGTTTTTAGATAATTGTTTAGC

AGCAGGTGGTGCTGACTTAACATTGAGAGTGTCAAGTGACCAAGTTACATATATATTTGATAACGAACCTCGTAATAAAG

AAATCATAAAAAGGATGTACGCTGTAATTGAAAAAAATTATAACGTAGTAGTCTGGCCAAATGACGTACAACTTAAAGAT

GTAAATGAAATGATAATGAATGGAATGAAAATAAGCGAGTTGAGAGATATCATAAGTAATAATACCTTTAGCAAATTAGA

AGCGTTAACAAAATTAAACTATTATAAGAAATGTTAGGAGTGTGTAAATAAAAATGGTGAATGAAAATATTAGTGTGGTG

AAACGAAATGGTAGAGGTAAGGAATCTCTAAACATTGAGAAGATACATCAAATGGTAGAATATGCGTGTGAAGATATAAC

GCAAGTTTCTGCTTCTTCTGTAGAAATGAATAGTGGTCTACAATTTTATGATGGCATATCAACAAACGAAATTCAACAAA

TCTTAATTAAATCAGCAAACGATTTAATCACTTTAGAAAATCCAAATTATCAATATGTTGCCGCTAGACTATTACTCTAT

AGTTTAAGAAAACAACTATTTCATAAAATGTGGGATCATCCACATATTTTTCAACACGTACAAAATGGTATAGACAAAGG

CGTCTATGATAAAGAAATTTTAAATTGGTATGACAAAAAAGATTTTGATAGAATGGAAAATTGGTTAAATCACGAAAGAG

ATTATACTTTTACATATGCAGGTTTAAGACAAGTCATAGACAAGTATCTAGTGCAAGATAGAAGTACAGGACAAATTTAT

GAAACGCCACAATTTATGTATATGATGATATCTGCTACATTATTTTCACAATACCCAAAAAACAAAAGGATGAGTTATGT

TAAAAAGTATTATGACGCTATTTCACGTTTTAAAATTAATATTCCAACACCTGTTATGGCTGGTGTACGAACTCCTATTA

GGCAGTATGCGAGTTGTGTGTTGGTGGATGTTGATGATACTTTGCCTAGTATCTTTTCTAGTGATATGGCCATTGGCAAG

TATGTTGCCCAAAGAGCAGGTATTGGTATCAATGCAGGAAGAATTAGAGGAATCAACTCACGTATTAGAGGTGGCGAAGT

ACAACATACTGGCGTTATTCCTTTTCTTAAAAAATTTGAAGCAACTGTTAAGTGTTGTACTCAAAATGGGGTACGAGGAG

GTTCGGCAACCGTTCACTTTCCTATTTGGCACAAAGAAATAGAAGATATTATTGTATTAAAAAATAATAAAGGTAGCGAA

GATAATAGAGTAAGAAAATTAGACTACTCTATACAGTTATCTAAATTATTTTATGAAAGATTTATTAATGATGAAGAGAT

AACATTATTTTCACCACACGAAGTACCAGAACTTTATGAAGCGTGGGGAACAAAAGAATTTGATGAAATATATAAAACGG

CAGAAAGAAAAACAAGTGTATGGAAAACTAAACTTAAAGCACAGGACTTGTTTATGGCAATTTTAAAAGAAAGAGCAGAA

ACAGGTCGTATTTACATTATGAATATAGACCATTGTAATACTCACTCCTCTTTTAAAGATAGAATTTATATGTCTAACTT

ATGTCAAGAAATAACATTACCAACAACACCTATAAGTCATATAGATGGTAATGGAGAAATTGCATTGTGTATATTATCAG

CAATTAATGTAGGACTTTTAAAAGATTTAGATGAATTAGAATCCTTATGCGATTTAGCAGTAAGGTCATTGGACGAAGTT

ATAGACCATCAAAGATATCCAGTTAAAGCGGCAGAAATTTCTACAAGAAATAGAAGAAGTTTAGGAATTGGATATATTGG

TCTTGCTCATTACTTAGCGACACTAGGAGTTGGTTATGAAACTAAAACTGCTTGTAAAGAAGTAGACAAGTTATCAGAAG

CATTCCAATATTATCTATTAAGAGCAAGTAATGAATTAGCAAAAGAAAAGGGCAAATGTGAATCCTTTTCTAAAACAAAG

TATTCAGACGGTATCTTACCAATAGACACCTATAAAAAAGAAGTTGATGAGATTGTATCTAGGAAACTATCTTATAAATG

GGAAGACTTGAGGAAAGATATTAAGGAATTTGGGTTAAGACATAGCACACTCACAGCTCAAATGCCTTCTGAAAGCTCTA

GCGTGGTTTGTAATGCCACAAACGGCATTGAACCACCTAGGGACTATATTTCAGTTAAGAAAAGTAAGAAAGGTACTTTA

AAACAAGTTGTACCTGATTATAAAAAATTGAAAAATAATTATACATTGTTATGGGATATGAAATCTAACGAAGGATATAT

AAACATAGTAGCAGTAATGCAGAAATACTTTGACCAATCAATTAGTGGTAATTGGTCGTATAATCCTGAAAATTATGATG

AAGGAGAAATACCTTTATCAATAATGGCGGAAGATTTATTAACAACTTATAAATTAGGATGGAAGACTTCCTATTATCAG

AATACATATGATAGTAAAAGAGATATAGAGGAACCTGTACACCCTATTGGTTGGAAAGATGATGTGCCAGAAACAAAAAC

TATAATGGAGAAAAAAGACGAAGAAGAATGTGAAACCTGCGTAATTTAAAGGAACTTTATGGCATTTTTATGTGCAAATG

TACCACATACGGAAGTACTAGTTAAAAAACAATACCTTTATGATTTAGAAAAAGGTCACGGAGAGTTTGAACCAGGTATC

TGGTGTACTGTTAAAAGTATTCAAGGTAGAGCATTATATTTTGAAACTTATTTGTATGAAACAGGAGCACTATATGATAA

GTTGCCTATAAATGCTTTTGTATGGAAAGAAACAAAAGAAGATATAGCATTACCAGAATTACAGTTATGGGATTGTTTTG

ATTATGATATTACTATTATACAGAAACAATTAGTAAGTGGCAATAGATGTACGTTTCTATCACCAAGTAAGAAATTATAT

GAAGGCAATTATATGTTTAGTATAGATAGTTGTTGTGCAACGAATAAAGAAAATAATGTAGGGTATAGTGAAACTCCTTC

TCAACATAAATCATTTAACATAATAAAGTTAGATAATGGGCATTTTGCTGCTCAACCTAATAATAGAGTTTTGTTTTATG

ATAAATCATTAACACCTAGTAAACCAAAAAGACCAGATTATAAAGTATCTACTAGAGAGTATAGTGTAGATAATATAAGT

AAATGGACAGCAGGTGATAGTGATGACCACCATTATGAATTAACAGAATCAGAAAGAATGCAAGAACAATTAGAACCGAT

AAATGACTAAAAGTGTATTTAATATAGATAAAAAATTAGATTATACCAAACAACCTATGTTTTTTGGTAAAGATTTACAG

GTACAAAGATATGATGTATTAAAATATCCTATCTTTAATAAACTGTTTCAACAACAGTTAGGTTATTTTTGGAGACCAGA

AGAAGTATCTTTACAAAAAGACATTGCAGATTATAGAGAATTAAATGAACAAAGTAAATTTATATTTACTTCTAATATAA

AATATCAAACAATGATGGATAGTGTGCAAGGTAGAGGTCCTGCGTTGGCATTATTACCTTTTGTTTCAATACCTGAATTA

GAAAGTTGTGTCATTGCTTGGGACTTCTTTGAAAACATACACTCACACTCATATACATACATTATAAAGAATTTGTATCC

TAATCCTAGTGAAATCTTTGATACTATAGTTACAGATGAGAAGATTGAAAATAGGGCAACTAGTATAACACAAACATATG

ATGATTTAATTAATTTAGGTTATAAATGGCAATTAACACCAGATAGTGTTGATATGTATGAGTTGAAAAAGAAACTATAT

TTAACATTGATGACTGTTAATATATTAGAAGGTTTAAGATTTTATGTTTCTTTTGCTTGTTCGTTTGCATTTGGAGAATT

AAAGATGTTAGAAGGTTCTGCTAAAATACTTTCTTTAATTGCAAGGGATGAAAGTTTGCATTTATCAATATCACAAAGAA

TAATTAATAACTATCGTGATAATGAAAATGATAAGATTATGAATAAGGTAATGAAAGATACACAAATAGAAGTTTATAAA

ATGTATGAACGAGCAGTTAATCAAGAAAAACGTTGGGCAACTTATTTGTTTTCAAAAGGTTCTATGATAGGACTTTCAGA

AAAATTATTACATCAATATGTAGAGTATATGGCAAATAGACGTATGAGAGCAATTGGATTAGACCCACAATATGACCAAA

AGACAAATCCATTACCTTGGGTTGACCATTGGTTAAATAGTAGGTCATTACAAAATGCACCACAGGAGACCGAAATTGAA

AGTTATGTTATAGGTGGAATTAAACAAGACGTACAGAAGGATCAATTCAAGAAATTTAAATTGTAAAAGTATATAATGGA

TCTAAATGATGATATAAAGAAATTTGAGACCACCTGTGGAAATTGTAAGACTAAATTTACAATTAAATATGATGATGAAG

AAACAGATATGAAACCTATGTCGTGTCCATTTTGTAGTTATGAACTGGATGATGAAGAAGATGAAATTGGAGAAGAAGAT

GAAACAAGTTGGGATTGATTATAGTTTAACAAGTCCTGCAATATGTGTAACAGAAGACTTTACTTTTGAGCATAGTCGCT

TTTATTTCCTTACAAATAAAAAGAAACATTTAGGTGTATTTGGAAATATAAATGGTTCTGAACATCAACCGTGGACAGAC

CCTATACAAAGATTTACTCAAATTTCTGATTGGGTTTTAAAAGTTTTACGTTTATATCAACCTGGTGGGATTACAATAGC

AATAGAAAACTATTCTTATGGTTCTAAAGGTCAAGCACTATTTCAAATAGCAGAAAATTGTGGTATACTTAAATATAGAT

TATTAGAACAAAAATGGAAATATAATGTTATTGTACCAAGTGTTGTTAAGAAATTTGCTACAGGTAAAGGTAATGCAGAT

AAAGAAATGATGTACGAACAATTTTGTAAAGATACAAAAACAGATTTAAAGAAACTATTAGACACAGCGAAAGCAGGCAA

TCCAGTATCAGACATAGTTGATAGTTGGTATATAGCAAAGGCAAATTATGGGCGACTTTAAAATATTAATATTAGCATAT

CTAATTGGTCATAGTCCAATAGAAACACAACAAACTTTCCAAATGGAAGGTTGGTATAAAAATATGGAAGAATGTAAAAA

AGAATTACTTTTACAAAAACCAGATGGAAGATATGAAGTGATGAACGAGTTTATTATAGACGGAGAATTTAAATGGGATT

GGTTAGTTGCAGGTTGTAAAAGTGATACAACTGGAGAAGAATTCCAACTTTGGCCGACTTATCCTAAAGGTAAACCAAAA

GAGTTAGAAGGCATTGAATTTGATGTTTTTGAATTACAAGTATGAAATTATTAAAAGCAAAAAAAAGAGTTACTTGTAAA

GATGAACCTTTAGTAGGTGTAACTCCAAGAGTAGTAGAAGTACCATTAGAAAATTTAATGTTAACTGCTGATAATGATTG

GATGATGAAAAGATATCCTAGATTTAAGAAAAGTATAGATAGGTTAGGTATGATGTATCCAATCATATATACTAATATGA

AGTATTATTGGTTAGTAGAAAAAAGATGGCCGAAGGACGCATATTCAGGAATTCCTATACCTGGTATTGCAGTACATACA

GGCAACAAGAGAGTGTATTGGGCAAAAGAAAATGGATATACACATATTGAAGGATATTATGTTAAAACTAAAGATGAACA

AGCAGCAATAGTTAGACGAACATTTATGGCACCGAGTAGTTATGGACAAAACTAAACCTAATTTTAATTCACGTTTAAGA

GATATGCCAACATCTACATTAGATGAATTTGCTAAGAATAATATGATGGTGTATAATCACGCAATGGACGTGGAAGATTG

TAAAATGATTGTTAATAAGTTTGAACAGATAGCCAACTACAATAAATCTAGTGTAGACGCATTTAAAACTGGTCATAAAG

AATTTACTGAAATTGATATAGACAAACCTGATAACCTTCTCTTTTGGAAAGAACCTAGAGATAAATTTCTTCATATGTTG

AAATTGTATAAAGAAAGATATATGATGAATTTAAATATAAAAAATGAACATTTTCCACCAATAATTGATAGGGAAAACAT

AAGAATAAAAAAATATTTACCTAATGATAAAGATGAATTTAAAGAACACGTTGATGTACTCCGTTCTAGGGGTCTTTCAG

CAAAACGTTTTTTAGTTTTTATTTTATATCTTAATGATGTTGAAAAAGGCGGTGAAACACATTTACCAAAACAGAATATA

ATGATTTCACCAAGAGCAACAAGATTGTTAGTGTTTCCACCTTTTTGGACTCATCCACACGCTGGATTAAAACCAATTAG

TGGACCAAAATATGTTATGATGTCTTATTTACATTATGGAGATAAAGATGATAAAATGGGACCAAGCTAATGTATAAACC

ATTACCAGACGGATTAATAATTAAAAAATCTTCTATAGAAGGTCAAGGATTATTTACAACAAAGTTTATTGAGAAAGATG

TAAAGTTAGGTTTATGTCATATTGTTGTTAATGATGAACTTATAAGAACACCTTTAGGTGGTCATATTAATCATAGTGAT

AAACCAAACTGTATAAAGGTGAGAGGTGAATTAGGACTTAAAGATGTTGAAAATTATAATAAGTATTTTTTATATACTGA

ACGACCTATAAAAGCGTGGGAAGAATTAACAGTTAAATATACTTTTTATAATGTGAAAAATGAATAATGAAAAAGATAGT

AGCTTTTGGATCGGGTAATGCATTGGCAGCAATATGTGGAGTTATTAATTCTAATCCACAATCTGATATGTCAGTACATA

GAGTTGTTGAAGTAAAAGATAAACCAATATCTTTAGAAGATTTATATTGGATAAAAAATGCTGACGCTTATCTAGTTGAT

GGTACGTGGGGTAGTACAAATCCAGCAAGACAATGGAAACCAGATAAAATTTATGCTGAAGATAGTAAACTAGCTCACGG

AAGACCAAGCACAGAAGTTAGAAGTGCAAGAATGGAATTTATAAATGTCTTTGTTCAAGAACTAGCAAAGATGTATAATA

AAAAAGTTATTGTAACTGAAAGTGCTACATTATCAAGAATAAAATGTAATTACATAGATTCTTGGTATAAAAATACAGGT

CCAAGATATTATCGTATGGGGTTAGGTCATTGGACATATGGAAGAACTAAATGGTGTAAAGTTGATAATTCAAACCCATC

AAGATTACATACTATGATTGAAAAAACTGAAAAGAAAAATAAAATAAAGTTGCAGAATATAGAACCACATCAATGGAAAA

ATAATAAAGATGGTGCAGTTTTAATTATACCTGGTTTAGAATATGACCCTACATCTTCTGTTTCTGTACCAGAATTTATT

AAAACTAGTGTTGAAAGAGTTAGACTAGCAACAAATAGAAAAATTATAGTTAAACCACACCCATTAAGTAAAATTGTAGT

TAAAGATTTGGTGAAAGATGTTGAAGTTTTACCAAGAGAGACCAAATTACGAACTATTGTAGATAGAGTTTATTGTGGTG

TGTTAGGAGAAAGTACAAGTATTTTTGAACTTATTAATTTAGGAATACCTTGTATTACTTCAAAATGGAATTTTGGAATT

AAATTAAACAATACTAGTATTGATAGGATAGAGAAAATATATTATGCTACTCCACTAGAAGTTTTAAATTGGTATAAAAT

GGTATCTCATACAGAATTTGATATGGAAGAATTTAATTCACACTTGATTATACCTTATATAAAGGAGTTATTACAAAGAT

AATTATGTGTGCGATACACGGAATATTATGGTCATCAACTAGTTTGATAAAAGAAATGTTGTGCGAAGCACATCATAGAG

GACCTGATGGTAATGGACATTGGCAAGATGAAGATATTACTTTAGGTCATAATTTATTATCAATCATAGACACTACAGAA

AATTCAAAACAACCTTGGATTCATAACGATAGGGTGTTAGTATATAATGGTGAGATATACAATTACAAAGAACTAGGTAA

AGAATTTAAACTTAAAACTAATACAGATACGGAAGTTTTAATGTTAGGTTTAGAAAGATATGGCATATCATTTATAGAAA

AATTAGATGGAATGTTTGCATTTGCTTGCTATAATAAGAAGACAAAAGAATTAATTATTGCCAGAGATAGTAATGGAACT

AAACCTCTTTATTATGGTTATATAAATGGTAAGTTTGCTTTTTCATCCGAGATTAAAAGTTTATTAACATTAGGATTTGA

AAGAAAAGTAGATAAAGAGGCATTCAAACAGTATTATAAACAAGGATATAATTCTGGTTATTTGACATTGTTTAAAGGTA

TTAAAAAATTAGTACCAGGTGAAGTTGTTAAGATGAACGTAGTAACTAGTGCTAAAATTTCTACTAATATTAATAATAGA

CAAATAGGGTTAAGAAATGTTAAAAATGTAGGTAAAATTAAAGAAGAAGTTAGAAATAGATTACACCAGGCAGTAAAAGA

AACCTTAATGGGTCGTAGAGAAATTGGATTGTTTTTAAGTGGTGGTATTGATAGTACGGCTATTTGTTATGAAATGTCCC

AATTAGGGAAAACAAAACCAAAAACATTTAGTTCAAAGTTTGAAACTTATGATTGGAAATCAAGGTCTAACGAAGATTGT

ATATTGGCAAAAGAAATGTCAGCAACATATGGTGGTGAACATAGAGAACCATATATTAATCAACAATATTTTATAGATAA

TTGGGAAAATACAGCGTTAGCTTTAGAAGAACCTAGACAAAGTAAAAGTTTGCCTGTATATTATGGTGTTAATGAAAATA

TAAAAAATCAAGGCATAACGGTAACTTTAAGTGGTGATGGTGGAGATGAATTATTTGCTGGTTATAAACATCATAGACAA

CCAGATTGGGCAACGAAGTTAAAAGCATTATGTGCTAATCATAGAGAATTAAAAAATAAAGAACTATGGGCAACACACGA

AGAACAATTAGAGTATCTTGATAGTTGGTTTCCAAAAGGTGGTTTGCAAGGTGATGAAAGAAATGATTTTATGTTCATTG

AGTGTTTAAATACATTATCAGAAGATTTTTTAATGAGAAATGATAAGTTAGGTATGAGATTTAGTTTAGAAGGAAGATTT

CCTTTTATGAATAAAACTTTTAGAGATTATATACGAAGTATCCCTAGTGAACATAAAATCAACAAGAATTTTATGGAAGA

TAATTGGGCATATTATAATAAACCACTATTAAAGACAGCATATTATAAAAGATTACCATTAAAAATATTAGAGAGAGAGA

AAACTGGTTGGAGATTTCCTACAGATGAATTTATTGTAGGTAGACTTAGCAAACGAGCTGATGATAGAAATGTATTAAAA

GATTATATTAGGAATGTATTATCAAATAAAGAAATGCAAGAAATATTTGAATATAACCAGTCCGAAATAGATGATAAATA

TATGTGTAATAAAAGAGAAAGTTGGTCAAAAGGTTTAAATAAAGCTGGAGAAAAGAAAATATTACCTAATATTGGTCAAC

GCTCACAAAAAGAACTGTTTACTATAATGGCATTTGCGATTTGGTATAAAGTATTTAAGATGAATATATAGGAAAATTAT

GAAATATCAATTAGCTAGTGATACTTGGGATCATAAAGAGTTACACGCAATACAAGAAGTCATAAAAAGTGGGCGATATA

CAATGGGTCCTTACGTTAAGAAGTTTGAGCAAGAGTTTGCCAAATATTTTAGATGTAATGACGCAGTTATGGTTAATAGT

GGTTCAACTGCTAATCTATTAATGATAGCATTGTTAAAATTAAAATATAAAAAAGGTGGTAATATAATTGTACCTGCTGT

TTCTTGGTCAACAACATACTTTCCATTACAACAATACGGTTTCAAATTAAATTTTGTAGATGTAGATAGAGAAACTTTAA

ATATAGACCCTAATAAAGTTAGAGAAGCAATTAATGACGATACTTGCGCTATATTTGCAGTTAATCTTTTAGGTAACTCC

TGTGACCATTATTCATTAATGCATATTGCTAGAGAACACAACCTTATGTTAATAGAGGACAATTGTGAGAGTTTAGGTGC

TCAGACATATAACTATGAATATTGTGGAACGTTTGCTGATTTAGGTAGTTTTTCTTTCTTCTTTTCACATCACTTACAAA

CAATGGAAGGTGGAATGATTGCTTGTAGAAATAAAGATGACGCTGATTATTTAAGGTCATTAAGAGCACACGGTTGGTGT

AGAGATTTACCAGACGATAATAAGATTTATAAAAAGACAGGTGATAAGTTTAAAGATAGTTTTACGTTTGTAACTCCAGG

TTATAGTGTAAGACCTTTAGAAATGAGTGGTGCAATTGGAAGTGTACAACTTAAAAAAGAACAAGAAATGAGAACACAAA

GAATTCGTAATGCAAACTATTTTCAACATAAGTTTGAAGAAATGCCAGGTGTACAATTACAAAAAGAAATAGGAGAGTCT

AGTTGGTTTGGATTTTCCATTTTGTTAGTTGATGAATATGAAGGCAAAAGAGACCAACTTGTTAAGAAACTTACAGAAAA

CGGAGTAGAGTGTAGACCTATTGTTGCAGGTAATTTTATGAACAATCCTGTAATAGATTATCTTGATTATTATAATAATA

GTTGTCCAAATGCAGATTATATTCATAACAATGGTTTGTTTATAGGAAATGATATAAGAGATTTAAAAGAAAATATTGAT

ATGGTATATCACATAACAAAGGACATAAAATGAAAAGAGCATTGATTACAGGTATAACTGGACAAGACGGCGCTTATCTT

GCTAAATTGTTATTAGAAAAAGGTTATAAAGTATTTGGTGGACAAAGACGAAGTACATCACCAAAACATTGGCGACTAGA

TGAAATGGGTATTACAGACCAAATAGAGTTTGTTGAACTTGATGTAATAGACCAAGCGAATATAAGAAGAGCAATAGAGG

AATCTCAACCAGACGAAGTATATAATTTAGCTGCTCAATCATTTGTATGGTTATCATTTAAACAACCAGAACTTGCTACT

CTAATAGACGCAATGGGTCCTTTGAGAATACTAGAAAGTATAAGACAAGTAAATCCTAAAATAAAATTCTATCAAGCAAG

TACAAGTGAAATGTATGGAAAAGTATTTGAAACGCCACAAAAAGAAACAACAAAATTCTGGCCGAGGTCACCATATGGTG

TTGCAAAACTATATGCTCATCATATAACAATTAATTATAGAGAAGCGTATGATATGTTTGCTTGTTGTGGTCTTTTATTT

AATCACGAAAGTCCACATAGAGGTGAGGACTTTGTAACTAGAAAGATATCAAAAGGTTTAGCACATTGGTTGCAAGAAGG

AAGACCAATTGTTTTAGGAAATTTAAATGCAAAAAGAGATTGGGGACACGCTGAGGATTTTGTTAGAGGTATGTGGCAAA

TGCTACAACACGATAAACCAGATGACTATGTATTAGCAACTGGTGAAATTCATACAGTAAAAGAATTTGCAGATATGGCA

TTAGATTATAAAGGAATAAAACATTATTGGAAAGATGGTAAGTGTTTTACAGACGGCAATCAATTAATTATTACTACTGA

TAAGAAACATTTAAGACCTGCTGAGGTAGATGTATTACAAGGGGATGCTAGTAAGGCAAGAGAAGTATTAGGTTGGGAAC

ATAAACATAATGTAGAGAGTTTAATGAAAGAAATGGTTGACGCTGATGTAGGTAGATATTGTAGTGACCATCAATCAGGA

GTACCAAGACTTTGGGATGCTCCAGAGAATTGTATATGAGCGGACTCGGAGCATACGGACCAACTAGGGGATTAGCCCCA

TACATATTTTGTACAACATTTAATAAAAGATTATATGATGATTATGCTCATCAATTAATTGACTCATACGTGGCAACAAA

TCAAAAACCACATATGTATGTTTTTGTTGAAGATAATCCAAATTTATATCCTAAAGTAGATAGAGTACATTATTATAATT

TATTTGACTTTGAACCAGATTGTAAAGATTTTGTTCAAAGAAATAAACATAGAACAGCAAATAATTTTTATGAAGAAGCG

GTAAGATTTAGTTATAAAGTATTTGCTCAATCAGCAGCAAGAGGTTGGGGAACTAAAATATATTATGTAGATAGTGATTG

TAAGTTTATGGACACAATACACGACTCTTGGTATCAGAACTGTTTACCAGATTTAACATTTTTATCATTTTATCATAGAC

CATCACAATATACAGAAACAGGTTTTGTTGCATTTAATAATAGAAGTCGTGTAGTAGATGACTTTTTTCGTGAATATAAA

AAATGGTATATAACAGATAAAGTATATACAATAAAGAAATTAGGAAAGAATTTTTGGACAGATTGCCATACATTGGACGG

TACTAGGCAAATGTTTAAAAACGATCCCAGGTATAGTGAAAAACCACTAGGGGATGGTAGAAATGGACATATAATGGCAA

GAGATACGTTTCTTAACCCATATATAGACCATAGAAAAGGACAAAGAAAAAAACAAGCAAATAGTCCAGAATGGAGAAGA

AACAGATGACAAACGGCGTAGATGACCAAGACAATGCTCATAACTTGACATATGAAAATGAAACACAACTTAATAGAACT

GTTACGATTCCTTTAAGAGAATATGATGAACTTAAAGAGCAACGACATTATATTACGGATGAGGATGCGATAGCGATTAT

TGATAAGTTAGGTGAGTTAGTAAGAGCGTTGCGTAAAAATATAAGAATGCCTATAAAAACGAAATAGTAATGATTAATAT

TTTTATTGGGTATGATAGTAAAGAGAAGGTTGCTTTTAATGTACTTGCATATAGTATATTAAAACATAGTACCAGACCTG

TATCTATTACACCAATATATTTAAAAAATATAAAAGATAATTTTACTAGAGAACGTAGTAATATAGAATCAACTGAATTT

AGTTTTAGTAGATTTATAGTACCTCACCTTATGAATTATAAGGGGTGGGCATTGTTTATGGATTGTGACCAGTTAATGTT

AACAGATATTGCTGAGTTATGGAGATTAAGGGATGAGAAATATGCTGTACAAGTGTGTAAGCACGATTATATACCAAGAA

AGACAAAGAAGTTTTTAGGTCAACCTCAAACAAAGTATGAAAAGAAAAATTGGTCAAGTTTTATGTTGATGAATTGTGAT

AAGTGTACAGCACTAACACCAGATTATGTGAATAGTGCAACAGGATTACAACTACACCAATTTAAATGGTTAGAAAATGA

TAATATGATTGGTGAACTACCTTTAGAGTGGAATTGGTTAAGTGGAGAATATCAAAAGAAAGATGATGTTAAGAACGTCC

ATTTTACAGAAGGTGGACCTTGGTTTGTAGATTATTTAAATTGTGATTATGCAGAAGAATGGTTAAAATTAAAGGAAGAA

ACAACTGAAACAAATATGGTTAAATGATACAAGGATTATTAACTAGACCAGCAACAGATGATATTGTAAGACATTTTGTT

TCAAGTGCAGGAGGAACATTACATAATGTTAAAGATGTTGATATAGATAAACCTATTACTTGTTTTGGAATATTAAGAGG

CACAGGCGAACTTTTGAAACAAAGTAAAGAGTTTTATTATTTTGACCACGCTTACTTATATGGTAATAGACACTCTCCAT

CAAAGGTAACTGGTGAGAGAATATATAGATTAACAAAAAATCATTATCATATACAAACTATACAAGAACTAACAGATGAA

GATAATGAAAGAATTAAAAAGTATAAACAGTATATAAAATTACAACCCTGGAAAAGTGATGGCAATTATATCTTAATTAT

AGCACCTTCTCATTTTCAAATAGCATATCATAATATAGGTAGTTGGGTTGATGATACTATAAAGACTTTAAAACAATATA

CAGATAGACCTATTAAAGTAAGAGATAAAAAGAGTAGCAGACCTTTAAGAGAAGAAGTACAAAGTGCATATGCTATAGTA

TCTCATAATTCAGCAGTTGTAGTTGACGCTATTTTAAATGGGGTACCTGTATTTTGTGATAAAATGAATATGGGTGTACC

AATGGGGTTAACCGATTTTAGTAAAATAGAACAACCTATAAGACCTGCTAGATTAAACTGGATATATAGTTTATTAGCAA

ATCAATTTACTATGACAGAAATAAAAAATGGAACAGCTTGGAGAAAAGTACAATGAGATTAGATAACGAAGTAAAATTAG

ATTATAAAGACGTATTGTTGAAACCTAAACGGTCAACATTATCATCAAGACGTGATGTAGAAATGACTAGGTCATTTACA

TTTAGAAATTCTGGTGAAACATATGAGTGTTGTCCAATAATAGCAAGTAATATGGATGGAGTGGGAACATTTAGTATGGC

GAAAGTTATACAAGAGTATAAGATGTTGACCACGATTACAAAGACAACAACGATAGAACAATGGAGAAAAGCAGTAGGTG

AAGGTATTAAATTAAAGTATCTATCAGTATGTACAGGCACAGGTAAATTATGGGATGATAATGCTGAAGATTATACTACA

ATGCAAAAAGTATTAAAGAGTTTTCCAGATGTTAAGTTTATTACAGTAGATGTTGCAAATGGTTACCATACAAACTTTTC

AGATTTTGTTGGTGCAGTTAGAGAAGAGTATCCAGACAAAACTATAATTGCAGGTAATGTAGTAACTGCTGAAATGACCG

AAGAACTAATTATACAAGGTGCAGACGTAGTTAAAGTAGGTATTGGACCAGGTAGTGTATGTACAACAAGAACAATGGCA

GGTGTAGGTGTACCTCAATTTAGTGCAGTAGTAGAGTGTGCTGACGCTGCTAATGGTGTTGGTGGTCATATAGTTGCAGA

TGGTGGTTGTAATATGCCAGGAGATATTGCGAAAGCATTTGGTGGTGGTGCTCATTTTGTGATGTTAGGTGGAATGTTAG

CAGGACATAATGAAAGTGAAGTAGAAAGTAAAGATGGTAAAAGAGAGTTTTATGGGATGTCTTCTGATAGAGCAAGAGAA

GTACACGGAAAACGAAAAGACGGTTATAGAGGTAATGAAGGACGAGCAGTAATATTACCTGATAGAGGTGCTGTTAAAGA

AACAATAGAAGATATATTAGGGGGTGTTAGGTCAAGTTGTACCTATATTGGTGCAAGACGACTAAAAGATATTCCTAAAT

GTGCAAGTTTTGTTAGATGTAACCAATCATTGAATACAGTATTTGAAACTTATGATAATAACGCATAATATACCTTGGGA

TAAATGTTTAAGTAAACAGTTATTTCCTGCCATAGAAAAAGGTTGGACTGATACAGATAAACCTGTTCACTTTTTTTGGG

GGTTAGCTGGACAGAATAGAAAAGAAATACGTAAATGTATGGAGAGTGGTGAGGAATGGTGGTACGTAGATGTTGGTTAT

TTAACACAACAAATTACAAGATATCCAGAACCTAAAATACACGATTACGATAAGACATATTTTAGAATATGTAAAGGTAA

TATACACACGATTAGGTGCAAAGTTGGACCTGGTTCAAGATTACAGAAACTAGAGCATCAAGGGATTGATGTACAGTTTA

AAGGGTGGAATACTGGAGAAACAACTCATATACTAGTAGCACCTTCTTCTGAAACGGTAACTTACCAAATCAATGGTATG

AGCCAATCACAATGGGTTGAACAAGCAACAAAACAGATAGCAGAACATACAGATAAACCAGTTAGATTTAGAAATAAACC

TAGACCTGGTAATGAGTTTTGGAATACAGATATTAAAGAAGACTTAAAAAATGCTCATTGTTTGGTAACCAATATGAGTT

TATCTGCTATTGATTCAATATTAAATCAAGTACCTGTAATATGCCATCAACGAAATATAGCGTCATTTGTTTCATCAAAA

GATATAAAGTTTATAAACAAACCAATGAGACCAGGACGAAAGACTATAACAGAATGGTTAAAGATGATTGCAGAAAATCA

ATTTACAATATCTGAAATTACAGATGGAACTGCTTATAGAACATTACAAGAACAAAACGTATGATAATTAAACCATTTGG

AGAAGAAGGCATTGCTATGTTACGAGGTAATATAAAAGACGTACCTATAACAGATTATATTTGTGCTTTGTTAGAAGAAG

AAACTGATACAGGAGATTTTTTAGAACAGATACAAAAAACAACTCTTCAAAATGGAAAAATGAAAAGGGTTCCTTTTAAT

GAAAAAGTATGGGATTTAATTAATAAGATAACTTATGAATATGAATCAAAAGATAGAAAAATAATGGAAGTTTGGGCAGG

TTTAATGCGAGAAGGAGATTTCCATATGCTCCACGGTCATCAGGAAGTGAAAAATGGTATATCTGGTGGTCTTTATTTAA

AAGTACCTAAATTGACACAACCACAAGGTAATATGAATTGGGTATCAAATAATAAAGTGTTTAGTTGGAGTCCAAAAGAT

GGAGATTACTTCGTTTGGCCTTCTACTTTATTACATTGTGTTTATCCTTTTAAAGGACCAGGAGATAGGATAATGATTTC

TTGGAATAGTGTATGATAAATTTTTGCTGTGTATATTATGGAACAAAGTATTCACTAGATTATGTACAAGTGCTATACAA

TATGGTTAAAAGACATTTAACCATACCCCATAAGTTTATATGTTTTTCAGACCACGTAAAACCTCAAAAGATATTAAAAG

GTGACATAGAATTTAGAAAGTTTAGAGATTCAACTTATGACGGTTGGTGGAATAAAATGCAGTTATTTAGAGAAGAGTCA

GAATTAAAAGGTCCTTGTTTATATATGGATTTAGATGTAGTGATTTTAGATAACATCAATGAATTAGCGACATTTGGTGA

TGATATGACATTTGGTGTAATAAACGATTTCAACCTAGCAACGAAAGAGTATAATTCAAGTATAATGAAATTCAATAATG

AAGTTGCAACTAAATTAGTATGGAATAAGTTTTTAGAAGATAAATCTAATTTAATGAAATTGCAAGGTGACCAAAACGCA

ATGTCCAAATTAGTTAAAAATAGTAAGAATTTAAAGGTTATGCCAGACGAATGGACATATTCTTATAAATGGCATAGTAG

AAAAGACCCTAGATTTCATAAGTCAGGTTGGAAGTTTGAAAAGAAAGAAAAAGCGAAAGTTGCAGTATTTCACGGAAGAC

CTTTACCACACGATTCAGACCAAGAATGGGTCAAGAAATTGTGGAATTAGAACAAAACAAGAACAAATATCTCTAAAAAG

TCAGTAAAATCAACGTAAATTAACTATTGACTCTCCCGAAAAACTCCTGTATAGTATACACATACTATGAAAATAAACAC

TATGAAAAAAACAAATCAAGTAAAATTAAATGACGTTGACTATACTTTTAATGTAGTTTATTTAAGAGAATATATTGATC

CAGATGACCAAGAATTCTTTTATGCATACGAAACTATCTATAGAAACGTTCCATATAAATTCAAAGACAAATTCAATACA

AAATCTATGAAGATGAAAATTCTTAAATATTGTGATTGGAATTATAAAGAACCTGCTGTTAACTTTCAAAACGTAACTAA

AGTTGAATTAATAGACCAAGATGAATATTATAAAACATATGAACAAGTATTCGGTGATGTTGCAGAAGACAATAAATCAA

TGTTTAATGATTACGGTCAATCTTATGACAGACAATCTTTCAGAAAAGATTTTAATAAAGAATTAACATATAAATTAAAC

CCAATAAAAAGAAAAGTAGAACAAATGAAAGGACTAAACTAATGAGTGCAACTAAAAATCTCGCTTGGGACCAAGCAACT

GAATTTTTAAGTAAAGTTGAAAGCAAGTTATTAGACGGTGAAATGACTAAAGAAGTTGCTTTAAAGAAACTTAATGAAAC

AAATTATAACATTGCAATGGAAGGACTAGACTCTCCTGATAATATAGAAGAGTGGATTGACCTTACAATTGCAGATAGAC

AAAATCAAGTACAACAATTAAGAAACGGAGGAACAATATGAGTAATATAACAGACCAATATATCGGAAAAGACGATATCG

GTAAAAACCTATACAGAAAGAAAACTTATTATACTTTATGCATAGAACAAGATTGTCTTGCTAAAAATCAAGAAGAAGCA

GACACTAAATTAAGTGATTGCGGAATTGATTATAGTAAAATCAATAAAGACTTAGCAGAAGAAAAAAACGGTGTTGAAAC

CTATATGACAGACGCCAATTATACAGATTCAGATAAAACTGAATATGTTGCAAAAGTTGTTTATGATGACTATGACGGTT

TAGAAAATGCCATAGAAAACGGTGATGTTGAGTTAGACACATACGCTTTAGAAAATGATATAGTTACAGCAGACGGTAAA

GTTGTTGATAAAGAAGAATCACCACTTGACGATTTGAAAGTAGCATTAAACCAATGACAATCGGATACGCAGTATTATTA

TTTACAATCGGAATGACCTTAACAATAGTTGGGTTTTTCCTTGCGTATAATTACGGAAGTAAAGAGAAGAAAAAAGAAAA

ATTAACAACTGTTCAACAATCATTAAGAGATTTAAATAATGGAGATACTGACTAATGAAATATAAAGAAGATAAAATATT

AAAAGAAGTATTAGAATATATTAAGACAACTTATTCTAAACATTATTCAACTACTAAAGAAGGTTTCCAAGTACAAGACA

TATTAAGACATTTAGATATAGATAAAGATTTCAGTTTATCAAATGCAATAAAATACCTTATGAGATATGGTAAAAAAGAC

GGAAGAAACAAATTAGATTTATATAAAGCAATACACTATATTGTTTTATTAATCAATAGTGAAGAAAACGGATCCACTTT

AATGGAACCTAATATGGAAAAAATACAACAAACAATAAAAGAATTATCAGTTACAAAATGAAAAAGAAAACAAAAAAACT

TATATTAGAATTATTAGATTTTTGGCCAATGACAATAGTTGTGCCAACAATGATTATTTTAATTTTAACAGCAAATATAT

GGTAAGTAATAAGATTATATACAAAAAAATGAATTTTTACTATGATGTAAATGATATGAATATATCTATTCACAGTACAG

ACTGGAAACCAGTAGAGTTTTTAAGTAATGAAGACCAAAGAGAAGAAGTAAGACAACATATATTAAAAAAAGATTTAACA

CAAAGAATAGGAGGCGAAAAGTATATGAAACTAGTAAAACCAGAACCAGAAGATTCAATTATAGATACAATGCTACAATT

GGAAAACGAAATGGCAATAGGAAAATAATATGGACGGAAACGGAATGATATTGTTAATCCTGTTTATAATTTCAATGGGA

TGTTTAGTTTATATGATAATTCTATCAAATGAAATGAGTACAATAATTGATAGATTATTGGGTAGAACTAAAAGACTAAT

GAATAAAATAGACAAGATAACAGACGAAAAAGATGAATAATCCCGCTATAGCAGACTATCAAAGCAGTTGGAAAAGCTCA

CCAGCACCCTTGCTAGAGCGTTGGAAATGCAGGAAAACGAGTAAAATAGAGCATAATTTAGGGATTGACATTTAGCACGT

TTTATGTTAATATTAATACAATTGAGAAAGGAATATACATTATGAGTACGGTGATATACAATAAAGAGAACATCTACAAA

GAGTTTAATGTTGCAAAACAAAAAGACATTGAACTATCAGACAAGAAAACACAAGAAGAAAAAGAAGACGATATCCATAC

AAACAGATTGCAGTTTTGTAAAGACCATAAAGAACTAAATGAGAAAGACCCAGGTTTATACGATTGCGATATTAAGTGGG

ACAGTTTAATAACTGCTTATTCTTCTGAAAGTCCAAGAGACCATTTCTATAAATCAGTATTCGGCAGAACATATGCTGAA

CAAATGAGTTTTGAAACTTCTGAATCAGAAAAAGATGATGACGGAGGAGAAGATTCATATTATAGAAGTAGAAGAAAGAA

TAGAAGTTATAAAAGATAATATGCCAAAGTTTAAAGAAATTTTTGACCCAATGCAAACCGTTTGTGATGATTTTCACGAA

TGGGTAAGAATAGAAACAGAAAAAGTTAATGATCCAGTAATGGTTCAGATGACAATTTTGGGTCAAACATTAAAAATTAT

GAAGTCAGTAATGCCTAGTGCAGACTATGACGGAATAATGGAAACGGTTTATAAATCAAAAGATAGAATTGAACCGTTTA

AAAAGGTAAGTGTACATTAATAGAAGGAGAATATTATGAAAACTATAATGTCAATACTAGTGTTAATTATACTATCAACG

TCTGCTAATGCAGGAGCCGTTGAAGATAAGATTAATGCAGTAAATACGTGGTTTGCTAATGAGAAAGCAACTACAATTGA

ATTTCAAAAAGTACAATGGGAAGATGGTAAAAACCAAATTGCTAGTACTATTGCGAAATTTAAAAAAATGTTGAACTGGA

GTAACTAATGAACGATTTATATGTAAATTTTTCATATACTCTTGAACCTTGGTTAATACTAGGGTTAAAAGTTTTAGCGG

TTTATTTTTTATATAAGATTGCTAGAAATTTAAGAAGTATATTAAAAGAGATTTGCCACATTGGTAATACAAAAATGGGC

AAACCTTTGGTATTAAAGAAAAGAGTGAAATAATGGCATACGGAGATTTTGTTTGTACAAGTGCCAATGACGGTACACAT

TATTTCAGACCTATTACTGCTAGAGCACATACGTTCTGGCAGGAAAAGGGTTTTAATAATTATGTTATTGATAATAACGA

AGACTATTACATAGTTAAGAGTGTTAATAGTCAGAAAATATGTAATGAGATACGCAAGAATAATATGGATTTTACTAGTT

AGTTTACTACTAACAAATTGTGCTAACAGGTCACATATGGGTGCTGTGTTGGGATCAACAACAGGAACAACAATGTGTTT

AGAGTACTTGGGAGATAATCCCTATTTGATTGCTACGTGTGCTGTCAGTACTGCTTTTGTCGGTGCAGAATTAATGTATA

AGAGTGATAAAGATATACACAATGCCGTATTTGTAGACCATTTGAATACAAGTCCAAATGGTTCTTCATATACGAACTGG

TATAATTCAAAAACAGGTAATGGTGGAATTATACACATAACAAGGTCATTTACAATTGGACCACTTAAATGTAAAGATTA

TGACACTACTACAGATATTACAAATAGTTGGCCAATGATAGGTATTGGTGGAGTTAATAGAGAAGTTAATTTTGGAACTG

CTTGTCAGTTGCCAGATGGAACTTGGATAGAAAAACCAGATGGATTAAGAGCTGATATTGACGTTGAATCAGCAACAGAC

AAGAATATTTACGAAATAAATGCTTATTATATGAAATATTTTCCTGATAAGAGGTTTCAATAATGCAAAATCCAGATAAA

ATTGTAGCAACAACAATATTAATAGTATTATTATTATGTGCATATGCAGTAAGCGGTGCTCAAGCGTGTGTTGATTGTGA

TTTAAATAAAGACGCATTTAAAAAAACTAGTACTATAAAATTAGAAACAGGTAAAATTTTATATGACAATGTTAGAGAAG

TTAAAAATGATACTGAACAGTATTGTTTTGTTAAAATATTAATTAAAGAAAAAGATGGAGTGATATCTAAAGAAGAAGAG

TTATATTGCTCCGATGGAAGAAAAGGAATTGACACTCCTAGTTATTGGGAGTTGTTTGCTCAGTTTTACTACCGTGATGT

AGCTACACCAGAGTATTGTAGATATTACAGTAGAAAAAAACACGCTTTTAAATCGTTCGGAAAAGTGTGCTTAAATCAGG

ACGGAGAATGGAAGGTAAAGAAATGATAAAGAATATTATCATAATTGCTCTCCTATTAGTTATTGTATATGGAGTAAGTG

CTACAGAATTTTTGGGTTATGCTCAATCTAGCATTGACTTATTGCAAGAACTGTTATATAATGTACAAAGGAGTGTGAAA

AACTAATGAACAAATACATTAAGATTTTATCAGTTGCAGTACTTGGTCTATTATTGACTAATTGTGCAGGCAATTATAAA

ATCAAAAGTGAAAAAGGTAAAGTAGTTAATACTGTTCCAAAATGGTATATGGCTGATTTTTCTGAAAAAGAAGCGTGTGA

TATAGCACGATTCGGTAAAGAGAAGGAAAAGCAATGTATATTTGGAGTTGGTACTAGCGTTTCACCAGACTTGAATCTCG

CAATTGAGAAAGCTAAAATGATAGCGAAAGCTGAAATAGCAGACATTATCAAAGGGGAGATGAATAAAGAGTCAAAACAA

TTTATTACTGAAATTGGAAAATCAAACAGTAAGACAGTTGTTAGTGAAGTAGAATCTGTATTGGTCAATATTATTAAAGA

TACACCAGTTAGAGGATATGAGATTTTTGCTCAAGACGTAACCTTAACAAAGAACGGTTACTATAGAGCTTGGATCGGTT

TGAGATTGCCATTAGGTGAATATAATAAAATGTTCAACTATACAATTGCTCAAGCAACAGACGCTTATAACTTAAAGTAT

CACGCTAACAAATCATTTGAAAATCTTATGAAAGAGGAAGATAACAATGATAAAGAAGTTAGCAATTAAAGATATCACAG

TATATACAAAACAAAATTGTGTATACTGTGTAAAGGCAAAGGCCTTGATAAAAGGCCTTGGTCTAACTTATACAGAAAAG

AAATTAGAAGAATTTTCGTCTGTTGACGAAATGATTAAGGACATTGGTAAAAAAGTAAGAGCAATGCCTCAAATCAAAAT

AGATGGCGAACTAGTCGGTGGATATAATCAACTTATAGAATATTTTAATAATAAAGGAATAGTGAATTATAAAGGTGAGA

TTACACGTGACTAAAGATAAAGATAAAAATAATTTAATTTTATTTCCTGAAAATAGAATTAAAAAGAGAATTACAAAACC

ACAAGAATCCCCATTTACAAAACGATTAAAAGAGCAACAAACTAGAGAGTTTATTGAACATAGTGTAGATGAAATTGGAT

TTGATTTATTAAGAAAATTTAATGAAATGGGATTAAAGACTTCAAAACAATCATTTACTAAAGACCTTGCGTTAGTTATT

GATTGTATAAGAGGTTTAATTTATAGAGATTTTGATATGGCACACGCCGCTCAATTAATGGCAGATAAAATGGTAATGAT

AAAATTTAATAGAATGGGTAAAGCATCCGCTGCCAGGATTGATTATTCAGCTTTTATGCCAAAAGCAAAACGACCAAATG

TTTTTAATAAAGAATTTAAAGAAGAGTTAAATGATTTACAAGATGGATCAGATATGTTTGAGTCTGATATGGATTTGAAC

GGTGATGATGATAAGAAATAGTTTAATAATATTAATAATGCTAACTTTTATGGGTTGTACAAAACCAAAAACTGAACTGA

ATGCAATGGAGAAATTTTTTGATTGTATTGGTAGTGGTAATTGTGAAGCATTTAAAAAGAATTCCGTAGAGGAATAGTCC

TATGCAGACTTTAAAAAGCAAAAATAAAGGAGGAAGAAACATTATGTTTTTTTCAAAAAGTAAAGTTGCAGTTGCAACTA

AAGGCAGAAAAAGACTGTCTAAAACTCAAAAAGTATTAAACTTATTTGAGAAAGGTGAACCAGTTTCTTGGAAACATTTA

AGAAACAGATATGACCTGATATCACCAAGAGCGATGGTTGACAAACTACGTTCAAAAGGTCATATGATTTATATTAATAA

ATCATCTTCAGGTACATCTTATAGATTGGGTACTCCTACAAAAGCTATTATAGCTGCTGGGATACAAAAACTATACGGTA

CTGAATACGCTTATAGTGCGTAATAGAATCGTAACCAATACGATTGATGTAGGCGACTCTCGGGTCGCCTATATTTTTAT

ATACAATGAAAACAACAGATTTAACACCAGTAGAAATACATAACAACATCTATTACAAAAGGGATGATTATTATGCTCCA

TATGGTAAAGACAATGTTAATGGAGGTAAGACAAGACAGGCAATTTGCTTGTTTAGAGAATTAAAAGATGAAATTAAAAA

CAAATATAACGGTGGAGTAGTTACAGGTTCATCTGTTAATAGTCCACAAGCACCTATCATAGCGGCAGTTGCTCAAGACT

TTGGTTTTAAATGTGTTATAGGTGTAGGTGGTACAACACCTAAAACAATAGACACCCACCATATGATAAGATTATCAAGA

CACTATGGTGCTGATATTGAAAACGTTGCAGGTCACGGATATACAGTTGCAATAGATAGTGGATTAAAAAAGAAAGTAAT

ATCTAAAAAAGGTTATATGTTAATCAAGTTTGGTAATAGTGCTGCTACGAATCCTGAATCAATATTTGATAGTGTTGCTA

ATCAAGTTAAAAACATACCTGACAAGTTAGATAATATAGTAATTTCAGTTGGTAGTGGTATACAGTTTGCAGGTATAGTA

AAAGGTATAGAGAAGTTTAAGAAAAAAGTAAAAAGAATTATAGGGGTCACCTTTGTTGACCGTAGTAAAAAGATTGACGA

GTATTTAAATCAATTTAGTAATCTTGAATCAGGTTTTAAGAAGTTTCAAGATTATGAAATGTACAAAACACCATATCCAT

ATTCAAAACCCATATGGGAAGATGTTGGTAATGGCTTTATTGACGATATATACGAAGGTAAAGCACATAAATGGATGAGA

GAGAATATAGATACTACAAAAGAAAAGACGCTATTTTGGAGTATAGGGAGAAGATTAACAGCGGAACAGGTAGATAAGTT

ATATAAATAGATATATGATTAATATTAGAAATTGGAGTATAAAATGGCAGAAGAAGCAAAACAACATCCATCATTAATAA

GTAAGTCCTCTATGGAAGCAATGGCCAGCACCGCTGGTTCAGGTGACTTGCTATTTTCAGAAGTCTTAACTAGAGTAAAT

AACGCAAAAGATAAATCTAAAAAGTTGACGGTCTTAAAACAATATGACCATCCATCTTTAAGGATGCTTTTAAAAGGATC

ATTTGATCCTAGTATTGAGTGGGAGTTACCAGACGGTACACCTCCTTATATGGAAAATCCAGCACCGAAAGGTACTGAAC

ATACAACACTTAAAACTGAAGCAAAACGTTTGTGGCATTTTATTAGAGGCGCAGACAATAAAACTACAAAAACTCAAAAA

GAAACTATGTTTATCCAAATGTTAGAAGGATTACATATGGACGAAGCGAGATTATTGCTTAGTGTAAAGAATAAAGAATT

ACATAGAGCATATAAAGGGTTAAGCGACTCTGTAGTTAAAGAAGCGTTTAGATGGAACGAATTGTATCAAAAAGAAGAAC

AAAAAAAGAACATATAGTCAAAAAGACTTGGTTTTACTTGATTTTAAGTGCTTGACTTTCCTTTCTTTTATGTGTATAAT

AGATACATATAAACAATAAATATAGAAAGAGAGAATATATTATGAAAAAAGTGATGTTTATTATACTATTGAATTTAGTA

ATATGGTTTGGACTTACTAGTCTATCCAATATTGCTAATGCAAATGATTATAATAAAGCAGTTATAGCACACGTTATCAA

GGAAAACCTTGACGGCAACGGTGTAGATTCAACTGCTTTAATGGAGGCAGAACTACATAGGATAGTATACGCTATGATAA

ACGAATTTAGTGGCGTATTACAAGAACACCTACCAAATATACTAGATAGTATTGCTAGTGAAATCAGACAAAAAAACGAT

AAAGAGTTTAAATGTGCTCTTTTAAAAGGTAGTGACTATGAGTGTAATTGAAAATATAGTTAACGTTTTACATTGGATAT

ATCAATATATTCCTAGAGAATTAGTAATAGTAATTCTTGGGAGTATGATTTTATTTGTTATTTTAGAATTAGGGGATAGA

AAAAGAAAAAGAGAATGGCTAAAAGAACAAAAACAGTTACAAAAAGGCAGAAAATCAAAAGAAAGTTAAAGAAGGAACTT

TCTGCTGTGAAGATGTTAAAATATAAAACTACATATAAGGATATCAAAAAGTATTTTAAACTTATTAATGAACACGTGTT

TGATAATAAGTTATCTCCCTTTAATGATATTGAGTTGGTACATAAACCAAGAAATTACATAGGACAAGTTGTAATAAATG

ATAAGATAGGCAAAGGGACTAGAAACTTTGTATTAGAAATGTTAAAGTCTTATGGTAATAAAAAAGAATTTGTTGATACG

TTGGCACACGAAATGATCCATCTGTATCAAATGGCAAATTTAGGTGATACAGGAAATCACAACGATACATTTTTTAGTTT

TAGACCAAAACTAAAAGCAGTCGGATTAGATATATAAATAAAACAAGGAATAGATTATGGCAGAAGTGAGAAAGACAAAG

GAACTAGACTACTATTTAAAGAGAATAATTTTAAAGGTTCCAGACAAAATTCAACAGTTTATAGATAATGAGAACGGTGA

ATTCTCTATGACTTATTATACTGGAGATTGGTCAAAAGACATATATGATAACTTTACTGAACTACAAGCAGAAAAGATAT

TTAAACGTATGGCACAATTTCAGAACAAGATAAGTTTTGTCCAAAAGAAAAATCAACCATCAATCGGTGGTTATGAATAT

CAGATAGCGAGGTTTTAATGAAACTGAACTTAAAAAAATATTCAGGTGCTTTCAGAAAAACATATTGGTGGATTAAAGCA

ATTTTAGTAGTTGTTTTTGTATCATCATTAGCATATGGTTGGGGAACATTTACACCAAATCCAATTGCAGTTAAGAAAGC

AACAGAAGAAGTTAGAATAGAACACGCAATTTGGGCAGAAAAATTAGGACTACACGAACCTAGTTTTGAATATACAAATA

AAAAAGAATTTATAATAGAAGTCAATACGTGCCTTGACTATTTAAATTGGAAAACACCACCAGATAAAAGAGTACCAATT

CAAATGGTGACAGCACAAGCCGCTTTAGAGAGTGGTTGGGGTACAAGTAGATTTGCTATAGAAGCAAATAACTTATTTGG

TATTAAGACTTGGGATAAAGATAAAGGTTTATTACCTATTGGTATGAGTGAAGACACACCTTGGCGTGTAAGAGTTTTTA

AAACGAAGTGTAATAGTGTTCAAGAATATATAAGAATATTAAATGAACACCCAGCATACAAAGAGTTTAGAGCATTAAGA

GCAAAACTATTAGAAAAAGGTGAACTGTTAGATTCAGTACAGTTGATTGCTACGTTAGATAAGTTTTCAACTACCGAAGA

TTATGATAAAAGAGTTATCAATATGATGAAAAAAATTGCTCAAGTGTTAGAGGAGTAATTTTAAATGAGAAGTTTACTTT

TTATATTCATAGTACTTTTTAGTGCTATATCTATATCAGGTATTGCTGCCGCTTATAGTATTATAGGACTGGCAACTCTA

TTTGCAGGTGCGAAGATAGCAATTATTGCTATGGGTACTTCATTAGAAGTTGGTAAGTTAGTTGCCGCCAGTTGGTTATA

TCATAATTGGAGAAATATAAATCTCCCACGTACAATAAGAGCATATTTAACAACGTCTGTTATTGTATTAGTATTTGTAA

CTAGTATGGGTATCTTTGGGTTCTTATCCAAGGCACACCTAGACCAAGTAAGACCTAGTAGTGATAATACAGTACACATA

GCATTAATAGATAGACAGATTTTACAAGAAAACGTTGTTATAGATAGAGCAGAAAAGACATTAAACCTATTAGACAAAGC

ATTAGAGGTTTACCTAGATAAAGAATATGTTAGTAGAGGTCTTAAAGAAAGAAAAAAGCAGAAAGAAGAAAGAGATTTTT

TAAATAATGAAATAAGAGTTGCAATGGATAACATTGCAGAATTGACATTAAAGAAAGGTAATATAGAATTAGAACAATTA

AAGATAGAGGCAGATGTAGGACCTCTTAAATATATTGCAGAACTAATATATGGTGATGAAGCAAAAGAACATTTTGATGA

AGCAGTTAGATGGATTATTATTGTATTAATATTTGTATTTGACCCATTAGCAGTATTGTTATTGATTGCTGCTAACATAT

CAATAAGGGAAAGAAAATTAGCAAATGAAGCGAAGAATAAGAAGAAAGAAAAAGAGATTAATTGGCAAAGGGAGGCGACT

AGAGCGAAAACTATATCGCAAGGTCTCCGAGATAAGCAAAGATTTTATAAAACATTTTTTAGTAAATTAGGTAAAAGAGA

TTTAAAGAATAGAGATTATGAAGACTTTTTTAAAGCAATGGGTACAGAAGAGTTAATGAAATTAGGTTTAGATCCAGATG

AGATAAGAATCAAACTAGACCAGATAATGGAATGGAATGAACCAAAGGATAAACCTTATTTAGAATCGGGAGTTAAGAAA

TGAGAAATATAGCAATACTATTATCACTATTACTTTTAAATGCGTGTGGAGCAACAGCACCAGCGTTTTTAGCAACAAGT

GCTGGTACATATTCTGAATATAAAGTTATGTCTATAATAAAAACAGGTGCAGATTTTACATTAAGTTTAGCTGATTTACC

AACAACTAACGATATGGTATTATCACGTATAACTGGTTATGAGTGTAAAGTTAGTAGAGCATTAAAAGAAGGTATAGAGT

ATATTTGTAAAGATGTAAAAATACATCCACCAACGAATACGACCATTGACAAAGATGTTAAAAAGTGATATTATGGTACT

TATGAATACTCATTTATACGCTTGTCCTAGATGTGCTGATAAACTGATTAAACAAGCAGAAAAAGCATTAGATAGGTCAG

AAACAAAGTGGTCAAAGAACTTTTGGCACGGAGTGTGGAAGAAGTTAAGAAGTAAATATCAACCAGAAGAGGTAACATAT

CATTAATGAAACAGAAAATATTAGACGCAGTAAGAAAACACGCTGAAGGTAATATTGCTAGAGCAAAGACAAACGTGGAT

GTATTTTTAAATAATCCAGTTGGTGTTGCAACACATATGGATTCAGTTGAAACAGTTGTTAAAGAATTAAAAGTCATAGC

AGATAATAAAGAGATTATTGAAACTCTAAATGACATCTAAAAAAAATATGTCTAGTAATAGAACTCCTAGACGTATACAT

AGAAATAAAGGTGGACGTAAAAAAGGTACGATTGCACCTATGGTAAAGAAGTATTATAATTTTGGGGCAGCAGATGATGA

ATTCGGAATAAGTAGTGAGTCAGACTATAATAGATTAAAAAAGAAATATGAAATTAAAAATTAAGAAAACTGAATATCAA

GATATCGCTGATTGTATCAGAAGTGACCAAGTACCTGCCTCGGCAGTATTTGAGTACTTTTCTGACAAACCTTTTTATAA

ATGGTACAAGAAGAGATATTTAAATGCCTAGATATACGTTTGAAAATACGAAGACAGGTAAAACTTGGGTAGACTTTATG
[truncated: 4,482,350 more chars]
