# Supplementary material for: Identification of Shemin pathway genes for tetrapyrrole biosynthesis in bacteriophage sequences from aquatic environments
Source: Nat Commun. 2024 Oct 15;15:8783. doi: 10.1038/s41467-024-52726-3 (PMC11480375; doi:10.1038/s41467-024-52726-3)
Supplement: Supplementary file 9 — Supplementary Dataset 6 [file 41467_2024_52726_MOESM9_ESM.docx]

**Ala Synthase Protein**

>SAMEA2622357_9342

MEHLDKFKAVIKDLKDDGRYRVFNDILRTRGDYPNAIWYSKYSIKKIVNWCSNDYLGMGQHSYVLDSMKTALETSGAGAGGTRNISGTTHYHIALEHELTQLHKKESALLFTSAYNANQTTLETMGKIMPDLLFISDAQNHSSIIQGLRHSKCRKEIFKHNDVQDLESILMSNPGPKCVVFESVYSMDGDIAPVKEIVEVCKKYNAITFIDEVHAVGLYGKTGAGICERDNVDVDIINGTLAKAFGVQGGYIAGKREFIDAIRSMASAFIFTTSLSPTICAGALTSIKYVKDHPELREKLQERAKKTKEEIERQGIEVLKNDSHIVPVIIGDPKKCKAVSDELLYKNGIYVQPINWPTVARGTERLRFTPTPFHTDAHIFDMVVKLKSALKRCGKKK.

>SAMEA2622841_6527

MKELEKFTQVIEDYKSDGRYRTFNDIIRIKGKYPHAIWYSKYSIKNIVNWCSNDYLGMGQHNYVIDSMKTALETSGAGAGGTRNISGTTHYHNALERELASLHKKEKALLFTSAYNANQTTLETMGKIIPDMLFISDAQNHSSIIQGLRHSRCRKEIFKHNDVKDLEGILQSNPGPKCVVFESVYSMDGDIAPVKEIVDLCNKYNAISYIDEVHAVGLYGEEGAGICERDNVEVDIINGTLAKAFGVQGGYIAGKRDFIDAIRSMASAFIFTTSVSPVICAGALTSVKYVRDHPELREKIHERANKTKEELTRQGIEVMKNDSHIVPVIIGEAKRCKAISDELLYKEGIYVQPINWPTVAVGTERLRFTPTPFHTDKLIFDMVVKVKSAIKRCGKGLKYDR.

>SAMEA2619791_29744

MEHLEDFKKIVKELKDDGRYRVFNDILRTAGDFPNAIWYSKYSINKIVNWCSNDYLGMGQHQYVLDSMKTALETSGAGAGGTRNISGTTHYHIALEGELGRLHNKEAALLFTSAYNANQTTIETMAKIIPDIMFISDEQNHSSIIQGIRHSKAKKEIFKHNDVDDLESILMSNTGPKCVIFESVYSMDGDIAPVKDERDNIDVDIINGTLAKAFGVQGGYIAGKREFIDAIRSLASAFIFTTSLSPVLCAGALTSIKYVKDHPELREKIQERAAKTKEEIERQGIEVLKNDSHIVPVIIGDAKKCKAVSDELLYKDGIYVQPINYPTVAVGTERLRFTPTPFHTDAHIFDMVVKLKSAMKRCGRNSI.

>Ga0115001_10000031

MEHLDKFKKIITELKADGRYRVFNDILRTRGSYPNAIWYSKYSIKKIVNWCSNDYLGMGQHSYVIDSMKTALETSGAGAGGTRNISGTTHYHIALEHELTQLHDKESALLFTSAYNANQTTLETMGKIMPEILFISDAENHSSIIQGLRHSKCKKEIFKHNDLDDLESILKSNPGPKCVVFESVYSMDGDIAPVKEIADLCKKYKAISYIDEVHGVGLYGPKGAGICERDNVDVDIINGTLAKAYGVQGGYICGKREFIDAIRSMASAFIFTTSLSPVLCAGALTSIKYVKDHPELREQLQERAQKTKEELTRQGIEVLQNDSHIVPVIIGDAKKCKAVSDELLYKDGIYVQPINYPTVAVGTERLRFTPTPFHTDMMIFDMVVKVKSAMRRCGKTK.

>3300000199_SI39nov09

MEHLDKFKQVITELKDDGRYRVFNDILRTRGSYPNAIWYSKYSIKKIVNWCSNDYLGMGQHSYVLDSMKTALETSGAGAGGTRNISGTTHYHIALEHELTQLHGKESALLFTSAYNANQTTLETMGKIMPDLLFISDAENHSSIIQGLRHSKCKKEIFKHNDLDDLESILMSNPGPKCVVFESVYSMDGDIAPVKEIADLCKKYKAISYIDEVHGVGLYGPNGAGICERDKVDVDIINGTLAKAYGVQGGYICGKREFIDAIRSMASAFIFTTSLSPVLCAGALTSIKYVKDHPELREKLQERAQKTKEELTRQGIEVLQNDSHIVPVIIGDAKKCKAVSDELLYKDGIYVQPINYPTVAVGTERLRFTPTPFHTDTMIFDMVVKVKSAMRRCGRTK.

>SAMEA2620861_1137947

MEHLDKFQQVIKDYKDDGRYRTFNDIIRKRGDYPNAIWYSKYSIKNIVNWCSNDYLGMGQHSYVIDSMKTALETAGAGAGGTRNISGTTHYHNALERELALLHKKESALLFTSAYNANQTTLETMGKIIPDLLFISDEENHSSIIQGLRHSKCRKEIFKHNDVQDLESILMSNPGPKCVVFESVYSMDGDIAPVKEIIEVSKKYNAITYIDEVHAVGLYGETGAGICERDKVEVDIINGTLAKAYGVQGGYITGKREFIDAIRSMASAFIFTTSLSPVICAGALTSIKYVKDHPELREKIQERARKTKEEIERQGIEVLKNDSHIVPVIIGDPIKCKAVSDELLYKEGIYVQPINWPTVKRGTERLRFTPTPFHTDAHIFDMVVKLKSALKRCGKKK.

>SAMEA2620413_422904

MEHLDKFQQVIKDYKDDGRYRTFNDIIRKRGDYPNAIWYSKYSIKNIVNWCSNDYLGMGQHSYVIDSMKTALETAGAGAGGTRNISGTTHYHNALERELALLHKKESALLFTSAYNANQTTLETMGKIIPDLLFISDEENHSSIIQGLRHSKCRKEIFKHNDVQDLESILMSNPGPKCVVFESVYSMDGDIAPVKEIIEVSKKYNAITYIDEVHAVGLYGETGAGICERDKVEVDIINGTLAKAYGVQGGYITGKREFIDAIRSMASAFIFTTSLSPVICAGALTSIKYVKDHPELREKIQERARKTKEEIERQGIEVLKNDSHIVPVIIGDPIKCKAVSDELLYKEGIYVQPINWPTVKRGTERLRFTPTPFHTDAHIFDMVVKLKSALKRCGKKK.

>SAMEA2620836_7330

MEHLDKFQQVIKDYKDDGRYRTFNDIIRKRGDYPNAIWYSKYSIKNIVNWCSNDYLGMGQHSYVIDSMKTALETAGAGAGGTRNISGTTHYHNALERELALLHKKESALLFTSAYNANQTTLETMGKIIPDLLFISDEENHSSIIQGLRHSKCRKEIFKHNDVQDLESILMSNPGPKCVVFESVYSMDGDIAPVKEIIEVSKKYNAITYIDEVHAVGLYGETGAGICERDKVEVDIINGTLAKAYGVQGGYITGKREFIDAIRSMASAFIFTTSLSPVICAGALTSIKYVKDHPELREKIQERARKTKEEIERQGIEVLKNDSHIVPVIIGDPIKCKAVSDELLYKEGIYVQPINWPTVKRGTERLRFTPTPFHTDAHIFDMVVKLKSALKRCGKKK.

>3300009593_Ga0115011_10000306

MEHLDKFKAVIKDLKEDGRYRVFNDILRTRGDYPNAIWYSKYSIKKIVNWCSNDYLGMGQHSYVLDSMKTALETSGAGAGGTRNISGTTHYHIALEHELTQLHKKESALLFTSAYNANQTTLETMGKIMPDMLFVSDAQNHSSIIQGLRHSKCRKEIFKHNDVKDLESILMSNPGPKCVVFESVYSMDGDIAPVKEIVEVCKKYNAITFIDEVHAVGLYGPTGAGICERDGIEVDIINGTLAKAYGVQGGYIAGKREFIDAIRSMASAFIFTTSLSPVLCAGALTSIKYVKDHPELRMKLQERALKTKEELERAGIEVLKNDSHIVPVIIGDAKKCKAVSDELLYKNGIYVQPINYPTVAVGTERLRFTPTPFHTDAMIFDMVVKVKSAMRRCGNKK.

>3300009790_Ga0115012_10000076

MEHLDKFKAVIKDLKEDGRYRVFNDILRTRGDYPNAIWYSKYSIKKIVNWCSNDYLGMGQHSYVLDSMKTALETSGAGAGGTRNISGTTHYHIALEHELTQLHKKESALLFTSAYNANQTTLETMGKIMPDMLFVSDAQNHSSIIQGLRHSKCRKEIFKHNDVKDLESILMSNPGPKCVVFESVYSMDGDIAPVKEIVEVCKKYNAITFIDEVHAVGLYGPTGAGICERDGIEVDIINGTLAKAYGVQGGYIAGKREFIDAIRSMASAFIFTTSLSPVLCAGALTSIKYVKDHPELRMKLQERALKTKEELERAGIEVLKNDSHIVPVIIGDAKKCKAVSDELLYKNGIYVQPINYPTVAVGTERLRFTPTPFHTDAMIFDMVVKVKSAMRRCGNKK.

>3300012953_Ga163179_10000108

MEHLEDFKKIVKELKDDGRYRVFNDILRTAGDFPNAIWYSKYSINKIVNWCSNDYLGMGQHQYVLDSMKTALETSGAGAGGTRNISGTTHYHIALEGELGRLHNKEAALLFTSAYNANQTTIETMAKIIPDIMFISDEQNHSSIIQGIRHSKAKKEIFKHNDVDDLESILMSNTGPKCVIFESVYSMDGDIAPVKDIVDVCKKYNAITFIDEVHAVGLYGPTGAGICERDNIDVDIINGTLAKAFGVQGGYIAGKREFIDAIRSLASAFIFTTSLSPVLCAGALTSIKYVKDHPELREKIQERAAKTKEEIERQGIEVLKNDSHIVPVIIGDAKKCKAVSDELLYKDGIYVQPINYPTVAVGTERLRFTPTPFHTDAHIFDMVVKLKSAMKRCGRKSV.

>3300005521_Ga0066862_10001059

MEHLDKFKQVIDDYKSDGRYRTFNDIIRTRGKYPHAIWYSKYSIKNIVNWCSNDYLGMGQHNYVIDSMKTALETSGAGAGGTRNISGTTHYHNALERELASLHKKEKALLFTSAYNANQTTLETMGKVMPDLLFISDAQNHSSIIQGLRHSRCRKEIFKHNDLDDLESILKSEPGPKCVVFESVYSMDGDIAPVKEIADLCKKYNAISYIDEVHAVGLYGKEGAGICERDNVEVDIINGTLAKAFGVQGGYIAGKREFIDTIRSMASAFIFTTSVSPVICAGALTSVKYVRDHPELRDKIHERANKTKEELERQGIEVMKNDSHIVPVIIGEAKRCKAVSDELLYKEGIYVQPINWPTVAVGTERLRFTPTPFHTDNLIFDMVVKVKAAIKRCGKKLNYD.

>3300006166_Ga0066836_10000757

MEHLDKFKQVIDDYKSDGRYRTFNDIIRTRGKYPHAIWYSKYSIKNIVNWCSNDYLGMGQHNYVIDSMKTALETSGAGAGGTRNISGTTHYHNALERELASLHKKEKALLFTSAYNANQTTLETMGKVMPDLLFISDAQNHSSIIQGLRHSRCRKEIFKHNDLDDLESILKSEPGPKCVVFESVYSMDGDIAPVKEIADLCKKYNAISYIDEVHAVGLYGKEGAGICERDNVEVDIINGTLAKAFGVQGGYIAGKREFIDTIRSMASAFIFTTSVSPVICAGALTSVKYVRDHPELRDKIHERANKTKEELERQGIEVMKNDSHIVPVIIGEAKRCKAVSDELLYKEGIYVQPINWPTVAVGTERLRFTPTPFHTDNLIFDMVVKVKAAIKRCGKKLNYD.

>Ga0066836_10000757|480248

MEHLDKFKQVIDDYKSDGRYRTFNDIIRTRGKYPHAIWYSKYSIKNIVNWCSNDYLGMGQHNYVIDSMKTALETSGAGAGGTRNISGTTHYHNALERELASLHKKEKALLFTSAYNANQTTLETMGKVMPDLLFISDAQNHSSIIQGLRHSRCRKEIFKHNDLDDLESILKSEPGPKCVVFESVYSMDGDIAPVKEIADLCKKYNAISYIDEVHAVGLYGKEGAGICERDNVEVDIINGTLAKAFGVQGGYIAGKREFIDTIRSMASAFIFTTSVSPVICAGALTSVKYVRDHPELRDKIHERANKTKEELERQGIEVMKNDSHIVPVIIGEAKRCKAVSDELLYKEGIYVQPINWPTVAVGTERLRFTPTPFHTDNLIFDMVVKVKAAIKRCGKKLNYD

>Ga0115011_10008698|739460

MEHLDKFKQVIDDYKSDGRYRTFNDIIRTRGKYPHAIWYSKYSIKNIDNWCSNDYLGMGQHNYVIDSMKTALETSGAGAGGTRNISGTTHYHNALERELASLHKKEKALLFTSAYNANQTTLETMGKVMPDLLFISDAQNHSSIIQGLRHSRCRKEIFKHNDLEDLERILKSDQGPKCVVFESVYSMDGDIAPVKEIADLCKKYNAISYIDEVHAVGLYGKEGAGICERDNVEVDIINGTLAKAFGVQGGYIAGKREFIDTIRSLASAFIFTTSVSPVICAGALTSVKYVRDHPELRDKIHERANKTKEELERQGIEVMKNDSHIVPVIIGEAKRCKAVSDELLYKEGIYVQPINWPTVAVGTERLRFTPTPFHTDNLIFDMVVKVKAAIKRCGKKLNYD

>Ga0066849_10000198|139039

MEHLDKFKQVIDDYKSDGRYRTFNDIIRTRGKYPHAIWYSKYSIKNIVNWCSNDYLGMGQHNYVIDSMKTALETSGAGAGGTRNISGTTHYHNALERELASLHKKEKALLFTSAYNANQTTLETMGKVMPDLLFISDAQNHSSIIQGLRHSRCRKEIFKHNDLDDLESILKSEPGPKCVVFESVYSMDGDIAPVKEIADLCKKYNAISYIDEVHAVGLYGKEGAGICERDNVEVDIINGTLAKAFGVQGGYIAGKREFIDTIRSMASAFIFTTSVSPVICAGALTSVKYVRDHPELRDKIHERANKTKEELERQGIEVMKNDSHIVPVIIGEAKRCKAVSDELLYKEGIYVQPINWPTVAVGTERLRFTPTPFHTDNLIFDMVVKVKAAIKRCGKKLNYD

>SAMEA2620894_15123

MEHLDKFQQVIKDYKDDGRYRTFNDIIRKRGDYPNAIWYSKYSIKNIVNWCSNDYLGMGQHSYVIDSMKTALETAGAGAGGTRNISGTTHYHNALERELALLHKKESALLFTSAYNANQTTLETMGKIIPDLLFISDEENHSSIIQGLRHSKCRKEIFKHNDVQDLESILMSNPGPKCVVFESVYSMDGDIAPVKEIIEVSKKYNAITYIDEVHAVGLYGETGAGICERDKVEVDIINGTLAKAYGVQGGYITGKREFIDAIRSMASAFIFTTSLSPVICAGALTSIKYVKDHPELREKIQERARKTKEEIERQGIEVLKNDSHIVPVIIGDPIKCKAVSDELLYKEGIYVQPINWPTVKRGTERLRFTPTPFHTDAHIFDMVVKLKSALKRCGKKK

>lcl|SAMEA2620894_15123

MEHLDKFQQVIKDYKDDGRYRTFNDIIRKRGDYPNAIWYSKYSIKNIVNWCSNDYLGMGQHSYVIDSMKTALETAGAGAGGTRNISGTTHYHNALERELALLHKKESALLFTSAYNANQTTLETMGKIIPDLLFISDEENHSSIIQGLRHSKCRKEIFKHNDVQDLESILMSNPGPKCVVFESVYSMDGDIAPVKEIIEVSKKYNAITYIDEVHAVGLYGETGAGICERDKVEVDIINGTLAKAYGVQGGYITGKREFIDAIRSMASAFIFTTSLSPVICAGALTSIKYVKDHPELREKIQERARKTKEEIERQGIEVLKNDSHIVPVIIGDPIKCKAVSDELLYKEGIYVQPINWPTVKRGTERLRFTPTPFHTDAHIFDMVVKLKSALKRCGKKK

>Ga0114999_10006015|760631

MKHLEEFTKIIDEYKQDGRYRVFNDIVRTRGNFPHAIWYSKYSIKKIVNWCSNDYLGMGQHSYVIDSMKTALESSGAGAGGTRNISGSTHYHNALEDELADFHKKEKALVFTSAYNANQTTLETLGKIIPDLLYISDSLNHSSLIQGIRHSRCKKEIFKHNDVEDLERILKSYEGPKCVVFESVYSMDGDIGPVKEINELAKKYNAITFLDEVHAVGLYGATGGGITERDNIDVDIINGTLAKAFGVQGGYIAGKKDFIDAIRSLASAFIFTTSLSPVICAGALTSIKYVRDHPELREQIHERANKTKEELDRQGIEVMKNDSHIVPVIIGDAKKCKAISDELLYKEGIYVQPINWPTVPVGTERLRFCPGPFHTDALIFDMVVKLKAAMKKIMISN

>Ga0115007_10001227|723900

MKHLEEFTKIIDEYKQDGRYRVFNDIVRTRGNFPHAIWYSKYSIKKIVNWCSNDYLGMGQHSYVIDSMKTALESSGAGAGGTRNISGSTHYHKALEDELADFHKKEKALVFTSAYNANQTTLETLGKIIPDLLYISDSLNHSSLIQGIRHSRCKKEIFKHNDVEDLERILKSYEGPKCVVFESVYSMDGDIGPVKEINELAKKYNAITFLDEVHAVGLYGATGGGITERDNIDVDIINGTLAKAFGVQGGYIAGKKDFIDAIRSLASAFIFTTSLSPVICAGALTSIKYVRDHPELREQIHERANKTKEELARQGIEVMKNDSHIVPVIIGDAKKCKAISDELLYKEGIYVQPINWPTVPVGTERLRFCPGPFHTDALIFDMVVKLKAAMKKIMISN

>Ga0098058_1000146|507114

MIHLNKFQDVIEGLKEEGNYRVFNDILRERGKFPQAIWYSKYAVKRIVNWCSNDYLGMGQHRYVIDAMQTALESSGAGSGGTRNISGTTHYHVALEHELAELHQKEAALLFTSAYIANSATLESMAKVLPDITFISDSMNHASIIQGIRHSRAKKEIWKHNDLKHLKELLEKTPDPKCVVFESVYSMDGDICPLKEIVTLCKKYKAITFLDEVHAVGLYGERGGGITERDGVDVDIINGTLAKAFGVQGGYIAADKDFLDAIRSLSSGFIFTTSISPVICAGALTSVKYVRDHNCLRLTMQERSQKTKVELERNGISVMENPSHIVPVIIGDAKICKRISDDLLYKDGIYVQPINYPTVAKGTERLRFTPGPFHTDLMLYDMVIKLRDAMRHNKIV

>SAMEA2622763_437556

MRELDKFTKVLDELKDDGRYRVFNDILRTAGDFPNAIWYSKYSIKKIVNWCANDYLGMGQHPYVIDAMKTALETAGAGAGGTRNISGTTHYHVALEAELAKLHKKESALLFTSAYNANQTTLETIPKVLDGVLYISDEMNHSSIIQGIRHSKARKEIFKHNDLKDLEGILMSNTGTKVVVFESIYSMDGDIAPVKEILALCEKYNAISYIDEVHAVGMYGENGAGVCEREGCQPDIINGTLAKAFGVQGGYIAGKKDFVDAIRSLASAFIFTTSLSPVICAGALTSIKYVRDHPELRDKLHERSQTTKKILKDHDINVMDNDSHIVPVMINDAKKCKAVSDELLYKFGIYVQPINFPTVPVGTERLRFTPTPYHTDAHIYDMVLKVKSAIKRCGKVK

>SAMEA2620413_111863

MNELDKFQSVIDELKDDGRYRVFNDIIRKAGDFPNAIWYSKYSIKNIVNWCANDYLGMGQHSYVLDSMKTALDTAGAGAGGTRNISGTTHYHVALETELAKHHNKESALLFTSAYNANQTTLETIPKVLGDVLYISDEMNHSSIIQGIRHSKARKEIFKHNDIKDLEGILMSNTGTKIVVFESVYSMDGDIAPVKEIVELCKKYKAISYIDEVHAVGMYGKNGAGICERDNVDVDIINGTLAKAFGVQGGYIAGKQIFIDAIRSLASAFIFTTSLSPVICAGALTSIKYVRDHPELREKLHERSQATKKILKEHNIPVMDNDSHIVPVMINDAKKCKAVSDELLYKFGIYVQPINYPTVPVGTERLRFTPTPYHTDAHIYDMVLKLKTAIQRCGKIK

>SAMEA2620413_474846

MIELDKFTKVLDELKDDGRYRVFNDILRTAGNFPEAIWYSKYSIKKIVNWCANDYLGMGQHSYVIDSMKTALETAGAGAGGTRNISGTTHYHVALELELAKLHKKESALLFTSAYNANQTTLETIPKVLGDVLYISDENNHSSIIQGIRHSKARKEIWKHNDLKDLEGILMSNTGTKIVVFESIYSMDGDIAPVKEILELCKKYKAISYIDEVHAVGMYGETGAGICEREGCQPDIINGTLAKAFGVQGGYIAGKKDFIDAIRSLSSAFIFTTSLSPVICAGALTSIKYVKDHPELREKLHERSKTTKEELKRQGIEVMDNDSHIVPVMIGDAKKCKAVSDELLYKFGIYVQPINFPTVPVGTERLRFTPTPHHQDAHIFDMVLKVKSAIRRCGKKK

>SAMEA2619888_2008

MIELDKFTKVIDELKDDGRYRVFNDILRTAGNFPEAIWYSKYSIKKIVNWCANDYLGMGQHSYVIDSMKTALETAGAGAGGTRNISGTTHYHVALELELAKLHKKESALLFTSAYNANQTTLETIPKVLGDVLYISDENNHSSIIQGIRHSKARKEIWKHNDLKDLEGILMSNTGTKVVVFESIYSMDGDIAPVKEILELCKKYKAISYIDEVHAVGMYGETGAGICEREGCQPDIINGTLAKAYGVQGGYIAGKKDFIDAIRSLASAFIFTTSLSPVICAGALTSIKYVKDHPELREKLHERSKTTKEELKRQGIEVMDNDSHIVPVMIGDAKKCKAVSDELLYKFGIYVQPINFPTVPVGTERLRFTPTPHHQDAHIFDMVLKVKSAIRRCGKKK

>SAMEA2619779_9150

MQHINEFTNKLNELKDDGRYRVFNDILRTAGDFPNAIWYSKYSIKKIVNWCANDYLGMGQHSYVIDAMKTALETTGAGAGGTRNISGTTHYHVALELELAKLHKKEAALLFTSAYNANQTTLETIPKILDNVLYISDEMNHSSIIQGIRHSKARKEIFKHNDLGDLESILQSNTGTKIVVFESVYSMDGDIAPVKEIVELCKRYNAISYIDEVHGVGMYGTNGAGICERDNVEVDIINGTLAKAYGVQGGYIAGKKDFIDAIRSLASAFIFTTSLSPVICAGALTSVKYVKDHPELREKLHERSNTTKEELKRHGINVMDNNSHIVPVMIGDAKKCKAVSDELLYKNGLYVQPINYPTVPVGTERLRFTPTPYHTDAHIYDMVLKVKSAIKRCGKKDSK

>Ga0163179_10002200|830865

MYELDKFTKVLDELKDDGRYRVFNDILRTAGNFPEAIWYSKYSIKKIVNWCANDYLGMGQHSYVIDSMKTALETAGAGAGGTRNISGTTHYHVALELELARLHKKESALLFTSAYNANQTTLETIPKVLGDVLYISDEMNHSSIIQGIRHSKARKEIWKHNDLKDLEGILMSNTGTKIVVFESVYSMDGDIAPVKEIVELCKKYNAISYIDEVHAVGMYGENGAGICERDNIDVDIINGTLAKAYGVQGGYIAGKKDFIDAIRSLASAFIFTTSLSPVICAGALTSIKYVKDHPELREKLHERSKTTKKILKDHNINVMDNDSHIVPVMIGDAKKCKAVSDELLYKFGIYVQPINYPTVPVGTERLRFTPTPYHTDAHIYDMVLKVKSAIRRCGKKK

>SAMEA2620894_17066

MIELDKFTKVLDELKDDGRYRVFNDILRTAGNFPEAIWYSKYSIKKIVNWCANDYLGMGQHSYVIDSMKTALETAGAGAGGTRNISGTTHYHVALELELARLHKKESALLFTSAYNANQTTLETIPKVLGDVLYISDEMNHSSIIQGIRHSKARKEIWKHNDLKDLEGILMSNTGTKIVVFESIYSMDGDIAPVKEILELCKKYNAISYIDEVHAVGMYGETGAGICEREGCQPDIINGTLAKAFGVQGGYIAGKKDFIDAIRSLSSAFIFTTSLSPVICAGALTSIKYVKDHPELREKLHERSKTTKEELKRQGIEVMDNDSHIVPVMIGDAKKCKAVSDELLYKFGIYVQPINFPTVPVGTERLRFTPTPHHKDAHIFDMVLKVKSAIRRCGKKK

>Ga0102963_1000701|666050

MQHINEFTNKLNELKTDGRYRVFNDILRTAGDFPNAIWYSKYSIKKIVNWCANDYLGMGQHSYVIDAMKTALETTGAGAGGTRNISGTTHYHVALELELAKLHKKEGALLFTSAYNANQTTLETIPKILDDVLYISDEMNHSSIIQGIRHSKARKEIFKHNDLGDLESILQSNTGTKIVVFESVYSMDGDIAPVKEIVELCKKYNAISYIDEVHGVGMYGTNGAGICERDNVDVDIINGTLAKAYGVQGGYIAGKKDFIDAIRSLASAFIFTTSLSPVICAGALTSVKYVKDHPELREKLHERSNTTKEELKRHGINVMDNNSHIVPVMIGDAKKCKAISDELLYKDGLYVQPINYPTVPVGTERLRFTPTPFHTDAHIYDMVLKVKSAIKRCGKKDVE

>Ga0068469_1063893|485779

VKHLEEFTKIINEYKEDGRYRVFNDIVRTRGNFPHAIWYSKYSIKKIVNWCSNDYLGMGQHSYVIDSMKTALESSGAGAGGTRNISGSTHYHNALETELADFHKKEKALIFTSAYNANQTTLETLGKIIPDLLYISDSLNHSSLIQGIRHSRCKKEIFKHNDVEDLERILKSYEGPKCVVFESVYSMDGDIGPVKEIVELAKKYNAITFLDEVHAVGLYGATGGGITERDNIEVDIINGTLAKAFGVQGGYIAGKKDFIDAIRSLASAFIFTTSLSPVICAGALTSIKYVRDHPELREQIHERANKTKLELARQGIEVMKNDSHIVPVIIGDPKRAKAISDELLYKEGIYVQPINWPTVPVGTERLRFTPTPFHTDALIFDMVVKLKAAMKKCGGKSAIQS

>Ga0068471_1051077|485885

VKHLEEFTKIINEYKEDGRYRVFNDIVRTRGNFPHAIWYSKYSIKKIVNWCSNDYLGMGQHSYVIDS

MKTALESSGAGAGGTRNISGSTHYHNALETELADFHKKEKALIFTSAYNANQTTLETLGKIIPDLLYISDSLNHSSLIQGIRHSRCKKEI

FKHNDVEDLERILKSYEGPKCVVFESVYSMDGDIGPVKEIVELAKKYNAITFLDEVHAVGLYGATGGGITERDNIEVDIINGTLAKAFGV

QGGYIAGKKDFIDAIRSLASAFIFTTSLSPVICAGALTSIKYVRDHPELREQIHERANKTKLELARQGIEVMKNDSHIVPVIIGDPKRAK

AISDELLYKEGIYVQPINWPTVPVGTERLRFTPTPFHTDALIFDMVVKLKAAMKKCGGKSAIQS

>Ga0068480_1108826|488449

VKHLEEFTKIINEYKEDGRYRVFNDIVRTRGNFPHAIWYSKYSIKKIVNWCSNDYLGMGQHSYVIDSMKTALESSGAGAGGTRNISGSTHYHNALETELADFHKKEKALIFTSAYNANQTTLETLGKIIPDLLYISDSLNHSSLIQGIRHSRCKKEIFKHNDVEDLERILKSYEGPKCVVFESVYSMDGDIGPVKEIVELAKKYNAITFLDEVHAVGLYGATGGGITERDNIEVDIINGTLAKAFGVQGGYIAGKKDFIDAIRSLASAFIFTTSLSPVICAGALTSIKYVRDHPELREQIHERANKTKLELARQGIEVMKNDSHIVPVIIGDPKRAKAISDELLYKEGIYVQPINWPTVPVGTERLRFTPTPFHTDALIFDMVVKLKAAMKKCGGRSAIQS

>Ga0068481_1090348|488670

VKHLEEFTKIINEYKEDGRYRVFNDIVRTRGNFPHAIWYSKYSIKKIVNWCSNDYLGMGQHSYVIDSMKTALESSGAGAGGTRNISGSTHYHNALETELADFHKKEKALIFTSAYNANQTTLETLGKIIPDLLYISDSLNHSSLIQGIRHSRCKKEIFKHNDVEDLERILKSYEGPKCVVFESVYSMDGDIGPVKEIVELAKKYNAITFLDEVHAVGLYGATGGGITERDNIEVDIINGTLAKAFGVQGGYIAGKKDFIDAIRSLASAFIFTTSLSPVICAGALTSIKYVRDHPELREQIHERANKTKLELARQGIEVMKNDSHIVPVIIGDPKRAKAISDELLYKEGIYVQPINWPTVPVGTERLRFTPTPFHTDALIFDMVVKLKAAMKKCGGKSAIQS

>SAMEA2619974_635

MIHLNKFQDVIEGLKEEGNYRVFNDILRERGKFPQAIWYSKYAVKRIVNWCSNDYLGMGQHRYVIDAMQTALESSGAGSGGTRNISGTTHYHVALEHELAELHQKEAALLFTSAYIANSATLESMAKVLPDITFISDSMNHASIIQGIRHSRAKKEIWKHNDLKHLKELLEKTPDPKCVVFESVYSMDGDICPLKEIVTLCKKYKAITFLDEVHAVGLYGERGGGITERDGVDVDIINGTLAKAFGVQGGYIAADKDFLDAIRSLSSGFIFTTSISPVICAGALTSVKYVRDHNCLRLTMQERSQKTKVELERNGISVMENPSHIVPVIIGDAKICKRISDDLLYKDGIYVQPINYPTVAKGTERLRFTPGPFHTDLMLYDMVIKLRDAMRHNKIV

>Ga0115002_10003437|755546

MKHLEEFTKIIDEYKQDGRYRVFNDIVRTRGNFPHAIWYSKYSIKKIVNWCSNDYLGMGQHSYVIDSMKTALESSGAGAGGTRNISGSTHYHNALEDELADFHKKEKALVFTSAYNANQTTLETLGKIIPDLLYISDSLNHSSLIQGIRHSRCKKEIFKHNDVEDLERILKSYEGPKCVVFESVYSMDGDIGPVKEINELAKKYNAITFLDEVHAVGLYGATGGGITERDNIDVDIINGTLAKAFGVQGGYIAGKKDFIDAIRSLASAFIFTTSLSPVICAGALTSIKYVRDHPELREQIHERANKTKEELARQGIEVMKNDSHIVPVIIGDAKKCKAISDELLYKEGIYVQPINWPTVPVGTERLRFCPGPFHTDALIFDMVVKLKAAMKKIMISN

>Ga0114997_10001596|717177

MEHLDKFKKIITELKADGRYRVFNDILRTRGSYPNAIWYSKYSIKKIVNWCSNDYLGMGQHSYVIDSMKTALETSGAGAGGTRNISGTTHYHIALEHELTQLHDKESALLFTSAYNANQTTLETMGKIMPEILFISDAENHSSIIQGLRHSKCKKEIFKHNDLDDLESILKSNPGPKCVVFESVYSMDGDIAPVKEIADLCKKYKAISYIDEVHGVGLYGPKGAGICERDNVDVDIINGTLAKAYGVQGGYICGKREFIDAIRSMASAFIFTTSLSPVLCAGALTSIKYVKDHPELREQLQERARKTKEELTRQGIEVLQNDSHIVPVIIGDAKKCKAVSDELLYKDGIYVQPINYPTVAVGTERLRFTPTPFHTDMMIFDMVVKVKSAMRRCGKTK

>Ga0114994_10001250|715058

MEHLDKFKKIITELKADGRYRVFNDILRTRGSYPNAIWYSKYSIKKIVNWCSNDYLGMGQHSYVIDSMKTALETSGAGAGGTRNISGTTHYHIALEHELTQLHDKESALLFTSAYNANQTTLETMGKIMPEILFISDAENHSSIIQGLRHSKCKKEIFKHNDLDDLESILKSNPGPKCVVFESVYSMDGDIAPVKEIADLCKKYKAISYIDEVHGVGLYGPKGAGICERDNVDVDIINGTLAKAYGVQGGYICGKREFIDAIRSMASAFIFTTSLSPVLCAGALTSIKYVKDHPELREQLQERAQKTKEELTRQGIEVLQNDSHIVPVIIGDAKKCKAVSDELLYKDGIYVQPINYPTVAVGTERLRFTPTPFHTDMMIFDMVVKVKSAMRRCGKTK

**Heme Oxygenase (HemO) protein**

>MOSIG

MLDIKELTLEQHKNAERQEFVKILMSGNIDHKLYATYLYNQFQCYSVLEKYGLHNSLFRDTPGLLRAEHILYDFKSFEIETPEITDSTKEYIEHIESIQDEAMKLYAHIYVRHMGDLSGGQMIRRKTPGPNRYYKFRDKEVGDYRRIVKETINTYLNVYEHSVVPEAIYCFESATKLFKEMKELHDLG*

>Marinovum

MKTLKELTWEHHKEAERQKFVKVLMSGKINPEIYAIYLANQHKTYDVLEAMAMADGLLDDMPEIRRAPRIKKDFDELWTYSWQPVIFPTTEKYIKYVAETLMDCPEKIIAHIYVRHMGDLSGGQMIKRKIPGAGTMYDFNFRYKDGDGSKKFQTIEEMKSALRLKVDSFQKYSDASTITENVNNVVYEARTCFNFATLLFKDIDKFINDNEKRFGDGTEK.

>SAMEA2620861_1137947

MKTLKELTWEHHKEAERQGFVKILMSGKIHPEVYANYLFNQHQCYNILEPLAMAEGLLDKFPFIRRAPAIKADFDELWTYAHQPMMMESTRKYVDYANKELMDCPEKIMAHIYVRHMGDLSGGQMIKRKVPGLGKYYEFKFNKNPEHGITYKDTNEIKEALRLAVDSHYVYNDASDKDKNVNNVVYEARQCFGFATDLFKEMLQFIKNNEKRFGDGTTK*

>SAMEA2622841_6527

MKTLKELTWEHHKEAERQKFVKVLMSGKINPEIYAIYLYNQHQCYDMLEALAMSEGIFDDMPEIRRAPSIKADFDELWTYNWQPVKMESTGAYLDYINKNLMDNPEKIAAHIYVRHMGDLSGGQMIQRKIPGQGKYYQFNIRYVEGRNQKYKNIQELKEALRTKVNSYQKYSDQSTLTENINSVVYEARQCFGFATDLFKDIYKFIEQNEKRFGDGTIQTK*

>SAMEA2620861_60095

MKTLKELTWEHHKEAERQKFVKVLMSGKIIPEIYAIYLANQHKTYDVLEAMAMADGLLDDMPEIRRAPHIKKDFDELWTYGWAPPIFPTTEKYIKYVAEALMDCPEKIMAHIYVRHMGDLSGGQMIKRKIPGAGTMYDFNLRYVEGRNQKYQNMDELKEALRTKVNSFQKYSDASTLTENVNNVVYEARTCFNFATNLFKDIDKFINDNEKRFGNGTEK*

>SAMEA2620861_551347

MKTLKELTWEHHKEAERQKFVKVLMSGKINPEIYAIYLYNQHQCYDMLEALAMSEGIFDDMPEIRRAPSIKADFDELWTYNWQPVKMESTGAYLDYINKNLMDNPEKIAAHIYVRHMGDLSGGQMIQRKIPGQGKYYQFNIRYVEGRNQKYKNIQELKEALRTKVNSYQKYSDQSTLTENINSVVYEARQCFGFATDLFKDIQKFIEQNEKRFGDGTIQTK*

>SAMEA2620413_422904

MKTLKELTWEHHKEAERQGFVKILMSGKIHPEVYANYLFNQHQCYNILEPLAMAEGLLDKFPFIRRAPAIKADFDELWTYAHQPMMMESTRKYVDYANKELMDCPEKIMAHIYVRHMGDLSGGQMIKRKVPGLGKYYEFKFNKNPEHGITYKDTNEIKEALRLAVDSHYVYNDASDKDKNVNNVVYEARQCFGFATDLFKEMLQFIKNNEKRFGDGTTK*

>3300009785_Ga0115001_100000

MKTLKELTWEHHKEAERQQFVKVLMSGKILEEVYAVYLYNQHQAYNILEAVAMSEGFFDDMPQLRRAPEILKDFNELWTWDHKPWLCESTKKYIDHCQTLMDSPEKIAAHIYVRHMGDLSGGQMIRRKTPGRNYYYDFNFKKVDDGVQKYKSVQELKDALRLKVDSYQKYSDASTLTENVNNVVYEARVCFSFATELFKEMMTFINNNEKRFGDGTKK*

>3300003620_JGI26273J51734_10000260|386802

MKTLKELTWEHHKEAERQHFVKVLMSGKILEEVYAVYLYNQHQAYNILEAVAMADGFFDDMPQLRRAPEILKDFNELWTWKHKPWLCESTKKYVEYVNKNLMDNPEKIAAHIYVRHMGDLSGGQMIKRKTPSRNYYYDFNFKKVDDGVQKYKSVQEIKDALRLKIDSFQKYSDASTLTENVNNVVYEARVCFSFATELFKEMMTFINNNEKRFGDGTKK*

>3300009790_Ga0115012_10000076|761838

MKTLKELTWEHHKEAERQHFVKVLMSGKILEEVYAVYLFNQHQAYNILEAIAMSEGFFDDMPQLRRAPEIKKDFDELWTWNHKPWLCESTKRYVYHCQNELMDSPEKIAAHIYVRHMGDLSGGQMIKRKTPGSNKYYDFNFKKIDDGVQRYKSVQELKDALRLKVDSYQKYSDASTLTENVNNVVYEARVCFSFATELFKEMMSFIKNNEKRFGDGTKK*

>3300009593_Ga0115011_10000306|738531

MKTLKELTWEHHKEAERQHFVKVLMSGKILEEVYAVYLFNQHQAYNILEAIAMSEGFFDDMPQLRRAPEIKKDFDELWTWNHKPWLCESTKRYVYHCQNELMDSPEKIAAHIYVRHMGDLSGGQMIKRKTPGSNKYYDFNFKKVDDGVQRYKSVQELKDALRLKVDSYQKYSDASTLTENVNNVVYEARVCFSFATELFKEMMSFIKNNEKRFGDGTKK*

>3300006166_Ga0066836_10000757|480248

MKTLKELTWEHHKEAERQGFVKTIMSGKINPEIYGIYLFNQHQCYNMLEALAMSEGIFDDMPELRRAPSIKADFDELWTYNWKPPLMESTSKYLDYINKNLMDNPEKIAAHIYVRHMGDLSGGQMIRKKIPGQGKYYQFNIRYVEGRNQPYKNIKELKEALRTKVDSYQKYSDQSTISENINSVVYEARICFGFATDLFKDMKKFIEQNEKRFGDGNL*

>3300005521_Ga0066862_10001059|417910

MKTLKELTWEHHKEAERQGFVKTIMSGKINPEIYGIYLFNQHQCYNMLEALAMSEGIFDDMPELRRAPSIKADFDELWTYNWKPPLMESTSKYLDYINKNLMDNPEKIAAHIYVRHMGDLSGGQMIRKKIPGQGKYYQFNIRYVEGRNQPYKNIKELKEALRTKVDSYQKYSDQSTISENINSVVYEARICFGFATDLFKDMKKFIEQNEKRFGDGNL*

>SAMEA2621173_96 /station=TARA_072 /depth=100 /filter=0-0.22

MTLSTELKTGTKKSHSAAENTKFVASFLRGVVDPEEYRKLIANFFFVYRAMEEEISKHHDDVAVAVIDDPALYRTQALEKDLAYYYGDNWRSIIQPTEACQQYVNRIREVDKMLLIGHHYTRYIGDLSGGQILKGIAEKTLTLPEGEGLHFYDFNEIEDAKDYKNEYRATLDALELDQSQIDAIISEANYAFKLNMYMFDEIQGNAGKSLWTLFTNWIKGIFK

>uncultured_Med_phage_BAR36618 gi|787065978|dbj|BAR36618.1|:3-130 Heme oxygenase like protein [uncultured Mediterranean phage uvMED]

DIKELTWEHHKNAERQAFVKILMSGEIDEKLYATYLYNQLQCYSVLEKYGLHNSLFRDTPNLLRAEHIHYDYKSLWQDVDNPPKITQSTKDYVAHIETIQDEAMKLYAHVYVRHMGDLSGGQMIRKKT

>uncultured_Med_phage_BAR36229 gi|787065515|dbj|BAR36229.1|:7-135 Heme oxygenase like protein [uncultured Mediterranean phage uvMED]

YRIKDLTWEYHKNAERQDFVKLLLSGSIDEKLYATYLYNQLICYGKLEEYCLESSLFHDTLNLPRAPHIFYDYRALWGDIGSPPVQTESTKAYVEHLEAIRGENEKLYAHVYVRHLGDLSGGQMIMRKT

>Deinococcus_peraridilitoris gi|505049414|ref|WP_015236516.1|:5-121 heme oxygenase [Deinococcus peraridilitoris]

RLKEHTQAQHERVEALVRVMDDPLTLQQYREVLGSMAGFYLPLEAQLSDLDLPQVFRFEARRKSALLRRDLQVLGLSQTAHAARAQLPALSTVAHALGCLYVLEGATLGGRIIARHV

>Paenibacillus_daejeonensis gi|522105792|ref|WP_020617001.1|:10-134 hypothetical protein [Paenibacillus daejeonensis]

RLKDETAPMHEQIEENEYATAIMNNHLTMDQYKAYLVKFYGFIKPLEIRFEDVEQPPGSALTEPSRNKTAWLEHDLMALGLDRTALDTVPQCDSLPDVSTRARALGCLYVLEGSTLGGQMITKKL

>ECF09659

AQLREGTKKSHTMAENTGFVSCFLKGVVDKASYRKLVADLYFVYSAMEEEIGKLTDHPVVGPVAMAQLNRREALEQDLTYYFGDNWNEEIKPSPSAAAYVERIHAVAQESPELLVGHHYTRYLGDLSGGQILKNIA

>WH8109

AQIREGTKKSHTMAENTGFVSCFLKGVVDKASYRKLVADLYFVYSAMEEEIGKLTDHPVVGPVAMAQLNRREALEQDLTYYFGDNWKAEIKPSPSAAAYVERIHAVAQESPELLVGHHYTRYLGDLSGGQILKNIA

>EBD94673

AQLREGTKKSHTMAENTGFVSCFLKGVVDKASYRKLVADLYFVYSAMEDEISNLADHPVVGPVAMAQLNRREALEQDLTYYFGDSWKENIQPSPSAAAYVERIHAVAQESPELLVGHHYTRYLGDLSGGQILKNIA

>RS9916

AQLREGTKKSHTMAENTGFVSCFLKGVVDKASYRKLVADLYFVYTAMEEEIAKLGDHPVVGPVGMKELNRSEALEQDLTYYFGANWKDEIKPSPSAAAYVERIHAVAQESPELLVGHHYTRYLGDLSGGQILKNIA

>WH8102

TQLREGTKKSHTMAENTGFVSCFLKGVVDKASYRKLVADLYFVYTAMEEEISKLGDHPVVGPVGMQELNRRDALEQDLVFYFGAGWKDQIQPSPSAAAYVERIHAVAQESPELLVGHHYTRYLGDLSGGQILKNIA

>EDA94704

TQLREGTKKSHTMAENTGFVSCFLKGVVDKASYRKLVADLYFVYTAMEEEISKLGDHPVVGPVGMQELNRRDALEQDLVFYFGAGWKDQIQPSPSAAAYVERIHAVAQESPELLVGHHYTRYLGDLSGGQILKNIA

>ECY98753

AQLREGTKKSHTMAENTGFVSCFLKGVVDKASYRKLVADLYFVYTAMEEEIAKLGDHPVVGPVGMQELNRREALEQDLVYYFGAGWKDQIQPSPSAAAYVERIHAVAQESPELLVGHHYTRYLGDLSGGQILKNIA

>EBE89898

AQIREGTKKSHTMAENTGFVSCFLKGVVDKSSYRKLVADLYFVYEAMEEEIDKLGDHPIVGPIGKKELNRRESLEQDLTYYFGAGWKEQIQPSPSAAAYVERIHAVAKESPELLVGHHYTRYLGDLSGGQILKNIA

>EDC84330

AQLREGTKKAHTMAENTGFVSCFLKGVVDKSSYRKLVADLYFVYSAMEEEVGQLKDHPVVGPVGMEQLNRRESLEKDLVYYFGENWNDEIQPSPSAVAYVERIREVAKESPELLVGHHYTRYMGDLSGGQILKNIA

>WH8016

TQLREGTKKSHTMAENTGFVSCFLKGVVDKLSYRKLVADLFFVYEAMEEEMHRLKDHPVLAPIAFEQLDRVTALEEDLAFYFGPEWRQQIEASPAATEYVARIREVAQTAPELLVGHHYTRYLGDLSGGQILKNIA

>ECW35071

AQLREGTKKSHTMAENTGFVSCFLKGVVDKGSYRTLVADLYVVYSAMEEEMARLSGHPVLAPIAFPELNRREALEQDLTYYYGSDWAQVVKATPAAEAYVARIRQVAQDSPELLVGHHYTRYLGDLSGGQILKTIA

>MIT9303

SQLREGTKAAHTMAENTGFVSCFLKGVVDQSSYRMLVADLYFVYSALEAEIGKLREQKHPVVAPVGFPELNRCEALEQDLIFYFGSNWRNLAKATLAAEEYVARIHTLAQESPELLVAHHYTRYLGDLSGGQILKTIA

>MIT9313

SQLREGTKAAHTMAENTGFVSCFLKGVVDQSSYRMLVADLYFVYSALEAEIGKLREQKHPVVAPIGFPELNRCEALEQDLIFYFGSDWRNLAKATLAAQEYVARIHKLAQESPELLVAHHYTRYLGDLSGGQILKTIA

>MIT9211

TQLREGTKKSHTMAENTGFVTCFLKGVVDKSTYKNLLADLYLVYSVMEDEVGRLCKEGHPVIAPIGFNELNRRESLEKDLKFYCGNNWLDVIKATPSANAYADRIQNIARESPELLIGHHYTRYIGDLSGGQILKKIA

>EBV15045

GQLREGTKKSHTMAENTGFVACFLKGVVEKKSYRKLISDLYFVYEAMEEEIERLVNEEHPVIKPIGFKSLFRKETLENDLKFYFGDNWKNEINISHSAKEYVERIHEVAKNSPELLVGHHYTRYIGDLSGGQILKRIA

>ECV49915

GQLREGTKKSHTMAENTGFVACFLKGVVEKKSYRKLISDLYFVYEAMEEEIERLVKEEHPVIKPIGFKSLFRKETLENDLKFYFGDNWKNEINISNSAKEYVERIREVAKNSPELLVGHHYTRYIGDLSGGQILKRIA

>EDE71868

GQLREGTKKSHTMAENTGFVACFLKGVVEKKSYRKLISDLYFVYEAMEEEIERLVTEDHPVIKPIGFKSLFRKETLENDLKFYFGDNWKNEINISQSAKEYVERIKEVAKKSPELLVGHHYTRYIGDLSGGQILKRIA

>MIT9312

GQLREGTKKSHTMAENTGFVACFLKGVVEKKSYRKLISDLYFVYEAMEEEIERLVHEEHPVIKPIGFKSLFRKKTLENDLKFYFGDNWKNQINISQSAKEYVERIRSVAKNSPELLVGHHYTRYIGDLSGGQILKRIA

>CCMP1986

GQLREGTKKSHTMAENTGFVACFLKGVVEKKSYRKLISDLYFVYEAMEEEIERLVQEEHPVIKHIGFKSLFRKETLENDLKFYFGDNWQKEINISKSAKEYVNRIHLVAKKSPELLVGHHYTRYIGDLSGGQILKKIA

>EDD66840

KKIKKDTSKSHSMAENTGFVTNFLAGVVSKESYKQLIADFYFIYTALEEQVEKFKDDPFIAPIAFDELKRVPALEKDCEFYWGENWKNTISPTDACKNYVKRVKKINAKFLVGHHYTRYLGDLSGGQILKNIA

>EBV38795

KKIKKDTSKSHSMAENTGFVTNFLAGVVSKESYKQLIADFYFIYTAIEEQVEKFKDDPFIAPIAFDELKRVPALEKDCEFYWGENWRNIISPTDACKNYVKRVKKINAKFLVGHHYTRYLGDLSGGQILKNIA

>SSSM6a

KAIKKGTQKSHTMAENTGFVTNFLAGVVDKESYKQLIADFFFIYTALEEQIDEFKDDSFIRPIAFDELRRVPALEKDCEFYWGENWRDTISPTDACKNYVNRVKKINAKFLVGHHYTRYLGDLSGGQILRNIA

>PHM1

ADLKEGTKKSHSAAENTKFVAGFLRGVVDEESYRKLIQDFYFIYSALEEEMERLEDDNFLSPINFSELDRVKSLKKDLRYYYGPNWNQTIKPSQACVQYVERIHEVADSNEPYLLVGHHYTRYLGDLSGGQILKTIA

>Phage_MED4_213

ADLKEGTKKSHSAAENTKFVAGFLRGVVDEESYRKLIQDFYFIYSALEEEMERLEDDNFLSPINFSELDRVKSLKKDLRYYYGPNWNQTIKPSQACVQYVERIHEVADSNEPYLLVGHHYTRYLGDLSGGQILKTIA

>PHM2

ADLKEGTKKSHSAAENTKFVAGFLRGVVDEESYRKLIQDFYFIYSALEEEMERLEDDNFLSPINFSELDRVKHLKKDLRYYYGPNWNQTIKPSQACIQYVERIHEVADSNEPYLLVGHHYTRYLGDLSGGQILKTIA

>ECH63529

KQLKEGTKKSHTMAENTSFVASFLRGVVDESKYRQLIANFYFIYHALESEMDLNKDNPFVGPMRLNGLERHDALVKDCQYFYGDDWKDIIRPTEETQKYVSRIHEVAKNNPELLIAHHYTRYMGDLSGGQILKGIA

>EDC11829

KQLKEGTKKSHTMAENTSFVASFLRGVVDESKYRQLIANFYFIYHALESEMDLNKDNPFVGPMRLNGLARHDALVKDCQYFYGDDWKDIIRPTEETQKYISRIHEVAKNNPELLIAHHYTRYMGDLSGGQILKGIA

>EBE36436

KQLKEGTKKSHTMAENTSFVSSFLRGVVDEISYRQLVANFYFIYHALESEMKLHKDHEYVGRIALDGLARHDALANDCKYFYGYKWQDTIRPTEQAQRYVSRIHEVGNKTPELLIAHHYTRYMGDLSGGQILKGIA

>EDG64588

AQIKLGTKKSHSMAENTSFVTSFLRGVVSEESYRTLVSNLYFVYTALEDVAEHLKDNDEVSPILFDELKRHKALAKDLDYFYGEGWHENIYPSDATKRYIDRIREVGRQEPYLFIGHHYTRYMGDLSGGQILKGIA

>EBH14149

KQIKVGTKKSHSAAENTKFVASFLRGVVDKESYRTLVANLYFVYSALEDVAGHLKDNPEVSPILSDALNRHDALVKDLNYFYGEGWHETIYPSPATKQYIERIREVGRGDHQYLFVGHHYTRYMGDLSGGQILKGIA

>PSSM2

VQIKEGTKKSHSAAENTSFVASFLRGVVSKESYKALVKDLYYVYRTLEEEFEKHKDHPVVGKLYLPELNRVNALERDLRFYYGPIWRSLIMPSEACANYVSRIKCCSIEDPTLLVGHHYTRYLGDLSGGQILKGIA

>EDD31396

KELKVGTKKSHTAAENTAFVKSFLRGVVSPDNYKCLVSDLYFVYKALEEEVYGLRTHPIVGSLYFPELERLIALEKDLQYFYGLEWRTKIEPSQACKQYVNRIREVAEDEPELLVGHHYTRYLGDLSGGQILKNIA

>EDD96572

LDIKEITMEHHKNAERQAFVRILMSGNIDEKLYATYLYNQFQCYSVLEKYGLHNSLFRDTPGLLRAEHIHYDYRSLWTDIGSPPEITQSTKDYIAHIESIQDEAMKLYAHIYVRHLGDLSGGQMIMKKT

>EDB17286

LDIKDLTLEHHKNAERQEFVRILMSGNIDEKLYATYLYNQLQCYSVLEKYGLHNSLFRDTPGLLRAEHIHYDYRHLWTDIGAPPEITQSTKDYIAHIESIQDEAMKLYAHIYVRHMGDLSGGQMIMKKT

>EBH01735

LDIKELTWEHHKNAERQAFVKILMSGQIDERLYATYLYNQLQCYSVLEKYGLHNSLFRDTPGLLRAEHIHYDYRSLWTDIGAPPEITQSTKEYIEHIESIQDEAMKLYAHVYVRHLGDLSGGQMIMKKT

>ECB38890

LDIKELTWEHHKNAERQEFVKILMSGKIDEKLYATYLYNQLQCYSVLEKYGLHNSLFRDTPNLLRAEHIHYDYKSLWQDTDNPPLITQSTRDYVEHIEKIQDEAMKLYAHVYVRHMGDLSGGQMIRKKT

>EBK42634

YKITELTWEYHKNAERQDFVKILLSGEIDERLYATYLYNQLACYSKLEEYCLESSLFMDTKNLPRAPHIHYDYTHLWTDIGSPPELTESTKAYVEHLDTIRGENEKLYAHVYVRHLGDLSGGQMIMRKT

>New_JCVI_SCAF_1096626956256

YDIKELTKEIHQKAERQEFVKTLLSGTIRPELYAIYLYNQLQCYSVLEKYGMHNDLFRQTPGLQRAENISKDYLKLWPDPKQPPRITESTKKYIEHIESIQDDPEKLYAHIYVRHLGDLSGGQMIAKKV

>EBV08642

YDIKELTKEIHQNAERQEFVKTLMSGTIRPELYAIYLYNQLQCYSVLEKYGMANDLFRQTPGLQRAENIHRDYKKLWPDTSNPPKITDSTKRYVEHIETIQDDPEKLYSHIYVRHLGDLSGGQMISKKV

>EBO67495

HDIKELVWEEHKNAERQEFVKILMSGEINSELYATYLYNQLQCYAELEKWGNHNGLFRQTPGLQRAENIHKDFTKLWTKSEKPMITQSTKEYIEHINTITDDPEKLYAHIYVRHLGDLSGGQMIAKKV

>ECK95789

YDIKDLVWEEHKNAERQEFVKTLMSGTIEKELYATYLYNQLQCYAELEKWGHHNGLFRHTTNLPRAEYIHRDYLKLWTDVGMPPNITESTKEYVEHIKTITDEPEKLYAHVYVRHMGDLSGGQMISKKV

>actinobacterium_SCGC_AAA041_L13

MSLKEITQDLHREAERTEFAKLLLSGKITKKDYANYLYQMIIIYASIEVGNRNNGFLVNLPGIERTEAIRSDFVELIENDLTRYEYLPETIAYQEYLSELCDDPTRQHLIKAHMYCRHMGDLFGGQIIANRV

>Bradyrhizobium_ORS278

LRLRNETRAAHESIEKNGCLKRLLADDLTRSEYAGILRRLTAFYVPVEREWLRWAEQLPAALELEARAGKAALLFADLAALQAPAPDRAAYADFAPFDAVEQAWGCIYVIEGSTMGGQLIARSL

>SAMEA2619974_635

MMRGYIMNKRTDNLRSLTMKIHHKSERHEIAEQMLKGDIDPETYAVYLWNLYQIYVVLENAALSLDLINQDTIRSSNLMFDFQELWDKDFPPPTCKTTIKHKDRIIENISDPTVIMAHVYVRHMGELHGGQILKNQVPGSGKFYEFDDAKTAIAEIVSKLDDSMYDEAVAVFKLSNELLDEVLEYQQQNNLGGE

>Ga0115002_10003437|755546

MYDIKELTKDIHQNAERQEFVKTLMSGSIEPSLYATYLYNQLQCYAVLEKYGIENSLFRTTPNLPRAEHLHYDYKALWTSEELPTVTQSTKNYIEHIESIKEDAEKLYAHIYTRHLGDVSGGQMIMKRTPGPNRYYKFKHGEIKEYKRIVKETINSYLNVYKLNILNEVKFCFASATQLFKEMNDMDYSKPLILTNEVITKDTENDPFKGTSIEGKD

>Ga0098058_1000146|507114

MMRGYIMNKRTDNLRSLTMKIHHKSERHEIAEQMLKGDIDPETYAVYLWNLYQIYVVLENAALSLDLINQDTIRSSNLMFDFQELWDKDFPPPTCKTTIKHKDRIIENISDPTVIMAHVYVRHMGELHGGQILKNQVPGSGKFYEFDDAKTAIAEIVSKLDDSMYDEAVAVFKLSNELLDEVLEYQQQNNLGGE

>Ga0115007_10001227|723900

MYDIKELTKDIHQNAERQEFVKTLMSGSIEPSLYATYLYNQLQCYAVLEKYGIENSLFRTTPNLPRAEHLHYDYKALWTSEELPTVTQSTKNYIEHIESIKEDAEKLYAHIYTRHLGDVSGGQMIMKRTPGPNRYYKFKHGEIKEYKRIVKETINSYLNVYKLNILNEVKFCFASATQLFKEMNDMDYSKPLILTNEVITKDTENDPFKGTSIEGKD

>Ga0114999_10006015|760631

MYDIKELTKDIHQNAERQEFVKTLMSGSIEPSLYATYLYNQLQCYAVLEKYGIENSLFRTTPNLPRAEHLHYDYKALWTSEELPTVTQSTKNYIEHIESIKEDAEKLYAHIYTRHLGDVSGGQMIMKRTPGPNRYYKFKHGEIKEYKRIVKETINSYLNVYKLNILNEVKFCFASATQLFKEMNDMDYSKPLILTNEVITKDTENDPFKGTSIEGKD

>Ga0163179_10002200|830865

MYDIKELTKDIHQNAERQEFVKTLMSGSIEPSLYATYLYNQLQCYAVLEKYGIENSLFRTTPNLPRAEHLHYDYKALWTSEELPTVTQSTKNYIEHIESIKEDAEKLYAHIYTRHLGDVSGGQMIMKRTPGPNRYYKFKHGEIKEYKRIVKETINSYLNVYKLNILNEVKFCFASATQLFKEMNDMDYSKPLILTNEVITKDTENDPFKGTSIEGKD

>Ga0068469_1063893|485779

MYDIKELTKDIHQNAERQEFVKTLMSGSIEPRLYATYLYNQLQCYAILEKYGIENSLFRTTPNLPRAEHLHYDFKALWTSEDLPTVTQSTKDYVAHIETIKEDAEKLYGHIYTRHLGDVSGGQMIMKRTPGPNRYYKFKHKEIKEYKRIVREMINSYLNVYKLNILNEVKFCFATATQLFKEMNDMDYSKPLILTNEVKDDLGTTN

>Ga0114997_10001596|717177

MKTLKELTWEHHKEAERQQFVKVLMSGKILEEVYAVYLYNQHQAYNILEAVAMSEGFFDDMPQLRRAPEILKDFNELWTWDHKPWLCESTKKYIDHCQTLMDSPEKIAAHIYVRHMGDLSGGQMIRRKTPGRNYYYDFNFKKVDDGVQKYKSVQELKDALRLKVDSYQKYSDASTLTENVNNVVYEARVCFSFATELFKEMMTFINNNEKRFGDGTKK

>Ga0114994_10001250|715058

MKTLKELTWEHHKEAERQQFVKVLMSGKILEEVYAVYLYNQHQAYNILEAVAMSEGFFDDMPQLRRAPEILKDFNELWTWDHKPWLCESTKKYIDHCQTLMDSPEKIAAHIYVRHMGDLSGGQMIRRKTPGRNYYYDFNFKKVDDGVQKYKSVQELKDALRLKVDSYQKYSDASTLTENVNNVVYEARVCFSFATELFKEMMTFINNNEKRFGDGTKK

>LP_A_09_P20_500DRAFT_1000343|289042

MKTLKELTWEHHKEAERQHFVKTLMSGKINPEIYAVYLFNQHQCYNIVESLAMSEGILDDMPELRRAKFIKADFDELWTYKWQPAMMTSTKKYLDHCNENLMDNPDKIAAHIYVRHMGDLSGGQMIKSKVPGQGTYYNFNIRYVEGRRQLYKNIKELEEALKVKVNSYQKYSDQSTLTENINSVVYEARMCFGFATELFKDMQKFIKDNEKRFGDGLLS

>GROS21-1_SAMEA9560540

MSLKELTKDSHTAAEATPFMQAVFKKRMPVKTWGDYTFQKSAIYNTIENVCRYNKLTHDILDIERSVHLYLDAQEIMGDMSAVRMRPVTGEYVRYLLSLVDEPDRILAHLYVWHMGDLHGGQMIKKVLPPPHRNLDFADIEGLKVAIRAKLNDSMADEANIAFEWAMKLMNTYDADFV*

>GROS21-1_SAMEA9560542

MSLKDLTKEKHRDAERCAFSQKLISGDMSVHDYANYLVQMGYVYQILENKAYTHNLTQDLPGIARAFAIQQDIVELVGEDHGIAYLPSSLKYGQYLESLSDPGAIMAHLYVRHMGDLFGGQIIAKKVPGSGKFYQFPDRERLIFLVRAKITDDLADEALVAFDCSISIMKELMSE*

>GROS21-1_SAMEA9560546

MSLKEITADLHDRAENTPFMKAVFAKTLPMEIWTEWTYWKLLFYYQIEQKCDEGGLLADLQGIKRCEGLWKDFLELRNGDESPTANCINNVLVSYENHIKSLTPEQALAHLYVWHMGDLFGGQMIKKIVPAPSHHALEFEDANTLKNNLRAKLTDDLGPEARLAFEYAIKMMEALYSE*

>GROS21-1_SAMEA9560586

MSLKEITADLHDRAENTPFMKAVFAKTLPMEIWTEWTYWKLLFYYQIEQKCDEGGLLADLQGIKRCEGLWKDFLELRNGDESPTANCINNVLVSYENHIKSLTPEQALAHLYVWHMGDLFGGQMIKKIVPAPSHHALEFEDANTLKNNLRAKLTDDLGPEARLAFEYAIKMMEALYSE*

>GROS21-1_SAMEA9560612

MSLKEITADLHDRAENTPFMKAVFAKTLPMEIWTEWTYWKLLFYYQIEQKCDEGGLLADLQGIKRCEGLWKDFLELRNGDESPTANCINNVLVSYENHIKSLTPEQALAHLYVWHMGDLFGGQMIKKIVPAPSHHALEFEDANTLKNNLRAKLTDDLGPEARLAFEYAIKMMEALYSE*

>GROS21-1_SAMEA9560623

MSLREITKDLHKEAERSIFAKKLIDGSISVEEYANYLWQMVLVYNGIETAANSQKMLINLPGIERTHKIYQDCIELVGTHHQLKWLPETIEYYQYLLALNYDQERKHLVKAHMYCRHMGDLYGGQIIAKRVPGSGKFYQFNNVEALQQQIRAELTDDYGDEARVAFQWAIKIMKALNGQM

E*

>GROS21-1_SAMEA9560794

MSLRELTKQAHTDAEKSEFVKILFSGKINPKLYANYLKNQHPMYEILEVCAMPHQLLHGLPDMRRAPAILADFQELWGDDPNEPTILPVVGDYVKYILSIKDDPKKLMAHLYVRHMGDLAGGQMIAKRVPGSGKYYKFEDPEALKVAIRERLNDDMADEAIVCFAYAKRFFDEMMTLVEYKDADQ*

>GROS21-1_SAMEA9560823

MSLKDLTHEAHKEAETKPFVKVLFSGKIDPKLYATYLKNQHPQYEILEVCAMIHPGLLGGTEDARRAPAINADFEELWNKETDGEPTILPVVKRYIDYIMSIKDDPKRLLAHIYVRHFGDMAGGQQIAKRVPGSGRMYQFKDAEALKANIRARLSDDMAEEAKVCFKFAGELFEEMMTLVPNE*

>crystal_bog_phage_scaffold_2_90

MATLKEVTRAVHDEAEHTEWGKLLVSGNITKEQYVHYLYNLLEIHQAIESRGVITMPEILRVKAIISDIEATDNTISPVTLDSTWKYIAHLKGLTDDLLWCHIYCHYLGYMYGGQMIKKAIPFSTTMLDFDNRAECIAYIRQHLENANHNEAIAAFKWATFMFNDLWNHYKDGK*

**Ferredoxin-dependent bilin reductases (FDBR)**

>MOSIG

MIWDRLIKWKDETVELLNKELVEYNEPGMERFNNEEFGWVNRTWKNKYIRRAHVDVVDVR

ETKGLWMAHVCLFPEVTNGGPIYGFDIIAGKKKVTGAFHDFSPLLQKEHPLTQWFLEETK

WFKPSKERELPDWAKAIFSGGMIAAGNVTDEDELNKICTMAVSNLANYIDKIRNHHDTDD

MENVINAQNYYCEHQQKNPHTPRVMQSLGLPEDDIKLFCQDNLFPKI*

>SAMEA2619974_635

MNDLWDNLISLQDEFIKKFSDVGVEIFEPGMDHFNQPGWMNKVWQTESVRRCHIDVVDVRDTKKLWMMHVCIFPNLDNTSPIFGFDVISGKNKMTGAFHDFSATTHKDHFMIKWFADSVSNFVPVKRRPLPEWALNIFSPSMVAASNVNREAESLDIIMLTLKNLNYYFDNVSESNGRGIVEEVSERQNYYCDNQKKNPHTPKVMKSLGLDEEDVNLFCSDILFPRINIGYDTSQ

>Ga0115011_10000306|738531

MAMERRSRIWEMLEQHTHSIIANFEREGEEIFEPTMKKFNRPEQGWVNRVWATPEARRCHLDVVDARDEKGLYMFHCCVFPKLTSTAPIYGLDVIAGAKKVTGFFHDFSPLAKKDHSMVDWFVKEASLYTPSKPRPLPDWAMKIFSPGMIAAGNINTEKELTQALSMAQSNLSVYFTLLRREKEQGDIQEIKDAQNRYAKHQRENPHTPRVMLSLGLPEDDVKEFCSDALFPYVE

>Ga0115000_10000447|754418

MVMERRSRIWEMLEQHTQSIIANFEREGEEIFEPAMKKFNRPEEGWVNRVWKTPEARRCHLDVVDARDEKGLFMFHCCVFPNLTSEAPIFGLDVIAGAKKVTGFFHDFSPLAKRDHSMVDWFVKEASNYTPSKARPLPDWAMKIFSPGMIAAGNINTEKELTQALSMAQANLQVYFTLLRRNNEVGDLQEIKDAQNRYAKHQRENPHTPRVMLSLGLPEDDVKEFCTDALFPYVE

>Ga0099957_1046306_490981_Split

MIWERLIKLEKEIIAILDRRCKEYNEEGMDRFNNDTWTNRTWSNMSVRRAHVDVVDARETKGLWMAHICLFPNLTNGGPIYGFDVIAGKKKITGVFHDFSPLLLKDHPLTKYFIEENKWFKPSKERELPDWAKAIFSPGMIAAGRVTEEKELNQICTLATSNLENYLDKIGHYNSDSKEEDVIRAQNFYCEHQQQNPHTPRVMKTLGLPEDDIKLFCTDNLFPKI

>JGI26273J51734_10000260|386802

MVMERRSRIWEMLEQHTHSIIANFEREGEEIFEPAMKKFNRPEEGWVNRVWKTPEARRCHLDVVDARDEKGLFMFHCCVFPNLTSEAPIFGLDVIAGAKKVTGFFHDFSPLAKRDHSMVDWFVKESKNYTPSKARPLPDWAMKIFSPGMIAAGNINTEKELTQALSMAQSNLQVYFTLLRRNKEVGDLQEIKDAQNRYAKHQRENPHTPRVMLSLGLPEDDVKEFCTDALFPYVE

>Ga0098058_1000146|507114

MNDLWDNLISLQDEFIKKFSDVGVEIFEPGMDHFNQPGWMNKVWQTESVRRCHIDVVDVRDTKKLWMMHVCIFPNLDNTSPIFGFDVISGKNKMTGAFHDFSATTHKDHFMIKWFADSVSNFVPVKRRPLPEWALNIFSPSMVAASNVNTEAESLDIIMLTLKNLNYYFDNVSESNGRGIVEEVSERQNYYCDNQKKNPHTPKVMKSLGLDEEDVNLFCSDILFPRINIGYDTSQ

>Ga0066849_10000198|410874

MAIYKRSRIWQMLEETTNYLTAVFDREGKEIFEPTMEKFNRPKDGWVNRVWETPEARRCHLDVVDARGTKGLYMFHCCVFPKLTHPGPIYGLDVIAGAKKVTGFFHDFSPLAKRDHSMVDWFVKEASNYKPSKVRELPDWAMKIFSPGMVAASNITQEKELNAALSLAQTNLGAYFTLLRREKGEGNIQEIKDAQNRYAKHQRENPHTPRVMKSLGLKDEDVEEFCTNALFPYVE

>Ga0115002_10003437_755546_Split

MIWERLIKLEKEIISILDRRCKEYNEEGMDRFNNDTWTNRTWSNMSVRRAHVDVVDARETKGLWMAHICLFPNLTNGGPIYGFDVIAGKKKITGVFHDFSPLLLKDHPLTKYFIEENKWFKPSKERELPDWAKAIFSPGMIAAGKVTEEKELNQICTLATSNLENYLDKIGHYNSDSKKEDVIRAQNFYCEHQQQNPHTPRVMKTLGLPEDDIRIFCTDNLFPKI

>Ga0114997_10001596|717177

LVMERRSRIWEMLEQHTQSIIANFEREGEEIFEPAMKKFNRPEEGWVNRVWKTPEARRCHLDVVDARDEKGLFMFHCCVFPNLTSEAPIFGLDVIAGAKKVTGFFHDFSPLAKRDHSMVDWFVKEASNYTPSKARPLPDWAMKIFSPGMIAAGNINTEKELTQALSMAQANLQVYFTLLRRNNEVGDLQEIKDAQNRYAKHQRENPHTPRVMLSLGLPEDDVKEFCTDALFPYVE

>Ga0114994_10001250|715058

LVMERRSRIWEMLEQHTQSIIANFEREGEEIFEPAMKKFNRPEEGWVNRVWKTPEARRCHLDVVDARDEKGLFMFHCCVFPNLTSEAPIFGLDVIAGAKKVTGFFHDFSPLAKRDHSMVDWFVKEASNYTPSKARPLPDWAMKIFSPGMIAAGNINTEKELTQALSMAQANLQVYFTLLRRNNEVGDLQEIKDAQNRYAKHQRENPHTPRVMLSLGLPEDDVKEFCTDALFPYVE

>Ga0115007_10001227_723900_Split

MIWERLIKLEKEIISILDRRCKEYNEEGMDRFNNDTWTNRTWSNMSVRRAHVDVVDARETKGLWMAHICLFPNLTNGGPIYGFDVIAGKKKITGVFHDFSPLLLKDHPLTKYFIEENKWFKPSKERELPDWAKAIFSPGMIAAGKVTEEKELNQICTLATSNLENYLDKIGHYNSDSKKEDVIRAQNFYCEHQQQNPHTPRVMKTLGLPEDDIRIFCTDNLFPKI

>BAR36617

RRAHVDVVDARDTKGLWMAHVCLFPELTNGGPIYGFDIIAGKKKVTGAFHDFSPLLKKEHPLTEWFKNETKWYKPSKERELPDWAKAIFSGGMIAAGNVQEEKELNQICTMAVSNLANYIDKIRNHHGEAKKEDVIKAQNYYCEHQQQNPH

>ECK26119

RRAHVDVVDARDTKGLWMAHVCLFPELTNGGPIYGFDIIAGKKKVTGAFHDFSPLLKKEHPLTEWFKNETKWYKPSKERELPDWAKAIFSGGMIAAGNVQEEKELNQICTMAVSNLANYIDKIRNHHGEAKKEDVIKAQNYYCEHQQQNPH

>EDC76490

RRAHVDVVDARETKGLWMAHVCLFPELTNGGPIYGFDIIAGKKKVTGAFHDFSPLLQKQHPLTEWFKEEVKWYKPSKERELPDWAKAIFSGGMIAAGNVQEEKELNQICTMAVSNLANYIDKIRSHHGESKKEDVIKAQNYYCEHQQQNPH

>EBQ35476

RRAHVDVVDVRDTKGLWMAHVCLFPELTNGGPIYGFDIIAGKNKVTGAFHDFSPLLQKHHPLTEWFLEETSWYKPSKERELPDWAKAIFSGGMVAAGNVTEEKELNQICTMATSNLANYIDKIRTHDGESNREDVIKAQNYYCEHQQMNPH

>BAR36228

RRAHLDVVDVRESKGLWMAHLCLFPMLTNGGPIYGFDVIAGEKKVTGAFHDFSPLLQKDHPLTKWFIEENKSFKPSKERELPDWAKAIFSGGMIAAGNIREEEELNKICTMAVSNLNNYIDKIRNHEGEADMKDVIKAQNYYSEHQQKNPH

>EBK42635

RRAHLDVVDVRESKGLWMAHLCLFPMLTNGGPIYGFDIIAGEKKVTGAFHDFSPLLQKDHPLTKWFIEENKWFKPSKERELPEWAKAIFSGGMIAAGNVREEDELNKICTMAVSNLNNYIDKIRNHEGEAEMADVIKAQNYYSEHQQKNPH

>ECE15585

RRAHLDVVDVRETKGLWMAHLCLMPMLTNGGPIYGFDIIAGKNKVTGAFHDFSPLLQKDHPLTEWFKEETKWYKPSKERELPDWAKAIFSGGMIAAGNVQEEKELNQICTMAVSNLENYIDKIRNHEGEAEMIDVIKAQNYYSEHQQKNPH

>EDE80231

RRAHLDVVDVRETKGLWMAHLCLMPMLTNGGPIYGFDIIAGKNKVTGAFHDFSPLLQKDHPLTEWFKEETKWYKPSKERELPDWAKAIFSGGMIAAGNVQEEKELNQICTMAVSNLENYIDKIRNHEGEAEMIDVIKAHNYYSEHQQKNPH

>ECK49855

RRAHLDVVDVRESKGLWMAHLCLMPMLTNGGPIYGFDIIAGKNKVTGAFHDFSPLLQKDHPLTEWFKEETKWYKPSKERELPDWAKAIFSGGMIAAGNVQEEKELNQICTMAVSNLENYIDKIRNHEGEAEMIDVIKAQNYYSEHQQKNPH

>EBN94631

RRAHLDVVDVRETKGLWMAHLCLMPMLTNGGPIYGFDIIAGKNKVTGAFHDFSPLLQKDHPLTEWFKEETKWFKPSKERELPDWAKAIFSGGMIAAGNVQEEKELNQICTMAVSNLENYIDKIRTHEGEAEMIDVIKAQNYYSEHQQKNPH

>ECZ53645

RRAHVDVVDARETKGLWMAHICLFPMKNNGGPIYGFDIIAGKNKITGAFHDFSPLLKKEHPLTRWFIEENKWFKPSKVRELPDWAKAIFSEGMIAAGNVREERELEQICTLALSNLNAYIDKIGHYNSDSNEEDVVRAQNFYCENQQKNPH

>EBI88122

RRAHVDVVDARETKGLWMAHICLFPMKKNGGPIYGFDIIAGKSKITGAFHDFSPLLKKEHPLTRWFIEENKWFKPSKVRELPDWAKAIFSEGMIAAGNVREEKELEQICTLALSNLNAYIDKIGHYNSDSNEEDVIRAQNFYCENQQKNPH

>EBI74694

RRAHVDVVDARKTKGLWMAHICLFPMTKNGGPIYGFDIIAGKNKITGAFHDFSPLLKKEHPLTRWFIEENKWFKPSKVRELPDWAKAIFSEGMIAAGNVREERELEQICTLAVSNLNAYIDKIGHFNSDSNEEDVIRAQNFYCENQQKNPH

>New_JCVI_SCAF_1096626956256

RRAHVDVVDARETKGLWMAHICLFPMKKNGGPIYGFDIIAGKKKVTGAFHDFSPLLKKEHPLTRWFIEENKWYKPSKVRELPDWAKAIFSEGMIAAGNVQEERELNQICTMAVSNLNAYIDKIGHFNSDSNEEDVIRAQNFYCENQQKNPH

>actinobacterium_SCGC_AAA041_L1

RRAHIDVVDARHSKGLWMMHCCIFPHTHNPAPIFGFDVIAGKSKITGCFYDYSPAGDVEHPMLDWFSSEAAKLQWNKTRKLPEWAERIFSSSMIAASNVSKPEEVEQILSIAKKGIDQYLVAVGETNKTAISTAHEQNFYCENQKLNPH

>CB0101

RKLHLETARLGAGLQILHCVYFPDPRYDLPVFGADIVAGRGVVSAAIVDLSPVSGQLPDAVAQGLAALPARSFSQERELPEWGSIFSPYVRFVRPADAAEEQQFIGVVSDFLQVLSAACQAAEPQPIDHPDTVKRHDGQLSYCRQQKRNDK

>CB0205

RKLHLETARLGAGLQILHCVFFPDPRYDLPVFGADIVAGRGVVSAAIVDLSPVDGALPQAVLQRLEALPKRAFSQERELPEWGTIFSPYVRFVRPADAAEEQSFIELVSDFLRVLGEASAEAVPQPIDHPDTVRRHSGQLSYCQQQKRNDK

>WH5701

RKLHLETARLGAGLQILHCVFFPDPRYDLPIFGADIVAGPAGVSAAIADLSPTGGELAAGIAADLAALPRRPYSQPRELPAWGTIFSPFVRFVRPMSEQEDGWFLEEVAALLAVMGKAVASTPEQASDDPATVSRYHGQLSYCQQQKRNDK

>EDA50093

RKIHLELARLGRGLQILHCVWFPDPRFDLPIFGADIVAGPAGVSAAIVDLSPVSGTLPSGIETALADTPSPAFRQVRDLPGWGTIFSPHVCFIRPDGAEEEVLFRSRVEEVLTILRTAVLQTACEPATAASTIRRYEGQLSYCLQQKRNDK

>EBT51052

RKIHLELARLGRGLQILHCVWFPDPRFDLPIFGADIVAGPAGVSAAIVDLSPVSGTLPSGIETALAGTPGPAFRQVRDLPGWGTIFSPHVCFIRPDGAEEEVLFRSRVEEVLTILRTAVLQTACEPATAASTIRRYEGQLSYCLQQKRNDK

>ECY52537

RKIHLELARLGRGLQILHCVWFPDPCFDLPIFGADIVAGPAGVSAAIVDLSPVSGTLPSGIKTALEGTPSPAFRQVRELPGWGTIFSPHVCFIRPDGVEEEALFRSRVEEVLAILRTAVLQTACEPATAASTIRRYEGQLSYCLQQKRNDK

>EDA45310

RKLHLEIARLGVGLQILHCVWFPDPRFDLPIFGADVVAGPAGISAAIVDLSPVGEALPDMLASSLSGLNRPRFRQERALPGWGTIFSPHVCFIRPDGADEERFFQCLVMDYLDVLSSAVVQAEGEQANSLETLKRYQGQLNYCIQQKRNDK

>MIT9303

RKLHLEIARLGGGLQVLHCVFFPDPRFDLPIFGADIVASPAGISAAIVDLSPVGLTMPVALLHGLESLPIPAFQQVRELPAWGSIFSPFVQFIRPASSEEESWFVDLADGYLKALISSVIDATPDASDAASTIQRHKSQLSYCIQQKRNDK

>MIT9313

RKLHLEIARLGGGLQVLHCVFFPDPRFDLPIFGADIVAGPAGISAAIVDLSPVGLTMPEALLHGLESLPIPAFQQVRELPEWGSIFSPFVQFIRPASSQEESWFVDLADGYLKALISSVIDATPDASDAASTIQRHKSQLSYCIQQKRNDK

>ECW36805

RKLHLETARLGLGLQILHCVFFPDPRFDLPVFGADLVASPAGISAAIVDLSPVTDRLPDAVRTPLEDLVLPPFQQVRALPPWGTIFSPFVRFIRPVDREEEGWFVDLVGSYLAILAAAIDQTEPDPPQAAPTIARYQGQLSYCLQQKRNDK

>RS9917

RKLHLETARLGLGLQILHCVFFPDPRFDLPVFGADLVASPAGISAAIVDLSPVTDRLPDAVRSPLEDLVLPPFQQVRELPPWGTIFSPYVRFIRPVDREEEGWFVDLVGSYLAILAAAIDQAEPDPPQAVPTIARYQGQLSYCLQQKRNDK

>RS9916_PcyA

RKLHLETARLGLGLQILHCVFFPDPRFDLPVFGADIVASPAGISAAIVDLSPVGSDLPASVRQPLEAITLPPFEQVRELPAWGTIFSPFVRFIRPVNSTEEGWFVDLVGQYLDVLRQAVETTEPDSSDNPSTINRHQGQLSYCLQQKRNDK

>ECX14994

RKLHLEIARLGLGLQILHCVFFPDPRFDLPVFGADIVASPAGISAAIIDLSPVGDELPERIERGLEAAVIPAFEQVRELPTWATIFSPFVRFIRPVNQQEEDWFVELVDDYLQVLGDAVQAAEPDDPSAPSTLARYHGQVSYCRQQKRNDK

>CCMP1375

RKLHIETAVFEPSLEILHVVFFPDPAFDLPIFGVDLIAVPQGISAAIVDLSPVRDKLPRTIENQLAQIEIPSFEKVRKLPDWGDIFSSHVQFITPIGAEENGFFLDLVDKFLTILIDYSESIEPDLDDSPFTIERIEGQMYYCLQQKQNDK

>MIT9211

RKIHLEIAMLGSSLEILHCVFFPDPTFDLPIFGVDIVAGMEGVTAAIVDLSPVGKALPTAIDLKLRELRIPSFTNPRKLPEWGNIFSSYVQFIKPNARSEDSLFLDLVDGFLNVLITCSISSTPDSLDSPLTIERYERQQFYCLQQKRNDK

>Chamaesiphon_minutus

RKLHLELAQVGKNLDILHCVMFPHPEYALPMFGTDIVAGRGQISAAIADLSPLNSQRILSDNYRLPLLLLPQHNFSQPRELPDWADIFSEFCFFVRPANPEEEQWFLDRVIAMLTIHCQQAAMATPVSNTERAEIVTAQNYYCTKQQQNDK

>PCC7120_PcyA

RKMHLELAKVGNMLDILHCVMFPRPEYDLPMFGCDLVGGRGQISAAIADLSPVHLDRTLPESYNSALTSLNTLNFSQPRELPEWGNIFSDFCIFVRPSSPEEEAMFLGRVREFLQVHCQGAIAASPVSAEQKQQILAGQHNYCSKQQQNDK

>Crocosphaera_watsonii

RKMHLELAKVGRGVDILHCVMFPNPDYALPMFGCDIVAGKKGVSAAIADLSPTNPELTLSNEYNQALSQLETPNFSDQRELPEWGDIFSDYCLFIRPHTQEEEKLFLQRVGNFLRVHCQQANQSQTVSNKERELNIQGQQYYCEKQQQNDK

>PCC6803

RKMHLELAKVGKGLDILHCVMFPEPLYGLPLFGCDIVAGPGGVSAAIADLSPTQSDRQLPAAYQKSLAELGQPEFEQQRELPPWGEIFSEYCLFIRPSNVTEEERFVQRVVDFLQIHCHQSIVAEPLSEAQTLEHRQGQIHYCQQQQKNDK

>PCC7502

RKLHIELAQVGSNLDILHCVMYPQFNYPLPIFGADLIGGKAGISAAIVDISPTVTIPDTISEPIRAWQASQPQFSQPRELPAWGKNIFSEFCIFVRPQGQLEENLFLQTLEKYCTQVCTVASELESVSEEIKTIIQDRHRHYCQQQQQNDK

>MIT9515

RKLHIEVAEFSRRLKILHCVFFPDPHYDIPIFGMDLVKVNEVVSAAIVDLSPSSKNQNLKYDNLLSTIDKSVFESEREIPGWGDIFSQNVFFASLKNESEKSAFCKIVDHYLLVLIKLSKSSILDHDQEIIQERIDFQKNYCRQQMKNEK

>ECX32477

RKLHIERANTDKIEIIHCVLYPDPEYRLPIFGCDIIQTTSTITAAIVDVSPVHGVDLNPQLSVVSEKYKFKDKRPLPIWAEDIFSNHCKFARLKDQKAKDDFYAVVQKYLLIYCNAVRHAKRDPHFPFIPIMKRLDDQCWYCVSQRKNQK

>EDF99713

RRIHLETAETDKIQIVHCVFWPDPAYYLPIFGADIIQTHAGVTAAIVDISFVDGVDWSDKLSPISKQIQFKDNRQLPEWGEIFSPYCKFARLKTEEEQNKFYQVVLEYLRIYCTEVQTAQWSDDWVGIMKRLDDQCWYTTSQRKNKK

>EBC53025

RKLHIERAITSKGIEILHCVLFPDPEYPIPIFGCDIVEAGGKVTAAIVDVSPVHKVDYSLGNITYDFKKNRHLPEWGEIFSPWCKFVRLEESEYDNFLLLCSEYLEVFCHIVRTAERETDWRNTMRRYDDQLWYCTSQMKNKK

>PRSM4_PcyA

RKLHIERAITNKGIEILHCVLFPDPEFPIPIFGCDIVEAGGKVTAAIVDVSPVHKVDYSLGNITYNFKKNRHLPEWGEIFSPWCKFVRLEKDEYDDFLLLCSEYLEVFCHIVRTAERETDWRNTMRRYDDQLWYCTSQMKNKK

>PRSM6_1

RKIHLETATAGKLNIVHTVFWPDPNYNIPIFGCDIVSVGNIITAAIVDISPVRGCEEIYDKISHISNSFQFSERRHLPLWAEDVFSPHCKFVRISKPAEKIEFVRIAKEYLDIVCDQVRKAEYDTKWVRTMLRYDDQIWYAKQQRKNKK

>EDE92611

RKIHLETATAGKLNIVHTVFWPDPNYNIPIFGCDIVSVGNIITAAIVDISPVRGCEEIYDKISHISNSFQFSERRHLPLWAEDVFSPHCKFVRISKPAEKIEFVRIAKEYLDIVCDQVRKAEYDTKWVRTMLRYDDQIWYAKQQRKNKK

>PRSM1

RKIHLERAITPKVEILHSVFWPDPEYNLPIFGMDIVVAGNKVTAAIVDISPVHGTEDIKRYREVSEISSRYNFSGIRHLPEWGDVFSPFCKFQRLETSDDIYKFYSCCQEYIKVYTQMVRESEHDFEWIDIMKRYDDQLHYVNQQRKNTK

>PSSM7_PcyA

RKMHLETGYTENISVMHCVLYPIPDYPIPIFGADIVETPHAVTAAIVDISPVFGTQKYVDVYRDISYKYKFKENRVLPLWTDEVFSQGCKFMRIRTDEEREMYMDLIKESIQLYKGIVENSEFDMEWINTMKRIDDQCYYCKQQRKNKK

>EBL72495

RKVHLETGYTDNIEVMHCVLYPDPEYPIPIFGADIVATPTVVTAAIADISPVYKTDKIYKKLGKLANKYEFKEKRPLPEWADIFSPYCQFMRLRDEEEKSSYGCMIEEFIDFYVDIVKKAKKDNDWVNTMLRFDDQIYYCKQQRKNKK

>PSSM4_PcyA

RKIHLETCKTKYLDVLHCVLFPEPRYKLPIFGCDIIANNRIVTAAIVDISPVKGVRGEFYKDIKPISERYMDFDFRKLPEWADIFSPHCKFMRLHKQTEQIMYVQLLEEYLQVYVNAVSKAEKCMDIDATYDRYQDQVYYCQQQKQNKK

>PSSM3

RKIHLETCKTKYLDVLHCVLFPEPRYKLPIFGCDIIANNRIVTAAIVDISPVKGVRGEFYKDIKPISERYMDFDFRKLPEWADIFSPHCKFMRLHKQTEQIMYVQLLEEYLQVYVNAVSKAEKCMDIDATYDRYQDQVYYCTQQKQNKK

>EDF08813

RKIHLETCKTKYLDVLHCVLFPEPRYKLPIFGCDIIANNRIVTAAIVDISPVKGVRGEFYKDIKPISERYMDFDFRKLPEWADIFSPHCKFMRLHKQTEQIMYVQLLEEYLQVYVNAVSKAEKCMDIDATYDRYQDQVYYCQQQKRNKK

>EBG86788

RKVHLEYGETGSLEVMHCVFFPDPLYNLPIFGCDIVANQHRVTAAIVDISPVHGVKDIYKDIKPICEEFHDFDYRKLPAWADIFSPYCKFMRLNEEWEKVAYWQIVDKYLKVFCKEVNNAKKGSIEDAYKRYQDQLYYCKKPKMNRK

>BAR31269

RKVHMEIASLGPLDILHCIWYPDPEFDLPIFGADIIANKNIVTAAITDISPVDDLCHPIYEDIADISRYYCFKHNREIPAWGTIFSPYCKFARLDDDQEIDTFCHVVDEYLDAFVGAVWKSTMDSCGAEQRWVAQSNYCSNQKKNDK

>WH8020_PebA

RRWRVTRLDAGDSLQVFNSVAYPDYNYDHPLMGVDLLWFGARQKLVAVLDFQPLVQDKDYLDRYFSGLKELNQRFPDLNGEETMRSFDPNQYFSSWLLFCRGGAEQADLSLPKAFSAFLKAYWDLHDNAKSIPSTIPPEEVKNLQDKYDIYSAERDP

>CC9311

RRWRVTRLDAGDSLQVFNSVAYPDYNYDHPLMGVDLLWFGARQKLVAVLDFQPLIQDKDYLDRYFYGLKDLNQRFPDLNSEETMRSFDPNQYFSSWLLFCRGGAEQADVSLPNAFSAFLKAYWELHDNAKSIASTIPPEEVKNLQEKYDIYSAERDP

>RS9916

RRWRVTRLDAGESLQVLNSVAYPDHDFDHPLMGVDLLWFGARQKLVAVLDFQPLVQDADYLDRYFTGLKNLNAQFPDLNGEETMRSFDPNQYFSSWLLFCRGGADQADSSLPPAFSSFLKAYWKLHDDACNGTASIPPDEVKRLQENYDIYSAERDP

>EDA84932

RRWRVTRLDAGDSLQVLNSVAYPDFDLDHPLMGVDLLWFGARQKLVAVLDFQPLVQDKDYLDRHFDGLKDLNARFPDLNGEETMRSFDPNQYFSSWLLFCRGGSEEADRSLPKAFSAFLKAYWGLHDEASKEPSSISPGDVERLQNAYDVYSAERDP

>EBN58148

RRWRVTRLDAGESLQVLNSVAYPDYSFDHPLMGVDLLWFGARQKLVAVLDFQPLVQDPDYLNRHFDGLKDLNARFPDLSGEETMRSFDPNQYFSSWLLFCRGGSEQAETSLPPAFSSFLKAYWSLHDEASTSSSSLSPEDVQSLQENYDIYSAERDP

>ECV82867

RRWRVTRLDAGDSLQVLNSVAYPEHGLDHPLMGVDLLWFGARQKLVAVLDFQPLIQDKEYLERHFTGLRALHEQYPELNGEETMRSFDPNQYFSPWLLFCRGGADEAEGSLPQAFDAFLSAYWSMHDQAKGQSALAASEVERLQNAYDVYSAERDP

>EBE82302

RRWRVTRLDAGDSLQVLNSVAYPEHGLDHPLMGVDLLWFGARQKLVAVLDFQPLIQDKEYLERHFTGLRALHEQYPELNGEETMRSFDPNQYFSPWLLFCRGGADEAEGSLPQAFDAFLSAYWSMHDQAKGQSALATSEVERLQNAYDVYSAERDP

>EBE98358

RRWRVTRLDAGDSLQVLNSVAYPEHGLDHPLMGVDLLWFGARQKLVAVLDFQPLIQDKEYLERHFTGLRALHEQYPELNGEETMRSFDPNQYFSPWLLFCRGGADEAEGSLPQAFDAFLSAYWSMHDQAKGQSGLTASEVERLQNAYDVYSAERDP

>EDJ00712

RRWRVTRLDAGENLQVLNSVAYPDHSYDHPLMGVDLLWFGKRQKLVAVLDFQPLVQDQDYLDRHFDGLKALNERFPDLNGEETMRSFDPNQYFSPWLLFCRGGAEQAEDSLPQAFDAFLSAYWAMHESADGSSKLSSQDVERLQNAYDVYSAERDP

>MIT9303_PebA

RRWRVSRLDAGESLQVLNSVAYPNYNIDQPLMGLDLLWFGKRQKLVAILDFQPLIQDHSYLERHFQGLKALQNRFPELSGEETMRLFDPNQYFSPWLLFCRGGAEKATNSLPEAFNAFLHCYWELHQQNSKKASLIPAAEVKQLQIAYDIYSAERDP

>EDA84770

RRWRVTRMDAGDSLQVLNSVAYPDLDNDQPLMGVDLLWFGARKKLVAVLDFQPLVQKQDYLDRHLVELKKLHDKFPDLSGEETMRSFDPNQYFSPYLLFCRGGAEQAEESLPEAFSSFLDSYWKLTARSQSEPSMIPPAEVQELQVAYNQYSAERDP

>NATL1a_PebA

RRWRVTRLDAGDRLQVLNSVAYPNEQNDMPIMGIDLLWFEKKQKLVAILDFQPLVQDKEYLDRYFDGLKSLKKSFNEFNSDMKSNIYDPTKYFSPWALFCKGGNFEAENILPKIFSSFLKCYWKNLDLSKANENHIKSQEVSILHIDYDKYSAEKDP

>ECX71163

RKWRITRLDGGKKLQVFNTVAYPNFDSEIPILGADILWFGTSQKLLAILDYQPLIQEDKYLEKYCSSLSSIKKNYSEFDNNKMKNIYDSKKYFSPWVIICRGSKLNLDRDLNNIFHSFVNNYLNIYKSNPANQFLNAEEIKINQIKYDKYSFEKDP

>EBW11418

IKAREVEIWNDKSCIYNNIIYPKTGSNLPCFGMDLMGFFDKKVIIVFDFQHPIENYLFSVDGLPKGKGDYRFFEPGNHFSENIYIQYCTMSEVDDYLGMFIEYLLKYKEMVDEAKPTGMDTSVYKDFDTYMTKLDP

>EBC59783

IKAREVEIWNEKSCIYNNIIYPKTGSNLPCFGMDLMGFFDKKVIIVFDFQHPTENYLFSVEGLPKGKGDYRFFEPGNHFSENIYIQYCTMNEVDDYLDMFKKYLLKYKEMVDEAKPTGMDTSVYKDFDTYMTKLDP

>EBL18613

IKSREVEIWDKKSCIYNNIIYPKTGANLPCFGMDLMGFFEKKVIIVFDFQHPTENYLFSVDGLPKGKGDYRFFEPGNHFSENIYIQYCTMNEVDDYLGMFTEYLLKYKEMVDEAKPTGMDTSVYKDFDTYMTKLDP

>EBM71994

IKAREVDIWSDKSSIYNNIIYPKTGSNLPCFGMDLMGFFEKKVIIVFDFQHPKEKYPFSVDGLPKHEGDYRFFEPGNHFSENIYIAKCTASEVDDHLEMFTTYLTKYRDMIELEKPTGNDTSEYKDFDSYMTKLDP

>EBY14952

IKAREVDILSDKSSIYNNIIYPKTGSNLPCFGMDLMGFFEKKIIIVFDFQHPKEKYPFSVKGLPKSEGDYRFFEPGNHFSDNIYIAKCTANEVDEHLEMFTTYLTKYKEMVELEKPTGMNTSEYKDFDAYMTKLDP

>EBI82107

IKAREVDIWSDKSCIYNNIIYPKTGADLPCFGMDLMGFNENRVIIVFDFQHPREKYSFSVDGLPKHEGDYRFFEPGNHFSENIYIAKCKSNEVDDHLEMFTTYLTKYRDMIELKKPIGIDTNQYKDFDSYMTKLDP

>ECE99069

IKSREVDIWSDKSSIYNTIIYPKTGSNLPCFGMDLMGFTEKRVIIVFDFQHPVEKYLFSVEGLPKAEKEYRFFEMGNHFSENIFVRYTTFDKVDEHLDMFKQYLTKYKEMVELERPSGTDTSTYKDFDAYMTKLDP

>ECV51398

IKSREVDIWSDKSCIYNTIIYPKTGSNLPCFGMDLMGFTEKRVIIVFDFQHPVEKYLFSVEGLPKAEKDYRFFEMGNHFSENIFVRYTTFDKVDEHLDMFGQYLTKYKDMIELEKPTGTDTSTYTDFDAYMTKLDP

>SSSM7

NKSREVEIWNEKSCIYNTIIYPRTGENLPCFGMDLMGFFEKKVIIVFDFQHPIENCPFSVQGLPKAEQDYRFFEMGNHFSDNIYVRYCTFAEVDEHLDMFKKYLTVYRDMLESKKPSQNLMHKTYHDFDKYMRKLDP

>PSSM5

LKAREAHITDPNSDIYNTILYPKTGADLPCFGMDLMKFNDKKVIIVFDFQHPREKYLFSVDGLPEDDGKYRFFEMGNHFSKNIFVRYCKPDEVDQYLDTFKLYLTKYKEMIDNNKPVDEDTTVYSDFDTYMTELDP

>PSSM2_PebS

LKAREAHIVDPNSDIYNTILYPKTGADLPCFGMDLMKFSDKKVIIVFDFQHPREKYLFSVDGLPEDDGKYRFFEMGNHFSKNIFVRYCKPDEVDQYLDTFKLYLTKYKEMIDNNKPVGEDTTVYSDFDTYMTELDP

>EDG78227

IKAREAHITDPRSDIYNTILYPKTGADLPCFGMDLMKFSEKKVILVFDFQHPREKYLFSVDGLPKDDGKYRFFEMGNHFSENIFVRYCKPDQVDEHLPMFKQYLTEYKKMVELNDPQGEDTTVYADFDKYMTELDP

>Phage_MED4_213

LKAREVEIYNEKSSIYNNILYPKTGSNLPCFGMDLMGFAEYKVIIVFDFQHPTENHMFSHPDLPVATEDYRFFEKGNHFSENIFVRKCKMDEVDQYVGEFAQYLDAYRRMVKAVEPDGEDTTIYSDFDTYMTRLDP

>PHM1

LKAREVEIYNEKSSIYNNILYPKTGSNLPCFGMDLMGFAEYKVIIVFDFQHPTENYMFSHPDLPVATEDYRFFEKGNHFSENIFVRKCKMDEVDQYVGEFAQYLDAYRRMVEAVQPDGEDTTIYSDFDTYMTRLDP

>PHM2

LKAREVEIYNEKSSIYNNILYPKTGSNLPCFGMDLMGFAEYKVIIVFDFQHPTENYMFSHPDLPVATEDYRFFEKGNHFSENIFVRKCKMDEVDQYVGEFAQYLDAYRRMVEAVEPDGEDTSVYADFDTYMTRLDP

>PRSM6_2

LKSRHVDIWDGKHLSIHNNIIYPKTPQVGEEIIPCFGMDLMGFSEKKVIIVFDFQHPTEKYLYEVESLPYAEKDYRFFEKGNHFSKNIYVRYCKRDEVDDYLPMFKTYLLWYKHLIDEGKPTGEDTTHYHDFDKYMIKLDP

>ECV37703

RQVRAACVSAGSSASVLNFVINPLARYDLPFFGGDLVTLPSGHLLALDLQPADKQDSEHTQGVWDRLMPIFDRWRSQLPDGGPIPDEAQPFFSPGFLWTRLPLGEEGDQLIKSVVRPAFNDYLNLYLELAAAAVPVSDQRMEHLLAGQRRYTDYRAEKDP

>EBE83203

RQVRAACVSAGSSASVLNFVINPLPRYDLPFFGGDLVTLPSGHLLALDLQPADKQDSEHTQGVWDRLLPIFDRWRSQLPGGGPIPEEAQPFFSPGFLWTRLPLGEEGDQLIESVVRPAFNDYLNLYLELAAAAVPVSDQRMEHLLAGQRRYTDYRAEKDP

>CC9902

RQVRAACVYGGTAASVLNFVINPSARFDLPFFGGDLVTLPSGHLLALDLQPADKSDEAHTQQVWEKLIPIFERWRSKLPDGGPIPEEAQPFFSPGFLWTRLPLGDEGDQLINSVVRPAFNDYLSLYLELAEAAKPVGDDRRDHLLKGQRRYTDYRAEKDP

>BL107

RQVRAACVFGGAAASVLNFVINPSARFDLPFFGGDLVTLPSGHLLALDLQPADKSDAAHTQPVWDKLIPIFERWRSKLPDGGPIPEEAQPFFSPGFLWTRLPLGDEGDQLIESVVRPAFNDYLRLYLELAEAAKPVTDDRRDHLLAGQRRYTDYRAEKDP

>xWH8020_PebB

RQVRAACVSAGSAASVLNFVINPKSTYDLPFFGGDLVTLPAGHLLALDLQPAIKTDEVHTTHVWDRLIPIFERWRDQLPYGGPIPEEAQPFFSPGFLWTRLPLGEEGDELIQSIVRPAFNDYLDLYLELAASAERVTDERSEVLLQGQRKYTDYRAEKDP

>EDA84772

RQVRAACVEAGSAASVLNFVINPLTTYDLPFFGADLVTLPAGHLLALDLQPALKSDEDHTEKVWSQLMPIFERWQAHLPSGGPIPEEAQPYFSPGFLWTRIPLGEEGDALIDDVVMPAFKDYLSLYLGLVDSAEVVAPDRSEALLTGQRRYTAYRAEKDP

>EDA84931

RQVRCACVEAGAAASVLNFVINPSCRYDLPFFGADLVTLPNGHLLALDLQPVDKADSEHTDPVWERLLPLFERWRAELPDGGPIPEEAQPYFSPAFLWTRIPLGADGDALIERVIRPAFSDYLQLYLDLVAKAQPVDDERAAHLLSGQKRYTAYRAEKDP

>CCMP1375_PebB

KQVRLACIKGGESLSVFNLLIHPLNDYDLPFFGADFVTLPNGHLLALDLQPALKLDNIHTENVWPRLIPLHDHWQSLLPSGGEIPKEAEPYFSPGFLWSRLPLSKESDNIISEILRPAFGEYLSLYIELLHIAKPLKKERALKILEGQKAYINYRSTKDP

>MIT9211_PebB

RQARAACIEAGPAASVLNLVINPFHNFELPFFGADFVTLPSGHLLALDLQPVLKKDEIHNQKVWSKLIPIHDHWQSLLPSGGPIPQEAETFFSPGFLWTRLPLDDQGSKLISKVIRPAFQEYLTLYIDLISDAQEVSKERSLEILSGQKAYINYRAEKDP

>3300009785_Ga0115001_100000

MKRGLVMERRSRIWEMLEQHTQSIIANFEREGEEIFEPAMKKFNRPEEGWVNRVWKTPEARRCHLDVVDARDEKGLFMFHCCVFPNLTSEAPIFGLDVIAGAKKVTGFFHDFSPLAKRDHSMVDWFVKEASNYTPSKARPLPDWAMKIFSPGMIAAGNINTEKELTQALSMAQANLQVYFTLLRRNNEVGDLQEIKDAQNRYAKHQRENPHTPRVMLSLGLPEDDVKEFCTDALFPYVE

>Bradyrhizobium_ORS278

MSDGDDGDDLICDLQHAAEDFAADLRAVPALERVRVPDFHAAAIAEGTLQKEITWRNDVFVGGRFRHAHVESFSIGEQIGVVHVCIFPHFDRAAPIFGFDIIAGRKKATGAFLDLSPTTMAANAIIDGWSEASAAQRANFRETRILPAWAASIFSRSALAIRPASRHEVASVVALGRSALAYYLDAHLATAAEAEMQVAQRKYIEAQRSNEHTFRMLAGCVGVDLARDFIDGWLFPAPPSPGESRSDAAARGALAHVD

>Rhodospirillales bacterium 20647

MGVLHVCVFPHWDDLAPIFGFDLVAGPARVTGIFLDLSPVLPSRPQLTLRDAVGSAALQAFATRRALPEWADIFSEDMVAIRPVSGEEIDRALALAEQALDVLLATVRVTTGQVVDAIAAGQARYCAGQRQNEHTVRMLTNFI

>Cyanothece_UBA12306

MLDTSPVSIRPQLNPLISQLADVILSHWEQYLSLSPYELPDGLGYVEGKLEGEKLVIENRCYQTPQFRKMHLELAKVGRGLDILHCVMFPNPTYSLPMFGCDIVAGPRGVSAAIVDLSPANLQLTLSSTYLNALSQLPLCEFSDTRDLPEWGDIFSEYCLFIRPSNSAEETQFLSRVADFLKIHCQLALQSQPVTLEQKTLNLAGQKYYCNKQLENDKTRRVLEKAFGQEWANKYMTRVLFDLPA

>SAMEASAMEA2620861_60095

MITKRGLVMARRSRIWEMLEGQVSNIIATFDREGTEIFEPTMKKFNRPEEGWINRVWETPEARRCHIDVVDARDTKGLYMFHCCIFPHLNSPAPIYGLDVIAGANKVTGFFHDWSPLAKRDHTMVDWFVKEASLYEPSKPRPLPDWAMKIFSPGMIAAGNIKEEKELTHALSMAQSNMGVYFTLLRREKNNETDISEQEIKDAQNRYAKHQRENPHTPRVMLSLGLPEDDVKEFCTDALFPYVE.

>SAMEA2622736_820952

MAIYKRSRIWQMLEETTTFIIEVFKREGKEVFEPTMEKFNRPKDGWVNRVWETPEARRCHLDVVDARETKGLYMFHCCVFPNLTHPGPIYGLDVIAGAKKVTGFFHDFSPLAKKDHSMVDWFVNEAKNYEPSKVRELPDWAMKIFSPGMVAASNITTEKELNAALSLAQCNLGAYFTLLRREKTKGDIEEIKDAQNRYAKHQRENPHTPRVMKSLGLPEADVKEFCTNALFPYIE.

>SAMEA2619791_29744

MKRGLVMAQRSRIWEMLEGQVRNIISTFEREGKEIFEPTMKKFNRPEDGWVNRVWETPEARRCHIDVVDVRDKKGLYMFHCCVFPHLNSPAPIYGLDVIAGANKVTGFFHDFSPLAKRDHTMVDWFVKEAKNYEPSKVRELPEWALKIFSPGMIAASNIKEEKELNAALSLAQSNMGAYFTLLRRERNNPTKVTEQEIKDAQNRYAKHQRENPHTPRVMLSLGIPEDDVKEFCTDALFPYVE.

>SAMEA2620861_551347

MKQDNVLDLQQIYLKIFKNLLNKTRRGLVMAQYKRSRIWNMLEETTTLIMAVFEREGKEIFEPTMEKFNRPKDGWVNRVWETPEARRCHLDVVDARATKGLYMFHCCVFPRLNSPAPIYGLDVIAGAKKVTGFFHDFSPLAKRDHSMVDWFVKESSNYKPSKVRELPEWALKIFSPGMVAASNITTEKELNHALSLSQCNLGAYFTLLRREKNNKTDITEQEIKDAQNRYAKHQRENPHTPRVMKSLGLPEDDVKEFCTNALFPYVE.

>SAMEA2622841_6527

MKQDNVLDLQQIYLKIFTNLLNKTRRGLVMAQYKRSRIWNMLEETTTLIMAVFEREGKEIFEPTMEKFNRPKDGWINRVWETPEARRCHLDVVDARATKGLYMFHCCVFPRLNSPAPIYGLDVIAGAKKVTGFFHDFSPLAKRDHSMVDWFVKESSNYKPSKVRELPEWALKIFSPGMVAASNITTEKELNHALSLSQCNLGAYFTLLRREKNNKTDITEQEIKDAQNRYAKHQRENPHTPRVMKSLGLPEDDVKEFCTNALFPYVE.

>SAMEA3300000199_SI39nov09_10m

MKRGLVMERRSRIWEMLEQHTHSIIANFEREGEEIFEPAMKKFNRPEEGWVNRVWKTPEARRCHLDVVDARDEKGLFMFHCCVFPNLTSEAPIFGLDVIAGAKKVTGFFHDFSPLAKRDHSMVDWFVKESKNYTPSKARPLPDWAMKIFSPGMIAAGNINTEKELTQALSMAQSNLQVYFTLLRRNKEVGDLQEIKDAQNRYAKHQRENPHTPRVMLSLGLPEDDVKEFCTDALFPYVE.

>SAMEA2620861_1137947

MLSMKQDNVLVLQLTYLKKCCNLLRITKRGLVMAQRSRIWEMLEDATKNLMSKFDDEGKEIVEESMAKFNRPEDGWINRVWETPEARRCHIDVVDARESKKLYMFHCVVIPHFHTPAPIWGLDVIAGPNKVTGFFHDWSPLSGKREMDHPMVEWFCEESKTYEPSKVRELPDWALQIFSPGMIAAGNINTEKELTNALSLACTDLGPYFTLLRRYKQDFNLNSNIKSEKEVKEAQNRYAKFQRENPHTPRTMKALGLPEKDIEEFCTDALFPYAE.

>SAMEA2620413_422904

MLSMKQDNVLVLQLTYLKKCCNLLRITKRGLVMAQRSRIWEMLEDATKNLMSKFDDEGKEIVEESMAKFNRPEDGWINRVWETPEARRCHIDVVDARESKKLYMFHCVVIPHFHTPAPIWGLDVIAGPNKVTGFFHDWSPLSGKREMDHPMVEWFCEESKTYEPSKVRELPDWALQIFSPGMIAAGNINTEKELTNALSLACTDLGPYFTLLRRYKQDFNLNSNIKSEKEVKEAQNRYAKFQRENPHTPRTMKALGLPEKDIEEFCTDALFPYAE.

>SAMEA2620836_7330

MSIVLASLRCKPMLVVGSRQANSRLVMAQRSRIWEMLEDATKNLMSKFDDEGKEIVEESMAKFNRPEDGWINRVWETPEARRCHIDVVDARESKKLYMFHCVVIPHFHTPAPIWGLDVIAGPNKVTGFFHDWSPLSGKREMDHPMVEWFCEESKTYEPSKVRELPDWALQIFSPGMIAAGNINTEKELTNALSLACTDLGPYFTLLRRYKQDFNLNSNIKSEKEVKEAQNRYAKFQRENPHTPRTMKALGLPEKDIEEFCTDALFPYAE.

>3300009593_Ga0115011_10000306

MLEQHTHSIIANFEREGEEIFEPTMKKFNRPEQGWVNRVWATPEARRCHLDVVDARDEKGLYMFHCCVFPKLTSTAPIYGLDVIAGAKKVTGFFHDFSPLAKKDHSMVDWFVKEASLYTPSKPRPLPDWAMKIFSPGMIAAGNINTEKELTQALSMAQSNLSVYFTLLRREKEQGDIQEIKDAQNRYAKHQRENPHTPRVMLSLGLPEDDVKEFCSDALFPYVE.

>3300009790_Ga0115012_10000076

MLEQHTHSIIANFEREGEEIFEPTMKKFNRPEQGWVNRVWATPEARRCHLDVVDARDEKGLYMFHCCVFPKLTSTAPIYGLDVIAGAKKVTGFFHDFSPLAKKDHSMVDWFVKEASLYTPSKPRPLPDWAMKIFSPGMIAAGNINTEKELTQALSMAQSNLSVYFTLLRREKEQGDIQEIKDAQNRYAKHQRENPHTPRVMLSLGLPEDDVKEFCSDALFPYVE.

>3300012953_Ga163179_10000108

MLEGQVRNIISTFEREGKEIFEPTMKKFNRPEDGWVNRVWETPEARRCHIDVVDVRDTKGLYMFHCCVFPHLNSPAPIYGLDVIAGANKVTGFFHDFSPLAKRDHTMVDWFVKEAKNYEPSKVRELPEWALKIFSPGMIAASNIKEEKELNAALSLAQSNMGAYFTLLRRERNNPTKVTEQEIKDAQNRYAKHQRENPHTPRVMLSLGIPENDVKEFCTDALFPYVE.

>3300005521_Ga0066862_10001059

MAIYKRSRIWQMLEETTNYLTAVFDREGKEIFEPTMEKFNRPKDGWVNRVWETPEARRCHLDVVDARGTKGLYMFHCCVFPKLTHPGPIYGLDVIAGAKKVTGFFHDFSPLAKRDHSMVDWFVKEASNYKPSKVRELPDWAMKIFSPGMVAASNITQEKELNAALSLAQTNLGAYFTLLRREKGEGNIQEIKDAQNRYAKHQRENPHTPRVMKSLGLKDEDVEEFCTNALFPYVE.

>3300006166_Ga0066836_10000757

MAIYKRSRIWQMLEETTNYLTAVFDREGKEIFEPTMEKFNRPKDGWVNRVWETPEARRCHLDVVDARGTKGLYMFHCCVFPKLTHPGPIYGLDVIAGAKKVTGFFHDFSPLAKRDHSMVDWFVKEASNYKPSKVRELPDWAMKIFSPGMVAASNITQEKELNAALSLAQTNLGAYFTLLRREKGEGNIQEIKDAQNRYAKHQRENPHTPRVMKSLGLKDEDVEEFCTNALFPYVE.

>Marinovum

MKRGLVMAQRSRIWEMLEGQVRNIIATFEREGKEIFEPTMKKFNRPEDGWVNRVWETPEARRCHIDVVDVRDKKGLYMFHCCVFPHLNSPAPIYGLDVIAGANKVTGFFHDFSPLAKRDHTMVDWFVKEAKNYEPSKVRELPEWALKIFSPGMIAASNIKEEKELNAALSLAQSNMGAYFTLLRRERNNPTKVTEQEIKDAQNRYAKHQRENPHTPRVMLSLGIPEDDVKEFCTDALFPYVE.

>GROS21-1_SAMEA9560540

MTQTLFDKVINCADTINRKFAATGALGTVYRTDLITDKEFSSLRYRRAHISIVDARETKKLYLLHVTVFPHLNDPSPIFGFDIVCGPTKVSGAFHDFSWAGDKTSPMYLWFKAKVAGLEWNKPRELPEWGKQIFSPAMVAIGAVGEQELDEFIRIGLETLEFYLANVGIEQQDVATFEMAQNRYCHYQKQNPRTPASLVHLGLTEEEVSDFIANRLFPEVG*

>GROS21-1_SAMEA9560542

MSEIWNRLIDIENHYINRFTETGKEILTDDISALGWSSRSWSSDTYRLANIVTADVRETKGMWMMHCCVFPHMENTAPIFGFDVVAGKNKITGCFHDFSSTGFASHPLIEWFGHEVSQLEWRKTRELPEWAQRIFSPHIIAAANVNEGDELEQIISMSTRNLDHYIDTVGETDGDCTDNTKGQNYYCENQKLNPHNPRVMTNLGLSEEQIRFFIEQCMFPEA*

>GROS21-1_SAMEA9560546

MSKVWDTLINIQHLLEKEFDRTGTEQFEPGMDRFNQPGWINRVWSSPSYRRAHVDVVDARETKGLWMMHCCIFPHLHNPAPIFGFDVVAGKNKITGCFIDYSPTEDKFHHMLDYFGEEVSRYEWIKKRELPDWAKRIFSQHMVAAGNVSDESELAQISSLASILINHYLETVGETNNRVLDTSSYQNYYCDNQKQNPHTPRVMVSLGLSEDDVQHFIQECLFPEIR*

>GROS21-1_SAMEA9560586

MSKVWDTLINIQHLLEKEFDRTGTEQFEPGMDRFNQPGWINRVWSSPSYRRAHVDVVDARETKGLWMMHCCIFPHLHNPAPIFGFDVVAGKNKITGCFIDYSPTEDKFHHMLDYFGEEVSRYEWIKKRELPDWAKRIFSQHMVAAGNVSDESELAQISSLASILINHYLETVGETNNRVLDTSSYQNYYCDNQKQNPHTPRVMVSLGLSEDDVQHFIQECLFPEIR*

>GROS21-1_SAMEA9560612

MSKVWDTLINIQHLLEKEFDRTGTEQFEPGMDRFNQPGWINRVWSSPSYRRAHVDVVDARETKGLWMMHCCIFPHLHNPAPIFGFDVVAGKNKITGCFIDYSPTEDKFHHMLDYFGEEVSRYEWIKKRELPDWAKRIFSQHMVAAGNVSDESELAQISSLASILINHYLETVGETNNRVLDTSSYQNYYCDNQKQNPHTPRVMVSLGLSEDDVQHFIQECLFPEIR*

>GROS21-1_SAMEA9560623

MSHIWNKMQDCADVMRDKMLRMGEIDQDPILEKYNWENHVYRSSYFRRGHVEVVDQRENYGLFILHATVFPRVDSDAPIWGFDAVCGKNKITGAFHDFSLVDTEDNFMYQWFKDTTKDITWKKERELPDWAKAIFSPSMVAVGNVQDELEVDQFIKLGLDTLDYYISNANLCKIVGKDYTEQQNRYCYYQKQNPHVVRSMVAMGYEQHIIENFVEEVLFPEIK*

>GROS21-1_SAMEA9560794

MQTSNVWDTLINIQELLETNFGRTGTEIFEPGMDRFNQPGWVNRVWTSDLYRRAHVDVVDARDTKGLWMMHCCIFPHLHNDAPIFGFDVVAGKSKITGCFYDFSPAGDSEHPLCEWFADETSQLEWNKKRKLPEWAERIFSESMVAAGNIQKEEELEQIFQMAKRGVEHYLQAVGETNHTASSTKDAQNYYAQNQKQNPHTPKVMVSLGLTEEDVTVFIQECLFPEIK*

>GROS21-1_SAMEA9560823

MSNTFEQTIAAAQQIEKRFRDTGECIEVGSTDFGLNDRVFSSMRYRRAHLCTVDARETKKLYLLHCTIMPHTNDNSPIYGFDIVCGPTKVSGAFHDFSEAGAKNHPMYEWFNAYTSELSWNRPRELPDWAKEIFSPSFVAIGAVGPEELASFITVGLQSLDYYLAHVGNTQESGADFHMAQNRYCQYQKMNPRTFTSLQHLGFTEQQAIDFVKDSLFPEL*

>crystal_bog_phage_scaffold_2_70

VTDYIELLDKTARQLNHIISAQPDVIPMETPEYGWENHRHSSPQFRMAHVEIFNQDRFMVVHCCVFPHVSDPAPIFGFDVIASQNKVTGVFLDLSPTVEEPGKFHNLTFKQQRERPQWGDIFSQNWIACRPDKTEMMSIVAESQRLLKHYLTNIINKRTAPVDAIREGQNRYCKQQQQNEHTLKALKNLIGPSRAREFMNTILFPTV*

**gp13 protein**

>MOSIG

MATPATRETLKQYALRALGKPVIEINVDDDQLEDRLDEALQYFAQYHYDGIQRAYLKYQYTSADKARITGNSSETVTKNSVTTSWTEGNNFLVVPESVISVINIFPFSNKSNMNLFDVRYQLRLNDLYDFSSTSVINYDIVLRHLDFLDHILVGEKPLRFNQHDNRLYIDMDWTNDLAVGEYLVIEAYRKLDPETHTDVYNDIFLKRYVTALFKKQWGANLSKFDGVAMIGGVTLNGRQIYSEALSDIEKLEQEIRSTFELNPAMMIG

>SYN9

PASKTELKNYALRRLGYPAIDINVCDEQLDDLVEEAIDYFQEYHYNGSYQSMMKIEVTDAIKTAAQSTTQQGSTNWYENNNYVSLPPGVLGVNHVYTNIGASSVVPGNIFNIKYQIFLNDIYAMTHGQILHYFMTSQYLETLDWVTNSQANRRVKFNEHQARLYLDFDWDTLQAGDFIMVDLTMRQDPDEYTGMYNDAWLKDYVEALFQQQWGRNLSKYDGIQMLGGVTLNGRQILEDASQFKKDLEENIRKEYELPPMDLIG

>GOS_EDB19297

PASKTELKNYALRRLGYPTIDINVADEQLDDLIEEAIDYYQEYHYSGSYNSFMKIEVTDAIKTAAMSSTQQGATNWYENNNYVDLPPGVLSINHVYTNIGASSVVPGNIFNIKYQIFLNDIYAMTHGQILHYFMTSQYLETLDWVTNSQANRRVKFNEHQARLYMDFDWDTLQAGDYILVDLHMRQDPEVYTSMYNDNWMKDYVEALFMQQWGRNLSKYDGIQMLGGVTLKGRDILEDANKRKEDLEKEIRETYELPPMDLIG

>GOS_EBD35787

PASKTELKNYALRRLGYPAIDINVCDEQLDDLIEEAIDYFQEYHYNGSYKTFMTIEVTDAIKTAAQSSTQQGSTNWYENNTYVDLPPGCMGVNHVYTGISTSSIVPGNIFNIKYQIFLNDIYAMTHGHILHYFMTSQYLETLDFVTNSQANRRVRFNEEQGRLYLDFDWNDLQSGDYIMVEMLMRQDPETYTAMYNNNWLKDYVEALFQQQWGRNLSKYDGIQMLGGVTLNGRQILEDASQFKKDLEEEVRSTYELPPLDLIG

>PSSM4

PASKTELKDYALRRLGFPTIDINVATEQLDDLVEEAIDYYQEYHYNGSFQTFMRIEVTEAIKTQAKGFTQEGSTPWYGQDNYVSTPPGTLGINHVYTNIGASSIVPGNIFNIKYQIFLNDIYSMTHGQILHYFLTSQYLETLDFVTNSQANRRVKWNEHSNRLYLDFDWDDLTVGDYIMVDMTMRQDPTTFTDMFNDNWLKDYVEALFQQQWGRNLSKYDGIQMLGGVTLNGRQILEDASKFKEDLEKDIRDRYEIPPLDLIG

>GOS_EDG13586

PASKTELKDYALRRLGFPTIDINVATEQLDDLVEEAIDYYQEYHYNGSFQTFMRIEVTEAIKTQAKGFTQEGSTPWYGQDNYVSTPPGTLGINHVYTNIGASSIVPGNIFNIKYQIFLNDIYSMTHGQILHYFLTSQYLETLDFVTNSQANRRVKWNEHSNRLYLDFDWDDLTVGDYIMVDMTMRQDPTTFTDMFNDNWLKDYVEALFQQQWGRNLSKYDGIQMLGGVTLNGRQILEDASKFKEDLEKDIRDRYEIPPLDLIG

>GOS_ECZ20225

PSSKTELKDYALRRLGYPTIDINVATEQLDDLVEEAIDYYQEYHYNGSYKAFLRIEVTEAIKNNAQAYSQEGSSAWYGINNYVDTAPGTLGINHVYTSIGASSIVPGNIFNIKYQIFLNDIYAMTHGHILHYFLTSQYLETLDWITNSQANRRVKWNEHQGRLYLDMDWSDFEVGDYILVDCTMRQDPDTYTGMYNDNWLKDYVEALFQQQWGRNLSKYDGIQMLGGVTLNGRQILEDGSTFKSDLEKELRDRYEIPPMDIVG

>Unc_Med_phage_BAR36616

PATRETLKQYALRALGKPVIEINVDDDQLEDRLDEALQYFAQYHYDGVQRAYLKYQYTQADKDRMTADSTESITKNSVTTSWKEGNNFLVVPESVISVINIFPFSNKSNMNLFDVRYQLRLNDLYDFSSTSIINYDIVLRHLDFLDHILVGEKPLRFNQHDNRLYIDMDWKNDLAVGEFIVIEAYRKMDPETHTDVYNDIYLKRYVTSLFKKQWGANLSKFDGVAMIGGVTLNGRQIYSEALQDIEKLETEIRSTFELNPAMIMG

>Unc_Med_phage_BAR36227

PATRENLKQYALRALGKPVIEINVDDDQLEDRIDEAIQYFAQYHYDGVKRTYLKYQYTAADKARMTADASETATIGSDTTTWKEGQNWLAIPSSILSVINIFPFSSKGSLNLFDVRYQLRLNDLYDFSSTSVVNYDVVLRHLDFLDHILVGEKPMRFNQHENKLYIDMDWKNDLAVDEYLVIECYRKLDPNTNTDVYNDIFLKRYVTALFKRQWGANLSKFGGVQMIGGVTLNGQEIFSQALQDIDKLEQEIRSSYELNPAMMIG

>Unc_Med_phage_BAR31202

ASTRQGLIDYALRQNGAPVLEINIEDDQIDDLVDDAIQFYNERNSDGYIRTHVKIKWTADMITNMTTDTTTSIASGTSNALPVSYLEQNNFVKMPDHITSVIKVFPFVSKNVTNLFDVRYQWRLNDLWDLTNTEILTYEMVNRRLEDIYFLLEGQKQTRFQLRGNKLYLDLDWKTDVKEDDFLVLECYRAVDPTADTDVYNDIWMKRYVSALVQRQWGANLIKFQGAQLPGGITMNGEFIYNEGKEKVAKLEEEMLTVYETMPMDMIG

>JCVI_SCAF_1096627235596_Split

PATRETLKQYSLRALGKPVIEINVDDDQLEDRIDEAVQYFQQFHSDGIRRTYLKYKLTAADKTRLSGLNQESESKTDLKDSSVSTTWYEDKNYLVVPETVLSIINIFPFSNKGTLNLFDVRYQMRLNDLYDFSSTSMVNYDVVLRHLDFLDHVLVGEKPMRFNQHDNRLYIDMDWKNDLEEDEWLVIECYRRLDPNTYTDIFNDIYLKRYVTALFKKQWGANLSKFNGVAMVGGVTLNGQQIYTEALADIEKLETEIRTTYELNPAFMIG

>GOS_EDE85049

PATRETLKQYSLRALGKPVIEINVDDDQLEDRIDEAVQYFQQFHYDGIRRTYLKYKLTAADKTRLSGLNAESESATDLKDTSVSTTWYEDKNYLVVPESVLSVINIFPFSDKGNLNLFDVRYQLRLNDLYDFSSTSVINYDVVLRHLDFLDHILVGEKPMRFNQHDNRLYIDMDWKNDLDTDEWLVIECYRKLDPNTYTDVFNDIYLKRYTTALFKKQWGANLSKFNNVAMVGGVTLNGQQIFSEACDDIAKLETEIRTTYELNPAFMIG

>GOS_EDD04697

PATRETLKQYSLRALGKPVIEINVDDDQLEDRLDEAIQYFQQYHYDGIRRTYLKYQLTAADKTRLAAINGSSETATKNSVSTTWYEDNNFLVVPDSVISVINIFPFSDKGNMNLFDVRYQLRLNDLYDFSSTSVINYDVVLRHLDFLDHILVGEKPLRFNQHDNRLYIDMDWTNDLTTDEWIVIECYRKLDPSTYTDVFNDIYLKRYTTALFKKQWGANLSKFNGVAMVGGVSLNGQQIYSEALSDIEKLENEIRTTYELNPAMMMG

>JCVI_SCAF_1096626956256_Split

PSTRETLKQYSLRALGKPVIEINVDDDQLEDRIDEAVQYFQQYHYDGIRRTYLKYKLTAADKTRLSAINGETESATDLKDNSVSTTWYEDRNFLVVPQSIISVINIFPFSDKGNLNLFDVRYQLRLNDLYDFSSTSVINYDVVLRHLDFLDHILVGEKPLRFNQHDNRLYIDMDWTNDLETDEWIVIECYRKLDPNSYTDVWNDIYLKRYTTALFKKQWGANLSKFGGVAMVGGVTLNGQQIYSEALQDLEKLETEIRTTYELNPAMMIG

>HTVC008M

PITSRETLKQYCLRALGKPVIEINVEDDQVEDRIDEAVQYFAQYHYDGSERMYLKYQVTADDITRARSNETLSTVTDTADSTVTSSFKEGKNYIPMPSNVMSVLQVFPFTDKAALNLFDVRYQLRLNDLYDFSSTSIIHYDMTLRHLDMLDHILTGERPIRYNQHKNRLYIDMDWAHDVKAGDYLIIECYRKLDGSTFTDLFDDIFLKKYLIQLIKKQWGTNLSKFQGVAMLGGVQMNGEQIYSQAQEEINKLEEQIQLSFELPPNYMVG

>Unc_Med_phage_BAR35761

PNSKETLKQYALRQLGKPVIEINVDDDQLDDIIDDALQYFAEYHYDGTIRTYLKHQINDNDLVNQKADASMAQSSTGTHISSNMTFKEGQGYVVLPESVYSVLRVFPFVDKSGLNMFDLRYQLRLNDLYDISSTSIVQYEMVQNHIQLLDEILIGQVPVRFNKAQNRLYLDMDWSAAVTSGEYIIIDCYRKIDPTQFTDVYNDVWLKKYVTALIKRQWGQNLSKFEGVQLPGGVTLQGRQILEDANTEIEKLEEQSNLLQTESAIMMG

>Unc_Med_phage_BAR31834

PTTKAELKEYCLRRLGKPVLEVNVSDDQIDDAIDYSIQKFQTYHYEGAERVYLKHLFTADEIAAGRTNTDTTGADGTTTWSEQNTYLTVPEHILAIEGMFAFTDKGTRSMFDIRYQMRLNDLYDFTSTQFYHYYMIQQHLSSIDFMLEGLKPIRYNNVQDRVYIDFDWTEDALSDQYVILKCWRALDPTTWTEIYNQMWLKDYATAKIKKQWGQNLTKFTNVQMPGGVTLNGEMIYNDAVEELKILDEQLRTTWETPPLDMIG

>PRSM6

PTSKSELKEYCLRKLGKPVLEINVSDDQIDDAIDYTIQKFQQYHYDGAERVYLKHKFTSAEIAAGKASVASTGVDGSTEWGEQTNYLSVPDHVISVEGLFGFTDKGTRNMFDIRYQLRLNDLYDFTSTQFYHYYMIQSHLSSIDWILEGLKPIRFSTVQNRLYIDFDWTEDSLEDQYIVIKCWRALDPTTWTEIYNQMWVKDYAAARIKKQWGSNMTKFQNVQMPGGVTLNGEMIYNDAVEELKILDEQLRQVWETPPLDMIG

>Unc_Med_phage_BAR35393

PTSKAELKEYALRRLGKPVLEVNVSDDQIDDAIDYTIETFQEFHYGGSEKVFLKHQFTAEDMTRFKADASETGTDTLQAGNTGTVFKTQNNYLILPDHVLAVNGIFTFTDKGTRNMFDIRYQMRLNDLFDFTSTQFYHYYMIQTHLETINFLLEGMKPTRFNATQGRLFIDFDAQTDAQEGSYVVIDCVRALDPLNWSKIYGTLWVKDYTTAMIKKYWGQNLTKFQNVQLPGGVTLNGEKIYSDAITELEQLDEKLRSTYEMPPLDMIG

>Unc_Med_phage_BAR31897

PASRSELIDYCKRQLGAPVLEINVADEQMDDLVDDALQLFQERDYDGSTNTFLKYKITQADIDRGRGRGGTNPVGIVTTTASSNINGSNVSFSFEENSNYLQVPPEVLGVTKIFHFSGANTVANNMFSIKYQLFLNDIYYFGSTEILTYAMTKRYLEDIDFALTTQKQIRFNIRQDRLYLDIDWSSVNADDYLVLDCYRLLDPNDFPRVYNDSFLKRYLTALIKRQWGQNLIKFQGVKLPGGVELNGRQIYDDAERELDKIREVMSNTYELPPLDMIG

>Unc_Med_phage_BAR36595

PSTRQELIDYCLRKLGAPVLEINVAEEQVDDLVDDAVQFFQERHFDGVYPAFLKYQVTEDDIKRGKGEVGISTTTTTAAITGSSINFDYEENSNYIQVPPHVIGVKKIFQFEGDNSVSSGMFSVKYQLFLNDVYNFSSIELLTYSMVKTYLEDINFLTSTQKQIRFNKRQDRLYLDIDWNSINKNQYLIIDCYRMMDPSDYAKVWNDSFLKQYVTALIKKQWGQNLIKFQGVKLPGGTELNGRQLYDDGQREIDTLMDKMSSYYELPPLDMIG

>GOS_EDE74209

PANRQDFIEYCLRKLGAPVLEINVDDEQIEDAVDDGIQMFNERHFDGVERMYLKYQISQDDIDRGRAINDSGSTNTAGIVTTVGTSSTITGYGATTSNWYETSNFLQVPDSVVGVEKIFKFDSSSISSGMFSIKYQLFLNDLYYFNSVELLQYAMTKTYLEDIDFLLTPDKQVRFNKRQGRLYLDMDWGSQTVDNFLVLDCFRALDPEDFNQIYNDIFLKEYVTLLIKRQWGQNMIKFRGVKLPGGIEMNGREIYDDAERSLEALRERIKLEYELPPLDFIG

>GOS_EBA99735

PTTREELKDYCLRQLGAPVLEINVADEQVDDLLDDTIQYFNERHFDGVEKTYLKYKISQEDIDRGRGSGGGTVGVTTTGVGIVTTTGTSTNIAGLGTITSNFYETSNFIQVPDSVIGIEKIFKFDTSSISGGMFSIKYQLFLNDLYYFNSVELLQYAMTKTYLEDIDMLLTTDKQVRFNQRQNRLYLDIDWKAQTAGNYLVIECYRALDPENYSKIFNDSFVKRYLTAAIKRQWGQNLIKFQGVKLPGGIELNGRQIYDDGQRELDEIRQRMSSDYELPPMDLIG

>PSSM2

PASRQQLIDYCLRKLGAPVLEINLDDDQIDDSVDDAIQLFNERHFDGVERMFLKYKITQADLDRGRAKGTDGVGIVTTTATSTNIAGYGTTTSSWYETSNFLQVPDSVVGIEKIFKFDTSTISGGMFSIKYQLFLNDLYNFNSVELLQYSMVKSYLEDIDFLLTTDKQVRFNKRQDRLYLDIDWGAESLDNFLVLDCYRALDPTSFTQVYNDPFLKLYLTALMKRQWGQNLIKFRGVKLPGGIELNGREIFDDAERDIESLRSRMASEYELPPYDFVG

>Unc_Med_phage_BAR32978

PSSRDGLIDYAKRQLGYPVLEINVADEQFSDLLDDSVQVYQERHYDGIMRMYLKYQITQEDIDRGQARGSNKSAGITTTTGTSTVGLTTTFDFEENQNYLQMPPSVIGVNQIFKIRSDTVYDGLFNIRYQLFLNDLYQFGSINMLQYSMVQTYLEDLTFLLNPDMRYRFNIRQDRLYIDADFGVLNVGDYFIIDCFRILDPNDFTRVYNDPFLKRYFTALCKKQWGQNLIKFQGVQLPGGVQLNGRQIYDDGELAEIRAKMSSDYEMPPLDMIG

>Unc_Med_phage_BAR30812

PSTRQELIDYCLRKLGFPVLEINVDDDQIEDLVDDAIQFFQERHFDGSIKTFLKLEVTEQMITDAKANSTISGTDFKEQNNYVTVPEHVLGVTQVYAYDNSSSAVSGNIFSMKYQLFLNDFYNFGSMEILNYYMVKQYLETLDFVIGNFKPVRFNKRENRLYIDTDWDNITPGQHLILDCYRMIDPTNASEVYNDKWLKRYLTALIKRQWGQNLIKFKNVALPGGTTLNGREFYEDAQREIQMIMDDFKLEYELPPLDMIG

>Unc_Med_phage_BAR27633

PASRSELKDYCLRQLGAPVLEINVDDDQIDDAIDNALQYYRERHFDGVERMYLKHQFTDADKTKFETPQTQTDTINGTDWERTDNYLDIPPHIVGISKVFGLNSNTIRNNLFGLEYQIFLNDLYAFGSLDILNYFMIKQYLETMDMVLNNGSFIEYRFNQRQDRLYLDVDASMIDPDNYLIIDCYRALDPDSFVQTYNDPFVKRYATSLIKKQWGQNLIKFNGVTLPGGVSLNGRELYQDAITEIAEMMAASASTFELPPLDMIG

>SPM2

PSSKQELIDYCKRQLGAPVLQINTDSAQDDDIIDQAIQYYHEYHFDGIERMYLKHQFTADDVTRFTSSDQLTTAPNNDDWENRNNYIQVPDAVIGISKVFGVSSNFLRNNLFGLSNQYYLMDLFSFSTGSAFSFGNFDLTNYYMIKQHFETIDMIINTGSLIEYRFNKRQDRLYIDIDTSRIVEDQYLLIDCYRYLNPDDFTQVYDDSFVKRYATALLKRQWGQNLIKYNSVQLPGGITLNGRQIWEDGNNEVRELESRMMTDYSLPPMDMIG

>Unc_Med_phage_BAR29030

PNSRADLITYCKRQLGEPVLQVNVDDEQVNNVIDDTVQFFQENCYNGMERAFLYHELTEDDKTRFAASVSTTKTDGSDTVTWKETTNYIPIPSHVTGISKVFGLVSNSIRSNLFGIEYQMFLNDLYAFGSLDILNYYMTKQYLETLDMVLNNGSFQQFRFTARRDRLYMDLDKDFLKKESNILIECHRMIDPNDATEMYNDLFVKRYATALLKKQWGQNLIKYNNVQLPGGITLNGRELYTDALVEIEKIESEVLSKYAIPPMDMIG

>Unc_Med_phage_BAR28986

PNSRADLITYCKRQLGEPVLQVNVDDEQVNNVIDDTVQFFQENCYNGMERAFLYHELTEDDKTRFAASVSTTKTDGADTVTWKETTNYIPIPSHVTGISKVFGLVSNSIRSNLFGIEYQMFLNDLYAFGSLDILNYYMTKQYLETLDMVLNNGSFQQFRFTARRDRLYMDLDKDFLKKESNILIECHRMIDPNDATEMYNDLFVKRYATALLKKQWGQNLIKYNNVQLPGGITLNGRELYTDALAEIEKIESEVLSKYAIPPMDMIG

>SRSM4

PSSRADLITYCKRQLGEPVLQVNIDDEQVNNVIDDTIQFFQENCYNGMERAYLRHKINADDLTRFDGEDTTASGTTDWEEATNYIPIPDHVVGVTRVFGLVSNSIRSNLFGVEYQLFLNDLYAFGSLDILNYYMNKQYLETLDMVLNNGSFQQFRYTMRRDRLYLDINKAFLKEDTWLLIEAHRLIDPNDATEMYNDMFVKRYATSLMKKQWGQNLIKFNNVQLPGGITLNGRELYTDALAEIEKIESEVLSKYAIPPMDMIG

>Unc_Med_phage_BAR29483

PSTRTELQNYCKRQLGEPVLQVNVAQEQIDDLTDDALQKFAEWTYNGTEKMMLKHEITEDDVTRFKSQNQTTSVSGSEWTERDNYIPIPEHVYGINRIFGIKSSGIRGALFGIEYQIFLNDLYHFGAVDILNYYMTKSYLETLDFVLNNGTFIQYRWNQRQDRLYLDTAAEDIKKGEFLIIECYRALDPTTYTQIYNDPFLKKYLTALVKRQWGTNLTKYTGVQLPGGISLNGEKIYTEAVAEVEKIESEILSTYALPPYDLIG

>Unc_Med_phage_BAR31854

PSTRQGLIDYALRQNGAPVLEINIEDDQISDLVDDAIQFFNERHMDGYIRTHLKVQYSQLMLDDMTTDTDTTVSSGTSSGQTVTFKEQNNYIKMPPYVTSVIKCFDFVSKNVTNLFDVRYQWRLNDLWDLTQTEILTYEMVNRRLEDIYYLLEGQKQIRFQRRGDRLYLDLDFKTDVPADQFLVLECYRAVDPTQFEDVYNDVWLKRYVTALIQRQWGANLIKFQGAQLPGGITMNGEFIYNEGKEKVAKLETEMLTQYEMPPLDMIG

>Enterobacteria_phage_T4

PQNPKELKDVILRRLGAPIINVELTPDQIYDCIQRALELYGEYHFDGLNKGFHVFYVGDDEERYKTGVFDLRGSNVFAVTRILRTNIGSITSMDGNATYPWFTDFLLGMAGINGGMGTSCNRFYGPNAFGADLGYFTQLTSYMGMMQDMLSPIPDFWFNSANEQLKVMGNFQKYDLIIVESWTKSYIDTNKMVGNTVGYGTVGPQDSWSLSERYNNPDHNLVGRVVGQDPNVKQGAYNNRWVKDYATALAKELNGQILARHQGMMLPGGVTIDGQRLIEEARLEKEALREELYLLDPPFGILVG

>ArctO_Ga0115001_100000

MAIPNTRQTLISYAKRALGHPVIEINVDDDQVDDRVDEAIQYYQQYHYDGIKRVYLKYQYTQADKTRILSDTSEGVTKNSVTTTWKTGNGYIIVPDSIVGVQNIFPFSSKGSLNLFDVRYQLRLNDLYDFSSTSVVNYDIVMRQLDFLDHILVGEKPLRFNQHDNKLHIDMDWGSDLQVGEYLVIDAYRKLDPDTYTDVYDDIWLKRYTTALVKKQWGANLSKFNGVAMIGGVTLNGQQIYSEALQDVEKLETEIRNSFELNPAMLIG.

>AtlO_Ga0163179_10000108

MATPNTRQTLISYAKRALGHPVIEINVDDDQIDDRIDEALQYYQQYHYDGIRRTYLKYQYTESDKTRILSNASDSGVKNSVTTTWKEGNNYIVVPETVLSVINIFPFSNKGNLNLFDVRYQMRLNDLYDFSSTSVINYDIVMRQLDFLDHILVGEKPLRFNQHDNRLYIDMDWENDLQVGEYIVIEAYRKLDPDTYTDVYNDIWLKRYTTALVKKQWGANLSKFNGVAMIGGVTLNGQQIYSEALQDVEKLETEIRSSFELNPAMLIG.

>AtlO_Ga0115012_10000076

MAQPNTRQTLIAYAKRALGHPVIEINVDDDQIDDRVDEALQYYQQYHYDGIKRVYLKYEYTQADKTRILADSSEGATKNSVTTTWKSGNNYIVVPESVISVTNIFPFSNKGNLNLFDVRYQLRLNDLYDFSSTSVINYDIVMRQLDFLDHILVGEKPLRFNQHDNRLYIDMDWANDLQVGEYLVIDAYRKLDPDTYTDVYDDIWLKRYTTALVKKQWGANLSKFNGVAMIGGVTLNGQQIYSEALQDVEKLETEIRNSFELNPAMLIG.

>EPO_Ga0066836_10000757

MAQPNTRQTLISYAKRALGHPVIEINVDDDQIDDRVDEALQYWQQYHYDGIKRTYLKWQYTQAEKNRILTSNSEAGTKNSVTSTWKEDNNYIVVPETVFSVTNIFPFSNKGNLNLFDVRYQLRLNDLYDFSSTSVINYDVVMRQLDFLDHILVGEKPLRFNQHDNRLYIDMDWENDLMIDEYIVIECYRKMDPDTYTDVYNDIWLKKYTTALVKKQWGANLSKFAGVAMIGGVTLNGEQIYTQALADIEKLEEEIKSLQEHQALMIG.

>T.Oceans_St.125_SAMEA2622841_6527

MAQPNTRQTLISYAKRALGHPVIEINVDDDQIDDRVDEALQYWQQYHYDGIKRTYLKWQYTQAEKNRILTSNSEAGTKNSVTSTWKEDNNYIVVPETVFSVTNIFPFSNKGNLNLFDVRYQLRLNDLYDFSSTSVINYDVVMRQLDFLDHILVGEKPLRFNQHDNRLYIDMDWENDLMIDEYIVIECYRKMDPDTYTDVYNDIWLKKYTTALVKKQWGANLSKFAGVAMIGGVTLNGEQIYTQALADIEKLEEEIKSLQEHQALMIG.

>T.Oceans_St.109_SAMEA2622357_9342

MATPNTRQTLISYAKRALGHPVIEINVDDDQIDDRVDEALQYYQQYHYDGIRRTYLKYQYTQADKTRILTDTSEGVTKNSVTTTWKEGNSFIVVPESVLSVINIFPFSNKGNLNLFDVRYQLRLNDLYDFSSTSVINYDIVLRQLDFLDHILVGEKPLRFNQHDNRLYIDMDWENDLQVGEFLVIEAYRKLDPDTYTDVYNDIWLKRYTTQLIKKQWGANLSKFNGVAMIGGVTLNGQQIYSEAMQDIEKLETEIRNSFELNPAMMIG.

>T.Oceans_St.64_SAMEA2620836_7330

MATPNTRQTLISYAKRALGHPVIEINVDDDQIDDRVDEALQYYQQYHYDGIRRTYLKYQYTQADKTRILTDSSEGVTKNSVTTTWKEGNAYIVVPESVISVINIFPFSNKGNLNLFDVRYQLRLNDLYDFSSTSVINYDIVLRQLDFLDHILVGEKPLRFNQHDNRLYIDMDWENDLQVGEYLVIEAYRKLDPDTYTDVYNDIWLKRYTTQLIKRQWGANLSKFNGVAMIGGVTLNGQQIYTEALQDIEKLETEIRTSFELNPAMMIG.

>T.Oceans_St.65_SAMEA2620861_832352

MATPNTRQTLISYAKRALGHPVIEINVDDDQIDDRIDEALQYYQQYHYDGIRRTYLKYQYTEADKTRILSNANDSGTKNSVTTNWKEGNNYIIVPETVLSVINIFPFSNKGNLNLFDVRYQMRLNDLYDFSSTSVINYDIVMRQLDFLDHILVGEKPLRFNQHDNRLYIDMDWENDLSVGEYIVIEAYRKLDPDTYTDVYNDIWLKRYTTALVKKQWGANLSKFNGVAMIGGVTLNGQQIYSEALQDVEKLETEIRSSFELNPAMLIG.

>P-TIM40

MAQPNSKATLKEYCLRRLGKPVLEINVSDDQVDDAIDYTLQKFQQYHYDGAERCYLKHKVTQDLIDRSESNTTSTSKAGNDTWTEGNGYIEIPDHILSVEGIFSFTDKGTSNMFDIRYQMRLNDLYDFTSTQFYHYYMIQQHLSTIDFLLEGIKPVRYHAVQDRLYIDFDWPQDAQLDQYIVIKAWRALDPETWTEIYNQMWVKDYASAKIKKQWGQNLTKFQGVQMPGGVTLNGEMIYNDAVEELKNLDEQLRTTYETPPLDMIG

>S-CBM2

MATPNSKATLIEFCKRRLGAPVLEINVASEQLDDAIDYTLEKFRTFNYDGIEKCYLKHKWTSDDVTRFKSDEVVETATQGSVSSDWTSQQNYLVVPDEVLSVSNVWSTTDKGTGNIFDIRYQIRLNDLYDFTSTQFFHYYIIQQHLANIDFLLEHFKPVRYTHVSDRLYIDLSATEDVIKDEWTIIECYRYLDPATYTRIYNNMWVKDYATALIKKYWGQNLTKFNGVQLPGGVTLNGEKIYDDAVQELEKLEGTLRDTYEMPPLDAVG

>S-WAM1

MASPTSKAELKEYCLRRLGKPVLEVNVSDDQCDDAIDYSIQKFQQFHYEGAERVYLKHQFTTAEIAAGKANTDSIAVDGTTIWKEQNAFLSVPEHILSIEGMFAFTDKGTRSMFDIRYQMRLNDLYDFTSTQFYHYYMIQQHLESIDFILEGMKPIRYNQVQDKLYLDFDWSEDALEDQYVIIKCWRALDPNTWTEIFNQMWLKDYATAKIKKQWGQNLTKFQNVQMPGGVTLNGEMIYNDAVEELKILDEQLRTTWETPPLDMIG

>MED640_133956

MATPNTRQTLISYAKRALGHPVIEINVDDDQIDDRIDEALQYYQQYHYDGIRRTYLKYQYTQADKTRILSNASDSGVKNSVTTTWKEGNNYIVVPETVISVINIFPFSNKGNLNLFDVRYQMRLNDLYDFSSTSVINYDIVMRQLDFLDHILVGEKPLRFNQHDNRLYIDMDWENDLQVDEYIVIEAYRKLDPDTYTDVYNDIWLKRYTTALVKKQWGANLSKFNGVAMIGGVTLNGQQIYSEALQDVEKLETEIRSSFELNPAMLIG.

>ArctO_Ga0114999_10006015_Split

MAKPASRENLKQYALRALGKPVIEINVDDDQMEDRLDEALQYFAQYHYDGVKRTYLKYKYTAEDKARALSQSSETASKTYGDSTTVSSTWQESNAYIIVPETVLSVVNIFPFSSKGNLNLFDVRYQLRLNDLYDFSSTSVINYDVVLRHLDFLDHILVGEKPMRFNQHDNRLYIDMDWKNDLQVDEFLVIECFRAVDPEQFTDVYNDLFLKKYVVALFKKQWGANLSKFDGVAMLGGVTLNGKDIYTQAMDDIENLEQRIRSQYELNPTFMIG

>EPO_Ga0066853_10000534_Split

MAEPASRETVKQYALRALGKPVIEINVDDDQLEDRLDEALQYFAQYHYDGVKRTYLKYKYTAADKARILADSTETESKTYGDSSVVNTEWKEGNQYIVCPESVISVINIFPFSNKGNLNLFDVRYQLRLNDLYDFSSTSVINYDVVLRHLDFLDHILVGEKPYRFNQLDNRLYVDMDWKNDLQVDEFLVIECWRKLDPNTYTDVFNDIWLKRYVTALFKKQWGANLSKFDGVAMLGGVTLNGKQIYSEALEDLDKLEIKLRSEFEEPQPFMIG

>ALOHA_st_Ga0068469_1063893_Split

MAEPASRETVKQYALRALGKPVIEINVDDDQLEDRLDEALQYFAQYHYDGVKRTYLKYKYTAADKARILADSTETESKTYGDSSVVNTEWKEGNQYIVCPESVISVINIFPFSNKGNLNLFDVRYQLRLNDLYDFSSTSVINYDVVLRHLDFLDHILVGEKPYRFNQLDNRLYVDMDWKNDLQVDEFLVIECWRKLDPNTYTDVFNDIWLKRYVTALFKKQWGANLSKFDGVAMIGGVTLNGKQIYSEALEDLDKLEIKLRSEFEEPQPFMIG

>ALOHA_st_Ga0068471_1051077_Split

MAEPASRETVKQYALRALGKPVIEINVDDDQLEDRLDEALQYFAQYHYDGVKRTYLKYKYTAADKARILADSTETESKTYGDSSVVNTEWKEGNQYIVCPESVISVINIFPFSNKGNLNLFDVRYQLRLNDLYDFSSTSVINYDVVLRHLDFLDHILVGEKPYRFNQLDNRLYVDMDWKNDLQVDEFLVIECWRKLDPNTYTDVFNDIWLKRYVTALFKKQWGANLSKFDGVAMLGGVTLNGKQIYSEALEDLDKLEIKLRSEFEEPQPFMIG

>ALOHA_st_Ga0068480_1108826_Split

MAEPASRETVKQYALRALGKPVIEINVDDDQLEDRLDEALQYFAQYHYDGVKRTYLKYKYTSADKARILADTTETESKSYGDSSVVNTEWKEGNQYIVCPESVISVINIFPFSNKGNLNLFDVRYQLRLNDLYDFSSTSVINYDVVLRHLDFLDHILVGEKPFRFNQLDNRLYVDMDWKNDLQVDEFLVIECWRKLDPNTYTDVFNDIWLKRYVTALFKKQWGANLSKFDGVAMLGGVTLNGKQIYSEALEDLDKLEIKLRSEFEEPQPFMIG

>ALOHA_st_Ga0068481_1058515_Split

MAEPASRETVKQYALRALGKPVIEINVDDDQLEDRLDEALQYFAQYHYDGVKRTYLKYKYTSADKARILADTTETESKSYGDSSVVNTEWKEGNQYIVCPESVISVINIFPFSNKGNLNLFDVRYQLRLNDLYDFSSTSVINYDVVLRHLDFLDHILVGEKPYRFNQLDNRLYVDMDWKNDLQVDEFLVIECWRKLDPNTYTDVFNDIWLKRYVTALFKKQWGANLSKFDGVAMLGGVTLNGKQIYSEALEDLDKLEIKLRSEFEEPQPFMIG

>ALOHA_st_Ga0099957_1046306_Split

MAEPASRETVKQYALRALGKPVIEINVDDDQLEDRLDEALQYFAQYHYDGVKRTYLKYKYTSADKARILADTTETESKSYGDSSVVNTEWKEGNQYIVCPESVISVINIFPFSNKGNLNLFDVRYQLRLNDLYDFSSTSVINYDVVLRHLDFLDHILVGEKPFRFNQLDNRLYVDMDWKNDLQVDEFLVIECWRKLDPNTYTDVFNDIWLKRYVTALFKKQWGANLSKFDGVAMIGGVTLNGKQIYSEALEDLDKLEIKIRSEFEEPQPFMIG

>Subarc_PO_Ga0098058_1000146_Split

MAIPNSKSTLKDWCLRKLGAPVLEINVDDDQVDDRIDEALQFFYTFQYSGMERCYLKHLITEADVTRSGTDESETATDIKDSGITTDWKTGKGFLVMPDAVQSVLRVLPFSDRGNLNMFDVRYQLRLNDLYDFSSESVIHYQMTMWHLDYLDMILIGEKPLQFNTHKNRLYINMDWGDDVQVGEHIIIECYRKLDPTTWTDVYDNLWLKRYSTALIKKQWGENLIKFQGVTMLGGVTMNGETIYNDAKDTIIKLEEESKTTWEEPLHFDIG

>ArctO_Ga0114995_10000109

MAIPNTRQTLISYAKRALGHPVIEINVDDDQVDDRVDEAIQYYQQYHYDGIKRVYLKYQYTQADKTRILSDTSEGVTKNSVTTTWKTGNGYIIVPDSIVGVQNIFPFSSKGSLNLFDVRYQLRLNDLYDFSSTSVVNYDIVMRQLDFLDHILVGEKPLRFNQHDNKLHIDMDWGSDLQVGEYLVIDAYRKLDPDTYTDVYDDIWLKRYTTALVKKQWGANLSKFNGVAMIGGVTLNGQQIYSEALQDVEKLETEIRNSFELNPAMLIG

>ArctO_Ga0115007_10001227_Split

MAKPASRENLKQYALRALGKPVIEINVDDDQMEDRLDEALQYFAQYHYDGVKRTYLKYKYTAEDKARALSQSSETASKTYGDSTTVSSTWQESNAYIIVPETVLSVVNIFPFSSKGNLNLFDVRYQLRLNDLYDFSSTSVINYDVVLRHLDFLDHILVGEKPMRFNQHDNRLYIDMDWKNDLQVDEFLVIECFRAVDPEQFTDVYNDLFLKKYVVALFKKQWGANLSKFDGVAMLGGVTLNGKDIYTQAMDDIENLEQRIR*QYELNPTFMIG*

>AtlO_Ga0115011_10000306

MAQPNTRQTLIAYAKRALGHPVIEINVDDDQIDDRVDEALQYYQQYHYDGIKRVYLKYEYTQADKTRILADSSEGATKNSVTTTWKSGNNYIVVPESVISVTNIFPFSNKGNLNLFDVRYQLRLNDLYDFSSTSVINYDIVMRQLDFLDHILVGEKPLRFNQHDNRLYIDMDWANDLQVGEYLVIDAYRKLDPDTYTDVYDDIWLKRYTTALVKKQWGANLSKFNGVAMIGGVTLNGQQIYSEALQDVEKLETEIRNSFELNPAMLIG

>AtlO_Ga0115011_10008698

MAQPNTRQTLISYAKRALGHPVIEINVDDDQIDDRVDEALQYWQQYHYDGIKRTYLKWQYTQAEKTRILTSNSEAGTKNSVTSTWKEDNNYIVVPETVFSVTNIFPFSNKGNLNLFDVRYQLRLNDLYDFSSTSVINYDVVMRQLDFLDHILVGEKPLRFNQHDNRLYIDMDWENDLMIDEYIVIECYRKMDPDTYTDVYNDIWLKKYTTALVKKQWGANLSKFAGVAMIGGVTLNGEQIYTQALADIEKLEEEIKSLQEHQAMLLG

>ArctO_Ga0115002_10003437_Split

MAKPASRENLKQYALRALGKPVIEINVDDDQMEDRLDEALQYFAQYHYDGVKRTYLKYKYTAEDKARALSQSSETASKTYGDSTTVSSTWQESNAYIIVPETVLSVVNIFPFSSKGNLNLFDVRYQLRLNDLYDFSSTSVINYDVVLRHLDFLDHILVGEKPMRFNQHDNRLYIDMDWKNDLQVDEFLVIECFRAVDPEQFTDVYNDLFLKKYVVALFKKQWGANLSKFDGVAMLGGVTLNGKDIYTQALDDIENLEQRIRSQYELNPTFMIG

>T.Oceans_St.37_SAMEA2619974_635

MAIPNSKSTLKDWCLRKLGAPVLEINVDDDQVDDRIDEALQFFYTFQYSGMERCYLKHLITEADVTRSGTDESETATDIKDSGITTDWKTGKGFLVMPDAVQSVLRVLPFSDRGNLNMFDVRYQLRLNDLYDFSSESVIHYQMTMWHLDYLDMILIGEKPLQFNTHKNRLYINMDWGDDVQVGEHIIIECYRKLDPTTWTDVYDNLWLKRYSTALIKKQWGENLIKFQGVTMLGGVTMNGETIYNDAKDTIIKLEEESKTTWEEPLHFDIG

>GROS21-1_SAMEA9560823_METAG-scaffold_19

MLGDGMIDVELDPKHYEIALDRALTRYRQRSPNAVEESYLFLELIQDVNEYRLPDEVIAVRQVFRRAIGSRSGIGAGGTLFEPFNLAYTNTYLMSGSMMGGLATYDAFAGYQKLVGRMFGSYIEFLWKPTTHLLTILQRPFAQGEQILIQSYNFRPDWVLLQDIYAKQWLKDYTLATCKLMLGEARSKFGTISGPSSPVTLNGTALQAAAKEEITNLDKELENLIAGGTGYYFITG*

>GROS21-1_SAMEA9560540

MSDPVPSTTQSNSTIERNKVFEFVRLMLGDGMVEVELDPAHYETALDRALTHYRARSSNSVEESYMFLELIQDQNEYRLPDEVITVRQVFRRAIGSRSGIGAGGTLFEPFNLAYTNTYLMSGSMMGGLATYDAFAGYQKLVGRMFGSYIEFLWKPTSHILDILQRPFAQGEQILIQCYNYRPDWVLLQDPYAKQWLRNYTLAICKQMLGQARSKFGSIAGPGSGGITLNGAALLSEAKEELVALDKEIDTYVAGGTPYTFVTG*

>GROS21-1_SAMEA9560542

MSNLDDAKQQVYDYVTAMLGGGMIDLELDPIHYQTALDRALNKYRQRGDSSVEESYMFLTLEIDVNTYTLPKEVVSVRQLFRRSIGSRSGLGNGGTVFEPFNMAYTNTYLLSSSNMGGLLTYEMYAQYQELVGRMFGSFINYNYNQSTRKLTITQRPRGEEEILIWTYNHRPDFVILEDVYANQWLKDYTLATCKMMMGEAREKFSQIAGPQGGGSLNGTAIKGEAKADIERLETELATQVTGGQGYTFIIG*

>GROS21-1_SAMEA9560546

MSDPVPSTTQSNSTIERNKVFEFVRLMLGDGMVEVELDPAHYETALDRALTHYRARSSNSVEESYMFLELIQDQNEYRLPDEVITVRQVFRRAIGSRSGIGAGGTLFEPFNLAYTNTYLMSGSMMGGLATYDAFAGYQKLVGRMFGSYIEFLWKPTSHILDILQRPFAQGEQILIQCYNYRPDWVLLQDPYAKQWLRNYTLAICKQMLGQARSKFGSIAGPGSGGITLNGAALLSEAKEELVALDKEIDTYVAGGTPYTFVTG*

>GROS21-1_SAMEA9560586

MSDPVPSTTQSNSTIERNKVFEFVRLMLGDGMVEVELDPAHYETALDRALTHYRARSSNSVEESYMFLELIQDQNEYRLPDEVITVRQVFRRAIGSRSGIGAGGTLFEPFNLAYTNTYLMSGSMMGGLATYDAFAGYQKLVGRMFGSYIEFLWKPTSHILDILQRPFAQGEQILIQCYNYRPDWVLLQDPYAKQWLRNYTLAICKQMLGQARSKFGSIAGPGSGGITLNGAALLSEAKEELVALDKEIDTYVAGGTPYTFVTG*

>GROS21-1_SAMEA9560612

MSDPVPSTTQSNSTIERNKVFEFVRLMLGDGMVEVELDPAHYETALDRALTHYRARSSNSVEESYMFLELIQDQNEYRLPDEVITVRQVFRRAIGSRSGIGAGGTLFEPFNLAYTNTYLMSGSMMGGLATYDAFAGYQKLVGRMFGSYIEFLWKPTSHILDILQRPFAQGEQILIQCYNYRPDWVLLQDPYAKQWLRNYTLAICKQMLGQARSKFGSIAGPGSGGITLNGAALLSEAKEELVALDKEIDTYVAGGTPYTFVTG*

>GROS21-1_SAMEA9560623

MSDSETTRQEVITYIKNMLGDGMVDIELDPHHYDTAINRALAKFRQRSQASTEESYGFMTMHQDVNEYTLAPEVMEVRQIFRRSIGSRSGGGDGGTIFEPFNLAYTNTYLLSSSNMGGLATYFAFASYQKLVGKMFGSEIDFLWNASTKKLTILQRPRGDETVLLWLYNHRPDFNLFTDPYAGIWLKDYALATCKIILGDAREKFATIASPQGGTQLNGTALKADGKAEIEALELDLINYKDGGKPLTFVIG*

>GROS21-1_SAMEA9560794

MCGDLKMDNLPSTPESNSTTERNIVFDYIRTMLGDGMVDMDLDPKHMETALDRALARFRQRSPNAVEESYLFLELIQDQNEYKLPDEVIEVRQVFRRAIGSRSGIGAGGTLFEPFNLAYTNTYLMSGSMMGGLATYELFSGYQKLVGKMFGSYIEFKWKPTTHTLTILQRPFAQGEQILVQSYNFRPDWVLLQDIYAKQWLKDYALATSKLILGQARSKFSTIAGPGSGIQLNGTALIAEAKEEIATLDKELENLVSGGTGYYFITG*

>GROS21-1_SAMEA9560823

MLGDGMIDVELDPKHYEIALDRALTRYRQRSPNAVEESYLFLELIQDVNEYRLPDEVIAVRQVFRRAIGSRSGIGAGGTLFEPFNLAYTNTYLMSGSMMGGLATYDAFAGYQKLVGRMFGSYIEFLWKPTTHLLTILQRPFAQGEQILIQSYNFRPDWVLLQDIYAKQWLKDYTLATCKLMLGEARSKFGTISGPSSPVTLNGTALQAAAKEEITNLDKELENLIAGGTGYYFITG*

>crystal_bog_phage_gp13

MVDIELDPEHYQNALNLSFDRYRQRSGASSEEAYMFLNLIYEQTDYTLPDEVVSVRQIFRRGLGETTGGTQLDPFSLAYTNLYLLQAGAGGGYTAGLLTFELFYQYLKQAGRMFGRDINYTFDPVTHKLSIIRKPTGDEAILLWVYKYKSDDHILSDPFSRPWIRDYTLAWCKMQLGEAYSKFNTVIGPQGGTTLKGDALKNEAKEIMDRLEKEIDLYIDSAMPLGIIIG
